# Artificial intelligence driven definition of food preference endotypes in UK Biobank volunteers is associated with distinctive health outcomes and blood based metabolomic and proteomic profiles

**DOI:** 10.1186/s12967-024-05663-0

**Published:** 2024-10-01

**Authors:** Hana F. Navratilova, Anthony D. Whetton, Nophar Geifman

**Affiliations:** 1 School of Biosciences, Faculty of Health and Medical Sciences, University of Surrey, Guildford, GU2 7XH UK; 2 Veterinary Health Innovation Engine, School of Veterinary Medicine, University of Surrey, Guildford, GU2 7AL UK; 3 Department of Community Nutrition, Faculty of Human Ecology, IPB University, Bogor, 16680 Indonesia; 4 School of Health Sciences, Faculty of Health and Medical Sciences, University of Surrey, Guildford, GU2 7YH UK

**Keywords:** Biomarkers, Food preferences, Latent Profile Analysis, Metabolomics, Proteomics, Relative risk, Unsupervised machine learning

## Abstract

**Background:**

Specific food preferences can determine an individual’s dietary patterns and therefore, may be associated with certain health risks and benefits.

**Methods:**

Using food preference questionnaire (FPQ) data from a subset comprising over 180,000 UK Biobank participants, we employed Latent Profile Analysis (LPA) approach to identify the main patterns or profiles among participants. blood biochemistry across groups/profiles was compared using the non-parametric Kruskal–Wallis test. We applied the Limma algorithm for differential abundance analysis on 168 metabolites and 2923 proteins, and utilized the Database for Annotation, Visualization and Integrated Discovery (DAVID) to identify enriched biological processes and pathways. Relative risks (RR) were calculated for chronic diseases and mental conditions per group, adjusting for sociodemographic factors.

**Results:**

Based on their food preferences, three profiles were termed: the putative Health-conscious group (low preference for animal-based or sweet foods, and high preference for vegetables and fruits), the Omnivore group (high preference for all foods), and the putative Sweet-tooth group (high preference for sweet foods and sweetened beverages). The Health-conscious group exhibited lower risk of heart failure (RR = 0.86, 95%CI 0.79–0.93) and chronic kidney disease (RR = 0.69, 95%CI 0.65–0.74) compared to the two other groups. The Sweet-tooth group had greater risk of depression (RR = 1.27, 95%CI 1.21–1.34), diabetes (RR = 1.15, 95%CI 1.01–1.31), and stroke (RR = 1.22, 95%CI 1.15–1.31) compared to the other two groups. Cancer (overall) relative risk showed little difference across the Health-conscious, Omnivore, and Sweet-tooth groups with RR of 0.98 (95%CI 0.96–1.01), 1.00 (95%CI 0.98–1.03), and 1.01 (95%CI 0.98–1.04), respectively. The Health-conscious group was associated with lower levels of inflammatory biomarkers (e.g., C-reactive Protein) which are also known to be elevated in those with common metabolic diseases (e.g., cardiovascular disease). Other markers modulated in the Health-conscious group, ketone bodies, insulin-like growth factor-binding protein (IGFBP), and Growth Hormone 1 were more abundant, while leptin was less abundant. Further, the IGFBP pathway, which influences IGF1 activity, may be significantly enhanced by dietary choices.

**Conclusions:**

These observations align with previous findings from studies focusing on weight loss interventions, which include a reduction in leptin levels. Overall, the Health-conscious group, with preference to healthier food options, has better health outcomes, compared to Sweet-tooth and Omnivore groups.

**Graphical Abstract:**

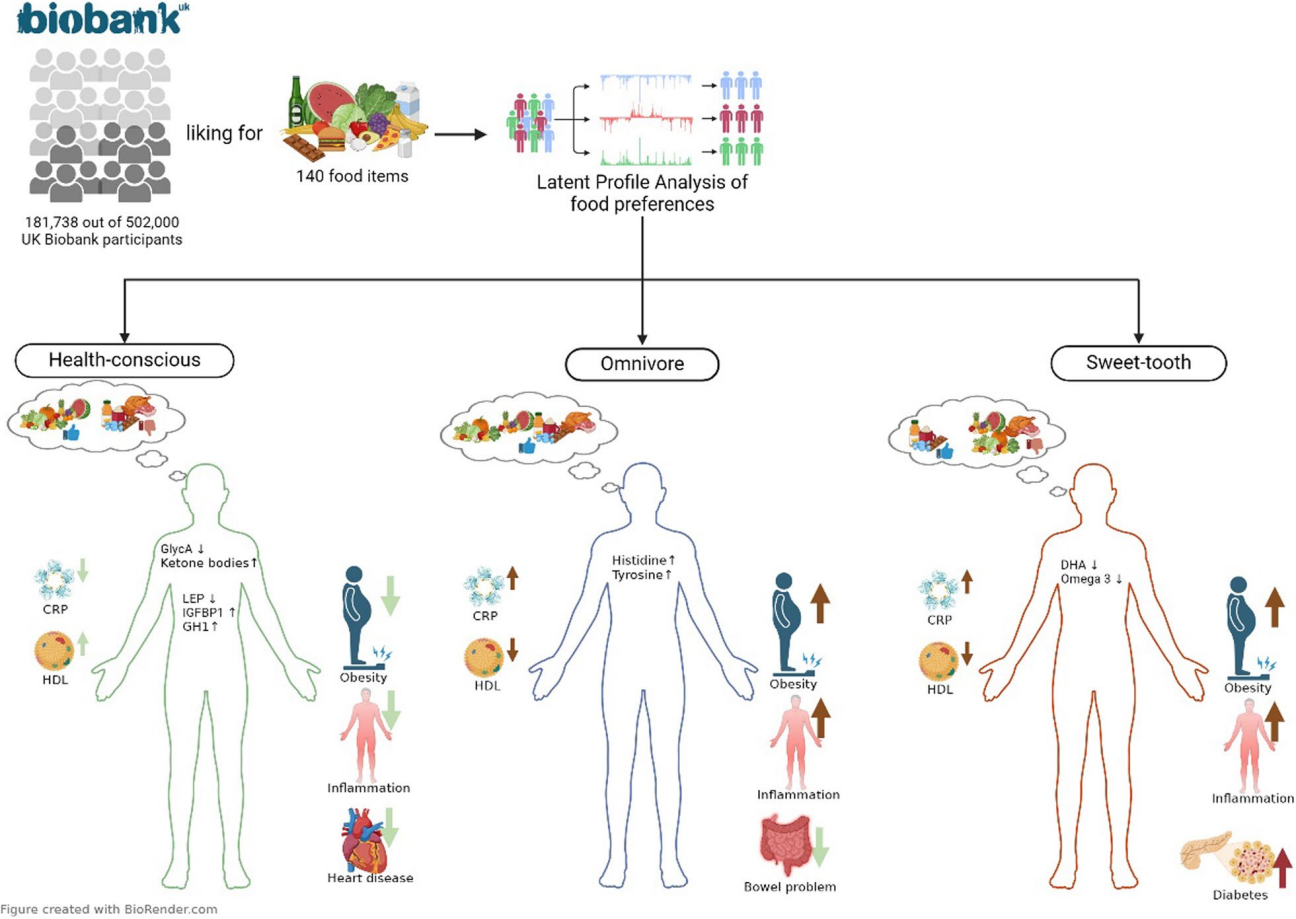

**Supplementary Information:**

The online version contains supplementary material available at 10.1186/s12967-024-05663-0.

## Background

Diet and nutrition significantly impact human health, but evaluating these relationships accurately poses challenges. Traditional methods, relying on self-reported data such as food frequency questionnaires or food recalls, are prone to inaccuracies and may lead to incorrect diet-disease associations. To mitigate this, dietary biomarkers based on metabolomics [1, 2] and proteomics [3] offer insights into food intake, metabolism, and overall diet quality. These biomarkers also shed light on the biological pathways linking dietary patterns to disease risk [4, 5].

An alternative method for assessing dietary patterns without relying on memory is the Food Preference Questionnaire (FPQ). This tool serves as a proxy for actual dietary intake, minimizing measurement errors. While some studies have explored associations between dietary patterns and blood/urinary protein and metabolite levels [6, 7], there remains a scarcity in research focused on examining the proteome and metabolome effects associated with food preferences. Given that food preferences are strongly associated with taste perceptions, investigations have leaned toward determining whether these preferences are influenced by genetic factors or environmental factors [8–10]. Existing research often emphasizes the link between sensory liking, genetic variations (such as SNPs), and disease risk [11–13]. Direct assessment of food preferences is less common.

Unsupervised machine learning or clustering approaches can be employed to identify subgroups of patients, for better stratification and improved association with biomarkers. These approaches are now widely used to identify strata within given diseases of interest [14–19]. Few studies have explored unsupervised machine learning methods for clustering populations based on their food preferences. Dimensional reduction techniques, such as Principal Component Analysis (PCA), are often employed to identify meaningful patterns. However, this method has some drawbacks compared to other methods that group data. These include the assumption of linear data structure, the possibility of unresolved highly correlated patterns due to uncorrelated principal components; the primary objective of PCA is maximizing variance rather than explicitly identifying clusters [20]. While a previous study has been carried out to identify dietary patterns derived from the Food Preference Questionnaire in UK Biobank samples using hierarchical clustering methods [21], this approach emphasized the collective preferences related to specific foods rather than individual variations or their associations with biomarkers.

**Fig. 1:**
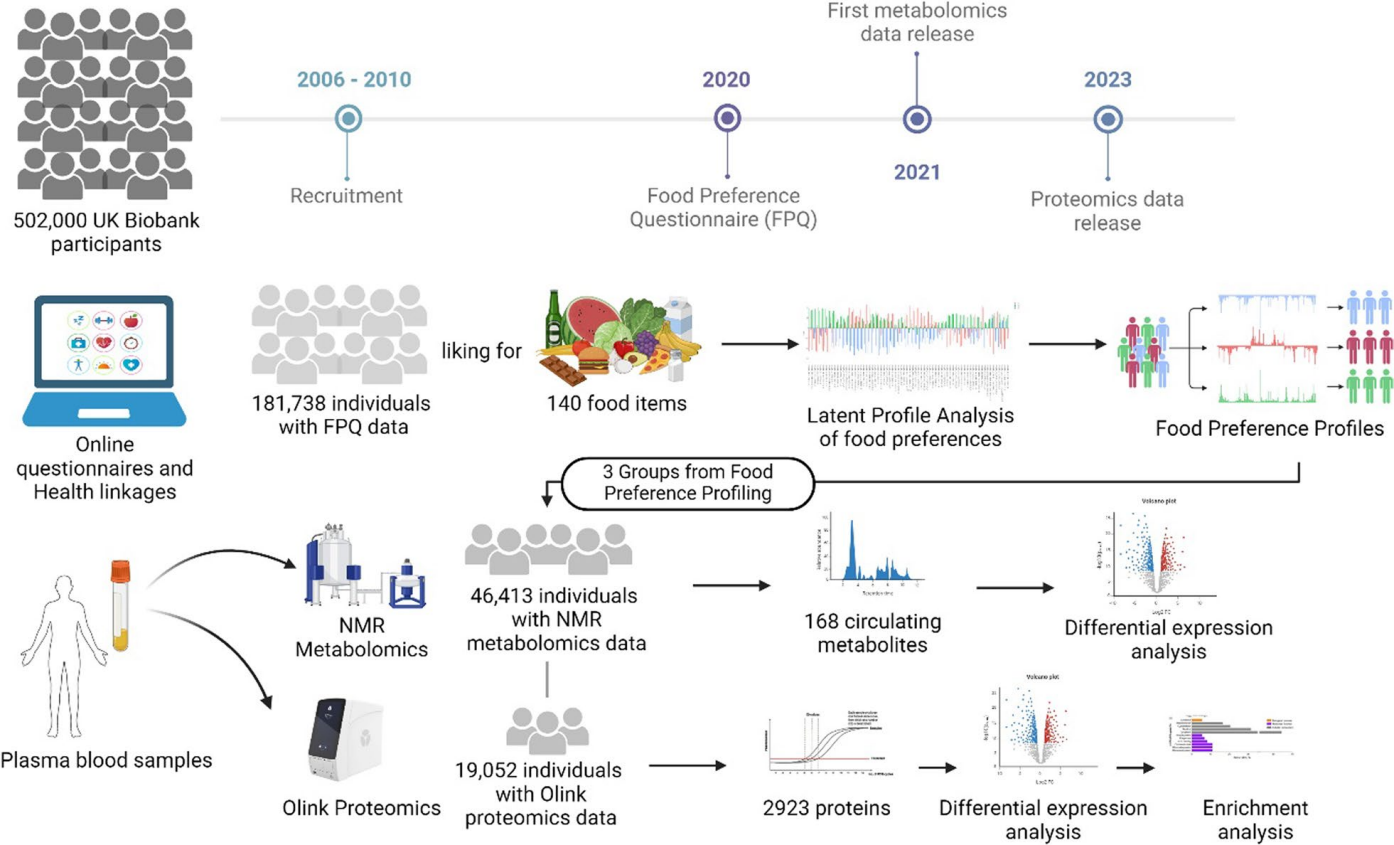
Schematic methodological approach. The figure illustrates the step-by-step process used in our study to identify food preference patterns and their biomarkers. Figure created with BioRender.com

Here, we employed latent class modelling, applied to categorical food preference questionnaire responses, to identify sub-populations within the UK Biobank, defined by their likes and dislikes of specific food types. By identifying meaningful food preference profiles we are able to assess associations with clinical outcome (such as risk of disease) as well as blood borne biomarker signatures. In other words, biomarkers can shed light on how food preferences impact individuals’ metabolic and proteomic status, or vice versa (see Fig. 1). We identify food preference profiles to explore whether these preferences lead to differences in blood metabolomics and proteomics profiles that pertain to disease risks. By leveraging machine learning techniques, our study aimed to provide fresh insights into the molecular mechanisms connecting food preferences to health outcomes.

## Methods

### Study population

Data for this study were obtained from the UK Biobank, which has recruited over 500,000 participants (aged 40–69 years), recruited between 2006 and 2010 from 22 assessment centres in the UK. Collected data includes touchscreen questionnaire (e.g. sociodemographic, lifestyle, health and medical history), and biological samples (blood, saliva, and urine). Health outcomes of the participants comes from both self-reported data collected at the UK Biobank assessment centre visits as well as linkage to electronic health records such as hospital inpatient records and primary care (GP). The protocols for the UK Biobank are overseen by The UK Biobank Ethics Advisory Committee (https://www.ukbiobank.ac.uk/ethics). For the current study, we used the UK Biobank Food Preference Questionnaire (Category ID 1039), NMR metabolomics data (Category ID 220), proteomics data (Table ID 1072) and ICD10 diagnoses (Field ID 41270). All field IDs used for extraction of data have been provided in Supplementary Data 1. We included only participants who completed the FPQ (*n* = 182,176). Participants who answered ‘prefer not to answer’ and/or ‘never tried’ greater than or equal to 25% were excluded (*n* = 438). A total of 181,738 participants were used for clustering analysis. Among these, for 46,413 participants NMR metabolomics data were available and for 19,052 participants Olink proteomics data were used.

### Assessment of food preference profile groups

Food preferences were collected through an online questionnaire comprising 152 items, covering foods and drinks, as well as additional non-food items that captured liking for health-related behaviours, such as physical activity. The questionnaire utilized a 9-point Hedonic scale, where 1 corresponded to “Extreme dislike” and 9 to “Extreme like.” Other options also included “Have never tried it” and “Prefer not to answer.” This survey was administered in 2019 to all UK Biobank participants who had agreed to be recontacted by the study. Of the 152 items, only 140 related to food and drink were retained for this specific study, while those referring to habits such as physical activity or watching TV were not included. Where participants selected “prefer not to answer” or “never tried” these were assigned the value of 0. The rationale for inputting the “never tried” answer as 0 for a 9-point Likert scale is to treat it as a separate category from the other response options, which range from 1 (strongly disagree) to 9 (strongly agree). This approach allows us to distinguish between respondents who have no opinion or experience regarding the statement or question and those who have some degree of agreement or disagreement. Ratings of preferences across questions were then transformed into z-scores for standardisation. Analysis was carried using the RStudio program (v4.3.3). The assessment was conducted in two phases. First, Exploratory Factor Analysis (EFA) was employed to discern the factorial structure of Food Preference Questionnaire responses within the UK Biobank, grouping foods based on similarities. This facilitated the creation of coherent food groups for easier interpretation. Subsequently, Latent Profile Analysis (LPA) was utilized to categorize participants according to their food preferences. LPA is particularly suited for FPQ data, offering greater adaptability in data distribution management and yielding more intelligible latent profiles compared to methods like k-means or hierarchical clustering.

#### Exploratory factor analysis

The R package *psych* (v2.4.3) was used to perform EFA. Kaiser-Meyer-Olkin (KMO) statistic was set to 0.70 for adequate sampling, ensuring the extraction of significant and consistent factors. The determination of factors were based on the scree plot breakpoint, where eigenvalues > 1, resulting in 19 initial factors (nfactor = 19) representing different food groups. Oblimin was used as the rotation to allows inter-factor correlations. Food items were then grouped based on factor loadings above 0.2, with items appearing in multiple groups assigned to the one with the highest loading. This method guaranteed accurate group associations. However, two groups (MR6 and MR19) were excluded post-extraction due to redundancy, reducing the total to 17 distinct food groups (Supplementary Table 2).

#### Latent profile analysis

To carry out LPA, the *mclust* package in R (v6.1) [22] was utilised. LPA is a statistical method that discerns distinct subgroups within a population by analysing observed variables. LPA is particularly suited for our research as it allows us to classify individuals into profiles based on their responses to the FPQ. We employed LPA to reveal latent profiles from indicator relationships, intentionally excluding covariates to capture the data’s inherent clustering more clearly. To determine the optimal number of profiles, we applied the Bayesian Information Criterion (BIC), evaluating models with 2 to 9 profiles. We selected the EEV (ellipsoidal, equal volume and shape) three-profile model that demonstrated the best fit without overfitting, as indicated by the BIC values, and each profile containing at least 10% of participants (supplementary Fig. 1). Participants were allocated to a specific profile (of the three) based on their highest posterior probability. After allocating the participants into profiles, we examined the weight of each food group from the EFA analysis contributed to main patterns. These loadings helped interpreting the profiles and assign meaningful names to those profiles.

### Statistical analysis

The characteristics of the study participants by their food preference group being compared including sociodemographic (age, sex, ethnic group, education background, Townsend deprivation indices, smoking status, IPAQ activity group), actual nutrient intake (energy, protein, fat, carbohydrate, total sugar, free sugar, dietary fibre, and saturated fatty acids), body composition (body weight, BMI, body fat percentage), and blood biochemistry (CRP, glucose, HbA1c, cholesterol, HDL, LDL, and triglycerides). Characteristics of ordinal or continuous variables were analysed using ANOVA or the Kruskal-Wallis test, the latter for datasets not adhering to a normal distribution. Subsequently, Dunn’s test was employed as a post-hoc analysis to conduct pairwise comparisons between groups, with adjustments made for multiple comparisons. Pearson’s Chi-squared test were used for testing categorical variables.

### Biomarker analysis

#### Metabolomics

Our analysis utilized UK Biobank-provided metabolomics data, obtained from high-throughput NMR profiling of plasma samples, encompassing a total of 249 metabolites, consists of 168 metabolites in absolute levels and 81 ratios derived from combinations of the original 168 measures. Only the 168 metabolites were included in the analysis here. Technical variation was eliminated using the R package ukbnmr (v2.2) [23]. The percentage of missing values for all metabolites was less than 5%, so all metabolites were included for further analysis. For absolute metabolite measures with missing data, these were imputed using half the minimum value for that metabolite. This approach helped us maintain the original spread and average of the dataset. To normalize all the metabolite measures, a natural logarithmic transformation (ln[x + 1]) was applied to the values of all metabolites. Finally, the obtained values were scaled using Pareto scaling (scaled by the square root of its median). A principal-component analysis (PCA) was conducted to examine association with the three food preference profiles (see supplementary Fig. 2). However, PCA revealed no clear separation between participants within each profile. Three-way differential abundance was visualized using a 3D cylindrical volcano plot created with the R package volcano3D v.2.0.9 [24]. Differences in metabolites across the three groups were calculated using a 3-class group test, either one-way ANOVA or Kruskal-Wallis test, depending on the data distribution. A likelihood ratio test was used to compare variation in metabolite abundance between food preference profile groups. This was followed by pairwise comparisons (either t-test or Wilcoxon test) between the groups. Multiple hypothesis testing was performed using the Benjamini-Hochberg false discovery rate (FDR), with an FDR cutoff set at q < 0.05. To further investigate individual metabolite-level differential abundance, analysis was performed using limma-trend method with the limma R package (v3.58.1) [25, 26]. A linear model was fit using the lmFit function for each metabolite where profile was set as independent variables as well as age and sex. Additionally, empirical Bayes smoothing was applied to the standard errors. Statistics for pair-wise abundance fold changes in metabolomics data were reported as the log 2 fold changes (logFC) of metabolites between two profiles. LogFC values exceeding ± 0.19 and FDR values < 0.05 were set as significant cut-off points.

#### Proteomics

We utilized proteomics data from the UK Biobank, which were generated using high-throughput Proximity Extension Assay technology [27]. This provided us with 2,923 unique protein measurements in Normalized Protein eXpression (NPX) format, indicative of relative quantity on a log-2 scale. NPX values were calculated as described previously [27]. Participants with > 25% missing values (*n* = 500) were excluded from the analysis. Additionally, two proteins with > 50% missing values (NPM1 and PCOLCE) were also excluded. Differential abundance analysis was performed using the limma-trend method. LogFC values exceeding ± 0.14 and FDR values < 0.05 were set as significant cut-off points. Functional enrichment analysis to identify enriched GO biological processes and KEGG pathways was performed pairwise across profiles using DAVID Bioinformatic Resources (https://david.ncifcrf.gov/tools.jsp)(28–32). GO terms and pathways with significant interaction were determined based on p-values, which were subsequently adjusted using the Benjamini-Hochberg False Discovery Rate (FDR) to obtain q-values.

### Disease risk

The status of chronic conditions was obtained from the UK Biobank inpatient hospital data, based on the International Classification of Disease, 10th revision (ICD-10). A total of 41 chronic and mental conditions related to multimorbidity (Supplementary Data 2) were selected. Relative risks (RRs) and 95% confidence intervals (CIs) were reported in this study. RRs were derived using a Poisson regression model, which calculated the incidence ratio of disease in one group relative to the other groups. The model adjusted for the following covariates: age, sex, BMI, ethnicity, physical activity level, smoking status, and Townsend deprivation index quintiles. We then converted the coefficient values to RR by exponentiation. The standard error of the relative risk was used to established a 95% CI for RR values. Significance was determined by two-tailed p-values, with a threshold of less than 0.05 indicating statistical significance.

## Results

### Identification of food preference profiles

The baseline characteristics of participants who completed the Food Preference Questionnaire (FPQ) were first compared with those of the entire UK Biobank participants (Supplementary Table 1). Overall, those who completed the FPQ had lower BMI, contained proportionally fewer current smokers, and were physically more active than the entire cohort. To explore the relationship between food items in the FPQ and groupings based on similarities or differences (i.e., factorial structure), we employed Exploratory Factor Analysis (EFA). This revealed seventeen food clusters (Supplementary Table 2), each representing a different factor (or “dimension”) in the data. The dimensions’ names were assigned according to the items clustered within the same factor, aiming to enhance readability and facilitate interpretation of the captured patterns of food preference. For example: Items such as baked/steamed fish, cod, fried fish, haddock, herring, mackerel, pollock, prawns, salmon, sardines, shellfish, smoked fish, and tinned tuna were grouped together and labelled as the ‘Fish/Seafood’ dimension; bitter ale, lager, red wine, spirits, whisky, and white wine were clustered and assigned the dimension name ‘Alcohol’; extra virgin olive oil, mayonnaise, salad dressing, soy sauce, tomato ketchup, and vinegar formed another cluster, which we labelled as the ‘Sauces/Condiments’ dimension. The analysis revealed that food types tended to group with other similar foods, suggesting data from the FPQs are robust.

**Fig. 2:**
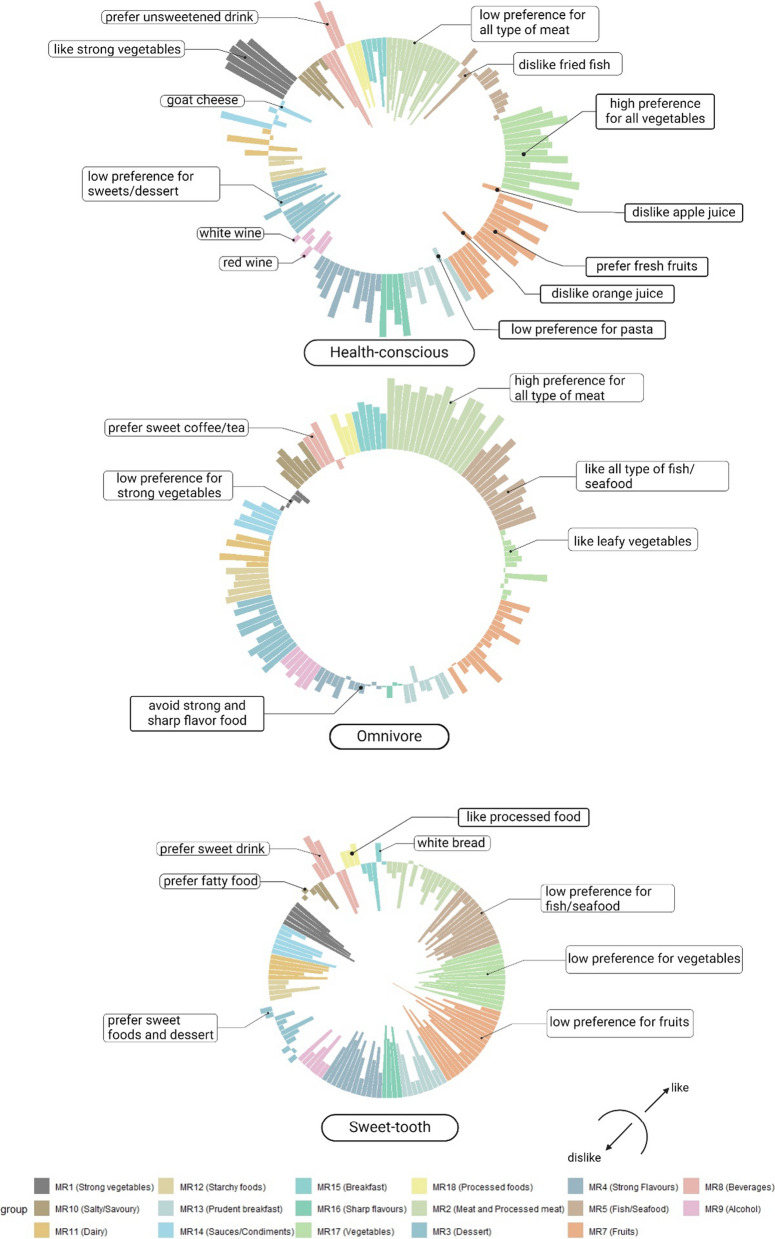
Food preference profiles are distinctly clustered by dietary choices and behaviours. Three food preferences profiles were generated through unsupervised machine learning method: putative Health-conscious, Omnivore, and putative Sweet-tooth. Bars toward outer circle represent high preference, bars toward inner circle represent low preference

Next, Latent Profile Analysis (LPA) was conducted to identify distinct groups of participants based on their individual food preferences. Using the Bayesian Information Criterion (BIC), and requiring at least 10% of participants in each resulting group, we selected the EEV (ellipsoidal, equal volume, and shape) model, resulting in three participant profiles as the optimal model. The three-profile model was taken forward for further analysis. The three profiles were then labelled as putative Health-conscious, Omnivore, and putative Sweet-tooth, based on their associations with related food preference variables (Fig. 2). The Health-conscious profile (*n* = 58,909) exhibited high preferences for defined healthy foods (as described in the literature), specifically fruits and vegetables. However, they had low preferences for meat, sweets, and fatty foods. The Omnivore profile (*n* = 72,286) showed preferences across almost all food groups. They had high preferences for meat and fish but avoided strongly and sharply flavoured foods. The Sweet-tooth profile (*n* = 50,543) had low preferences for all types of food except for sweetened beverages and sweet foods. These labels are intended to provide an immediate, intuitive description of each group’s dietary patterns, while the term ‘putative’ acknowledges that these are inferred profiles based on self-reported data.

### Overview of the characteristics of the food preference profiles

The baseline characteristics of the three groups are presented in Table 1. These groups showed significant differences in terms of age, sex, ethnicity, education, Townsend deprivation indices, smoking status, and IPAQ activity group. The average age was 56 ± 7 years for the Health-conscious group, 56 ± 8 years for the Omnivore group, and 55 ± 8 years for the Sweet-tooth group. These averages are similar with the overall UK Biobank cohort’s average age of 56 ± 8 years. While the Omnivore and Sweet-tooth groups had nearly equal proportions of females and males, the Health-conscious group had a higher proportion of females (71%, *p* < 0.001). Additionally, most participants were white (97% in the Health-conscious group, 98% in the Omnivore group, and 96% in the Sweet-tooth group), which limits the generalizability of the study to other ethnic groups. Examining healthy behaviours, the Health-conscious group had the lowest percentage of current smokers (5.2%, *p* < 0.001) and the highest percentage of individuals in the high IPAQ (International Physical Activity Questionnaire) activity group (49%). This suggests that a preference for healthy eating patterns may also reflect an overall healthy lifestyle in this group. The Health-conscious group has a higher proportion of vegetarians, but the proportions are overall relatively low (1.4%, 0.05%, and 0.7% in the Health-conscious group, the Omnivore group, and the Sweet-tooth group, respectively).

**Table 1:** Baseline characteristics for each food preferences profile

Characteristics	Health-conscious *n* = 58,909	Omnivore *n* = 72,286	Sweet-tooth *n* = 50,543	*p*-value
Age, years	56 (7)	56 (8)	55 (8)	< 0.001^a^
Sex				< 0.001^b^
Female	41,669 (71%)	36,024 (52%)	26,027 (49%)	
Male	16,956 (29%)	33,480 (48%)	27,581 (51%)	
Ethnicity				< 0.001^b^
White	56,565 (97%)	68,015 (98%)	51,326 (96%)	
Asian	733 (1.3%)	437 (0.6%)	866 (1.6%)	
Black	378 (0.6%)	330 (0.5%)	539 (1.0%)	
Mixed	334 (0.6%)	305 (0.4%)	311 (0.6%)	
Other ethnic group	416 (0.7%)	227 (0.3%)	325 (0.6%)	
Education				< 0.001^b^
None	2509 (4.3%)	5564 (8.1%)	4,772 (9.0%)	
College or University degree	31,946 (55%)	27,791 (40%)	21,541 (40%)	
O-levels/GCSEs or equivalent	9399 (16%)	15,377 (22%)	11,351 (21%)	
CSEs or equivalent	1,499 (2.6%)	3025 (4.4%)	2491 (4.7%)	
A levels/AS levels or equivalent	7663 (13%)	9482 (14%)	7191 (13%)	
NVQ or HND or HNC or equivalent	2055 (3.5%)	4017 (5.8%)	3193 (6.0%)	
Other professional qualifications	2993 (5.1%)	3570 (5.2%)	2518 (4.7%)	
Townsend deprivation indices				< 0.001^b^
Q1	22,082 (38%)	28,090 (40%)	19,386 (36%)	
Q2	12,729 (22%)	16,062 (23%)	11,705 (22%)	
Q3	10,057 (17%)	11,126 (16%)	9120 (17%)	
Q4	8,612 (15%)	8947 (13%)	7921 (15%)	
Q5	5,087 (8.7%)	5193 (7.5%)	5402 (10%)	
Smoking status				< 0.001^b^
Never	34,391 (59%)	40,315 (58%)	30,358 (57%)	
Previous	21,032 (36%)	24,192 (35%)	18,126 (34%)	
Current	3067 (5.2%)	4,853 (7.0%)	4,998 (9.3%)	
IPAQ activity group				< 0.001^b^
Low	7314 (14%)	11,372 (20%)	9409 (21%)	
Moderate	21,605 (43%)	25,112 (43%)	19,044 (42%)	
High	21,632 (43%)	21,763 (37%)	16,482 (37%)	
Body Mass Index (BMI, kg/m^2^)	25.65 (4.23)	26.97 (4.35)	27.07 (4.71)	< 0.001^a^
BMI class				< 0.001^b^
Underweight	493 (0.8%)	252 (0.4%)	278 (0.5%)	
Normal	27,977 (48%)	24,096 (35%)	17,870 (33%)	
Overweight	21,687 (37%)	30,596 (44%)	22,796 (43%)	
Obesity	8468 (14%)	14,560 (21%)	12,664 (24%)	

This table shows the number of people in each food preference group (Health-conscious, Omnivore, or Sweet-tooth) in a total sample of 181,738 participants with number for each group stated in the table header. Data are presented as mean (SD) for continuous data or number (%) for categorical data. The p-value indicates the probability of observing a difference in characteristics across groups by chance. A p-value < 0.05 (uncorrected for multiple testing) suggests that the difference is statistically significant. The p-value is calculated by ^a^ANOVA (two-sided); ^b^ Pearson’s Chi-squared test (one-sided)

**Fig. 3:**
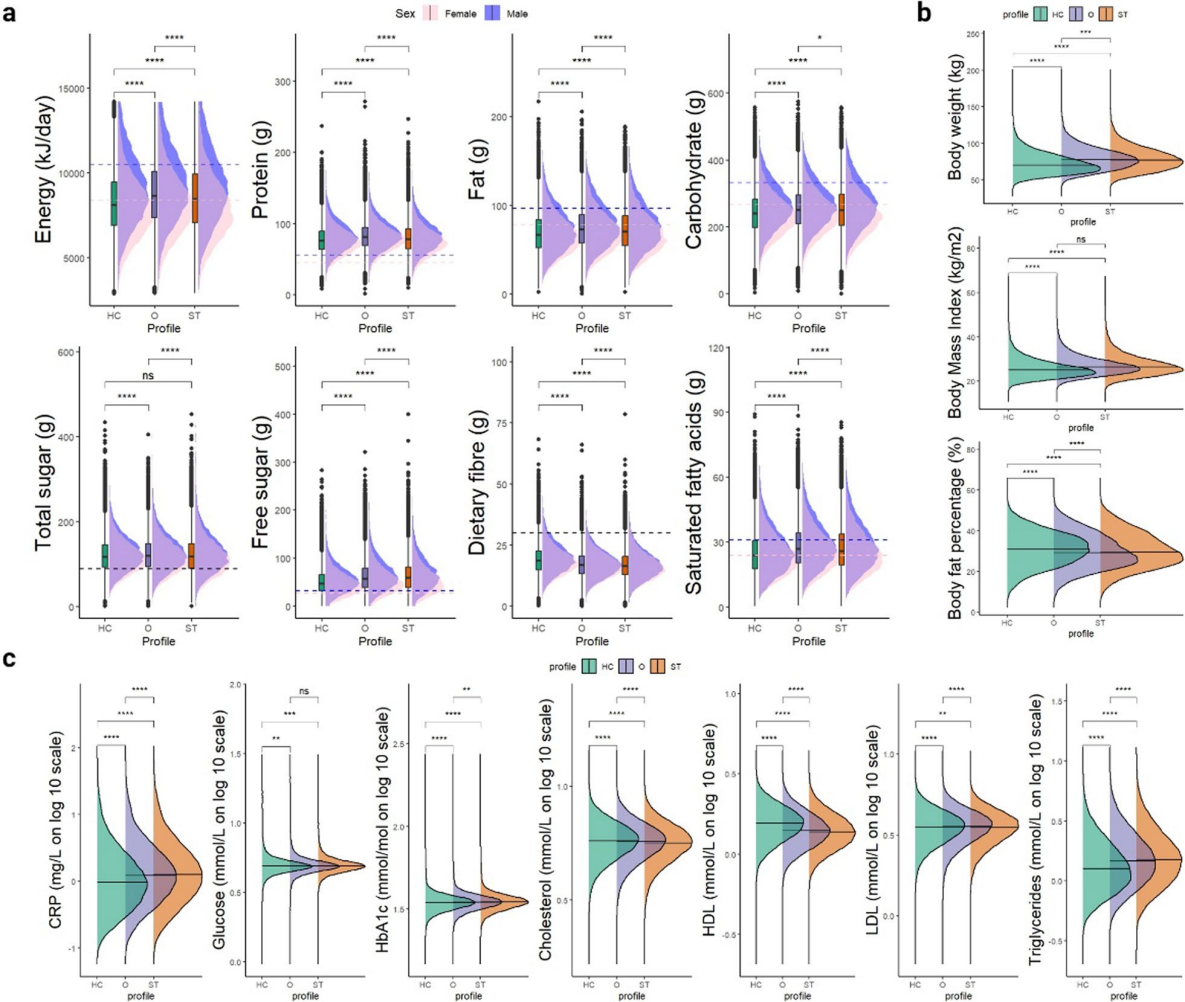
Characteristics dietary intake, body composition and blood biomarkers for each food preference profiles. **A** Boxplot for the average intake of nutrients across the three food preference profiles with a density plot delineates the variation in intake stratified by sex (pink for female, blue for male). Dashed lines refers to reference intake value based on UK Government Dietary Recommendations 2016. **B** Comparison of body weight, BMI, and body fat percentage among the three groups. **C** Blood biochemistry levels (in log 10 scale) associated with each food preference profile. HC = Health-conscious, O = Omnivore, ST = Sweet-tooth. The density plots in a, b and c indicate the median and distribution. Statistical analysis was performed using Kruskal–Wallis tests with Dunn’s test (post-hoc test). A one-sided p-value < 0.05 (uncorrected for multiple testing) suggests that the difference is statistically significance

We assessed the actual nutrient intake across the three groups and compared the differences using the Kruskal–Wallis rank sum test, focusing on participants whose values fell within 1.5 times the interquartile range (IQR) of the first and third quartiles (Q1 and Q3) to minimize effect of outliers (Fig. 3a). The Omnivore group exhibited the highest intake of nearly all nutrients, while the Health-conscious group had the lowest intake. The Health-conscious group’s preference for vegetables correlates with their significantly higher dietary fibre intake, which was evident with values of 19 ± 7 g/day, 18 ± 6 g/day, and 17 ± 6 g/day for the Health-conscious, Omnivore, and Sweet-tooth groups, respectively (*p* < 0.001). Notably, the Sweet-tooth and Omnivore groups’ intake of free sugars was substantially higher, with averages of 51 ± 28 g/day, 63 ± 34 g/day, and 65 ± 37 g/day for the Health-conscious, Omnivore, and Sweet-tooth groups, respectively (*p* < 0.001). Supplementary Table 4 provides the mean actual nutrient intake for each group. Furthermore, the Health-conscious group’s lower energy and macronutrient intake is also reflected in their BMI, which is significantly lower with a mean and standard deviation of 25.7 ± 4.2 (*p* < 0.001), compared to the Omnivore and Sweet-tooth groups, which have mean ± sd values of 27.0 ± 4.4 and 27.1 ± 4.7, respectively, as illustrated in Fig. 3b. Furthermore, given that the Health-conscious group predominantly consists of females, they exhibited a higher body fat percentage, with respective values of 30.9 ± 8.1%, 29.9 ± 8.4%, and 30.2 ± 8.6% for the Health-conscious, Omnivore, and Sweet-tooth groups (*p* < 0.001).

We next examined the levels of standard blood biochemistry tests measured during the participants’ initial UK Biobank assessment visit. The Health-conscious group exhibited the lowest levels of C-reactive Protein (CRP) (1.95 ± 3.7 mg/L, p value = < 0.001) and the highest HDL level (1.59 ± 0.40 mmol/L, p value = < 0.001) compared to the Omnivore group (2.32 ± 3.91 mg/L for CRP; 1.45 ± 0.37 mmol/L for HDL) and the Sweet-tooth group (2.47 ± 4.07 mg/L for CRP; 1.42 ± 0.37 mmol/L for HDL) (Fig. 3c and Supplementary Table 5). After analysing the blood biochemistry results of the three food preference groups, we investigated their medical history for any diagnoses of diabetes and vascular heart conditions (Supplementary Table 12) prior to food preference, metabolomic and proteomic data being collected. The data reveals that the Sweet-tooth group has the highest proportion of individuals with diabetes (4.1%, 3.0%, and 2.5% for Sweet-tooth, Omnivore, and Health-conscious groups, respectively), as well as varying rates of vascular heart conditions (4.2%, 3.7%, and 2.3% for Sweet-tooth, Omnivore, and Health-conscious groups, respectively). These suggest that pre-existing health conditions may contribute to the observed metabolic differences. Overall, the Health-conscious group demonstrated a more favourable blood lipid profile and lower levels of markers associated with inflammation.

### Association of food preference profile with plasma metabolite concentration

**Fig. 4:**
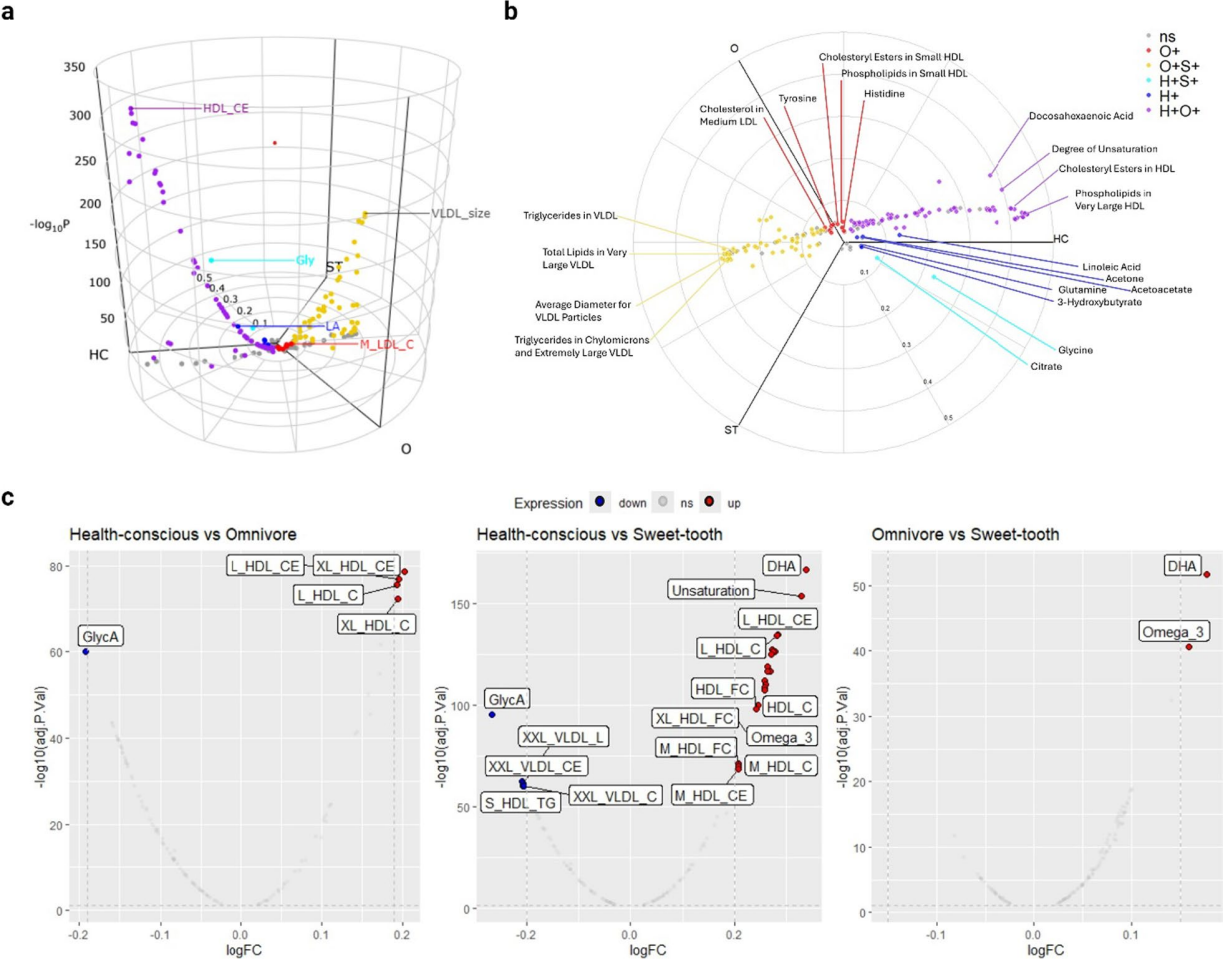
Differential metabolite abundance revealed by comparison of 168 circulating metabolites across the three food preference profiles. **a** Three-dimensional volcano plots for inter-group comparison were employed. Vectors for Pathotype Mean Z Score per metabolite are projected onto a polar coordinate space analogous to RGB (red-green-blue) colour space, mapped to HSV (hue-saturation-value). The Health-conscious, Omnivore, and Sweet-tooth groups are mapped to three axes: Health-conscious (HC), Omnivore (O), and Sweet-tooth (ST) using polar coordinates in the horizontal plane. The z-axis represents – log10 p-value for the likelihood ratio test. Metabolites with an adjusted p-value for the likelihood ratio test < 0.05 (z-axis) were considered significant (non-significant genes are coloured grey). Colours demonstrate pairwise comparisons (FDR < 0.05) between the three food preference profiles (Blue: Health-conscious (H+); Red: Omnivore (O+); Green: Sweet-tooth (S+)). Composite colours show genes significantly upregulated in two groups (Purple: Health-conscious + Omnivore (H + O+); Yellow: Omnivore + Sweet-tooth (O + S+); Cyan: Health-conscious + Sweet-tooth(H + S+)) (**b**) a lateral view and 2D polar plots of three-way comparisons for further visualization. **c** Volcano plot of differentially expressed metabolites using Limma. This plot illustrates the differential abundance of proteins between two groups: Health-conscious vs. Omnivore, Health-conscious vs. Sweet-tooth, and Omnivore vs. Sweet-tooth. Blue dots represent significantly differentially expressed metabolites (decrease), red dot represent significantly increased metabolites

We next compared the levels of metabolites across the three food preference profiles to gain a general overview of group differences. Using FDR-adjusted likelihood ratio tests and pairwise group comparisons, we investigated variations in 168 metabolites levels. The three-way volcano plots (Fig. 4a, b) shows that the Health-conscious group had six differentially expressed plasma metabolites (represented in blue). Among these, Linoleic Acid (LA) has the highest significance, followed by Glutamine (Gln) and ketone bodies (3-Hydroxybutyrate, Acetoacetate, and Acetone). In the Omnivore group (depicted in red), phospholipids and cholesteryl esters components in lipoprotein, total fatty acids, and two amino acids (Histidine (His) and Tyrosine (Tyr)) are at a higher level than in other groups. No metabolites are found to be up-regulated in the Sweet-tooth group alone. When combining the Health-conscious and Omnivore groups (depicted in purple), we observed up-regulation of free cholesterols and cholesterols as compared to the third group. The largest number of differentially expressed metabolites occurred between the Health-conscious and Omnivore groups (purple). In contrast, the Health-conscious and Sweet-tooth groups (cyan) revealed the fewest differentially expressed metabolites (specifically, Glycine (Gly) and Citrate). These results suggest that the Health-conscious and Sweet-tooth groups differ in their metabolic profiles. Supplementary Table 6 provides complete pairwise group tests for differential abundance.

After gaining initial insights through pairwise comparisons, we developed a comprehensive approach to decipher metabolome constituents and differences between groups. Specifically, we utilized Limma, a robust statistical framework, to investigate individual metabolite-level differential abundance. Our goal was to reveal subtle yet impactful changes across the entire metabolome. Notably, we found that Glycoprotein acetyls (GlycA) are expressed at lower levels in the Health-conscious group compared to both the Omnivore and Sweet-tooth groups. Additionally, two fatty acids—Docosahexaenoic acid (DHA) and Omega-3 fatty acids—were found to be lower in the Sweet-tooth group (see Fig. 4c). Taken together, these metabolomics results highlight distinct metabolic profiles associated with each food preference profile.

### Proteomic associations with food preference profiles

**Fig. 5:**
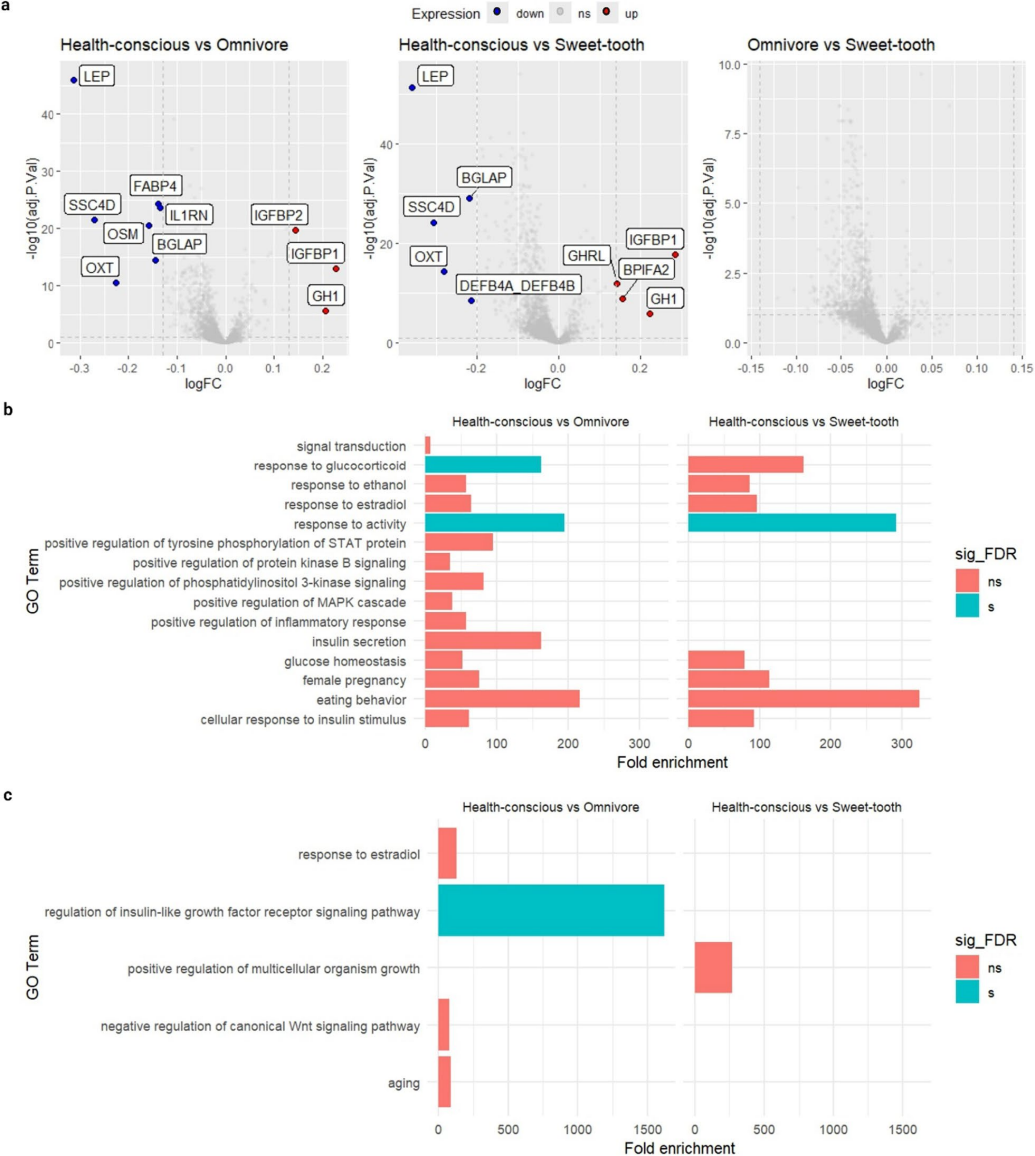
Food preference groups show differential abundance of 2923 proteins. **a** Volcano plot of differentially expressed proteins using limma. This plot illustrates the differential abundance of proteins between two groups: Health-conscious vs. Omnivore, Health-conscious vs. Sweet-tooth, and Omnivore vs. Sweet-tooth. Blue dot represent significantly decreased proteins, red dot represent significantly increased proteins. **b** The enrichment analysis revealed biological process that are significantly overrepresented with respect to proteins with lower abundance in the Health-conscious group compared to the Omnivores and Sweet-tooth groups. A higher fold enrichment score suggests a more pronounced overrepresentation of proteins within a specific pathway, highlighting distinct biological processes that are characteristic of the Health-conscious group. **c** This section identifies biological process GO terms enriched with target genes corresponding to proteins of increased abundance. Terms with significance interaction (P-value < 0.05) are shown. The blue bars indicate significance after multiple-testing correction (FDR < 0.05)

We then performed an enrichment analysis to determine underlying biological significance of proteins that have statistically significant association with food preference profiles. The differentially expressed proteins analysis revealed that LEP (leptin), BGLAP (osteocalcin/bone gamma-carboxyglutamate protein), SSC4D (scavenger receptor cysteine rich family member with 4 domains), and OXT (oxytocin/neurophysin I prepropeptide) are notably down in the Health conscious group data set, while IGBFP1 (insulin like growth factor binding protein 1) and GH1 (growth hormone 1) are up in levels in the Health-conscious group when compared to the Omnivore group and the Sweet-tooth group. We found no proteins are differentially expressed in the Omnivore group vs. Sweet-tooth group (Fig. 5a). OSM (oncostatin M), FABP4 (fatty acid binding protein 4), and IL1RN (interleukin 1 receptor antagonist) were only decreased in the Health-conscious compared to Omnivore. While GHRL (ghrelin and obestatin prepropeptide) and BPIFA2 (BPI fold containing family A member 2) were only up in the Health-conscious compared to Sweet-tooth.

The proteins we identified as differentially abundance have previously been associated with demographic factors such as age, sex, and BMI [27]. To further assess significance, we implemented multinomial logistic regression, adjusting for age, sex, and BMI (supplementary Table 9). The initial associations were slightly attenuated. Notably, the statistical significance changed for proteins such as OXT, FABP4, IL1RN, IGFBP1, GH1. Conversely, differences in GHRL, IGFBP2 and DEFB4A/DEFB4B (defensin beta 4 A/defensin beta 4B) were found to be no longer significant. These findings suggest that age, sex, and BMI play a role in modifying protein expression.

Enrichment analysis revealed Gene Ontology (GO) terms in specific biological processes (Fig. 5b, c). Amongst these terms were “Response to activity” (GO:0014823) in the Heath-conscious group. Additionally, we observed a decreased biological process related to “Response to glucocorticoid” (GO:0051384) in the Health-conscious group. However, this was significant only after multiple testing adjustment, when compared to the Omnivore group. This reduction in responsiveness to activity and glucocorticoids is coupled with heightened regulation of the insulin-like growth factor 1 receptor (IGF1R) signalling pathway (GO:0043567) in the Health-conscious group relative to the Omnivore group. Interestingly, this pattern is not observed in comparison to the Sweet-tooth group. In addition “glucose homeostasis” (GO:0042593) and “cellular response to insulin stimulus” (GO:0032869), emanated from the proteins modulated in the Health-conscious group compared to others and this then provides insights into carbohydrate-related metabolism differentials between the groups. Although not statistically significant after multiple testing adjustment, the GO term “eating behaviour” (GO:0042755) exhibits a high fold enrichment. This suggests a potential alteration in how the Health-conscious group responds to food cues or hunger signals which hints at metabolic adaptations, such as in energy utilization or nutrient intake adjustment. In the down-regulated protein comparison between Health-conscious and Omnivore groups, the KEGG pathway analysis initially revealed an association with Cytokine–cytokine receptor interaction. However, after applying the False Discovery Rate (FDR) correction, this pathway no longer remained statistically significant. Nevertheless, it is important to note that Cytokine-–cytokine receptor interaction plays a crucial role in immune responses and cell communication through cytokines and their receptors. Supplementary Table 10 gives the complete GO Term for enrichment analysis of biological processes, molecular functions, and cellular components, as well as KEGG pathways.

### Food preference links with disease

**Fig. 6:**
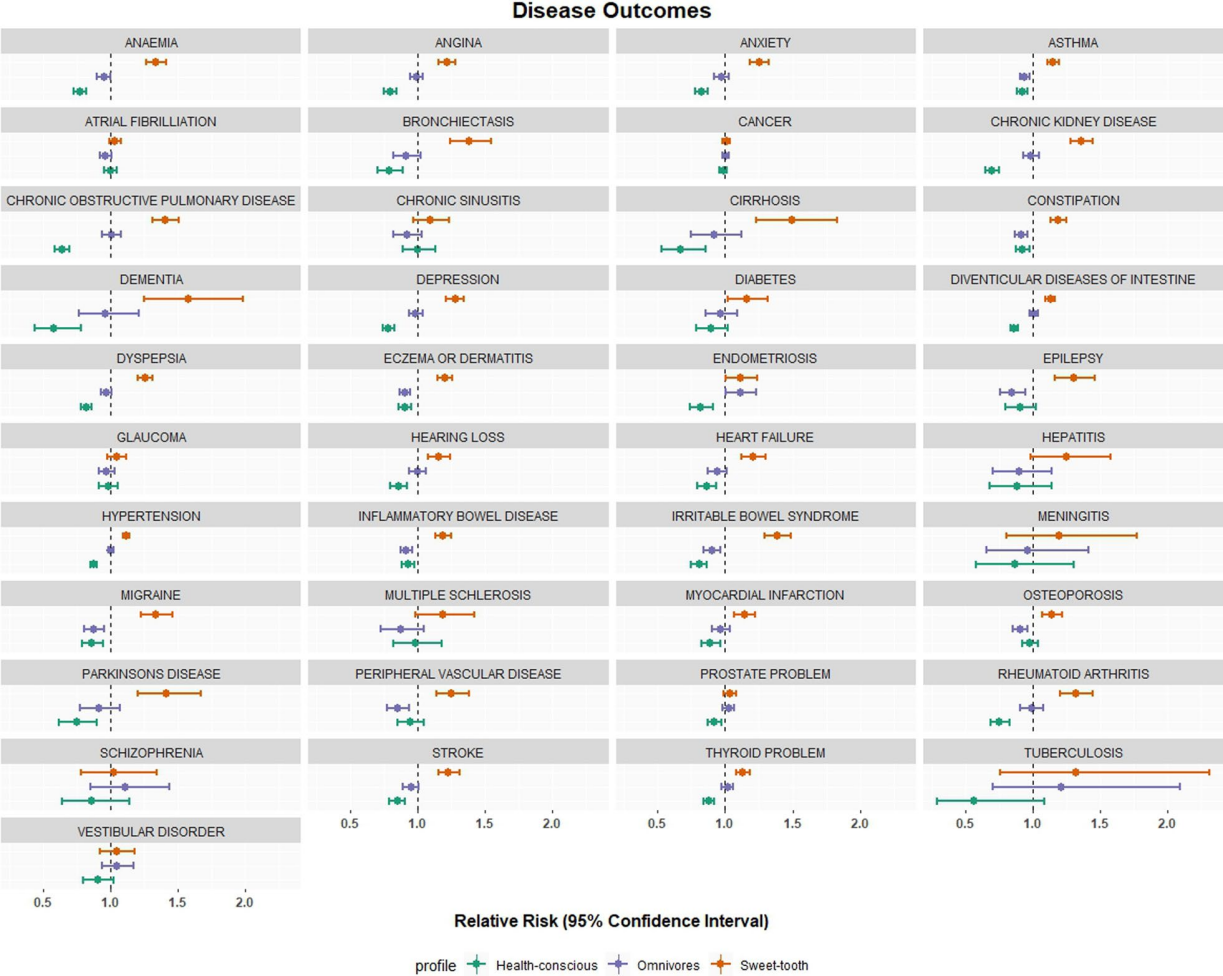
The relative risk (RR) of several disease outcomes related to multimorbidity within each profile. Dashed vertical line set at 1 indicates no association

To investigate whether being identified within one of the food preference profiles can predict association with disease, we assessed the link between each of the three groups and disease incidence during the UK Biobank observation period (median duration of follow-up = 14.4 years). The relative risk for disease outcomes indicates that, in general, those who are in the Health-conscious group have a reduced risk of having chronic diseases compared to those in the Omnivore and Sweet-tooth (Fig. 6). For example, those in Health-conscious group had 14% reduction in risk for heart failure (RR = 0.86, 95%CI 0.79–0.94, p-value ≤ 0.001), 30% reduction in risk for chronic kidney disease (RR = 0.70, 95%CI 0.65–0.75, p-value ≤ 0.001), and 15% reduction in risk for stroke (RR = 0.85, 95%CI 0.79–0.91, p-value ≤ 0.001) compared to those in the two other groups. Although not statistically significant after adjusting for sociodemographic factors, the Health-conscious group still exhibits a reduced risk for diabetes with a relative risk (RR) of 0.89 (95% Confidence Interval [CI]: 0.78–1.02, p-value = 0.09), compared to the Omnivore group (RR = 0.96, 95%CI 0.85–1.09, p-value = 0.56) and the Sweet-tooth group (RR = 1.16, 95%CI 1.02–1.32, p-value = 0.02). Depression showed 31% increase in risk for those in the sweet-tooth group (RR = 1.31, 95%CI 1.26–1.38, p value ≤ 0.001), and 22% reduction in risk for those in the health-conscious group (RR = 0.78, 95%CI 0.74–0.82, p value ≤ 0.001). However, infectious diseases such as hepatitis and tuberculosis, along with neurological conditions like meningitis, dementia, and schizophrenia, exhibit wide confidence intervals (CIs), indicating imprecise relative risk estimates. Furthermore, our observations reveal that cancer and glaucoma show minimal risk differences across the three groups, and while specific cancer types were not examined individually, this suggests complex factors beyond dietary influences, as previously documented [33]. In summary, the identified profiles provide valuable information on disease risk probabilities, particularly for metabolic-related diseases (Fig. 6).

## Discussion

In this study, we employed unsupervised machine learning to identify three novel groups based on their itemised food preferences: the Health-conscious group (with higher preference for vegetables and fresh fruit, and low preference for animal-based protein foods and sweet foods), the Omnivore group (high preference for all foods), and the Sweet-tooth group (high preference for sweet foods and drinks). Our analysis revealed that these distinct food preference profiles correlate with variable risk for different diseases, as well as specific metabolomic and proteomic features. These insights provide a deeper understanding of the underlying mechanisms connecting food preferences to health outcomes.

Prior studies have employed various machine learning methods to identify food liking patterns across different age groups [8, 10, 34, 35]. In a study by May-Wilson et al. [21], hierarchical factor analysis (HFA) revealed three food-liking traits (highly palatable, low caloric, and acquired food) among UK Biobank participants. These factors (or traits) where then used to determine association with genetic markers. In our study, we applied Latent Profile Analysis to identify subgroups of participants based on food preferences, later associating these groups with potential molecular biomarkers and health characteristics. LPA assumes categorical latent profiles and accommodates non-linear relationships between observed variables. However, it is important to acknowledge that the assumption of local independence, inherent in LPA, may be violated when dealing with correlated food factors. Despite this limitation, LPA remains a valuable method for identifying the optimal number of profiles (or groups) representing discrete clusters of participants with similar food preferences. Consequently, the profiles obtained through LPA are more interpretable as distinct subgroups with unique characteristics.

In our study of middle-aged volunteers, we considered Bawajeeh et al.‘s research on taste preferences among UK adolescents [36]. Their findings revealed that sweet foods (including snacks, desserts, sweetened beverages, dairy products, and fruit) and neutral foods (such as potatoes, bread, white fish, and select vegetables) dominate the diet of UK adolescents. Savory taste foods, including poultry products and flavored/spiced foods, followed closely. Interestingly, we observed a similar pattern in both the Sweet-tooth and Health-conscious groups in our study. However, they also found that consuming bitter-tasting non-Brassica vegetables (like spinach and kale) was associated with higher meat and high-protein food intake, which we did not observe in our Health-conscious group. This suggests that taste alone does not fully explain food preferences.

Research on food preferences among elderly individuals highlights the impact of structural factors such as education, income, social class, and access to quality healthcare [37]. Among the factors, only three factors (health, price, and mood) are statistically associated with healthy eating behaviour in elderly. The Health-conscious group is characterized by a female majority, this group has a lower smoking rate, engages in more physical activity, and has a lower BMI relative to the other two groups. Their lower degree of deprivation likely facilitates the adoption of a healthy lifestyle, unimpeded by economic constraints. Additionally, their higher educational attainment enable them to access, comprehend, and adopt health-related information effectively, further promoting their well-being. In our study, the Sweet-tooth group, associated with a lower socioeconomic status, aligns with prior research suggesting that participants in this socioeconomic category tend to care less about health and weight management when making food choices [38]. The Sweet-tooth group also has a higher proportion of obese individuals, possibly due to their preference for sugar. Studies consistently link liking for sweet foods to sugary drink consumption and refined sugars, rather than natural sweet foods like fresh fruit [39–41].

While debates continue about sugar versus fat as the primary cause of obesity, evidence points to sweetened beverages contributing to weight gain [42, 43]. Similar to the Health-conscious group, the Omnivore group predominantly comprises individuals from less deprived quintiles, enabling greater food access without budgetary constraints, likely resulting in increased consumption and, subsequently, a higher BMI than other groups. Furthermore, the higher BMI observed in this meat-preferring group corroborates previous research demonstrating a strong correlation between meat-liking and increased levels of BMI and fat mass [44]. Although this group enjoys vegetables and fruits as well, prior studies suggest that the preference for these foods has a weak association with BMI [45, 46]. This suggests that the preference for meat plays a more substantial role in contributing to higher BMI within the Omnivore group.

The differences in BMI observed across the food preference groups may influence their blood biochemistry profiles. Although the Sweet-tooth group’s blood glucose and HbA1c levels remain within normal ranges, they exhibit the highest levels among all profiles, likely due to their high intake of free sugars. In contrast, the Health-conscious group shows a more favourable blood lipid profile. Our findings align with Concas et al., who found that liking vegetables is associated with healthier serum lipid levels, including higher HDL cholesterol [44]. Moreover, the Health-conscious group showed reduced markers of inflammation, as indicated by their CRP level. Previous studies have shown that eating a reportedly healthy diet (e.g. DASH diet, Mediterranean diet), regardless of the macronutrient intake, is able to lower CRP [47, 48].

While routine blood analysis can reveal some differences among each profile, these differences are subtle. Therefore, it becomes essential to explore metabolomic and proteomic data to identify specific biomarker signatures corresponding to these profiles. In our study, the Omnivore group, characterized by a high preference for meat, exhibits elevated levels of histidine—a biomarker associated with meat consumption in previous research [49–51]. Furthermore, other studies have linked meat consumption to specific metabolites such as tyrosine [52, 53], fatty acids [51, 53], and glycoprotein acetyls [51, 53]. These findings align with our observations for both the Omnivore and Health-conscious groups.

Very little was found in the literature that directly investigates the relationship between food preferences and metabolomics. However, the study by Pallister et al. revealed associations between food liking patterns and metabolite levels [35]. They found that the Sweet and High Carbohydrate Food-Liking pattern was linked to low levels of Docosahexanoic acid (DHA). Interestingly, our research also observed lower abundance of DHA in the Sweet-tooth group, consistent with the existing literature. Additionally, similarities were evident in the Omnivore group in our study, aligning with the Meat-Liking pattern observed in their study, where amino acids were elevated. We have observed that the Health-conscious group has high levels of ketone bodies and Linoleic acid, and decreased Glycoprotein acetyl compared to the other two groups. However, previous research did not find blood metabolites associated with fruit and vegetable liking, possibly due to other influences such as environmental factors [35].

Unlike metabolomics, proteome changes occur more slowly and reflect longer-term adaptations. Through the biological processes identified via enrichment analysis, we found that carbohydrate utilization, insulin dynamics, and overall metabolic regulation are highly linked to food preferences. Additionally, anti-inflammation pathways emerge prominently in the analysis. In the down-regulated protein comparison between Health-conscious and Omnivore groups, the KEGG pathway analysis initially associated Cytokine-cytokine receptor interaction, though it lost statistical significance after FDR correction. This suggests that the Health-conscious group differs in these processes compared to the other two groups. Among the differentially expressed proteins, LEP (leptin), GH1 (growth hormone 1), IGFBP1 (insulin-like growth factor-binding protein 1), and IGFBP2 (insulin-like growth factor-binding protein 2) have been associated with a healthy dietary pattern [4, 5, 7, 54, 55]. In particular, LEP, GH1, and IGFBP1 appear to be key markers distinguishing the Health-conscious group. Their roles in metabolic processes include influencing appetite, energy expenditure, insulin sensitivity, and glucose regulation [56, 57]. Taken together with the metabolomics results for the Health-conscious, these data further support modulated abundance of biochemicals with a protective role against T2DM and reduced inflammation [58, 59].

This is borne out by the fact that when we examined the relative risk of disease, the Health-conscious group demonstrates a reduced probability metabolic-related conditions. Interestingly, the Health-conscious group exhibits features reminiscent of a fasting state: decreased leptin abundance, elevated growth hormone secretion, and increased ketone bodies. Beyond appetite regulation, fasting also influences energy metabolism, cellular repair, and stress adaptation. Studies shown beneficial effect of fasting for promoting overall health [60]. In a well controlled study in patients undergoing bariatric surgery, also known as weight loss surgery, leptin levels were shown to decrease with weight loss and the growth hormone system was also activated [61]. This direct experiment on weight loss indicates that the differences we see between the putative healthy eaters and other groups fit to endocrine differences related to adipose tissue. Perhaps, instead of opting for surgery or fasting, a shift toward healthier food choices could yield similar benefits. UK Biobank is in the process of recording abdominal adipose tissue levels using an imaging approach [62]. When sufficient data are collected we will compare adipose tissue parameters to leptin, growth hormone, IGF, IGFB levels and diet.

As food preference is often influenced by liking certain types of flavours, many studies attempted to classify dietary patterns based on taste preference. Several studies suggest that a preference for high-fat foods and meat is associated with greater adiposity and blood pressure [11, 44]. Liking fatty foods was also found to increase the odds ratio for metabolic syndrome [63]. Similar findings, albeit with a weaker correlation, show an association of liking for fat and salt with risk of cardiometabolic diseases [13]. Thus, food liking may be a useful predictor of health outcomes. In our study, the Sweet-tooth group not only has the highest proportion of individuals previously diagnosed with diabetes and other vascular health conditions, they also possess a higher risk of non-communicable diseases (NCDs) compared to the Omnivore and the Health-conscious groups. These results reflect those of Concas et al. [44] who found that individuals with higher blood pressure have a low liking for fish, one of the Sweet-tooth group characteristics. While the current study found some links between food preference and health status, whether people who prefer healthy foods developed this preference as a way of managing previously diagnosed disease remains unclear.

The present study yields significant findings in at least two key aspects. First, unsupervised machine learning clustering effectively identifies distinct and interpretable food preference profiles. These profiles, in turn, reveal multi-omics derived markers associated with dietary choices. Second, our study pioneers the direct link between food preferences and disease risk with a link to biochemical differences and biochemical pathways, inclusive of leptin, GH1 and IGFBP. However, we acknowledge several limitations in our research. Foremost among them is the lack of validation or verification in other independent studies. This is in part due to the unique nature of UK Biobank at present. Validation studies can be undertaken when appropriate datasets are available. Second, while recall bias is not a concern in the food preference questionnaire, it is still susceptible to social desirability bias when completing the questionnaire. Thirdly, although we accounted for confounding variables in the biomarker and health outcomes analysis, it remains possible that residual confounding still exists. Lastly, although questions often raised about UK Biobank’s generalizability and selection bias, given the relative magnitude and the diversity of its exposure measurements, data from UK Biobank still yield reliable and important insights regarding the relationship between environmental factors and health outcomes.

## Conclusions

Our study leveraged unsupervised machine learning to categorize food preferences and identify new meaningful population strata, revealing potential implications for health. Despite not directly observing actual food intake, blood metabolomics and proteomics can reveal biomarkers linked to dietary pattern. Food preferences alone provided a glimpse into metabolic disease risk probabilities. Although our results have highlighted interesting and relevant associations, they do not confirm causality between food preferences and health outcomes. The potential influence of genetic factors on both food preferences and chronic diseases, while not the focus of this study, should not be disregarded. This research implies that integrating food choice into personalized chronic disease management is a promising avenue. Further longitudinal and experimental studies, as well as studies in other populations outside the UK, need to be carried out to establish causality which will be essential for confirming the influence of dietary preferences on health promotion and disease prevention.

## Supplementary Information

Supplementary Material 1.

Supplementary Material 2.

Supplementary Material 3.

Supplementary Material 4.

## Data Availability

The UK Biobank database is accessible to researchers upon successful application through the UK Biobank website. Our study utilized the UK Biobank under research application number 83988. Supplementary Data 1 contains all field IDs used for data extraction. For individual-level sensitive data from UK Biobank data, descriptions of input file formats alongside the code used to run the analysis is provided. Additionally, links to publicly-available datasets used in our analyses are provided on the GitHub repository (https://github.com/hanavratilova/fpq_omics.git). All analyses were performed on publicly available software, and all parameters are provided in methods wherever relevant. The code used for this study was tailored to the UK Biobank data and is no use as a standalone without access to the UK Biobank.

## Abbreviations

BGLAP: Osteocalcin/bone gamma-carboxyglutamate protein
BIC: Bayesian Information Criterion
BMI: Body Mass Index
BPIFA2: BPI fold containing family A member 2
CI: Confidence Interval
CRP: C-reactive Protein
DEFB4A/DEFB4B: Defensin beta 4 A/defensin beta 4B
DHA: Docosahexaenoic acid
EFA: Exploratory Factor Analysis
FABP4: Fatty acid binding protein 4
FDR: False Discovery Rate
FPQ: Food Preference Questionnaire
GH1: Growth hormone 1
GHRL: Ghrelin and obestatin prepropeptide
Gln: Glutamine
Gly: Glycine
GlycA: Glycoprotein acetyls
GO: Gene Ontology
HDL: High Density Lipoprotein
His: Histidine
IGBFP1: Insulin like growth factor binding protein 1
IGBFP2: Insulin like growth factor binding protein 2
IGF1R: Insulin-like growth factor 1 receptor
IL1RN: Interleukin 1 receptor antagonist
IPAQ: International Physical Activity Questionnaire
KMO: Kaise-Meyer = Olkin
LA: Linoleic Acid
LEP: Leptin
LPA: Latent Profile Analysis
OSM: Oncostatin M
OXT: Oxytocin/neurophysin I prepropeptide
PCA: Principal Component Analysis
RR: Relative Risk
SNP: Single-nucleotide Polymorphism
SSC4D: Scavenger receptor cysteine rich family member with 4 domains
T2DM: Type 3 Diabetes Mellitus
Tyr: Tyrosine
