# Supplementary material for: Artificial intelligence driven definition of food preference endotypes in UK Biobank volunteers is associated with distinctive health outcomes and blood based metabolomic and proteomic profiles
Source: J Transl Med. 2024 Oct 1;22:881. doi: 10.1186/s12967-024-05663-0 (PMC11443809; doi:10.1186/s12967-024-05663-0)
Supplement: Supplementary file 1 — Supplementary Material 1. [file 12967_2024_5663_MOESM1_ESM.docx]

Supplementary information for

Artificial intelligence driven definition of food preference endotypes in UK Biobank volunteers is associated with distinctive health outcomes and blood based metabolomic and proteomic profiles

Supplementary Figures

Supplementary Fig.1


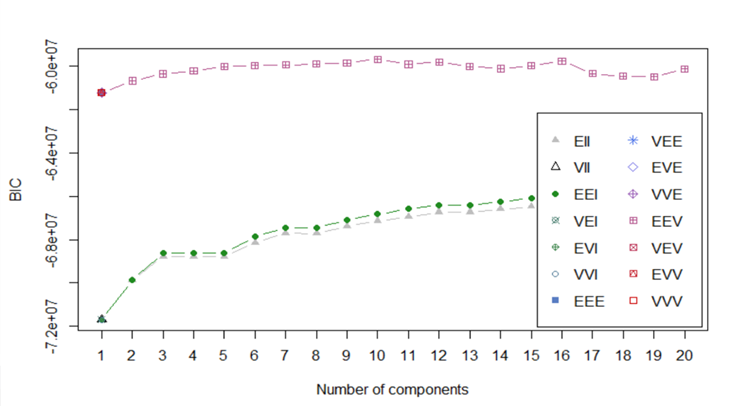


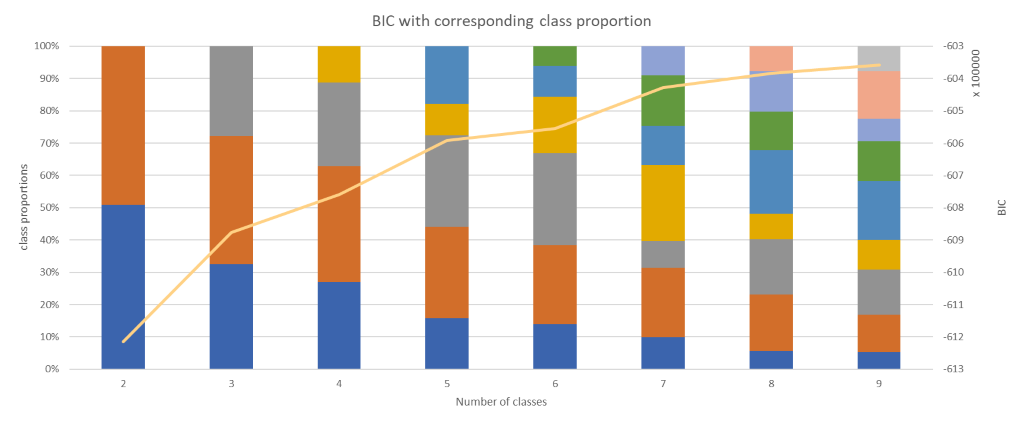


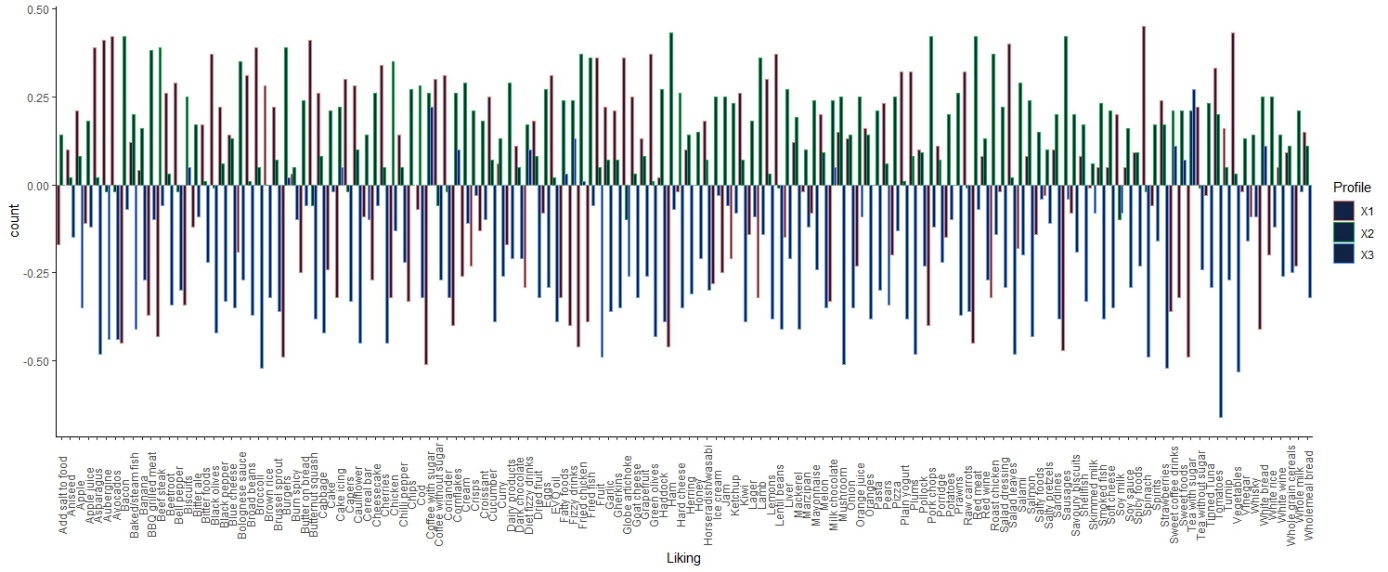


Supplementary Fig 1: Latent Profile Analysis. 1A) BIC plot for 20 classes, the Bayesian Information Criterion (BIC) values for different numbers of latent classes (ranging from 1 to 20). The x-axis represents the number of classes, and the y-axis represents the BIC score. 1B) BIC plot of selected model (EEV), BIC as a function of number of classes (line) with corresponding class proportions (bars). 1C) Histogram of LPA class (3 Groups), it illustrates the distribution of individuals across three latent classes (X1, X2, and X3). Each bar represents a class, with the red bar corresponding to X1, the blue bar to X2, and the green bar to X3. The y-axis represents the mean score for each group, and the x-axis lists the food items associated with the latent classes.

Supplementary Fig.2


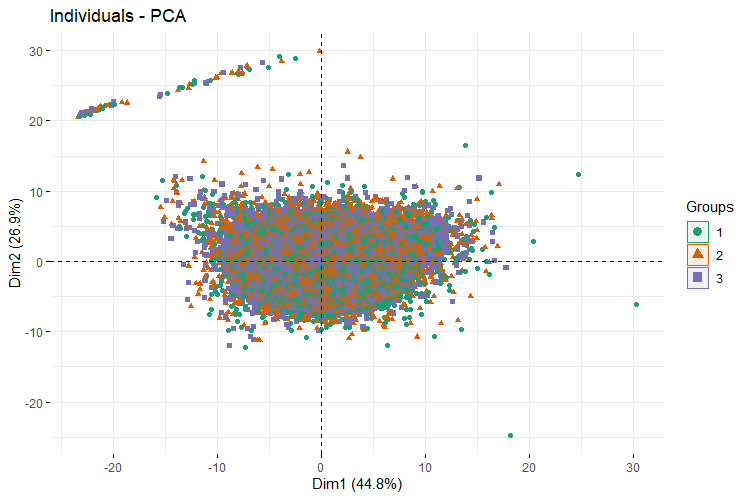


Supplementary Fig 2: PCA for metabolites of each profiles. The figure illustrates the application of PCA to metabolomic data from three distinct profiles (X1, X2, and X3). Each dot represents a participant, and the position of the dots in the PCA plot reflects their overall metabolite profiles. Green circle represent participants belongs to X1, Red triangle represent participants belongs to X2, Purple square represent participants belongs to X3.
