# Supplementary material for: Artificial intelligence driven definition of food preference endotypes in UK Biobank volunteers is associated with distinctive health outcomes and blood based metabolomic and proteomic profiles
Source: J Transl Med. 2024 Oct 1;22:881. doi: 10.1186/s12967-024-05663-0 (PMC11443809; doi:10.1186/s12967-024-05663-0)
Supplement: Supplementary file 2 — Supplementary Material 2. [file 12967_2024_5663_MOESM2_ESM.pdf]

Supplementary information for

Artificial intelligence driven definition of food preference endotypes in UK Biobank volunteers is associated with distinctive health outcomes and blood based metabolomic and proteomic profiles

INDEX

Supplementary Table 1. Study population characteristics of whole UK Biobank cohort and participants with FPQ
Supplementary Table 2: Exploratory Factor Analysis (EFA) results
Supplementary Table 3: Dimension of FPQ items based on Exploratory Factor Analysis
Supplementary Table 4: Actual daily nutrient intake for each food preference groups
Supplementary Table 5: Blood biochemistry and blood count for each food preference groups
Supplementary Table 6: pairwise group tests for metabolomics differential expression for Volcono3D plot
Supplementary Table 7: Limma differential expression for metabolomics data
Supplementary Table 8: Limma differential expression for proteomics data
Supplementary Table 9: Multinomial logistic regression for differentially expressed protein adjusted for age, sex and BMI
Supplementary Table 10: GO Term from Enrichment analysis of differentially expressed protein
Supplementary Table 11: Relative Risk of chronic diseases
Supplementary Table 12: Medical History, medication, and supplement taken for each food preference groups
Supplementary Data 1: UK Biobank Field ID
Supplementary Data 2: List of the 41 chronic conditions included in the definition of multimorbidity and its ICD10 code use for analysis.

Supplementary Table 1. Study population characteristics of whole UK Biobank cohort and participants with FPQ

Characteristics	Total UK Biobank (n=502,389)	FPQ (n=182,176)	p-value
**Age (median)**	58 (50, 63)	57 (50, 62)	<0.001[a]
**Gender**			<0.001[b]
Male	273,325 (46%)	78,017 (43%)	
Female	273,086 (54%)	103,720 (57%)	
**Ethnicity**			<0.001[b]
White	472,612 (95%)	175,906 (97%)	
Mixed	2,335 (0.5%)	954 (0.5%)	
Asian or Asian British	9,836 (2%)	5 (0.003%)	
Black or Black British	3,543 (0.7%)	1,254 (0.7%)	
Chinese	1,573 (0.3%)	420 (0.2%)	
Other ethnic group	4,557 (0.9%)	968 (0.5%)	
**Body Mass Index (BMI)**			<0.001[b]
Underweight	2,626 (0.5%)	1,023 (0.6%)	
Normal	162,371 (33%)	69,943 (38%)	
Overweight	212,078 (42%)	75,079 (41%)	
Obese	122,229 (24%)	35,692 (20%)	
**Smoking status**			<0.001[b]
Current	52,960 (11%)	12,958 (7%)	
Previous	173,007 (34%)	63,498 (35%)	
Never	273,449 (54%)	105,293 (58%)	
**IPAQ activity group**			<0.001[b]
low	76,190 (15%)	28,211 (15%)	
moderate	163,992 (33%)	65,885 (36%)	
high	162,102 (32%)	59,980 (33%)	

Note: Data are presented as median (IQR) for continuous data or number (%) for categorical data. The p-value indicates the probability of observing a difference in characteristics between groups by chance. A low p-value (<0.05) suggests that the difference is statistically significant. The p-value is calculated by [a] Wilcoxon rank sum test; [b] Pearson's Chi-squared test.

Supplementary Table 2: Exploratory Factor Analysis (EFA) results

**item**	**MR2**	**MR5**	**MR7**	**MR3**	**MR4**	**MR9**	**MR16**	**MR12**	**MR1**	**MR14**	**MR11**	**MR10**	**MR8**	**MR15**	**MR13**	**MR17**	**MR18**
Add salt to food	0.03	0.01	0.03	-0.03	-0.01	-0.01	0	0.03	-0.01	0.05	0	0.53	0.14	-0.08	-0.06	0.09	-0.03
Aniseed	0	0	0.1	0.08	0.13	0.02	0.01	0.07	0.06	0.06	-0.03	0.16	-0.01	0.14	0	-0.01	-0.03
Apple juice	0	0.03	0.13	0.11	-0.07	-0.04	0.03	-0.02	0.05	0.06	0	-0.04	0.09	0.14	0.41	0.03	-0.04
Apple	0.03	0.01	0.5	-0.03	0	0.03	-0.02	0.01	-0.06	-0.04	0	0.02	-0.03	0.13	0.13	-0.01	-0.01
Asparagus	-0.01	0.05	0.05	-0.01	-0.03	0.23	0.16	0.07	0.41	0.02	0.03	0.02	-0.04	-0.05	0.01	-0.01	-0.04
Aubergine	-0.04	0	0.04	-0.01	0.05	0.1	0.2	0.01	0.45	0.02	0.02	0.02	-0.06	0.06	0.01	0.01	-0.08
Avocados	-0.06	0.02	0.09	-0.02	-0.03	0	0.26	0.03	0.45	0.07	0.04	0	-0.05	0	-0.03	0.03	-0.02
Bacon	0.73	0.01	-0.02	0.04	-0.02	-0.02	-0.02	0.02	-0.07	0.01	0.06	0.1	0	-0.03	0.02	-0.02	-0.03
Baked/steam fish	-0.01	0.78	0	0	-0.02	0.08	-0.01	-0.01	0.05	-0.03	-0.01	0	-0.01	0.06	-0.03	0	0.03
Banana	0	0.01	0.36	0.04	0	0.02	0.01	0.02	-0.03	0.04	-0.02	-0.09	0.03	0.14	-0.02	0.08	0.08
BBQ grilled meat	0.78	-0.01	0.02	-0.02	0.07	-0.03	-0.01	0.02	0.08	0	-0.01	0.04	0.01	-0.01	0.02	-0.02	0.08
Beef steak	0.8	-0.06	0.03	-0.03	0.02	0	-0.02	0.09	0.04	-0.03	-0.02	0.01	0.01	-0.05	0.01	0.06	0.02
Beetroot	-0.04	0	0.14	0.01	-0.04	0.29	0.07	0.01	-0.1	0.26	-0.01	-0.01	-0.02	0.07	-0.08	0	0.01
Bell pepper	0	-0.02	0.07	-0.03	0.28	0.08	0.03	-0.01	0.22	0.06	0.01	0.09	-0.05	0.06	-0.08	-0.05	-0.03
Biscuits	0.01	0.01	0.01	0.63	-0.03	0	-0.01	0.03	-0.06	-0.01	0	0.06	0.01	0.03	0.03	-0.01	0.05
Bitter foods	0.01	0.02	-0.02	-0.1	0.25	0.05	0.03	0.09	0.02	0	0.02	0.22	-0.12	0.08	0.17	0.01	-0.1
Bitter ale	0.03	0.04	-0.1	-0.07	0.09	-0.01	0.08	0.51	-0.11	-0.09	-0.03	-0.02	0	0.09	0.16	0.09	-0.05
Black olives	-0.01	-0.01	0.01	0.01	0	0	0.91	0.01	0.01	-0.02	0.01	-0.01	0	0	-0.01	0	0
Black pepper	0.01	0.02	0.04	-0.03	0.39	0.07	0.05	0.06	0.01	0.11	0.02	0.07	-0.03	0.03	-0.03	-0.02	-0.15
Blue cheese	0.01	0.05	-0.02	-0.05	0.06	0.02	0.23	0.13	-0.04	-0.01	0.45	0.02	-0.01	-0.03	0.01	-0.01	-0.06
Bolognese sauce	0.46	0	-0.02	0.05	0.12	-0.01	-0.01	-0.01	0.07	0.18	0.09	-0.07	0	0.07	-0.01	-0.11	-0.04
Broad beans	-0.01	0.04	0.07	-0.04	0.01	0.31	0.13	0.05	0.05	-0.06	0.03	0.03	-0.02	0.13	0.03	0.03	-0.12
Broccoli	-0.01	0.05	-0.01	0.02	-0.02	0.64	0.01	-0.01	0.14	-0.04	0.02	-0.04	-0.02	0.04	0	-0.06	0.02
Brown rice	-0.02	0.04	0.07	-0.02	0.12	0.09	-0.02	0.02	0.13	-0.02	0.05	-0.01	-0.03	0.35	0	-0.11	-0.06
Brussel sprout	0.01	0.01	-0.07	0.04	0	0.7	0.03	0.02	-0.06	-0.02	0	-0.03	-0.01	-0.04	0.04	0.03	0.01
Burgers	0.59	-0.04	-0.09	0.03	0.07	-0.04	0	0.02	0.03	0.02	-0.02	0.1	0.02	0.03	0.11	0.04	0.13
Burn spicy	0.01	0	-0.04	-0.02	0.73	-0.01	0	0.03	-0.04	-0.05	-0.01	0.06	0	0.01	0.03	0.02	0.03
Butter on bread	0.05	0.01	0	0.1	-0.04	-0.01	-0.02	0.01	0.02	0.09	0.16	0.14	0.03	-0.06	-0.04	0.35	-0.05

Butternut squash	-0.05	0	0.06	0.06	0.04	0.14	0.05	-0.03	0.4	0.03	0.04	0.06	-0.08	0.19	-0.11	-0.05	-0.01
Cabbage	0.05	0.02	0.01	-0.02	0.01	0.73	0	-0.02	-0.04	0.03	-0.04	0.03	0.01	0	-0.02	0.04	-0.01
Cake	0	0.01	0.02	0.81	0.01	0.02	0	-0.01	0	-0.02	0	-0.02	-0.04	0	-0.02	0.02	-0.04
Cake icing	0.05	-0.02	-0.05	0.65	-0.01	0.02	-0.04	0.04	-0.02	0.04	0	0	0.02	-0.03	0.08	-0.04	-0.02
Capers	-0.02	0.1	-0.02	-0.04	0.08	0	0.31	0.04	0.17	0.14	0	0.18	-0.07	0	0.04	-0.05	-0.12
Cauliflower	0	0.01	0	0.02	0	0.73	-0.01	-0.01	0	0.01	0.06	-0.03	0	-0.05	0.02	0	0.04
Cereal bar	0.01	0.01	0.03	0.18	-0.02	-0.04	-0.01	0.03	0.05	0.02	0.04	0.01	0.01	0.35	0.16	-0.09	0.13
Cheesecake	0.03	0	-0.05	0.42	0.05	0.01	-0.04	0	0.02	0.03	0.21	-0.08	0	0.06	0.09	0.06	0.1
Cherries	0.01	0.01	0.57	0.08	-0.01	-0.03	0.1	-0.01	0.05	0.02	0.03	0.05	-0.01	-0.07	-0.04	-0.01	-0.07
Chicken	0.76	0.13	0.02	0	-0.02	-0.01	0.04	-0.07	-0.06	0.03	-0.04	-0.09	0.01	0.09	-0.1	-0.04	0.07
Chilli pepper	-0.01	0	-0.01	-0.03	0.79	0.01	0.02	0.03	0.02	0	-0.04	0.03	-0.01	0.02	0.02	0	0
Chips	0.06	0	0.02	0.17	-0.02	-0.02	0.02	0.09	-0.07	0.06	-0.05	0.25	-0.01	-0.03	0.05	0.13	0.07
Cod	0.08	0.73	0.01	-0.02	0	0.05	-0.01	0	-0.09	-0.02	-0.04	0	0.01	0.03	-0.02	0.07	0.08
Coffee with sugar	-0.01	0	0	0.02	0	0.01	0.02	0.05	0.01	0.02	0.02	0.01	0.77	0.04	0.01	0.02	-0.02
Coffee without sugar	0.02	0	-0.01	0.03	0.03	0.01	0.04	0.14	0.01	0	0.05	-0.01	-0.54	0.06	0.08	0.04	0.03
Coriander	-0.02	0.02	0.04	-0.02	0.34	0.06	0.06	0.01	0.26	0.05	-0.02	0.04	-0.04	0.08	-0.03	-0.02	-0.08
Cornflakes	0.07	-0.01	0.01	0.1	-0.05	0.04	-0.05	0.01	-0.11	0.1	-0.06	0.02	0.1	0.17	0.2	0.13	0.22
Cream	0.08	-0.02	-0.02	0.23	0.02	0.02	-0.05	0.02	0.1	0.1	0.21	0	0	-0.08	0.02	0.42	0.01
Croissant	0.03	0	-0.02	0.27	-0.03	-0.09	0.06	0.02	0.2	0.03	0.12	0.05	-0.04	0.02	0.19	0.07	-0.01
Cucumber	-0.01	0	0.22	-0.03	0	0.25	0.03	-0.03	0.04	0.18	0.02	0.03	-0.04	-0.03	0.01	-0.06	0.08
Curry	0	0	0.01	0.05	0.76	-0.01	0.02	0.01	-0.01	0.03	0.03	-0.07	0.01	0	-0.03	0	0.03
Dairy products	0.06	0.02	0.06	0.05	0.02	0.02	-0.05	-0.03	-0.04	0.02	0.45	0.01	0	0.04	-0.01	0.38	0.05
Dark chocolate	0	0.06	0.04	0.14	0	0	0.03	0.15	0.01	-0.04	0.07	0.03	-0.05	0.13	0.05	-0.02	-0.18
Diet fizzy drinks	0.07	-0.06	0	0.09	0.07	0.02	-0.07	0.03	-0.06	0.05	0.02	0.13	0.04	0.02	0.07	-0.11	0.39
Dried fruit	0	0.03	0.3	0.16	-0.02	-0.01	0.06	-0.01	0.01	0.02	0.06	0.01	-0.02	0.21	0.06	-0.03	-0.16
Eggs	0.1	0.14	0.08	-0.03	-0.01	0.1	-0.06	0.02	-0.03	0.11	0.15	-0.02	0.02	0.04	-0.09	0.2	0.02
EVO oil	-0.02	0.05	0.04	-0.08	0.1	0.02	0.17	0.04	0.24	0.17	0	0	0	0.09	-0.05	0.05	-0.17
Fatty foods	0.17	-0.03	-0.13	0.11	0.1	-0.01	-0.01	0	0.06	0.01	0	0.3	0	-0.07	0.02	0.25	-0.01
Tomatoes	-0.02	0.01	0.32	-0.08	0	0.17	0.06	0.01	-0.04	0.17	0.05	-0.04	-0.02	-0.06	0.01	-0.01	-0.02
Fried chicken	0.49	0.08	-0.04	0.04	0.02	-0.06	0.03	-0.02	-0.02	0.04	-0.07	0.09	0.07	0.04	0.09	0.13	0.1
Fried fish	0.12	0.39	-0.07	0.11	0.02	-0.03	0.01	0.04	-0.12	0.03	-0.05	0.13	0.01	-0.05	0.08	0.2	0.07

Fruit	-0.01	0.01	0.68	-0.01	0	0.02	0.01	0	-0.02	-0.05	0.01	-0.03	-0.02	0.04	0.03	-0.03	0.01
Garlic	0.01	0.01	0.02	-0.05	0.36	0.06	0.04	0.03	0.19	0.18	0.01	0	0.01	0	-0.09	0	-0.12
Gherkins	0.02	0	0.01	-0.05	0.09	0.09	0.37	0	-0.04	0.27	0	0.14	-0.03	-0.01	0.06	-0.06	-0.02
Globe artichoke	-0.05	0.01	0.01	-0.06	0	0.06	0.19	0.05	0.38	-0.01	-0.02	0.13	-0.05	0.03	0.06	0.01	-0.09
Goat cheese	-0.02	0.02	0	-0.05	0.01	-0.02	0.14	0.04	0.19	0.01	0.39	0.04	-0.04	0.11	-0.04	-0.01	-0.04
Grapefruit	-0.01	0.02	0.23	-0.01	0.06	0.05	-0.03	0.03	0.04	0	0.04	0.1	-0.06	0.01	0.29	0.01	-0.06
Green olives	-0.01	-0.01	0.01	-0.01	0.01	0.01	0.94	0	-0.02	0	0	-0.01	0	-0.01	-0.01	0	0.03
Haddock	0.03	0.79	0.01	0	0	0.05	-0.01	0.01	-0.07	-0.03	-0.01	0.02	-0.01	0	-0.01	0.03	0
Ham	0.75	0.01	0	0.04	-0.06	0.03	-0.03	0.01	-0.09	0.03	0.06	0.03	0.01	-0.02	0.05	-0.05	0
Hard cheese	0	0.02	0.04	-0.01	0.03	0.06	0	0.02	-0.14	0.01	0.61	0.07	-0.01	0	-0.01	0.03	-0.02
Herring	0	0.57	0.02	-0.04	0.02	0	0.05	0.03	0.02	0.02	-0.01	0.01	0	0.03	0.1	0.01	-0.13
Honey	-0.01	0.07	0.08	0.21	-0.03	-0.06	0.12	0.07	0.08	0.11	0.02	-0.11	0.11	0.21	0.05	0.13	-0.15
Horseradish/wasabi	0.03	0.02	-0.05	-0.04	0.22	0.07	0.22	0.08	0	0.23	0.02	0.06	-0.03	0	0.04	-0.02	-0.06
Ice cream	0.04	0.01	0.11	0.41	0.02	-0.02	-0.03	0.05	-0.04	-0.01	0.08	-0.02	0.02	-0.01	0.09	0.07	0.12
Jam	0.03	0.01	0.07	0.5	-0.05	-0.01	0	0.06	-0.05	0.12	-0.06	-0.03	0.05	0.08	0.1	0.08	-0.09
Kiwi	0	0.02	0.42	0.01	0.02	0.03	0.01	-0.02	0.17	0.05	0.02	-0.02	-0.03	0.1	0.02	-0.03	0.09
Lager	0.01	0	-0.05	-0.04	0.08	0.01	0.03	0.46	-0.04	0	-0.03	0.04	0	0.04	0.12	0.03	0.16
Lamb	0.74	0.06	0.03	0	-0.03	0.02	-0.01	0.02	0.07	-0.01	0	-0.05	0	-0.04	-0.02	0.07	-0.09
Lemons	-0.01	0.03	0.21	-0.03	0.13	0.04	0	0.02	0.14	0.14	0.01	0.16	-0.09	0.02	0.16	0	-0.18
Lentil beans	-0.1	0.04	0.1	-0.03	0.17	0.25	0.07	0	0.07	-0.02	0.01	0.05	-0.01	0.26	-0.05	-0.01	-0.11
Liver	0.44	0.15	0.02	-0.03	-0.02	0.13	-0.01	0.03	-0.04	0.02	0.05	-0.05	0.03	0.02	-0.02	0.04	-0.09
Mackerel	-0.03	0.71	0.01	-0.01	0.04	-0.01	0.03	0.01	0.02	0.02	0.02	-0.01	-0.01	0.02	0.03	-0.03	-0.1
Marzipan	0.01	0	0.03	0.47	-0.03	0.02	0.04	0.05	-0.02	0.03	0.07	0.02	-0.04	0.05	0.01	-0.05	-0.16
Mayonnaise	0.01	0.01	-0.09	0.06	-0.05	-0.01	0.01	0.02	0.09	0.5	0.16	-0.01	-0.02	-0.05	0.01	0.08	0.02
Melon	0.03	0	0.45	0.02	-0.03	0.05	0.01	0	0.11	0.06	0.02	-0.02	0.01	0	0.01	-0.04	0.11
Milk chocolate	0.01	0	0.02	0.43	0.05	0.02	-0.03	-0.02	-0.02	0.02	0.05	0.02	0.03	-0.03	0	0.08	0.25
Mushroom	0.01	0.06	0.1	-0.04	0.06	0.21	0.02	0.04	0.12	0.1	0.1	-0.08	0.02	-0.04	-0.01	-0.01	0.02
Onion	0.03	0	0.09	-0.06	0.3	0.24	-0.04	0.01	-0.03	0.2	-0.01	0.02	0.02	-0.03	-0.04	0.05	-0.12
Orange juice	0.03	0.01	0.11	0.04	0.02	0.01	-0.03	0.01	-0.02	0.06	0	-0.07	0.08	-0.02	0.59	0.05	0.04
Oranges	0.02	0.03	0.51	-0.06	0.07	0.03	-0.02	0	-0.04	-0.01	-0.01	0	-0.01	-0.01	0.3	0.03	0.01
Pasta	0.03	0	0	0.03	0.09	0.03	0.01	0.01	0.12	0.12	0.13	-0.08	0.01	0.16	0.08	-0.12	-0.04

Pears	0.02	0.03	0.56	0.04	-0.03	0.04	0.02	0.01	-0.02	-0.03	-0.03	0	0.02	0.08	-0.03	0.05	-0.01
Pizza	0.01	-0.05	-0.06	0.15	0.07	-0.04	0.04	0.04	0.04	0.06	0.18	0.07	0	0.06	0.18	-0.06	0.09
Plain yogurt	-0.04	0.02	0.11	-0.08	0.04	0.01	0.06	0	0.12	0.05	0.24	-0.03	-0.12	0.28	-0.04	0.13	0.01
Plums	0.02	0.01	0.69	0.03	0	0.03	0.03	-0.01	-0.01	-0.01	0.02	0.02	-0.02	-0.02	-0.03	0.01	-0.05
Pollock	0.01	0.45	-0.01	-0.05	0.04	0.05	-0.04	0.02	0.13	-0.01	-0.02	0.08	-0.02	0.1	-0.01	0	0.03
Pork chops	0.74	0.01	0.03	0.02	-0.03	0.07	-0.05	0.04	-0.02	-0.01	0.02	-0.01	0	-0.02	0.04	0.02	-0.03
Porridge	-0.02	0.03	0.1	0.03	-0.01	0.06	0.01	0.02	-0.02	0.07	0	-0.06	-0.02	0.37	-0.12	0.17	0.01
Crisps	0.02	-0.01	0.05	0.13	-0.04	0	0.01	0.06	-0.05	0.07	0.02	0.38	-0.02	-0.01	0.01	-0.02	0.16
Potatoes	0.03	0	0.09	0.03	-0.02	0.13	-0.02	0.05	-0.1	0.07	-0.05	0.1	0.04	0.06	0.04	0.15	-0.06
Prawns	0.11	0.46	0.09	-0.04	0.03	-0.06	0.02	0.07	0.19	0.15	0.09	-0.07	0.02	-0.2	-0.04	-0.04	0.14
Raw carrots	0	-0.01	0.3	-0.06	-0.01	0.19	0	-0.01	0.05	0.07	0.02	0.1	-0.05	0.14	0	-0.08	0.03
Red meat	0.86	-0.05	0.02	-0.01	0.01	0.01	-0.03	0.06	0.02	-0.03	-0.01	0.01	0	-0.05	0	0.07	-0.03
Red wine	0.04	0.03	-0.02	-0.01	0.03	-0.02	0.1	0.6	0.08	-0.04	0.05	-0.05	-0.05	-0.02	0.05	-0.02	-0.12
Fizzy drinks	0.05	-0.03	-0.03	0.14	0	0	-0.02	0.06	0	0.04	-0.05	0.11	0.16	-0.01	0.23	0.06	0.21
Roast chicken	0.77	0.13	0.02	0.02	-0.03	-0.01	0.05	-0.07	-0.05	0.03	-0.03	-0.05	0.01	0.07	-0.1	-0.01	0.05
Salad dressing	-0.02	-0.01	-0.02	0.02	-0.01	0.03	0.05	0.03	-0.02	0.58	0.07	0	0.01	-0.03	0.05	0	0
Salad leaves	-0.03	0.02	0.21	-0.08	0	0.29	0.01	-0.01	0.08	0.15	0.04	0.04	-0.04	0.07	0	-0.09	-0.02
Salami	0.47	0.02	-0.11	-0.01	0.13	-0.07	0.17	0.02	0.11	0.06	0.11	0.08	0.01	0	0.11	-0.09	-0.07
Salmon	0.02	0.72	0.01	0.06	-0.05	0	0.01	0.01	0.08	0	0.03	-0.06	-0.03	-0.03	-0.02	-0.02	0.04
Salty foods	0.01	0.03	-0.01	0.02	0.04	-0.04	0.01	0.01	0.01	0	0.06	0.7	0.06	-0.02	-0.04	0.03	-0.01
Salty pretzels	0.02	-0.01	-0.02	0.07	0.01	-0.05	0.08	0.05	0.12	0.01	0.09	0.45	-0.03	0.13	0.06	-0.14	0.13
Sardines	0	0.63	0.05	-0.02	0.01	-0.01	0.05	0.01	-0.02	0.06	0.03	-0.04	0.01	0.01	0.03	0	-0.11
Sausages	0.69	0	-0.06	0.06	0.01	-0.01	-0.01	0.02	-0.05	0	0.03	0.05	0	-0.01	0.08	0	-0.04
Savoury biscuits	0.01	0.03	0.06	0.17	-0.04	0.04	-0.01	0.03	-0.05	0.06	0.25	0.25	-0.01	0.08	0.04	-0.11	0.01
Shellfish	0.08	0.41	0.08	-0.09	0.07	-0.05	0.05	0.1	0.23	0.09	0.05	-0.01	0.01	-0.15	-0.02	-0.03	0.08
Skimmed milk	0.03	0.04	0.03	-0.03	0.02	0.05	-0.02	0.02	-0.07	0.02	0.09	-0.05	-0.04	0.2	-0.01	-0.04	0.2
Smoked fish	0.01	0.68	-0.01	0.02	0.05	-0.01	0.04	0.02	-0.01	0	0.07	0.08	0	-0.03	0.05	-0.06	-0.07
Soft cheese	0	0.01	0.01	0	0	0.01	0.05	0.01	0.07	0.09	0.61	0.03	0.01	0.02	0.01	0.05	0.04
Soy sauce	0.02	0.03	0	0.01	0.25	-0.01	0	0.04	0.14	0.25	0.03	0.15	0.02	0.06	0	-0.09	-0.01
Soy milk	-0.18	-0.01	0	-0.01	0.01	0.01	0.03	0.02	0.18	0.06	-0.1	0.06	0.02	0.3	-0.03	0.02	0.07
Spicy foods	-0.01	0	0.01	0.03	0.92	-0.02	0.01	0	-0.04	-0.03	0.02	-0.02	0	-0.03	-0.01	0	0.01

Spinach	-0.04	0.07	-0.01	-0.05	0.05	0.34	0.12	0	0.33	-0.01	0.02	0	-0.03	0.11	0	0	-0.06
Spirits	0.02	-0.02	0.04	0.03	0.03	0.01	-0.05	0.74	0	0.08	0.01	0.05	0.02	-0.02	-0.13	-0.04	0.1
Strawberries	0.02	0	0.49	0.08	0.02	0	-0.02	0.02	0.06	0.03	0.03	-0.04	-0.02	-0.07	0.03	0.03	0.06
Sweet coffee drinks	-0.01	-0.01	-0.04	0.11	0.01	0.01	-0.02	0.03	0.03	0.08	0.01	-0.03	0.3	0.13	0.07	0.08	0.21
Sweet foods	-0.02	-0.01	0.02	0.76	0.05	-0.01	-0.02	-0.06	0.06	0	-0.05	0.07	0.08	-0.01	-0.05	0.02	0.06
Tea with sugar	-0.01	0	0	-0.02	0.01	0.01	0.02	0.03	0.01	-0.03	0.02	0.03	0.76	0.02	0.03	0	-0.02
Tea without sugar	0	0.04	0.01	0.03	-0.03	0.01	0.03	0.07	-0.03	0.04	-0.04	-0.01	-0.48	0.13	0.01	0.14	0.01
Ketchup	0.01	-0.02	-0.04	0.09	-0.02	0	0.01	0.06	-0.08	0.39	-0.07	0.01	0.05	0.08	0.06	0.05	0.09
Tinned Tuna	0.07	0.46	-0.02	-0.01	0.01	0.02	-0.01	-0.03	-0.06	0.17	0.04	-0.02	-0.02	0.1	-0.02	-0.06	0.12
Turnip	0.04	0.01	0.04	-0.01	0.04	0.41	-0.01	0.02	0.02	0.06	-0.06	0.03	-0.01	0.14	0	0.03	0.02
Vegetables	-0.03	0.02	0.14	-0.02	-0.01	0.6	0.02	0	0.04	0	0.01	-0.02	-0.01	0.03	-0.05	-0.04	-0.03
Vinegar	0	-0.01	0.05	-0.02	0.12	0.05	0	0.03	-0.18	0.38	-0.06	0.21	-0.02	0.05	0.05	0.04	-0.03
Whisky	0.01	0	0	0	0.01	-0.01	0.02	0.66	-0.07	-0.01	-0.03	-0.01	0.04	0.05	-0.03	0.04	-0.04
White bread	0.08	-0.03	-0.02	0.11	0	0.03	-0.01	0	-0.03	0.06	-0.07	0.16	0.06	-0.11	0.15	0.2	0.17
White rice	0.09	0.02	0.04	0.07	0.12	0.05	0	-0.02	0.04	0.09	-0.01	0.02	0.05	0.01	0.14	0.05	0.04
White wine	0.01	0.05	0.03	0.04	-0.04	-0.01	0	0.5	0.13	0.01	0.09	0.02	-0.06	-0.09	0.08	-0.06	-0.03
Whole grain cereals	0	0.04	0.1	0.04	-0.02	0.01	0	0.03	-0.02	-0.03	0.08	-0.06	-0.04	0.48	0.09	-0.01	0.02
Whole milk	0.05	0.01	-0.02	0.01	0	-0.03	0	0.02	0.07	-0.01	0.1	0.04	0.11	0.07	0.12	0.49	-0.06
Wholemeal bread	0	0.05	0.11	0.02	0.02	0.09	-0.03	0.03	-0.03	-0.02	0.14	-0.03	-0.05	0.3	0.03	-0.07	-0.09

Supplementary Table 3: Dimension of FPQ items based on Exploratory Factor Analysis

Factor	SS loadings	Percentage of variance	Items	Dimension
**MR2**	7.89	0.06	Bacon, BBQ grilled meat, Beef steak, Bolognese sauce, Burgers, Chicken, Fried chicken, Ham, Lamb, Liver, Pork chop, Red meat, Roast chicken, Salami, Sausages	Meat and Processed meat
**MR5**	5.64	0.04	Baked/steam fish, Cod, Fried fish, Haddock, Herring, Mackerel, Pollock, Prawns, Salmon, Sardines, Shellfish, Smoked fish, Tinned tuna	Fish/Seafood
**MR7**	4.43	0.03	Apple juice, Apples, Bananas, Cherries, Dried fruit, Tomatoes, Grapefruit, Fruits, Kiwi, Lemons, Melon, Orange juice, Oranges, Pears, Plums, Raw carrots, Strawberries	Fruits
**MR3**	4.41	0.03	Biscuits, Cake, Cake icing, Cheesecake, Croissant, Dark Chocolate, Ice cream, Jam, Marzipan, Milk chocolate, Sweet foods	Dessert
**MR4**	4.29	0.03	Aniseed, Bell pepper, Bitter foods, Black pepper, Burn spicy, Chilli pepper, Coriander, Curry, Garlic, Horseradish/wasabi, Onions, Spicy foods	Strong flavours
**MR9**	4.2	0.03	Bitter ale, Lager, Red wine, Spirits, Whisky, White wine	Alcohol
**MR16**	3.43	0.02	Black olives, Capers, Gherkins, Green olives	Sharp flavours
**MR12**	2.79	0.02	Chips, Pasta, Pizza, Potatoes, white rice	Starchy foods
**MR1**	2.77	0.02	Asparagus, Aubergine, Avocados, Butternut squash, Globe artichoke	Strong vegetables
**MR14**	2.58	0.02	EVO oil, Mayonnaise, Salad dressing, Soy sauce, Tomato ketchup, Vinegar	Sauces
**MR11**	2.48	0.02	Blue cheese, Dairy products, Goat cheese, Hard cheese, Soft cheese	Dairy
**MR10**	2.39	0.02	Add salt to food, Crips, Fatty foods, Salty foods, Salty pretzel, Savoury biscuits	Salty/Savoury
**MR8**	2.29	0.02	Coffee with sugar, Coffee without sugar, Sweet coffee drinks, Tea with sugar, Tea without sugar	Beverages
**MR15**	2.18	0.02	Butter on bread, Cream, Eggs, White bread, Whole milk	Breakfast
**MR13**	1.81	0.01	Brown rice, Cereal bar, Honey, Plain yogurt, Porridge, Skimmed milk, Soya milk, Whole grain cereals, Wholemeal bread	Prudent breakfast
**MR17**	1.67	0.01	Beetroot, Broad beans, Broccoli, Brussel sprouts, Cabbage, Cauliflower, Cucumber, Lentil beans, Mushrooms, Salad leaves, Spinach, Turnip, Vegetables	Vegetables
**MR18**	1.55	0.01	Corn flakes, Diet fizzy drinks, Fizzy drinks	Processed foods

Note: Factors are ordered based on SS loadings

Supplementary Table 4: Actual daily nutrient intake for each food preference groups

Nutrient	Reference value (age 19 – 64)	X1 (Health-conscious)	X2 (Omnivore)	X3 (Sweet-tooth)	p-value
**Energy (kJ)**	Males: 10,500 kJ/day<br>Females: 8,400 kJ/day	8,330 ± 2,182	8,910 ± 2,348	8,712 ± 2,456	<0.001
**Protein (g)**	Males: 55,5 g/day<br>Females: 45 g/day	78 ± 22	84 ± 23	80 ± 24	<0.001
**Carbohydrate (g)**	Males: [at least] 333 g/day<br>Females: [at least] 267 g/day	245 ± 71	258 ± 74	258 ± 79	<0.001
**Fat (g)**	Males: [less than] 97 g/day<br>Females: [less than] 78 g/day	70 ± 26	76 ± 27	74 ± 28	<0.001
**Saturated fatty acids (g)**	Males: [less than] 31 g/day<br>Females: [less than] 24 g/day	25 ± 11	29 ± 12	28 ± 12	<0.001
**Total sugar (g)**	90 g	123 ± 44	126 ± 45	125 ± 49	<0.001
**Free sugar (g)**	Males: [less than] 33 g/day<br>Females: [less than] 27 g/day	51 ± 28	63 ± 34	65 ± 37	<0.001
**Dietary fibre (g)**	30 g/day	19 ± 7	18 ± 6	17 ± 6	<0.001

Note: This table shows the number of people in each food preference group (Health-conscious, Omnivore, or Sweet-tooth) in a total sample of 124,062 participants with complete data; Data are presented as mean ± SD; The p-value indicates the probability of observing a difference in nutrient intake across groups by chance. A low p-value (<0.05) suggests that the difference is statistically significant. The p-value is calculated by Kruskal-Wallis rank sum test; Reference value based on Government Dietary Recommendations 2016.

Supplementary Table 5: Blood biochemistry and blood count for each food preference groups

**Characteristic**	**N**	**Health-conscious**, N = 58,625[1]	**Omnivore**, N = 69,504[1]	**Sweet-tooth**, N = 53,608[1]	**p-value**[2]	**Reference range**
alanine_aminotransferase_f30620_0_0	170,883	21 (12)	23 (13)	24 (14)	<0.001	< 35 U/L
albumin_f30600_0_0	156,275	45.44 (2.56)	45.35 (2.55)	45.30 (2.61)	<0.001	35–54 g/L
alkaline_phosphatase_f30610_0_0	170,929	79 (24)	82 (24)	83 (24)	<0.001	36–150 U/L
apolipoprotein_a_f30630_0_0	155,293	1.62 (0.28)	1.54 (0.26)	1.52 (0.26)	<0.001	0.98–2.1 g/L (females); 0.88–1.8 g/L (males)
apolipoprotein_b_f30640_0_0	170,166	1.02 (0.23)	1.04 (0.23)	1.03 (0.24)	<0.001	0.44–1.48 g/L (females); 0.55–1.51 g/L (males)
aspartate_aminotransferase_f30650_0_0	170,313	25 (9)	26 (9)	26 (10)	<0.001	< 35 U/L
creactive_protein_f30710_0_0	170,591	1.94 (3.73)	2.32 (3.89)	2.47 (4.08)	<0.001	< 8.0 mg/L
calcium_f30680_0_0	156,238	2.38 (0.09)	2.38 (0.09)	2.38 (0.09)	0.001	2.2–2.6 mmol/L
cholesterol_f30690_0_0	170,929	5.80 (1.09)	5.74 (1.11)	5.68 (1.12)	<0.001	3.88–5.15 mmol/L
creatinine_f30700_0_0	170,835	68 (14)	73 (15)	74 (16)	<0.001	Men: 60–110 mcmol/L;Women: 45–90 mcmol/L
cystatin_c_f30720_0_0	170,925	0.86 (0.13)	0.89 (0.14)	0.90 (0.15)	<0.001	
direct_bilirubin_f30660_0_0	146,321	1.80 (0.82)	1.84 (0.82)	1.85 (0.81)	<0.001	0–5.1 mcmol/L
gamma_glutamyltransferase_f30730_0_0	170,851	30 (31)	35 (34)	37 (38)	<0.001	Adult male: 8–61 U/L; Adult female: 5–36 U/L
glucose_f30740_0_0	156,107	5.03 (0.99)	5.04 (1.02)	5.08 (1.13)	<0.001	< 7.8 mmol/L (2 h pp)
glycated_haemoglobin_hba1c_f30750_0_0	169,871	35.0 (5.1)	35.3 (5.4)	35.6 (6.0)	<0.001	<42 mmol/mol
hdl_cholesterol_f30760_0_0	156,226	1.59 (0.40)	1.46 (0.37)	1.42 (0.37)	<0.001	≥ 1.04 mmol/L
igf1_f30770_0_0	170,025	21.8 (5.6)	21.9 (5.5)	21.6 (5.7)	<0.001	
ldl_direct_f30780_0_0	170,591	3.58 (0.83)	3.60 (0.85)	3.57 (0.85)	<0.001	≤ 3.36 mmol/L
lipoprotein_a_f30790_0_0	137,100	44 (49)	44 (49)	44 (49)	0.3	
oestradiol_f30800_0_0	30,480	514 (450)	466 (448)	463 (441)	<0.001	30 - 500 pg/mL (females); <56 pg/mL (males)
phosphate_f30810_0_0	155,959	1.18 (0.16)	1.16 (0.16)	1.15 (0.16)	<0.001	0.8 – 1.5 mmol/L
rheumatoid_factor_f30820_0_0	14,667	24 (20)	24 (19)	24 (20)	0.9	<20 IU/mL
shbg_f30830_0_0	154,713	59 (30)	51 (27)	50 (27)	<0.001	
testosterone_f30850_0_0	154,616	4.8 (5.7)	6.9 (6.1)	7.2 (6.1)	<0.001	0.7–2.6 nmol/L (females); 10–42 nmol/L (males)
total_bilirubin_f30840_0_0	170,218	9.1 (4.4)	9.3 (4.6)	9.3 (4.5)	<0.001	5.1–20.5 mcmol/L
total_protein_f30860_0_0	156,120	72.1 (4.0)	72.4 (4.0)	72.4 (4.0)	<0.001	60 – 80 g/L
triglycerides_f30870_0_0	170,788	1.50 (0.87)	1.71 (0.98)	1.78 (1.05)	<0.001	< 2.30 mmol/L (non-fasting)

urate_f30880_0_0	170,742	286 (75)	309 (78)	313 (80)	<0.001	150–470 mcmol/L
urea_f30670_0_0	170,811	5.18 (1.25)	5.44 (1.26)	5.34 (1.33)	<0.001	2.5 – 7.8 mmol/L
vitamin_d_f30890_0_0	163,688	50 (21)	50 (21)	48 (21)	<0.001	25–65 pg/mL

[1] Mean (SD)

[2] Kruskal-Wallis rank sum test

Note: This table shows the number of people in each food preference group (Health-conscious, Omnivore, or Sweet-tooth) in a total sample of 181,738 participants; Data are presented as mean ± SD; The p-value indicates the probability of observing a difference in parameter value across groups by chance. A low p-value (<0.05) suggests that the difference is statistically significant. The p-value is calculated by Kruskal-Wallis rank sum test; Reference ranges are for non-fasting adults, taken from MSD manual, USA

Supplementary Table 6: pairwise group tests for metabolomics differential expression for Volcono3D plot

	X1	X2	X3	x	y	r	angle	z	pvalue	col	lab
Clinical_LDL_C	0.039174	0.006158	-0.0547	0.063446	0.052707	0.082483	39.71773	12.16539	6.8330395	purple	H+O+
VLDL_size	-0.19421	0.074871	0.118519	-0.2909	-0.0378	0.29335	187.4035	168.5763	2.6527994	gold2	O+S+
LDL_size	0.011524	-0.01294	0.005278	0.015356	-0.01578	0.022017	314.2234	1.013319	0.09698	grey60	ns
HDL_size	0.284062	-0.10624	-0.17809	0.426227	0.06223	0.430746	8.306637	264.871	1.3457311	purple	H+O+
Phosphoglyc	0.147748	-0.03389	-0.12359	0.226486	0.077686	0.239439	18.93218	105.6772	2.1026465	purple	H+O+
Cholines	0.166539	-0.04276	-0.1327	0.254267	0.077891	0.26593	17.03169	129.7583	1.7446125	purple	H+O+
Phosphatidylc	0.173119	-0.04358	-0.1392	0.264509	0.082811	0.277169	17.38404	140.4789	3.3197347	purple	H+O+
Sphingomyelins	0.171414	-0.04328	-0.13764	0.261874	0.081715	0.274327	17.33002	137.5047	3.1284315	purple	H+O+
ApoB	-0.00574	0.019406	-0.02141	-0.00474	0.035344	0.03566	97.63598	2.570218	0.00269	red	O+
ApoA1	0.224385	-0.06911	-0.16213	0.340005	0.080552	0.349416	13.32852	223.3712	4.2543789	purple	H+O+
Unsaturation	0.246878	-0.05827	-0.20412	0.378072	0.126305	0.398612	18.47319	286.6787	2.0957083	purple	H+O+
Omega_3	0.142539	2.3813607	-0.16662	0.225837	0.144319	0.268012	32.58018	11.91578	1.2140056	purple	H+O+
Omega_6	0.081445	-0.0226	-0.06244	0.123968	0.034499	0.128679	15.55133	30.55569	2.7816890	purple	H+O+
MUFA	-0.11289	0.042311	0.07064	-0.16936	-0.02453	0.17113	188.2425	59.06138	8.6820688	gold2	O+S+
SFA	-0.05258	0.036137	0.009098	-0.07519	0.023416	0.078754	162.7029	13.72797	1.8708155	gold2	O+S+
LA	0.087945	-0.03371	-0.05395	0.131776	0.017529	0.132937	7.577177	33.37616	4.2057558	blue	H+
DHA	0.22388	-0.03134	-0.21625	0.347676	0.160137	0.382783	24.73048	257.589	2.5764429	purple	H+O+
Ala	-0.05336	0.010848	0.046645	-0.0821	-0.031	0.087762	200.6854	13.98006	1.0469881	gold2	O+S+
Gln	0.028002	-0.01681	-0.00838	0.040595	-0.00731	0.041248	349.7983	3.504041	0.000313	blue	H+
Gly	0.150324	-0.10989	-0.01651	0.21352	-0.08087	0.228321	339.256	107.3751	4.2160852	cyan	H+S+
His	-0.00171	0.013572	-0.01766	0.000338	0.027051	0.027053	89.28368	1.452029	0.035316	red	O+
Ile	-0.10679	0.065608	0.029769	-0.15448	0.031038	0.157567	168.6395	51.40983	3.8919371	gold2	O+S+
Leu	-0.13949	0.0917	0.030192	-0.20044	0.053267	0.207396	165.1175	60.27748	5.2785896	gold2	O+S+
Val	-0.12134	0.086259	0.016853	-0.17289	0.060107	0.183042	160.8297	71.23334	5.8433216	gold2	O+S+
Phe	-0.0393	0.017704	0.020281	-0.05829	-0.00223	0.058332	182.192	1.39494	0.040277	grey60	ns
Tyr	-0.02317	0.029435	-0.01556	-0.0301	0.03897	0.049243	127.685	5.191246	6.4380517	red	O+
Glucose	-0.0024	-0.00559	0.010903	-0.00506	-0.01428	0.015152	250.4991	0.400612	0.397547	grey60	ns
Lactate	-0.06546	0.0168	0.052173	-0.09995	-0.03063	0.10454	197.0396	20.83727	1.4545525	gold2	O+S+

Pyruvate	0.010544	-0.00985	0.001949	0.014496	-0.01022	0.017736	324.8132	0.650841	0.223439	grey60	ns
Citrate	0.05614	-0.04409	-0.00175	0.079057	-0.03667	0.087146	335.1187	16.24356	5.7073830	cyan	H+S+
bOHbutyrate	0.029029	-0.01875	-0.00676	0.041786	-0.01039	0.043058	346.0417	3.651046	0.000223	blue	H+
Acetate	-0.00109	0.012283	-0.01652	0.001022	0.02494	0.024961	87.65322	1.110527	0.07753	grey60	ns
Acetoacetate	0.020448	-0.00397	-0.01815	0.031507	0.012286	0.033817	21.3027	1.772276	0.016894	blue	H+
Acetone	0.029559	-0.00752	-0.02365	0.045145	0.013967	0.047257	17.19101	2.092289	0.008086	blue	H+
Creatinine	-0.1915	0.084556	0.101327	-0.28445	-0.01452	0.284817	182.923	168.0486	8.9415817	gold2	O+S+
Albumin	0.066434	9.2934646	-0.07778	0.105276	0.067437	0.125023	32.64266	28.06168	8.6759919	purple	H+O+
GlycA	-0.14228	0.041842	0.105678	-0.21604	-0.05528	0.223005	194.3534	94.15887	6.9364128	gold2	O+S+
XXL_VLDL_P	-0.18148	0.068283	0.113188	-0.27222	-0.03889	0.274983	188.1302	2.8929823	1	grey60	ns
XXL_VLDL_PL	-0.18385	0.067716	0.116774	-0.27609	-0.04249	0.279344	188.7481	111.2784	5.2677937	gold2	O+S+
XXL_VLDL_CE	-0.18861	0.071068	0.117479	-0.28288	-0.04019	0.285722	188.0867	40.88199	1.3122362	gold2	O+S+
XXL_VLDL_FC	-0.17913	0.066531	0.112977	-0.26889	-0.04022	0.271878	188.5079	14.93809	1.1532030	gold2	O+S+
XXL_VLDL_TG	-0.18084	0.068768	0.111739	-0.2711	-0.03721	0.273641	187.8162	159.3186	4.8014964	gold2	O+S+
XL_VLDL_P	-0.18948	0.076993	0.109917	-0.28293	-0.02851	0.284368	185.7547	6.2681285	1	grey60	ns
XL_VLDL_PL	-0.18503	0.074872	0.107792	-0.27637	-0.02851	0.277832	185.8897	9.012747	9.7107651	gold2	O+S+
XL_VLDL_CE	-0.15917	0.070037	0.084567	-0.23647	-0.01258	0.236805	183.0461	11.46638	3.4168270	gold2	O+S+
XL_VLDL_FC	-0.17768	0.072919	0.102025	-0.26515	-0.02521	0.266345	185.4305	0.457352	0.348858	grey60	ns
XL_VLDL_TG	-0.19083	0.078198	0.109744	-0.2848	-0.02732	0.286104	185.4793	16.72429	1.8867292	gold2	O+S+
L_VLDL_P	-0.17792	0.075143	0.099085	-0.26503	-0.02073	0.265842	184.4733	1.1523713	1	grey60	ns
L_VLDL_PL	-0.18551	0.076992	0.105279	-0.27665	-0.0245	0.27773	185.0604	5.82141	1.5086546	gold2	O+S+
L_VLDL_CE	-0.13381	0.063126	0.064939	-0.19784	-0.00157	0.197846	180.4546	6.24733	5.6580892	gold2	O+S+
L_VLDL_FC	-0.18105	0.076917	0.100173	-0.26959	-0.02014	0.270345	184.2724	1.061684	0.086759	grey60	ns
L_VLDL_TG	-0.17703	0.077388	0.094789	-0.26311	-0.01507	0.263545	183.278	46.38416	4.1289506	gold2	O+S+
M_VLDL_P	-0.07678	0.045299	0.024112	-0.11148	0.018349	0.112984	170.6536	9.0164618	1	grey60	ns
M_VLDL_PL	-0.06459	0.040623	0.016641	-0.09322	0.020769	0.095508	167.4399	0.18217	0.6574	grey60	ns
M_VLDL_CE	0.052831	-0.00356	-0.05659	0.082904	0.045923	0.094774	28.98308	0.023726	0.946835	grey60	ns
M_VLDL_FC	-0.03862	0.032328	-0.00169	-0.05394	0.029463	0.061462	151.3557	0	1	grey60	ns
M_VLDL_TG	-0.1538	0.070153	0.078126	-0.22794	-0.0069	0.228045	181.7351	7.486948	3.2587553	gold2	O+S+
S_VLDL_P	-0.11068	0.052746	0.052942	-0.16352	-0.00017	0.163519	180.0595	1.7598976	1	grey60	ns
S_VLDL_PL	-0.06654	0.040155	0.019595	-0.09641	0.017806	0.098043	169.5361	12.24967	5.6277027	gold2	O+S+

S_VLDL_CE	-0.08341	0.044183	0.033476	-0.12224	0.009273	0.122587	175.6619	1.979401	0.010486	gold2	O+S+
S_VLDL_FC	-0.03194	0.028601	-0.0041	-0.04419	0.028319	0.052489	147.3487	0.022816	0.94882	grey60	ns
S_VLDL_TG	-0.13804	0.060071	0.074306	-0.20522	-0.01233	0.205594	183.4377	84.7977	1.5933267	gold2	O+S+
XS_VLDL_P	0.018332	0.00885	-0.03424	0.031029	0.037321	0.048535	50.25964	5.2106402	1	grey60	ns
XS_VLDL_PL	0.012231	0.00746	-0.0251	0.021051	0.028198	0.035189	53.25711	2.232275	0.005858	purple	H+O+
XS_VLDL_CE	0.095767	-0.01542	-0.08958	0.14827	0.064225	0.161583	23.42029	0.251239	0.56074	grey60	ns
XS_VLDL_FC	0.034389	0.003647	-0.04547	0.055302	0.042539	0.06977	37.56829	8.583169	2.6111465	purple	H+O+
XS_VLDL_TG	-0.08368	0.035305	0.046661	-0.12467	-0.00983	0.125055	184.5105	30.92916	1.1771762	gold2	O+S+
IDL_P	0.040166	0.004791	-0.05388	0.064712	0.050814	0.082278	38.14007	1.1369420	1	grey60	ns
IDL_PL	0.113756	-0.02309	-0.0995	0.175051	0.066169	0.187139	20.70663	64.20008	6.3084200	purple	H+O+
IDL_CE	0.121077	-0.02052	-0.11179	0.187228	0.079043	0.203229	22.88827	75.92295	1.1941133	purple	H+O+
IDL_FC	0.116534	-0.02067	-0.10626	0.179996	0.074127	0.194662	22.383	69.47011	3.3875837	purple	H+O+
IDL_TG	-0.04189	0.017568	0.023503	-0.06242	-0.00514	0.062633	184.7072	7.769908	1.6986034	gold2	O+S+
L_LDL_P	0.019577	0.006561	-0.03238	0.032488	0.033727	0.046829	46.07228	4.1489095	0.99999	grey60	ns
L_LDL_PL	0.043703	0.00399	-0.05686	0.070136	0.052695	0.087726	36.91827	13.81361	1.5359938	purple	H+O+
L_LDL_CE	0.0579	-0.00162	-0.06533	0.091372	0.055176	0.106739	31.1264	20.52587	2.9794020	purple	H+O+
L_LDL_FC	0.095515	-0.01568	-0.08891	0.147812	0.063421	0.160844	23.22253	47.56193	2.7420294	purple	H+O+
L_LDL_TG	-0.05444	0.021478	0.03251	-0.08143	-0.00955	0.08199	186.6918	13.34569	4.5114219	gold2	O+S+
M_LDL_P	-0.04543	0.037976	-0.00191	-0.06346	0.034546	0.072256	151.4379	6.1484939	0.999986	grey60	ns
M_LDL_PL	-0.0357	0.03099	-0.00317	-0.04961	0.029583	0.05776	149.191	7.289648	5.1327724	gold2	O+S+
M_LDL_CE	-0.05422	0.04078	0.004299	-0.07676	0.031593	0.083012	157.6297	14.83395	1.4657191	gold2	O+S+
M_LDL_FC	0.026733	0.009219	-0.0446	0.044423	0.046607	0.064386	46.3745	6.774085	1.6823446	purple	H+O+
M_LDL_TG	-0.09688	0.035879	0.061255	-0.14545	-0.02198	0.147102	188.5918	42.12485	7.5015429	gold2	O+S+
S_LDL_P	-0.03909	0.029435	0.003049	-0.05533	0.02285	0.059867	157.562	1.0591922	1	grey60	ns
S_LDL_PL	0.008409	0.011245	-0.02612	0.015845	0.032356	0.036028	63.90946	2.362595	0.004339	purple	H+O+
S_LDL_CE	-0.04445	0.032912	0.004273	-0.06304	0.024802	0.067746	158.524	9.930178	1.1744170	gold2	O+S+
S_LDL_FC	0.036224	0.00443	-0.04875	0.058385	0.046057	0.074364	38.26821	9.832317	1.4712367	purple	H+O+
S_LDL_TG	-0.13012	0.050101	0.079496	-0.19492	-0.02546	0.196573	187.441	0.951056	0.111929	grey60	ns
XL_HDL_P	0.271491	-0.10217	-0.1693	0.407224	0.058141	0.411354	8.125449	2.5642648	0.999999	grey60	ns
XL_HDL_PL	0.274037	-0.10511	-0.16801	0.410597	0.054475	0.414195	7.557455	306.3267	4.7129627	purple	H+O+
XL_HDL_CE	0.285034	-0.10731	-0.17767	0.427527	0.060931	0.431847	8.111137	Inf	0	purple	H+O+

XL_HDL_FC	0.243205	-0.09351	-0.14878	0.36435	0.047862	0.36748	7.483619	23.21686	6.0693700	purple	H+O+
XL_HDL_TG	0.028404	-0.00851	-0.02088	0.043095	0.010713	0.044406	13.95997	1.287689	0.05156	grey60	ns
L_HDL_P	0.289574	-0.10537	-0.1858	0.435156	0.069656	0.440696	9.094241	0.006448	0.985262	grey60	ns
L_HDL_PL	0.282786	-0.10142	-0.18358	0.425287	0.071158	0.431199	9.498666	Inf	0	purple	H+O+
L_HDL_CE	0.291285	-0.10743	-0.1848	0.437403	0.067003	0.442505	8.709136	Inf	0	purple	H+O+
L_HDL_FC	0.287891	-0.10477	-0.18469	0.432623	0.069215	0.438125	9.089678	234.4855	3.2693707	purple	H+O+
L_HDL_TG	0.094554	-0.02845	-0.06929	0.143426	0.035371	0.147723	13.85379	2.49386	0.003207	purple	H+O+
M_HDL_P	0.21337	-0.0644	-0.15608	0.32361	0.07939	0.333206	13.78396	0.000174	0.9996	grey60	ns
M_HDL_PL	0.175032	-0.04843	-0.1344	0.26645	0.074453	0.276656	15.61165	122.3614	4.3515994	purple	H+O+
M_HDL_CE	0.22512	-0.07087	-0.16044	0.340775	0.077569	0.349492	12.8234	210.9981	1.0042742	purple	H+O+
M_HDL_FC	0.233465	-0.07191	-0.16869	0.353764	0.083818	0.363558	13.32942	30.94825	1.1265381	purple	H+O+
M_HDL_TG	-0.08326	0.041981	0.036493	-0.1225	0.004753	0.122587	177.7781	4.655038	2.2128994	gold2	O+S+
S_HDL_P	-0.01828	0.030777	-0.02322	-0.02206	0.046764	0.051704	115.2509	7.7902880	0.999998	grey60	ns
S_HDL_PL	-0.00565	0.025803	-0.03077	-0.00316	0.048997	0.049099	93.69463	4.821581	1.5080616	red	O+
S_HDL_CE	-0.01336	0.027408	-0.02409	-0.01502	0.044597	0.047059	108.6133	2.764977	0.001718	red	O+
S_HDL_FC	0.071957	-0.00326	-0.07937	0.113274	0.065908	0.131053	30.19266	2.563214	0.002734	purple	H+O+
S_HDL_TG	-0.18125	0.073109	0.105923	-0.27077	-0.02842	0.272252	185.9914	1.627791	0.023562	gold2	O+S+
XXL_VLDL_C	-0.18503	0.069266	0.115906	-0.27761	-0.04039	0.280536	188.2783	3.623626	0.000238	gold2	O+S+
XL_VLDL_C	-0.17045	0.072096	0.094764	-0.25388	-0.01963	0.254634	184.4216	0.304239	0.496319	grey60	ns
L_VLDL_C	-0.15998	0.070976	0.084151	-0.23754	-0.01141	0.237813	182.7501	0.198809	0.632691	grey60	ns
M_VLDL_C	0.013303	0.012462	-0.0336	0.023872	0.03989	0.046488	59.10174	0.11212	0.772467	grey60	ns
S_VLDL_C	-0.06542	0.038882	0.020131	-0.09493	0.016239	0.096305	170.2924	0	1	grey60	ns
XS_VLDL_C	0.07855	-0.0099	-0.07746	0.122231	0.058512	0.135514	25.58044	0.287217	0.516158	grey60	ns
IDL_C	0.120123	-0.02027	-0.11102	0.18577	0.07859	0.20171	22.93082	75.39877	3.9923282	purple	H+O+
L_LDL_C	0.068664	-0.00549	-0.07229	0.107557	0.057854	0.122129	28.27556	27.0689	8.5329271	purple	H+O+
M_LDL_C	-0.03328	0.033024	-0.00895	-0.04531	0.036348	0.058092	141.2658	7.495484	3.1953345	red	O+
S_LDL_C	-0.02328	0.026321	-0.01092	-0.03098	0.032255	0.044721	133.8434	0.543732	0.285935	grey60	ns
XL_HDL_C	0.275972	-0.10377	-0.17222	0.413964	0.059282	0.418187	8.149713	5.681276	2.0831685	purple	H+O+
L_HDL_C	0.291026	-0.10702	-0.1851	0.437085	0.067616	0.442284	8.793869	Inf	0	purple	H+O+
M_HDL_C	0.227843	-0.07141	-0.16285	0.344969	0.079191	0.353942	12.92882	231.0615	8.6804377	purple	H+O+
S_HDL_C	0.008555	0.020576	-0.0398	0.018169	0.05229	0.055357	70.83974	1.669123	0.021423	purple	H+O+

XXL_VLDL_L	-0.18336	0.068902	0.11448	-0.27505	-0.03947	0.277865	188.1668	88.8123	1.5406263	gold2	O+S+
XL_VLDL_L	-0.18765	0.077213	0.107461	-0.27999	-0.0262	0.281211	185.345	158.989	1.0256544	gold2	O+S+
L_VLDL_L	-0.17685	0.076461	0.095929	-0.26305	-0.01686	0.263587	183.6672	5.23131	5.8706973	gold2	O+S+
M_VLDL_L	-0.09605	0.05204	0.036872	-0.14051	0.013136	0.14112	174.6591	40.27248	5.3397975	gold2	O+S+
S_VLDL_L	-0.10425	0.051867	0.046709	-0.15354	0.004467	0.153608	178.3335	46.50443	3.1301556	gold2	O+S+
XS_VLDL_L	0.027778	0.005031	-0.03975	0.045138	0.038783	0.059512	40.66958	6.288876	5.1419047	purple	H+O+
IDL_L	0.111262	-0.02032	-0.1006	0.171722	0.069528	0.185264	22.04245	63.06478	8.6142265	purple	H+O+
L_LDL_L	0.056806	-0.00262	-0.06259	0.089413	0.05194	0.103404	30.1521	19.33544	4.6191282	purple	H+O+
M_LDL_L	-0.03884	0.032448	-0.00161	-0.05426	0.029498	0.061757	151.468	8.32618	4.7186759	gold2	O+S+
S_LDL_L	-0.02472	0.025427	-0.00794	-0.03346	0.028899	0.044214	139.1855	4.365242	4.3127828	red	O+
XL_HDL_L	0.272862	-0.10376	-0.16859	0.409039	0.056148	0.412875	7.816011	296.5947	2.5426741	purple	H+O+
L_HDL_L	0.287309	-0.10422	-0.18481	0.431824	0.069787	0.437427	9.180226	Inf	0	purple	H+O+
M_HDL_L	0.196191	-0.05727	-0.14633	0.297992	0.077133	0.307813	14.51207	176.0682	8.5464418	purple	H+O+
S_HDL_L	-0.02079	0.029805	-0.01888	-0.02625	0.042162	0.049667	121.9082	5.444895	3.5900860	red	O+
VLDL_CE	-0.05069	0.033096	0.011299	-0.07289	0.018877	0.075291	165.4798	11.74117	1.8147889	gold2	O+S+
VLDL_FC	-0.10781	0.052108	0.050514	-0.15912	0.00138	0.159125	179.5032	50.53091	2.9450599	gold2	O+S+
VLDL_C	-0.07565	0.041628	0.028111	-0.11052	0.011706	0.111137	173.9538	24.87487	1.3339074	gold2	O+S+
VLDL_PL	-0.12381	0.055853	0.063794	-0.18364	-0.00688	0.183766	182.1446	66.75642	1.7521939	gold2	O+S+
VLDL_TG	-0.17218	0.072285	0.096518	-0.25658	-0.02099	0.257438	184.6758	134.3599	4.3664909	gold2	O+S+
VLDL_L	-0.14219	0.063301	0.07448	-0.21108	-0.00968	0.2113	182.6263	89.22271	5.9881381	gold2	O+S+
VLDL_P	-0.08333	0.043747	0.034022	-0.12222	0.008423	0.122508	176.0578	2.3797604	0.999999	grey60	ns
LDL_CE	0.017805	0.013581	-0.04048	0.031255	0.04682	0.056294	56.27492	5.717488	1.9165140	purple	H+O+
LDL_FC	0.071712	-0.00649	-0.0744	0.112161	0.058812	0.126645	27.67051	29.2577	5.5245592	purple	H+O+
LDL_C	0.032614	0.008677	-0.05069	0.053618	0.05141	0.074283	43.7958	9.827007	1.4893385	purple	H+O+
LDL_PL	0.015832	0.013093	-0.03747	0.02802	0.043789	0.051986	57.38508	4.90506	1.2443427	purple	H+O+
LDL_TG	-0.07646	0.029322	0.046888	-0.11457	-0.01521	0.115573	187.5639	26.70217	1.9853032	gold2	O+S+
LDL_L	0.021964	0.010577	-0.04099	0.037171	0.044661	0.058106	50.22906	6.055638	8.7975620	purple	H+O+
LDL_P	-0.00529	0.018283	-0.0203	-0.00429	0.033411	0.033685	97.31229	5.7129998	0.999868	grey60	ns
HDL_CE	0.270277	-0.09157	-0.18323	0.407677	0.079381	0.415333	11.01846	313.6946	2.0202688	purple	H+O+
HDL_FC	0.263029	-0.08649	-0.18211	0.397331	0.082808	0.405869	11.77252	264.2371	5.7935538	purple	H+O+
HDL_C	0.264552	-0.08801	-0.1817	0.399406	0.081144	0.407565	11.48399	298.2057	6.2270994	purple	H+O+

HDL_PL	0.229795	-0.07362	-0.16192	0.347564	0.076467	0.355877	12.40782	232.4282	3.7305320	purple	H+O+
HDL_TG	-0.07533	0.03618	0.035631	-0.11124	0.000475	0.111239	179.7551	23.27008	5.3692703	gold2	O+S+
HDL_L	0.237342	-0.07716	-0.16561	0.358728	0.076598	0.366815	12.05319	243.9805	1.0459533	purple	H+O+
HDL_P	0.181756	-0.04816	-0.14265	0.277164	0.081829	0.288991	16.44856	0.022365	0.949806	grey60	ns
Total_CE	0.132032	-0.02925	-0.11194	0.202625	0.071609	0.214907	19.46366	85.59526	2.5394556	purple	H+O+
Total_FC	0.081551	-0.00949	-0.08156	0.127076	0.062408	0.141574	26.15593	36.35963	4.3688679	purple	H+O+
Total_C	0.112222	-0.02028	-0.10178	0.173252	0.070587	0.187079	22.16724	64.03058	9.3199810	purple	H+O+
Total_PL	0.126298	-0.0306	-0.10328	0.193237	0.06294	0.203229	18.04114	76.55441	2.7899315	purple	H+O+
Total_TG	-0.15672	0.065419	0.0884	-0.23363	-0.0199	0.234479	184.8689	111.7693	1.7009846	gold2	O+S+
Total_L	0.044066	0.000392	-0.05207	0.069905	0.045433	0.083372	33.02111	12.5489	2.8255160	purple	H+O+
Total_P	0.163063	-0.04301	-0.12827	0.248702	0.073833	0.25943	16.53485	0.000928	0.997865	grey60	ns
PUFA	0.109337	-0.01907	-0.10016	0.168951	0.070228	0.182966	22.57127	60.42102	3.7929629	purple	H+O+
Total_FA	-0.0223	0.021791	-0.0055	-0.03045	0.023636	0.038543	142.1757	3.347306	0.000449	red	O+
Total_BCAA	-0.12845	0.086854	0.024301	-0.18402	0.054172	0.191832	163.5968	2.286898	0.005165	gold2	O+S+
non_HDL_C	0.021789	0.01251	-0.04359	0.037327	0.048581	0.061265	52.46281	6.769268	1.7011067	purple	H+O+
Remnant_C	0.01296	0.01598	-0.0383	0.024118	0.047004	0.052831	62.8376	5.156441	6.9752347	purple	H+O+

Supplementary Table 7: Limma differential expression for metabolomics data

Health-conscious vs Omnivore

	metabolite	logFC	AveExpr	t	P.Value	adj.P.Val	B	FC
1	XL_HDL_CE	0.201787	-9E-17	19.17065	1.4412065	2.4212269	174.9204	1.150122
2	L_HDL_CE	0.195874	-1.2E-16	18.9335	1.2870510	1.0811229	170.4539	1.145418
3	L_HDL_C	0.193205	-5.8E-17	18.73533	5.2686979	2.9504708	166.7632	1.143301
4	XL_HDL_C	0.194035	-1.2E-16	18.31125	1.3052999	5.4822596	158.9938	1.143958
5	L_HDL_P	0.188455	8.0693581	18.21102	8.0668586	2.7104645	157.1832	1.139543
6	XL_HDL_PL	0.189407	-1.5E-16	17.96349	6.9526651	1.8359416	152.7533	1.140295
7	L_HDL_FC	0.18536	1.7729533	17.95814	7.6497569	1.8359416	152.6583	1.137101
8	HDL_size	0.185031	-5.8E-15	17.83766	6.5466088	1.3339819	150.5244	1.136841
9	XL_HDL_L	0.188242	1.5007609	17.83273	7.1463320	1.3339819	150.4373	1.139375
10	XL_HDL_P	0.187543	-4.4E-17	17.68135	1.0386650	1.7449572	147.7771	1.138823
11	L_HDL_L	0.180503	6.0973373	17.64475	1.9775369	3.0202382	147.1371	1.133279
12	L_HDL_PL	0.172965	-3.5E-17	16.85793	1.4776518	2.0687125	133.6977	1.127373
13	GlycA	-0.19279	5.7501732	-16.6206	7.7545896	1.0021315	129.7629	1.142971
14	Unsaturation	0.185937	-4.7E-16	16.56997	1.7929326	2.1515191	128.9303	1.137556
15	XL_HDL_FC	0.172457	-2E-16	15.86339	1.6515472	1.8497329	117.576	1.126976
16	HDL_CE	0.157851	-4.6E-16	15.17332	7.2688845	7.6323287	106.959	1.115624
17	DHA	0.160168	7.1687594	14.45414	3.0569621	3.0209979	96.39134	1.117417
18	S_HDL_TG	-0.16024	-1.3E-16	-14.1096	4.1949207	3.9152593	91.50791	1.117475
19	XL_VLDL_L	-0.15871	7.7064098	-14.0916	5.4064787	4.7804654	91.25619	1.116291
20	XL_VLDL_TG	-0.15704	7.2210866	-13.9667	3.1208493	2.6215134	89.517	1.114999
21	HDL_C	0.144002	-3.3E-17	13.75016	6.2818830	5.0255064	86.53903	1.104966
22	HDL_FC	0.141699	-6.4E-18	13.66115	2.1296926	1.6263107	85.32811	1.103204
23	XXL_VLDL_CE	-0.154	4.5534688	-13.5891	5.6891308	4.1555390	84.35361	1.11265
24	XXL_VLDL_L	-0.15248	8.5438066	-13.509	1.6858019	1.1800613	83.27634	1.111478
25	L_VLDL_PL	-0.1521	9.8460974	-13.4789	2.5316793	1.7012885	82.87309	1.111185
26	L_VLDL_L	-0.15193	1.2301678	-13.4495	3.7630881	2.4315338	82.48004	1.111057
27	VLDL_TG	-0.15203	-2E-16	-13.4467	3.9102856	2.4330666	82.44199	1.111135
28	XL_VLDL_P	-0.15115	5.3183668	-13.4036	6.9719508	4.1831705	81.86856	1.110455
29	XL_VLDL_PL	-0.1508	6.4972395	-13.3413	1.6067259	9.3079293	81.0407	1.110186
30	XXL_VLDL_C	-0.14969	2.4222851	-13.2139	8.7475997	4.8986558	79.36053	1.109333
31	XXL_VLDL_TG	-0.14833	4.3822190	-13.119	3.0573699	1.6568972	78.11992	1.108287
32	L_VLDL_FC	-0.14802	-6.5E-20	-13.1029	3.7772596	1.9830613	77.91031	1.108045
33	L_VLDL_TG	-0.1471	-9.9E-17	-13.0251	1.0444767	5.3173362	76.90204	1.107338
34	XL_VLDL_C	-0.14662	5.3256163	-12.873	7.5147713	3.7131811	74.94601	1.106971
35	XL_VLDL_FC	-0.14592	-1.6E-17	-12.8658	8.2552696	3.9625294	74.85286	1.106438
36	L_VLDL_P	-0.14342	2.4577343	-12.6725	9.7956226	4.5712905	72.40132	1.104521
37	XXL_VLDL_PL	-0.14189	-6.1E-17	-12.5514	4.5288816	2.0563570	70.88412	1.103353
38	Total_TG	-0.1427	1.2565476	-12.5199	6.7259224	2.9735657	70.49225	1.103971
39	XL_VLDL_CE	-0.1426	1.7860660	-12.4501	1.6118755	6.9434636	69.62629	1.103892
40	XXL_VLDL_P	-0.1404	-3.2E-17	-12.3888	3.4583815	1.4525202	68.86998	1.102211
41	XXL_VLDL_FC	-0.14047	-7.4E-17	-12.3835	3.6941482	1.5136997	68.80464	1.102266
42	L_VLDL_C	-0.13994	-5.3E-17	-12.2697	1.5067127	6.0268511	67.41205	1.101861
43	VLDL_L	-0.13594	1.0713500	-11.8451	2.5654057	1.0022980	62.32433	1.09881
44	M_VLDL_TG	-0.13407	2.3406861	-11.7667	6.4960919	2.4803260	61.40448	1.097386

45	MUFA	-0.13244	3.4142440	-11.4128	3.9937603	1.4910038	57.32779	1.096143
46	L_VLDL_CE	-0.12589	1.3212552	-10.927	9.3291438	3.4071655	51.93281	1.091181
47	VLDL_PL	-0.12298	7.3491816	-10.651	1.8629922	6.6592062	48.9724	1.088978
48	M_HDL_C	0.111648	-2.3E-16	10.48995	1.0329580	3.6153530	47.27949	1.080462
49	M_HDL_CE	0.111948	-2.2E-16	10.47349	1.2287988	4.2130246	47.10793	1.080686
50	M_HDL_FC	0.109744	1.4129149	10.41885	2.1826881	7.3338323	46.54021	1.079037
51	M_HDL_TG	-0.12108	1.2682950	-10.3988	2.6922827	8.8686960	46.33287	1.08755
52	VLDL_size	-0.11681	-1.9E-15	-10.3114	6.6975277	2.1638166	45.43244	1.084332
53	S_VLDL_TG	-0.11678	-2.3E-16	-10.2221	1.6858258	5.3437498	44.52054	1.08431
54	HDL_L	0.10619	-2E-16	9.94866	2.7106400	8.4331022	41.77764	1.076382
55	HDL_PL	0.103504	-3.5E-16	9.79621	1.2352995	3.7732785	40.28044	1.07438
56	VLDL_FC	-0.11259	7.4027796	-9.71106	2.8533600	8.5600802	39.45423	1.08117
57	ApoA1	0.101954	3.3953703	9.626509	6.5063186	1.9176518	38.64089	1.073226
58	Gly	0.105817	-1.9E-16	9.57847	1.0359992	3.0008253	38.18196	1.076103
59	SFA	-0.10965	-2.3E-16	-9.45078	3.5284345	1.0047067	36.97314	1.07897
60	S_VLDL_L	-0.10769	-1.1E-16	-9.31625	1.2610430	3.5309205	35.71716	1.077498
61	Leu	-0.10266	-2.2E-16	-9.12792	7.2807983	2.0052034	33.98887	1.07375
62	S_LDL_TG	-0.10486	-1.1E-16	-9.12477	7.4950564	2.0309185	33.96029	1.07539
63	HDL_TG	-0.10524	4.3909897	-9.05009	1.4873873	3.9663663	33.28493	1.075676
64	Total_BCAA	-0.1025	-2E-16	-9.04759	1.5217968	3.9947168	33.2624	1.07363
65	M_VLDL_L	-0.10435	1.5944468	-8.98841	2.6089738	6.7431938	32.73129	1.075011
66	S_VLDL_P	-0.10224	-7.3E-17	-8.85668	8.5549754	2.1776301	31.56156	1.07344
67	M_HDL_P	0.09312	-1.8E-16	8.713002	3.0640362	7.6829566	30.30538	1.066675
68	Albumin	0.100748	2.2343148	8.641204	5.7527360	1.4212641	29.68532	1.072329
69	Val	-0.09758	1.0272672	-8.59527	8.5856117	2.0904098	29.29125	1.069979
70	VLDL_C	-0.09742	3.5231299	-8.35035	7.0094116	1.6822587	27.22568	1.069861
71	VLDL_P	-0.09584	3.4645858	-8.23744	1.8090795	4.2806388	26.2935	1.068686
72	S_VLDL_CE	-0.09587	-2.1E-16	-8.23326	1.8732372	4.3708868	26.25924	1.068709
73	XS_VLDL_TG	-0.09165	-3.7E-16	-7.91158	2.6026306	5.9896157	23.67406	1.06559
74	Creatinine	-0.08258	-1E-16	-7.76783	8.1626461	1.8531412	22.55207	1.058911
75	M_VLDL_P	-0.08815	1.1477390	-7.5587	4.1528667	9.3024215	20.95634	1.063004
76	S_HDL_PL	-0.08461	-4.6E-16	-7.27463	3.5336360	7.8111954	18.85837	1.060397
77	S_VLDL_C	-0.08431	2.4576191	-7.22392	5.1358215	1.1205428	18.49228	1.060179
78	Ile	-0.08289	-3.1E-17	-7.20698	5.8158307	1.2526404	18.37056	1.059138
79	M_VLDL_PL	-0.08358	1.7384725	-7.15542	8.4764321	1.8025830	18.00185	1.059641
80	M_LDL_TG	-0.0825	1.1059306	-7.1406	9.4414204	1.9826982	17.89634	1.058854
81	Omega_3	0.08001	1.5143020	7.131222	1.0106630	2.0779678	17.82972	1.057025
82	VLDL_CE	-0.08342	-1E-16	-7.13073	1.0142461	2.0779678	17.82625	1.059526
83	S_HDL_L	-0.08305	4.0575449	-7.10948	1.1831494	2.3948085	17.67554	1.059253
84	S_VLDL_PL	-0.08237	1.3901784	-7.06593	1.6199548	3.2399096	17.36814	1.058754
85	M_HDL_L	0.07544	-2.7E-16	7.015137	2.3315333	4.6082071	17.012	1.053683
86	LDL_TG	-0.07855	-1.7E-17	-6.77857	1.2295274	2.4018676	15.38704	1.055953
87	M_LDL_CE	-0.0756	-3E-16	-6.46827	1.0020679	1.9350277	13.33992	1.053797
88	Gln	0.073748	-4.3E-16	6.299357	3.0180422	5.7617170	12.26581	1.052447
89	HDL_P	0.06812	-7.3E-17	6.227661	4.7787420	9.0205468	11.81847	1.048349
90	Total_FA	-0.07146	1.0603077	-6.16288	7.2075849	1.3454158	11.41865	1.050779
91	S_HDL_P	-0.06992	-2.3E-17	-5.96894	2.4068396	4.4433962	10.24674	1.049661
92	M_LDL_P	-0.06932	1.9975603	-5.92805	3.0891978	5.6411439	10.00438	1.049222

93	L_LDL_TG	-0.06827	2.1863235	-5.88496	4.0112401	7.2461112	9.750853	1.048457
94	IDL_TG	-0.06765	-2.6E-16	-5.83002	5.5820048	9.9763491	9.43024	1.048011
95	M_VLDL_FC	-0.06673	-1.4E-16	-5.70284	1.1860706	2.0974723	8.699581	1.047338
96	Total_P	0.057823	-5.6E-16	5.224126	1.7580381	3.0765667	6.093628	1.040894
97	S_LDL_CE	-0.06007	5.6499773	-5.13232	2.8745001	4.9785156	5.619932	1.042518
98	S_VLDL_FC	-0.05997	9.2305629	-5.12527	2.9840591	5.1155300	5.583916	1.042446
99	M_LDL_L	-0.059	1.4161511	-5.04205	4.6246148	7.8478312	5.162334	1.041742
100	M_HDL_PL	0.054469	-4.5E-16	5.016158	5.2927139	8.8917594	5.032578	1.038477
101	M_LDL_C	-0.05816	-1.2E-16	-4.96728	6.8160446	1.1337579	4.789468	1.041135
102	S_HDL_CE	-0.05764	4.9696560	-4.9091	9.1836229	1.5125967	4.503131	1.040762
103	Phosphatidylc	0.053531	3.6137649	4.907068	9.2789701	1.5134630	4.493215	1.037802
104	S_LDL_P	-0.05568	2.8481904	-4.76168	1.9262153	3.1115786	3.792792	1.039351
105	XS_VLDL_PL	-0.0523	-2.3E-16	-4.52255	6.1263467	9.8021548	2.686555	1.03692
106	Acetone	0.052833	4.8684887	4.506079	6.6212441	1.0494047	2.612443	1.0373
107	M_LDL_PL	-0.0519	-3.1E-16	-4.43132	9.3893109	1.4742095	2.279491	1.036628
108	Tyr	-0.05067	-1E-16	-4.35125	1.3567705	2.1105319	1.929096	1.035743
109	Cholines	0.047315	7.7621062	4.324113	1.5349526	2.3657985	1.811767	1.03334
110	Sphingomyelin	0.046531	2.3502380	4.280391	1.8697390	2.8556014	1.624295	1.032779
111	XS_VLDL_P	-0.04926	-4.1E-19	-4.26025	2.0463447	3.0971704	1.53859	1.034733
112	S_HDL_C	-0.04859	-3.9E-17	-4.15288	3.2895694	4.9343541	1.08843	1.034257
113	Remnant_C	-0.04832	-2.3E-16	-4.14208	3.4483820	5.1267980	1.043777	1.034057
114	XS_VLDL_L	-0.04762	-1.9E-16	-4.13335	3.5819435	5.2786536	1.007795	1.033561
115	LA	0.047654	3.1065025	4.110807	3.9501896	5.7707118	0.915168	1.033583
116	Lactate	-0.04558	-4.8E-16	-3.89096	0.0001	0.000145	0.038497	1.032098
117	Total_CE	0.041457	-3.5E-16	3.664437	0.000248	0.000355	-0.81445	1.029153
118	S_LDL_C	-0.04294	-2.5E-16	-3.66304	0.00025	0.000355	-0.81953	1.030213
119	ApoB	-0.04262	-1.5E-16	-3.64115	0.000272	0.000384	-0.89924	1.02998
120	XS_VLDL_FC	-0.04171	2.5723480	-3.62268	0.000292	0.000409	-0.9661	1.029336
121	S_LDL_L	-0.04068	8.4810774	-3.47201	0.000517	0.000718	-1.49884	1.028595
122	PUFA	0.03722	-3.4E-16	3.268786	0.001081	0.001489	-2.18161	1.026135
123	Phe	-0.03789	-8.4E-17	-3.22794	0.001248	0.001704	-2.31386	1.026612
124	Citrate	0.033218	-2.5E-16	2.889642	0.003859	0.005194	-3.3454	1.023292
125	LDL_P	-0.03384	-2.1E-16	-2.88914	0.003865	0.005194	-3.34684	1.023733
126	His	0.033442	-1.1E-16	2.865337	0.004168	0.005557	-3.41511	1.023451
127	bOHbutyrate	0.033007	6.5966249	2.813947	0.004896	0.006477	-3.56059	1.023142
128	Phosphoglyc	0.030397	-3E-16	2.760877	0.005767	0.007569	-3.70806	1.021293
129	non_HDL_C	-0.03196	5.3692532	-2.73622	0.006217	0.008097	-3.77561	1.022402
130	M_VLDL_C	-0.03028	2.1030542	-2.58754	0.00967	0.012496	-4.17016	1.02121
131	L_LDL_FC	0.029805	-1.6E-16	2.584761	0.009748	0.012501	-4.17732	1.020874
132	IDL_P	-0.02831	-3E-16	-2.43807	0.01477	0.018798	-4.5446	1.019817
133	IDL_FC	0.025854	-2.3E-16	2.278877	0.022679	0.028648	-4.9189	1.018082
134	LDL_PL	-0.02514	8.7788114	-2.14835	0.031692	0.039733	-5.20696	1.017575
135	Total_PL	0.023697	-9.1E-16	2.105596	0.035245	0.043861	-5.29762	1.016561
136	LDL_CE	-0.02399	-3.4E-16	-2.05214	0.040162	0.049612	-5.40841	1.016766
137	IDL_C	0.02277	2.2016384	2.011063	0.044325	0.054355	-5.49161	1.015908
138	IDL_CE	0.02239	2.2618503	1.979549	0.047761	0.058143	-5.5543	1.015641

Health-consious vs Sweet-tooth

	metabolite	logFC	AveExpr	t	P.Value	adj.P.Val	B	FC
1	DHA	0.33743	7.1687594	27.90099	8.8863724	1.4929105	377.2919	1.263504
2	Unsaturation	0.327786	-4.74E-16	26.76472	1.6641603	1.3978947	346.8142	1.255085
3	L_HDL_CE	0.282551	-1.21E-16	25.02469	3.2634172	1.8275136	302.5194	1.216344
4	L_HDL_C	0.28105	-5.84E-17	24.97149	1.2127069	5.0933691	301.2106	1.215079
5	L_HDL_L	0.271547	6.0973373	24.3217	8.9575751	3.0097452	285.4433	1.207102
6	XL_HDL_CE	0.27824	-9.00E-17	24.22035	1.0177374	2.8496648	283.0205	1.212714
7	L_HDL_P	0.273075	8.0693581	24.17833	2.7795347	6.6708833	282.0189	1.20838
8	L_HDL_FC	0.27093	1.7729533	24.05026	5.8807452	1.2349565	278.9764	1.206585
9	L_HDL_PL	0.262642	-3.55E-17	23.45467	6.9720474	1.3014488	265.0347	1.199674
10	XL_HDL_C	0.268722	-1.19E-16	23.23591	1.0896040	1.8305348	259.9993	1.20474
11	HDL_size	0.262816	-5.82E-15	23.21471	1.7734868	2.7085981	259.5137	1.199818
12	HDL_CE	0.258124	-4.64E-16	22.73411	9.8644370	1.3810211	248.6231	1.195923
13	XL_HDL_L	0.259677	1.5007609	22.53994	7.6540662	9.8914086	244.2861	1.197211
14	XL_HDL_PL	0.258087	-1.46E-16	22.42735	9.3869831	1.1264379	241.7878	1.195892
15	XL_HDL_P	0.257618	-4.36E-17	22.25394	4.3527219	4.8750485	237.9641	1.195503
16	HDL_C	0.245276	-3.29E-17	21.45902	1.3059924	1.3712920	220.8069	1.185319
17	HDL_FC	0.240857	-6.39E-18	21.27629	6.2697814	6.1960193	216.9493	1.181694
18	GlycA	-0.26547	5.7501732	-20.9701	3.8265032	3.5714030	210.5585	1.202028
19	XL_HDL_FC	0.232105	-1.99E-16	19.56224	7.6795150	6.7903080	182.3384	1.174548
20	Omega_3	0.238945	1.5143020	19.5135	1.9781818	1.6616727	181.396	1.180129
21	M_HDL_FC	0.207668	1.4129149	18.06456	1.1349487	9.0795902	154.434	1.15482
22	M_HDL_C	0.207938	-2.32E-16	17.90079	2.1291961	1.6259315	151.5152	1.155036
23	M_HDL_CE	0.206483	-2.19E-16	17.70022	7.4497026	5.4415219	147.9762	1.153872
24	ApoA1	0.198159	3.3953703	17.14335	1.1730470	8.2113293	138.3567	1.147234
25	XXL_VLDL_CE	-0.20884	4.5534688	-16.8852	9.3446841	6.2796277	133.9999	1.15576
26	XXL_VLDL_L	-0.20647	8.5438066	-16.7608	7.5268160	4.8634811	131.9238	1.153864
27	HDL_PL	0.192648	-3.49E-16	16.70643	1.8636829	1.1596249	131.0216	1.142859
28	S_HDL_TG	-0.20576	-1.32E-16	-16.6003	1.0848259	6.5089558	129.2688	1.153295
29	HDL_L	0.193341	-2.01E-16	16.5967	1.1523331	6.6755854	129.2088	1.143409
30	XXL_VLDL_C	-0.20506	2.4222851	-16.5856	1.3856614	7.7597039	129.0253	1.152735
31	XXL_VLDL_PL	-0.19991	-6.15E-17	-16.2026	7.2649349	3.9371260	122.7949	1.148629
32	XL_VLDL_L	-0.19899	7.7064098	-16.1878	9.2193079	4.8401366	122.5579	1.147892
33	XL_VLDL_TG	-0.1986	7.2210866	-16.1834	9.8999637	5.0399815	122.487	1.147583
34	XXL_VLDL_TG	-0.19944	4.3822190	-16.162	1.3993810	6.9145887	122.1427	1.148252
35	M_HDL_P	0.18536	-1.76E-16	15.89123	1.0624701	5.0998568	117.8356	1.137101
36	XXL_VLDL_FC	-0.19596	-7.42E-17	-15.8286	2.8609509	1.3351104	116.8503	1.145488
37	XL_VLDL_P	-0.19468	5.3183668	-15.8176	3.4037800	1.5455001	116.6775	1.144466
38	XL_VLDL_PL	-0.19387	6.4972395	-15.7154	1.7006552	7.5186864	115.0774	1.14383
39	XXL_VLDL_P	-0.19423	-3.16E-17	-15.7034	2.0521475	8.8400202	114.8905	1.144113
40	L_VLDL_PL	-0.19097	9.8460974	-15.5067	4.3975749	1.8469814	111.8424	1.141534
41	VLDL_TG	-0.18582	-2.03E-16	-15.0587	4.1035934	1.6814724	105.0421	1.137465
42	XL_VLDL_FC	-0.18528	-1.59E-17	-14.9676	1.6081440	6.4325760	103.6842	1.137036
43	L_VLDL_FC	-0.18228	-6.48E-20	-14.7845	2.4461179	9.5569260	100.978	1.134674
44	L_VLDL_L	-0.18182	1.2301678	-14.7473	4.2372294	1.6178512	100.4319	1.134313
45	Albumin	0.184952	2.2343148	14.53494	9.4802231	3.5392832	97.34254	1.136779
46	L_VLDL_P	-0.17832	2.4577343	-14.4364	3.9481997	1.4419512	95.92456	1.131563
47	XL_VLDL_C	-0.17929	5.3256163	-14.4233	4.7687568	1.7045768	95.73689	1.132325

48	M_HDL_L	0.167501	-2.74E-16	14.27147	4.2137162	1.4748006	93.57146	1.123111
49	L_VLDL_TG	-0.17445	-9.91E-17	-14.1535	2.2553815	7.7327367	91.90439	1.128532
50	Total_TG	-0.1758	1.2565476	-14.132	3.0535737	1.0260007	91.6033	1.12959
51	HDL_P	0.16189	-7.31E-17	13.56099	8.3364121	2.7461122	83.7427	1.118752
52	XL_VLDL_CE	-0.1667	1.7860660	-13.3358	1.7289258	5.5857603	80.73131	1.122491
53	MUFA	-0.16851	3.4142440	-13.3052	2.5995223	8.2399953	80.3263	1.123895
54	VLDL_size	-0.16381	-1.90E-15	-13.2497	5.4400707	1.6924664	79.59296	1.120242
55	L_VLDL_C	-0.164	-5.35E-17	-13.1747	1.4668982	4.4807073	78.60796	1.120388
56	VLDL_L	-0.15614	1.0713500	-12.4654	1.3317864	3.9953592	69.56119	1.114298
57	Phosphatidylc	0.147852	3.6137649	12.41839	2.3927361	7.0522748	68.97979	1.107919
58	M_VLDL_TG	-0.15297	2.3406861	-12.3007	1.0283668	2.9787178	67.53303	1.111853
59	M_HDL_PL	0.141454	-4.54E-16	11.93598	8.6718867	2.4692830	63.13399	1.103016
60	Total_P	0.141222	-5.62E-16	11.69043	1.5946377	4.4649857	60.24666	1.102839
61	S_LDL_TG	-0.14643	-1.05E-16	-11.675	1.9109467	5.2629351	60.06725	1.106827
62	Sphingomyelin	0.138372	2.3502380	11.66295	2.2006317	5.9630020	59.92732	1.100663
63	S_VLDL_TG	-0.14436	-2.28E-16	-11.5785	5.8980070	1.5728018	58.94993	1.105241
64	Cholines	0.135673	7.7621062	11.36076	7.2420143	1.9010287	56.46413	1.098605
65	VLDL_PL	-0.14109	7.3491816	-11.1962	4.6730057	1.2077922	54.61651	1.102734
66	L_VLDL_CE	-0.1382	1.3212552	-10.991	4.6088514	1.1731621	52.34903	1.100532
67	M_HDL_TG	-0.12655	1.2682950	-9.95826	2.4618030	6.1728792	41.5747	1.09168
68	Creatinine	-0.1138	-9.99E-17	-9.80783	1.1013449	2.7209698	40.0934	1.082074
69	Phosphoglyc	0.11719	-3.02E-16	9.752598	1.8983771	4.6221355	39.5552	1.08462
70	Total_CE	0.119767	-3.48E-16	9.699743	3.1875114	7.6500273	39.04297	1.086559
71	M_LDL_TG	-0.12165	1.1059306	-9.64699	5.3321978	1.2617031	38.53448	1.087979
72	VLDL_FC	-0.12111	7.4027796	-9.57046	1.1192771	2.6116466	37.80176	1.087568
73	XS_VLDL_TG	-0.11701	-3.67E-16	-9.25464	2.2464720	5.1699629	34.83934	1.084484
74	IDL_CE	0.113492	2.2618503	9.193796	3.9587679	8.9874732	34.27998	1.081844
75	IDL_C	0.113203	2.2016384	9.160831	5.3728872	1.2035267	33.97846	1.081627
76	HDL_TG	-0.11612	4.3909897	-9.14897	5.9954280	1.3253051	33.87024	1.083814
77	S_VLDL_P	-0.11515	-7.35E-17	-9.13924	6.5588083	1.4310127	33.78159	1.083085
78	S_VLDL_L	-0.11454	-1.14E-16	-9.0795	1.1362986	2.4474124	33.23918	1.08263
79	PUFA	0.112245	-3.40E-16	9.032191	1.7514918	3.7246914	32.81217	1.080909
80	IDL_FC	0.108308	-2.33E-16	8.747155	2.2666318	4.7599268	30.28647	1.077963
81	LDL_TG	-0.10971	-1.73E-17	-8.67533	4.2670204	8.8501165	29.66273	1.079012
82	L_LDL_FC	0.101494	-1.59E-16	8.064744	7.5295172	1.5426328	24.56756	1.072884
83	M_VLDL_L	-0.09837	1.5944468	-7.76375	8.4295375	1.7062196	22.19218	1.070564
84	IDL_L	0.095797	3.6370159	7.735	1.0567700	2.1135401	21.97002	1.068655
85	Total_C	0.096425	-5.60E-16	7.723241	1.1588711	2.2904747	21.87938	1.069121
86	VLDL_P	-0.09751	3.4645858	-7.67924	1.6345517	3.1930777	21.54145	1.069925
87	IDL_PL	0.094547	1.3736073	7.65463	1.9795972	3.8226704	21.35329	1.06773
88	S_VLDL_CE	-0.09543	-2.13E-16	-7.5095	6.0513888	1.1552651	20.25591	1.068386
89	L_LDL_TG	-0.09319	2.1863235	-7.36036	1.8672789	3.5247512	19.15001	1.066724
90	VLDL_C	-0.09263	3.5231299	-7.27487	3.5273355	6.5843596	18.52608	1.066315
91	SFA	-0.08859	-2.28E-16	-6.99625	2.6679410	4.9254296	16.54305	1.063334
92	Total_PL	0.085199	-9.06E-16	6.936311	4.0821672	7.4543923	16.12658	1.060834
93	IDL_TG	-0.08695	-2.56E-16	-6.86578	6.7036920	1.2109895	15.64103	1.062126
94	LDL_FC	0.083726	-8.80E-17	6.601024	4.1312178	7.3834530	13.86269	1.059751
95	L_LDL_C	0.076253	4.1314792	6.021508	1.7421440	3.0808441	10.21365	1.054276

96	M_VLDL_P	-0.07629	1.1477390	-5.99412	2.0623631	3.6091355	10.04947	1.054304
97	S_VLDL_C	-0.07505	2.4576191	-5.89234	3.8362951	6.6443049	9.445902	1.053399
98	Lactate	-0.07493	-4.83E-16	-5.86114	4.6307974	7.9385098	9.262965	1.053313
99	Total_FC	0.071714	4.1548594	5.706829	1.1586479	1.9661904	8.37234	1.050965
100	S_VLDL_PL	-0.07219	1.3901784	-5.67452	1.3998000	2.3516640	8.188883	1.051314
101	LA	0.070926	3.1065025	5.606019	2.0831636	3.4650642	7.803325	1.050391
102	His	0.069566	-1.12E-16	5.461322	4.7524751	7.8276061	7.004294	1.049401
103	VLDL_CE	-0.06937	-1.00E-16	-5.43337	5.5604082	9.0694037	6.852327	1.049259
104	M_VLDL_PL	-0.06813	1.7384725	-5.34465	9.1056807	1.4709176	6.375229	1.048358
105	XS_VLDL_CE	0.064928	-2.25E-16	5.255164	1.4860554	2.3776887	5.90191	1.046033
106	L_LDL_CE	0.065018	-3.38E-16	5.124257	3.0001896	4.7550175	5.223903	1.046098
107	Acetone	0.064022	4.8684887	5.003123	5.6633658	8.8920137	4.61173	1.045376
108	Gln	0.060479	-4.34E-16	4.733367	2.2153587	3.4461136	3.301025	1.042812
109	S_LDL_FC	0.059968	-2.26E-16	4.685213	2.8053379	4.3238235	3.074682	1.042443
110	L_LDL_L	0.057048	-3.72E-16	4.499327	6.8349681	1.0438860	2.222633	1.040335
111	S_HDL_FC	0.054756	4.0871187	4.384873	1.1633198	1.7607002	1.715152	1.038684
112	Omega_6	0.054241	5.4126847	4.32339	1.5399914	2.3099871	1.447934	1.038313
113	Ile	-0.05336	-3.06E-17	-4.25092	2.1335288	3.1719721	1.137782	1.037679
114	Total_FA	-0.05236	1.0603077	-4.13786	3.5122946	5.1760131	0.664446	1.036962
115	L_HDL_TG	0.050323	-1.85E-17	4.069856	4.7127204	6.8846699	0.385846	1.035497
116	M_VLDL_CE	0.05104	1.0407764	4.014399	5.9699704	8.6461641	0.162075	1.036011
117	Clinical_LDL_C	0.048593	-1.09E-16	3.81499	0.000136	0.000196	-0.6172	1.034256
118	Total_BCAA	-0.04664	-1.98E-16	-3.77184	0.000162	0.000231	-0.7806	1.032853
119	Leu	-0.0461	-2.22E-16	-3.75599	0.000173	0.000244	-0.84016	1.032472
120	XS_VLDL_C	0.045854	8.0342097	3.690748	0.000224	0.000313	-1.08267	1.032294
121	M_LDL_CE	-0.04694	-2.97E-16	-3.67959	0.000234	0.000325	-1.12371	1.033068
122	Ala	-0.04367	-3.34E-16	-3.43624	0.00059	0.000813	-1.9881	1.030734
123	M_LDL_FC	0.043682	7.2258201	3.416191	0.000636	0.000868	-2.05667	1.030741
124	Phe	-0.0436	-8.43E-17	-3.403	0.000667	0.000904	-2.10159	1.03068
125	L_LDL_PL	0.042781	-4.92E-17	3.367328	0.00076	0.001021	-2.22213	1.030097
126	LDL_C	0.042803	2.8162820	3.358024	0.000786	0.001048	-2.25337	1.030114
127	S_HDL_L	-0.04111	4.0575449	-3.22489	0.001261	0.001668	-2.69086	1.028908
128	M_VLDL_FC	-0.04081	-1.43E-16	-3.19609	0.001394	0.00183	-2.78318	1.028694
129	S_LDL_CE	-0.03864	5.6499773	-3.02441	0.002493	0.003246	-3.3163	1.027142
130	S_LDL_P	-0.03831	2.8481904	-3.00206	0.002683	0.003468	-3.38354	1.026913
131	M_LDL_P	-0.03827	1.9975603	-2.99847	0.002715	0.003482	-3.39427	1.02688
132	Val	-0.03698	1.0272672	-2.98439	0.002843	0.003618	-3.43632	1.025963
133	S_VLDL_FC	-0.03551	9.2305629	-2.78049	0.00543	0.006859	-4.02315	1.024918
134	Acetate	0.034348	-6.35E-17	2.678479	0.007399	0.009276	-4.30115	1.024094
135	S_HDL_PL	-0.03322	-4.64E-16	-2.61706	0.008872	0.011041	-4.46352	1.023293
136	M_LDL_L	-0.03236	1.4161511	-2.53426	0.011272	0.013924	-4.67647	1.022687
137	XS_VLDL_PL	-0.02949	-2.30E-16	-2.336	0.019496	0.023907	-5.15852	1.020648
138	Pyruvate	-0.02975	1.8351869	-2.32612	0.020017	0.024368	-5.18152	1.020838
139	Acetoacetate	0.028724	-9.09E-18	2.245575	0.024736	0.029897	-5.36536	1.02011
140	IDL_P	0.025715	-3.00E-16	2.029132	0.042451	0.050941	-5.82729	1.017984
141	LDL_CE	0.025807	-3.41E-16	2.022892	0.043091	0.051342	-5.83991	1.018049
142	LDL_L	0.025699	2.1712433	2.014918	0.043919	0.051961	-5.85599	1.017973

Omnivore vs Sweet-tooth

	metabolite	logFC	AveExpr	t	P.Value	adj.P.Val	B	FC
1	DHA	0.177263	7.1687594	15.60016	1.0305184	1.7312710	113.494	1.130736
2	Omega_3	0.158935	1.5143020	13.81443	2.5889357	2.1747060	87.48068	1.116462
3	Unsaturation	0.141848	-4.74E-16	12.32745	7.3947649	4.1410683	68.20878	1.103318
4	HDL_C	0.101273	-3.29E-17	9.430308	4.2879153	1.8009244	36.92001	1.07272
5	HDL_CE	0.100273	-4.64E-16	9.399568	5.7419610	1.9292989	36.63248	1.071976
6	HDL_FC	0.099157	-6.39E-18	9.322622	1.1877404	3.3256733	35.91687	1.071148
7	M_HDL_FC	0.097924	1.4129149	9.06619	1.2836903	3.0808568	33.57426	1.070233
8	ApoA1	0.096205	3.3953703	8.8584	8.4240765	1.7690560	31.72376	1.068958
9	M_HDL_C	0.096289	-2.32E-16	8.822542	1.1605320	2.1663264	31.40875	1.06902
10	L_HDL_L	0.091044	6.0973373	8.679141	4.1266134	6.9327106	30.16169	1.065141
11	M_HDL_CE	0.094535	-2.19E-16	8.625114	6.6203854	1.0111134	29.69714	1.067721
12	L_HDL_PL	0.089678	-3.55E-17	8.523642	1.5962190	2.2347066	28.83245	1.064132
13	Phosphatidylc	0.094322	3.6137649	8.431883	3.5070020	4.5321257	28.05929	1.067563
14	M_HDL_P	0.09224	-1.76E-16	8.416583	3.9956287	4.7947544	27.93119	1.066024
15	HDL_P	0.093771	-7.31E-17	8.360149	6.4516937	7.2258970	27.46069	1.067156
16	M_HDL_L	0.092061	-2.74E-16	8.348388	7.1263880	7.4827074	27.36303	1.065892
17	L_HDL_C	0.087845	-5.84E-17	8.307198	1.0085200	9.9665509	27.02209	1.062781
18	Sphingomyelin	0.091841	2.3502380	8.23899	1.7858235	1.6667686	26.4612	1.065729
19	HDL_PL	0.089144	-3.49E-16	8.227882	1.9591195	1.7322740	26.3703	1.063739
20	L_HDL_CE	0.086677	-1.21E-16	8.170556	3.1536747	2.6490868	25.9031	1.061921
21	L_HDL_FC	0.08557	1.7729533	8.084613	6.3997255	5.1197804	25.20877	1.061107
22	L_HDL_P	0.08462	8.0693581	7.97432	1.5702629	1.1991098	24.32844	1.060408
23	HDL_L	0.087151	-2.01E-16	7.962424	1.7286399	1.2626587	24.23422	1.06227
24	Cholines	0.088358	7.7621062	7.874721	3.4956722	2.4469705	23.54384	1.063159
25	IDL_CE	0.091102	2.2618503	7.854774	4.0985393	2.7542184	23.38789	1.065183
26	M_HDL_PL	0.086986	-4.54E-16	7.812063	5.7546152	3.7183667	23.05528	1.062149
27	IDL_C	0.090433	2.2016384	7.788957	6.9092098	4.2990638	22.87609	1.06469
28	Phosphoglyc	0.086793	-3.02E-16	7.687572	1.5317128	9.1902772	22.09613	1.062007
29	Total_P	0.083399	-5.62E-16	7.3479	2.0495521	1.1873267	19.55729	1.059511
30	HDL_size	0.077785	-5.82E-15	7.312805	2.6622604	1.4908658	19.3015	1.055396
31	Gly	-0.08221	-1.87E-16	-7.25747	4.0112362	2.1738312	18.90068	1.058642
32	IDL_FC	0.082454	-2.33E-16	7.087496	1.3868332	7.2808744	17.68841	1.058818
33	XL_HDL_CE	0.076453	-9.00E-17	7.083216	1.4303150	7.2816040	17.65825	1.054422
34	Albumin	0.084204	2.2343148	7.043058	1.9091749	9.4335703	17.37621	1.060102
35	XL_HDL_C	0.074687	-1.19E-16	6.873531	6.3494664	3.0477438	16.20316	1.053133
36	Total_CE	0.07831	-3.48E-16	6.750166	1.4957045	6.9799547	15.36746	1.05578
37	IDL_L	0.07744	3.6370159	6.655028	2.8668211	1.3016917	14.73328	1.055144
38	XL_HDL_L	0.071435	1.5007609	6.59945	4.1752730	1.8459101	14.36698	1.050762
39	XL_HDL_P	0.070074	-4.36E-17	6.44267	1.1864330	5.1107883	13.35015	1.049771
40	PUFA	0.075025	-3.40E-16	6.425509	1.3281764	5.5783409	13.24034	1.053379
41	Total_C	0.075104	-5.60E-16	6.402506	1.5443933	6.3282461	13.09359	1.053437
42	XL_HDL_PL	0.06868	-1.46E-16	6.352095	2.1454667	8.5818669	12.77384	1.048757
43	IDL_PL	0.073093	1.3736073	6.298409	3.0365396	1.1863689	12.43609	1.05197
44	GlycA	-0.07268	5.7501732	-6.1107	1.0005299	3.8202054	11.27768	1.05167
45	L_LDL_FC	0.071689	-1.59E-16	6.062887	1.3482429	5.0334401	10.98817	1.050946
46	XS_VLDL_CE	0.070066	-2.25E-16	6.035773	1.5951166	5.8256435	10.82502	1.049764

47	S_HDL_FC	0.068283	4.0871187	5.819859	5.9319581	2.1203594	9.551856	1.048468
48	Total_FC	0.066983	4.1548594	5.673236	1.4103419	4.9361967	8.713673	1.047524
49	LDL_FC	0.066288	-8.80E-17	5.5624	2.6768932	9.1779198	8.094237	1.047019
50	L_LDL_C	0.06463	4.1314792	5.432003	5.6030224	1.8826155	7.381111	1.045817
51	XL_HDL_FC	0.059648	-1.99E-16	5.350685	8.8074757	2.9012861	6.94494	1.042212
52	XS_VLDL_C	0.062346	8.0342097	5.340997	9.2911069	3.0017422	6.893415	1.044162
53	Total_PL	0.061502	-9.06E-16	5.329145	9.9178360	3.1437669	6.830504	1.043552
54	Val	0.060604	1.0272672	5.205729	1.9413426	6.0397327	6.183726	1.042902
55	L_LDL_CE	0.060197	-3.38E-16	5.049518	4.4475504	1.3585244	5.386783	1.042608
56	Clinical_LDL_C	0.060215	-1.09E-16	5.031489	4.8866638	1.4659991	5.296366	1.042621
57	XXL_VLDL_PL	-0.05802	-6.15E-17	-5.0048	5.6143607	1.6547589	5.163101	1.041035
58	Leu	0.056555	-2.22E-16	4.903941	9.4279161	2.7308446	4.66592	1.03998
59	Total_BCAA	0.055862	-1.98E-16	4.808732	1.5241039	4.3398213	4.205858	1.03948
60	S_HDL_C	0.057327	-3.92E-17	4.77768	1.7792066	4.9817785	4.057763	1.040536
61	XXL_VLDL_FC	-0.05549	-7.42E-17	-4.77054	1.8433903	5.0768783	4.02386	1.039213
62	XXL_VLDL_C	-0.05537	2.4222851	-4.76634	1.8821854	5.1001153	4.003937	1.039124
63	LDL_C	0.056709	2.8162820	4.735124	2.1962630	5.8567013	3.856356	1.04009
64	L_LDL_PL	0.056412	-4.92E-17	4.725935	2.2979216	6.0320444	3.813098	1.039877
65	XXL_VLDL_CE	-0.05484	4.5534688	-4.71929	2.3741894	6.1363665	3.781887	1.038745
66	L_LDL_L	0.055585	-3.72E-16	4.665939	3.0814716	7.7623828	3.532756	1.03928
67	XXL_VLDL_L	-0.05399	8.5438066	-4.66499	3.0957122	7.7623828	3.528353	1.038135
68	XXL_VLDL_P	-0.05383	-3.16E-17	-4.63211	3.6303474	8.9690936	3.376251	1.038017
69	Citrate	-0.0537	-2.53E-16	-4.55575	5.2342946	1.2744369	3.027221	1.037926
70	IDL_P	0.054026	-3.00E-16	4.537269	5.7143190	1.3714365	2.943593	1.038158
71	XXL_VLDL_TG	-0.05111	4.3822190	-4.40805	1.0456054	2.4741085	2.368477	1.03606
72	S_HDL_PL	0.051386	-4.64E-16	4.308804	1.6450808	3.8385218	1.938029	1.03626
73	M_VLDL_CE	0.051106	1.0407764	4.2782	1.8882252	4.3455046	1.807269	1.036059
74	non_HDL_C	0.050416	5.3692532	4.208968	2.5706625	5.8360987	1.514906	1.035564
75	S_LDL_FC	0.050404	-2.26E-16	4.191288	2.7793606	6.2257677	1.441006	1.035555
76	LDL_CE	0.049794	-3.41E-16	4.154274	3.2695973	7.2275310	1.287303	1.035117
77	S_HDL_CE	0.049947	4.9696560	4.148324	3.3556870	7.3214990	1.262724	1.035227
78	M_LDL_FC	0.049569	7.2258201	4.126006	3.6982091	7.9653736	1.170835	1.034956
79	Remnant_C	0.048969	-2.30E-16	4.093947	4.2487532	9.0353233	1.039711	1.034525
80	VLDL_size	-0.047	-1.90E-15	-4.04638	5.2108736	0.000108	0.847022	1.033116
81	S_HDL_P	0.0486	-2.33E-17	4.045804	5.2235919	0.000108	0.844722	1.034261
82	S_HDL_TG	-0.04552	-1.32E-16	-3.9086	9.2976547	0.00019	0.301655	1.032054
83	LDL_L	0.046479	2.1712433	3.878624	0.000105	0.000213	0.185498	1.032741
84	LDL_PL	0.045492	8.7788114	3.791805	0.00015	0.0003	-0.14588	1.032035
85	XL_VLDL_P	-0.04352	5.3183668	-3.76395	0.000167	0.000331	-0.25061	1.030629
86	XL_VLDL_PL	-0.04307	6.4972395	-3.71596	0.000203	0.000396	-0.42925	1.030305
87	XL_VLDL_TG	-0.04156	7.2210866	-3.60421	0.000313	0.000605	-0.8363	1.029224
88	S_LDL_TG	-0.04157	-1.05E-16	-3.52757	0.00042	0.000802	-1.10831	1.029233
89	S_HDL_L	0.041934	4.0575449	3.500821	0.000464	0.000876	-1.20186	1.029493
90	XL_VLDL_L	-0.04027	7.7064098	-3.48709	0.000489	0.000912	-1.2496	1.028309
91	M_VLDL_C	0.041705	2.1030542	3.475538	0.00051	0.000942	-1.28964	1.029329
92	XS_VLDL_FC	0.039994	2.5723480	3.387151	0.000707	0.001291	-1.59149	1.028109
93	XL_VLDL_FC	-0.03935	-1.59E-17	-3.38381	0.000716	0.001293	-1.60276	1.027654
94	L_VLDL_PL	-0.03887	9.8460974	-3.35962	0.000781	0.001396	-1.68393	1.027312

95	M_LDL_TG	-0.03915	1.1059306	-3.30406	0.000954	0.001687	-1.86817	1.027506
96	Omega_6	0.038732	5.4126847	3.285789	0.001018	0.001781	-1.9281	1.027211
97	Ala	-0.03911	-3.34E-16	-3.27538	0.001056	0.001829	-1.9621	1.027481
98	Total_L	0.037532	-6.17E-16	3.149657	0.001636	0.002804	-2.36408	1.026357
99	XS_VLDL_L	0.036424	-1.86E-16	3.082923	0.002051	0.003481	-2.57107	1.025568
100	MUFA	-0.03607	3.4142440	-3.03139	0.002436	0.004092	-2.72789	1.025318
101	His	0.036124	-1.12E-16	3.018369	0.002543	0.00423	-2.76709	1.025355
102	L_VLDL_P	-0.0349	2.4577343	-3.00693	0.002641	0.004349	-2.8014	1.024483
103	L_VLDL_FC	-0.03426	-6.48E-20	-2.95769	0.003101	0.005058	-2.94755	1.024032
104	ApoB	0.035419	-1.47E-16	2.951143	0.003168	0.005117	-2.96681	1.024855
105	M_LDL_C	0.03531	-1.17E-16	2.941117	0.003272	0.005235	-2.99621	1.024777
106	VLDL_TG	-0.03379	-2.03E-16	-2.91422	0.003568	0.005654	-3.07459	1.023696
107	Creatinine	-0.03122	-9.99E-17	-2.86357	0.004191	0.00658	-3.22024	1.021874
108	Total_TG	-0.0331	1.2565476	-2.83174	0.004632	0.007205	-3.31045	1.023206
109	XL_VLDL_C	-0.03267	5.3256163	-2.79739	0.005154	0.007925	-3.40669	1.022904
110	XS_VLDL_P	0.033141	-4.08E-19	2.795199	0.005189	0.007925	-3.41279	1.023237
111	L_HDL_TG	0.032395	-1.85E-17	2.788427	0.005299	0.00802	-3.43161	1.022708
112	L_LDL_P	0.033402	-2.67E-16	2.782137	0.005403	0.008104	-3.44905	1.023422
113	Tyr	0.032858	-9.97E-17	2.751898	0.005928	0.008813	-3.53233	1.023037
114	LDL_P	0.031535	-2.09E-16	2.625579	0.008653	0.01274	-3.87041	1.022099
115	LDL_TG	-0.03117	-1.73E-17	-2.62294	0.008721	0.01274	-3.87729	1.021837
116	S_LDL_C	0.03133	-2.49E-16	2.606175	0.009159	0.013265	-3.92093	1.021954
117	S_LDL_PL	0.031183	1.3105022	2.594224	0.009484	0.013618	-3.95186	1.02185
118	M_LDL_P	0.031052	1.9975603	2.589671	0.00961	0.013682	-3.96361	1.021757
119	L_VLDL_L	-0.02989	1.2301678	-2.58001	0.009883	0.013953	-3.98847	1.020932
120	Ile	0.02953	-3.06E-17	2.503872	0.012288	0.017203	-4.18112	1.02068
121	Acetate	0.029855	-6.35E-17	2.477914	0.013219	0.018354	-4.24548	1.02091
122	Lactate	-0.02935	-4.83E-16	-2.44372	0.014541	0.020023	-4.32924	1.020555
123	M_LDL_CE	0.028662	-2.97E-16	2.391561	0.016781	0.022921	-4.45477	1.020066
124	L_VLDL_TG	-0.02735	-9.91E-17	-2.36181	0.01819	0.024645	-4.52514	1.019139
125	S_VLDL_TG	-0.02758	-2.28E-16	-2.35472	0.018541	0.024919	-4.54179	1.019304
126	M_LDL_PL	0.02724	-3.10E-16	2.268149	0.023325	0.0311	-4.74099	1.01906
127	S_LDL_L	0.026948	8.4810774	2.243239	0.024887	0.032921	-4.79692	1.018855
128	M_LDL_L	0.026634	1.4161511	2.219725	0.026443	0.034706	-4.84916	1.018633
129	M_VLDL_FC	0.025913	-1.43E-16	2.159723	0.0308	0.040111	-4.97994	1.018124
130	XS_VLDL_TG	-0.02536	-3.67E-16	-2.13462	0.032798	0.042385	-5.03361	1.017732
131	L_LDL_TG	-0.02492	2.1863235	-2.09483	0.036192	0.046414	-5.11736	1.017423
132	L_VLDL_C	-0.02406	-5.35E-17	-2.05679	0.039712	0.050543	-5.19596	1.016814
133	XL_VLDL_CE	-0.02411	1.7860660	-2.05238	0.040139	0.050702	-5.20499	1.016849
134	S_VLDL_FC	0.024463	9.2305629	2.038815	0.041475	0.051998	-5.23261	1.017101

Supplementary Table 9: Multinomial logistic regression for differentially expressed protein adjusted for age, sex and BMI

	Profile 2 (Omnivore)		Profile 3 (Sweet-tooth)	
	Coefficient	Pvalue	Coefficient	Pvalue
LA	0.62	0.00	0.67	0.00
Gln	0.73	0.04	1.18	0.32
bOHbutyrate	1.24	0.28	5.18	1.62e-14
Acetoacetate	0.28	0.00	0.00	0.00
Acetone	0.00	0.00	4.921069334	0.00
ApoB	1.34	0.38	0.39	0.01
His	0.08	0.00	0.00	0.00
Tyr	3.70	0.01	0.28	0.01
S_HDL_PL	3.87	0.03	0.07	0.00
S_HDL_CE	0.12	0.00	0.13	0.00
M_LDL_C	25.28	2.26e-09	65.13	6.66e-16
S_LDL_L	0.03	9.23e-08	0.02	1.18e-11
S_HDL_L	0.92	0.81	3.65	0.02
Total_FA	1.06	0.00	1.12	5.33e-13
sex_f31_0_0Male	2.50	0.00	2.36	0.00
age_when_attended_assessment_centre_f21003_0_0	0.99	2.65e-06	0.98	0.00
body_mass_index_bmi_f21001_0_0	1.04	0.00	1.05	0.00

Supplementary Table 10: GO Term from Enrichment analysis of differentially expressed protein

Category	Code	Term	Count	%	PValue	Fold Enrichment	Bonferroni	Benjamini	FDR	group
GOTERM_BP_DIRECT	GO:0014823	response to activity	3	42.86	6E-05	2E+02	1E-02	9E-03	9E-03	Health-conscious vs Omnivore
GOTERM_BP_DIRECT	GO:0051384	response to glucocorticoid	3	42.86	9E-05	2E+02	2E-02	9E-03	9E-03	Health-conscious vs Omnivore
GOTERM_CC_DIRECT	GO:0005615	extracellular space	5	71.43	1E-03	7E+00	2E-02	2E-02	2E-02	Health-conscious vs Omnivore
GOTERM_CC_DIRECT	GO:0005576	extracellular region	5	71.43	2E-03	7E+00	3E-02	2E-02	2E-02	Health-conscious vs Omnivore
GOTERM_BP_DIRECT	GO:0042755	eating behavior	2	28.57	8E-03	2E+02	8E-01	5E-01	5E-01	Health-conscious vs Omnivore
GOTERM_BP_DIRECT	GO:0030073	insulin secretion	2	28.57	1E-02	2E+02	9E-01	5E-01	5E-01	Health-conscious vs Omnivore
KEGG_PATHWAY	hsa04060	Cytokine-cytokine receptor interaction	3	42.86	1E-02	1E+01	1E-01	1E-01	1E-01	Health-conscious vs Omnivore
GOTERM_BP_DIRECT	GO:0042531	positive regulation of tyrosine phosphorylation of STAT protein	2	28.57	2E-02	1E+02	1E+00	5E-01	5E-01	Health-conscious vs Omnivore
GOTERM_BP_DIRECT	GO:0014068	positive regulation of phosphatidylinositol 3-kinase signaling	2	28.57	2E-02	8E+01	1E+00	5E-01	5E-01	Health-conscious vs Omnivore
GOTERM_BP_DIRECT	GO:0007565	female pregnancy	2	28.57	2E-02	8E+01	1E+00	5E-01	5E-01	Health-conscious vs Omnivore
GOTERM_BP_DIRECT	GO:0032355	response to estradiol	2	28.57	3E-02	6E+01	1E+00	5E-01	5E-01	Health-conscious vs Omnivore
GOTERM_BP_DIRECT	GO:0032869	cellular response to insulin stimulus	2	28.57	3E-02	6E+01	1E+00	5E-01	5E-01	Health-conscious vs Omnivore
GOTERM_BP_DIRECT	GO:0045471	response to ethanol	2	28.57	3E-02	6E+01	1E+00	5E-01	5E-01	Health-conscious vs Omnivore
GOTERM_BP_DIRECT	GO:0050729	positive regulation of inflammatory response	2	28.57	3E-02	6E+01	1E+00	5E-01	5E-01	Health-conscious vs Omnivore
GOTERM_BP_DIRECT	GO:0042593	glucose homeostasis	2	28.57	3E-02	5E+01	1E+00	5E-01	5E-01	Health-conscious vs Omnivore
GOTERM_MF_DIRECT	GO:0005179	hormone activity	2	28.57	4E-02	5E+01	6E-01	8E-01	8E-01	Health-conscious vs Omnivore
GOTERM_BP_DIRECT	GO:0007165	signal transduction	3	42.86	4E-02	7E+00	1E+00	6E-01	6E-01	Health-conscious vs Omnivore
GOTERM_BP_DIRECT	GO:0043410	positive regulation of MAPK cascade	2	28.57	4E-02	4E+01	1E+00	6E-01	6E-01	Health-conscious vs Omnivore
GOTERM_BP_DIRECT	GO:0051897	positive regulation of protein kinase B signaling	2	28.57	5E-02	4E+01	1E+00	6E-01	6E-01	Health-conscious vs Omnivore
GOTERM_BP_DIRECT	GO:0006629	lipid metabolic process	2	28.57	6E-02	3E+01	1E+00	7E-01	7E-01	Health-conscious vs Omnivore
GOTERM_MF_DIRECT	GO:0005125	cytokine activity	2	28.57	6E-02	3E+01	8E-01	8E-01	8E-01	Health-conscious vs Omnivore
KEGG_PATHWAY	hsa04630	JAK-STAT signaling pathway	2	28.57	9E-02	2E+01	7E-01	6E-01	6E-01	Health-conscious vs Omnivore
GOTERM_BP_DIRECT	GO:0043567	regulation of insulin-like growth factor receptor signaling pathway	2	66.67	8E-04	2E+03	3E-02	3E-02	3E-02	Health-conscious vs Omnivore
GOTERM_MF_DIRECT	GO:0031995	insulin-like growth factor II binding	2	66.67	8E-04	2E+03	8E-03	7E-03	7E-03	Health-conscious vs Omnivore
GOTERM_MF_DIRECT	GO:0031994	insulin-like growth factor I binding	2	66.67	1E-03	1E+03	1E-02	7E-03	7E-03	Health-conscious vs Omnivore
GOTERM_MF_DIRECT	GO:0005520	insulin-like growth factor binding	2	66.67	2E-03	6E+02	2E-02	8E-03	8E-03	Health-conscious vs Omnivore
GOTERM_CC_DIRECT	GO:0005615	extracellular space	3	100.00	9E-03	1E+01	9E-02	6E-02	6E-02	Health-conscious vs Omnivore
GOTERM_BP_DIRECT	GO:0032355	response to estradiol	2	66.67	1E-02	1E+02	3E-01	2E-01	2E-01	Health-conscious vs Omnivore
GOTERM_CC_DIRECT	GO:0005576	extracellular region	3	100.00	1E-02	9E+00	1E-01	6E-02	6E-02	Health-conscious vs Omnivore
GOTERM_BP_DIRECT	GO:0007568	aging	2	66.67	2E-02	9E+01	4E-01	2E-01	2E-01	Health-conscious vs Omnivore
GOTERM_BP_DIRECT	GO:0090090	negative regulation of canonical Wnt signaling pathway	2	66.67	2E-02	8E+01	5E-01	2E-01	2E-01	Health-conscious vs Omnivore
GOTERM_MF_DIRECT	GO:0005102	receptor binding	2	66.67	4E-02	3E+01	4E-01	1E-01	1E-01	Health-conscious vs Omnivore
GOTERM_BP_DIRECT	GO:0014823	response to activity	3	60.00	2E-05	3E+02	4E-03	4E-03	4E-03	Health-conscious vs Sweet-tooth
GOTERM_CC_DIRECT	GO:0005576	extracellular region	5	100.00	1E-04	9E+00	2E-03	2E-03	2E-03	Health-conscious vs Sweet-tooth
GOTERM_CC_DIRECT	GO:0005615	extracellular space	4	80.00	3E-03	8E+00	4E-02	2E-02	2E-02	Health-conscious vs Sweet-tooth
GOTERM_BP_DIRECT	GO:0042755	eating behavior	2	40.00	5E-03	3E+02	6E-01	4E-01	4E-01	Health-conscious vs Sweet-tooth

GOTERM_BP_DIRECT	GO:0051384	response to glucocorticoid	2	40.00	9E-03	2E+02	8E-01	4E-01	4E-01	Health-conscious vs Sweet-tooth
GOTERM_BP_DIRECT	GO:0007565	female pregnancy	2	40.00	1E-02	1E+02	9E-01	4E-01	4E-01	Health-conscious vs Sweet-tooth
GOTERM_BP_DIRECT	GO:0032355	response to estradiol	2	40.00	2E-02	1E+02	9E-01	4E-01	4E-01	Health-conscious vs Sweet-tooth
GOTERM_BP_DIRECT	GO:0032869	cellular response to insulin stimulus	2	40.00	2E-02	9E+01	1E+00	4E-01	4E-01	Health-conscious vs Sweet-tooth
GOTERM_BP_DIRECT	GO:0045471	response to ethanol	2	40.00	2E-02	9E+01	1E+00	4E-01	4E-01	Health-conscious vs Sweet-tooth
GOTERM_BP_DIRECT	GO:0042593	glucose homeostasis	2	40.00	2E-02	8E+01	1E+00	4E-01	4E-01	Health-conscious vs Sweet-tooth
GOTERM_CC_DIRECT	GO:0005796	Golgi lumen	2	40.00	2E-02	8E+01	2E-01	9E-02	9E-02	Health-conscious vs Sweet-tooth
GOTERM_MF_DIRECT	GO:0005179	hormone activity	2	40.00	2E-02	7E+01	3E-01	4E-01	4E-01	Health-conscious vs Sweet-tooth
GOTERM_CC_DIRECT	GO:0005615	extracellular space	4	100.00	9E-04	1E+01	1E-02	9E-03	9E-03	Health-conscious vs Sweet-tooth
GOTERM_CC_DIRECT	GO:0005576	extracellular region	4	100.00	1E-03	9E+00	2E-02	9E-03	9E-03	Health-conscious vs Sweet-tooth
GOTERM_BP_DIRECT	GO:0040018	positive regulation of multicellular organism growth	2	50.00	6E-03	3E+02	3E-01	4E-01	4E-01	Health-conscious vs Sweet-tooth
KEGG_PATHWAY	hsa04935	Growth hormone synthesis, secretion and action	2	50.00	1E-02	7E+01	8E-02	8E-02	8E-02	Health-conscious vs Sweet-tooth
GOTERM_MF_DIRECT	GO:0005179	hormone activity	2	50.00	2E-02	8E+01	2E-01	3E-01	3E-01	Health-conscious vs Sweet-tooth
KEGG_PATHWAY	hsa04080	Neuroactive ligand-receptor interaction	2	50.00	4E-02	2E+01	2E-01	1E-01	1E-01	Health-conscious vs Sweet-tooth
GOTERM_CC_DIRECT	GO:0005788	endoplasmic reticulum lumen	2	50.00	4E-02	3E+01	5E-01	2E-01	2E-01	Health-conscious vs Sweet-tooth

Supplementary Table 11: Relative Risk of chronic diseases

profile	disease	rr	lower	upper	Pvalue
Health-conscious	ANAEMIA	0.776951	0.736199	0.819958	0
Health-conscious	ANGINA	0.614239	0.582707	0.647477	0
Health-conscious	ANXIETY	0.862837	0.817503	0.910684	8.43E-08
Health-conscious	ASTHMA	0.89703	0.865478	0.929733	2.71E-09
Health-conscious	ATRIAL FIBRILLIATION	0.78474	0.751526	0.819423	0
Health-conscious	BRONCHIECTASIS	0.824458	0.742155	0.915888	0.000321
Health-conscious	CANCER	0.939335	0.914511	0.964834	4.65E-06
Health-conscious	CHRONIC KIDNEY DISEASE	0.580415	0.544266	0.618964	0
Health-conscious	CHRONIC OBSTRUCTIVE PULMONAR	0.527254	0.488059	0.569596	0
Health-conscious	CHRONIC SINUSITIS	0.948135	0.851252	1.056044	0.332826
Health-conscious	CIRRHOSIS	0.526278	0.423957	0.653293	5.90E-09
Health-conscious	DEMENTIA	0.496233	0.38165	0.645218	1.68E-07
Health-conscious	DEPRESSION	0.780838	0.74288	0.820735	0
Health-conscious	DIABETES	0.650162	0.620362	0.681394	0
Health-conscious	ECZEMA OR DERMATITIS	0.85741	0.762286	0.964405	0.010345
Health-conscious	EPILEPSY	0.811781	0.72715	0.906262	0.000205
Health-conscious	GLAUCOMA	0.951799	0.894959	1.01225	0.115845
Health-conscious	HEART FAILURE	0.638406	0.591652	0.688856	0
Health-conscious	HEPATITIS	0.792608	0.627383	1.001345	0.051328
Health-conscious	HYPERTENSION	0.744334	0.726918	0.762168	0
Health-conscious	INFLAMMATORY BOWEL DISEASE	0.925983	0.885358	0.968471	0.00078
Health-conscious	IRRITABLE BOWEL SYNDROME	0.899438	0.843643	0.958924	0.00118
Health-conscious	MENINGITIS	0.875001	0.605004	1.26549	0.478146
Health-conscious	MIGRAINE	0.997107	0.919914	1.080778	0.943817
Health-conscious	MULTIPLE SCHLEROSIS	1.038096	0.882333	1.221357	0.652165
Health-conscious	MYOCARDIAL INFARCTION	0.637637	0.593702	0.684823	0
Health-conscious	OSTEOPOROSIS	1.324988	1.257946	1.395603	0
Health-conscious	PARKINSONS DISEASE	0.636132	0.53458	0.756975	3.44E-07
Health-conscious	PERIPHERAL VASCULAR DISEASE	0.864783	0.789946	0.946709	0.001656
Health-conscious	PROSTATE PROBLEM	0.541558	0.515277	0.56918	0
Health-conscious	RHEUMATOID ARTHRITIS	0.776157	0.712449	0.845562	6.67E-09
Health-conscious	SCHIZOPHRENIA	0.751377	0.580054	0.973301	0.03039
Health-conscious	STROKE	0.759445	0.713321	0.808552	0
Health-conscious	THYROID PROBLEM	0.993312	0.954339	1.033875	0.742438
Health-conscious	TUBERCULOSIS	0.525	0.285874	0.964152	0.037736
Health-conscious	VESTIBULAR DISORDER	0.845676	0.757405	0.944236	0.002881
Health-conscious	CONSTIPATION	0.917103	0.874657	0.961608	0.000345
Health-conscious	DYSPEPSIA	0.847489	0.812588	0.883888	1.24E-14
Health-conscious	HEARING LOSS	0.770565	0.722094	0.82229	3.77E-15
Health-conscious	DIVENTICULAR DISEASES OF INTESTI	0.822512	0.798793	0.846936	0
Health-conscious	ENDOMETRIOSIS	1.073027	0.979045	1.176032	0.131773
Omnivores	ANAEMIA	0.957744	0.911429	1.006414	0.087785
Omnivores	ANGINA	1.125636	1.076043	1.177514	2.63E-07
Omnivores	ANXIETY	0.953087	0.905883	1.002751	0.063738
Omnivores	ASTHMA	0.947444	0.915762	0.980223	0.001864

Omnivores	ATRIAL FIBRILLIATION	1.109511	1.066737	1.154	2.21E-07
Omnivores	BRONCHIECTASIS	0.980537	0.889333	1.081094	0.693139
Omnivores	CANCER	1.069992	1.043185	1.097489	1.73E-07
Omnivores	CHRONIC KIDNEY DISEASE	1.101177	1.04313	1.162454	0.000486
Omnivores	CHRONIC OBSTRUCTIVE PULMONAR	1.061844	0.996572	1.131391	0.063754
Omnivores	CHRONIC SINUSITIS	0.933469	0.84163	1.035329	0.192594
Omnivores	CIRRHOSIS	0.962825	0.805051	1.151518	0.678215
Omnivores	DEMENTIA	1.071531	0.867801	1.323089	0.520789
Omnivores	DEPRESSION	0.962085	0.918935	1.007261	0.098746
Omnivores	DIABETES	0.978001	0.938403	1.019269	0.29147
Omnivores	ECZEMA OR DERMATITIS	0.963909	0.863242	1.076316	0.513644
Omnivores	EPILEPSY	0.920655	0.83081	1.020217	0.114577
Omnivores	GLAUCOMA	1.010429	0.952879	1.071455	0.72877
Omnivores	HEART FAILURE	1.082916	1.014012	1.156503	0.017557
Omnivores	HEPATITIS	0.903399	0.726884	1.122779	0.359712
Omnivores	HYPERTENSION	1.100965	1.077369	1.125079	0
Omnivores	INFLAMMATORY BOWEL DISEASE	0.926273	0.887318	0.966939	0.000476
Omnivores	IRRITABLE BOWEL SYNDROME	0.860977	0.809531	0.915692	1.92E-06
Omnivores	MENINGITIS	0.999599	0.707245	1.412803	0.998188
Omnivores	MIGRAINE	0.816202	0.753686	0.883904	5.87E-07
Omnivores	MULTIPLE SCHLEROSIS	0.837133	0.712437	0.983655	0.030753
Omnivores	MYOCARDIAL INFARCTION	1.134214	1.066613	1.206099	5.90E-05
Omnivores	OSTEOPOROSIS	0.816134	0.773685	0.860912	8.95E-14
Omnivores	PARKINSONS DISEASE	1.098021	0.945323	1.275385	0.220955
Omnivores	PERIPHERAL VASCULAR DISEASE	0.904999	0.830611	0.986049	0.022546
Omnivores	PROSTATE PROBLEM	1.319027	1.266393	1.373849	0
Omnivores	RHEUMATOID ARTHRITIS	0.976151	0.902446	1.055877	0.546775
Omnivores	SCHIZOPHRENIA	1.132172	0.898076	1.427288	0.29354
Omnivores	STROKE	1.019511	0.963115	1.079209	0.505709
Omnivores	THYROID PROBLEM	0.974965	0.93808	1.0133	0.197564
Omnivores	TUBERCULOSIS	1.300761	0.797556	2.121453	0.292057
Omnivores	VESTIBULAR DISORDER	1.085742	0.980701	1.202034	0.113056
Omnivores	CONSTIPATION	0.942509	0.900855	0.986089	0.010245
Omnivores	DYSPEPSIA	0.982222	0.944346	1.021616	0.371276
Omnivores	HEARING LOSS	1.077059	1.015495	1.142355	0.013435
Omnivores	DIVENTICULAR DISEASES OF INTESTI	1.049433	1.021358	1.078279	0.000487
Omnivores	ENDOMETRIOSIS	0.942858	0.861824	1.031512	0.199381
Sweet-tooth	ANAEMIA	1.330717	1.265679	1.399096	0
Sweet-tooth	ANGINA	1.361683	1.300209	1.426063	0
Sweet-tooth	ANXIETY	1.218253	1.156546	1.283252	9.77E-14
Sweet-tooth	ASTHMA	1.18211	1.141427	1.224244	0
Sweet-tooth	ATRIAL FIBRILLIATION	1.128381	1.082598	1.176099	1.09E-08
Sweet-tooth	BRONCHIECTASIS	1.23275	1.1155	1.362324	4.07E-05
Sweet-tooth	CANCER	0.987645	0.961089	1.014935	0.371343
Sweet-tooth	CHRONIC KIDNEY DISEASE	1.452583	1.374943	1.534607	0
Sweet-tooth	CHRONIC OBSTRUCTIVE PULMONAR	1.610756	1.511818	1.716168	0
Sweet-tooth	CHRONIC SINUSITIS	1.138384	1.023102	1.266656	0.017347
Sweet-tooth	CIRRHOSIS	1.774385	1.489346	2.113977	1.38E-10

Sweet-tooth	DEMENTIA	1.660764	1.346207	2.04882	2.19E-06
Sweet-tooth	DEPRESSION	1.318925	1.259012	1.381689	0
Sweet-tooth	DIABETES	1.50504	1.444115	1.568534	0
Sweet-tooth	ECZEMA OR DERMATITIS	1.210976	1.081591	1.355839	0.000899
Sweet-tooth	EPILEPSY	1.333331	1.202767	1.478068	4.47E-08
Sweet-tooth	GLAUCOMA	1.04012	0.977504	1.106747	0.214331
Sweet-tooth	HEART FAILURE	1.376481	1.287289	1.471853	0
Sweet-tooth	HEPATITIS	1.387143	1.117291	1.72217	0.003029
Sweet-tooth	HYPERTENSION	1.192763	1.165921	1.220223	0
Sweet-tooth	INFLAMMATORY BOWEL DISEASE	1.174368	1.123708	1.227312	9.05E-13
Sweet-tooth	IRRITABLE BOWEL SYNDROME	1.300418	1.222809	1.382953	0
Sweet-tooth	MENINGITIS	1.143117	0.798005	1.637478	0.46572
Sweet-tooth	MIGRAINE	1.243936	1.14899	1.346728	7.11E-08
Sweet-tooth	MULTIPLE SCHLEROSIS	1.165402	0.990146	1.371679	0.065637
Sweet-tooth	MYOCARDIAL INFARCTION	1.312562	1.232052	1.398333	0
Sweet-tooth	OSTEOPOROSIS	0.917577	0.867293	0.970777	0.002777
Sweet-tooth	PARKINSONS DISEASE	1.360549	1.167647	1.585319	7.92E-05
Sweet-tooth	PERIPHERAL VASCULAR DISEASE	1.282209	1.176092	1.397901	1.70E-08
Sweet-tooth	PROSTATE PROBLEM	1.261072	1.208417	1.316021	0
Sweet-tooth	RHEUMATOID ARTHRITIS	1.306215	1.206212	1.414509	4.91E-11
Sweet-tooth	SCHIZOPHRENIA	1.147274	0.899261	1.463687	0.268918
Sweet-tooth	STROKE	1.272282	1.200246	1.34864	6.66E-16
Sweet-tooth	THYROID PROBLEM	1.036131	0.994796	1.079184	0.087487
Sweet-tooth	TUBERCULOSIS	1.308894	0.787147	2.176469	0.299498
Sweet-tooth	VESTIBULAR DISORDER	1.078131	0.967957	1.200844	0.171361
Sweet-tooth	CONSTIPATION	1.16381	1.110876	1.219265	1.69E-10
Sweet-tooth	DYSPEPSIA	1.20002	1.15236	1.249651	0
Sweet-tooth	HEARING LOSS	1.184907	1.114456	1.259813	5.80E-08
Sweet-tooth	DIVENTICULAR DISEASES OF INTESTI	1.149	1.116829	1.182098	0
Sweet-tooth	ENDOMETRIOSIS	0.991398	0.90133	1.090467	0.858899

Note: the p-values associated with these RRs were two-tailed, considering both directions of effect

Supplementary Table 12: Medical History, medication, and supplement taken for each food preference groups

	Health-conscious (n = 58,909)	Omnivore (n = 72,286)	Sweet-tooth (n = 50,543)	p-value
**Medical history**				
Diabetes	1,487 (2.5%)	2,200 (3.0%)	2,085 (4.1%)	< 0.001
Heart attack	460 (0.8%)	1,066 (1.5%)	824 (1.6%)	< 0.001
Angina	513 (0.9%)	1,111 (1.5%)	852 (1.7%)	< 0.001
Stroke	356 (0.6%)	541 (0.7%)	471 (0.9%)	< 0.001
High blood pressure	10,557 (18%)	16,128 (22%)	11,150 (22%)	< 0.001
**Medication**				
Blood pressure medication	435 (4.5%)	493 (4.1%)	339 (4.1%)	
Cholesterol lowering medication	224 (2.3%)	225 (1.9%)	176 (2.1%)	
Insulin	6 (<0.1%)	9 (<0.1%)	3 (<0.1%)	
Combination				
Blood pressure medication AND Cholesterol lowering medication	216 (2.2%)	274 (2.3%)	208 (2.5%)	
Blood pressure medication AND Insulin	0 (0%)	0 (0%)	4 (<0.1%)	
Cholesterol lowering medication AND Insulin	8 (<0.1%)	4 (<0.1%)	5 (<0.1%)	
Blood pressure medication AND Cholesterol lowering medication AND Insulin	12 (0.1%)	5 (<0.1%)	11 (0.1%)	
Other (Hormone replacement therapy, Oral contraceptive pill)	424 (4.4%)	370 (3.1%)	275 (3.3%)	
Combination with other medication	98 (1.0%)	79 (0.6%)	83 (1.0%)	
**Supplement**				
vitamin				<0.001
no	49,155 (83%)	63,135 (87%)	43,620 (86%)	
yes	9,748 (17%)	9,147 (13%)	6,915 (14%)	
multivitamin_minerals				<0.001
no	43,930 (75%)	57,335 (79%)	38,876 (77%)	
yes	14,973 (25%)	14,947 (21%)	11,659 (23%)	
fish_oil				<0.001
no	38,866 (66%)	48,791 (68%)	35,829 (71%)	
yes	20,037 (34%)	23,491 (32%)	14,706 (29%)	
glucosamine				<0.001
no	44,693 (76%)	57,290 (79%)	41,200 (82%)	
yes	14,210 (24%)	14,992 (21%)	9,335 (18%)	
calcium				<0.001
no	52,960 (90%)	68,039 (94%)	47,171 (93%)	
yes	5,943 (10%)	4,243 (5.9%)	3,364 (6.7%)	

zinc				<0.001
no	55,519 (94%)	69,599 (96%)	48,330 (96%)	
yes	3,384 (5.7%)	2,683 (3.7%)	2,205 (4.4%)	
iron				<0.001
no	56,514 (96%)	70,481 (98%)	48,731 (96%)	
yes	2,389 (4.1%)	1,801 (2.5%)	1,804 (3.6%)	
selenium				<0.001
no	56,839 (96%)	70,596 (98%)	49,252 (97%)	
yes	2,064 (3.5%)	1,686 (2.3%)	1,283 (2.5%)	

[1] n (%)

[2] Pearson's Chi-squared test
