# Supplementary material for: Artificial intelligence driven definition of food preference endotypes in UK Biobank volunteers is associated with distinctive health outcomes and blood based metabolomic and proteomic profiles
Source: J Transl Med. 2024 Oct 1;22:881. doi: 10.1186/s12967-024-05663-0 (PMC11443809; doi:10.1186/s12967-024-05663-0)
Supplement: Supplementary file 3 — Supplementary Material 3. [file 12967_2024_5663_MOESM3_ESM.pdf]

Supplementary Table 8: Limma differential expression for proteomics data

Health-conscious vs Omnivore

	logFC	AveExpr	t	P.Value	adj.P.Val	B	protein_type
LEP	-0.31393858	-0.17409801	-14.9368917	4.2723683661	1.2475315629	102.907714	down
APCS	-0.10530101	-0.08561453	-13.7799842	6.0604024717	8.8481876087	86.63126599	ns
CFH	-0.06874826	-0.04566867	-12.8292747	1.7940650014	1.7462232680	74.18468187	ns
GHR	-0.07238536	-0.02835094	-11.5937799	5.9870165991	4.3705221173	59.3688597	ns
CFI	-0.05039995	-0.0294017	-11.496479	1.8262666735	1.0665397373	58.26804843	ns
HGF	-0.0887776	-0.04313962	-11.4416886	3.4190665349	1.6639457136	57.64963038	ns
SERPINF1	-0.06236418	-0.03790164	-11.1321889	1.1280133632	4.7054271723	54.2049789	ns
FABP4	-0.13832311	-0.0562609	-10.9125914	1.2645848923	4.6157348570	51.82219129	down
RARRES2	-0.11859941	-0.0752127	-10.8821423	1.7657899375	5.7290073528	51.4941138	ns
IL1RN	-0.13524294	-0.00163616	-10.7464012	7.6820575370	2.2431608008	50.04661203	down
CD99L2	-0.05479181	-0.01799669	-10.471208	1.4281200537	3.7910095972	47.16834913	ns
CFB	-0.07267368	-0.0487602	-10.2567507	1.3276801387	3.1408269593	44.97563285	ns
SSC4D	-0.27004079	0.095505149	-10.2515852	1.3983133722	3.1408269593	44.92374118	down
ORM1	-0.04167374	-0.00760453	-10.2059765	2.2281577557	4.6473004619	44.4638033	ns
IL18R1	-0.07396011	-0.03755503	-10.0952175	6.8729948272	1.3379429930	43.35451606	ns
F9	-0.04102981	-0.02595323	-10.0518494	1.0676461809	1.9484542803	42.9232888	ns
FSTL3	-0.06588597	-0.0245828	-10.0231457	1.4257566688	2.4489467488	42.63917694	ns
OSM	-0.15736491	-0.07276487	-10.0001068	1.7974751655	2.9159041574	42.41127528	down
SERPIND1	-0.05794929	-0.03243131	-9.99001702	1.9851067267	3.0507956011	42.31212739	ns
ADM	-0.06124972	-0.05568744	-9.8340568	9.3606152734	1.3666498299	40.78571354	ns
CKB	0.125943441	0.014531382	9.821192684	1.0631172753	1.4782392589	40.66126853	ns
IGFBP2	0.14375176	-0.02548447	9.773703331	1.6961047397	2.2511935636	40.20196405	up
STC1	-0.08724617	-0.02194037	-9.38454961	7.1770211131	9.1116963696	36.52069379	ns
INHBC	-0.09074493	-0.10462579	-9.32562683	1.2506607840	1.5216372872	35.97715877	ns
CPM	-0.07356868	-0.0439586	-9.29483591	1.6678238748	1.9480182858	35.693427	ns
FURIN	-0.07279442	-0.04240017	-9.25460688	2.4270574857	2.7257722532	35.32528851	ns
ADAMTS15	-0.09148524	-0.04762613	-9.238886	2.8122369697	3.0413822043	35.18254429	ns
BPIFB2	-0.12394538	0.05996641	-9.22006455	3.3450925086	3.4884536161	35.00976336	ns
SEMA3F	-0.05297249	-0.02214061	-9.21227351	3.5997485891	3.6245744415	34.93999762	ns
CLEC4D	-0.12197123	-0.05301058	-9.19714956	4.1397199751	4.0293274424	34.80181562	ns
ITIH4	-0.04726545	0.006965556	-9.14966126	6.4095565826	6.0373887810	34.37084208	ns
TGFA	-0.08908901	0.008263632	-9.13449906	7.3771606559	6.7316590985	34.23455928	ns
NHLRC3	-0.05859318	-0.04392814	-9.08300334	1.1879093887	1.0511198227	33.77606571	ns
LILRA5	-0.06491803	-0.05301764	-9.06951741	1.3383924051	1.1494428891	33.65107406	ns
RTN4R	-0.06819992	-0.03712188	-8.89220369	6.6401486846	5.5397811883	32.07527708	ns
NTRK3	0.042379584	0.014814134	8.881865565	7.2845792929	5.9086032043	31.98470647	ns
LGALS9	-0.06136941	-0.04005442	-8.75170823	2.3176199842	1.8290406362	30.85284385	ns
SHBG	0.094192974	0.033412638	8.696687039	3.7558439579	2.8860695676	30.37689099	ns
DDC	0.087644464	0.041972203	8.586204403	9.8453986252	7.3714266630	29.43495795	ns
ASGR1	-0.05634839	-0.04427822	-8.57187436	1.1145718193	8.1363742810	29.31348028	ns
MPO	-0.08542623	-0.00808592	-8.49907647	2.0846302884	1.4846635225	28.69809744	ns
CD300A	-0.04983107	-0.01863664	-8.47223103	2.6265279479	1.8260622876	28.47638822	ns
BGLAP	-0.14400081	-0.05549405	-8.41068619	4.4281441397	3.0070188111	27.95801033	down
GUCA2A	0.056370907	0.007761604	8.275440622	1.3847174273	9.1894883811	26.84493536	ns
GFRA1	-0.04711645	-0.0221911	-8.26655173	1.4896899861	9.6664327992	26.76896505	ns
CST3	-0.04729642	-0.04064463	-8.17266432	3.2506326462	2.0634450710	26.00850251	ns
TNFRSF1A	-0.04785216	-0.02483846	-8.1447195	4.0916214996	2.5420286763	25.78222084	ns
CDHR5	-0.07051791	-0.06542682	-8.07150389	7.4563852606	4.5359677002	25.19622574	ns
CLMP	-0.03739035	-0.01790542	-8.0413379	9.5232828622	5.6750991750	24.95226647	ns
CNTN3	-0.05769752	-0.041332	-8.03238018	1.0249064286	5.9854535431	24.88351515	ns
FAM20A	-0.04631034	-0.04298675	-8.02897765	1.0530482520	6.0292174430	24.85479414	ns
EZR	-0.04281365	-0.02376248	-7.99728536	1.3611585994	7.6434290583	24.60312389	ns
CSF1	-0.04435398	-0.0134698	-7.95452347	1.9224215322	1.0591454479	24.26751112	ns
IGFBP1	0.227280632	0.008823951	7.945157548	2.0721410327	1.1204910769	24.19271774	up
C3	-0.07682246	-0.00887647	-7.94179333	2.1369942660	1.1345496830	24.17932998	ns
MFAP5	-0.07038582	-0.03717505	-7.92641023	2.4083063820	1.2557597563	24.0450956	ns
CHCHD10	-0.06137828	-0.04371587	-7.89756345	3.0337835484	1.5541487651	23.81974174	ns
AMBP	-0.03306156	-0.03425718	-7.86656454	3.8844063612	1.9555976853	23.5772611	ns
CEACAM8	-0.08207342	-0.03628282	-7.8608892	4.0712115945	2.0149047213	23.53966978	ns
ITGA5	-0.04235312	-0.02706417	-7.84087405	4.7665831645	2.3197371400	23.38001839	ns
RETN	-0.06857596	-0.01707067	-7.81955649	5.6463916113	2.7028628696	23.21712479	ns
A1BG	-0.02722322	-0.02054699	-7.8156026	5.8199391592	2.7410036040	23.18261308	ns

PGLYRP1	-0.07051884	-0.00844634	-7.79797117	6.6917435033	3.1015700047	23.0476259	ns
VWC2	-0.0641688	-0.04137541	-7.78224611	7.5814397425	3.4590318825	22.92963677	ns
RNASE6	-0.04311633	-0.02731617	-7.76797521	8.4774063110	3.8083117581	22.81783035	ns
PALM2	-0.05969522	-0.01341872	-7.75175438	9.6236640817	4.2577422906	22.69093416	ns
RELT	-0.04343376	-0.03067685	-7.73984852	1.0570644526	4.6069077639	22.60137615	ns
CCL22	-0.08587767	-0.04447282	-7.73266253	1.1190561279	4.8053586672	22.54988038	ns
PZP	-0.08448365	0.208852998	-7.69589583	1.4894843766	6.3033251880	22.2639796	ns
MMP9	-0.10208809	-0.07186344	-7.67269845	1.7852183582	7.4450397324	22.08990524	ns
SLC39A5	-0.08897319	-0.06035542	-7.6708975	1.8102665102	7.4450397324	22.07577862	ns
LCAT	-0.03566743	-0.02928045	-7.66860173	1.8419009668	7.4699316989	22.05701884	ns
NOMO1	-0.04019137	-0.02945913	-7.66197661	1.9398938489	7.7595753958	22.00802533	ns
PLAT	-0.08842021	-0.09331766	-7.65739387	2.0095057044	7.9294008878	21.97225623	ns
MZB1	-0.08309904	-0.05932045	-7.65282808	2.0823537768	8.1072973712	21.93869216	ns
LCN2	-0.06023072	-0.01664785	-7.64749102	2.1756044125	8.3589011641	21.9078972	ns
BMP10	0.044115562	0.01137317	7.634811092	2.3962023846	9.0868973549	21.80720042	ns
TGFBR2	-0.04803128	-0.01406392	-7.57195034	3.8842923612	1.4541197044	21.32867945	ns
F7	-0.04957172	-0.03198176	-7.56539351	4.0862997683	1.5103791548	21.28149458	ns
GGH	-0.05826794	-0.04112374	-7.52514998	5.5641960303	2.0309315510	20.98535171	ns
CCN5	-0.06425045	-0.03328942	-7.52054335	5.7560077636	2.0750052678	20.94559285	ns
SIT1	-0.09088575	-0.022559	-7.50586623	6.4425307218	2.2941694765	20.84149255	ns
PALM	-0.04426399	-0.01213568	-7.49974319	6.7433785709	2.3723693285	20.79162651	ns
C1RL	-0.02998284	-0.02239994	-7.48645148	7.4572519631	2.5922828252	20.69101421	ns
CRIP2	-0.06000811	-0.04293681	-7.48354245	7.6286216158	2.6206558962	20.67264377	ns
PAG1	-0.08889989	-0.01126442	-7.4568148	9.3464237256	3.1734368929	20.47811829	ns
CANT1	-0.0317409	-0.01148163	-7.42297022	1.2054429608	4.0458545351	20.22546265	ns
IL6	-0.11872306	-0.00484653	-7.39513972	1.4858070045	4.9033141952	20.02154581	ns
ADAM8	-0.04481302	-0.02241135	-7.39427988	1.4945032992	4.9033141952	20.01155885	ns
RNASE4	-0.039312	-0.03006874	-7.37662527	1.7088435681	5.5442480212	19.89167766	ns
CLEC6A	-0.07353688	-0.02380811	-7.37406454	1.7413018542	5.5874740816	19.87126327	ns
SNCG	-0.10811062	-0.09860755	-7.33883065	2.2631712760	7.1831088326	19.61125552	ns
BOC	0.037186632	0.00589777	7.317863261	2.6466492068	8.3099093375	19.4623676	ns
COLEC12	-0.03970365	-0.03369293	-7.28758812	3.3094449392	1.0280403428	19.2398787	ns
GPD1	-0.08078794	0.002666076	-7.26847774	3.8096207295	1.1709571084	19.09920094	ns
OXT	-0.2262838	0.000105097	-7.1493131	9.1104960233	2.7711092070	18.247644	down
IGSF9	-0.12628387	-0.0458287	-7.14515908	9.3898686174	2.8266408621	18.21881651	ns
RNASE1	-0.03366809	-0.01455021	-7.14340653	9.5102660424	2.8336711065	18.20716174	ns
CDHR2	-0.12116805	-0.17423928	-7.13814648	9.8851175796	2.9156104376	18.17311	ns
DDT	-0.06057242	0.017563997	-7.10615287	1.2452742465	3.6362007998	17.94388761	ns
GUSB	-0.09365998	-0.04504515	-7.10253766	1.2784472231	3.6961048432	17.91990209	ns
COL6A3	-0.04971354	-0.03672984	-7.0878987	1.4203174090	4.0660067003	17.81503226	ns
PGF	-0.03638396	-0.02196675	-7.08384011	1.4623423797	4.1456696591	17.78624545	ns
SFRP1	-0.06518951	-0.05033451	-7.0821585	1.4814838862	4.1595509114	17.78081905	ns
VSIG4	-0.05957065	-0.04366268	-7.06059553	1.7289219578	4.8080496352	17.62640794	ns
LILRB4	-0.06518645	-0.03482651	-7.02553097	2.2236714116	6.1053655036	17.3875893	ns
YAP1	-0.03709568	-0.01888789	-7.02375766	2.2496121810	6.1053655036	17.36745968	ns
CLEC4G	-0.05093279	-0.01516147	-7.02328493	2.2581488849	6.1053655036	17.3664057	ns
CD83	-0.04916672	-0.0265991	-7.01884109	2.3308975858	6.2442394044	17.33551491	ns
PLTP	0.05501556	0.006951874	7.000414872	2.6571513089	7.0535289291	17.20619755	ns
PON3	0.042475101	0.001823046	6.985257754	2.9596595078	7.7857709575	17.10196473	ns
LDLR	-0.08353745	-0.04661866	-6.96387989	3.4440473496	8.9791234473	16.95416338	ns
RBP7	-0.08598111	0.042330818	-6.95687438	3.6186422304	9.3508277106	16.90516704	ns
LGALS1	-0.05013377	-0.03511353	-6.93455813	4.2380084830	1.0855249798	16.7547188	ns
CA14	0.053839149	0.057241502	6.922334501	4.6166999562	1.1722403367	16.66595345	ns
DPT	-0.04117202	-0.06173397	-6.91001832	5.0345747412	1.2673239865	16.58226433	ns
VEGFB	-0.04025599	-0.0276868	-6.88525472	5.9969374459	1.4966715677	16.42271471	ns
RIDA	-0.07026283	0.006284974	-6.87323595	6.5212393319	1.6137304109	16.33970491	ns
SKAP1	-0.11042308	-0.01034146	-6.84825982	7.7613563979	1.9044672841	16.16712405	ns
GOLM2	-0.03321434	-0.02292632	-6.83548255	8.4777433702	2.0629175534	16.07471757	ns
DSG2	0.039182433	0.024864908	6.813761071	9.8612672097	2.3797438225	15.93092382	ns
COL4A1	0.058382563	0.018352175	6.807162967	1.0324174673	2.4710319711	15.88800586	ns
NOS3	-0.07608106	-0.03188146	-6.78935442	1.1684626701	2.7739113795	15.77257371	ns
FGA	-0.04264903	-0.01775554	-6.77189463	1.3179716330	3.1036106196	15.65478685	ns
FCAMR	-0.10021236	-0.02923245	-6.74274458	1.6091835030	3.7514223681	15.45177707	ns
CRELD1	-0.05127048	-0.04364029	-6.74199419	1.6187644465	3.7514223681	15.45510183	ns
GDF2	0.052132574	0.019448341	6.719252593	1.8910517132	4.3418301151	15.29923552	ns
BAIAP2	-0.09167438	-0.01061989	-6.71840081	1.9032679956	4.3418301151	15.2982022	ns

IGFBP4	-0.05581948	-0.04467782	-6.70078474	2.1458427212	4.8572563922	15.17827966	ns
CFP	-0.03094189	-0.0121674	-6.69412194	2.2442217623	5.0408673431	15.12839396	ns
LRIG1	-0.04915186	-0.02725682	-6.6658644	2.7200652377	6.0630461787	14.94111931	ns
CCL16	-0.07501758	-0.12633205	-6.66225238	2.7873959037	6.1660576052	14.91706507	ns
MMP8	-0.10553875	-0.03214886	-6.64868413	3.0561513184	6.7097457517	14.82792434	ns
COL15A1	-0.03443774	-0.01423475	-6.63335995	3.3900627566	7.3873009324	14.72746631	ns
SSC5D	-0.06326399	-0.05329165	-6.6229667	3.6375739745	7.8679377820	14.66195997	ns
COL18A1	-0.03217347	-0.03496446	-6.60559321	4.0894490839	8.7802877390	14.54859282	ns
APOA2	0.063496279	-0.00634954	6.597330321	4.3220937588	9.2120538509	14.4919093	ns
FRZB	-0.03676118	-0.03604365	-6.59496439	4.3938683659	9.2971707452	14.48175042	ns
CD300C	-0.04715706	-0.02114561	-6.58851817	4.5854825161	9.6328121921	14.43292515	ns
CD302	-0.03977051	-0.0288168	-6.56592182	5.3377943366	1.1133113902	14.29071385	ns
CREG1	-0.05368223	-0.00221972	-6.5524097	5.8441911516	1.2102863945	14.20570773	ns
CTSO	-0.0429205	-0.0237383	-6.54272833	6.2296163028	1.2786792458	14.13502068	ns
ADAMTS8	0.06319738	0.03360228	6.541979081	6.2620250739	1.2786792458	14.13237463	ns
FCN1	-0.07359025	-0.06135624	-6.53118507	6.7290775197	1.3645073859	14.06255331	ns
SERPINA3	-0.01868434	-0.00450974	-6.49593527	8.5045845935	1.7126473802	13.83503356	ns
AHNAK	-0.04215194	-0.01915085	-6.48969621	8.8694217713	1.7738843542	13.8026324	ns
CD300E	-0.05542135	-0.02326622	-6.47391762	9.8371119643	1.9540385670	13.69200163	ns
LAMP3	-0.07283832	-0.04649444	-6.43374328	1.2814840355	2.5283333673	13.43494967	ns
CFD	-0.02464755	-0.01534626	-6.41703402	1.4298988264	2.8022178344	13.32906318	ns
CSF3R	-0.03607187	-0.0081714	-6.4013869	1.5853680602	3.0861831572	13.2397372	ns
PLAUR	-0.03623183	-0.03703801	-6.36410005	2.0206893306	3.9075581758	13.00071659	ns
IFI30	-0.04951007	-0.04655162	-6.34318089	2.3147575185	4.4467710225	12.87001008	ns
NPC2	-0.03830631	-0.03478036	-6.32457006	2.6103140932	4.9817759165	12.75146148	ns
PRCP	-0.04318492	-0.04334977	-6.31993826	2.6884691950	5.0976169153	12.71788744	ns
CD22	-0.05633676	-0.00931444	-6.31832721	2.7161281425	5.1168349524	12.7054334	ns
SMOC1	-0.0442805	-0.05614878	-6.31654809	2.7475431637	5.1428372040	12.69394416	ns
TREML2	-0.04503292	-0.00246043	-6.28677642	3.3287535148	6.1676942079	12.51184864	ns
HSPG2	-0.03294035	-0.01699601	-6.28599016	3.3469069068	6.1676942079	12.51176598	ns
EPHA1	-0.04553235	-0.0608638	-6.28538775	3.3584362296	6.1676942079	12.50289638	ns
PRAP1	-0.05584985	-0.09612293	-6.28005055	3.4749333825	6.3417534230	12.46760142	ns
FETUB	-0.04614069	-0.03306284	-6.27742161	3.5349668381	6.4112442032	12.4545583	ns
SORD	-0.09283578	-0.03154459	-6.27364989	3.6207259246	6.5262467284	12.42804349	ns
WFIKKN2	0.048713918	0.029312183	6.26402323	3.8516157654	6.8998270154	12.37108285	ns
QPCT	-0.03567972	-0.03056179	-6.23618836	4.6010272834	8.1920729680	12.19910015	ns
SMAD5	-0.02746737	-0.03768428	-6.21085632	5.4058848943	9.5429809599	12.0437815	ns
SHISA5	-0.02911494	-0.02141815	-6.21024888	5.4251193128	9.5429809599	12.0356832	ns
CD74	-0.04523779	-0.03293008	-6.20820356	5.4957918372	9.6094084819	12.02213886	ns
LMNB2	-0.03368467	-0.02366368	-6.19747442	5.8827582632	1.0224794124	11.95706563	ns
CD5	-0.0446607	-0.01939922	-6.17791662	6.6575488239	1.1502983766	11.83619792	ns
ADH4	-0.11774517	-0.03484316	-6.15981552	7.4680397288	1.2827456475	11.73551225	ns
CXCL13	-0.07776076	0.011000087	-6.14410879	8.2378477501	1.4066968088	11.62988482	ns
APBB1IP	-0.06216326	0.008427928	-6.13678579	8.6283885977	1.4648194596	11.59070937	ns
INHBB	-0.06147362	-0.02699148	-6.10426686	1.0579238906	1.7856287633	11.3980846	ns
C1S	-0.02592139	-0.01995883	-6.09134634	1.1461143656	1.9233643377	11.31086742	ns
IGFBP6	-0.03553817	-0.01031694	-6.08395864	1.2006269268	2.0033317865	11.27295973	ns
BCHE	-0.03497072	-0.02785392	-6.07550293	1.2655940359	2.0997355595	11.2236517	ns
CNTN1	0.035329603	0.024234468	6.070480086	1.3049891520	2.1528634599	11.18455054	ns
GZMA	-0.04492125	-0.01291418	-6.04961515	1.4852405049	2.4364619518	11.05886094	ns
TNFRSF4	-0.04585353	-0.01322573	-6.03415796	1.6342947305	2.6660003426	10.96677695	ns
ITGAV	0.02091561	-0.00060153	6.031032505	1.6661176259	2.7028130377	10.94726449	ns
FN1	-0.03233228	-0.0144894	-6.00444598	1.9627437505	3.1664153324	10.79004594	ns
POLR2F	-0.04448518	-0.00156641	-5.99972735	2.0210694459	3.2425949352	10.76661999	ns
PAMR1	-0.03742066	-0.03393919	-5.99728265	2.0514375029	3.2668552399	10.75007109	ns
MAD1L1	-0.06316837	9.3353285535	-5.9955634	2.0737163472	3.2668552399	10.74365627	ns
CCDC80	-0.04381546	-0.03987433	-5.99499948	2.0805832938	3.2668552399	10.73831658	ns
BST2	-0.06489261	0.015131446	-5.99492502	2.0809420364	3.2668552399	10.7325824	ns
HSD11B1	0.052168357	0.013495465	5.973391729	2.3750641176	3.7086562693	10.60855179	ns
HSPB6	-0.04831713	-0.02650018	-5.96927049	2.4350912222	3.7821629622	10.58000315	ns
ENPP6	0.045783348	0.036315162	5.965759532	2.4894832855	3.8357711155	10.57045043	ns
PTS	-0.0712963	0.018822638	-5.96536016	2.4958784655	3.8357711155	10.56922662	ns
CA6	0.082090851	0.055544365	5.95635131	2.6358000393	4.0219343222	10.50880454	ns
IL12B	-0.06922625	-0.02336091	-5.95575735	2.6445595543	4.0219343222	10.50051323	ns
HAVCR1	-0.07568739	-0.07149016	-5.95215186	2.7034818035	4.0902418997	10.4792787	ns
LAIR1	-0.04643503	-0.03529584	-5.93149413	3.0665178875	4.6155836246	10.3629689	ns

CA5A	-0.11741176	-0.01591403	-5.91133061	3.4668701713	5.1914158463	10.24964837	ns
IL10RB	-0.03536483	-0.02621208	-5.90663099	3.5652336754	5.3114705777	10.21462881	ns
OLR1	-0.07475463	0.012470295	-5.89051034	3.9316409022	5.8276098652	10.127177	ns
ADIPOQ	0.081098953	0.057234325	5.884837039	4.0666857917	5.9973346019	10.08537372	ns
PCBD1	-0.05584627	0.020059573	-5.87297317	4.3703331855	6.4127502019	10.02662834	ns
LSP1	-0.05212219	-0.01668445	-5.86706484	4.5271178467	6.6095920562	9.984738399	ns
CNTN4	0.029900402	0.008460208	5.838467989	5.3745527763	7.8078080133	9.818676926	ns
FCAR	-0.05973932	-0.03028272	-5.8324192	5.5728569677	8.0558130424	9.783883637	ns
ADGRG2	0.034215201	0.033110771	5.824917759	5.8287283078	8.3841806202	9.742216316	ns
IL12A_IL12B	-0.09553978	-0.00296878	-5.82112045	5.9625106788	8.5345741089	9.720514944	ns
ADAM12	-0.04045907	0.001891794	-5.80013611	6.7592543237	9.6278159148	9.605937687	ns
CALB2	-0.03788039	-0.00223921	-5.79703391	6.8848495246	9.7591070932	9.586963772	ns
SCARB2	-0.03472246	-0.02528381	-5.78878468	7.2313563054	1.0200753822	9.540665038	ns
SMNDC1	-0.07278679	0.040771788	-5.78711885	7.2990274408	1.0246711599	9.520994875	ns
PLA2G2A	-0.06959662	0.038841251	-5.77948484	7.6389325642	1.0672575640	9.480610882	ns
GSTA1	-0.11140361	-0.00775496	-5.76905067	8.1259556034	1.1298947791	9.421251807	ns
AOC3	0.037241435	-0.00902145	5.755743623	8.7908030936	1.2165471579	9.343948905	ns
HJV	-0.05215504	-0.01201012	-5.75373198	8.8940297274	1.2250267360	9.32844597	ns
ANGPTL2	-0.05149416	-0.03470297	-5.74186037	9.5410802151	1.3068392216	9.26468127	ns
TREM2	-0.06272111	-0.06070506	-5.7412231	9.5775203228	1.3068392216	9.262438581	ns
SFRP4	-0.04797201	0.021823741	-5.73465621	9.9574392571	1.3523591921	9.230217148	ns
S100A11	-0.05657537	0.026371865	-5.72713772	1.0407214481	1.4069012168	9.185160822	ns
SIGLEC8	-0.05160535	-0.03199942	-5.71683958	1.1057787849	1.4879603925	9.129685844	ns
NT5C1A	-0.03652707	-0.01621346	-5.70638004	1.1751369579	1.5740366593	9.060278949	ns
SERPINB8	-0.06908599	-0.01466746	-5.69289997	1.2715689921	1.6954253228	8.98306606	ns
SOST	-0.04762427	-0.03158784	-5.6747853	1.4138177879	1.8765217912	8.887117495	ns
CCL18	-0.08819608	-0.03386284	-5.67258605	1.4323755940	1.8925505587	8.879384749	ns
CDH17	0.082639825	0.068689641	5.650464293	1.6286489819	2.1421869491	8.75016323	ns
ACVRL1	-0.02784932	-0.02774798	-5.629768	1.8363419532	2.4045374454	8.635883934	ns
CD72	-0.04722631	-0.01876961	-5.61652568	1.9826116273	2.5844758714	8.565768123	ns
CD4	-0.03555799	-0.02624323	-5.60539104	2.1137006471	2.7431137286	8.498021908	ns
BSG	-0.02212194	-0.0147872	-5.58819286	2.3330685257	3.0144071217	8.39809419	ns
IL16	-0.05009851	-0.03643258	-5.58670727	2.3530047802	3.0267726688	8.389367499	ns
KRT18	-0.09770724	-0.00335384	-5.58246194	2.4116807204	3.0886437296	8.371814302	ns
RBP5	-0.06961416	-0.0139115	-5.56372719	2.6850241437	3.4236989082	8.268320686	ns
GGT1	-0.05805808	0.002610699	-5.53901351	3.0918378746	3.9252898234	8.131004059	ns
AGRN	-0.03849993	-0.02782697	-5.53401325	3.1808746545	4.0208458836	8.101944767	ns
F10	-0.02886996	-0.01711836	-5.52917218	3.2695073481	4.1150695934	8.074136969	ns
OCLN	-0.04649976	-0.03554469	-5.51520887	3.5393059313	4.4355250298	7.996183835	ns
NPPC	-0.05262783	-0.00057409	-5.50811398	3.6853073969	4.5828008528	7.961195882	ns
GSTA3	-0.09399771	0.061480986	-5.50803136	3.6882130151	4.5828008528	7.968185023	ns
LGALS3BP	-0.03368113	0.011790123	-5.5059195	3.7308500076	4.6161364501	7.946200823	ns
EFNA4	-0.03224916	-0.02651292	-5.49013619	4.0804347541	5.0273710895	7.866371234	ns
NT5E	-0.06575944	0.037834143	-5.4631707	4.7494460009	5.8270513961	7.715307082	ns
FABP3	-0.05451273	-0.02465986	-5.44880681	5.1483185592	6.2899958966	7.635892259	ns
GLA	-0.04210695	-0.02396195	-5.43309126	5.6243213910	6.8429243591	7.562163771	ns
COCH	-0.04954734	-0.01111773	-5.42389739	5.9188032684	7.1713301011	7.50163651	ns
AZU1	-0.09075996	0.066564546	-5.4226731	5.9638212817	7.1960157614	7.513560994	ns
FNDC1	-0.05060898	-0.03292912	-5.41364373	6.2673740863	7.5311655688	7.446759063	ns
CCL7	-0.06989087	-0.02767532	-5.40720564	6.4973311244	7.7754946242	7.415241494	ns
STAB2	-0.03179322	-0.03118137	-5.39683367	6.8825247152	8.2028457830	7.356786007	ns
DPP6	0.039109731	0.021483137	5.392378477	7.0563346356	8.3758118439	7.33784302	ns
CD59	-0.02455709	-0.02144653	-5.37814156	7.6408040431	9.0328533627	7.275856569	ns
PARP1	-0.06372404	0.039102978	-5.36566815	8.1820891211	9.6337500942	7.193428695	ns
EFNA1	-0.02974588	-0.01533742	-5.36429095	8.2447399560	9.6442546611	7.186733979	ns
CSTB	-0.05004174	-0.05140729	-5.36405356	8.2570673469	9.6442546611	7.190959479	ns
PROS1	-0.02668351	-0.01725959	-5.3568797	8.5880896296	9.9909249874	7.143643262	ns
LTBP3	-0.06299079	0.009939478	-5.35144307	8.8534549002	1.0247351446	7.125443009	ns
HNMT	-0.0549409	-0.05734174	-5.35025381	8.9105394789	1.0247351446	7.11624587	ns
EGLN1	-0.06137494	0.007027714	-5.35015993	8.9137920119	1.0247351446	7.111050661	ns
PRSS8	-0.04710257	-0.05488094	-5.34741143	9.0505902213	1.0363813116	7.09903719	ns
CXCL17	-0.05065084	-0.04873897	-5.33434376	9.7234776252	1.1090841666	7.023137224	ns
NCF2	-0.09854042	0.06640549	-5.31784208	1.0647973415	1.2098086526	6.940866244	ns
ELOA	-0.06388591	0.022069614	-5.29210069	1.2258775964	1.3874273572	6.806751866	ns
MEGF9	-0.02376545	-0.00403363	-5.27850815	1.3200179649	1.4882055821	6.731617961	ns
BPIFA2	0.118178642	0.132636422	5.252775174	1.5193563543	1.7009274345	6.620719475	ns

TNFRSF14	-0.03226067	-0.0197507	-5.25252017	1.5203495219	1.7009274345	6.598426443	ns
FGR	-0.06721624	0.049065703	-5.24995571	1.5420189283	1.7185859812	6.592003438	ns
TMPRSS5	0.044346445	-0.00588975	5.245878927	1.5762852462	1.7500961669	6.568298822	ns
CSF3	-0.05615151	-0.0016873	-5.24069532	1.6212487146	1.7931993359	6.543720171	ns
PRTN3	-0.05245335	-0.00236403	-5.22342467	1.7801565943	1.9615310397	6.460527467	ns
FTCD	-0.09095753	0.059164144	-5.21719238	1.8400123427	2.0198631732	6.412532958	ns
LECT2	-0.06825122	-0.06099332	-5.20671225	1.9467467746	2.1290264351	6.357029579	ns
NPDC1	-0.03149875	-0.01434314	-5.20256271	1.9909526964	2.1692469678	6.3407047	ns
S100A12	-0.08441179	-0.022183	-5.19480062	2.0759549570	2.2534529645	6.30394902	ns
NBL1	-0.02713684	-0.01560759	-5.19373118	2.0874082738	2.2561388848	6.29186246	ns
HYOU1	-0.02380583	-0.01521823	-5.19314255	2.0938823212	2.2561388848	6.286593531	ns
GH1	0.205746206	0.313304763	5.172639159	2.3372727379	2.5091310274	6.187275692	up
C19orf12	-0.0696094	0.073978553	-5.1718311	2.3478091388	2.5112097748	6.188425803	ns
AMY2A	0.053322914	0.014897039	5.168611017	2.3895426100	2.5465198618	6.185006108	ns
GDF15	-0.04222535	-0.05624946	-5.15886608	2.5151158056	2.6705956918	6.110665563	ns
BGN	0.131157622	0.192235778	5.151650791	2.6147271114	2.7663054947	6.08583056	ns
ADH1B	-0.07957948	0.054649693	-5.14248342	2.7451064250	2.8937583975	6.037980713	ns
THY1	-0.02991281	-0.01898924	-5.12383747	3.0297290960	3.1823053814	5.931787708	ns
CFHR5	-0.04208062	-0.05389789	-5.11538493	3.1690132067	3.3166733203	5.896149022	ns
CHRDL1	-0.03276097	-0.02839594	-5.10133172	3.4128442804	3.5591090353	5.82162825	ns
GPR37	-0.07013006	-0.09750955	-5.09373311	3.5532122094	3.6923059257	5.790659994	ns
RBFOX3	-0.03725568	-0.03056084	-5.07955192	3.8271263889	3.9628400906	5.70787521	ns
EFEMP1	-0.03305588	-0.03396737	-5.07609839	3.8977776634	4.0217352570	5.69564299	ns
CNTN5	0.040138383	0.045766772	5.055866982	4.3334360137	4.4555046338	5.590327757	ns
PROK1	-0.06816058	-0.09842003	-5.05071189	4.4521709830	4.5359954875	5.567000355	ns
CALCA	-0.06582715	0.02962224	-5.05067447	4.4534083548	4.5359954875	5.570318518	ns
LRP1	0.029063639	0.014044627	5.050454202	4.4583243319	4.5359954875	5.567755002	ns
NXPH3	0.030875084	0.00862956	5.044782783	4.5932999374	4.6570957699	5.546246172	ns
CD6	-0.05808366	-0.03912866	-5.0435696	4.6208523118	4.6688196368	5.527502352	ns
TNFRSF10A	-0.03535982	-0.04336158	-5.02722299	5.0326855984	5.0673937750	5.45212445	ns
DPY30	-0.0650759	-0.00910265	-5.01973546	5.2317805685	5.2497591959	5.409219341	ns
BID	-0.07225411	0.014622055	-5.0081837	5.5556211263	5.5556211263	5.354896847	ns
CES1	-0.08764159	0.040288191	-5.00563497	5.6294456867	5.6102325615	5.342582172	ns
C1R	-0.01941815	-0.00150595	-5.00283931	5.7110661795	5.6722153891	5.325167843	ns
PDGFC	-0.03107596	-0.00982866	-4.99806772	5.8558516423	5.7963006087	5.312599268	ns
GNLY	-0.0559545	-0.04255065	-4.9969712	5.8877804965	5.8082158952	5.299053946	ns
CRHBP	-0.03025892	-0.04154438	-4.99059824	6.0852782754	5.9828325132	5.269431596	ns
CTSZ	-0.03379981	-0.02756025	-4.98583909	6.2368973572	6.1113222426	5.245942491	ns
SERPINA5	-0.03248073	0.018690177	-4.98403732	6.2954442469	6.1480592646	5.238961368	ns
FST	-0.05460072	0.010201476	-4.98209608	6.3581942920	6.1886424442	5.225507341	ns
SCLY	-0.05172656	-0.00612037	-4.97686409	6.5316881271	6.3363884821	5.196278902	ns
TNFRSF11A	-0.03908882	-0.0354379	-4.95672626	7.2446424384	7.0047536159	5.096327346	ns
ACE2	-0.05077885	0.018380216	-4.95342763	7.3707797651	7.1031937010	5.091827713	ns
CTSD	-0.04148084	0.007988467	-4.94134986	7.8467743850	7.5370332909	5.055269582	ns
UPB1	-0.09278785	-0.00720081	-4.92732854	8.4222523098	8.0632710638	4.953168195	ns
IDUA	-0.0484002	-0.06730344	-4.92256268	8.6295321464	8.2347169501	4.928569261	ns
VNN2	-0.04974856	-0.07590843	-4.91923413	8.7786308297	8.3497074993	4.919123433	ns
MNDA	-0.10605416	0.086793132	-4.91587375	8.9302762471	8.4663657927	4.902498942	ns
RNASET2	-0.02681709	-0.01705792	-4.91043079	9.1805709499	8.6599275392	4.872630649	ns
CPB2	-0.02227	-0.01355439	-4.91014642	9.1937586889	8.6599275392	4.870936415	ns
SELE	-0.05569116	-0.10645912	-4.90744378	9.3207929391	8.7513554284	4.855708587	ns
PI3	-0.05588973	-0.03348576	-4.90607531	9.3885058386	8.7866785413	4.860738536	ns
THBS4	-0.04833387	-0.02449886	-4.89875386	9.7432899233	9.0895867655	4.81909933	ns
FCN2	-0.04448853	-0.06413633	-4.88720894	1.0331745303	9.6078650595	4.765043396	ns
KIT	0.030779561	0.015016934	4.880955869	1.0663514127	9.8849083341	4.73193357	ns
CCL20	-0.08976374	0.014891783	-4.87926783	1.0753625090	9.9368940705	4.718512967	ns
HAVCR2	-0.03533055	-0.03890064	-4.86929674	1.1310966245	1.0418934206	4.67444598	ns
IL1RAP	0.047763778	0.143366245	4.868578966	1.1352056370	1.0423900817	4.670980057	ns
CST7	-0.10016563	0.069101993	-4.86656004	1.1468537999	1.0497846695	4.662288471	ns
TCTN3	-0.02905369	-0.02225449	-4.86130716	1.1775637294	1.0745269030	4.632691599	ns
DEFA1_DEFA1I	-0.05268357	0.009762663	-4.83665143	1.3336287888	1.2131451911	4.530591114	ns
MME	-0.08262676	0.083099284	-4.82858803	1.3883424093	1.2589937376	4.480992211	ns
NCAM2	0.028967608	0.025691835	4.814715456	1.4881297069	1.3453061127	4.412341149	ns
FCER2	-0.05189116	-0.0254095	-4.81264965	1.5035769390	1.3550755129	4.402478785	ns
IGSF8	-0.03030636	-0.02036144	-4.80456951	1.5655494426	1.4065859607	4.36602186	ns
REN	-0.07061053	-0.01075304	-4.79647034	1.6300672311	1.4600602193	4.327462592	ns

SERPING1	-0.01808208	-0.01211803	-4.79088468	1.6758919344	1.4965151219	4.297205866	ns
ANXA2	-0.06898489	0.022895266	-4.78871105	1.6947473896	1.5046051810	4.302922486	ns
LRP11	-0.03540702	-0.02729547	-4.78859394	1.6952572074	1.5046051810	4.290026108	ns
KRT5	-0.04948554	0.027792833	-4.76550658	1.9013777296	1.6824312032	4.186673158	ns
CTSS	-0.02539837	-0.01793221	-4.76143993	1.9447327855	1.7155950857	4.259506688	ns
TNFRSF1B	-0.03491384	-0.01985275	-4.76022662	1.9513732881	1.7162680726	4.153618432	ns
PILRA	-0.04207748	-0.07638171	-4.75925148	1.9608096797	1.7193886681	4.149015043	ns
ACHE	0.030852284	0.032052004	4.758156695	1.9717199074	1.7237790807	4.151538957	ns
SLITRK1	0.032125441	0.037649771	4.755564188	1.9972898672	1.7393943430	4.141026235	ns
WFDC2	-0.03512849	-0.01115656	-4.75517616	2.0014948605	1.7393943430	4.148222535	ns
MXRA8	0.029879685	0.034231663	4.74417748	2.1126569684	1.8305514385	4.085674761	ns
PTP4A3	-0.05009754	0.02628235	-4.74331027	2.1218524051	1.8330795926	4.083314194	ns
PLA2G1B	0.043027682	0.024443962	4.73005323	2.2644240716	1.9504773714	4.009333703	ns
C4BPB	-0.03929754	0.004697046	-4.71834228	2.3985859474	2.0599620489	3.955344752	ns
TNFSF14	-0.05986866	-0.01293491	-4.70609772	2.5471888405	2.1796344546	3.902309585	ns
MET	0.020181892	0.019595455	4.705623607	2.5528595325	2.1796344546	3.895510501	ns
ENTR1	-0.05536425	0.065945696	-4.69588347	2.6779595689	2.2797789916	3.860285662	ns
PLG	-0.02092017	-0.0213721	-4.68603211	2.8099103713	2.3851564780	3.814958613	ns
NUCB2	-0.03772989	-0.00627497	-4.66888645	3.0537708380	2.5846408252	3.722592582	ns
IFNLR1	-0.03812138	-0.01527757	-4.65866664	3.2093658640	2.7084821742	3.67945971	ns
FABP6	0.054156909	0.047857735	4.658012339	3.2200621915	2.7096776943	3.684037387	ns
ANGPTL4	-0.04556942	-0.05190204	-4.64511118	3.4270863026	2.8756011504	3.616737393	ns
DCXR	-0.05415097	0.019268869	-4.6272862	3.7350137588	3.1249971850	3.533306623	ns
ART3	0.032839064	0.031598704	4.619889878	3.8706407109	3.2292202502	3.501430748	ns
PXN	-0.05330033	-0.0001078	-4.6147112	3.9688787168	3.3017452572	3.485303035	ns
RET	-0.03916464	-0.01237474	-4.6132254	3.9968353041	3.3149843271	3.472633953	ns
IMMT	-0.04004818	-0.00701378	-4.61265456	4.0074981763	3.3149843271	3.465234856	ns
XG	-0.03023624	-0.006128	-4.6018715	4.2205008652	3.4813170978	3.41761919	ns
RNF149	-0.02682235	-0.02630013	-4.60103314	4.2382509108	3.4861106083	3.423031212	ns
HMOX2	-0.04615835	0.033659794	-4.59953265	4.2688710986	3.5014335978	3.415951068	ns
AMY2B	0.048389808	0.02244243	4.582468349	4.6346319560	3.7907914038	3.365740401	ns
ARSA	-0.04416228	0.019352267	-4.57861648	4.7173230228	3.8476489460	3.312503083	ns
ERI1	-0.03128106	0.003819022	-4.57442808	4.8127947142	3.9145851157	3.296694736	ns
NBN	-0.05042121	0.041310369	-4.56661415	4.9958065305	4.0521541858	3.26617421	ns
HHEX	-0.0714251	-0.03271949	-4.56420674	5.0523839918	4.0865331212	3.244088589	ns
ZHX2	-0.03116056	0.027861721	-4.56363374	5.0661814722	4.0865331212	3.241494372	ns
CRLF1	-0.02551403	-0.00557887	-4.54904488	5.4300959527	4.3680110693	3.17774073	ns
ERBB4	0.02294811	0.011304172	4.544760392	5.5415153595	4.4453914422	3.157890458	ns
CD8A	-0.04822879	-0.01229686	-4.53884473	5.6988417165	4.5590733732	3.128556768	ns
OGN	-0.03837779	-0.04045438	-4.52617516	6.0511446745	4.8276891939	3.075694765	ns
LBR	-0.06713739	0.035417087	-4.51974138	6.2373121381	4.9626570690	3.042722122	ns
KLRD1	-0.04498701	-0.02532786	-4.51702501	6.3176498514	5.0129178169	3.030380954	ns
APLP1	0.04925737	0.060587433	4.51492044	6.3813150064	5.0497126880	3.027357717	ns
LRCH4	-0.06354616	0.028761743	-4.51021192	6.5253049651	5.1497001346	3.013472198	ns
C2	-0.02322177	-0.03676342	-4.4898777	7.1783896016	5.6498376379	2.91453829	ns
TXNDC15	-0.02556961	0.000448018	-4.47944675	7.5373991453	5.9164530926	2.865549973	ns
SETMAR	-0.03026154	-0.01864916	-4.47114119	7.8366030133	6.1348205895	2.834447106	ns
TALDO1	-0.04072337	-0.00492585	-4.45261759	8.5425670717	6.6695978206	2.746806353	ns
S100A13	-0.0312533	-0.01072207	-4.44782846	8.7364980812	6.8028198392	2.73664573	ns
MSR1	-0.04426823	-0.07517273	-4.44424188	8.8813822629	6.8815071065	2.707023333	ns
APOM	0.025908064	0.01448561	4.444179174	8.8846855451	6.8815071065	2.712042236	ns
BTN3A2	-0.03523968	-0.03912376	-4.44260007	8.9500134660	6.9137670161	2.704155187	ns
COQ7	-0.03914351	0.063308906	-4.43196167	9.4038452083	7.2451788940	2.66568678	ns
PHOSPHO1	-0.02766991	-0.03324967	-4.4284318	9.5579968091	7.3445659691	2.644444623	ns
CELA2A	0.051924043	0.010258095	4.421945314	9.8482449709	7.5477363031	2.610122332	ns
CXCL10	-0.06408733	0.031406084	-4.41261734	1.0282146043	7.8553299652	2.571325586	ns
RSPO1	-0.03269836	-0.0177006	-4.41218384	1.0303395125	7.8553299652	2.573201172	ns
PROC	-0.02421276	-0.0123426	-4.40595932	1.0603133703	8.0627995869	2.544271843	ns
NPL	-0.03945173	-0.04347188	-4.39992078	1.0901377066	8.2563587535	2.513392812	ns
CD79B	-0.0406551	-0.02614915	-4.39914396	1.0940212703	8.2563587535	2.509024273	ns
PLIN1	-0.03458647	0.007576662	-4.39914284	1.0942502868	8.2563587535	2.521716939	ns
CXCL16	-0.02207586	-0.020789	-4.39613032	1.1093180229	8.3484758429	2.497177361	ns
CCL21	-0.0382039	-0.03138217	-4.39341528	1.1232835708	8.4318458273	2.487451612	ns
C5	-0.01320429	-0.00710707	-4.39203161	1.1304106665	8.4635875543	2.479170646	ns
NECTIN2	-0.02560536	-0.01902468	-4.38394599	1.1732671925	8.7619954025	2.449805075	ns
SPINK2	-0.03164226	-0.02333042	-4.37677892	1.2125241867	9.0320679218	2.423929835	ns

ITGA11	0.032009786	0.015084387	4.375314776	1.2205760848	9.0689113685	2.409866688	ns
CAPG	-0.05592804	-0.06983188	-4.36921829	1.2551490415	9.3021198001	2.385680081	ns
PTGDS	-0.02591132	-0.01244456	-4.36848196	1.2594494089	9.3103601875	2.386155382	ns
UMOD	0.050455822	0.009973374	4.343527862	1.4110446630	0.000104047	2.269130787	ns
LPL	0.044660657	0.009287675	4.334029945	1.4733822632	0.00010837	2.233438742	ns
ACY1	-0.05826149	-0.00095489	-4.32089868	1.5637492309	0.000114727	2.17706676	ns
LCP1	-0.04158404	0.058434104	-4.31829121	1.5821992672	0.00011579	2.160721896	ns
SERPINA6	0.014898954	0.020901187	4.314153434	1.6120785105	0.000117682	2.142834737	ns
TNFRSF10B	-0.0355469	-0.03318523	-4.31273028	1.6226280494	0.000118046	2.142813799	ns
PON2	-0.03250052	-0.03382266	-4.31227362	1.6261174854	0.000118046	2.146035261	ns
CD300LF	-0.0555183	-0.16256069	-4.31181334	1.6292010706	0.000118046	2.131247791	ns
IL18	-0.04275476	-0.0342467	-4.30931433	1.6478566975	0.000119103	2.128400396	ns
RNASE3	-0.10233288	0.064748747	-4.30732288	1.6627618886	0.000119883	2.120991489	ns
TAFA5	-0.03091226	-0.0233528	-4.30134833	1.7081474595	0.000122852	2.093705156	ns
CST1	0.087833171	0.048081719	4.293181976	1.7735889325	0.000127245	2.112468239	ns
ST6GAL1	-0.02671291	-0.02674019	-4.28892606	1.8062516424	0.000129271	2.034475305	ns
AMY1A_AMY1E	0.043285145	0.02286981	4.281249337	1.8704129654	0.000133536	2.029978485	ns
SIRPB1	-0.04102411	-0.03952379	-4.27980172	1.8819769120	0.000133965	2.002144185	ns
CD27	-0.03487177	-0.03557877	-4.27936045	1.8856073203	0.000133965	1.996246504	ns
AMOT	-0.0349296	0.015524282	-4.27336397	1.9372224809	0.000137298	1.980265671	ns
SNRPB2	-0.03780379	0.03765377	-4.25777942	2.0768303288	0.000146836	1.903151288	ns
ENG	0.015551504	0.000188558	4.244182864	2.2066641936	0.000155639	1.845070876	ns
CD38	-0.02753705	-0.02282377	-4.24025323	2.2457878579	0.00015784	1.834853663	ns
IGF2R	-0.02152423	-0.01476592	-4.23995845	2.2486747430	0.00015784	1.831183731	ns
LRTM2	0.02308434	0.040097765	4.237511468	2.2731550151	0.000159175	1.816832394	ns
GFRA3	0.025358595	0.018740388	4.233997578	2.3092050505	0.000161313	1.810475069	ns
NOTCH3	0.03061288	0.005703887	4.218449511	2.4738484947	0.000172402	1.73703546	ns
DNER	0.023779907	0.008813408	4.217482072	2.4846622732	0.000172743	1.739181987	ns
CFHR2	-0.0482625	-0.12068006	-4.21451243	2.5174848514	0.000174609	1.724858742	ns
PINLYP	-0.05716225	-0.06601671	-4.2074701	2.5973263334	0.00017972	1.703091804	ns
KLB	0.067455112	0.004789808	4.203863517	2.6391095795	0.00018218	1.688507904	ns
CCN3	-0.02835158	-0.02570954	-4.19631046	2.7281136480	0.00018788	1.645726078	ns
MCAM	0.029792524	0.016806363	4.184584946	2.8727708815	0.000197376	1.6004307	ns
S100P	-0.04821654	0.033779393	-4.1782339	2.9543744557	0.000202506	1.581577758	ns
SCARF2	0.024805489	-0.01020922	4.172110557	3.0343459599	0.000207501	1.543837233	ns
USP28	-0.03123993	0.036867591	-4.16678778	3.1059673758	0.000211902	1.52286332	ns
OMD	0.039421805	-0.00650596	4.16295366	3.1586383796	0.000214994	1.50927431	ns
TNFRSF9	-0.03452276	-0.0101551	-4.15319514	3.2964702320	0.000223853	1.472866637	ns
RRM2	-0.04533343	0.037135017	-4.15222966	3.3106682035	0.000224296	1.474712729	ns
CD274	-0.02950073	-0.00287671	-4.14785572	3.3741904474	0.00022807	1.450880078	ns
SMOC2	-0.03349809	-0.02954394	-4.14596248	3.4019916908	0.000229418	1.43923736	ns
PIK3IP1	-0.02180211	-0.01434001	-4.14163954	3.4667457402	0.000233246	1.42238744	ns
IL1B	-0.04982697	0.051392367	-4.13641505	3.5469366463	0.000238093	1.411761907	ns
RBKS	-0.04568098	0.009818999	-4.13332213	3.5949863172	0.000240436	1.398552936	ns
BAG3	-0.03625393	-0.04105563	-4.13309122	3.5983008968	0.000240436	1.391150019	ns
BLNK	-0.03151683	0.001154452	-4.13064213	3.6370748592	0.000242472	1.387173636	ns
BAP18	-0.05714324	0.013947613	-4.12962248	3.6526337067	0.000242954	1.36989326	ns
HEG1	-0.02223067	-0.03468438	-4.12162776	3.7820750634	0.000250992	1.348452716	ns
GC	-0.02119045	0.026826384	-4.11979836	3.8117394666	0.000252387	1.329650032	ns
VSNL1	-0.03053023	0.002937973	-4.11863296	3.8310357870	0.000253091	1.324923031	ns
LRRC25	-0.03436645	-0.0111443	-4.11279718	3.9293715527	0.000259001	1.30673305	ns
B2M	-0.02424474	-0.0022426	-4.10291524	4.1006321263	0.000269681	1.260776044	ns
PILRB	-0.0503712	-0.12970868	-4.099889	4.1548201788	0.000272631	1.25388908	ns
CEACAM19	-0.03170309	-0.03398322	-4.09278933	4.2837862242	0.000280463	1.220606087	ns
WDR46	-0.03097599	0.006255983	-4.08561534	4.4182024567	0.000288616	1.191194042	ns
RAB6A	0.040711324	0.055474091	4.076618922	4.5925575349	0.000299336	1.157542168	ns
CLSTN2	0.033841199	0.021206573	4.071083414	4.7026756037	0.000305831	1.130496253	ns
ALDH1A1	-0.04739833	0.032720692	-4.0651071	4.8249387127	0.000313085	1.11079977	ns
LYPD3	0.029142355	0.004749544	4.06380094	4.8520406204	0.000314145	1.106618593	ns
GHRL	0.070802388	0.053246639	4.057210616	4.9908653487	0.000322419	1.081187393	ns
SCARA5	-0.02470871	-0.04072241	-4.0545795	5.0469908586	0.000324984	1.064135128	ns
THBD	-0.0235982	-0.01595167	-4.05432352	5.0528341140	0.000324984	1.069569625	ns
APEX1	-0.05281537	-0.01695463	-4.04555913	5.2451895830	0.000336614	1.029001122	ns
LBP	-0.05085048	-0.06112739	-4.04305364	5.3017076020	0.000339495	1.022755303	ns
CLEC7A	-0.05076771	-0.12431534	-4.03394134	5.5111381181	0.000352134	0.981137639	ns
TMSB10	-0.06713251	-0.04443116	-4.03264417	5.5419055899	0.000353327	0.980384472	ns

ISM1	-0.03387188	-0.03915717	-4.02999486	5.6047155064	0.000356553	0.970033016	ns
ANG	-0.02968961	-0.02723474	-4.02313707	5.7705408606	0.000366304	0.94658204	ns
SEPTIN8	-0.02602056	-0.00304184	-4.01303861	6.0224127132	0.000381463	0.899361422	ns
CIT	-0.03793802	0.040308686	-4.01204619	6.0484565414	0.000382283	0.906821417	ns
AFM	-0.01626118	-0.01221922	-4.00814053	6.1485101510	0.000387242	0.879385523	ns
NADK	-0.047358	0.003413089	-4.00796448	6.1534381396	0.000387242	0.884149673	ns
THOP1	-0.02706646	-0.00935662	-4.00675513	6.1851327935	0.0003884	0.88135648	ns
FABP5	-0.0603297	-0.02514401	-4.00589	6.2073711725	0.00038896	0.871083662	ns
CST5	0.047538521	0.032074523	4.001362931	6.3279531403	0.000395666	0.863906421	ns
DNMBP	-0.05434035	0.06235885	-3.99157092	6.5943880495	0.000411445	0.819836863	ns
PEAR1	0.019372439	-0.00098816	3.988613574	6.6765565005	0.000415683	0.80119973	ns
CTSB	0.046861609	0.074758993	3.984113067	6.8045854835	0.000422753	0.789078005	ns
ENAH	-0.04033848	-0.00498068	-3.97992869	6.9258108084	0.000429371	0.779318773	ns
GBP1	-0.05231513	-0.01883414	-3.97282701	7.1346589478	0.000441381	0.740344585	ns
B4GALT1	-0.02244247	-0.01137044	-3.97112846	7.1857845590	0.000443604	0.735180296	ns
INPP5D	-0.04682001	0.010741509	-3.96418741	7.3986057265	0.000455779	0.717696453	ns
FGFR2	-0.01749726	-0.02253775	-3.95924851	7.5523098434	0.000464268	0.68788737	ns
TIMP1	-0.02289808	-0.01831708	-3.94013858	8.1801838314	0.00050181	0.624055185	ns
TIGIT	-0.02834348	0.007857633	-3.93584066	8.3273488763	0.000509766	0.601050547	ns
GRN	-0.02017272	-0.01926381	-3.93511853	8.3523849746	0.000510229	0.597797874	ns
DKK3	0.024811677	0.007080971	3.928185497	8.5959880388	0.000524014	0.56383532	ns
SRPX	0.027355575	-0.02342491	3.920332223	8.8807642768	0.000540246	0.533778061	ns
GZMH	-0.0611199	-0.03104039	-3.90897905	9.3092715920	0.000565137	0.501451443	ns
GAPDH	-0.02700474	-0.08122497	-3.90528337	9.4533119163	0.00057269	0.49722523	ns
SIGLEC6	-0.02705223	-0.02438559	-3.8946129	9.8767812304	0.000597106	0.432620298	ns
IL32	0.034394042	0.009635475	3.893437374	9.9252147334	0.000598794	0.433220581	ns
CELSR2	-0.02175769	-0.00448069	-3.88775694	0.000101606	0.000611734	0.419273298	ns
CCL23	-0.0342494	-0.02345672	-3.88006601	0.000104862	0.000630038	0.380459398	ns
FMNL1	-0.0533914	0.016024313	-3.87644216	0.000106444	0.00063823	0.376172147	ns
ERP44	-0.02178221	-0.00243641	-3.87539373	0.000106897	0.000639632	0.366489839	ns
CCL19	-0.09964738	0.236038158	-3.86743138	0.000110437	0.000659429	0.328522385	ns
CLUL1	0.038010928	0.035076086	3.866958147	0.000110658	0.000659429	0.333778241	ns
FABP2	0.065154601	0.057176035	3.855859953	0.000115795	0.000688215	0.290729729	ns
RASSF2	-0.04849528	0.032255231	-3.85553045	0.00011596	0.000688215	0.296173639	ns
LEPR	0.026569309	0.027827652	3.851472756	0.000117888	0.00069824	0.273953475	ns
TPR	-0.03827535	0.035559665	-3.8469128	0.000120109	0.000709959	0.264105647	ns
CILP	-0.04130552	-0.08372801	-3.84415751	0.000121451	0.000716438	0.240672181	ns
TCOF1	-0.02341262	-0.00607701	-3.84263044	0.000122208	0.000719453	0.23448365	ns
ANXA1	-0.03334116	-0.02239906	-3.84129573	0.000122889	0.000722001	0.243821052	ns
GPKOW	-0.02372651	-0.00350804	-3.83653406	0.000125292	0.000734642	0.223473202	ns
PSPN	-0.08138719	-0.13095437	-3.83148034	0.000127882	0.000748328	0.195755145	ns
C7	0.023557808	-0.02684234	3.830963589	0.000128159	0.000748447	0.201328663	ns
PTPRZ1	0.025274125	0.020720232	3.830019508	0.000128638	0.000749748	0.186472468	ns
GM2A	-0.02832316	-0.00674073	-3.82909985	0.000129132	0.000751129	0.19453025	ns
HAO1	-0.10661952	0.020620964	-3.82836919	0.000129502	0.00075178	0.1787661	ns
TNFRSF6B	-0.04532602	-0.0338869	-3.82408994	0.000131778	0.000763474	0.170430344	ns
PREB	-0.02410844	0.001283572	-3.81392938	0.000137319	0.000794001	0.137511402	ns
ST3GAL1	0.035882362	0.060155425	3.809364149	0.000139869	0.000807152	0.114163686	ns
PHLDB1	-0.04102904	-0.00647467	-3.805462	0.000142085	0.000818321	0.094643677	ns
LTBR	-0.02134446	-0.00391684	-3.80223635	0.000143955	0.000827457	0.089501631	ns
ENO3	-0.04586888	0.053892122	-3.8004682	0.000144991	0.000831775	0.087076822	ns
DTNB	-0.03299719	-0.00528024	-3.79289845	0.000149468	0.000855708	0.048240797	ns
NMNAT1	-0.06117048	0.037024758	-3.79244001	0.000149749	0.000855708	0.050828583	ns
LAMP2	-0.01569596	-0.01160369	-3.79188537	0.000150094	0.000856002	0.055150157	ns
SCRG1	-0.01938237	-0.02391007	-3.78000436	0.000157414	0.000896001	-0.00238938	ns
ERN1	-0.02838486	-0.00836406	-3.77331382	0.000161695	0.00091858	-0.02655952	ns
GCHFR	-0.03924298	0.002623943	-3.76612306	0.000166445	0.000943727	-0.03206081	ns
VEGFA	-0.0472149	0.029447754	-3.75346805	0.000175043	0.000990552	-0.10075711	ns
APOL1	-0.04535483	0.088976768	-3.751742	0.000176251	0.00099546	-0.10696171	ns
APOD	0.032737185	0.047112269	3.750472335	0.000177175	0.000998745	-0.09145493	ns
CPXM2	-0.01822866	-0.0228814	-3.74295204	0.000182528	0.001026942	-0.14070463	ns
MAMDC2	-0.02472086	-0.00412573	-3.74003851	0.000184655	0.00103691	-0.15152305	ns
NRP1	0.021887416	-0.00772109	3.739060684	0.000185379	0.001038974	-0.15219338	ns
ARSB	-0.04485404	-0.00677018	-3.73616408	0.000187528	0.001049007	-0.1598804	ns
CPQ	-0.02621204	-0.01981516	-3.72134489	0.000198867	0.001110308	-0.21963217	ns
SEZ6L	0.021863882	0.011829324	3.707289287	0.000210222	0.001171469	-0.27055365	ns

DNPEP	-0.04182836	0.009947578	-3.70519547	0.000211962	0.001178913	-0.28002242	ns
CPE	0.030965454	-0.03581652	3.704343468	0.000212679	0.001180651	-0.28162616	ns
CR1	-0.02663114	-0.00605057	-3.70092825	0.00021556	0.001194376	-0.29274257	ns
ITGB7	0.036393128	0.023779023	3.700425891	0.000215982	0.001194444	-0.29922353	ns
EFCAB14	-0.01917985	-0.00638845	-3.69571602	0.000220025	0.001214504	-0.31384287	ns
TNFRSF10C	-0.0393775	-0.09670437	-3.69447746	0.000221105	0.001216833	-0.31554896	ns
HBEGF	-0.05867727	-0.02610994	-3.69426813	0.00022128	0.001216833	-0.32055487	ns
PTPRF	-0.02249297	-0.01171792	-3.69079729	0.000224318	0.001231217	-0.33362559	ns
LGALS7_LGAL	-0.03240271	-0.00016966	-3.68970424	0.000225292	0.001234246	-0.33211346	ns
IGSF3	-0.02725222	0.002100542	-3.68783176	0.000226956	0.001241032	-0.33791635	ns
PENK	-0.02123555	-0.00879142	-3.67431682	0.000239309	0.001306135	-0.38146695	ns
FBP1	-0.05880151	-0.01463605	-3.66870368	0.00024461	0.001332579	-0.40799972	ns
SORBS1	-0.02917672	0.031570506	-3.66619956	0.000247003	0.001343108	-0.42360179	ns
LY96	-0.02825555	-0.01404267	-3.66442175	0.000248737	0.001350024	-0.42281615	ns
TCN1	-0.02468735	-0.00375252	-3.65853577	0.000254521	0.00137885	-0.44042064	ns
FCRL1	-0.03551156	-0.0024533	-3.65217659	0.000260894	0.001410761	-0.46803475	ns
SGSH	-0.04106598	-0.01126797	-3.64712882	0.00026607	0.00143609	-0.48508049	ns
SUOX	-0.04574175	0.065689172	-3.64518705	0.00026807	0.001444214	-0.49983974	ns
TNC	-0.03718386	0.00349746	-3.64184326	0.000271583	0.001460449	-0.50701699	ns
HEPACAM2	0.030490242	0.026025487	3.635155467	0.000278712	0.001496028	-0.53514383	ns
HDGFL2	-0.04707256	0.024071242	-3.63063449	0.000283636	0.001519665	-0.55144454	ns
BECN1	-0.04511568	0.047563299	-3.62459047	0.000290372	0.001552906	-0.56080191	ns
PAXX	-0.03303243	0.01019013	-3.61621494	0.000299913	0.001600999	-0.5916286	ns
SPINK6	-0.04299019	-0.11993858	-3.61548417	0.000300736	0.001602463	-0.60760419	ns
CCL5	-0.08057883	-0.11593381	-3.61250735	0.000304218	0.001618065	-0.61261643	ns
GALNT3	-0.03302852	0.001683694	-3.60723862	0.000310471	0.001648321	-0.62348072	ns
TIMD4	-0.03402189	-0.02280482	-3.60475037	0.000313442	0.001661074	-0.64039643	ns
CWC15	-0.04561571	0.103608748	-3.60305297	0.000315493	0.001668914	-0.6468097	ns
PSMD9	-0.0447079	0.050005513	-3.59985801	0.000319398	0.001686511	-0.65603532	ns
CNTN2	0.034838464	0.041309625	3.598080046	0.000321572	0.001694928	-0.66990741	ns
FGFBP3	-0.02629629	-0.01857842	-3.59646027	0.00032358	0.001699562	-0.67400714	ns
STC2	-0.01991534	-0.00342332	-3.5964386	0.000323615	0.001699562	-0.67078214	ns
MYBPC2	-0.05933512	0.18236127	-3.59522882	0.000325141	0.00170423	-0.66597673	ns
SEL1L	-0.02264805	0.00306191	-3.59480417	0.000325671	0.00170423	-0.66749156	ns
PCDH9	-0.0237775	-0.00992646	-3.5930641	0.000327827	0.001712442	-0.68512481	ns
ICAM4	0.041659817	-0.0441496	3.591309589	0.000330048	0.001720966	-0.68882132	ns
IL7R	0.041009691	0.016943953	3.584570771	0.000338679	0.001762819	-0.71307164	ns
DTYMK	-0.04991787	-0.01380721	-3.58396962	0.000339451	0.001763694	-0.71874342	ns
CTHRC1	-0.02001579	-0.02702126	-3.58247098	0.000341403	0.001770689	-0.72370347	ns
PAGR1	-0.03069028	0.030777056	-3.57797021	0.000347358	0.001798381	-0.72739936	ns
DEFB4A_DEFB	-0.11260147	-0.14645893	-3.57463976	0.0003518	0.001818152	-0.74267696	ns
EIF4EBP1	-0.0506674	0.012086571	-3.5735656	0.000353235	0.001820159	-0.74988652	ns
OLFM4	-0.08890508	-0.15120375	-3.57345813	0.000353435	0.001820159	-0.72719022	ns
VNN1	-0.05292093	-0.10123829	-3.57249783	0.000354666	0.001823283	-0.75842072	ns
PSMG3	-0.0401953	0.0163393	-3.56782199	0.000361058	0.001852882	-0.77226144	ns
GLRX	-0.04452555	0.00327093	-3.5570679	0.000376156	0.001926974	-0.80621628	ns
ICAM1	-0.02175685	-0.02943166	-3.54454579	0.000394471	0.002017261	-0.84952547	ns
FCRL3	-0.0412912	-0.02485141	-3.54135948	0.000399269	0.002036621	-0.85714085	ns
GIPC2	-0.02058079	-0.00497731	-3.54108645	0.000399652	0.002036621	-0.87014347	ns
PBXIP1	-0.0219069	0.009272955	-3.53938107	0.000402244	0.002046257	-0.87513343	ns
PEBP1	-0.04541587	0.00087832	-3.52484095	0.000424969	0.002158106	-0.92379271	ns
SH2D1A	-0.04231461	0.05250215	-3.52102927	0.000431122	0.002185551	-0.93686418	ns
FBLN2	-0.0207902	-0.00874705	-3.51438744	0.000442028	0.002236952	-0.96348496	ns
BCL2	-0.03735681	0.027505554	-3.50710921	0.000454288	0.002292169	-0.98798387	ns
SCPEP1	-0.03531485	-0.00077027	-3.5070016	0.000454509	0.002292169	-0.97529124	ns
CDCP1	-0.03668293	-0.07187559	-3.50408207	0.000459496	0.002313325	-0.99314373	ns
LYPD8	0.037425435	-0.00238495	3.501673287	0.000463687	0.002330404	-0.99543109	ns
CKAP4	-0.01872599	-0.00595406	-3.50113821	0.000464583	0.002330897	-1.0083134	ns
ADGRF5	-0.01976426	-0.02799286	-3.50058298	0.000465547	0.002331729	-1.01191377	ns
PSIP1	-0.0477577	0.035757826	-3.49913568	0.000468092	0.00234046	-1.01313427	ns
FGF23	-0.04092337	0.061514744	-3.49528484	0.00047488	0.00237034	-1.03123883	ns
HPSE	-0.0652656	-0.08430357	-3.48188942	0.000499282	0.00248789	-1.07006521	ns
SRP14	-0.04679556	-0.01340821	-3.46861295	0.000524592	0.002609555	-1.11637397	ns
MB	-0.038116	0.020739999	-3.45653621	0.000548631	0.002721336	-1.164533	ns
F11R	-0.04247144	0.017438556	-3.45639753	0.000548927	0.002721336	-1.16090831	ns
TGFB1	-0.0282705	-0.01279642	-3.4486094	0.000564978	0.002796162	-1.1915984	ns

MRC1	-0.01965351	-0.02580756	-3.44031634	0.000582567	0.002878335	-1.21843796	ns
NOS1	-0.03859198	-0.01122511	-3.43343266	0.000597593	0.002947588	-1.2304674	ns
NCAN	0.02871307	0.031964853	3.432663336	0.000599244	0.002950748	-1.24647573	ns
DUT	-0.07011851	-0.03891855	-3.42486608	0.0006167	0.003031587	-1.27091088	ns
TNR	0.031343718	0.04344359	3.415653169	0.000637949	0.003130773	-1.29678704	ns
CA9	0.031946855	0.034974485	3.413078866	0.000643974	0.00315504	-1.31362086	ns
SERPINE1	-0.0501797	-0.09002708	-3.4092047	0.000653184	0.003194801	-1.32581073	ns
AHSG	-0.01839753	-0.01146139	-3.40674703	0.000659092	0.003218309	-1.33250868	ns
GAST	-0.07980796	0.237719385	-3.40142723	0.000672042	0.003276066	-1.35132642	ns
FGF5	0.024243836	-0.00604983	3.398933572	0.000678239	0.003300765	-1.34760101	ns
CA2	-0.05254953	0.022368739	-3.39073786	0.000698811	0.003395219	-1.38202985	ns
TOMM20	-0.0521181	0.041646656	-3.38784262	0.0007062	0.003425424	-1.39732981	ns
EXTL1	0.032963871	0.074238247	3.3858834	0.000711311	0.003444492	-1.39025499	ns
C9	-0.03486955	-0.06124499	-3.383594	0.000717211	0.003467311	-1.41053342	ns
PSG1	0.083097762	-0.18310122	3.38104568	0.000723915	0.003493938	-1.41351107	ns
PRTG	0.017716401	0.009911725	3.378663815	0.000730182	0.003516869	-1.4296266	ns
HIP1R	-0.02886333	0.001337291	-3.37832944	0.000731075	0.003516869	-1.42809252	ns
IL19	-0.0492737	-0.00312237	-3.37552341	0.000738628	0.003547358	-1.42337971	ns
KIRREL2	0.02869673	0.000188261	3.372758321	0.000746065	0.003572701	-1.43537478	ns
HSPA2	-0.02283043	0.008452284	-3.37263538	0.000746352	0.003572701	-1.44742512	ns
PDZK1	-0.02959377	0.020532367	-3.36511551	0.00076703	0.003665675	-1.45950408	ns
CNDP1	-0.03278033	-0.0301518	-3.35910879	0.000783845	0.003739916	-1.48927252	ns
AK1	-0.04726566	0.007444219	-3.35453732	0.000796909	0.003792788	-1.50289395	ns
F11	-0.02059278	0.004171939	-3.35432632	0.000797525	0.003792788	-1.50146136	ns
ITGBL1	-0.02276738	0.002287456	-3.35346519	0.000799978	0.00379827	-1.51197266	ns
NFKBIE	-0.03604113	0.023023827	-3.35251864	0.000802773	0.003805352	-1.50269568	ns
TDP1	-0.04844213	0.089321489	-3.35181832	0.000804752	0.003808553	-1.51689808	ns
PLXDC2	0.017650138	0.012825755	3.344645769	0.000825831	0.003901986	-1.54143446	ns
SPON2	-0.0236817	-0.02850134	-3.34342919	0.000829494	0.003912958	-1.53730233	ns
PKD2	-0.04017666	0.05123548	-3.34017755	0.000839286	0.003947679	-1.54279397	ns
ASGR2	-0.01691049	-0.00895729	-3.34006808	0.000839558	0.003947679	-1.55577569	ns
VASN	-0.01960709	-0.01119955	-3.33621905	0.000851328	0.003996588	-1.55361086	ns
SNX15	-0.03697013	0.000995616	-3.33190867	0.00086459	0.004052331	-1.57177886	ns
NPTXR	0.024630006	0.046595402	3.32982694	0.000871053	0.00407608	-1.5848627	ns
NUDT5	-0.03721469	0.009041588	-3.32552219	0.000884597	0.004132839	-1.60138795	ns
HDGF	-0.06810899	0.043572348	-3.32166445	0.000896893	0.00418359	-1.61840576	ns
KCTD5	-0.0297955	-0.00222491	-3.31981852	0.000902881	0.004204804	-1.61448419	ns
AGXT	-0.05505075	0.063705728	-3.31311596	0.000924766	0.004299869	-1.63995298	ns
SERPINA7	-0.01234353	-0.01020756	-3.31004605	0.000934936	0.004340242	-1.65574657	ns
GPRC5C	-0.03630131	0.065085458	-3.30864046	0.000939644	0.004355177	-1.65962665	ns
C8B	-0.02022629	-0.01490046	-3.30719682	0.000944491	0.004370703	-1.66510409	ns
TP53I3	-0.03551928	-0.00374249	-3.30616401	0.000948078	0.004380361	-1.64541765	ns
PIGR	-0.0253518	-0.01968801	-3.30526495	0.000951072	0.004387252	-1.66129048	ns
ODAM	0.038206126	0.080863638	3.304536495	0.000953559	0.004391786	-1.6607774	ns
SIAE	-0.03333579	-0.00954337	-3.29576754	0.00098375	0.0045237	-1.69992043	ns
EDA2R	-0.02404703	-0.05887849	-3.29395794	0.000990084	0.004545669	-1.7101513	ns
LTBP2	0.021553595	-0.01365268	3.292709187	0.00099449	0.004558729	-1.71330049	ns
TOP2B	-0.05254841	0.014177289	-3.29087183	0.001001074	0.004581717	-1.70470663	ns
PDCD5	-0.04236062	-0.00980959	-3.28834872	0.001010013	0.004615395	-1.72786598	ns
COMP	-0.02393603	-0.03413648	-3.28473955	0.001023089	0.004667845	-1.72759559	ns
ADAM22	-0.02391192	-0.00237658	-3.28257511	0.001030946	0.004696354	-1.73913897	ns
BOLA2_BOLA2	-0.03698232	-0.02938422	-3.27679398	0.001052302	0.004786172	-1.75176444	ns
FUOM	-0.04235051	-0.00858004	-3.27598566	0.001055306	0.004791314	-1.75514793	ns
APOA1	0.01788097	0.031703827	3.275594391	0.001056714	0.004791314	-1.76835381	ns
IGDCC4	0.016999358	0.023081224	3.274637996	0.001060297	0.004800105	-1.77128196	ns
IL13RA1	-0.01579294	-0.00252625	-3.26659078	0.00109091	0.004927919	-1.79083099	ns
YTHDF3	-0.05914468	-0.01614724	-3.26633916	0.001091905	0.004927919	-1.78613892	ns
FEN1	-0.04546202	0.090077812	-3.26260917	0.001106377	0.004985528	-1.79782252	ns
PRG2	-0.03289322	0.009338928	-3.2577119	0.001125563	0.005064165	-1.82615864	ns
MEPE	-0.01748043	-0.0170716	-3.25180981	0.001149206	0.005155204	-1.83981343	ns
YOD1	-0.04210623	0.005495756	-3.25179152	0.001149328	0.005155204	-1.83190191	ns
NPTX1	0.027905571	0.025436673	3.249015732	0.001160539	0.005189616	-1.8510189	ns
CASC3	-0.02724592	0.029887722	-3.24902212	0.001160554	0.005189616	-1.84194423	ns
CDH6	0.025987023	0.002957018	3.248520668	0.001162569	0.005190673	-1.85029857	ns
AXL	0.01873051	0.001915937	3.246586968	0.001170483	0.00521803	-1.85815047	ns
CDH2	-0.02464843	-0.04225964	-3.24491328	0.001177386	0.005240805	-1.86192359	ns

SDC1	-0.02608821	-0.00568996	-3.24392765	0.001181465	0.005250956	-1.86509759	ns
IFNL2	-0.04043507	0.110522125	-3.2392924	0.001200862	0.005329052	-1.87389056	ns
COL9A2	-0.03009746	0.034086214	-3.23686124	0.001211099	0.005359341	-1.8866355	ns
SNCA	-0.07502249	-0.03125633	-3.23680519	0.001211358	0.005359341	-1.8817726	ns
FAS	-0.02273868	-0.00716048	-3.23549208	0.001216888	0.005375665	-1.89654845	ns
LILRA2	-0.02553453	-0.03608417	-3.23189577	0.00123231	0.005435566	-1.90608481	ns
TPK1	-0.01739984	-0.00937659	-3.23099436	0.00123618	0.005444413	-1.91257909	ns
SEZ6	0.020009954	0.016932741	3.228391219	0.001247542	0.005486179	-1.90825754	ns
CSF2RA	-0.04313459	-0.05182923	-3.22715823	0.0012529	0.005501455	-1.91816852	ns
APOF	0.016210769	-0.01808688	3.215724914	0.001303827	0.005716478	-1.9603535	ns
GIGYF2	-0.05033353	0.043087227	-3.21521378	0.001306142	0.005718041	-1.96379621	ns
RECK	0.013186528	0.005920219	3.21098879	0.001325564	0.005794383	-1.96361717	ns
ADAMTSL2	-0.02017268	-0.00973417	-3.20918277	0.001333841	0.005817049	-1.98192226	ns
PTPRB	-0.01875947	-0.0190152	-3.20898998	0.001334734	0.005817049	-1.98291328	ns
ACAA1	-0.05641963	0.00510804	-3.20605096	0.001348514	0.005868346	-1.97936945	ns
MSTN	-0.03572319	0.014857572	-3.20355204	0.001360259	0.00591065	-1.98793733	ns
GASK1A	-0.0281386	-0.01555889	-3.19307622	0.001410558	0.006120103	-2.01967808	ns
AIFM1	-0.07234578	-0.00148481	-3.18771175	0.001436924	0.006225251	-2.04334311	ns
HNRNPUL1	-0.03211663	0.024157675	-3.18414109	0.001454709	0.006285753	-2.06249153	ns
GLO1	-0.04045695	-0.01448294	-3.18406166	0.001455195	0.006285753	-2.04971761	ns
ATRAID	-0.01661102	-0.00162156	-3.18029759	0.001474142	0.00635819	-2.07264931	ns
TNFSF11	-0.04244516	0.033923726	-3.17092204	0.001522548	0.006542955	-2.09958359	ns
DPP4	0.019935645	-0.0035965	3.170924302	0.001522608	0.006542955	-2.08843208	ns
ARTN	-0.03156777	0.04621528	-3.1707109	0.001523702	0.006542955	-2.09160207	ns
RBP2	0.052352827	0.011937797	3.163973273	0.001559357	0.006686229	-2.11909581	ns
XCL1	-0.0379824	0.011526021	-3.16076752	0.001576565	0.006750101	-2.1368604	ns
BOLA1	-0.03454666	0.01293374	-3.15145071	0.001627795	0.006959241	-2.15246698	ns
ARHGAP1	0.03156044	-0.00398809	3.142380552	0.001679053	0.007167889	-2.1799525	ns
VEGFD	0.0245853	0.020503335	3.13723297	0.001708698	0.007283794	-2.209333	ns
MYH9	-0.07151341	-0.01766112	-3.12720938	0.001768011	0.007525644	-2.23972908	ns
SIGLEC10	-0.02222945	-0.01629258	-3.12415333	0.001786496	0.007593259	-2.24570536	ns
WARS	-0.02303439	-0.02033913	-3.12280457	0.001794692	0.007617008	-2.25014946	ns
ASAH2	-0.03664973	-0.07316103	-3.12111542	0.001805034	0.007649781	-2.25226438	ns
TPP1	-0.02994965	-0.01642557	-3.11759036	0.001826765	0.007730658	-2.25995768	ns
NCAM1	0.020546866	0.017233854	3.114106304	0.001848424	0.007810997	-2.27637248	ns
ICOSLG	0.01277627	0.003342288	3.112946919	0.001855708	0.007830445	-2.27805502	ns
LPCAT2	-0.02719368	0.042602369	-3.10375753	0.001914283	0.008062197	-2.30640786	ns
MFGE8	-0.02885214	-0.00135325	-3.10330859	0.001917172	0.008062197	-2.31039557	ns
MAP1LC3A	0.019464252	-0.01068705	3.103050068	0.001918913	0.008062197	-2.3026942	ns
PDIA3	-0.01605379	-0.00153876	-3.10199377	0.001925754	0.008079311	-2.30583641	ns
MDGA1	0.046842324	-0.03926383	3.10058622	0.001934895	0.008106017	-2.31565529	ns
MPRIP	-0.02756862	-0.02048133	-3.09113046	0.001997513	0.008356359	-2.35005392	ns
CXCL14	-0.04144545	0.046778147	-3.08886646	0.002012863	0.008408527	-2.34583758	ns
CD46	-0.01852331	-0.00838459	-3.08694959	0.002025865	0.008450751	-2.3544015	ns
FGF21	-0.08047926	-0.12870227	-3.08537497	0.002036537	0.008483151	-2.36982193	ns
MEGF10	0.02640584	0.010438591	3.078497779	0.002084147	0.0086691	-2.38431112	ns
TNFRSF13B	-0.0220082	-0.01794622	-3.077057	0.002094233	0.008698663	-2.39001085	ns
PTPRC	-0.01412377	-0.00194468	-3.07059414	0.002140034	0.008876277	-2.41389488	ns
WASL	-0.03528833	0.092964813	-3.06588168	0.002174142	0.009004956	-2.41441491	ns
CCER2	-0.03670146	0.035620043	-3.06356941	0.002190993	0.009054172	-2.42264073	ns
PLAU	0.018512292	-0.0023092	3.06339094	0.002192226	0.009054172	-2.43257267	ns
HS6ST1	-0.02161859	0.001433456	-3.06024862	0.002215308	0.009136582	-2.44725279	ns
KIF1C	-0.02194793	0.023050998	-3.04935865	0.002297294	0.009461354	-2.46488057	ns
CLGN	-0.0266069	0.023910683	-3.04714859	0.002314221	0.00951764	-2.47268842	ns
SORCS2	-0.02161634	-0.0212106	-3.04535455	0.002328082	0.009561182	-2.47788961	ns
NID1	-0.0249098	-0.02001742	-3.04490587	0.00233149	0.009561729	-2.48636279	ns
MMP7	-0.0290177	-0.07096548	-3.04370121	0.002340904	0.009586871	-2.48270133	ns
CCL25	0.032244852	-0.04666595	3.034591891	0.002412597	0.009866644	-2.52444387	ns
FCRL5	-0.0373347	-0.05477698	-3.02884918	0.002458947	0.010042132	-2.53702066	ns
CCL14	-0.02567598	-0.02449548	-3.02380142	0.002500359	0.010196993	-2.54773884	ns
CC2D1A	-0.04956046	0.043907301	-3.01701769	0.002556953	0.010413254	-2.56938946	ns
BAG4	-0.02972303	0.03790814	-3.01613111	0.002564503	0.010429455	-2.56457397	ns
ITGAM	0.020148439	0.011571648	3.00771496	0.002636458	0.010707171	-2.60034335	ns
THPO	-0.02460868	-5.06E-05	-3.00638702	0.002648079	0.010739432	-2.59554628	ns
SIGLEC7	-0.01816057	-0.03691398	-3.0045563	0.002663989	0.010788971	-2.60742488	ns
OSCAR	-0.02155681	-0.0420941	-3.00268535	0.002680396	0.010840385	-2.61443323	ns

SCAMP3	-0.05579063	0.02090481	-3.00180217	0.002688196	0.010856893	-2.61536283	ns
CRTAC1	0.01929531	-0.03935703	3.000753125	0.002697409	0.010879053	-2.62530332	ns
DTX3	-0.01481672	-0.02128341	-2.99845682	0.002717867	0.010946445	-2.62532561	ns
TIMP3	-0.08460939	0.089397347	-2.99443225	0.002753925	0.011076394	-2.64090639	ns
PTX3	0.032027627	-0.07781515	2.988096599	0.00281165	0.011293009	-2.65846363	ns
CCL17	-0.0583063	-0.08126845	-2.98714159	0.002820458	0.011312827	-2.65845465	ns
QDPR	-0.02790005	-0.01044036	-2.98633822	0.002827894	0.011327092	-2.65890033	ns
CA3	-0.03973054	0.016364658	-2.98524639	0.002837927	0.011351709	-2.67048616	ns
SLC16A1	-0.03710496	0.072578494	-2.97167921	0.002966437	0.011849516	-2.69811632	ns
ZBTB17	-0.02447624	0.004713698	-2.96962396	0.002986342	0.011912732	-2.70457754	ns
PLA2G10	0.03852643	0.023106515	2.958711354	0.003094021	0.012325432	-2.74232083	ns
DDAH1	-0.02816433	0.0037605	-2.95563815	0.003124988	0.012431832	-2.75186382	ns
RPE	-0.04314238	0.079024553	-2.94722378	0.00321126	0.012757659	-2.77558269	ns
CEBPB	-0.02347037	0.014006497	-2.93229993	0.003369678	0.013368833	-2.81390175	ns
SEMA4D	-0.01866769	-0.00860303	-2.92775223	0.003419183	0.013546829	-2.84046092	ns
RGMA	0.019353656	0.024678316	2.924163835	0.003458827	0.013685331	-2.85088477	ns
BCAN	0.021817118	0.042684075	2.923309689	0.003468403	0.013704649	-2.84567437	ns
ENPP5	0.027667556	-0.014431	2.921582302	0.003487636	0.013756352	-2.85483014	ns
MAPT	-0.03183928	-0.05906622	-2.92130024	0.00349091	0.013756352	-2.84545498	ns
PRKRA	-0.04277033	0.022198325	-2.91349859	0.003579296	0.014085638	-2.8678202	ns
MIA	-0.02251792	0.014763211	-2.91257334	0.003589776	0.01410787	-2.88150354	ns
IL18BP	-0.01821466	-0.01793789	-2.91179681	0.003598735	0.014124069	-2.88145929	ns
NCR1	-0.02211513	0.008840206	-2.90968912	0.003623081	0.014195643	-2.88799369	ns
GRPEL1	-0.03729423	0.019964552	-2.90937859	0.003626695	0.014195643	-2.88652766	ns
F13B	-0.01489156	-0.01699364	-2.90850021	0.003636862	0.014216383	-2.89170049	ns
KDR	-0.01600708	-0.01898273	-2.89773098	0.003764015	0.014693749	-2.92431055	ns
CFHR4	-0.04212976	-0.10948649	-2.89345523	0.003815607	0.014875265	-2.93758964	ns
VAMP5	-0.03326192	0.07240875	-2.89255432	0.003826702	0.01488769	-2.92880045	ns
RSPO3	-0.01634784	-0.01457522	-2.89235317	0.003828992	0.01488769	-2.94335442	ns
BNIP3L	-0.02568181	0.024587305	-2.88475241	0.003922781	0.015232076	-2.94931089	ns
PPL	-0.01853901	-0.00607081	-2.88359585	0.003937207	0.015267789	-2.95262184	ns
NRCAM	-0.01691349	0.002249471	-2.88186791	0.00395875	0.01533097	-2.96588039	ns
KLKB1	-0.01450279	0.002368563	-2.88070041	0.003973494	0.015367686	-2.9647996	ns
CA1	-0.03865558	0.002897596	-2.87829488	0.004004001	0.015465189	-2.96330513	ns
DKK4	-0.02802968	0.009476416	-2.87383823	0.004060775	0.015663754	-2.98662093	ns
RPL14	-0.02324771	0.018893004	-2.87150672	0.004090892	0.015759111	-2.98835411	ns
CHAC2	-0.03690768	0.03139876	-2.87061134	0.004102504	0.015783018	-2.99093341	ns
KHK	-0.03746072	-0.05240327	-2.86882133	0.004125765	0.015851624	-2.99600457	ns
MELTF	0.016530097	0.000390219	2.863975239	0.004189228	0.016074303	-3.02293556	ns
LILRA6	-0.04209563	-0.08383911	-2.86301775	0.004201901	0.016101773	-3.02565759	ns
NRN1	0.020464385	0.02868848	2.856598901	0.004287752	0.016409222	-3.04462285	ns
CD2AP	-0.03945365	0.014470425	-2.8527185	0.004340509	0.016589379	-3.04885111	ns
CTSF	0.024580261	-0.00066035	2.850717513	0.004367852	0.016672064	-3.05846075	ns
CEACAM16	-0.04567879	-0.02144254	-2.84795549	0.00440591	0.016795374	-3.06708473	ns
TAX1BP1	-0.03696325	0.078405696	-2.8429409	0.004475778	0.017039469	-3.08228914	ns
MYOC	0.02817338	-0.01576526	2.840922126	0.004504244	0.017125511	-3.08455041	ns
PGD	-0.03087204	-0.00138277	-2.83337868	0.004611831	0.017511762	-3.1094686	ns
SLITRK2	-0.02399575	-0.01402865	-2.82877433	0.004678654	0.017742428	-3.12392141	ns
PLA2G7	-0.0189504	-0.01671476	-2.82626397	0.004715521	0.017859042	-3.12578493	ns
VGF	0.016696671	0.015262499	2.819003395	0.004823399	0.018201303	-3.15054601	ns
NME3	-0.01400308	0.003821683	-2.81881824	0.004826247	0.018201303	-3.14572675	ns
GALNT2	0.015314364	0.01457175	2.818518064	0.004830694	0.018201303	-3.15267416	ns
TNFRSF19	-0.01708722	-0.0182122	-2.81850934	0.004830825	0.018201303	-3.15269858	ns
NSFL1C	-0.04085885	-0.01148979	-2.81771368	0.00484281	0.018203387	-3.15430081	ns
CDH3	-0.02041183	0.008297827	-2.81764495	0.004843847	0.018203387	-3.15449309	ns
DNPH1	-0.03190289	0.001263057	-2.8143843	0.004893279	0.018365519	-3.15948525	ns
F3	0.016247388	0.001808745	2.812983593	0.004914622	0.018421948	-3.16485731	ns
CLEC5A	-0.01698998	-0.01994965	-2.81045577	0.004953446	0.018543669	-3.1654369	ns
TREH	-0.04268683	-0.05782232	-2.81000372	0.004960308	0.01854558	-3.1745683	ns
AREG	-0.0265223	0.02018985	-2.80835481	0.004985842	0.018617211	-3.17323571	ns
GIMAP8	-0.02886944	0.021448399	-2.8071517	0.005004608	0.018663419	-3.16769828	ns
TSPAN1	-0.03333987	0.154218891	-2.79860767	0.005138866	0.019139654	-3.19415969	ns
HDDC2	-0.02461377	0.034345231	-2.7970638	0.005163306	0.019206181	-3.20969289	ns
CTRL	0.036879441	-0.00451214	2.79658183	0.005170994	0.019210309	-3.21207179	ns
CD300LG	0.021771577	0.019753201	2.796104619	0.005178686	0.019214439	-3.20995526	ns
FOLR2	-0.01737808	-0.01235875	-2.78664947	0.005332078	0.019758461	-3.24072514	ns

FGFR4	-0.02935342	0.040690262	-2.78220662	0.005405746	0.020006055	-3.23970434	ns
DYNLT1	-0.04450145	0.141053321	-2.780627	0.005432116	0.0200782	-3.24354476	ns
PSME1	-0.02272911	-0.00260616	-2.77381565	0.005547032	0.020477032	-3.26841103	ns
DNAJB2	-0.03092916	0.015920004	-2.77333883	0.005555218	0.02048136	-3.26415593	ns
ARMCX2	-0.03536308	0.119234436	-2.76769042	0.005652391	0.020813343	-3.27979971	ns
NRTN	-0.02951059	-0.00696535	-2.76622233	0.005677876	0.020880854	-3.28338662	ns
NFATC3	-0.03555484	0.109977462	-2.76358239	0.005723988	0.021023957	-3.29063212	ns
CD40	-0.02948253	0.023642807	-2.75917748	0.005801585	0.021282197	-3.30952247	ns
ECHS1	-0.05310065	-0.02536621	-2.75762255	0.005829347	0.021357205	-3.30652288	ns
AFP	0.040367928	0.046349366	2.756972687	0.005840877	0.021372631	-3.3129963	ns
PAM	-0.0164778	-0.0376591	-2.75470175	0.005881452	0.021494167	-3.32548083	ns
TNFRSF13C	-0.02486738	-0.02108308	-2.75204425	0.005929516	0.021642733	-3.32221823	ns
SCGB3A2	0.049410916	0.052983151	2.750302477	0.005960986	0.021730436	-3.336003	ns
KLF4	-0.02286791	0.003301661	-2.74652662	0.006030145	0.021955143	-3.33772198	ns
IGSF21	0.02187054	0.029836018	2.739932188	0.006152414	0.022372414	-3.35479207	ns
IL20RA	-0.02682643	0.052029597	-2.73788245	0.006190857	0.022484208	-3.36080624	ns
CHRDL2	-0.03196807	0.019447355	-2.734303	0.006258438	0.022701414	-3.37724393	ns
EEF1D	-0.05136413	0.01657469	-2.73034663	0.00633393	0.022946744	-3.39403577	ns
IGLC2	-0.0127979	0.004191152	-2.72824416	0.006374436	0.023064874	-3.39870574	ns
CHI3L1	-0.04289499	0.00532414	-2.7278225	0.006382857	0.023066761	-3.38263687	ns
DNAJB8	-0.03291537	0.109921576	-2.72237191	0.006488971	0.023421253	-3.40306078	ns
IDO1	-0.03475343	0.01788566	-2.72081462	0.006519596	0.023475049	-3.40660656	ns
HLA-E	-0.01599553	-0.00444427	-2.72050113	0.006525648	0.023475049	-3.41672065	ns
TOR1AIP1	-0.02831928	0.021777381	-2.72038886	0.006527993	0.023475049	-3.40775638	ns
NACC1	-0.03098756	0.03640718	-2.7167271	0.006600435	0.023706358	-3.42990595	ns
DAAM1	-0.04535584	0.022401802	-2.71500269	0.006635033	0.023801347	-3.42306514	ns
IL17RB	0.031755781	0.021288541	2.713600583	0.006663149	0.023872879	-3.4299142	ns
TGOLN2	-0.0135347	-0.01089464	-2.7118915	0.006697435	0.023963829	-3.44278832	ns
CAT	-0.03035055	0.057282782	-2.71152545	0.006704948	0.023963829	-3.43688362	ns
ISLR2	0.02193157	-0.00849424	2.707898697	0.006778741	0.02419795	-3.4392481	ns
HMBS	-0.02558848	-0.02120055	-2.7019019	0.006901902	0.024607515	-3.47226239	ns
NEFL	0.023904269	-0.03065688	2.699334663	0.006955603	0.024768733	-3.46297711	ns
TF	-0.01178659	-0.00701648	-2.69808584	0.006981499	0.024830665	-3.48077411	ns
ADAMTS16	-0.01830335	0.003784119	-2.6959146	0.007027362	0.024963379	-3.4742119	ns
CLEC11A	-0.02703912	-0.03148448	-2.69424958	0.007062413	0.025057408	-3.4863579	ns
LY9	-0.01667255	-0.01034892	-2.69270619	0.007095252	0.02514337	-3.48516097	ns
CSDE1	-0.06542914	0.236191659	-2.69157012	0.007119525	0.025198804	-3.48428189	ns
CRISP3	-0.01455482	0.008000267	-2.68379945	0.00728689	0.025759949	-3.51372043	ns
VIT	-0.01955979	-0.04245974	-2.68309809	0.007302106	0.025777407	-3.52101143	ns
HAGH	-0.04292535	0.031452897	-2.68276041	0.007309484	0.025777407	-3.52210056	ns
ADAMTS13	0.012987389	0.008123303	2.678995913	0.007392118	0.026037375	-3.53249302	ns
LRG1	-0.01357363	-0.00069428	-2.67711727	0.007433685	0.02615224	-3.53672717	ns
ESYT2	-0.04463462	0.031658512	-2.6736973	0.007509875	0.02638849	-3.54577422	ns
FARSA	-0.03758787	0.118898617	-2.67159186	0.007557331	0.026501186	-3.5386039	ns
BCL2L15	-0.03057455	0.052175926	-2.67139802	0.007561738	0.026501186	-3.53764756	ns
TFF3	-0.02550271	0.034613405	-2.67094696	0.007571777	0.026501186	-3.5460653	ns
CTSE	0.027779365	0.016807315	2.670662861	0.00757825	0.026501186	-3.54159059	ns
SSB	-0.03256004	0.078825803	-2.66921704	0.007610977	0.026583797	-3.54489928	ns
DLL1	-0.01580962	-0.01921755	-2.66880584	0.007620102	0.026583868	-3.55688427	ns
BTN2A1	-0.01380982	-0.01175012	-2.66447711	0.007718837	0.026896186	-3.5677544	ns
NIT2	-0.03998476	-0.04011081	-2.66152966	0.007786705	0.027100331	-3.57685826	ns
STX8	-0.02809765	0.031013476	-2.65904253	0.007844441	0.027268772	-3.58084113	ns
LETM1	-0.02810185	0.027133092	-2.65550518	0.007927058	0.027511097	-3.5948874	ns
ULBP2	-0.02003162	-0.02186822	-2.65525828	0.007932995	0.027511097	-3.58790744	ns
PNLIP	0.031993048	0.042523442	2.65325824	0.007980025	0.027641367	-3.59895235	ns
APOA4	0.018492171	0.017433054	2.651260946	0.008027343	0.027772326	-3.60512236	ns
DLK1	-0.03123534	-0.01779627	-2.65085402	0.008037008	0.027772854	-3.60695539	ns
CCL26	-0.05117225	0.034622279	-2.64772198	0.008111898	0.027975984	-3.61066318	ns
CD36	-0.02392902	0.029869116	-2.64760179	0.008114951	0.027975984	-3.60046893	ns
TP53BP1	-0.04751937	0.251193469	-2.64281003	0.008230637	0.028319058	-3.61305112	ns
WFIKKN1	-0.02301358	0.013343391	-2.64267316	0.008233863	0.028319058	-3.61934253	ns
ACYP1	-0.03554301	-0.00051881	-2.64134828	0.008266198	0.028396823	-3.6167782	ns
TIE1	0.023722835	-0.07811984	2.636139631	0.00839387	0.028801529	-3.64557632	ns
DNAJA4	-0.03863551	0.047081841	-2.63499494	0.008422223	0.028864897	-3.64777183	ns
PAFAH1B3	-0.0632796	0.44885687	-2.63268287	0.008479729	0.029027911	-3.65480294	ns
HSD17B14	-0.03234791	-0.01463328	-2.62615463	0.008644046	0.029555754	-3.67086147	ns

C1QTNF9	0.025794752	-0.03522758	2.621180926	0.008771341	0.029955924	-3.67083828	ns
BMPER	-0.01158615	-0.01125567	-2.61820663	0.008848122	0.030128323	-3.6792943	ns
BLVRB	-0.03848599	0.066930919	-2.61803284	0.0088525	0.030128323	-3.68853928	ns
IL22	-0.03444613	0.027189357	-2.6180205	0.008852774	0.030128323	-3.69131346	ns
NCLN	-0.03069107	0.102801357	-2.61642084	0.00889454	0.030235224	-3.68359618	ns
NENF	-0.05971424	0.381918551	-2.61548956	0.008918624	0.030281839	-3.69788913	ns
KLRF1	-0.02174615	0.007081496	-2.61344263	0.00897244	0.030429181	-3.6896665	ns
TIGAR	-0.02632173	-0.02390478	-2.60716891	0.009138448	0.030956227	-3.70617997	ns
ANXA3	-0.03944295	-0.01449547	-2.59451562	0.009481356	0.032080602	-3.75318613	ns
LAMA4	-0.01811753	-0.00547047	-2.5928526	0.009527345	0.032198896	-3.75468463	ns
SLITRK6	0.01901543	0.001927655	2.591449392	0.009566477	0.032293772	-3.74836614	ns
AGER	0.022659488	0.032440312	2.589632584	0.009616946	0.032426652	-3.75883552	ns
KYNU	-0.02531573	-0.01720816	-2.5847421	0.009754219	0.032851579	-3.77931942	ns
SRPK2	-0.04640435	0.120764505	-2.58372149	0.009783226	0.032911315	-3.77687074	ns
TACSTD2	0.017160627	0.012061232	2.581661447	0.009842855	0.033073805	-3.73001183	ns
IL31RA	0.020776984	0.021292701	2.578380627	0.009935925	0.033348161	-3.77818093	ns
NPY	-0.03629797	-0.09276635	-2.57706776	0.009973387	0.033414139	-3.79942282	ns
LHPP	-0.03357734	0.026361004	-2.57689472	0.009978469	0.033414139	-3.79456521	ns
LIFR	0.012934124	-0.0012579	2.573484867	0.010077306	0.033706453	-3.80307805	ns
TFPI2	-0.01990008	-0.00301056	-2.57205081	0.010119088	0.03380748	-3.80768796	ns
MSLN	-0.03082363	0.033705889	-2.56880788	0.010214317	0.034086636	-3.81152147	ns
IMPACT	-0.02944444	-0.00900124	-2.56767192	0.010247689	0.034138553	-3.82165699	ns
WASHC3	-0.03994557	0.072432157	-2.56722002	0.010261048	0.034138553	-3.82280898	ns
IL13RA2	0.014645104	-0.01318803	2.567096999	0.010264948	0.034138553	-3.81094781	ns
IL1RL2	-0.02156788	-0.01005191	-2.56588488	0.010300854	0.034218991	-3.81358762	ns
MOG	0.01796287	0.006724024	2.564068918	0.010354627	0.034358533	-3.83260098	ns
SIGLEC1	-0.02365252	-0.0364217	-2.56074121	0.01045435	0.034650058	-3.83683882	ns
IGF2BP3	-0.02440003	0.075782969	-2.55341484	0.01067702	0.035347956	-3.8439391	ns
CPPED1	-0.03748875	-0.01339715	-2.55294103	0.010691248	0.035354978	-3.86024006	ns
ITGB1	0.011780203	0.010320345	2.546591255	0.010887811	0.035964264	-3.86819373	ns
PLSCR3	-0.03075441	0.021866194	-2.54372377	0.010977418	0.036178895	-3.88166749	ns
GADD45GIP1	-0.0266386	0.036425935	-2.54372706	0.010977569	0.036178895	-3.86933932	ns
MAPRE3	-0.07585545	0.546414276	-2.5409967	0.011063666	0.03642154	-3.87417729	ns
MVK	-0.04251389	-0.01441882	-2.53441935	0.011273396	0.037070176	-3.89324544	ns
FLT4	-0.01878597	-0.05505408	-2.53315325	0.011313967	0.037161736	-3.90719677	ns
AMN	-0.02592873	-0.00356769	-2.53075328	0.011391852	0.037364169	-3.90246255	ns
MMP13	0.017122781	-0.00648146	2.530466507	0.01140119	0.037364169	-3.90299549	ns
MDH1	-0.02073251	0.01677717	-2.52869128	0.011458782	0.037510812	-3.91642497	ns
HGFAC	-0.01405583	-0.02443488	-2.52630577	0.011536911	0.037724278	-3.91997581	ns
PSAP	-0.01391133	-0.01470993	-2.52538199	0.011567257	0.037781198	-3.92388539	ns
CPB1	0.030970775	0.031093953	2.524791468	0.011586583	0.037802035	-3.93086993	ns
WFDC1	-0.02809498	0.056233955	-2.52206264	0.011677064	0.038054717	-3.92721856	ns
DCTN6	-0.03892638	0.086028779	-2.51972846	0.011754792	0.038265319	-3.92991287	ns
DNAJC9	-0.0393757	-0.0158368	-2.51792921	0.01181472	0.038386063	-3.94619415	ns
CD58	0.009540309	-0.00246532	2.517829346	0.011818175	0.038386063	-3.94118136	ns
C1QTNF5	-0.01907535	0.003171889	-2.51621205	0.01187263	0.038520089	-3.93922984	ns
TNFRSF12A	-0.01810162	-0.03569182	-2.51378693	0.01195435	0.038742178	-3.95571973	ns
GLOD4	-0.01960915	-0.01595977	-2.512706	0.011991115	0.038818244	-3.95395376	ns
MYOM1	-0.0234437	0.053938588	-2.51038499	0.012070343	0.039031452	-3.95387832	ns
TFPI	-0.01733148	-0.02593067	-2.50768177	0.012162926	0.039284387	-3.96478796	ns
PLA2G15	-0.01379091	-0.01449308	-2.5073133	0.012175469	0.039284387	-3.97185662	ns
CBLN4	-0.01663469	-0.00472011	-2.50543423	0.012240272	0.039449885	-3.98017626	ns
MTDH	-0.04613769	-0.00682864	-2.50286983	0.012329609	0.039693999	-3.97046196	ns
TRIAP1	-0.03435788	-0.0090717	-2.50194454	0.012361536	0.039752957	-3.98885178	ns
FUS	-0.02170565	0.009529481	-2.50150822	0.012377113	0.039759264	-3.97451839	ns
ALPP	-0.06032652	-0.01356746	-2.50076558	0.012402892	0.039793048	-3.98385633	ns
ADAM15	0.016338714	0.009517776	2.500427637	0.012414886	0.039793048	-3.97841598	ns
DMP1	0.030104921	0.010928345	2.49508147	0.012603431	0.040353091	-3.99100554	ns
TGFBR3	0.01688185	-0.00747867	2.49362298	0.012655012	0.040473861	-4.00852566	ns
PADI4	-0.03979386	0.078538967	-2.49276728	0.012685858	0.040528123	-3.99633159	ns
SLAMF8	-0.02803984	-0.09001592	-2.48901039	0.012820648	0.040913981	-4.006052	ns
CA12	0.0164855	0.010701771	2.487000402	0.012893101	0.04110028	-4.01793921	ns
HSPA1A	-0.03874904	0.024582347	-2.48516312	0.012959747	0.04124941	-4.02559226	ns
PSRC1	-0.03152187	0.098925761	-2.48493939	0.012968137	0.04124941	-4.01610294	ns
FYB1	-0.04996346	-0.03554146	-2.48241903	0.013059992	0.041496384	-4.03092717	ns
GCG	-0.06690327	0.020933821	-2.47159947	0.013462	0.042727217	-4.04892274	ns

SWAP70	-0.01789656	0.015111235	-2.46740119	0.013620528	0.043183432	-4.07125111	ns
SCRIB	-0.0456139	0.181398674	-2.46491901	0.013715484	0.043437325	-4.06526518	ns
CD164	-0.01469611	0.011745166	-2.46398313	0.013751124	0.043441954	-4.07718649	ns
DMD	-0.02579278	0.068997858	-2.46375049	0.013760258	0.043441954	-4.06812393	ns
GBA	-0.0269082	-0.02220117	-2.46371539	0.013761578	0.043441954	-4.06846872	ns
ICAM5	0.024290368	-0.04201857	2.463168038	0.013782457	0.04346088	-4.07678291	ns
PRND	-0.0237852	0.015342246	-2.46194233	0.013829493	0.043562156	-4.08566467	ns
IRAG2	-0.04699775	0.145537998	-2.46064627	0.013879838	0.043673627	-4.07637938	ns
DAPK2	-0.02657851	0.045879284	-2.45711243	0.014017096	0.044058041	-4.08311363	ns
ITGB6	0.016015107	-0.00077488	2.455898312	0.014064469	0.044159408	-4.0877454	ns
C9orf40	-0.04095197	0.072596166	-2.45524145	0.014089928	0.044191825	-4.10096837	ns
VWF	-0.03927823	-0.04195729	-2.45170117	0.014229216	0.044580805	-4.11145311	ns
GOT1	-0.01979897	0.016197126	-2.45081174	0.014264428	0.044643226	-4.11266221	ns
CCL3	-0.02891493	-0.02506131	-2.4488042	0.014344243	0.044835109	-4.11393596	ns
ATP6AP2	-0.02156494	0.028756029	-2.44850333	0.014356447	0.044835109	-4.10532816	ns
PLXNA4	-0.04323498	-0.03786337	-2.44653147	0.014434964	0.045032151	-4.11945927	ns
TMPRSS15	0.048149955	-0.00061091	2.445635124	0.014470915	0.045092848	-4.11922934	ns
TCL1A	-0.05256782	-0.04303184	-2.44527743	0.014485305	0.045092848	-4.11864659	ns
ADGRD1	-0.01845308	-0.01126659	-2.44454294	0.014514901	0.04513686	-4.11570751	ns
RCC1	-0.02476668	0.097805566	-2.44343158	0.014559441	0.045227198	-4.13001559	ns
CAPN3	0.018940404	0.041324709	2.441860509	0.014622927	0.045376139	-4.13382604	ns
THRAP3	-0.05618756	0.382559705	-2.4407263	0.014669222	0.045471474	-4.12328114	ns
SERPINI2	0.027228697	0.090164688	2.437301581	0.014808527	0.04585461	-4.14454384	ns
PSAPL1	-0.02393393	0.009602024	-2.43443977	0.014926098	0.046169711	-4.15146558	ns
PSMG4	-0.02873982	0.064253249	-2.43173604	0.015038251	0.046467401	-4.14469336	ns
IGDCC3	-0.02338988	0.07706132	-2.43031028	0.015097488	0.046601126	-4.15025725	ns
ERC2	-0.03601032	0.145471502	-2.42979471	0.015118943	0.04661807	-4.15086283	ns
SOD1	-0.02492175	0.008830319	-2.42839343	0.015177579	0.046749506	-4.15217813	ns
CBS	-0.02425676	0.053951592	-2.42556578	0.015296204	0.047065244	-4.16079561	ns
ASPN	0.012930714	0.007381007	2.423999961	0.01536198	0.047217876	-4.17794161	ns
RBP1	-0.034555	0.140594665	-2.4235229	0.015382445	0.047231063	-4.16597219	ns
PPCDC	-0.02517636	0.011964808	-2.42286408	0.015410063	0.047266159	-4.18157637	ns
DKK1	-0.03104368	-0.04478916	-2.4215306	0.015466696	0.047390086	-4.18478363	ns
SPON1	-0.01626899	-0.03630137	-2.42040189	0.015514887	0.047487914	-4.18192781	ns
NEDD9	-0.01636094	0.020931097	-2.41936659	0.015559349	0.047574136	-4.17447817	ns
EPHB6	-0.01531405	0.000193873	-2.41583349	0.015710925	0.047987344	-4.18940739	ns
ATP1B2	0.015099441	0.018758175	2.415269128	0.015735439	0.048011998	-4.18464237	ns
MCEMP1	-0.04387988	0.065267171	-2.41420421	0.015781487	0.048102237	-4.18686396	ns
APOBR	0.020626995	-0.06262169	2.409725183	0.015976441	0.048637655	-4.19881519	ns
TNF	-0.01949868	-0.00662835	-2.40940519	0.015990462	0.048637655	-4.20024955	ns
PPP1R12B	-0.03513857	0.110767768	-2.40847195	0.016031229	0.048710916	-4.20782783	ns
CCL27	-0.0309705	-0.07629135	-2.40610783	0.016135281	0.048952532	-4.21471209	ns
LACRT	0.024033788	0.134445009	2.405902247	0.016144277	0.048952532	-4.21952426	ns
AP2B1	-0.02368384	0.078026705	-2.40308871	0.016269216	0.049280199	-4.21492928	ns
F2	-0.00716911	-0.00502788	-2.39562851	0.016603688	0.050241212	-4.24562899	ns
IL6R	-0.01767753	-0.02117648	-2.3902724	0.016848006	0.050927721	-4.24861706	ns
CARHSP1	-0.02686608	0.032933344	-2.38971942	0.016873464	0.050928596	-4.24673509	ns
CHGA	-0.03701669	0.07489896	-2.38950081	0.016883178	0.050928596	-4.25999817	ns
IDI2	-0.02381564	0.025150723	-2.38777333	0.016963038	0.05109571	-4.25135021	ns
CRYBB1	-0.03133794	0.013665339	-2.38753781	0.016973575	0.05109571	-4.26504359	ns
TRIM58	-0.0416311	0.022676065	-2.38489498	0.017095984	0.051411197	-4.27033526	ns
CLSTN3	-0.01452464	0.002806415	-2.38396781	0.017139062	0.051487716	-4.2732618	ns
TNXB	0.012940191	0.005160074	2.383480438	0.017161866	0.051503237	-4.27091802	ns
TNFAIP8L2	-0.02995526	0.055873011	-2.38092366	0.017281578	0.051809249	-4.26809438	ns
FCGR2B	-0.02968901	0.025043393	-2.37980158	0.017334352	0.051872363	-4.26998357	ns
SCG3	0.017284611	0.010986714	2.379713857	0.017338224	0.051872363	-4.27888747	ns
CDC25A	0.019780318	0.022917025	2.379343373	0.017355924	0.051872363	-4.26897374	ns
VWA5A	-0.03738342	0.153254762	-2.37010993	0.017795352	0.053131317	-4.29074146	ns
EPCAM	0.050926854	0.117250887	2.369456221	0.017826579	0.053170185	-4.3020477	ns
SDC4	-0.02339707	-0.0308522	-2.36891538	0.017852674	0.053193682	-4.30287688	ns
DPEP1	0.025230998	-0.05640025	2.36741543	0.017925236	0.053355441	-4.30437657	ns
PPP1R14D	0.01773329	0.021593085	2.359884625	0.018292923	0.054394435	-4.33097825	ns
PTPRK	-0.01013522	0.011105959	-2.35854174	0.018359268	0.054519345	-4.33207117	ns
MYBPC1	-0.03252699	0.055926679	-2.35827842	0.018372272	0.054519345	-4.33398537	ns
TRAF3	-0.0177702	0.046941262	-2.35765841	0.018403344	0.054556107	-4.3208001	ns
DNAJA2	-0.04214274	-0.04836276	-2.35410728	0.018579686	0.055023005	-4.34262196	ns

CSPG4	0.015499759	0.004740762	2.353601043	0.018604984	0.0550421	-4.34453736	ns
TMEM25	0.020828833	0.030939852	2.352751743	0.018647481	0.05511199	-4.34766771	ns
NDUFA5	-0.01663852	-0.00320435	-2.35106276	0.01873274	0.055307988	-4.335102	ns
EREG	-0.0406899	0.055358132	-2.3496976	0.018801361	0.055454519	-4.34586647	ns
PLIN3	-0.02145399	0.011751979	-2.34923996	0.018824417	0.055466497	-4.34896246	ns
LILRB5	-0.03611814	-0.1654132	-2.34859384	0.018856933	0.055506294	-4.35718624	ns
MAP2K1	-0.03034266	0.02138386	-2.34783044	0.018895648	0.055564242	-4.35709934	ns
TANK	-0.03521663	0.03529172	-2.34638328	0.018969454	0.055667303	-4.34880515	ns
CDH5	0.014936782	0.02301617	2.346321166	0.018972279	0.055667303	-4.36248381	ns
SCGN	0.022066516	0.019422048	2.346021136	0.018987888	0.055667303	-4.34964869	ns
LRIG3	-0.01295335	0.005890878	-2.33853712	0.01937248	0.056710914	-4.3637313	ns
TGFBI	-0.0134822	-0.01174741	-2.33833684	0.019382703	0.056710914	-4.37052764	ns
PRAME	0.011993165	-0.00051939	2.335651569	0.019522497	0.057062754	-4.37122985	ns
AGRP	-0.02628925	-0.06768214	-2.33160913	0.019733983	0.057623232	-4.39718355	ns
CHGB	0.020064039	-0.00350925	2.330592583	0.019787662	0.05772225	-4.39738998	ns
MYL4	-0.02455893	0.067817221	-2.32288532	0.020198552	0.058862047	-4.40281992	ns
LRRC38	-0.02228928	0.009561434	-2.32230949	0.020229491	0.058888011	-4.40440818	ns
ESM1	0.017592427	0.024057836	2.321966189	0.020247795	0.058888011	-4.41234199	ns
ELN	-0.01552198	-0.04060549	-2.31888558	0.020414189	0.059312867	-4.42546214	ns
PTPRS	-0.01192827	0.002261013	-2.31732716	0.020498868	0.059456973	-4.42961888	ns
ADGRG1	-0.03081433	0.085784056	-2.31722685	0.020504511	0.059456973	-4.42284544	ns
GSR	-0.01038348	-0.00752614	-2.31449443	0.020653727	0.059830242	-4.4311711	ns
NAPRT	-0.01554736	0.036937627	-2.31349575	0.020708416	0.059929213	-4.43761892	ns
SLMAP	-0.03916235	0.053210249	-2.31105783	0.020842747	0.060258238	-4.44215645	ns
UHRF2	0.016250116	0.026746076	2.310630698	0.02086673	0.060267905	-4.43010785	ns
FDX1	-0.0291072	0.029802993	-2.30896619	0.020958951	0.060473842	-4.43392477	ns
RABGAP1L	-0.03005604	0.039207529	-2.3084613	0.020986928	0.060473842	-4.43642952	ns
SIL1	-0.01499578	0.036300461	-2.30769544	0.021029554	0.060473842	-4.43773532	ns
GFRA2	0.010178406	-0.0100504	2.307535376	0.021038273	0.060473842	-4.44509659	ns
MTUS1	-0.02553084	0.001213783	-2.30725791	0.021053758	0.060473842	-4.44477445	ns
SELENOP	0.010165203	0.003889723	2.307101282	0.021062294	0.060473842	-4.45232631	ns
KLRK1	-0.02079353	-0.05432116	-2.30000837	0.021460967	0.061557979	-4.46851278	ns
ZNRD2	-0.03131385	0.042293767	-2.29750623	0.021603198	0.06190514	-4.47316372	ns
PVR	-0.01754936	-0.01870719	-2.2936395	0.021824524	0.06247805	-4.48418348	ns
LRP2BP	-0.03851433	0.218250407	-2.29166573	0.021938625	0.062743178	-4.47459372	ns
AHCY	-0.03316372	-0.01930193	-2.28993648	0.022038538	0.062967252	-4.48554497	ns
NELL1	0.019753215	0.038081458	2.289558249	0.02206037	0.062968016	-4.49043618	ns
BMP4	0.022717041	0.039707954	2.285241785	0.022312324	0.063624988	-4.49437895	ns
NTRK2	-0.01243006	-0.00515125	-2.28259282	0.022468014	0.064006441	-4.50221542	ns
PDGFB	-0.04232999	-0.07750377	-2.2814937	0.022532821	0.064128497	-4.50813943	ns
CDHR1	-0.02078926	0.027509691	-2.28065947	0.022582503	0.06420731	-4.49957377	ns
TBC1D5	-0.03903286	0.097886446	-2.27997051	0.022623095	0.064260154	-4.51029529	ns
LEFTY2	0.025441892	0.002759612	2.278708914	0.022697915	0.064344671	-4.5179075	ns
NAGK	-0.02613953	0.008383735	-2.27812951	0.022732817	0.064344671	-4.5039689	ns
ABL1	-0.03416774	0.054459085	-2.27774508	0.022755503	0.064344671	-4.51291016	ns
CEACAM6	-0.01775854	-0.00209125	-2.27738438	0.022776856	0.064344671	-4.51977798	ns
IPCEF1	-0.04469282	-0.05433919	-2.2773688	0.022778001	0.064344671	-4.51221176	ns
GAS6	0.012190788	-0.00389406	2.27724816	0.022785065	0.064344671	-4.51730302	ns
CELA3A	0.027376976	0.035984228	2.272333961	0.023079998	0.065114584	-4.53111954	ns
TNIP1	-0.04125218	0.017423636	-2.27180672	0.023111829	0.065141449	-4.53252745	ns
FSHB	0.029975155	0.487550514	2.271293509	0.023143248	0.065167101	-4.52047709	ns
VWA1	-0.02083318	-0.01039964	-2.26641908	0.02343946	0.065937595	-4.54564174	ns
ERCC1	0.01467444	0.004867171	2.25626101	0.024068604	0.067642275	-4.55218828	ns
AHSP	-0.04218891	-0.00631884	-2.24777186	0.024604894	0.069082973	-4.58195116	ns
TXN	-0.01987998	0.030304596	-2.24197225	0.024977523	0.070061831	-4.59034596	ns
GAL	0.031674196	0.068500376	2.237215082	0.025286521	0.070860499	-4.60747671	ns
MFAP4	0.014017873	0.016450145	2.236809622	0.02531328	0.070867476	-4.59844011	ns
REG1A	0.022689204	0.030304079	2.236288711	0.025347342	0.070894865	-4.60225622	ns
MATN2	-0.01411772	-0.00991168	-2.23547064	0.025400782	0.070976349	-4.60938592	ns
CD7	-0.01903029	-0.00790243	-2.23357997	0.025525402	0.071256381	-4.60257864	ns
IGFBPL1	-0.01519843	-0.00875039	-2.22801727	0.025893835	0.072215853	-4.62442798	ns
AP3B1	-0.03466689	0.032140695	-2.22724514	0.025945232	0.072290151	-4.63100558	ns
ST13	-0.02547007	-0.0014959	-2.22579717	0.026042432	0.072491802	-4.62283601	ns
LRRC59	-0.02067076	0.06827152	-2.22530791	0.026074918	0.072513105	-4.63659142	ns
CHL1	0.014820659	0.032665862	2.223912298	0.026168837	0.072705046	-4.63518979	ns
ECHDC3	-0.03215049	0.055146059	-2.21644587	0.026675455	0.074042138	-4.65613547	ns

HDAC9	-0.02931574	0.087882006	-2.21082679	0.027062786	0.075045902	-4.65591673	ns
EIF2S2	-0.01878306	0.058394612	-2.20476878	0.027485367	0.07614542	-4.66829149	ns
IL2RA	-0.01869729	-0.01663132	-2.2016917	0.027702062	0.076636182	-4.67891133	ns
FAM13A	-0.03021112	0.053318985	-2.20150509	0.027715003	0.076636182	-4.68760465	ns
FOLH1	-0.04861582	0.318674446	-2.20022775	0.027805895	0.076786148	-4.67698594	ns
CLEC1A	-0.01490387	-0.0125372	-2.2000027	0.02782183	0.076786148	-4.67938633	ns
EIF4G1	-0.04784458	-0.09664997	-2.19774978	0.027981648	0.077154307	-4.69821566	ns
CEP164	-0.02930627	0.098847399	-2.19729485	0.028014522	0.07717208	-4.68507465	ns
TNFSF12	-0.01343441	0.020499813	-2.195847	0.02811763	0.07738311	-4.7023666	ns
NTF4	-0.01884039	0.050154806	-2.19419799	0.028236417	0.077588201	-4.69140168	ns
GGT5	0.01007258	-0.00087408	2.194071341	0.028245294	0.077588201	-4.69813396	ns
CD55	-0.01214512	-0.00149866	-2.19330467	0.028301033	0.077668249	-4.68189466	ns
TNFAIP2	-0.02091924	0.023714261	-2.19285144	0.028332927	0.077682767	-4.70648523	ns
CDH23	0.011581408	0.015483431	2.186826815	0.028770064	0.078807304	-4.7206503	ns
CD109	0.015637985	-0.00691789	2.183132438	0.029041134	0.079475269	-4.72514519	ns
CFC1	-0.02165473	0.017080982	-2.18105495	0.029194787	0.079804145	-4.71994753	ns
PF4	-0.04229158	-0.05642136	-2.18076425	0.029215969	0.079804145	-4.72983599	ns
CRELD2	-0.0243825	-0.02925098	-2.17975999	0.029290369	0.079932596	-4.73239557	ns
STIP1	-0.03137601	0.019212221	-2.17923869	0.02932911	0.079963587	-4.73256244	ns
PQBP1	-0.02715155	0.044443246	-2.17810821	0.02941346	0.080067135	-4.72632414	ns
NAGPA	-0.00930101	-0.00723809	-2.17792463	0.029427024	0.080067135	-4.72748955	ns
AMIGO2	0.010447693	-0.01109965	2.177591645	0.029451679	0.080067135	-4.73443909	ns
LRRFIP1	-0.02169051	0.057675848	-2.17725309	0.029476771	0.080067135	-4.74065819	ns
GMPR2	-0.02937853	0.02200311	-2.1759241	0.029576342	0.080262935	-4.73181339	ns
PSME2	-0.01756309	-0.00070037	-2.17537828	0.029617129	0.080298995	-4.73686513	ns
GALNT5	0.011661392	-0.00234879	2.174134188	0.029710149	0.08047647	-4.74837378	ns
TXLNA	-0.04204385	-0.04624047	-2.17206677	0.029865962	0.080823549	-4.74583157	ns
EFHD1	-0.01464918	-0.0017446	-2.17035879	0.029994786	0.081097015	-4.75727794	ns
KIF22	-0.05323349	-0.16132158	-2.16951703	0.030059069	0.081195635	-4.74393644	ns
ERBB2	-0.01137897	-0.025448	-2.16824723	0.030155106	0.08137977	-4.75892911	ns
TRAF3IP2	0.020447981	0.067928226	2.16696874	0.030252891	0.081568276	-4.74942208	ns
DDI2	-0.03431763	0.046574761	-2.16534191	0.030377072	0.081827536	-4.75461908	ns
TNFSF13B	-0.01228064	-0.01005825	-2.16268653	0.030580559	0.082299753	-4.77266157	ns
VTI1A	-0.02566001	0.054787506	-2.15880706	0.030880795	0.083031235	-4.76864687	ns
CALCOCO2	-0.03345428	0.032612566	-2.15701121	0.031020543	0.083330254	-4.77169867	ns
JAM3	-0.02137373	0.039494943	-2.1560064	0.031098863	0.083463859	-4.77464582	ns
CYB5R2	-0.0238802	0.014019797	-2.15385792	0.031266894	0.08383777	-4.7845543	ns
PAEP	-0.06304187	0.603761557	-2.15261361	0.031364676	0.0840228	-4.78759854	ns
IL25	-0.01481871	-0.01733542	-2.15190264	0.031420969	0.084096453	-4.77846821	ns
SEPTIN7	-0.02309218	0.063744073	-2.14812527	0.031719491	0.084817686	-4.79675783	ns
OPTC	0.021031147	0.035810386	2.14553659	0.03192566	0.085290876	-4.80323888	ns
RNASEH2A	-0.02150727	0.083288805	-2.14317531	0.03211452	0.085717001	-4.81549858	ns
POLR2A	0.012772949	0.012630235	2.14233259	0.03218274	0.08582064	-4.80178925	ns
DUSP13	0.017830703	0.043209695	2.140903548	0.032297419	0.086047868	-4.81859181	ns
SELPLG	-0.01232433	-0.01906227	-2.14033084	0.032343757	0.086092772	-4.81674712	ns
VIM	-0.05310233	-0.15861497	-2.13904105	0.032448371	0.086292571	-4.81069733	ns
ATXN3	-0.02732335	0.011150893	-2.13857822	0.032485428	0.08631251	-4.82427024	ns
SCN4B	-0.0175533	-0.01807436	-2.13795614	0.032536333	0.086369174	-4.81232612	ns
TNFRSF8	-0.02021392	0.016251564	-2.13661495	0.032644928	0.086538111	-4.82958836	ns
CLPP	-0.04400181	-0.01695133	-2.13614664	0.032683355	0.086538111	-4.82143846	ns
CHMP6	-0.02422648	-0.00209713	-2.13607562	0.032688883	0.086538111	-4.82982264	ns
DBI	-0.03688828	-0.00851293	-2.1283477	0.033324217	0.088140139	-4.83972695	ns
ADAMTSL5	0.016582779	0.005379238	2.125981122	0.033520605	0.088579336	-4.8511846	ns
GOLGA3	-0.03309629	0.05655925	-2.12306954	0.033763804	0.089141326	-4.85808864	ns
AMOTL2	-0.02094459	0.101791668	-2.12257364	0.033805518	0.089170832	-4.85524573	ns
SERPINF2	-0.00582443	0.00282687	-2.11925791	0.034084863	0.089826535	-4.85526168	ns
CD80	-0.0137421	-0.01691122	-2.11851119	0.034147676	0.089910923	-4.86692735	ns
NECTIN4	-0.01267525	-0.00957149	-2.11420673	0.034513605	0.090647711	-4.87383295	ns
HCLS1	-0.0256982	-0.04580436	-2.11395745	0.034534797	0.090647711	-4.87760414	ns
PRUNE2	-0.01248221	0.013648507	-2.11386683	0.034542816	0.090647711	-4.86924652	ns
IL17C	-0.03135598	0.029765727	-2.11376513	0.034551679	0.090647711	-4.86383702	ns
PGA4	-0.02681424	0.005538634	-2.10578201	0.035239354	0.092368864	-4.89249576	ns
MYO9B	-0.03034751	0.098124964	-2.10422923	0.035374937	0.092575336	-4.88380455	ns
ENDOU	-0.02199616	0.032050028	-2.10416318	0.035381533	0.092575336	-4.86232136	ns
PRDX3	-0.03224025	0.01579031	-2.10345638	0.035442386	0.092651538	-4.8854189	ns
CD5L	-0.01947517	0.013826106	-2.10257508	0.035519131	0.092769108	-4.89656573	ns

ALDH5A1	-0.01959954	0.044616667	-2.10203399	0.035566366	0.092809464	-4.90221877	ns
MKI67	-0.01648383	0.045300991	-2.09538404	0.036153551	0.094257471	-4.90055324	ns
NINJ1	-0.02581548	0.09235126	-2.09312186	0.036354985	0.094698087	-4.90652319	ns
PNMA1	-0.02339153	0.061157013	-2.088066	0.036808619	0.095794268	-4.91699071	ns
CLEC4C	-0.02087543	-0.00674288	-2.08505987	0.037080354	0.096346309	-4.93269895	ns
AKR1C4	-0.02383491	0.040982254	-2.084993	0.03708673	0.096346309	-4.92403257	ns
EPS8L2	-0.01321362	-0.01011755	-2.08406619	0.037170496	0.096465109	-4.93929915	ns
PTH1R	-0.0171814	0.036501877	-2.08371068	0.037202999	0.096465109	-4.9354915	ns
CD82	0.017620427	0.063498084	2.083091509	0.037259482	0.096465109	-4.93337209	ns
SYAP1	-0.02366084	0.062045785	-2.08292094	0.037275165	0.096465109	-4.92844176	ns
MUC2	0.03856505	0.023005902	2.082670511	0.03729764	0.096465109	-4.94129707	ns
FKBP4	-0.01993782	-0.02131707	-2.08165496	0.037390612	0.096619988	-4.93676738	ns
TDO2	-0.01594961	0.031305279	-2.07961987	0.037577226	0.097016357	-4.93472822	ns
PTN	-0.02161631	-0.04440961	-2.07886256	0.037646863	0.097110283	-4.93670466	ns
SLC51B	0.031809256	0.179493295	2.077967708	0.037729262	0.097236934	-4.93697003	ns
INSL5	-0.03993972	0.07745515	-2.07712313	0.037806665	0.097332852	-4.95183932	ns
CEP20	-0.03297659	0.010310323	-2.07684126	0.037833146	0.097332852	-4.94022548	ns
VSTM1	-0.02199203	-0.00777882	-2.07490412	0.038012352	0.097707806	-4.94445283	ns
FOLR1	0.011971088	0.006530722	2.074536511	0.038046102	0.097708548	-4.95411133	ns
CREB3	0.022870826	0.072483621	2.070788579	0.038395596	0.098519455	-4.95057473	ns
ACP5	-0.01456433	-0.05512518	-2.06903045	0.038559863	0.098854083	-4.96701585	ns
ARHGEF1	-0.03764837	-0.03548987	-2.06820909	0.038636914	0.098964726	-4.97125478	ns
ADAMTSL4	0.009735342	0.001425792	2.067384733	0.038714938	0.099077667	-4.95965385	ns
RTBDN	0.012793152	0.002371294	2.064304248	0.039005697	0.099688545	-4.9730218	ns
ASAH1	-0.01945206	-0.00626588	-2.06413275	0.03902192	0.099688545	-4.97450996	ns
MMP12	-0.022709	-0.05599083	-2.06272924	0.039155107	0.099941356	-4.98004162	ns
SHD	-0.03544859	0.212118268	-2.06221093	0.039204838	0.099980897	-4.96834874	ns
CASP1	-0.03025213	-0.00098782	-2.05873031	0.039537231	0.10074059	-4.97764265	ns
RARRES1	-0.01478777	0.017547976	-2.05767481	0.039638555	0.100884386	-4.97956464	ns
NTF3	0.015612826	0.011981507	2.057419343	0.039662766	0.100884386	-4.98954828	ns
CNP	-0.03327686	-0.0023474	-2.05534039	0.039863357	0.101306356	-4.98322658	ns
ERMAP	-0.01849157	-0.01702093	-2.04992238	0.040389312	0.102509743	-4.99410501	ns
SDK2	-0.01562735	0.019304664	-2.04954771	0.040425774	0.102509743	-4.99690731	ns
EPHA2	-0.01236873	-0.02213227	-2.04938239	0.0404422	0.102509743	-4.99023205	ns
SPAG1	-0.02383138	0.053027227	-2.04326747	0.041042816	0.103843441	-5.02317685	ns
ENPEP	-0.0179439	0.057241602	-2.04256392	0.041113034	0.103843441	-5.00938972	ns
CACNB1	-0.02410078	0.082709257	-2.0424835	0.041120951	0.103843441	-5.01136789	ns
ATG4A	-0.02661664	0.029637334	-2.04234098	0.04113512	0.103843441	-5.01077487	ns
ALPI	0.050840226	0.218777755	2.042229455	0.041146185	0.103843441	-5.00973277	ns
PRDX6	0.026562414	0.071347133	2.039705352	0.041396579	0.104385157	-5.03118898	ns
ENPP7	-0.03828229	-0.08705465	-2.03840768	0.041526873	0.104623357	-5.01076283	ns
DPP7	-0.02589735	-0.0288278	-2.03639976	0.041727495	0.105038176	-5.02770435	ns
PDIA4	-0.02483905	0.038371811	-2.03476882	0.041891141	0.105359288	-5.03922831	ns
DSC2	-0.0140454	-0.01553323	-2.03400235	0.041968708	0.105463534	-5.03103972	ns
ATOX1	-0.03163951	-0.06073943	-2.03336206	0.042033028	0.105534345	-5.04031807	ns
STX7	-0.02117845	-0.01084607	-2.03230634	0.042140193	0.105712512	-5.0298043	ns
EIF1AX	-0.02224741	0.097428929	-2.03006194	0.042367287	0.106190967	-5.04888094	ns
HMMR	-0.01972974	0.057454686	-2.0265692	0.042724159	0.106923773	-5.03910787	ns
SULT2A1	-0.02362257	0.05863813	-2.02647991	0.042732892	0.106923773	-5.05228322	ns
EIF4B	-0.02487374	-0.04025364	-2.02317037	0.043072973	0.107682433	-5.05595695	ns
CENPF	-0.02314946	0.062890825	-2.0195105	0.043451625	0.108536137	-5.06287704	ns
ADA2	-0.01777212	-0.01800107	-2.01782803	0.043626747	0.108880428	-5.06353179	ns
COL1A1	0.010994516	0.012764636	2.016817198	0.043731791	0.109049384	-5.07631154	ns
UBE2L6	-0.0251753	0.006833735	-2.01576463	0.043841829	0.109230496	-5.07784419	ns
DNM3	-0.02208364	0.052783543	-2.01333404	0.044096727	0.109759161	-5.08347067	ns
SPART	-0.03182606	0.012599756	-2.01302941	0.044129197	0.109759161	-5.07055866	ns
MAP2	-0.01837057	0.062906109	-2.00952572	0.044498709	0.110584027	-5.0900723	ns
CLNS1A	-0.01389384	0.035504115	-2.00533453	0.04494509	0.111598352	-5.08537296	ns
CPA4	-0.01743519	-0.00519132	-2.00453765	0.045030655	0.111715813	-5.07650062	ns
AAMDC	-0.02142235	0.012003783	-2.00275447	0.045221518	0.111920957	-5.0902432	ns
CHIT1	-0.06576327	-0.33566846	-2.00261048	0.045236651	0.111920957	-5.0998765	ns
GHRHR	-0.02374636	0.085430385	-2.00251686	0.045247056	0.111920957	-5.09097774	ns
NAMPT	-0.02306389	0.040595742	-2.00233473	0.045266661	0.111920957	-5.09110448	ns
CD63	-0.02244995	-0.05073654	-2.00104053	0.045405722	0.112169805	-5.09914991	ns
AMDHD2	0.010279969	-0.03569156	1.999384272	0.045584236	0.112467554	-5.11010672	ns
SLC9A3R2	-0.02029698	0.062944497	-1.99920836	0.045603282	0.112467554	-5.10953838	ns

VTCN1	0.012979885	0.000449847	1.995886921	0.045964231	0.113262072	-5.10390258	ns
STK11	-0.03101574	-0.00298081	-1.99078045	0.046523063	0.114542448	-5.11465991	ns
PKD1	0.010224121	-0.01444359	1.986656257	0.046978094	0.115565319	-5.1356928	ns
CLEC1B	-0.04128448	-0.0024357	-1.98488043	0.047175367	0.115952921	-5.14010869	ns
ELAVL4	-0.03372261	0.071513876	-1.98401487	0.047272285	0.116093416	-5.12824281	ns
SART1	-0.01509225	0.02881335	-1.98311973	0.047372225	0.116241091	-5.12818751	ns
BCR	-0.03904281	-0.03220914	-1.98116483	0.047590615	0.116676322	-5.14245245	ns
LGMN	-0.0153117	-0.01921954	-1.98081729	0.047629512	0.116676322	-5.14444206	ns
SPACA5_SPAC	0.011492749	0.002076908	1.978100222	0.047935055	0.11732637	-5.15330135	ns
RILP	-0.02608108	-0.00541491	-1.97696751	0.04806302	0.117541054	-5.15495494	ns
PARK7	-0.02275056	-0.00786113	-1.97374992	0.048428067	0.11830551	-5.15728814	ns
STXBP1	-0.03070703	-0.04055021	-1.97349729	0.04845664	0.11830551	-5.16233301	ns
B3GAT3	0.008074502	0.005472928	1.972247321	0.048599681	0.118555612	-5.14953644	ns
LRRC37A2	0.016910164	0.009153847	1.967477984	0.049145978	0.119788193	-5.17203395	ns
TBC1D17	-0.01881578	0.041676135	-1.96630149	0.049282123	0.119960469	-5.16233273	ns
AIF1	-0.02210947	-0.01505848	-1.96615714	0.049298823	0.119960469	-5.16237889	ns
DNAJC6	-0.03336752	0.033372294	-1.96566866	0.049355221	0.119997706	-5.16330568	ns
DTX2	0.02558438	0.139973222	1.962585682	0.049712651	0.120766173	-5.16958182	ns
MAN1A2	-0.01016082	-0.00399554	-1.9617272	0.049812623	0.120908446	-5.17099048	ns
SATB1	-0.01196061	0.036292245	-1.95456582	0.050652264	0.122796458	-5.1992583	ns
NFYA	-0.01543037	0.041567128	-1.95438204	0.050674566	0.122796458	-5.18436174	ns
LONP1	-0.03376681	0.009066422	-1.95038801	0.051148393	0.12384188	-5.19301293	ns
FLT1	-0.00893085	-0.00074714	-1.9497648	0.051222443	0.123918421	-5.20027682	ns
MECR	-0.03551954	-0.01244222	-1.947804	0.051456952	0.124382699	-5.19801361	ns
IL31	0.02177373	0.107510903	1.946528433	0.051609896	0.124649212	-5.19923468	ns
SOD2	-0.01526791	0.000823853	-1.94572277	0.051706255	0.124778732	-5.21183125	ns
CCN1	-0.03225635	-0.1504574	-1.94485228	0.05181109	0.124928475	-5.21016302	ns
HEXIM1	-0.03457607	-0.00232697	-1.94173433	0.05218755	0.125683251	-5.21925412	ns
GAMT	0.014270589	0.048941785	1.941550512	0.0522102	0.125683251	-5.20917916	ns
SDHB	-0.02648652	0.110010598	-1.94110263	0.052263976	0.125709068	-5.22322177	ns
PTRHD1	-0.02356263	-0.00351064	-1.93996606	0.052402355	0.12593817	-5.21394265	ns
FCRL6	-0.02830725	-0.06208882	-1.93540018	0.052959557	0.12717262	-5.23144773	ns
PCDH1	-0.0084456	0.012253928	-1.93283155	0.053275171	0.127825391	-5.24023955	ns
SPRR3	-0.02567319	0.035261931	-1.93065103	0.05354444	0.128337708	-5.24438712	ns
CRTAM	-0.02084687	-0.01534863	-1.93039347	0.053576598	0.128337708	-5.2380693	ns
FES	0.01968951	0.018532069	1.927053999	0.053991713	0.129226066	-5.23795637	ns
FGF9	0.01545588	-0.02044946	1.923732545	0.054407036	0.130113467	-5.24068001	ns
CLEC4A	0.014469253	0.005587358	1.922954645	0.054504232	0.130239246	-5.25529017	ns
THSD1	0.013057738	0.00957023	1.922458368	0.054567157	0.130282992	-5.24146503	ns
ACTA2	-0.01753906	0.001956434	-1.92197965	0.054627156	0.130319686	-5.24830486	ns
RBM25	-0.01803086	0.017726948	-1.92054103	0.054808503	0.130611805	-5.24827464	ns
CTRB1	0.022174696	0.01138384	1.920295522	0.054839066	0.130611805	-5.26064019	ns
IL7	-0.02672082	0.010567027	-1.91917833	0.054980296	0.130822807	-5.26249422	ns
LEG1	0.026101686	0.056077238	1.918860485	0.055021395	0.130822807	-5.23818457	ns
PROCR	0.013609145	0.035921165	1.918535489	0.055062065	0.130822807	-5.25328308	ns
IL1A	-0.02329673	0.074834604	-1.91786927	0.055146422	0.13091671	-5.25591642	ns
ZBP1	-0.01538445	0.042093612	-1.91668988	0.055296277	0.131120034	-5.25646072	ns
SIRT1	-0.03433306	0.197516286	-1.91648854	0.055321877	0.131120034	-5.25684385	ns
GSTM4	0.027873243	0.149705727	1.915805754	0.055408773	0.131219479	-5.25847923	ns
SBSN	-0.01292133	0.002892931	-1.91026867	0.05611712	0.132789296	-5.28196204	ns
SIRPA	-0.02353454	-0.09106742	-1.90934577	0.056236142	0.132963187	-5.28024751	ns
SYTL4	-0.02769015	0.093125142	-1.90464081	0.056845284	0.134294684	-5.29416328	ns
CXCL11	-0.03175989	0.023056925	-1.90237497	0.057140658	0.134843354	-5.29936255	ns
C1QTNF1	-0.01729565	-0.00940625	-1.90215272	0.057169888	0.134843354	-5.29433886	ns
TXNRD1	-0.01935201	0.014519003	-1.89961271	0.057502665	0.135518791	-5.29986865	ns
PTGES2	-0.02061965	0.00635611	-1.89923319	0.057553119	0.135528312	-5.28578962	ns
CTRC	0.025290901	0.04297903	1.894668279	0.058155169	0.136832617	-5.30713467	ns
L3HYPDH	-0.03384927	0.011406265	-1.89432329	0.058200723	0.136832617	-5.31362602	ns
MICB_MICA	-0.06191859	-0.52832135	-1.88979462	0.058804265	0.138041756	-5.3180657	ns
IFT20	-0.01706568	0.054336889	-1.88975711	0.05880957	0.138041756	-5.3088666	ns
KIAA0319	0.011457295	0.017264363	1.888780617	0.058939884	0.138225939	-5.32379559	ns
PPBP	-0.03197706	-0.07303779	-1.88846245	0.058982713	0.138225939	-5.31979923	ns
ALDH3A1	-0.02568666	0.035957889	-1.88715755	0.05915803	0.138450481	-5.32208283	ns
PCNA	-0.02496192	0.143699095	-1.88685656	0.059198886	0.138450481	-5.31194281	ns
DLGAP5	0.019927081	0.113455707	1.886288153	0.059274784	0.138450481	-5.3294767	ns
BDNF	-0.03121055	-0.09279052	-1.8862839	0.059275406	0.138450481	-5.3283224	ns

KAZALD1	0.018344095	-0.03563132	1.885987247	0.0593156	0.138450481	-5.32275444	ns
COL4A4	0.01989538	-0.0504766	1.884585373	0.059504845	0.138781269	-5.32374994	ns
SMTN	-0.03725645	0.163001878	-1.88381268	0.059609055	0.138913361	-5.33240483	ns
PDCD1	-0.01657007	0.010834746	-1.88065208	0.060038	0.139801403	-5.33992952	ns
BLMH	-0.01266888	-0.00352135	-1.87963589	0.060176447	0.140012133	-5.34172684	ns
C7orf50	-0.00908983	0.021179467	-1.87859437	0.060318653	0.140231262	-5.34285093	ns
PM20D1	-0.07658348	-0.5239588	-1.87807807	0.060389584	0.140284475	-5.33536049	ns
CASP10	-0.02935224	-0.00639692	-1.87474886	0.06084661	0.141233785	-5.33691419	ns
TTR	-0.00912667	-0.00211707	-1.87191865	0.061236907	0.142026821	-5.34922115	ns
UBXN1	-0.02605374	0.028770496	-1.86584067	0.062082976	0.143874834	-5.35399704	ns
VWC2L	0.017670479	0.087433601	1.860821087	0.062788339	0.145339399	-5.37686228	ns
CRISP2	0.023076066	-0.04755949	1.860602331	0.062819254	0.145339399	-5.37806079	ns
LTA4H	-0.02324858	0.041349871	-1.86028393	0.062864267	0.145339399	-5.37864909	ns
NGRN	-0.0083021	0.010941475	-1.85773037	0.063226262	0.146060669	-5.38155953	ns
TBCA	-0.03204086	0.01215821	-1.85702843	0.063326509	0.146176606	-5.37028443	ns
GFER	-0.02610016	0.021887839	-1.85499506	0.063616485	0.146555487	-5.3732552	ns
BACH1	-0.02890246	0.030804346	-1.85491227	0.063627924	0.146555487	-5.38292385	ns
GADD45B	0.018602607	0.073312096	1.854819875	0.063641218	0.146555487	-5.3796718	ns
CYTL1	-0.00853618	-0.0130558	-1.85398747	0.063759957	0.146713219	-5.38830451	ns
IL12RB1	-0.01457761	-0.02995799	-1.85354056	0.063824401	0.146745868	-5.37636208	ns
JUN	-0.02092532	0.052226357	-1.8525512	0.063966205	0.146956191	-5.37818245	ns
PLXNB2	-0.00916539	-0.01441118	-1.85202167	0.064041813	0.147014224	-5.38989008	ns
POSTN	0.013636531	0.005027423	1.850411752	0.064273263	0.147429638	-5.39488179	ns
CEBPA	-0.01438226	0.048671643	-1.84778656	0.064652854	0.148183936	-5.38523132	ns
MATN3	-0.01250254	0.008724201	-1.84719708	0.064737951	0.148262602	-5.39308472	ns
CDC27	-0.02422438	0.075676815	-1.84286959	0.065367717	0.149587565	-5.3955156	ns
YJU2	-0.01217846	0.015210591	-1.84246601	0.065426162	0.149604066	-5.40886156	ns
ATP1B4	0.015638954	0.04827829	1.841789164	0.065525694	0.149714419	-5.39536511	ns
DHODH	-0.01758587	0.04496766	-1.83895741	0.065941163	0.150545892	-5.40172825	ns
EBI3_IL27	0.011957749	0.003729888	1.836636039	0.066282782	0.151207596	-5.42124285	ns
NIT1	-0.01771373	-0.06819289	-1.83550955	0.066449908	0.151436561	-5.4089296	ns
PRDX2	-0.02364367	0.061656396	-1.83525881	0.066486874	0.151436561	-5.41395758	ns
EPHB4	-0.00894835	-0.01053221	-1.83424272	0.066637117	0.151660469	-5.42543922	ns
DNAJB14	-0.0238879	0.022745037	-1.83230258	0.066925574	0.152198345	-5.42707574	ns
CEP43	-0.02683418	0.019300825	-1.83143161	0.067056006	0.152376294	-5.41433314	ns
SH3GLB2	-0.02518495	-0.01082004	-1.82806987	0.067558276	0.153281677	-5.43584432	ns
CEACAM21	-0.03382858	0.246080803	-1.82806353	0.067559424	0.153281677	-5.43202221	ns
STX4	-0.02452232	0.071075477	-1.82671849	0.067761583	0.153559302	-5.43201007	ns
ATP2B4	-0.02300902	0.152626899	-1.82655208	0.067786966	0.153559302	-5.42430296	ns
AP1G2	-0.02981027	0.03947534	-1.82546123	0.067950752	0.153811005	-5.43983262	ns
MRI1	-0.02503665	-0.07440542	-1.82384134	0.068195798	0.154246111	-5.43039249	ns
ARHGAP5	-0.02768236	0.160408679	-1.82205398	0.068466668	0.154738908	-5.43126145	ns
SERPINH1	-0.04721853	-0.02301683	-1.82022107	0.068744915	0.155247604	-5.44280338	ns
SELP	-0.02234414	-0.00719233	-1.81937082	0.068874344	0.155419693	-5.44730508	ns
DDR1	0.008508879	-0.00572732	1.816888363	0.069253503	0.156154618	-5.45724294	ns
MLN	-0.03505622	-0.0711332	-1.81333784	0.069799259	0.157263763	-5.45864727	ns
RALY	-0.01789211	0.029304294	-1.81065183	0.070214179	0.158076641	-5.46657133	ns
CEACAM5	0.02471971	-0.01066302	1.810019027	0.070313036	0.158177245	-5.44918218	ns
ILKAP	-0.02262607	0.019983732	-1.80942708	0.070404635	0.158261381	-5.45570424	ns
BMP6	-0.01604996	-0.01019986	-1.80624249	0.070900453	0.159253325	-5.46177151	ns
AMBN	0.01042635	0.017408876	1.80225754	0.071524821	0.160532266	-5.46943955	ns
SLC9A3R1	-0.0307017	0.006415534	-1.80025412	0.071840412	0.161116745	-5.47337084	ns
SFTPA2	-0.01925294	0.029464584	-1.79906728	0.072028075	0.161413645	-5.47471394	ns
BHMT2	0.009298988	-0.01491583	1.797929042	0.072208312	0.161693458	-5.47580723	ns
DNAJC21	-0.01412455	0.045629235	-1.79738816	0.072293476	0.161760115	-5.49107923	ns
LAP3	-0.01881581	-0.01718026	-1.7969207	0.072368178	0.161803277	-5.47897584	ns
SSNA1	-0.01850966	0.035008125	-1.79634482	0.072459206	0.161882846	-5.49186479	ns
TXNDC5	-0.0264032	0.024704223	-1.79595299	0.072521453	0.161898046	-5.49480146	ns
RABEP1	-0.028363	0.025085284	-1.79510973	0.072656149	0.162074832	-5.48255356	ns
CD200	0.010111251	0.016014069	1.794286323	0.072787158	0.162243131	-5.49277433	ns
CKMT1A_CKMT	0.026286798	-0.0308338	1.792622935	0.073053032	0.162711559	-5.49172749	ns
DENND2B	0.01793027	0.084360074	1.791504309	0.073232512	0.162986993	-5.48725615	ns
IFNGR1	-0.0076622	-0.00138398	-1.78849214	0.073716274	0.163938706	-5.50439484	ns
CEP170	-0.02817094	0.081483561	-1.78784497	0.073821098	0.164046884	-5.49467172	ns
TOP1	-0.04047359	-0.06258099	-1.78710093	0.073940757	0.164187842	-5.50831313	ns
FLT3	0.011982289	0.013453869	1.785321909	0.074229259	0.164703219	-5.49917421	ns

C2orf69	-0.02047225	0.071056661	-1.7802466	0.075055693	0.166410497	-5.50839702	ns
CD34	0.009110954	0.007987352	1.77904374	0.07525222	0.166719637	-5.52360288	ns
TNFAIP8	-0.0136016	0.006146925	-1.77822202	0.075387506	0.166892735	-5.51216508	ns
TIMM10	-0.01420099	0.043848896	-1.7765836	0.0756564	0.167237957	-5.5282441	ns
FLI1	-0.04230158	0.050508276	-1.7765071	0.07566958	0.167237957	-5.51476172	ns
CLTA	-0.0174614	0.04076626	-1.77622955	0.075715267	0.167237957	-5.51590601	ns
CCL13	-0.02638501	-0.01076245	-1.77562991	0.075813551	0.167328472	-5.53033887	ns
CLSPN	-0.01371846	0.036394742	-1.77453279	0.075995297	0.167602922	-5.51722867	ns
PALM3	-0.02703267	0.152686517	-1.77361177	0.076147631	0.167812137	-5.51892486	ns
GUK1	0.015159353	0.021779593	1.772713476	0.076296445	0.168013287	-5.52050641	ns
FKBPL	-0.02107382	0.027985797	-1.77047649	0.076667496	0.168608099	-5.53768079	ns
ADA	-0.01330781	0.013489919	-1.7703886	0.07668204	0.168608099	-5.54027301	ns
RAB44	-0.01554296	0.038410105	-1.76688105	0.077267891	0.169768429	-5.54432784	ns
VAMP8	-0.03126317	0.008701051	-1.76152524	0.078169866	0.171621059	-5.53979975	ns
PDGFA	-0.02724543	-0.04660387	-1.76073183	0.078303506	0.171785303	-5.55666679	ns
WWP2	-0.02075385	0.033169945	-1.76011534	0.07840827	0.171879496	-5.55008046	ns
PRKAR2A	-0.02930501	-0.04797224	-1.75978423	0.078464167	0.171879496	-5.5566606	ns
CNTNAP4	-0.03751238	0.363790031	-1.75799399	0.078768769	0.172417395	-5.5474867	ns
TMCO5A	-0.0193508	0.040031296	-1.75618885	0.079076418	0.172961154	-5.55063613	ns
PRG3	-0.01732049	0.011750375	-1.75554263	0.079186307	0.17307187	-5.56475156	ns
GKN1	0.010288457	-0.02270903	1.752400301	0.079725405	0.174119808	-5.55583053	ns
HRC	0.011847789	0.043459423	1.751503638	0.079879525	0.174326019	-5.55852983	ns
GRHPR	-0.02241101	-0.00937699	-1.74687571	0.080678721	0.175849443	-5.580222	ns
LAMTOR5	-0.01451299	0.028809154	-1.7467647	0.080698032	0.175849443	-5.57942558	ns
IRAK1	-0.0264024	0.040088056	-1.74604212	0.08082396	0.175886156	-5.56847031	ns
PPP3R1	-0.01579282	-0.02268153	-1.74597532	0.08083535	0.175886156	-5.57469592	ns
APP	-0.02483951	0.003109772	-1.74322884	0.081313646	0.17679512	-5.58273426	ns
OTOA	-0.01851697	0.09108119	-1.74195176	0.081537277	0.177105414	-5.57601272	ns
CCN2	-0.02192992	-0.01454191	-1.74171921	0.081577665	0.177105414	-5.58341837	ns
MANSC1	-0.009791	-0.00295025	-1.73703115	0.08240162	0.178761315	-5.5950902	ns
NID2	-0.02797097	0.02735187	-1.73506876	0.082748672	0.179380937	-5.59682772	ns
HMOX1	0.01731571	0.017567842	1.733081285	0.083101626	0.180012425	-5.59170097	ns
MMP1	-0.0346289	-0.08202117	-1.73210237	0.08327629	0.180257054	-5.57903363	ns
GPI	-0.01633981	0.021123681	-1.73121168	0.083433942	0.180464527	-5.60613365	ns
ANGPTL7	0.013259083	-0.01204065	1.730000393	0.083650085	0.180798111	-5.61092387	ns
ITPR1	-0.02433378	0.061217129	-1.72766021	0.084069638	0.18139428	-5.59971479	ns
PPP1R12A	-0.03581793	0.033457035	-1.72728138	0.084137145	0.18139428	-5.61186197	ns
LY6D	-0.01308328	-0.01867656	-1.72709421	0.084171	0.18139428	-5.60613422	ns
SPINT1	-0.00884004	-0.01325497	-1.72705596	0.084177804	0.18139428	-5.6085126	ns
LYVE1	0.006660864	0.000175936	1.726727636	0.084236522	0.18139428	-5.6147702	ns
SPRY2	-0.03454799	0.051050357	-1.72485438	0.084574173	0.181987167	-5.6049814	ns
TMPRSS11D	-0.01254801	0.003513503	-1.72396927	0.084733352	0.182195426	-5.61896281	ns
F12	-0.01442359	-0.05682967	-1.72205984	0.085078636	0.182771334	-5.62276409	ns
CD70	-0.01602961	-0.03548506	-1.72179743	0.085126375	0.182771334	-5.61933054	ns
ASS1	-0.02698938	0.017910722	-1.7187464	0.085681148	0.183827297	-5.61405224	ns
VSIG10L	0.011228557	0.011452428	1.718186294	0.085783233	0.183840099	-5.61500797	ns
HS1BP3	-0.03124396	-0.01056884	-1.71785913	0.085842845	0.183840099	-5.61648063	ns
S100G	0.015744676	0.06627761	1.717674254	0.085875992	0.183840099	-5.63122982	ns
ALDH2	-0.01813495	0.109272298	-1.71725151	0.085953269	0.183870729	-5.63019131	ns
ITGB2	0.01049797	0.001628109	1.714260341	0.086501358	0.184907733	-5.62565098	ns
CLEC12A	-0.00665934	-0.00314861	-1.71320258	0.086695892	0.185188006	-5.62315998	ns
CAMSAP1	-0.03792293	0.043670477	-1.71249608	0.086825257	0.185328764	-5.64005003	ns
AARSD1	-0.02104514	0.006943245	-1.7107454	0.087148396	0.185882626	-5.63570758	ns
MARCO	-0.0097789	-0.03160433	-1.71032808	0.087225484	0.18591125	-5.63670165	ns
HS6ST2	-0.01097404	0.026341478	-1.70920141	0.087434284	0.186220357	-5.62995841	ns
DGCR6	-0.01501928	0.039580816	-1.70583503	0.088059395	0.187415038	-5.63783587	ns
CHCHD6	-0.00952523	0.005997195	-1.70475716	0.088259829	0.187704807	-5.65142234	ns
CEP290	-0.01844012	0.057710574	-1.70275426	0.088634421	0.188364272	-5.64827694	ns
NTproBNP	0.034974291	0.013750353	1.702268919	0.088725271	0.188416611	-5.65035753	ns
EIF5A	-0.01106311	0.008168778	-1.70193515	0.088788102	0.188416611	-5.64397452	ns
IQGAP2	-0.02003511	0.000275428	-1.70025233	0.089103799	0.188949232	-5.6534792	ns
FOXO3	-0.03546007	0.111166139	-1.69865627	0.089404648	0.189449617	-5.64907955	ns
PDRG1	-0.01823094	0.06402353	-1.69731622	0.089657478	0.189847597	-5.65157805	ns
CTF1	-0.03083671	0.019650402	-1.69544571	0.090011564	0.190459251	-5.6551457	ns
PRSS2	0.018510318	0.007863912	1.694354325	0.090218256	0.190758368	-5.66541988	ns
STX5	-0.01392281	0.040134448	-1.69364799	0.090352907	0.190861247	-5.65623443	ns

TXNDC9	-0.01796619	0.074873574	-1.69340951	0.090397639	0.190861247	-5.67131732	ns
TG	-0.03756185	0.198544512	-1.69211767	0.090643704	0.191242498	-5.67274232	ns
IL4R	-0.01259609	-0.01938261	-1.69069824	0.09091476	0.191675884	-5.6720267	ns
MIF	-0.0251065	-0.04617923	-1.68816568	0.091399766	0.192470429	-5.67552317	ns
ANPEP	-0.01008618	0.002068346	-1.68804233	0.091423454	0.192470429	-5.67511331	ns
OTUD7B	-0.0216597	0.023676332	-1.68658901	0.091702501	0.192815508	-5.68377897	ns
STX3	-0.01307111	0.064155683	-1.68650118	0.091719432	0.192815508	-5.68291208	ns
ANGPT2	-0.01377966	-0.00772069	-1.6846864	0.092069197	0.19341155	-5.68776179	ns
VEGFC	-0.0199902	-0.02186157	-1.68354984	0.09228892	0.193733749	-5.68592899	ns
ABO	-0.04866049	-0.17263805	-1.68317936	0.092360541	0.193744813	-5.68757087	ns
MEP1B	-0.04313542	-0.20000856	-1.68142191	0.092701391	0.194320216	-5.68538271	ns
DSCAM	-0.01169299	0.00866266	-1.68052809	0.092874733	0.194543917	-5.69391309	ns
CPLX2	0.010294193	0.002855245	1.678598488	0.093251119	0.195192306	-5.68057546	ns
ATP1B1	0.016580018	0.0610765	1.677849031	0.093397363	0.19535838	-5.68301784	ns
IFNAR1	-0.00862931	0.02396281	-1.67628012	0.093703552	0.195858533	-5.69890047	ns
GGCT	-0.01638396	0.019026555	-1.67176114	0.09459167	0.197573445	-5.70692752	ns
ADAMTS4	-0.01178931	-0.01515381	-1.66961731	0.09501592	0.198157396	-5.69635988	ns
PMCH	0.027495902	0.231961657	1.669344647	0.095069919	0.198157396	-5.69715035	ns
RLN2	-0.04148216	0.209122159	-1.66931926	0.095074833	0.198157396	-5.69814045	ns
SNX2	-0.02768518	0.062905844	-1.66818287	0.095299695	0.198484385	-5.71268043	ns
BCAM	0.008449285	0.002000712	1.663814321	0.096169928	0.200154091	-5.71708419	ns
KLK3	-0.02860724	0.561798654	-1.66058154	0.096817767	0.201236842	-5.72583829	ns
RWDD1	-0.02301946	-0.00280278	-1.66053047	0.096828001	0.201236842	-5.72724193	ns
PRDX1	0.030589356	0.10592598	1.659534892	0.09702903	0.201445152	-5.71138467	ns
CNST	-0.03855916	-0.05921551	-1.65934782	0.097066209	0.201445152	-5.72199926	ns
MYDGF	-0.03352915	-0.03194386	-1.65718164	0.097503012	0.202008829	-5.73183764	ns
CPA1	0.020866967	0.018610704	1.657143972	0.097510594	0.202008829	-5.73247996	ns
IFNGR2	-0.01855496	0.106773127	-1.65697358	0.097545359	0.202008829	-5.72507717	ns
VSIR	-0.02180015	0.061948223	-1.65217462	0.098519468	0.203881535	-5.73379405	ns
RAB6B	0.016538897	0.037101954	1.651246227	0.098709146	0.203887779	-5.72816765	ns
IGBP1	-0.0204798	0.021935775	-1.65065998	0.098828254	0.203887779	-5.74332124	ns
CAMKK1	-0.0160126	0.027057884	-1.65037828	0.098886468	0.203887779	-5.72935268	ns
ACTN2	-0.01965329	0.079728462	-1.65024386	0.09891396	0.203887779	-5.72863019	ns
SAA4	-0.01079378	0.03625041	-1.64969766	0.099025059	0.203887779	-5.74392199	ns
NUDT16	-0.02899295	0.000502154	-1.6494772	0.099070263	0.203887779	-5.74249388	ns
DHRS4L2	0.015839831	0.068242472	1.649430273	0.09907978	0.203887779	-5.74432158	ns
NAGA	-0.01972835	0.083597461	-1.64942718	0.099081082	0.203887779	-5.72861174	ns
CCAR2	-0.01811789	0.095377742	-1.64815654	0.099340819	0.204278305	-5.74640733	ns
CALB1	0.01498943	-0.01608964	1.647472221	0.099481559	0.204423752	-5.74121279	ns
LHB	0.031015014	0.17978426	1.645884441	0.099808445	0.204949094	-5.73670702	ns
SF3B4	-0.02677874	0.043033013	-1.64554706	0.099877589	0.204949094	-5.7443606	ns
BRK1	-0.0155189	-0.01238464	-1.64446113	0.100101847	0.205265024	-5.73926988	ns
GYS1	-0.03275281	0.009472521	-1.64278803	0.100447666	0.205731599	-5.74241964	ns
SNAP29	-0.03204681	-0.03306719	-1.64267629	0.100470295	0.205731599	-5.75343898	ns
TIMP4	-0.01355902	-0.00530411	-1.6408044	0.100858488	0.206381769	-5.75484153	ns
AZI2	-0.01858677	0.067031324	-1.63882062	0.101270889	0.20708053	-5.76266092	ns
CSNK2A1	0.014797605	0.038722309	1.636292178	0.101798725	0.208014189	-5.76677311	ns
ABRAXAS2	-0.0218314	0.023782127	-1.63238721	0.102618886	0.209428306	-5.75773206	ns
SPRR1B	-0.02812133	0.165800298	-1.63231288	0.102634214	0.209428306	-5.76492375	ns
TMOD4	0.013297294	-0.0037945	1.631908054	0.102719816	0.209456608	-5.7573138	ns
FLT3LG	0.011564526	-0.01131521	1.62865404	0.103406558	0.210612954	-5.77950695	ns
ARID4B	-0.01472235	0.039316032	-1.62854087	0.103431156	0.210612954	-5.76514028	ns
GPC1	-0.0099652	-0.00822718	-1.61970146	0.105316739	0.214253506	-5.7943972	ns
ANXA10	-0.02698713	0.044104346	-1.61944278	0.105372955	0.214253506	-5.77981169	ns
MAN2B2	-0.01928888	-0.06338169	-1.6191324	0.10543914	0.214253506	-5.79374196	ns
DNAJB1	-0.03093581	-0.03943587	-1.6179875	0.105685908	0.214571211	-5.78901975	ns
GAGE2A	-0.02057687	0.102086336	-1.61761949	0.105765619	0.214571211	-5.78036543	ns
GAS2	-0.01727232	0.061635028	-1.61738463	0.10581594	0.214571211	-5.7890115	ns
ARG1	-0.01850514	0.014040779	-1.6164213	0.106023865	0.214843641	-5.79253699	ns
OGT	-0.02101639	0.108604218	-1.61380486	0.106590427	0.215841918	-5.79475482	ns
GH2	-0.05735596	0.507137291	-1.61306741	0.10675084	0.215984366	-5.78980561	ns
B4GAT1	0.008621546	0.014502876	1.612610688	0.106849647	0.215984366	-5.80038844	ns
XPNPEP2	0.033951879	-0.08891704	1.612414648	0.106892182	0.215984366	-5.80235295	ns
KLK8	-0.01212281	-0.02051705	-1.61212154	0.106956642	0.215984366	-5.78695961	ns
LAT	-0.02673063	-0.11615883	-1.61144791	0.10710245	0.216129339	-5.80719165	ns
MTSS1	-0.03642612	-0.00883268	-1.60955779	0.107514842	0.216811698	-5.807768	ns

COL5A1	0.012324383	0.026862329	1.608840579	0.107672048	0.216978868	-5.79734859	ns
PPP1R9B	-0.03338145	0.021549955	-1.60791113	0.10787497	0.21723787	-5.81284592	ns
KLK1	0.043975855	0.43172829	1.604953211	0.108524812	0.2183959	-5.80901765	ns
TLR1	0.008360945	0.003647543	1.602613522	0.109041013	0.219283579	-5.80521291	ns
CCNE1	-0.03094413	0.258550342	-1.60224477	0.1091225	0.219296422	-5.80579964	ns
NME1	0.006838928	0.006782124	1.601264201	0.109339423	0.219541534	-5.80735921	ns
SLIT2	-0.01792657	-0.00611232	-1.60101374	0.109394839	0.219541534	-5.80927825	ns
MTSS2	-0.03204546	-0.01262639	-1.60031123	0.109550002	0.219701927	-5.82283937	ns
TBCC	-0.02831107	-0.01503337	-1.59853923	0.109943711	0.220340175	-5.81953424	ns
GBP4	0.016713581	0.037923251	1.59732118	0.110215101	0.220732575	-5.81514181	ns
MPHOSPH8	-0.02523594	0.034927246	-1.59518205	0.110692507	0.221536751	-5.81895202	ns
CXCL6	-0.02624601	-0.01521787	-1.59090965	0.111650261	0.223150967	-5.83985293	ns
LTB	-0.01094699	0.01994736	-1.59090283	0.111651905	0.223150967	-5.83774874	ns
RANBP2	0.010580816	0.007332881	1.58980094	0.111900647	0.223495137	-5.82769627	ns
CTBS	-0.00863005	-0.02082867	-1.58872928	0.11214226	0.223824607	-5.83357125	ns
OMG	0.023035587	0.052601493	1.586559377	0.112633141	0.224650801	-5.83902754	ns
PCDH12	0.007856539	0.000365436	1.586096335	0.112737826	0.224706111	-5.84709655	ns
SCN3B	-0.0154372	0.065220673	-1.5857272	0.112822176	0.224720841	-5.83411906	ns
GBP6	-0.0138807	0.046211873	-1.58518054	0.112946354	0.224814829	-5.83314275	ns
DSG3	-0.0113889	-0.00361557	-1.58432814	0.113139532	0.225045935	-5.84841803	ns
CORO1A	-0.03037264	0.098387256	-1.583467	0.113336198	0.225283661	-5.83306363	ns
DRAXIN	-0.01438611	0.000665371	-1.58247648	0.113561547	0.225578039	-5.84490515	ns
AHNAK2	-0.00929848	0.007986883	-1.58151123	0.113782167	0.225820093	-5.83982921	ns
VPS37A	-0.02748295	0.032096605	-1.58126525	0.113838075	0.225820093	-5.84735495	ns
PFKFB2	-0.02641953	0.001772355	-1.58037278	0.114042229	0.226071493	-5.84812125	ns
OXCT1	-0.02176704	0.010908425	-1.57980838	0.114171732	0.226174665	-5.84250195	ns
GZMB	-0.02155748	0.032553069	-1.57823901	0.114531325	0.226733199	-5.85457018	ns
SERPINB1	-0.03084118	0.019040598	-1.57681055	0.114859809	0.227229432	-5.85494016	ns
THTPA	-0.02784619	0.001931599	-1.57550306	0.115160882	0.227670802	-5.86106505	ns
ACE	0.008222381	-0.01228102	1.574888956	0.115302515	0.227796578	-5.86277062	ns
ING1	-0.01567281	0.014180341	-1.57115921	0.116166718	0.229348761	-5.85504101	ns
SERPINE2	-0.02011892	0.019631934	-1.56761484	0.116991476	0.230442611	-5.87526802	ns
PODXL	0.005090929	-0.00496073	1.567491289	0.117020583	0.230442611	-5.86889036	ns
SIRT5	0.01874053	0.092522442	1.567443946	0.117031957	0.230442611	-5.86208727	ns
IMPG1	-0.00993873	0.031766187	-1.56742471	0.117036436	0.230442611	-5.86277215	ns
LMNB1	-0.01864214	-0.01662526	-1.56547159	0.117492888	0.231185467	-5.8757782	ns
KIAA1549	-0.02156983	-0.01014461	-1.56276097	0.118129554	0.232281683	-5.87526476	ns
PFDN6	0.020698495	0.110482804	1.56187047	0.118339442	0.232537799	-5.8707485	ns
SMPD1	-0.01614624	-0.01653308	-1.55867399	0.119094061	0.233863254	-5.88787374	ns
HSDL2	-0.01486032	0.00206624	-1.55755674	0.119359348	0.234226677	-5.87716274	ns
TARBP2	-0.0270247	0.019241948	-1.55716719	0.119451796	0.234250668	-5.8780301	ns
TINAGL1	-0.00734174	-0.01256524	-1.55597287	0.119735283	0.234644533	-5.88546586	ns
PODXL2	0.009012545	0.013059613	1.555435224	0.119862951	0.234644533	-5.89198511	ns
CD28	-0.01191433	0.002871527	-1.55530824	0.119893714	0.234644533	-5.88066454	ns
PGR	-0.03283723	0.317079291	-1.55348643	0.120328016	0.235336777	-5.88219229	ns
RBPMS2	-0.03262841	0.010229808	-1.55042737	0.121059962	0.236609832	-5.88816401	ns
KLRB1	-0.01214131	0.003513487	-1.54814471	0.121607834	0.237388143	-5.9065166	ns
DCLRE1C	0.009911384	0.012530805	1.548093815	0.121620775	0.237388143	-5.88963863	ns
TST	0.025066538	0.043955913	1.547594313	0.121741053	0.237464178	-5.89160941	ns
INSL4	-0.01526102	0.050455278	-1.54620868	0.12207466	0.237955946	-5.90737456	ns
VCAN	0.009909653	0.00580492	1.544689359	0.122441826	0.238512429	-5.91162403	ns
CXCL9	-0.0218031	0.030408137	-1.5441408	0.122574754	0.238612187	-5.90875811	ns
MOCS2	-0.0207874	0.023097775	-1.54311216	0.122824459	0.238938987	-5.90020913	ns
RASGRF1	0.013780642	0.009859614	1.542704611	0.122923525	0.238941021	-5.89790844	ns
CDH15	-0.01862942	0.121763737	-1.54243175	0.122989162	0.238941021	-5.91386189	ns
SLAMF6	-0.01230889	-0.01333314	-1.54007429	0.12356328	0.239896793	-5.90407663	ns
SUSD2	0.009383434	0.000705283	1.535805347	0.124606713	0.241761861	-5.92046423	ns
CEMIP2	-0.00963928	0.00662168	-1.53404358	0.125039972	0.242441379	-5.91199645	ns
METAP2	-0.01928659	-0.05762615	-1.53263527	0.125386155	0.242834762	-5.92987013	ns
ESPL1	0.017427238	0.103070526	1.532544486	0.125409185	0.242834762	-5.91342089	ns
FXN	-0.02405384	-0.00255013	-1.53079082	0.12584148	0.243399106	-5.93350028	ns
BEX3	0.014473066	0.045650722	1.530688931	0.125867346	0.243399106	-5.91624288	ns
SPRING1	-0.01919723	0.102760336	-1.52807792	0.126514246	0.244282928	-5.92020801	ns
USP47	0.026793457	0.166035214	1.527904542	0.126557281	0.244282928	-5.92166866	ns
EHBP1	-0.0233457	0.041242695	-1.52775644	0.126593442	0.244282928	-5.93653553	ns
MLLT1	0.020930662	0.080641185	1.527494987	0.126659025	0.244282928	-5.92109233	ns

CA7	0.010371081	0.021657627	1.525882166	0.127059927	0.244894381	-5.93182306	ns
PDZD2	-0.01864279	0.09565729	-1.52455152	0.127392034	0.245118337	-5.9267502	ns
MORC3	0.008531788	0.020092046	1.524536452	0.12739516	0.245118337	-5.94117181	ns
GATD3	-0.02357025	0.025262772	-1.52440735	0.127427957	0.245118337	-5.92788715	ns
ZBTB16	-0.02361827	0.114089428	-1.52338649	0.127683062	0.245447362	-5.92945866	ns
IKZF2	-0.03061272	0.245988819	-1.52259848	0.12788021	0.245664615	-5.93065037	ns
P4HB	-0.01277904	-0.00640458	-1.52224682	0.12796786	0.245671369	-5.93989856	ns
S100A4	-0.01867199	-0.02685058	-1.52140628	0.128178398	0.245913878	-5.9439774	ns
CD244	-0.00880992	-0.00300112	-1.51946181	0.128666647	0.246688516	-5.95021125	ns
BCAT2	0.016907601	0.087976137	1.519102465	0.128757673	0.246701053	-5.93682411	ns
CCN4	-0.01299426	0.020350445	-1.51778261	0.129089579	0.2471748	-5.95319381	ns
ATG16L1	-0.02620395	-0.00849424	-1.51735973	0.129196327	0.247217087	-5.95092953	ns
WFDC12	-0.02084715	-0.06381056	-1.5157841	0.129594363	0.247816333	-5.95502057	ns
BRD1	0.009446918	0.026491922	1.515314958	0.129713364	0.24788156	-5.94778033	ns
RPS10	0.012007592	0.03458539	1.513184475	0.13025383	0.248751591	-5.94389402	ns
TSC22D1	-0.02045317	0.051698312	-1.51233817	0.130468213	0.248998158	-5.96058577	ns
USP25	-0.0222279	0.006409947	-1.51070407	0.130884316	0.249629133	-5.96127112	ns
MAPK9	-0.01382025	-0.01240264	-1.50858033	0.131426652	0.250499885	-5.96134267	ns
SCARF1	-0.01352776	-0.00530759	-1.50791589	0.131596422	0.250519908	-5.96702725	ns
UNC5D	0.008070393	0.01333901	1.50787026	0.131608746	0.250519908	-5.95152524	ns
RGCC	-0.02309411	-0.03088473	-1.50751918	0.13169798	0.250526451	-5.96781259	ns
ANGPTL1	0.009367493	0.012677382	1.505819651	0.132134466	0.251193126	-5.95445559	ns
MYL3	-0.02170376	0.031650908	-1.50428184	0.132529757	0.251780671	-5.95815056	ns
SCT	0.013667306	0.04387826	1.499947841	0.1336488	0.253741545	-5.96488223	ns
IL5RA	0.01793703	-0.01403151	1.498338548	0.134065885	0.25436802	-5.97663501	ns
GALNT7	0.006692704	-0.00772705	1.497680302	0.134236964	0.25452723	-5.97514243	ns
IST1	-0.02981898	-0.02995086	-1.49725014	0.134348747	0.254534541	-5.97705872	ns
TPSG1	0.007747901	0.01295535	1.496996506	0.134415158	0.254534541	-5.96774761	ns
TP73	0.010290704	-0.00385965	1.495748884	0.134739984	0.254984286	-5.97112761	ns
TUBB3	0.008475117	-0.01333607	1.49298454	0.135462157	0.256184908	-5.9758838	ns
CHRM1	0.01118858	0.020869204	1.492503181	0.135588155	0.256257225	-5.97594316	ns
RAB10	-0.01606651	0.033676488	-1.48935868	0.136413305	0.257533413	-5.99406611	ns
UROS	-0.02064401	0.06859401	-1.48871544	0.136583179	0.257533413	-5.98154968	ns
RAPGEF2	-0.01587907	0.06697825	-1.48859738	0.136614077	0.257533413	-5.9876314	ns
CTSL	-0.00741254	-0.0219277	-1.48858917	0.136616184	0.257533413	-5.98890965	ns
CIAPIN1	-0.02027541	-0.0048109	-1.48440963	0.1377207	0.259448028	-5.99919951	ns
DCN	0.006793357	-0.00308751	1.483105007	0.138067431	0.259933526	-5.99024672	ns
CCL28	0.018233988	0.041427269	1.476388649	0.139860184	0.263139007	-6.00682934	ns
FAM3C	-0.00887162	-0.02927723	-1.47559367	0.140073604	0.263370846	-6.00743619	ns
NFKB1	-0.02217788	0.016001264	-1.47204055	0.141030863	0.265000076	-6.00487511	ns
CD99	-0.0059915	-0.0158305	-1.47026469	0.141510552	0.265730425	-6.01829784	ns
PTTG1	-0.01212393	0.007710266	-1.46899303	0.141855284	0.266206573	-6.01642181	ns
AMPD3	-0.02160236	0.047963447	-1.46846568	0.141998065	0.266303372	-6.02451002	ns
MTIF3	-0.02554061	-0.00924784	-1.46778956	0.142181732	0.266476674	-6.0244115	ns
CDC42BPB	-0.03086455	0.060279564	-1.46678285	0.142455473	0.266801996	-6.02587856	ns
GAD1	0.007771976	-0.00547326	1.466188748	0.142617803	0.266801996	-6.01221264	ns
ITGA6	-0.01671021	0.070687955	-1.46614448	0.142629423	0.266801996	-6.0240268	ns
APOB	0.006661446	-0.03276531	1.463560089	0.14333449	0.267812579	-6.0307488	ns
CLC	-0.02087081	0.045408347	-1.46349284	0.143353103	0.267812579	-6.02569748	ns
SCARB1	-0.01842428	0.101875395	-1.46298008	0.143493686	0.267903812	-6.01926361	ns
ARNT	-0.011742	0.047674603	-1.45947297	0.144455769	0.269527698	-6.02454697	ns
AP3S2	-0.01397776	0.074404045	-1.45831803	0.144773163	0.269812563	-6.0392589	ns
CD14	-0.01270274	-0.0185986	-1.45824807	0.144793249	0.269812563	-6.01970391	ns
GRSF1	-0.02373143	0.073775296	-1.45549317	0.145553797	0.271056817	-6.02462079	ns
SFTPA1	-0.017673	0.089518938	-1.45496956	0.145698554	0.27115346	-6.03063026	ns
EDN1	-0.00825553	-0.02383583	-1.4530774	0.146223076	0.271956295	-6.03333553	ns
PDAP1	-0.02847571	-0.03326092	-1.45155382	0.14664596	0.272569194	-6.04927816	ns
KAZN	-0.03065217	-0.05088238	-1.4500846	0.14705521	0.273033773	-6.05006571	ns
DAG1	-0.01513878	-0.01277484	-1.44998546	0.14708292	0.273033773	-6.04874911	ns
PCYT2	-0.02220662	-0.02545529	-1.44918181	0.147307094	0.273276185	-6.0513655	ns
LPA	-0.05230136	-0.13674861	-1.44844804	0.147512544	0.273483573	-6.0398192	ns
CEP350	0.01096949	0.013706483	1.446637782	0.148019262	0.274152149	-6.04048687	ns
TXNL1	-0.02931023	0.30466603	-1.44648895	0.148060938	0.274152149	-6.04296469	ns
MAG	-0.01362455	-0.02653284	-1.4442783	0.148681436	0.274864797	-6.04626088	ns
DFFA	-0.02101113	0.024625016	-1.44390701	0.148785303	0.274864797	-6.06139922	ns
MCFD2	-0.01360958	0.003837824	-1.44380532	0.148814234	0.274864797	-6.05353875	ns

JMJD1C	-0.01663106	0.054007275	-1.44377779	0.148822344	0.274864797	-6.04459113	ns
SCGB2A2	0.008987444	0.01818387	1.441063273	0.149587637	0.275927887	-6.04967889	ns
ECI2	-0.01786539	0.081604194	-1.44088372	0.149638364	0.275927887	-6.0499358	ns
REST	0.010599026	0.023897697	1.440731371	0.149681429	0.275927887	-6.04895398	ns
SH3BP1	-0.01435286	0.081250058	-1.43812865	0.150417792	0.277110381	-6.06721365	ns
KCNH2	-0.02187091	0.214804303	-1.43752481	0.15058963	0.27725203	-6.05592398	ns
VMO1	-0.02085869	-0.09710761	-1.43579326	0.151081623	0.277982571	-6.0652884	ns
FCGR3B	-0.01538597	-0.04711524	-1.43371187	0.151674951	0.278898525	-6.06819963	ns
ACP1	-0.01293631	0.062168837	-1.43253905	0.152010401	0.279225707	-6.06150048	ns
RGMB	-0.00767148	0.002459327	-1.43241888	0.152044135	0.279225707	-6.07760884	ns
ECSCR	-0.01493537	0.102597749	-1.43117597	0.152400513	0.279704273	-6.06562178	ns
ITGB5	0.011354477	0.024052368	1.429989952	0.152740142	0.280054971	-6.07795798	ns
LZTFL1	-0.02046331	0.015091715	-1.42926291	0.152949306	0.280054971	-6.06822778	ns
NPHS2	0.030059922	0.279344091	1.429059026	0.153008044	0.280054971	-6.0664448	ns
NAA80	-0.03476535	0.011217804	-1.42890934	0.153050957	0.280054971	-6.06818554	ns
MAP3K5	-0.03087924	0.119041837	-1.42883947	0.153071142	0.280054971	-6.0680439	ns
GCC1	-0.03128282	-0.0693852	-1.42801777	0.15330689	0.280310657	-6.08268974	ns
PKLR	-0.0208688	0.06966314	-1.42764872	0.153413324	0.280329729	-6.07897954	ns
PTK7	-0.01145095	-0.0118655	-1.4253673	0.154071768	0.281293109	-6.07320334	ns
STX6	-0.02121929	0.130004856	-1.42515479	0.154133211	0.281293109	-6.07326519	ns
SSBP1	0.010250629	0.023045651	1.424401727	0.154350969	0.281514572	-6.07304294	ns
CXCL3	-0.02940703	-0.09518409	-1.42212856	0.155009293	0.282412764	-6.0850793	ns
FOS	-0.01025617	-0.02025912	-1.42203511	0.15503687	0.282412764	-6.07400546	ns
KDM3A	-0.02612426	0.255803182	-1.42126717	0.155259844	0.282642608	-6.07747165	ns
PYY	-0.0272941	0.091013097	-1.41899551	0.155921058	0.283603745	-6.08101542	ns
USP8	-0.02914952	0.046545447	-1.41874644	0.155993638	0.283603745	-6.08297398	ns
ARHGAP45	-0.02351892	-0.00201376	-1.4182988	0.156124123	0.283603745	-6.08372936	ns
HMGCL	-0.01387391	0.036305756	-1.41812031	0.156176309	0.283603745	-6.08316902	ns
LTO1	-0.01272318	0.019807427	-1.41631012	0.156705346	0.284387577	-6.08617693	ns
TTN	-0.01898501	0.133064876	-1.41344092	0.157546828	0.285737104	-6.08882686	ns
CPTP	0.011385441	0.063736817	1.410892644	0.158296966	0.286919392	-6.09240026	ns
IL13	-0.01534291	0.067688192	-1.40926882	0.158776281	0.28760964	-6.09605537	ns
NLGN2	0.013799144	0.090306197	1.408080068	0.159127979	0.288068009	-6.09818066	ns
PRR4	0.019859657	0.058512286	1.407519083	0.15929419	0.288120134	-6.09557656	ns
SMARCA2	-0.01936861	0.053405972	-1.40731674	0.159354115	0.288120134	-6.09859056	ns
SDCCAG8	-0.03275928	-0.01535221	-1.40557705	0.159869863	0.288873762	-6.11351605	ns
SIGLEC5	-0.03591154	-0.34862665	-1.40500561	0.160039822	0.289002028	-6.11147367	ns
KIRREL1	0.016530252	0.123520414	1.404383149	0.160225384	0.289158295	-6.10334314	ns
DGKA	-0.01525334	0.034660368	-1.40209272	0.160908206	0.290120803	-6.10435023	ns
FNTA	-0.00996232	0.02094789	-1.40150688	0.161082639	0.290120803	-6.11918982	ns
DIPK1C	0.011980336	0.053822815	1.401286223	0.16114903	0.290120803	-6.10700196	ns
RALB	0.00850739	-0.01002296	1.401262789	0.161156145	0.290120803	-6.10550538	ns
CDSN	-0.01273474	-0.00954196	-1.40043643	0.161402677	0.290385593	-6.12206235	ns
YWHAQ	-0.02069565	0.026547805	-1.39718641	0.162377991	0.291859132	-6.11324287	ns
CLEC14A	-0.00896595	-0.01898047	-1.39704035	0.162421606	0.291859132	-6.12255678	ns
CDH1	0.01194389	0.007051752	1.395719198	0.162820119	0.292395294	-6.10307613	ns
RNF168	-0.01165838	0.034100253	-1.39407369	0.163315954	0.293105462	-6.11548299	ns
CSF2RB	-0.01204475	-0.04826485	-1.39093306	0.16426631	0.294546586	-6.11982571	ns
REG3A	0.018004716	0.007334635	1.390342525	0.164444839	0.294546586	-6.1362487	ns
ANGPT1	-0.02546001	-0.07270749	-1.390137	0.164507516	0.294546586	-6.12888001	ns
ATP5IF1	-0.03313936	-0.08554101	-1.39008735	0.164522425	0.294546586	-6.13322524	ns
PTPN1	-0.02401119	0.050322175	-1.38385048	0.166424467	0.29774913	-6.13746489	ns
LUZP2	-0.0120274	0.030133536	-1.38355357	0.166515181	0.29774913	-6.14402021	ns
PTPN9	-0.0106939	0.02560713	-1.38144174	0.167163749	0.298697238	-6.13185432	ns
SNX9	-0.02043395	0.030647864	-1.38115979	0.167249994	0.298697238	-6.14199576	ns
NFKB2	-0.01587052	0.103181734	-1.37987235	0.167646158	0.299221749	-6.14249199	ns
VCPKMT	-0.02427908	0.200195614	-1.37917699	0.167860722	0.299421691	-6.13513386	ns
TERF1	0.007854006	0.013963673	1.378087324	0.168196837	0.299838072	-6.13748627	ns
EXOSC10	-0.01648099	0.148431382	-1.37766591	0.168326969	0.299886974	-6.13840345	ns
TNFRSF17	-0.01093416	-0.03280297	-1.37485491	0.169196771	0.300906223	-6.14397905	ns
CLEC2L	-0.01452702	0.095013194	-1.37472774	0.169236247	0.300906223	-6.14360807	ns
TAGLN3	-0.01680321	-0.00809489	-1.37465192	0.169259887	0.300906223	-6.14132082	ns
PIBF1	-0.02146054	0.025787118	-1.37448413	0.169311275	0.300906223	-6.1582147	ns
PSMD1	-0.01363078	0.032713185	-1.37363415	0.169575125	0.301191827	-6.1593747	ns
ZFYVE19	-0.02475753	-0.00220973	-1.37322436	0.169702519	0.301234867	-6.15782659	ns
PAIP2B	-0.01470668	0.038378239	-1.37231545	0.169985797	0.301326124	-6.14298377	ns

GIPR	-0.01231038	0.022491418	-1.37218657	0.170025835	0.301326124	-6.14554383	ns
GMFG	-0.01982493	-0.01471268	-1.37196789	0.170093305	0.301326124	-6.16087101	ns
PRSS53	0.010307348	0.003924755	1.371732102	0.170166705	0.301326124	-6.16094716	ns
ICAM2	0.009366647	-0.03876842	1.370499202	0.170550957	0.301823511	-6.16345509	ns
APOC1	0.010105245	0.016069799	1.36950218	0.170862248	0.302191256	-6.16233668	ns
LGALS8	-0.01806864	0.045095938	-1.3660093	0.171955853	0.303941337	-6.16990006	ns
CDKN1A	-0.03164001	-0.04700679	-1.36282829	0.172956693	0.305525435	-6.16668622	ns
TLR2	0.00636037	-0.00580661	1.361833684	0.173270472	0.305894667	-6.16704991	ns
CD3E	-0.00951423	0.022095897	-1.36026519	0.173766104	0.306584304	-6.16917	ns
ENTPD2	0.012473405	-0.0141158	1.357584899	0.174615803	0.307870814	-6.16662339	ns
LYPLA2	-0.02933236	0.294517046	-1.35699589	0.17480283	0.307870814	-6.16831432	ns
IL11	-0.01876543	0.099355037	-1.35696824	0.174811579	0.307870814	-6.1678887	ns
OTUD6B	-0.01602369	0.022561018	-1.3553943	0.17531176	0.308306673	-6.18198677	ns
IL34	0.010219838	-0.02777598	1.355383363	0.175315796	0.308306673	-6.1691449	ns
EIF2AK2	-0.02979418	0.030491877	-1.35473269	0.175522599	0.308306673	-6.18112172	ns
PON1	0.006943859	-0.00682404	1.354514681	0.175592003	0.308306673	-6.18335146	ns
MORF4L1	0.011019025	0.042431567	1.35429965	0.175660841	0.308306673	-6.17721113	ns
RNF43	-0.0161243	0.106206631	-1.35420016	0.17569257	0.308306673	-6.17734493	ns
IL10RA	-0.01687807	0.098333949	-1.35317075	0.176021338	0.308597556	-6.1729987	ns
COX5B	-0.02025535	0.011783154	-1.35301854	0.176069701	0.308597556	-6.17991551	ns
SULT1A1	0.037951685	-0.14694227	1.351901299	0.176426609	0.309037611	-6.18894236	ns
NPR1	0.011474837	0.083365112	1.349834203	0.177089497	0.310012788	-6.1760936	ns
DPP10	0.009672159	0.009161886	1.348219929	0.177607558	0.310733414	-6.18883122	ns
EGFLAM	0.0081784	0.02256098	1.347699319	0.177774855	0.310839867	-6.19231616	ns
FSTL1	-0.00592186	-0.01952285	-1.3472243	0.177928249	0.310921895	-6.18051059	ns
EIF4G3	-0.01815299	0.084483404	-1.34666695	0.178107183	0.31104843	-6.19580566	ns
SNU13	-0.01759619	0.150081495	-1.34507436	0.178621515	0.311760206	-6.18246245	ns
SEPTIN3	-0.02017073	-0.00302157	-1.34145199	0.179793371	0.313618067	-6.2027674	ns
HTR1A	0.020049648	0.193318319	1.339539749	0.180415212	0.314514878	-6.191686	ns
ALMS1	-0.01192255	0.03938938	-1.33686442	0.181286817	0.31579399	-6.20051007	ns
SLIRP	-0.01549525	0.093171032	-1.33631737	0.181465802	0.31579399	-6.19291863	ns
GP1BA	-0.0092791	0.007094555	-1.33629339	0.181473395	0.31579399	-6.20010244	ns
CASQ2	0.012912785	0.063475369	1.335403797	0.181764443	0.315924815	-6.19533275	ns
ATRN	-0.00452123	-0.01673262	-1.33540037	0.181764962	0.315924815	-6.20982946	ns
MAVS	-0.02705393	0.080756009	-1.33237742	0.182756383	0.317353689	-6.20029051	ns
AMFR	-0.01875286	0.073026188	-1.33223106	0.18280442	0.317353689	-6.20072351	ns
SKAP2	-0.03023064	-0.01939159	-1.32979352	0.183605986	0.318322591	-6.21466171	ns
SMAD1	-0.02366754	0.030859878	-1.32967484	0.183645591	0.318322591	-6.20386264	ns
GP2	0.02442723	0.113621163	1.329009596	0.183865217	0.318322591	-6.19886861	ns
NFE2	-0.02023611	0.033972294	-1.32893474	0.183889611	0.318322591	-6.20481282	ns
CD163	-0.01249507	-0.02174901	-1.32858969	0.184003123	0.318322591	-6.21445969	ns
RAB27B	-0.02194826	-0.14291056	-1.32854827	0.184016621	0.318322591	-6.21910097	ns
LRPAP1	-0.01650666	0.08690824	-1.32801819	0.184191643	0.318436707	-6.22072813	ns
VSIG2	-0.01427144	0.014193269	-1.32674325	0.184613238	0.318976719	-6.22048324	ns
XRCC4	-0.01042969	0.008474459	-1.32313481	0.185810626	0.320831669	-6.21271629	ns
AFAP1	-0.02255304	0.1946143	-1.322846	0.18590657	0.320831669	-6.21364276	ns
DLG4	-0.0191469	0.079126732	-1.32179426	0.186256177	0.321245148	-6.22774518	ns
AK2	-0.02830584	-0.03490324	-1.31989608	0.18688921	0.322090553	-6.23023538	ns
SERPINB5	0.01350289	-0.03698574	1.319664984	0.186966948	0.322090553	-6.21769134	ns
RYR1	0.009253407	0.028298676	1.317979939	0.187530447	0.322870817	-6.21828739	ns
SOX9	-0.0070531	-0.01590623	-1.31740727	0.187722115	0.323010357	-6.22022742	ns
BAX	-0.02525222	0.023373501	-1.31702746	0.18784948	0.323039153	-6.22003857	ns
ARF6	-0.02830135	-0.07728748	-1.31479153	0.188599473	0.324137999	-6.23582665	ns
MSMB	-0.01985758	-0.09660219	-1.31419778	0.18879944	0.324183637	-6.22985391	ns
ZNF830	0.021411328	0.29561881	1.314052427	0.188848071	0.324183637	-6.23712635	ns
PI16	0.006353762	0.005984555	1.313716441	0.188961116	0.324187108	-6.23741108	ns
LAMA1	0.014150182	0.125759385	1.311496751	0.189710021	0.325270622	-6.22675157	ns
ITGAL	-0.01166011	-0.03994738	-1.3110849	0.189849104	0.325270622	-6.22694683	ns
CLIP2	-0.03230617	-0.05597876	-1.30974134	0.190302963	0.325270622	-6.2396189	ns
MYO6	0.009713784	0.021837581	1.309617986	0.190344483	0.325270622	-6.24467886	ns
CXADR	-0.01429802	0.043997185	-1.30961466	0.190345833	0.325270622	-6.23978367	ns
LGALS4	0.01479256	-0.03602431	1.309608598	0.190347912	0.325270622	-6.23773363	ns
BTD	-0.00540963	-0.0210061	-1.30934078	0.190438359	0.325270622	-6.24405619	ns
QSOX1	-0.01098717	0.00675125	-1.30920764	0.190483823	0.325270622	-6.23476081	ns
SPINT2	-0.01133487	-0.02112183	-1.30648641	0.19140669	0.326655486	-6.24517433	ns
MYLPF	-0.01241803	0.075472313	-1.30611271	0.191534105	0.326682002	-6.23493997	ns

DCTPP1	0.008514046	-0.01273703	1.305563841	0.191720544	0.3268091	-6.24285082	ns
IGFL4	0.019601545	0.178911512	1.301426469	0.193132459	0.329023793	-6.23947507	ns
CSH1	-0.00813172	-0.01500649	-1.30079445	0.193348186	0.329065634	-6.25418869	ns
PRELP	-0.00625179	-0.02761078	-1.30069508	0.193382407	0.329065634	-6.24928731	ns
PFDN2	-0.01545102	0.000587926	-1.29766672	0.194421748	0.330641528	-6.24585725	ns
STAT2	-0.02025442	0.025058675	-1.29693417	0.194673741	0.330877371	-6.24527062	ns
HSP90B1	-0.01886074	0.15075284	-1.29633473	0.194880154	0.331035515	-6.24487633	ns
BLOC1S2	-0.01915411	0.087829121	-1.29463423	0.195466264	0.331838076	-6.25041902	ns
CLEC3B	-0.00559054	0.000759801	-1.29237475	0.196247175	0.332970221	-6.25378197	ns
UROD	-0.01821167	0.00481737	-1.28951115	0.197240135	0.334460623	-6.25688113	ns
KLK14	-0.01276218	0.00705239	-1.28767561	0.197878107	0.335347691	-6.2737856	ns
STX16	-0.01634324	0.08181261	-1.28505727	0.198792059	0.336701167	-6.26178768	ns
CES2	0.015429621	0.022069719	1.284018966	0.199154765	0.336951107	-6.27026776	ns
SEMA6C	0.006911212	0.031047802	1.283975211	0.199170415	0.336951107	-6.26187679	ns
HGS	-0.02156437	0.049616302	-1.28353479	0.199324552	0.337016614	-6.26372938	ns
PLEKHO1	-0.01814463	0.046256373	-1.2823363	0.199743876	0.337530161	-6.27877374	ns
CGA	0.023025348	0.196103708	1.281963902	0.199874647	0.337555794	-6.27564832	ns
NOP56	-0.028113	0.32764567	-1.28023237	0.20048318	0.338387796	-6.26698321	ns
RNF41	-0.01879077	0.040599887	-1.27944254	0.200760995	0.33866095	-6.26893703	ns
CETN3	-0.01986987	0.020868665	-1.27887209	0.200961295	0.338803107	-6.2820917	ns
AKT1S1	-0.01834649	-0.02756892	-1.27737974	0.201487896	0.339494897	-6.2717954	ns
GRK5	-0.01417683	0.073062347	-1.27663141	0.201752085	0.339743995	-6.27316666	ns
SPOCK1	0.006980252	0.00351267	1.275996303	0.201976234	0.339925419	-6.27991592	ns
FDX2	-0.01476297	0.026710124	-1.27453807	0.20249249	0.340516287	-6.27420628	ns
CA8	0.006610564	0.02581641	1.274272656	0.202586499	0.340516287	-6.27420092	ns
BLOC1S3	-0.01052422	0.066570655	-1.27401512	0.20267716	0.340516287	-6.28933761	ns
SLC27A4	-0.0184979	0.076762618	-1.27226128	0.203299933	0.34136619	-6.2782751	ns
CD200R1	0.009548269	0.016221367	1.270562724	0.203903589	0.342183035	-6.28819089	ns
OGFR	-0.00910197	0.002892987	-1.26868916	0.204571253	0.343106294	-6.28969974	ns
REXO2	0.012811734	0.098420301	1.266362938	0.205402796	0.34419338	-6.28451994	ns
ERP29	-0.03017799	-0.04564359	-1.26621622	0.205455158	0.34419338	-6.28644195	ns
PPM1B	-0.0160916	0.110689739	-1.26505605	0.205870843	0.344692008	-6.28582124	ns
FLRT2	0.007518494	-0.00756602	1.264025213	0.206240272	0.345112662	-6.2950149	ns
LSM8	-0.02206879	0.284035071	-1.26250874	0.206785067	0.345826114	-6.29648426	ns
ACAN	0.007733301	-0.00876062	1.261642559	0.207096797	0.346149197	-6.29551081	ns
STAU1	-0.0173698	0.141479601	-1.26018408	0.207622537	0.346783542	-6.29106717	ns
ACTN4	-0.0085792	0.020719715	-1.25993066	0.207713841	0.346783542	-6.29397352	ns
OPLAH	-0.01745677	0.026716267	-1.25710616	0.208734667	0.348288702	-6.29704168	ns
SPTLC1	-0.00938767	0.044138998	-1.25292015	0.21025367	0.35062291	-6.31580992	ns
NCR3LG1	0.00794462	-0.00379248	1.25206196	0.210566225	0.350943708	-6.31578874	ns
GCNT1	-0.00917148	-0.01039401	-1.25131398	0.210839083	0.351198016	-6.31144233	ns
SELL	-0.00570325	0.010235057	-1.24819665	0.211977911	0.352893671	-6.32171569	ns
IGFBP3	-0.00733103	-0.00385257	-1.24714333	0.212363971	0.353334927	-6.31863463	ns
GORASP2	-0.01187464	0.037415115	-1.24418245	0.213451279	0.354941762	-6.3255566	ns
FGL1	-0.01544489	-0.03821862	-1.24105734	0.214603374	0.356462177	-6.3295949	ns
CTNNA1	0.008834191	0.053493578	1.241041401	0.214609762	0.356462177	-6.3178939	ns
SAMD9L	-0.01967468	0.019324208	-1.23996899	0.215005828	0.356917008	-6.3279612	ns
SPARC	-0.01894185	-0.10878091	-1.23649264	0.216294314	0.35885193	-6.33417937	ns
ARG2	-0.01058041	0.049651298	-1.23458861	0.217002428	0.359822311	-6.33770168	ns
PPP2R5A	-0.02926677	0.03624698	-1.23273571	0.217693125	0.360762727	-6.33963676	ns
COMMD1	-0.01535867	0.020862042	-1.23173209	0.218068388	0.361179634	-6.32839798	ns
EFNB2	0.014397066	0.027226755	1.231298488	0.218230404	0.361243073	-6.32919679	ns
IFNL1	-0.01164417	-0.01199736	-1.22769848	0.219579355	0.363270094	-6.33359031	ns
CEP152	-0.01029483	0.020859488	-1.22655998	0.220007243	0.363605113	-6.33258232	ns
UPK3A	0.012485386	0.034600832	1.226496877	0.220030902	0.363605113	-6.3357139	ns
IFNG	-0.02995099	-0.05072172	-1.22435578	0.220837136	0.364658581	-6.33785547	ns
RGL2	0.015615218	0.101106061	1.224140391	0.220918161	0.364658581	-6.34386081	ns
FZD10	-0.01450907	0.078663035	-1.21783919	0.223304098	0.368388682	-6.34556407	ns
CD160	-0.01175055	0.001850212	-1.21703263	0.223610293	0.368570974	-6.3612065	ns
CRACR2A	-0.02268404	0.108605813	-1.21688506	0.223667043	0.368570974	-6.34647573	ns
ROBO1	-0.00605366	-0.00732379	-1.21570375	0.224116483	0.369103288	-6.35686645	ns
GIP	-0.01251905	0.075396273	-1.21512246	0.224338415	0.369260525	-6.34857734	ns
TGFB2	-0.01300133	-0.02043669	-1.21217171	0.225465195	0.370906123	-6.36494023	ns
ADGRV1	0.016749797	0.076882391	1.211200233	0.225837656	0.371309659	-6.35422879	ns
SIGLEC9	-0.0077182	-0.02995732	-1.2075542	0.227237209	0.373282938	-6.37251569	ns
CACNA1C	0.007848168	0.02314259	1.207409249	0.227293515	0.373282938	-6.35878144	ns

CRTAP	-0.01229178	0.062242733	-1.20640505	0.22768023	0.373707854	-6.35932734	ns
FOSB	-0.01026304	0.025276102	-1.2058951	0.227876873	0.373748748	-6.35993622	ns
PRKAB1	-0.01412854	0.058418285	-1.20535588	0.228084827	0.373748748	-6.36077728	ns
THBS2	-0.00893633	0.012417318	-1.20534388	0.228089133	0.373748748	-6.37028734	ns
AGT	0.005384479	0.029067909	1.203828183	0.228674403	0.37449762	-6.37479693	ns
SLC28A1	-0.01460306	0.097488772	-1.20343589	0.228826649	0.374536892	-6.36048186	ns
PPP1CC	-0.02014574	0.042253375	-1.2028304	0.229060391	0.374709435	-6.37614346	ns
APOH	0.009334758	0.002796025	1.202343807	0.229248875	0.37480779	-6.37387436	ns
COX6B1	-0.00772935	0.015733357	-1.19975937	0.230251161	0.376088149	-6.38080781	ns
CASP9	0.014832853	0.027052238	1.199661866	0.230289593	0.376088149	-6.36584837	ns
CINP	0.011062494	0.058903026	1.198299987	0.230818852	0.376741783	-6.37494435	ns
ROBO4	0.004242582	-0.00486291	1.197441366	0.231153392	0.376962544	-6.36975746	ns
F2R	-0.01494961	-0.01192922	-1.19728921	0.2312123	0.376962544	-6.37984184	ns
EBAG9	-0.02461258	-0.01514031	-1.19680927	0.231399485	0.377057196	-6.377151	ns
TCL1B	0.020312153	0.143829201	1.195504054	0.23190897	0.377676627	-6.37274896	ns
ARHGAP30	-0.00970257	0.08474333	-1.19236766	0.233135646	0.379462701	-6.38198679	ns
SPINK4	0.015882473	0.028102072	1.190919621	0.233703563	0.380175156	-6.38717149	ns
CCL24	-0.02157652	0.04480936	-1.18956955	0.234233981	0.38082585	-6.38899196	ns
CABP2	-0.0159592	0.107420937	-1.18887163	0.234508864	0.381060592	-6.38018018	ns
ADGRE2	0.007142254	-0.00303074	1.188297195	0.234734649	0.381215337	-6.3915993	ns
LAT2	-0.03243218	0.02581102	-1.18715031	0.23518677	0.381737281	-6.38913661	ns
CDH22	0.006771851	0.013496106	1.183272411	0.236719628	0.384011841	-6.39271619	ns
ASPSCR1	-0.01615788	0.002254882	-1.18187457	0.237273576	0.384696748	-6.40273962	ns
GLB1	-0.01042142	-0.00268415	-1.18122992	0.237529795	0.384898447	-6.39554815	ns
PGLYRP4	0.014778414	0.107113057	1.180775717	0.237710515	0.384965312	-6.38851222	ns
DNAJB6	-0.02017731	-0.00258238	-1.18046329	0.237834734	0.384965312	-6.38553544	ns
YARS1	-0.03485351	-0.18103958	-1.17914876	0.238357089	0.385403957	-6.4038215	ns
MYH4	0.011062604	0.062714261	1.179118368	0.238369708	0.385403957	-6.39045419	ns
CTSH	-0.01893539	-0.02036375	-1.17499937	0.240013583	0.387847074	-6.39571253	ns
DIPK2B	-0.006338	0.021271387	-1.17368	0.240541682	0.388313846	-6.39854765	ns
COL2A1	-0.0189423	0.076571737	-1.17361351	0.240568407	0.388313846	-6.39780977	ns
PCDH7	-0.00807313	0.044885238	-1.17283913	0.240878388	0.388466801	-6.41119132	ns
CPOX	0.006181623	0.008684269	1.172493694	0.241017479	0.388466801	-6.39784747	ns
MMP15	0.01121826	0.08078203	1.172380703	0.241062275	0.388466801	-6.4130614	ns
SEC31A	-0.01824119	-0.07002778	-1.17097152	0.241628731	0.389164861	-6.40088544	ns
TP53INP1	0.008261076	0.047242235	1.169181168	0.242349144	0.390109978	-6.40365571	ns
LGALS3	-0.00747873	-0.02689129	-1.16815311	0.242763173	0.390399195	-6.4131004	ns
NT5C3A	0.022148712	-0.05079403	1.167960037	0.242841032	0.390399195	-6.41406645	ns
TPD52L2	-0.02541555	-0.09761639	-1.16774032	0.24292991	0.390399195	-6.40545307	ns
GPNMB	-0.00703334	-0.01349286	-1.16576175	0.243729484	0.391468698	-6.40108052	ns
HS3ST3B1	0.009362757	-0.00308259	1.163640527	0.244587869	0.392631433	-6.41658072	ns
DGKZ	-0.00873031	0.008008366	-1.16236008	0.245107596	0.39324955	-6.41132489	ns
MTHFSD	-0.02626896	0.005947298	-1.16028594	0.245950551	0.393938121	-6.4194665	ns
KIR3DL2	-0.02000782	0.221900985	-1.16019926	0.245986126	0.393938121	-6.41122314	ns
EPHA10	-0.01596449	0.094182104	-1.16006134	0.246042213	0.393938121	-6.41378007	ns
PXDNL	0.0135054	0.078730815	1.159976214	0.246076415	0.393938121	-6.42642879	ns
ARAF	-0.0156503	0.068298952	-1.15831915	0.246751939	0.394803103	-6.42173097	ns
EVI2B	0.009544313	0.035949431	1.157145782	0.247231202	0.395353292	-6.41473644	ns
LTA	-0.0099522	-0.01785238	-1.1552493	0.248006342	0.396375762	-6.42873076	ns
TJP3	0.009707356	0.033083203	1.154497379	0.248314609	0.396651345	-6.42083458	ns
OBP2B	0.015731459	-0.03002272	1.153151137	0.248866473	0.397115086	-6.42594531	ns
AOC1	-0.01416631	0.084091405	-1.15312599	0.24887692	0.397115086	-6.42194142	ns
SUMF2	-0.01313901	-0.07254732	-1.15265169	0.249071147	0.397176952	-6.43677239	ns
TDRKH	-0.02133803	0.085080033	-1.15236906	0.249187732	0.397176952	-6.42261227	ns
JAM2	-0.00591405	-0.01175586	-1.14979962	0.250244144	0.398643154	-6.44003364	ns
TSC1	-0.01764644	0.165846253	-1.14843501	0.250807297	0.399305331	-6.42557234	ns
LACTB2	-0.0162492	0.015450258	-1.14812885	0.250933316	0.399305331	-6.43467615	ns
PHACTR2	-0.02646343	-0.03393942	-1.1476914	0.25111367	0.399374682	-6.44228712	ns
CDC26	-0.01633945	0.044959505	-1.14713015	0.251345624	0.399525977	-6.43940473	ns
SCN2B	-0.0066424	0.011103532	-1.14381144	0.252719959	0.401491991	-6.43237095	ns
ZNF75D	0.014195193	0.052044865	1.141441204	0.253704269	0.402664609	-6.44100552	ns
SSH3	-0.00685128	0.020138762	-1.14137075	0.253733863	0.402664609	-6.43273962	ns
SNAP25	0.010470093	0.03154438	1.137537043	0.255331924	0.404980563	-6.44013674	ns
NDUFB7	-0.0202405	0.022389467	-1.13674047	0.255664411	0.405260277	-6.45259545	ns
LY75	-0.00961148	-0.02239787	-1.13645042	0.255785853	0.405260277	-6.45012481	ns
CEP85	-0.01559879	0.061124103	-1.13571097	0.256095644	0.405531064	-6.44129738	ns

FGF19	0.019083468	0.02515215	1.135272525	0.256278801	0.405601138	-6.45278417	ns
RAP1A	-0.01529407	0.117862017	-1.13258396	0.25740707	0.407166113	-6.44352481	ns
ROBO2	-0.0054195	-0.0017756	-1.13104006	0.258055721	0.407971145	-6.46128327	ns
EPHA4	0.006284076	0.001125959	1.129570241	0.258675246	0.408729285	-6.44899345	ns
SEZ6L2	0.005654347	0.005849029	1.128864987	0.258972494	0.408872102	-6.45616955	ns
KLK11	-0.00709029	-0.0091138	-1.12869148	0.259045681	0.408872102	-6.45670741	ns
FHIP2A	-0.01086674	0.014918149	-1.12808679	0.259301271	0.409054409	-6.44891484	ns
ZP4	0.007704313	0.038133797	1.126131276	0.260127946	0.410136934	-6.44989697	ns
CCL2	0.012980595	0.028783068	1.125365972	0.260451705	0.410425785	-6.45849296	ns
CMC1	-0.02042215	0.010765939	-1.12474977	0.260712514	0.410509112	-6.46739929	ns
ECM1	-0.00834908	-0.05004231	-1.12457819	0.260785754	0.410509112	-6.45189922	ns
GNAS	-0.01973629	0.228743839	-1.12221205	0.261789648	0.411867334	-6.47101008	ns
CD48	-0.00699387	-0.02350429	-1.12185304	0.26194244	0.411885797	-6.46672005	ns
PCBP2	-0.01616028	-0.00017281	-1.11788834	0.263631985	0.414319374	-6.47582023	ns
ZNF174	-0.00820001	0.051263643	-1.11480611	0.264951336	0.416168855	-6.46336272	ns
DXO	-0.01020842	0.016189572	-1.11391711	0.265332021	0.416542743	-6.47919666	ns
NMT1	-0.01472134	0.101346928	-1.11280824	0.265808028	0.416928949	-6.48144815	ns
SMS	-0.01428148	0.122758021	-1.11268007	0.265863597	0.416928949	-6.46790984	ns
MENT	0.00488834	0.007306714	1.109960292	0.26703376	0.418539227	-6.4707999	ns
CNTF	0.007610502	-0.00629605	1.108266272	0.267764515	0.419459433	-6.47277731	ns
RANGAP1	-0.01179237	0.059555221	-1.10653188	0.268514068	0.420408086	-6.47378372	ns
M6PR	-0.00886515	0.065963534	-1.10453571	0.269378446	0.421535403	-6.47594839	ns
SCGB3A1	-0.0056801	0.007533294	-1.10379114	0.269701286	0.421814545	-6.4775811	ns
ESAM	-0.00714952	-0.00190025	-1.10317782	0.269967067	0.422004194	-6.49185403	ns
NMRK2	0.00936858	0.067304802	1.10211924	0.270427492	0.42249774	-6.476463	ns
IZUMO1	-0.01055065	0.059786932	-1.10149092	0.270700702	0.422698423	-6.47715047	ns
GJA8	-0.01332785	0.059133844	-1.09988847	0.271398342	0.423561282	-6.47890198	ns
ICA1	-0.0188828	0.050665138	-1.09827131	0.27210354	0.42443501	-6.48326319	ns
RP2	-0.01003583	0.025341801	-1.09687698	0.272712437	0.425157671	-6.49152469	ns
NEDD4L	-0.01081337	-0.03797548	-1.0964478	0.272900417	0.425223703	-6.48351903	ns
NFAT5	-0.01936749	0.141869548	-1.09522827	0.273434188	0.425668265	-6.48484614	ns
ATP6V1F	-0.01581895	0.046970394	-1.09512989	0.273477283	0.425668265	-6.48522059	ns
SYT1	0.008638902	0.032338692	1.094282524	0.273848092	0.426018342	-6.50072891	ns
PDP1	-0.01201249	0.069024917	-1.09352863	0.274179277	0.426306437	-6.4879885	ns
SAT2	-0.01521432	0.05538566	-1.09244669	0.274654047	0.426817359	-6.49535473	ns
NFX1	-0.01504842	0.063418784	-1.08972563	0.275851493	0.428110193	-6.49115816	ns
PPP1R14A	-0.01725114	-0.00945929	-1.08957116	0.275919009	0.428110193	-6.50686427	ns
TFF2	0.012894834	-0.01632929	1.089556059	0.275925816	0.428110193	-6.50218819	ns
LATS1	-0.01856666	-0.00192792	-1.08624399	0.277388474	0.430151007	-6.4945775	ns
JCHAIN	-0.01447452	-0.03091229	-1.08583957	0.277567305	0.430199857	-6.49674529	ns
GDNF	-0.00871841	-0.0150574	-1.08474596	0.278051539	0.430597334	-6.4977281	ns
SLC34A3	0.010892917	0.03523829	1.084594517	0.278118689	0.430597334	-6.49549034	ns
ACY3	0.011895636	0.017321984	1.080016874	0.280151994	0.433515539	-6.50161774	ns
SAFB2	0.006080769	0.00595971	1.077560664	0.281246609	0.434978866	-6.51979083	ns
C1QTNF6	-0.00675548	0.015246609	-1.07503541	0.282375584	0.43633987	-6.52249046	ns
SMAD3	0.011174169	0.055930727	1.074924174	0.282425464	0.43633987	-6.52082767	ns
LARP1	-0.01383583	0.079535597	-1.07442236	0.282650202	0.436456156	-6.52136331	ns
VASP	-0.0155371	0.021510264	-1.07269631	0.283424117	0.437394525	-6.52304951	ns
SORT1	-0.00855021	-0.00768881	-1.07239969	0.283557478	0.437394525	-6.51757557	ns
GABARAPL1	-0.01476771	0.151129101	-1.07191704	0.28377424	0.437497773	-6.51741945	ns
MPI	-0.02005269	0.02168952	-1.07136092	0.284024081	0.437651882	-6.51899813	ns
IL15RA	-0.00791983	-0.0277225	-1.06978755	0.284732042	0.438511373	-6.51392656	ns
HNRNPK	-0.01736792	0.051842254	-1.06876271	0.2851938	0.438990983	-6.51308734	ns
ANXA11	-0.01543653	-0.13165385	-1.06772444	0.285661626	0.439479425	-6.52535171	ns
ANXA5	-0.00667116	-0.01588458	-1.06628986	0.286309742	0.440244574	-6.51675381	ns
BIN2	-0.02114633	0.032309115	-1.06414304	0.287280623	0.441504958	-6.52942416	ns
IL2RB	-0.00853891	0.031361709	-1.06286135	0.287861821	0.441996524	-6.52126099	ns
CLU	-0.00680118	0.027943405	-1.06276915	0.287903216	0.441996524	-6.53452652	ns
PDLIM5	0.025408434	-0.09499585	1.062217183	0.288153626	0.442102376	-6.53609618	ns
KLK13	-0.00992054	-0.01980778	-1.06195118	0.288274974	0.442102376	-6.51933335	ns
CUZD1	0.007882521	0.048279027	1.058404436	0.289888012	0.44432875	-6.52576142	ns
SPTBN2	-0.00681746	0.035285245	-1.05809074	0.290031027	0.44432875	-6.52675029	ns
IL33	-0.00876154	-0.01659228	-1.05729157	0.290395447	0.444653752	-6.52712452	ns
CCL4	-0.01511363	0.017137481	-1.05441579	0.291709191	0.446278893	-6.53839928	ns
FCRL2	-0.01100785	0.008403667	-1.05379211	0.291994529	0.446278893	-6.54465678	ns
COL28A1	-0.01584767	0.109267738	-1.05365999	0.292055457	0.446278893	-6.53073701	ns

RANBP1	-0.01133625	0.010970865	-1.05363239	0.292068138	0.446278893	-6.53049658	ns
NUDT15	-0.01088599	0.061754334	-1.05274014	0.292477027	0.446669936	-6.5305031	ns
CD3G	0.019311845	0.247847091	1.050191284	0.293647055	0.448222374	-6.53282204	ns
NUB1	-0.01893721	0.044894868	-1.04882667	0.294274419	0.4489453	-6.54521487	ns
CHM	-0.012817	0.04361748	-1.04671636	0.295247154	0.450194093	-6.53797864	ns
IL4	-0.02017723	-0.15304078	-1.04589339	0.295626991	0.450430255	-6.53902779	ns
LRP2	0.00756662	0.008463945	1.045712423	0.295710547	0.450430255	-6.5390217	ns
RNASE10	-0.01006552	-0.00605866	-1.04462474	0.296213186	0.450960638	-6.53988129	ns
PITHD1	0.01697421	0.174158417	1.043616569	0.296679658	0.451435435	-6.5372595	ns
IVD	-0.01105539	-0.00277769	-1.04294633	0.296989883	0.451594062	-6.54188848	ns
FXYD5	-0.01452921	0.054979395	-1.04272316	0.297093217	0.451594062	-6.54231558	ns
LILRA3	0.029099446	-0.39521281	1.041344225	0.297732078	0.452189008	-6.5567199	ns
INPPL1	-0.02203342	-0.01308788	-1.04121046	0.297794336	0.452189008	-6.55062814	ns
BPIFB1	0.014555604	-0.01802848	1.040605769	0.298075045	0.452380007	-6.54977808	ns
COL3A1	0.004733663	-0.00211634	1.037674547	0.299437804	0.454212149	-6.56050885	ns
RRAS	-0.02719913	0.130325547	-1.0367263	0.299880015	0.454250213	-6.54831345	ns
TSHB	-0.01729842	0.028700225	-1.03643679	0.300014784	0.454250213	-6.5558422	ns
UFD1	-0.02921723	-0.07061145	-1.03619083	0.300129328	0.454250213	-6.56110061	ns
TJAP1	-0.01870555	0.026097169	-1.0358967	0.300266567	0.454250213	-6.56055013	ns
TFAP2A	-0.0126738	0.062959625	-1.03571709	0.300350756	0.454250213	-6.55001138	ns
CPA2	-0.01439127	0.031789948	-1.03561869	0.300396288	0.454250213	-6.56156966	ns
NOTCH2	0.004323838	0.0187563	1.032232877	0.30197916	0.456407426	-6.56610276	ns
CD33	-0.01785449	-0.21778005	-1.03132879	0.30240282	0.456811296	-6.56523964	ns
BTNL10	0.008122888	0.049262722	1.027931731	0.303998492	0.458984279	-6.55492467	ns
FCRLB	-0.01306147	0.046436327	-1.02586582	0.304971334	0.46021514	-6.55919377	ns
LAMB1	-0.00619544	-0.02319061	-1.02162007	0.306977146	0.462773785	-6.56348334	ns
DCTN1	-0.014557	0.000973225	-1.02160494	0.306983843	0.462773785	-6.57893339	ns
DCUN1D1	-0.01565866	0.195712265	-1.01972432	0.307875584	0.463532376	-6.56633388	ns
SEPTIN9	-0.00632135	0.026481088	-1.01946808	0.307996921	0.463532376	-6.57287727	ns
SPARCL1	0.006089478	0.006737745	1.019232265	0.308108839	0.463532376	-6.57302203	ns
CETN2	-0.01489467	0.059669333	-1.01920496	0.308122035	0.463532376	-6.56619819	ns
CPXM1	-0.01501193	-0.0013521	-1.01586721	0.309708548	0.465497899	-6.58095904	ns
FOXJ3	-0.00907001	0.039407395	-1.01578652	0.309747403	0.465497899	-6.56811502	ns
PDE4D	-0.01238456	0.136357609	-1.01494468	0.310148533	0.465860965	-6.56930653	ns
CCL8	-0.01339851	-0.08009675	-1.01350495	0.310834852	0.466651809	-6.5865623	ns
NUBP1	-0.02051755	0.132931119	-1.0130034	0.3110744	0.466771453	-6.58503436	ns
CYB5A	-0.01463009	0.073296799	-1.01218488	0.311465964	0.46711896	-6.57087568	ns
RBM17	-0.01533382	0.027297952	-1.00950822	0.312746697	0.46879895	-6.59032826	ns
C1QL2	0.008511123	0.037425956	1.008995984	0.312992798	0.468927126	-6.57494199	ns
SHMT1	-0.01874818	-0.02802833	-1.00416354	0.31531559	0.472164884	-6.59097517	ns
FCGR2A	-0.01336278	-0.16488572	-1.00370041	0.315539012	0.472257261	-6.58592097	ns
AGR2	0.021241874	-0.02632782	1.000745856	0.316965858	0.474149747	-6.5822177	ns
NFATC1	-0.02104862	0.017820846	-1.00007934	0.317287883	0.474388437	-6.59481511	ns
ACP6	-0.01474499	-0.13529999	-0.99893101	0.31784371	0.474976271	-6.6008546	ns
OMP	-0.00975268	0.061522709	-0.99747805	0.318548531	0.475786041	-6.58861647	ns
ROR1	0.006556208	-0.00128194	0.997121134	0.318721476	0.475800977	-6.59554492	ns
MMP3	-0.01009249	0.007562566	-0.99604554	0.319243702	0.476337051	-6.5990949	ns
CD276	-0.00730062	-0.04616761	-0.99369808	0.320385566	0.477350007	-6.5990651	ns
SUSD1	-0.01040277	-0.0524417	-0.99367798	0.320395625	0.477350007	-6.59213605	ns
CDKN2D	0.020344489	-0.14583199	0.993641766	0.320413019	0.477350007	-6.59869553	ns
HNF1A	-0.01457804	0.140364752	-0.99098639	0.321708091	0.479034995	-6.59436628	ns
RTN4IP1	-0.01799227	-0.01789122	-0.98887142	0.322741466	0.480328787	-6.61080976	ns
ABHD14B	-0.01212581	0.007687654	-0.98835205	0.322995959	0.480405097	-6.60209544	ns
CDKL5	0.010468588	0.076932762	0.988095399	0.323121784	0.480405097	-6.5978671	ns
COL24A1	-0.01084333	-0.05964221	-0.98764841	0.323340323	0.480485365	-6.60989005	ns
SIGLEC15	-0.01693226	0.215549695	-0.9871417	0.323589028	0.480610357	-6.59814069	ns
FAM171A2	0.031473504	0.39026599	0.986654375	0.323827482	0.480705001	-6.61298497	ns
EPHX2	-0.01288634	0.095137112	-0.98634028	0.323982001	0.480705001	-6.59935025	ns
MAMDC4	-0.01073859	0.011583313	-0.98352191	0.325366037	0.482513371	-6.61426615	ns
GABARAP	0.00936002	0.031043897	0.982831066	0.325705979	0.482772314	-6.61494079	ns
SERPINA11	0.008618242	-0.04257808	0.980285377	0.32696056	0.484386015	-6.61901358	ns
IL22RA1	0.009130198	0.035876052	0.977583796	0.328295869	0.486117616	-6.60766125	ns
ITM2A	-0.01247784	-0.00060033	-0.97688553	0.328641228	0.486382354	-6.61758622	ns
PVALB	0.024095504	-0.19307949	0.976393662	0.328884709	0.486496125	-6.62157991	ns
PPP1R2	-0.01676015	-0.00658199	-0.9744515	0.329847809	0.487673723	-6.61774003	ns
EVPL	0.006483654	0.029015047	0.974024024	0.330060043	0.487740549	-6.61687634	ns

LRRN1	0.008267811	0.029754759	0.970863279	0.331631911	0.489815468	-6.61416328	ns
RFC4	-0.00417724	0.007016726	-0.96864946	0.332735279	0.49119667	-6.62868507	ns
SERPINA9	0.01478502	0.001594051	0.967382027	0.333368473	0.491882739	-6.62168749	ns
L1CAM	0.005924528	-0.02232053	0.966935261	0.333591547	0.491963291	-6.62914232	ns
GMPR	-0.01049398	-0.0478651	-0.96642178	0.333848215	0.492093279	-6.6329642	ns
PBK	0.005926073	-0.00099361	0.965778624	0.334170513	0.492319828	-6.61645167	ns
FASLG	0.007756146	0.042671887	0.963600241	0.335261387	0.493677887	-6.63566875	ns
TSPYL1	-0.00777394	0.030297154	-0.96106165	0.336536626	0.495305921	-6.62183115	ns
TK1	-0.00872097	0.034499433	-0.95947097	0.33733673	0.495910454	-6.63084937	ns
RAB3GAP1	-0.01119794	0.078988306	-0.95934594	0.337399883	0.495910454	-6.62566775	ns
FUT3_FUT5	-0.00928798	-0.02595265	-0.95923193	0.337456874	0.495910454	-6.63963866	ns
S100A16	-0.01286014	0.050048385	-0.95680523	0.338680946	0.497458935	-6.62673537	ns
STAMBP	-0.01348536	0.012628826	-0.95561282	0.339283048	0.49809276	-6.63369966	ns
ARL13B	-0.01359243	0.079931612	-0.9548626	0.339662542	0.498321588	-6.62807139	ns
FGFBP1	0.006476411	0.004076195	0.95439762	0.339897356	0.498321588	-6.64110545	ns
GNE	-0.01347318	0.031629852	-0.95405887	0.340069174	0.498321588	-6.62978806	ns
ITGAX	-0.01028087	0.048829583	-0.95366768	0.340266983	0.498321588	-6.63636271	ns
KIR2DS4	-0.03290617	0.940693841	-0.9534242	0.340390087	0.498321588	-6.64288184	ns
CD86	-0.00543787	-0.00165069	-0.95328051	0.340462866	0.498321588	-6.6430179	ns
TNFSF10	-0.00531135	-0.01090176	-0.94983668	0.342210022	0.500527844	-6.64874824	ns
PLXNB3	-0.00958756	0.018616671	-0.94954011	0.342361041	0.500527844	-6.6394313	ns
PLPBP	-0.01388224	0.002447923	-0.9492972	0.342484463	0.500527844	-6.64177047	ns
CLPS	0.01391698	0.003085875	0.948752589	0.342761414	0.500682005	-6.64374469	ns
RAB11FIP3	-0.02073265	-0.04093516	-0.94676479	0.343773419	0.501674878	-6.65050676	ns
CLSTN1	0.01054439	0.03174935	0.946743389	0.343784737	0.501674878	-6.63693349	ns
FUT1	0.012083004	0.076210297	0.944407296	0.344976525	0.503162564	-6.64458197	ns
MICALL2	-0.01360083	0.130576645	-0.94255169	0.345925325	0.504294533	-6.64086774	ns
GLI2	0.006324765	0.027447968	0.940565263	0.346942801	0.505525438	-6.64118429	ns
WAS	-0.01357779	0.044807297	-0.93890039	0.347796899	0.50651718	-6.6444732	ns
JPT2	-0.0164931	-0.03595741	-0.93720413	0.348668202	0.507532976	-6.65811152	ns
GGACT	-0.01336858	0.024296281	-0.93679146	0.348880444	0.507588887	-6.65983627	ns
MARS1	-0.01615546	-0.01219094	-0.93642627	0.349068718	0.507609889	-6.64602045	ns
PPIE	0.011515929	0.125400629	0.935247246	0.349675687	0.508239426	-6.65364124	ns
CAPS	-0.01752722	0.183919936	-0.93488447	0.349862405	0.508257822	-6.66136212	ns
PADI2	-0.01168846	0.060466111	-0.93449878	0.350061602	0.50829432	-6.64856771	ns
CCL11	0.007735892	-0.02863414	0.933600912	0.350524307	0.508713209	-6.66393521	ns
PRL	0.014472662	0.108852202	0.931204727	0.351762169	0.510163514	-6.66595269	ns
TEK	0.004212565	0.00625458	0.930990469	0.351873054	0.510163514	-6.66302221	ns
CIRBP	-0.01795089	-0.07475311	-0.93055734	0.352097117	0.510235029	-6.66427699	ns
FGF7	-0.01502673	0.183344146	-0.92847113	0.353178152	0.511480327	-6.65154116	ns
VPS53	-0.01575927	0.062721379	-0.92822254	0.353306787	0.511480327	-6.66113046	ns
GUCY2C	-0.01539072	0.227597508	-0.92573063	0.354600617	0.513044161	-6.6624359	ns
DYNC1H1	0.008863969	0.047359834	0.92546524	0.354738411	0.513044161	-6.66930745	ns
TDGF1	0.028848903	0.194851732	0.924292658	0.355348765	0.513672473	-6.65779144	ns
GNPDA1	-0.00599079	0.029462089	-0.92010539	0.357532409	0.516573297	-6.66135839	ns
EIF4E	-0.01672193	0.016366396	-0.91870675	0.358263715	0.51729432	-6.66136393	ns
MYCBP2	-0.0125228	0.084376171	-0.91847262	0.358385757	0.51729432	-6.67749634	ns
DAND5	0.010483297	-0.02889761	0.916205165	0.359573992	0.518620072	-6.66364312	ns
FGF3	-0.00565773	0.022298657	-0.91583077	0.35977035	0.518620072	-6.6639837	ns
PNLIPRP1	0.013883681	-0.00369922	0.915702731	0.359837078	0.518620072	-6.6789915	ns
HARS1	-0.01529189	-0.01981114	-0.91189637	0.361837573	0.521246035	-6.67882669	ns
PALLD	0.007385068	0.043042615	0.911413777	0.362092009	0.521355358	-6.66799121	ns
TOP1MT	-0.00913933	0.038744892	-0.910443	0.362603537	0.521617755	-6.6680022	ns
MORN4	-0.01441315	0.11814595	-0.90990259	0.362888483	0.521617755	-6.66849066	ns
ADD1	-0.013069	0.017132858	-0.90977062	0.362957971	0.521617755	-6.67156723	ns
GCLM	-0.00837409	0.058291975	-0.90971239	0.362988794	0.521617755	-6.66987285	ns
CACNB3	-0.01533877	-0.01162826	-0.90735742	0.364231904	0.523146661	-6.6875755	ns
PDE5A	-0.01904174	-0.10372039	-0.90518292	0.365382886	0.524346236	-6.67570174	ns
IL17D	-0.00657109	0.000777489	-0.90510117	0.365426229	0.524346236	-6.67542094	ns
ENTPD6	-0.00547584	-0.04582874	-0.9046379	0.365671713	0.524440767	-6.67606558	ns
PPME1	-0.01203334	-0.01763579	-0.90393113	0.366046156	0.524720067	-6.68323171	ns
ANP32C	0.007559279	0.067964884	0.903389567	0.366333607	0.524874452	-6.67649369	ns
CA11	0.005943099	0.047150199	0.901863601	0.367143769	0.525777246	-6.67788849	ns
PAFAH2	-0.00905987	0.031797092	-0.89975441	0.368265155	0.527124633	-6.68598371	ns
PECAM1	-0.00582885	-0.00117818	-0.89832158	0.369028205	0.52781419	-6.69103016	ns
TWF2	-0.01523753	0.016038867	-0.89817089	0.369108416	0.52781419	-6.69462854	ns

SNX5	-0.00532652	0.045764639	-0.89664667	0.369921802	0.528478466	-6.68252049	ns
PEPD	0.004660827	-0.00093538	0.896621386	0.369934926	0.528478466	-6.69525384	ns
GPIHBP1	-0.01259387	-0.08396748	-0.89543914	0.370566324	0.529121598	-6.6961238	ns
SYNGAP1	0.010355381	0.080529715	0.894461176	0.37108947	0.529453659	-6.68539475	ns
FHIT	-0.01428996	-0.02496578	-0.89432648	0.371161521	0.529453659	-6.6848518	ns
FGF16	-0.00851279	0.023372222	-0.89338345	0.371666164	0.529914648	-6.68414906	ns
IL21R	-0.00956225	0.098147031	-0.89284829	0.371952709	0.529942687	-6.6846237	ns
SUGP1	-0.00736168	0.04969054	-0.89266815	0.372048804	0.529942687	-6.69858453	ns
ATP6V1G1	-0.01311101	0.034673694	-0.89230823	0.372241983	0.529959332	-6.68637432	ns
GIMAP7	-0.00547036	0.012774345	-0.89091551	0.372988649	0.530763575	-6.68842621	ns
CREBZF	0.004679518	0.007433767	0.889993615	0.373483566	0.531208968	-6.68628338	ns
MTHFD2	0.004565217	0.013441766	0.888418875	0.374329262	0.531871383	-6.70343776	ns
SNAP23	-0.0168253	-0.11194088	-0.88815821	0.374469609	0.531871383	-6.6975582	ns
IL1R2	-0.00371612	-0.01122243	-0.88810965	0.374495741	0.531871383	-6.69638481	ns
DHPS	-0.00731699	0.015589513	-0.88764185	0.374747252	0.531924631	-6.70336552	ns
C1QBP	-0.01213795	0.112604731	-0.8873624	0.374897565	0.531924631	-6.70539787	ns
PMS1	-0.00912954	0.028484365	-0.88680651	0.375197192	0.53209121	-6.69150453	ns
MRPL46	-0.00506404	0.007839907	-0.88601927	0.375621282	0.53243405	-6.6921956	ns
DDHD2	-0.01192766	0.006013636	-0.88487684	0.376236764	0.533041587	-6.7075856	ns
WASF1	-0.01791207	0.084448294	-0.88454725	0.376414984	0.533041587	-6.69391455	ns
PARD3	-0.01313878	0.088625529	-0.88411264	0.376649335	0.53311491	-6.69983996	ns
TGFBR1	0.003897373	0.001379211	0.882597673	0.377467797	0.534014519	-6.6936604	ns
RLN1	-0.01275557	0.178978	-0.88098363	0.378340323	0.534888687	-6.71099999	ns
NFASC	0.005045928	-0.0135977	0.880565533	0.378566645	0.534888687	-6.7117202	ns
HEPH	0.004743595	-0.01941702	0.880438913	0.378635245	0.534888687	-6.710695	ns
MTR	0.011438388	0.098553785	0.879485139	0.379152346	0.535360179	-6.69672696	ns
TPPP3	0.007468028	0.026668434	0.878055504	0.379927599	0.536017317	-6.69917145	ns
MSLNL	0.00650298	0.029639386	0.877949869	0.379984879	0.536017317	-6.69926549	ns
AKAP12	0.003386397	0.01417554	0.877327021	0.380322956	0.536195328	-6.70035724	ns
NUDC	-0.01211814	-0.0570737	-0.87671814	0.380653312	0.536195328	-6.71507803	ns
CDON	-0.00497162	-0.01290001	-0.87670251	0.380661957	0.536195328	-6.70911729	ns
RASA1	-0.00613705	-0.0007947	-0.87605977	0.381011506	0.536428928	-6.70090974	ns
ZPR1	0.016836909	0.223972318	0.87502444	0.381574545	0.536860586	-6.70247272	ns
GP5	-0.00633693	-0.03932689	-0.87481984	0.381685814	0.536860586	-6.70253858	ns
VASH1	-0.0152266	0.036014577	-0.87379594	0.382243069	0.537385537	-6.71233265	ns
TSPAN15	-0.00876247	0.077819981	-0.87324869	0.382541488	0.537546268	-6.70215456	ns
GID8	0.005871101	0.040262802	0.872761107	0.382806802	0.537660348	-6.71816174	ns
HIP1	-0.0059116	0.038352973	-0.87202956	0.383206181	0.537962523	-6.70321107	ns
AXIN1	-0.01806293	-0.02693033	-0.871213	0.383651492	0.538328859	-6.71457101	ns
SIRT2	-0.01683469	-0.02350642	-0.87026211	0.384170834	0.538641052	-6.71289606	ns
ARHGAP25	-0.01122609	0.061211885	-0.87013061	0.384242915	0.538641052	-6.70580964	ns
NUMB	-0.01475171	-0.0424034	-0.86776849	0.385534931	0.539987477	-6.70654879	ns
CDK5RAP3	-0.01041361	0.097683472	-0.86746352	0.385701501	0.539987477	-6.72274034	ns
KYAT1	-0.01053862	0.009767978	-0.86736043	0.385758177	0.539987477	-6.71471509	ns
DTD1	-0.01515029	0.014357312	-0.86586914	0.386575645	0.540872489	-6.71027507	ns
CACYBP	-0.01796021	-0.06890145	-0.86543019	0.386816454	0.540950213	-6.70954067	ns
CADPS	-0.00746685	0.059040309	-0.86395347	0.38762716	0.54165215	-6.71137155	ns
TSPAN7	-0.01692896	0.292569993	-0.86384027	0.387689381	0.54165215	-6.71212958	ns
GSTP1	0.012456859	0.065066671	0.861813201	0.388804074	0.542949735	-6.7119505	ns
REG1B	0.010213423	0.042539651	0.860473837	0.389541199	0.543719073	-6.7285454	ns
SCGB1A1	-0.00998112	-0.03176435	-0.86001051	0.389796681	0.543815723	-6.72363895	ns
MPIG6B	-0.01423208	-0.16822671	-0.85929667	0.39019025	0.544104838	-6.72503741	ns
GLP1R	0.006301237	0.040982294	0.854861712	0.392641292	0.547261371	-6.71979215	ns
HSD17B3	-0.00824744	0.038744087	-0.85388521	0.393182203	0.547753833	-6.71876056	ns
KLK10	-0.00715171	-0.01359044	-0.84807046	0.396411915	0.551964286	-6.73515725	ns
PRSS22	-0.00566325	0.007900904	-0.84776463	0.396582559	0.551964286	-6.72358867	ns
TMED4	0.009588935	0.08490461	0.84648636	0.397294955	0.552692363	-6.72500746	ns
MFAP3	0.008825494	0.035822233	0.844265145	0.398534664	0.554152961	-6.72849458	ns
NFU1	-0.01727879	-0.04513239	-0.84341177	0.399011235	0.55455155	-6.74105315	ns
KIR3DL1	-0.0222394	8.5951209784	-0.84292475	0.399283931	0.554666546	-6.72895136	ns
IL3RA	-0.00566543	-0.01526634	-0.84137874	0.400149109	0.555604088	-6.73068201	ns
CDA	-0.00807097	0.005324099	-0.84066236	0.400550127	0.555896564	-6.74357047	ns
ACRV1	0.01246352	0.3736473	0.839511702	0.401195574	0.556313172	-6.73050298	ns
UNC79	-0.00956802	0.06290999	-0.83944794	0.40123135	0.556313172	-6.7296882	ns
POF1B	-0.00734662	0.042908855	-0.83856954	0.401724241	0.556732218	-6.73128803	ns
VIPR1	0.004002655	0.008147347	0.836734574	0.402755041	0.557895977	-6.73408722	ns

ATF2	-0.01261902	0.154533829	-0.83636066	0.402965297	0.557922555	-6.734666	ns	
CD164L2	-0.00609923	0.023539022	-0.83333677	0.404667991	0.559927328	-6.73717501	ns	
CCS	-0.00847055	0.028143193	-0.83310813	0.404796777	0.559927328	-6.74377028	ns	
LMOD2	0.010811011	0.087807676	0.832587325	0.405090696	0.560068576	-6.73845612	ns	
SKIV2L	-0.00649846	0.03970892	-0.83107784	0.405942859	0.560962406	-6.73784249	ns	
ATXN2L	-0.00796488	0.064236122	-0.83076104	0.406121413	0.560962406	-6.75369599	ns	
AIDA	-0.01464814	0.179887789	-0.82944712	0.406864543	0.561652234	-6.74038653	ns	
MSRA	-0.01302271	-0.01907864	-0.82881484	0.407222007	0.561652234	-6.75232032	ns	
TET2	0.016836966	0.202701237	0.828691609	0.407291913	0.561652234	-6.74607878	ns	
TMEM106A	-0.01161002	0.019383319	-0.82851762	0.407390216	0.561652234	-6.75375752	ns	
TMED1	0.009030241	0.028285274	0.827361214	0.408045289	0.561967955	-6.74807317	ns	
WIF1	0.006109785	0.005403827	0.827136841	0.408172221	0.561967955	-6.75748955	ns	
MILR1	-0.00903419	0.004733664	-0.82709446	0.408196587	0.561967955	-6.74251632	ns	
TPRKB	0.010634507	0.082619474	0.826041375	0.408793739	0.562524844	-6.74077507	ns	
CORO6	0.006076871	0.031450729	0.825444092	0.40913257	0.562725909	-6.74433719	ns	
LXN	-0.01146381	0.101115315	-0.82421069	0.4098328	0.563072845	-6.74838489	ns	
CNPY4	-0.01353218	-0.02078161	-0.82407073	0.409912219	0.563072845	-6.75176164	ns	
RTKN2	0.008464739	0.068110995	0.823981203	0.409963311	0.563072845	-6.74367456	ns	
NRP2	0.005051894	-0.00977445	0.823503882	0.410234289	0.563180124	-6.75500582	ns	
IL17A	-0.00754815	0.032403889	-0.82259692	0.410750237	0.563623445	-6.74620053	ns	
FKBP14	-0.01642653	-0.03884897	-0.82048501	0.41195227	0.564995537	-6.76212328	ns	
TNFRSF11B	-0.00503767	-0.02346092	-0.82016088	0.412137156	0.564995537	-6.7553802	ns	
MERTK	-0.00441975	-0.01656015	-0.81943576	0.412550414	0.565296673	-6.76333254	ns	
GLRX5	-0.01439075	0.00724146	-0.81891644	0.412846973	0.565437692	-6.74955514	ns	
MGMT	0.021785486	-0.18246909	0.818419386	0.413130465	0.565560693	-6.75887003	ns	
PTH	-0.01311802	-0.00385061	-0.81775614	0.413509338	0.565814089	-6.74994884	ns	
CDH4	0.00773112	0.042352424	0.816028666	0.414496631	0.566899374	-6.75201155	ns	
SARG	-0.01812702	-0.06807593	-0.81543392	0.414836574	0.567098687	-6.76323272	ns	
RBM19	-0.00774734	0.061012731	-0.81385877	0.415738753	0.568066055	-6.75190673	ns	
MAX	-0.01849872	-0.00364015	-0.8128561	0.416313408	0.568386191	-6.75391439	ns	
MORF4L2	-0.00630779	0.058965624	-0.81277082	0.41636235	0.568386191	-6.7515737	ns	
PIK3AP1	-0.01083105	0.0190266	-0.81209208	0.416751397	0.568651438	-6.7639935	ns	
PRKG1	-0.02081245	-0.03720489	-0.81151863	0.417080651	0.56883489	-6.75554442	ns	
CD69	-0.01533553	-0.07174419	-0.81036286	0.417744332	0.569474066	-6.75959364	ns	
TAP1	-0.00967246	0.077547711	-0.80976201	0.418089592	0.569678772	-6.76238105	ns	
SH2B3	-0.01707504	-0.04187252	-0.80836176	0.418895143	0.570510176	-6.75729117	ns	
RBPMS	-0.01071675	0.131082483	-0.80752677	0.419375833	0.570826628	-6.75666098	ns	
TGM2	-0.01344937	0.029870414	-0.80726993	0.419523552	0.570826628	-6.76441773	ns	
MITD1	-0.01717836	-0.07896957	-0.80693941	0.419713962	0.570826628	-6.76508318	ns	
RCOR1	-0.00774494	0.070401337	-0.8058849	0.42032194	0.571387367	-6.75994297	ns	
GPA33	-0.02386066	0.084181281	-0.80429973	0.421236391	0.572077825	-6.76774945	ns	
DDX25	-0.00606476	0.03802396	-0.80399131	0.421414638	0.572077825	-6.76103343	ns	
TP53	-0.00745215	-0.00850661	-0.80398628	0.421417604	0.572077825	-6.76079423	ns	
PHYKPL	0.006756922	-0.02113701	0.803599178	0.42164111	0.57211526	-6.7618958	ns	
LILRA4	0.008240175	0.083317148	0.802716051	0.422151564	0.572541834	-6.760851	ns	
PCSK7	-0.00736236	0.031842952	-0.80158939	0.422802844	0.573158916	-6.77555792	ns	
CTSV	0.007005001	0.03414461	0.800912149	0.423194813	0.573323872	-6.77673933	ns	
PRKAR1A	-0.01449278	-0.03534093	-0.80070096	0.423317215	0.573323872	-6.7709076	ns	
ZCCHC8	-0.0096735	0.078328402	-0.79907441	0.424259893	0.57394004	-6.76374742	ns	
ENSA	-0.00829268	0.040915338	-0.79877708	0.42443196	0.57394004	-6.77779316	ns	
IGLON5	0.014730435	0.259901331	0.798711082	0.424470586	0.57394004	-6.76282372	ns	
ACOX1	-0.00839964	0.023873932	-0.7985593	0.424558385	0.57394004	-6.7726085	ns	
COMT	-0.014432	-0.0497991	-0.79621328	0.425920737	0.575515295	-6.76384643	ns	
CD93	0.005120293	-0.00281538	0.795374081	0.426408761	0.575795921	-6.75554477	ns	
REEP4	-0.01018982	0.104006957	-0.79517684	0.4265228	0.575795921	-6.78243107	ns	
CBLN1	0.015286011	0.206291956	0.793907861	0.427261567	0.576526698	-6.76969551	ns	
MDM1	-0.00502097	0.006545231	-0.790832	0.429054444	0.578678511	-6.77145448	ns	
YY1	0.004368791	0.011204456	0.789576337	0.429787683	0.578954778	-6.77089621	ns	
GTPBP2	-0.01285584	0.035633194	-0.78951341	0.429824379	0.578954778	-6.77248921	ns	
ANKRD54	-0.01026808	0.086611443	-0.78946265	0.429854095	0.578954778	-6.77228577	ns	
RAB39B	-0.0145044	0.211991087	-0.78710505	0.431232783	0.580543903	-6.77317461	ns	
FOLR3	0.032187506	-2.48704626	0.785771467	0.432013472	0.581043943	-6.78592447	ns	
FOXO1	-0.01446461	-0.01779692	-0.78571454	0.432046877	0.581043943	-6.78492408	ns	
GPHA2	-0.00734047	0.102839806	-0.78545107	0.43220118	0.581043943	-6.78794061	ns	
DDX53	-0.00573534	0.00335894	-0.78418257	0.432945281	0.581607098	-6.77665486	ns	
CD3D	0.005139023	0.032253359	0.784057931	0.433018435	0.581607098	-6.77741344	ns	

FKBP1B	0.018593591	0.054004095	0.782884274	0.433707076	0.582035785	-6.78981093	ns
HMGCS1	0.006873048	0.023525376	0.782835149	0.433736256	0.582035785	-6.7765032	ns
LELP1	-0.011548	0.057995305	-0.78239308	0.433995887	0.582116669	-6.77870842	ns
FADD	-0.01280467	-0.00390822	-0.78177837	0.434356924	0.582333434	-6.78577819	ns
ATP6V1G2	-0.01514164	-0.00683579	-0.77932097	0.435802936	0.583918612	-6.77922909	ns
TSPAN8	-0.01544689	-0.01467177	-0.77908896	0.435939238	0.583918612	-6.79288404	ns
CES3	-0.0121262	0.105642	-0.77666718	0.437367165	0.585342668	-6.78944406	ns
LILRB2	-0.00578768	-0.05750719	-0.77660575	0.437403323	0.585342668	-6.79343781	ns
EVI5	-0.01405021	-0.00236064	-0.77424127	0.438800302	0.586943144	-6.78280394	ns
DBN1	-0.00818138	0.046510328	-0.77281113	0.439646315	0.587805513	-6.78390255	ns
TRPV3	0.00771051	0.049848417	0.770665042	0.44091761	0.589235433	-6.78589103	ns
CA4	0.003868743	-0.00525554	0.76813158	0.44242071	0.590973685	-6.80323606	ns
FAM3B	0.005753545	-0.03352769	0.764899718	0.444343061	0.593270113	-6.79845259	ns
MAGEA3	0.012840337	-0.08175182	0.763212858	0.445348255	0.594340449	-6.79874206	ns
ITGB1BP1	-0.00665799	-0.01081812	-0.75988803	0.447333427	0.596717043	-6.79503854	ns
BANK1	-0.01745543	-0.09860749	-0.75696629	0.44908168	0.598775573	-6.80694885	ns
CX3CL1	0.005240545	0.008456181	0.752216769	0.451932305	0.602301384	-6.80745352	ns
ALCAM	0.003007159	-0.00660483	0.751424889	0.452408389	0.60266081	-6.81584445	ns
LAIR2	-0.01700555	-0.13744056	-0.75024838	0.453116683	0.603329099	-6.80892217	ns
SNAPIN	-0.00874517	0.020923945	-0.74949764	0.453568677	0.603655668	-6.81747464	ns
SNED1	-0.0047498	0.001627895	-0.74875238	0.454018148	0.603978584	-6.80335351	ns
ECE1	0.005626288	-0.01220863	0.746498298	0.455378106	0.605196771	-6.80529551	ns
MAP4K5	-0.01818014	-0.05973833	-0.74644381	0.455410879	0.605196771	-6.81049282	ns
CLASP1	-0.01124395	0.108088646	-0.74620436	0.455555652	0.605196771	-6.80310211	ns
PDXDC1	0.008277457	0.082029105	0.745617286	0.455910303	0.605240011	-6.80353698	ns
LPO	-0.00966223	-0.00400836	-0.74546449	0.456002748	0.605240011	-6.79913238	ns
HMCN2	0.008459992	-0.04793224	0.744042738	0.456862188	0.60610522	-6.80675584	ns
SLC1A4	0.007820534	0.083987891	0.737885133	0.460595709	0.610639487	-6.81164603	ns
CXCL5	-0.01834365	-0.08330736	-0.7377163	0.460698216	0.610639487	-6.81707333	ns
IRAK4	-0.01669226	-0.12338317	-0.73734377	0.460924598	0.610662353	-6.82151083	ns
MINK1	-0.01380683	-0.04931503	-0.73671643	0.461305962	0.610679613	-6.82587881	ns
PPM1A	0.005000837	0.023008063	0.736388528	0.461505768	0.610679613	-6.81249826	ns
DDX39A	-0.00328321	0.007271949	-0.73629112	0.461565037	0.610679613	-6.81126834	ns
LPP	0.009916575	0.01550582	0.735523786	0.462032035	0.610824555	-6.81217297	ns
BRDT	-0.00538512	0.00774433	-0.73542371	0.462092959	0.610824555	-6.81224606	ns
INSR	-0.00242287	0.002053235	-0.73328332	0.463397079	0.612057256	-6.81346317	ns
CD2	-0.00553078	0.022154011	-0.73320519	0.463444723	0.612057256	-6.81386391	ns
ART5	0.009140985	0.126352444	0.731686611	0.464371242	0.612747758	-6.81683114	ns
PLB1	0.00876129	-0.00954359	0.731659871	0.464387256	0.612747758	-6.82956639	ns
COL9A1	-0.0070505	0.032680861	-0.73054496	0.465068295	0.613369206	-6.82646706	ns
SUSD5	0.004905498	-0.0123051	0.723195501	0.469571028	0.619028172	-6.81982585	ns
AIF1L	-0.00842959	0.121793856	-0.72192234	0.470353423	0.619779781	-6.82294669	ns
KCNIP4	0.007902267	0.049283922	0.720596875	0.471168755	0.620574094	-6.82416459	ns
ANKRA2	-0.00821511	0.03782801	-0.71995078	0.471566501	0.620817937	-6.82221483	ns
SOX2	0.003352834	-0.00078117	0.718446608	0.472492795	0.62175708	-6.83871468	ns
FAM172A	-0.00889606	0.025463418	-0.71691295	0.473438915	0.622721455	-6.82652571	ns
ARHGEF12	-0.01610533	-0.03255201	-0.71620219	0.473877408	0.623017573	-6.83677214	ns
IL20	-0.0076996	0.048024629	-0.71299063	0.475862571	0.625345953	-6.82977593	ns
CHAD	0.007174064	0.005613369	0.709769812	0.47785782	0.627685485	-6.83240644	ns
DCUN1D2	-0.01149327	0.144878423	-0.70709154	0.479520559	0.629586345	-6.83132669	ns
FRMD4B	0.010229909	0.107944685	0.702273766	0.482519324	0.633238843	-6.83591139	ns
HDAC8	-0.00889333	0.127582516	-0.70162017	0.482926929	0.633451996	-6.83602305	ns
MESD	-0.01846409	-0.03171814	-0.70131734	0.483115615	0.633451996	-6.84753275	ns
KLK4	-0.00893389	0.014642121	-0.70025091	0.483781119	0.634039887	-6.853744	ns
LYN	-0.01303676	-0.06077442	-0.69978789	0.484070416	0.634134417	-6.84581301	ns
STK4	-0.01317223	-0.01258294	-0.69922245	0.484423648	0.634312579	-6.84649592	ns
SHPK	0.005874996	0.068751513	0.696391253	0.486194441	0.636141232	-6.84717678	ns
HLA-A	-0.00455156	-0.03275361	-0.69629281	0.4862559	0.636141232	-6.85567887	ns
SMC3	-0.00767359	0.048593028	-0.69564982	0.48665892	0.636344311	-6.83929607	ns
BCL2L11	0.006542894	-0.07996345	0.695349256	0.486846983	0.636344311	-6.85139023	ns
TTF2	-0.00693485	0.04208929	-0.69475288	0.487220864	0.636548063	-6.84232976	ns
PYDC1	-0.00577982	0.02500071	-0.69417471	0.487583344	0.636736747	-6.84245803	ns
REG3G	0.00547226	0.001160403	0.693607406	0.487938875	0.636916189	-6.85574292	ns
GPR158	0.004846491	0.046783736	0.691662881	0.489159407	0.63822407	-6.85887046	ns
CDAN1	0.003174169	0.008007341	0.690749475	0.489733335	0.638687512	-6.85846705	ns
TEX33	0.005470655	0.043406608	0.68774034	0.491626759	0.640724574	-6.85312306	ns

LDLRAP1	-0.01344235	-0.0308755	-0.68756954	0.491734168	0.640724574	-6.86167442	ns
PSMD5	-0.00764386	0.079579642	-0.68694152	0.492130123	0.640954487	-6.84768908	ns
CRADD	-0.01134869	-0.01467828	-0.6863939	0.492475198	0.6411115	-6.85322056	ns
ACOT13	-0.01552999	-0.05739855	-0.6858266	0.492832743	0.6411115	-6.86073579	ns
GNGT1	0.00733245	0.068466667	0.685705498	0.492909355	0.6411115	-6.84853154	ns
RELB	-0.0067478	0.077298736	-0.68506417	0.493313987	0.641352111	-6.84742277	ns
CXCL12	-0.00579711	0.002110372	-0.68466866	0.493563533	0.641390972	-6.84943197	ns
GOPC	-0.0150137	-0.06497915	-0.68406248	0.493946043	0.641521552	-6.85912222	ns
GLT8D2	-0.00678821	0.061756019	-0.68381357	0.494103415	0.641521552	-6.84981622	ns
DDX58	-0.00855183	0.063343732	-0.68275993	0.494769066	0.642100298	-6.85028963	ns
IFIT3	0.005179085	0.031379092	0.681158972	0.495781237	0.643128037	-6.85759705	ns
VAT1	-0.00284132	0.004499444	-0.6805943	0.496138537	0.64330574	-6.85897035	ns
SHC1	0.015653725	0.233263641	0.67955575	0.496796266	0.643824196	-6.85028587	ns
EDIL3	0.005276771	-0.00434516	0.679195032	0.497024729	0.643824196	-6.85336785	ns
SEMA7A	-0.00387552	0.004378665	-0.67891822	0.49719985	0.643824196	-6.86504762	ns
ATP1B3	0.00544794	0.053492184	0.677942973	0.497818286	0.644339271	-6.85258643	ns
EDDM3B	0.006977755	0.185020185	0.677481126	0.498110886	0.644432338	-6.86672402	ns
ENTPD5	0.003068452	-0.01061553	0.676187623	0.498931909	0.645128459	-6.85539193	ns
CASP4	0.023092841	0.424528505	0.675937103	0.499090818	0.645128459	-6.86111624	ns
SPINK1	-0.0055141	-0.00950071	-0.67497259	0.499703336	0.645634398	-6.8655394	ns
PRSS27	0.005784972	0.003530051	0.674052781	0.500287979	0.646103891	-6.86132454	ns
TRIM25	-0.01474921	-0.00986909	-0.66949152	0.503191981	0.649567014	-6.87173108	ns
METAP1D	-0.01180255	-0.01158085	-0.66909149	0.503447182	0.649609267	-6.86918285	ns
KLHL41	-0.00670652	0.073846963	-0.66691828	0.504834249	0.651111311	-6.87556686	ns
GRIK2	-0.00542362	0.066927855	-0.66569579	0.505615456	0.651830963	-6.87637596	ns
PACS2	-0.00840558	0.04715147	-0.66478888	0.506195732	0.652291058	-6.86135672	ns
ITIH5	0.006105023	0.028145376	0.663784662	0.506838048	0.652830658	-6.87584769	ns
GTF2IRD1	0.009828334	0.201916723	0.661058203	0.508584963	0.654360412	-6.87099513	ns
CASP7	-0.0111168	-0.01620743	-0.66084262	0.508723066	0.654360412	-6.87854259	ns
ANK2	-0.00951649	0.170783597	-0.66058136	0.508890946	0.654360412	-6.86412575	ns
BTLA	-0.01135476	0.116180113	-0.66053283	0.508922088	0.654360412	-6.86294394	ns
DAB2	-0.01598353	-0.10293728	-0.65748209	0.510880731	0.656229421	-6.88258453	ns
FCER1A	0.00310235	-0.01828823	0.657142389	0.511099108	0.656229421	-6.88199553	ns
CXCL8	0.009500362	-0.00524954	0.656981032	0.511203006	0.656229421	-6.87566714	ns
GET3	-0.00744348	0.09234847	-0.65644706	0.511546275	0.656229421	-6.88166585	ns
NGFR	0.003734937	0.000947915	0.655984714	0.511843704	0.656229421	-6.88196722	ns
CD209	0.005514441	0.0383518	0.65557338	0.51210846	0.656229421	-6.87835054	ns
UXS1	0.007223214	0.002101821	0.655529014	0.512137238	0.656229421	-6.86838561	ns
FAM3D	0.011043193	0.096593424	0.655472125	0.512173579	0.656229421	-6.88095634	ns
OSMR	-0.00285115	-0.01526847	-0.65462836	0.512716846	0.656534789	-6.87957286	ns
NPHS1	0.00447488	0.008526345	0.65440396	0.512861594	0.656534789	-6.86781523	ns
MYOM3	0.012065151	0.000596725	0.653844105	0.513222175	0.65670848	-6.87027485	ns
CRYM	-0.0097621	0.103205441	-0.65234573	0.51418811	0.657321713	-6.87135891	ns
PDLIM7	-0.01805702	0.018858306	-0.65223053	0.514262191	0.657321713	-6.88158542	ns
ERBIN	-0.01462044	-0.05398046	-0.65205304	0.514376752	0.657321713	-6.87785903	ns
SLC13A1	-0.00820043	0.119842829	-0.65101417	0.515047021	0.657890333	-6.88418669	ns
BAG6	-0.00506791	0.019409766	-0.65023327	0.515551469	0.658246737	-6.8724868	ns
PCDHB15	0.005126771	0.013597725	0.647142898	0.517549078	0.660508439	-6.8866826	ns
DDX1	-0.0064935	0.089173718	-0.64562976	0.518528739	0.661274786	-6.88731632	ns
SERPINA12	0.015333169	0.197030936	0.64550042	0.518612901	0.661274786	-6.8691708	ns
TLR4	0.003688464	0.003920223	0.645166788	0.51882895	0.661274786	-6.87377599	ns
UGDH	-0.01297094	0.276952341	-0.64471397	0.519122403	0.661360129	-6.87441016	ns
SLC39A14	0.004417751	0.00447781	0.643779109	0.51972848	0.661843508	-6.8762081	ns
FZD8	-0.00541258	0.031289131	-0.64337074	0.519993351	0.661892147	-6.87713349	ns
ITPA	0.008669128	0.00633964	0.641221122	0.521388804	0.663282358	-6.87664046	ns
TPBGL	-0.00538834	0.053055573	-0.64098857	0.521539826	0.663282358	-6.87799029	ns
TACC3	0.016278149	-0.05320279	0.640028015	0.522163971	0.663787024	-6.88557498	ns
FUT8	0.008036032	0.050723072	0.638275665	0.523303822	0.664013425	-6.88613234	ns
NXPH1	0.005248321	0.010402595	0.638253933	0.523318094	0.664013425	-6.87972547	ns
PTPRR	0.004639228	0.016323987	0.63813846	0.52339298	0.664013425	-6.89318998	ns
LYSMD3	-0.01315721	0.162853046	-0.63810511	0.523414924	0.664013425	-6.87982172	ns
PCSK9	-0.00543431	-0.02306852	-0.63775204	0.52364452	0.664013425	-6.89427212	ns
S100A14	0.006681248	0.084926831	0.637656859	0.523706479	0.664013425	-6.89452616	ns
DBNL	-0.01283961	-0.04412986	-0.63628944	0.524597234	0.664854133	-6.8917865	ns
PIKFYVE	-0.00711013	0.065213124	-0.63523369	0.525285679	0.665437823	-6.88009128	ns
LIPF	-0.00747155	0.015005395	-0.63432109	0.525880917	0.665802765	-6.88194197	ns

PPIB	-0.01303082	-0.09391115	-0.63409272	0.526029787	0.665802765	-6.89004476	ns
ABCA2	-0.00465192	0.000989298	-0.6330653	0.526700632	0.666363018	-6.88180106	ns
NAAA	0.008119148	0.052243935	0.632194928	0.52726904	0.666526342	-6.88751336	ns
ATP6V1D	0.003709962	0.006619696	0.632168463	0.52728625	0.666526342	-6.89054052	ns
CAMLG	-0.00691712	0.086588549	-0.63037189	0.52846083	0.667362127	-6.88469269	ns
DOC2B	-0.00445793	0.019526688	-0.63029748	0.528509497	0.667362127	-6.88473927	ns
TLR3	-0.00836816	-0.12203715	-0.63010817	0.528633082	0.667362127	-6.8996345	ns
TEF	0.00544232	0.016446648	0.628655859	0.529583934	0.66827359	-6.88456363	ns
ACRBP	-0.00479683	0.029913228	-0.62468351	0.532188025	0.671269561	-6.90086905	ns
GRIN2B	0.007161138	0.105742331	0.624219119	0.532493137	0.671364404	-6.88918831	ns
ITIH3	0.005131268	-0.04103274	0.623181358	0.533174608	0.671933472	-6.89817739	ns
HPCAL1	-0.00993659	-0.10820676	-0.621954	0.533981248	0.672659726	-6.90470519	ns
DCDC2C	0.00779232	0.044513062	0.61935579	0.535691391	0.674240099	-6.88911282	ns
TFF1	-0.00968533	0.023579233	-0.61908475	0.53586976	0.674240099	-6.89821673	ns
CLINT1	-0.00610899	0.025287201	-0.61899579	0.535928517	0.674240099	-6.88933418	ns
TPSAB1	-0.0069729	-0.03114601	-0.61573138	0.538080864	0.676544369	-6.90788028	ns
MYL1	0.004966866	0.036114753	0.615468864	0.538254443	0.676544369	-6.89271021	ns
HLA-DRA	0.004592616	-0.00638852	0.615164556	0.538455176	0.676544369	-6.90357715	ns
TAB2	0.013320672	0.011923119	0.611931524	0.540592176	0.678757971	-6.90205151	ns
BST1	-0.00629842	-0.13496272	-0.6114684	0.540898499	0.678757971	-6.9109262	ns
IL2	0.004116314	-0.01581604	0.611444857	0.540914315	0.678757971	-6.89655884	ns
NELL2	0.003703614	0.012721347	0.609164471	0.542424405	0.680360508	-6.90856428	ns
TRDMT1	-0.01039689	0.072068488	-0.60826315	0.543022009	0.680817632	-6.90427437	ns
PDGFRA	0.00364648	-0.00175355	0.6070422	0.54383191	0.68154042	-6.90629363	ns
KRT14	0.003642781	0.012872668	0.605438292	0.544897239	0.682582556	-6.88477655	ns
FBN2	-0.00723654	-0.03299428	-0.60478583	0.545330389	0.682806978	-6.90038544	ns
KEL	-0.00470857	-0.00622713	-0.60430828	0.5456478	0.682806978	-6.90067024	ns
CGREF1	-0.00544648	-0.01338478	-0.60411234	0.545777907	0.682806978	-6.90805586	ns
PRKCQ	-0.00565472	0.043364403	-0.60231896	0.546970882	0.684006413	-6.90205912	ns
TSNAX	-0.00485471	0.028248271	-0.60150477	0.547512906	0.684391133	-6.90114862	ns
KCNC4	0.006820521	0.109911548	0.600778821	0.547996342	0.684702319	-6.90344689	ns
FUCA1	-0.01073793	-0.2108768	-0.60023371	0.5483593	0.684862769	-6.91518567	ns
CSNK1D	-0.00764384	0.102801037	-0.59813006	0.54976216	0.686321295	-6.90281459	ns
BTC	-0.00966083	0.089865594	-0.59757594	0.550131918	0.686489402	-6.90444642	ns
IL36G	-0.00808922	0.077347882	-0.59707671	0.550465201	0.68661187	-6.90163786	ns
EFCAB2	0.004194861	0.026918773	0.596342343	0.550955391	0.686929864	-6.91140553	ns
TMED10	0.006076713	0.0723704	0.595803063	0.551315767	0.686991306	-6.90453844	ns
SERPINB6	-0.00773207	0.008138884	-0.59556407	0.551475212	0.686991306	-6.91871713	ns
CERT	-0.00777498	-0.00329397	-0.59433588	0.552296407	0.687720899	-6.90660583	ns
FAP	0.003380056	-0.00573277	0.591963467	0.55388368	0.689403387	-6.92223743	ns
ESR1	-0.00695908	0.099098763	-0.58753155	0.556855593	0.692807129	-6.90905439	ns
SNX18	0.005232726	0.09396236	0.585828678	0.5579992	0.693934269	-6.92389057	ns
KRT19	0.008061428	0.016545685	0.583265449	0.559723205	0.695781933	-6.92161335	ns
SV2A	-0.00719316	0.061479963	-0.58216328	0.560465387	0.696408055	-6.91371905	ns
SERPINB9	-0.00420861	-0.06070617	-0.57952911	0.562240758	0.698316892	-6.92166045	ns
PER3	-0.01378848	0.093361329	-0.57892328	0.562649612	0.69852758	-6.91404035	ns
NECTIN1	-0.00378676	0.008548204	-0.5770751	0.563897364	0.699779134	-6.91510124	ns
PLXDC1	0.004225177	0.029775412	0.574961972	0.565325616	0.701253525	-6.91761311	ns
SRC	0.015021271	-0.05705954	0.573816689	0.566100231	0.70191621	-6.92894758	ns
MAP1LC3B2	0.007311091	0.15389875	0.572672356	0.566875123	0.702578676	-6.91795916	ns
BRSK2	-0.00446134	0.068094003	-0.57012369	0.568602057	0.704420029	-6.93503638	ns
ADGRE1	-0.0061822	0.019804601	-0.56840481	0.569768382	0.705565596	-6.93387875	ns
COPB2	-0.00492854	0.014551357	-0.56772904	0.570227451	0.705834743	-6.92075843	ns
TMEM132A	-0.00724367	0.003499861	-0.56727525	0.570535633	0.70591697	-6.92194591	ns
ZP3	0.029914034	-1.06320738	0.566409405	0.571123933	0.706345567	-6.92243333	ns
MZT1	-0.00627515	0.068944765	-0.56422722	0.572607914	0.707619901	-6.92368552	ns
TRIM5	0.010488819	-0.00204847	0.564181326	0.572638982	0.707619901	-6.93343782	ns
SCIN	-0.00836335	0.081131353	-0.56276977	0.573600032	0.708507654	-6.92540778	ns
MED18	-0.00929952	-0.01869304	-0.56160569	0.574393069	0.709187214	-6.92515108	ns
ATXN2	-0.01273967	0.226510673	-0.56072456	0.574993652	0.709628683	-6.92654879	ns
FKBP7	-0.00568947	0.046984911	-0.55948597	0.575838439	0.710371036	-6.925297	ns
FIS1	-0.00928691	0.041544459	-0.55795163	0.576885562	0.711197207	-6.93533031	ns
NUDT10	0.002702311	0.022348909	0.557430797	0.577241403	0.711197207	-6.9274439	ns
STX1B	-0.00478716	0.03973848	-0.55727636	0.57734688	0.711197207	-6.92780018	ns
KHDC3L	0.007421222	0.114029158	0.556731024	0.57771951	0.711197207	-6.92568452	ns
ZNRF4	-0.00609179	0.023850275	-0.55672126	0.57772595	0.711197207	-6.94074545	ns

SPINK5	0.003944219	0.02931036	0.555714277	0.578414284	0.711588154	-6.93842938	ns
TARS1	0.004134435	0.01433718	0.555385595	0.578638985	0.711588154	-6.94148317	ns
HIF1A	-0.00738269	0.100497978	-0.5551876	0.578774612	0.711588154	-6.92740702	ns
LAYN	0.003724438	-0.00549234	0.554135572	0.579494201	0.712173008	-6.9346974	ns
CNPY2	0.006956929	0.093270407	0.553628509	0.579841327	0.712299821	-6.93023675	ns
MCTS1	-0.002855	0.037621962	-0.55316341	0.580159513	0.712390992	-6.94236857	ns
ID4	-0.00495169	0.076025211	-0.55260041	0.580545151	0.712564876	-6.93636337	ns
CHMP1A	-0.00943348	-0.05802189	-0.55205665	0.580917624	0.712722462	-6.93709872	ns
FABP1	-0.01131653	-0.09211696	-0.55129047	0.581442606	0.712980344	-6.93850112	ns
MEP1A	-0.00779488	0.00702397	-0.55103717	0.581616157	0.712980344	-6.9435346	ns
TBC1D23	-0.01085594	0.017106392	-0.54902295	0.582997713	0.714374033	-6.93931303	ns
ADAM9	-0.00313773	-0.00934361	-0.54628275	0.58487947	0.716379217	-6.94612567	ns
GFAP	0.005061369	0.000249587	0.545683548	0.585291589	0.716583414	-6.9331913	ns
TIA1	-0.01414098	0.008295272	-0.5446441	0.586006454	0.717157941	-6.93025641	ns
FGFBP2	-0.00543993	0.026673303	-0.54387756	0.586533561	0.717502304	-6.94836923	ns
C1QA	-0.00266424	9.8688376300	-0.54282072	0.587261066	0.718091421	-6.94903683	ns
TSLP	0.007254793	0.094718357	0.541353173	0.588272181	0.719026693	-6.93691124	ns
MANSC4	0.00686442	0.052623327	0.536936391	0.591319499	0.722360655	-6.93764906	ns
MRPL58	-0.00788071	0.125381575	-0.53640019	0.591689689	0.722360655	-6.95356976	ns
FAM171B	0.002877548	0.026016658	0.536324509	0.591742016	0.722360655	-6.95181806	ns
BATF	0.009246683	0.214586247	0.535694281	0.592177739	0.722590471	-6.93951343	ns
PTPN6	0.010344564	-0.16876903	0.535162781	0.592545037	0.722736636	-6.94950719	ns
COPE	-0.0052676	0.040668882	-0.53202336	0.594717663	0.725083747	-6.94145781	ns
REG4	-0.0052986	-0.02641816	-0.53163084	0.594989388	0.725112275	-6.95046129	ns
GABRA4	0.006873709	0.120533067	0.530954741	0.595457963	0.725366268	-6.94268684	ns
NUP50	-0.00344788	0.034373407	-0.53061328	0.595694627	0.725366268	-6.94100094	ns
MCEE	-0.00456792	0.031594142	-0.52906118	0.596770576	0.726373523	-6.95745338	ns
SERPINC1	0.001553559	0.002459017	0.526410183	0.598610795	0.7283098	-6.95614706	ns
MMP10	-0.00680269	0.044186699	-0.52558766	0.599182357	0.728701576	-6.95172202	ns
PNLIPRP2	0.033880995	-0.91199875	0.52356553	0.600588333	0.729322363	-6.95330042	ns
MAGED1	-0.00538912	0.037134512	-0.52314923	0.600878112	0.729322363	-6.94586562	ns
OGA	-0.00746987	0.01674354	-0.52314735	0.600879212	0.729322363	-6.9584136	ns
AKR7L	-0.00729249	0.074744932	-0.52283763	0.601094958	0.729322363	-6.94472389	ns
NPPB	0.014153207	-0.10626043	0.522770247	0.601141834	0.729322363	-6.94673064	ns
EIF2AK3	-0.00715058	0.105939308	-0.52269903	0.601191414	0.729322363	-6.94514032	ns
TCN2	0.003319408	-0.01894231	0.518336232	0.604231104	0.732705492	-6.96184633	ns
GIT1	-0.01026895	0.003260664	-0.51783901	0.604578215	0.732822079	-6.94730694	ns
DBH	0.011386206	-0.1734946	0.517061188	0.605120853	0.733175474	-6.96061051	ns
FGF12	0.004823399	0.065087724	0.516464402	0.605537691	0.733268981	-6.94801292	ns
DOCK9	-0.01210832	0.374213515	-0.51623155	0.605700268	0.733268981	-6.95034292	ns
SEMA3G	-0.00446579	0.028069017	-0.51518101	0.606434057	0.733853065	-6.95076863	ns
HEBP1	-0.00579046	0.021654101	-0.51188398	0.608739716	0.736338016	-6.95232375	ns
EIF5	0.00447455	0.045853798	0.511195563	0.609221633	0.736615805	-6.95104607	ns
MTPN	-0.00363889	0.025931889	-0.51020893	0.609912573	0.737145991	-6.95317391	ns
GP6	-0.00976879	-0.0482244	-0.50914215	0.610659914	0.737588697	-6.96202814	ns
GRP	-0.00537589	-0.0078918	-0.5089652	0.610784065	0.737588697	-6.95310803	ns
KLK7	-0.00399792	-0.00131242	-0.50833142	0.611228534	0.737820306	-6.9416368	ns
MRPL28	-0.00372727	0.041628354	-0.50622732	0.612704268	0.73929474	-6.96919432	ns
RAB2B	-0.00622675	0.019342448	-0.50586825	0.612956358	0.73929474	-6.96758242	ns
CALCB	-0.00389507	0.028331241	-0.50539522	0.61328846	0.739389886	-6.96858033	ns
SAG	-0.00536099	0.010505829	-0.50412405	0.614181575	0.74016104	-6.95647753	ns
MDK	-0.00550565	-0.00417166	-0.50280249	0.615110318	0.740974476	-6.96792217	ns
CRNN	0.00723169	0.007228069	0.50118841	0.616245927	0.742036332	-6.95303139	ns
UPK3BL1	-0.00541574	0.070514426	-0.49898409	0.617797799	0.743504043	-6.96293279	ns
PNMA2	-0.00448563	0.013310812	-0.49873398	0.617974079	0.743504043	-6.95916173	ns
FABP9	0.006020137	0.02416113	0.497850496	0.618596557	0.743946435	-6.96842313	ns
SFTPD	-0.00712144	-0.00148899	-0.4972916	0.618990577	0.744113827	-6.96648388	ns
DENR	0.005744433	0.057299741	0.496568159	0.619500871	0.7444208	-6.95802126	ns
DOK1	0.013600236	-0.15897007	0.494870155	0.620698793	0.745553466	-6.97380907	ns
LYPD1	0.006826588	0.163751606	0.493998195	0.621314655	0.745826233	-6.96083114	ns
CGB3_CGB5_C	0.004223752	-0.00984943	0.493778378	0.621469711	0.745826233	-6.97342948	ns
SERPINI1	-0.00296965	0.028351068	-0.49346372	0.621692141	0.745826233	-6.96053036	ns
NDRG1	-0.00740123	0.134455926	-0.4919079	0.622791622	0.746838413	-6.96185448	ns
NRGN	-0.00890388	-0.02731205	-0.49030414	0.623925646	0.747891169	-6.9751275	ns
PTPRM	-0.00254252	-0.00924396	-0.48911093	0.624770165	0.748596176	-6.97169236	ns
IL5	0.015278843	0.307193057	0.487766115	0.625722594	0.74942985	-6.96406646	ns

HTR1B	-0.00421129	0.067602951	-0.48657312	0.626567951	0.750134653	-6.96511174	ns
RILPL2	-0.00960844	-0.07044128	-0.48573867	0.627159336	0.750373964	-6.97850421	ns
NLGN1	-0.00660126	0.147300529	-0.48556621	0.627281796	0.750373964	-6.96493453	ns
CEP112	-0.0043195	0.066259552	-0.48346424	0.628773187	0.751850002	-6.96474274	ns
POMC	0.006705349	-0.02480853	0.483013328	0.629093286	0.751924844	-6.96589144	ns
INPP1	-0.00560871	0.027317281	-0.48236458	0.629553876	0.752167479	-6.9734555	ns
ADGRB3	0.003425601	0.013372381	0.480158678	0.631121458	0.753731966	-6.97290517	ns
SLC12A2	0.002549331	0.01478247	0.479512795	0.631580844	0.753765388	-6.96663222	ns
CLEC4M	0.004650681	0.040276335	0.479393191	0.63166572	0.753765388	-6.98009285	ns
BRD3	-0.00463449	0.040608058	-0.4786395	0.632202073	0.754046878	-6.96704772	ns
RRP15	0.008836777	0.006131283	0.477843295	0.632768671	0.754046878	-6.96929246	ns
ANXA4	-0.00736257	0.023679777	-0.47761371	0.63293202	0.754046878	-6.97408269	ns
ENO2	-0.00679925	0.024600322	-0.47760999	0.632934554	0.754046878	-6.98333095	ns
ELAC1	-0.00819587	-0.03533095	-0.47682987	0.633489986	0.754400799	-6.98354761	ns
DUSP29	-0.00618621	0.098036284	-0.47498052	0.63480772	0.75566186	-6.96998364	ns
LRFN2	-0.0045574	0.057596577	-0.47337569	0.635952007	0.75671551	-6.9707394	ns
IL20RB	-0.00402849	0.00850253	-0.47204196	0.636903692	0.757539218	-6.96981761	ns
ANKMY2	0.007654233	0.016214311	0.47100025	0.63764739	0.758114975	-6.97030542	ns
CCL15	0.004296885	0.03510263	0.470146318	0.638257094	0.758531019	-6.9864971	ns
IKBKG	-0.00925399	-0.00835634	-0.46729217	0.640297495	0.760646332	-6.98307035	ns
PRTFDC1	0.011371278	-0.15489868	0.466652822	0.640755035	0.760732629	-6.97387563	ns
PRKD2	-0.00445677	0.08054571	-0.46646258	0.640891187	0.760732629	-6.97276233	ns
STAM	-0.00351702	0.041060788	-0.46598768	0.641230887	0.760826571	-6.98683176	ns
KIAA2013	0.005464905	0.130391587	0.463933352	0.642702033	0.762262362	-6.98778055	ns
FRMD7	-0.00304585	-0.00560272	-0.46242412	0.643783859	0.763235431	-6.97582845	ns
IL3	0.005580768	-0.06562363	0.461636547	0.644348665	0.763595009	-6.9737709	ns
DCTD	-0.00878521	-0.05431009	-0.4607227	0.645004181	0.764061748	-6.97715939	ns
B3GNT7	0.003350335	-0.0376696	0.460294249	0.645311666	0.764116003	-6.97653337	ns
ITIH1	0.001715994	-0.0184061	0.459388107	0.645961932	0.764575939	-6.99066453	ns
BSND	0.003199295	0.025292972	0.458456647	0.646631034	0.765057788	-6.97522371	ns
KLK15	-0.0047	0.013451287	-0.45753271	0.647294642	0.765532748	-6.98983894	ns
BABAM1	0.002640584	0.022090663	0.456823267	0.64780468	0.765825775	-6.97683699	ns
GP1BB	-0.00623265	0.0065882	-0.45625881	0.648210468	0.765995373	-6.97743755	ns
TNFSF8	-0.00322298	-0.00572543	-0.45580662	0.648535435	0.766069365	-6.99209638	ns
DUOX2	-0.00712439	0.152954815	-0.45385302	0.649941154	0.767419397	-6.98015283	ns
CSRP3	0.008762372	0.120643488	0.453023291	0.650538509	0.767814246	-6.97889843	ns
CCND2	0.003399896	-0.02658451	0.452612719	0.650834069	0.76785272	-6.98627681	ns
SCP2	0.007575051	0.213841977	0.451639519	0.651535207	0.768369469	-6.98047961	ns
LIF	-0.00503541	0.014678149	-0.45046072	0.65238475	0.769060747	-6.98124966	ns
GPR101	-0.00547472	0.112691005	-0.44735991	0.654621646	0.771243836	-6.98329744	ns
WNT9A	-0.00268279	-0.0054908	-0.44716147	0.654764887	0.771243836	-6.98291739	ns
CGN	-0.00356913	0.032727112	-0.44662055	0.655155451	0.771392708	-6.98296221	ns
IGF1R	-0.00201096	-0.00848575	-0.44559246	0.655897965	0.771955687	-6.99040147	ns
DDX4	-0.0049106	0.069556098	-0.44480549	0.656466731	0.772313801	-6.98221747	ns
PMM2	-0.00533624	0.041929373	-0.44379717	0.65719562	0.772649683	-6.98300705	ns
RGS10	-0.00531191	0.050055733	-0.44357575	0.657355523	0.772649683	-6.99874919	ns
HBQ1	-0.00598589	0.055969472	-0.44311896	0.657685869	0.772649683	-6.99976243	ns
NUDT2	0.005347344	0.044004771	0.442946556	0.657810655	0.772649683	-6.991569	ns
FGF6	0.003537286	0.044644467	0.442410223	0.658198747	0.772794669	-6.98327272	ns
CRYGD	-0.00854964	-4.33E-05	-0.44127875	0.659017364	0.773444816	-6.99872617	ns
VPS4B	-0.00833723	0.019127495	-0.43954292	0.660274337	0.774608704	-6.9987249	ns
VSIG10	0.003035475	0.022119706	0.438316523	0.661162978	0.775339717	-6.9992596	ns
MED21	0.004555509	0.072308115	0.437666103	0.661634633	0.775581344	-6.98569061	ns
MAPK13	-0.00374807	-0.01175197	-0.43723521	0.661947062	0.775636204	-6.98466209	ns
CCDC28A	0.002572872	-0.00903071	0.436827426	0.662242747	0.775671409	-6.98725838	ns
RIPK4	-0.00590684	0.172018718	-0.43435344	0.664038038	0.777462338	-6.98712503	ns
AMIGO1	0.004482498	0.071392548	0.433275478	0.664820833	0.778066867	-6.98879305	ns
USO1	-0.00846583	-0.04736892	-0.43185959	0.665849554	0.778877976	-6.99638566	ns
ERVV-1	0.003952124	0.046614404	0.431587595	0.666047366	0.778877976	-6.98831432	ns
DOK2	-0.01137718	-0.04117119	-0.42951794	0.667552386	0.780325447	-6.99767802	ns
HBZ	-0.00914619	0.091404729	-0.4287074	0.668142137	0.780392952	-7.00440604	ns
SMAD2	-0.00594383	0.062553313	-0.42864237	0.668189481	0.780392952	-7.00342508	ns
EPN1	-0.00374972	0.069220627	-0.42833689	0.668411908	0.780392952	-6.99689718	ns
CNTNAP2	-0.00375793	-0.00203018	-0.42759936	0.668948829	0.780707666	-7.00071111	ns
PDE1C	-0.00468941	0.090444885	-0.42667224	0.669624183	0.781046211	-6.98919333	ns
PRR5	0.004038749	0.03743917	0.426466754	0.669773874	0.781046211	-6.99015148	ns

MYOM2	0.00423802	0.048417307	0.425856139	0.670218774	0.781253022	-6.99075462	ns
APOE	0.006117672	0.064077749	0.425029261	0.670821246	0.781643272	-7.00575679	ns
EGFL7	-0.00364727	-0.00495129	-0.42251704	0.672653708	0.783465826	-6.993121	ns
CXCL1	0.008723995	0.068295683	0.419796032	0.674640354	0.785466441	-7.00186978	ns
DPEP2	-0.00232521	-0.00543528	-0.41783377	0.676074524	0.786609614	-7.00230415	ns
ANGPTL3	0.003142471	0.002493272	0.417715499	0.676161004	0.786609614	-7.00273603	ns
ARHGEF5	0.006256319	0.062920942	0.416498815	0.677050963	0.787331267	-6.99653589	ns
CASP2	-0.00606349	0.02706732	-0.41571907	0.677621397	0.787680923	-7.00568681	ns
TADA3	-0.00331501	0.008539283	-0.4151762	0.678018687	0.787829116	-7.00906857	ns
SPRED2	0.003789203	0.055718089	0.414297361	0.678662257	0.788149726	-6.99523206	ns
CALCOCO1	-0.0093972	0.011091652	-0.41406201	0.678834439	0.788149726	-7.01008589	ns
PHLDB2	-0.00771867	0.156974196	-0.41305311	0.679573595	0.788694315	-6.99487206	ns
TXK	0.00630852	0.224679134	0.411576252	0.680655819	0.789458059	-7.00388819	ns
VSTM2B	-0.00353292	0.074227913	-0.41141723	0.680772395	0.789458059	-7.00395318	ns
PDIA5	0.004947159	0.027333797	0.408353894	0.683019698	0.791749709	-6.99678874	ns
DIABLO	-0.00967331	-0.02743327	-0.40786225	0.683380538	0.791853639	-7.00434253	ns
SLAMF7	-0.00513982	-0.04434031	-0.40719748	0.683868634	0.792104884	-7.00916949	ns
SUMF1	-0.00547805	0.130165319	-0.40596854	0.684771493	0.792786085	-6.99862447	ns
IL10	0.007113576	0.056158783	0.405062248	0.685437491	0.792786085	-7.0007333	ns
CD84	0.002729335	-0.00421392	0.404853936	0.685590523	0.792786085	-7.01011462	ns
UBAC1	-0.00308161	0.007699667	-0.40456024	0.685806442	0.792786085	-7.00772525	ns
KIR2DL3	0.006605967	0.005696045	0.404549736	0.685814264	0.792786085	-7.00050007	ns
PDCD1LG2	0.002667205	-0.01851512	0.402598599	0.687249166	0.794130417	-7.00851157	ns
MBL2	0.008669299	-0.09040089	0.401778697	0.687852536	0.794313564	-7.00693477	ns
ADAM23	-0.00300078	-0.03862747	-0.40126117	0.688233369	0.794313564	-7.01686037	ns
DEFB116	-0.00606494	0.196574092	-0.40125726	0.688236411	0.794313564	-7.00272512	ns
ARHGEF10	-0.00757392	0.042159981	-0.40090476	0.688495763	0.794313564	-7.01664647	ns
IFIT1	-0.00421406	0.039074776	-0.40053139	0.688770773	0.794317005	-7.00834259	ns
UBQLN3	0.00383316	0.065592933	0.399790768	0.689316311	0.794474078	-7.00144099	ns
MASP1	-0.00200202	-0.01695541	-0.39937781	0.689620442	0.794474078	-7.00797779	ns
GNPDA2	-0.00292563	0.030758233	-0.39893965	0.689943297	0.794474078	-7.0035336	ns
NPTN	0.006987388	0.178361566	0.398869134	0.689995295	0.794474078	-7.00276636	ns
TRIM40	0.005766588	0.036227833	0.398408027	0.690335069	0.794551992	-7.00319289	ns
CSF2	0.002911675	0.012711188	0.397620909	0.690915292	0.794906482	-7.00230018	ns
NFIC	0.003497649	0.05872239	0.396645248	0.691634533	0.795420574	-7.01833373	ns
ITPRIP	0.003682974	0.085454469	0.395310619	0.692619256	0.79623946	-7.00507734	ns
RGS8	0.00560038	0.149895962	0.393376492	0.694046981	0.797424073	-7.00536649	ns
KLK6	0.00248222	-0.00094985	0.393174708	0.694195888	0.797424073	-7.0156404	ns
TCP11	-0.0046931	0.119664768	-0.39023837	0.696365733	0.799602022	-7.0123847	ns
ATP5PO	-0.00337444	-0.00259435	-0.3888522	0.69739098	0.800464489	-7.00692664	ns
LYZL2	-0.00278414	0.034659601	-0.38767228	0.698264062	0.801151694	-7.00496354	ns
PPM1F	-0.00410915	0.009556691	-0.38699366	0.698766219	0.801412946	-7.02130541	ns
MUC13	-0.00402348	-0.03085114	-0.3853808	0.699960749	0.80246776	-7.00826125	ns
PTPRN2	0.00259687	-0.00382663	0.38449295	0.7006185	0.802619632	-7.01228236	ns
ADGRE5	-0.00246093	0.006426324	-0.38445984	0.70064296	0.802619632	-7.01955996	ns
FKBP5	-0.00790955	-0.06021899	-0.38368644	0.701216203	0.802853895	-7.01534039	ns
CDC37	-0.0069494	0.032377899	-0.38344199	0.701397359	0.802853895	-7.02045863	ns
ARFIP1	0.005319353	0.050202676	0.381866938	0.702565292	0.803875648	-7.02404794	ns
PSTPIP2	-0.00830316	-0.0979737	-0.38009192	0.703882714	0.805005498	-6.99288842	ns
AKT3	0.004683448	0.020927993	0.379793467	0.704104124	0.805005498	-7.00738579	ns
CDK1	-0.00278086	-0.01174814	-0.37647175	0.70657153	0.80751032	-7.00921344	ns
ACADSB	-0.0066134	0.063970581	-0.37600037	0.706921772	0.807594513	-7.02547133	ns
RRM2B	-0.00547206	0.014017249	-0.37464889	0.707926888	0.808426482	-7.01206945	ns
DSG4	0.004042903	0.019924538	0.373692622	0.708638205	0.808701791	-7.01843783	ns
CEND1	0.004367506	0.07432179	0.3729416	0.709197152	0.808701791	-7.01361099	ns
METAP1	0.002416538	0.010479665	0.372936687	0.709200812	0.808701791	-7.01294718	ns
BRME1	-0.00309962	0.022215792	-0.37283595	0.709275783	0.808701791	-7.01365012	ns
GFRAL	-0.00457663	0.029409398	-0.37161016	0.710188368	0.809426243	-7.01182704	ns
CNGB3	0.004114533	0.079514158	0.368107428	0.7127983	0.812060591	-7.0153899	ns
HADH	-0.00482805	0.121435639	-0.36751967	0.713236598	0.812060591	-7.01373682	ns
MINDY1	-0.00802418	-0.0426184	-0.36738881	0.713334046	0.812060591	-7.02764185	ns
KIFBP	-0.00657403	-0.1074722	-0.36699533	0.713627575	0.812078144	-7.02211891	ns
UNG	0.002546827	0.002390642	0.364872661	0.715211457	0.81356348	-7.02855693	ns
PKN3	-0.00546306	0.05040816	-0.36413413	0.715762893	0.813630191	-7.02216412	ns
STXBP3	-0.00339212	0.054212812	-0.363696	0.71609014	0.813630191	-7.01608521	ns
GAD2	0.003351731	0.056749684	0.363674736	0.716106024	0.813630191	-7.01478803	ns

BIRC2	-0.00505022	0.039239979	-0.36307024	0.716557517	0.813826508	-7.01631104	ns
CDNF	0.002368992	-0.00268305	0.361610448	0.71764815	0.814748289	-7.02406737	ns
MNAT1	0.003214867	0.041180447	0.360480454	0.718492858	0.815390263	-7.0234789	ns
CASP8	-0.00482416	0.032287333	-0.35985666	0.71895924	0.815602557	-7.03157012	ns
AKR1B1	-0.00585849	-0.02913306	-0.35864811	0.719863387	0.816000523	-7.01789585	ns
SH3GL3	0.006232309	-0.01478156	0.358304377	0.720120528	0.816000523	-7.02072749	ns
TPM3	0.003115363	0.046215884	0.358267181	0.720148407	0.816000523	-7.0170714	ns
TIMM8A	0.006265229	0.032083689	0.357423497	0.720779655	0.816099948	-7.03199017	ns
ACADM	-0.00502468	0.12953047	-0.35712482	0.721003299	0.816099948	-7.02467475	ns
STAT5B	-0.01066526	-0.04174943	-0.35702955	0.721074612	0.816099948	-7.02570225	ns
CHP1	0.005319651	0.05134312	0.356208915	0.7216891	0.816478951	-7.01966952	ns
PGLYRP2	-0.00217261	-0.03856765	-0.35580193	0.721993814	0.816507334	-7.02478721	ns
APPL2	0.008096252	-0.06227341	0.354931309	0.722645994	0.816928495	-7.02000822	ns
CR2	-0.00352218	0.002131041	-0.35353967	0.723688748	0.81779069	-7.02897153	ns
FGF2	-0.0051085	0.05881199	-0.35216836	0.72471684	0.818635656	-7.02992246	ns
GLYR1	-0.00427629	0.120905003	-0.35142999	0.725270582	0.818944355	-7.03333924	ns
MAP2K6	-0.0076595	-0.04439432	-0.35036701	0.726068089	0.819527955	-7.03055096	ns
KRT6C	0.00263623	0.040945078	0.345431493	0.729774838	0.823393558	-7.02155657	ns
BAMBI	0.002112581	0.002512311	0.34444	0.730520227	0.823843422	-7.02285635	ns
XIAP	0.004039013	0.048017503	0.344150618	0.73073783	0.823843422	-7.02165032	ns
EHD3	0.008057266	-0.09078662	0.342727874	0.731807969	0.824361188	-7.02341348	ns
EP300	0.002040424	0.001158383	0.342460247	0.732009343	0.824361188	-7.02222664	ns
RAD51	-0.00372621	0.045274108	-0.34241405	0.732044027	0.824361188	-7.02978526	ns
RNF4	-0.00455875	0.127070525	-0.3404501	0.733522279	0.825707423	-7.03045127	ns
INPP5J	-0.00283699	0.024664776	-0.33670439	0.736344307	0.828564692	-7.04016606	ns
NEXN	-0.00470307	0.069892856	-0.33560321	0.737174654	0.829179503	-7.03858423	ns
KRT17	0.002609722	-0.00040967	0.334069343	0.738331893	0.830161389	-7.02539033	ns
MRPS16	-0.00196708	0.03521769	-0.33285396	0.739249165	0.830825595	-7.02544795	ns
PCDH17	-0.00245353	0.01419953	-0.33253259	0.739491685	0.830825595	-7.03438202	ns
NAP1L4	-0.00272771	-0.02855715	-0.32933973	0.741903389	0.833214575	-7.03905141	ns
IL6ST	-0.00122119	-0.00855254	-0.32700089	0.743671685	0.8348794	-7.03619386	ns
EDF1	-0.00456642	0.083597385	-0.32601687	0.744415984	0.835393802	-7.04368389	ns
DEFB104A_DE	-0.00360926	0.097146164	-0.32509256	0.745115533	0.835553683	-7.03019673	ns
SPESP1	0.001931524	0.025112008	0.325072292	0.74513075	0.835553683	-7.04320432	ns
NRXN3	0.002345572	0.05584233	0.32411438	0.745855925	0.836045797	-7.02984819	ns
CEACAM1	-0.00142186	-0.00458456	-0.32160576	0.747755737	0.837568644	-7.04211124	ns
GPC5	0.003873517	0.023330055	0.321563013	0.74778817	0.837568644	-7.03709831	ns
IL1RL1	0.003232423	-0.03532132	0.320964315	0.748241776	0.83773685	-7.04280322	ns
IL2RG	0.002287222	0.025331718	0.320215712	0.748809144	0.83773685	-7.04375161	ns
SEMA4C	-0.00187033	0.004560033	-0.31957826	0.74929242	0.83773685	-7.03828647	ns
EGF	-0.00717248	-0.08533551	-0.31934275	0.74947098	0.83773685	-7.03850268	ns
CD177	0.008818457	-0.24156238	0.319303109	0.749501023	0.83773685	-7.04023054	ns
CSF1R	-0.00250629	0.001406766	-0.31909376	0.749659722	0.83773685	-7.04395242	ns
ST8SIA1	0.003191244	0.0905163	0.31812998	0.750390762	0.838232986	-7.03020752	ns
NDUFS6	-0.00537668	0.046530358	-0.31637377	0.751723213	0.839400299	-7.032066	ns
DNLZ	0.005774815	0.235143263	0.31438954	0.753229549	0.840760812	-7.03359549	ns
PFDN4	-0.003451	0.061249285	-0.31352421	0.753886782	0.841172872	-7.03078054	ns
HYAL1	-0.00152542	-0.01284062	-0.31277425	0.754456468	0.84148697	-7.03816545	ns
SMPDL3B	0.004750924	0.164424298	0.309994508	0.756569314	0.84314898	-7.04695083	ns
TNFSF13	-0.00172544	-0.01283804	-0.30991095	0.756632865	0.84314898	-7.04515854	ns
TRIM24	-0.00401547	0.040235476	-0.30967437	0.756812835	0.84314898	-7.03885031	ns
CD207	-0.00250967	-0.00969881	-0.30881912	0.757463401	0.843551918	-7.03361645	ns
TNNI3	-0.00552754	-0.10155824	-0.30833696	0.757830206	0.843638659	-7.03522812	ns
SMPD3	0.003686339	0.115722524	0.306574328	0.759171642	0.844809906	-7.03379213	ns
PGM2	0.003719967	0.097148545	0.304558069	0.760706953	0.846195925	-7.03595329	ns
IL17RA	0.002902358	-0.04241618	0.302596883	0.762201185	0.847296796	-7.04632493	ns
MUC16	-0.0122629	-0.57778733	-0.30249751	0.762276946	0.847296796	-7.04356331	ns
CRH	0.005702849	0.086425125	0.299360506	0.76466914	0.849632378	-7.03618711	ns
SLC4A1	-0.00293315	0.081927052	-0.29818507	0.765565918	0.850305241	-7.05112204	ns
SERPINA4	-0.00139013	-0.0074732	-0.29459242	0.768309283	0.853027798	-7.04260036	ns
SCRN1	-0.00352088	0.043232895	-0.29217452	0.77015714	0.854409909	-7.0491207	ns
OFD1	0.003834175	0.153501516	0.292029415	0.770268102	0.854409909	-7.04566006	ns
PSMA1	-0.00298877	0.049360822	-0.2918152	0.770431949	0.854409909	-7.03948289	ns
OSTN	-0.00247256	0.042508414	-0.28905878	0.772540359	0.856422874	-7.04052206	ns
MANEAL	0.002950894	-0.01158438	0.287346481	0.773850999	0.857550253	-7.03859106	ns
CEACAM18	0.003843	0.137896503	0.28584192	0.775003077	0.858198344	-7.04743573	ns

IFNW1	0.002021616	0.040090482	0.285815151	0.775023641	0.858198344	-7.04024354	ns
SAT1	-0.00285389	0.094941549	-0.28387166	0.776512648	0.859521203	-7.04079332	ns
MYH7B	-0.00247141	0.05545574	-0.28337845	0.77689065	0.859613754	-7.03971559	ns
TMPRSS11B	-0.00439635	0.181872052	-0.28269808	0.777412168	0.859864974	-7.04299199	ns
TMED8	-0.00583342	-0.03805682	-0.28085219	0.778827529	0.860828913	-7.05516403	ns
UBE2Z	-0.00338251	0.096815073	-0.28079268	0.778873284	0.860828913	-7.04131169	ns
GGA1	0.004191192	0.162136785	0.27974812	0.779674597	0.861099205	-7.04314999	ns
DLL4	0.00488877	0.096239804	0.279705056	0.779707636	0.861099205	-7.04289264	ns
PTGR1	0.004561475	0.000851081	0.278059402	0.780970471	0.862167779	-7.05728721	ns
BRD2	0.001910072	0.017556094	0.277297718	0.781555313	0.862487345	-7.04449372	ns
ENOPH1	-0.00298157	0.092678459	-0.27608049	0.782489936	0.863192525	-7.05682247	ns
RPGR	-0.00233394	0.026118205	-0.27522755	0.783145188	0.863589104	-7.0443977	ns
DCC	0.001244327	0.018421443	0.273370099	0.78457247	0.864836396	-7.0449037	ns
HCG22	-0.00291937	0.06826037	-0.27127408	0.786183962	0.866285724	-7.04426603	ns
CD101	-0.00234114	-0.06271614	-0.27065731	0.786658222	0.866481331	-7.05795651	ns
CSPG5	0.002665013	0.057561548	0.270084118	0.787099236	0.866640185	-7.0464545	ns
STK24	-0.00356103	0.03087498	-0.26926259	0.787731303	0.867009199	-7.04600859	ns
INSL3	0.003975186	0.640485637	0.268315177	0.788460296	0.867176481	-7.05968331	ns
MYL6B	-0.00506597	0.267128984	-0.26829327	0.788477246	0.867176481	-7.04626924	ns
CRYBB2	-0.00355125	0.138784028	-0.26690436	0.789546352	0.868025357	-7.06005833	ns
IL18RAP	-0.00993537	0.437480727	-0.26429394	0.791557015	0.869908349	-7.05082549	ns
STOML2	0.004989607	0.116380286	0.263554979	0.792126468	0.870206655	-7.0450993	ns
ARID3A	-0.00211468	0.033694877	-0.26106146	0.794048675	0.871856533	-7.04883427	ns
MRPL24	-0.00202006	0.013004816	-0.26069182	0.794333744	0.871856533	-7.04706139	ns
LMOD1	0.001763088	0.004095823	0.260444919	0.794524053	0.871856533	-7.06278353	ns
GFOD2	0.007156136	0.48734443	0.259797837	0.795023275	0.87207662	-7.04825291	ns
IGHMBP2	0.002925013	0.082171458	0.258458489	0.79605662	0.872882212	-7.04763713	ns
DKKL1	-0.00480007	-0.12792802	-0.25613151	0.797852733	0.874523266	-7.05642644	ns
FMR1	-0.00185284	-0.00868669	-0.25461289	0.799025587	0.875480192	-7.04957736	ns
NCK2	-0.00574555	-0.03250535	-0.25128814	0.801594692	0.877965679	-7.06149771	ns
CRYZL1	-0.00464662	-0.00707406	-0.24949485	0.802981426	0.879154768	-7.05083143	ns
SUSD4	-0.00202547	0.018281077	-0.24695981	0.804942606	0.880971668	-7.06438722	ns
HSBP1	-0.00467269	0.031978279	-0.24274166	0.808208832	0.884214983	-7.05248211	ns
DECR1	-0.00499947	-0.04681159	-0.24184447	0.80890391	0.884374401	-7.06246891	ns
LEO1	-0.00143187	0.00957895	-0.24164863	0.80905563	0.884374401	-7.06747085	ns
TFRC	-0.00200625	0.008120676	-0.24138088	0.809263151	0.884374401	-7.06261599	ns
SERPINA1	-0.00037757	-0.00429912	-0.24025437	0.810136311	0.884997392	-7.05728857	ns
RAB37	-0.00434301	0.193401466	-0.23979719	0.810490736	0.885053459	-7.05365435	ns
SLURP1	0.001772978	0.000671023	0.238501802	0.811495096	0.885818946	-7.06364429	ns
MGLL	0.004500387	-0.09340847	0.237437639	0.812320455	0.886171698	-7.06374443	ns
MFAP3L	-0.00319875	0.15983596	-0.23730266	0.812425218	0.886171698	-7.05404989	ns
CENPJ	-0.00336967	0.10837507	-0.23638457	0.813137439	0.886617372	-7.06026222	ns
SCN2A	0.00223506	0.04412435	0.235965301	0.8134628	0.886641051	-7.0550283	ns
BCL2L1	-0.00467652	-0.02771123	-0.23412364	0.81489209	0.88691253	-7.06821556	ns
VAV3	-0.0047165	0.016715733	-0.23402474	0.814968947	0.88691253	-7.0554812	ns
GSTT2B	0.011221005	-0.51650693	0.233955849	0.815022345	0.88691253	-7.06825457	ns
VPS28	-0.00315873	0.065348707	-0.23394535	0.81503054	0.88691253	-7.06083196	ns
RAD23B	-0.00227002	0.020030052	-0.23368778	0.815230559	0.88691253	-7.0546512	ns
CYP24A1	0.003995828	0.218778828	0.233102446	0.815685029	0.887076456	-7.05382632	ns
CRHR1	0.003079725	0.068512175	0.231682116	0.816788069	0.887514755	-7.05578388	ns
STEAP4	0.003408187	0.136177115	0.231617803	0.816838022	0.887514755	-7.05603775	ns
RHOC	0.005910689	-0.14197442	0.231368358	0.817031765	0.887514755	-7.05848161	ns
VSTM2L	-0.00284348	0.021654526	-0.22964	0.81837464	0.887514755	-7.05625169	ns
TRIM26	0.002567263	0.028640413	0.229092924	0.818799812	0.887514755	-7.05352965	ns
OPHN1	0.005052143	-0.01509244	0.228899689	0.818949902	0.887514755	-7.06941689	ns
KIAA1549L	0.001293956	0.009595429	0.228338945	0.819385808	0.887514755	-7.06211906	ns
FGF20	0.001982447	0.08274584	0.228299726	0.819416342	0.887514755	-7.05458169	ns
MMUT	0.002321652	0.052119822	0.228268383	0.819440707	0.887514755	-7.05493381	ns
CST6	-0.00232406	-0.00076379	-0.2281319	0.819546835	0.887514755	-7.05023694	ns
CEACAM20	-0.00202903	0.044162581	-0.22806611	0.819597903	0.887514755	-7.0621809	ns
ARL2BP	0.001868906	0.02596605	0.227889333	0.819735375	0.887514755	-7.05501967	ns
NCS1	0.001341449	-0.0294056	0.226997158	0.820428942	0.887936438	-7.07169403	ns
DUSP3	0.004183724	0.133040328	0.225196576	0.821829407	0.88896485	-7.05682833	ns
PRC1	0.002075531	0.072129964	0.224992549	0.821988046	0.88896485	-7.06953632	ns
MST1	-0.00295656	-0.17445556	-0.22246597	0.823954149	0.890761242	-7.06198322	ns
LAMP1	0.001242576	0.021496303	0.221113418	0.825007125	0.891453884	-7.05701363	ns

KLRC1	0.002682352	0.097034294	0.220858725	0.825205427	0.891453884	-7.05845521	ns
DEFB118	0.002572394	0.098682971	0.218627693	0.826943038	0.893000618	-7.05894211	ns
CA13	-0.00492546	-0.11725211	-0.21800793	0.827425849	0.893191674	-7.06454039	ns
LILRB1	-0.00129707	-0.02574908	-0.21751523	0.827809697	0.893275801	-7.07278033	ns
IL15	-0.0015075	-0.02098871	-0.2162956	0.828760217	0.893971125	-7.06828203	ns
CACNA1H	0.002497497	0.101908115	0.215070854	0.829714995	0.894670526	-7.0578392	ns
GALNT10	0.001260385	-0.01727252	0.21315376	0.831209874	0.895724404	-7.06644269	ns
SPINK8	0.001627634	0.036054929	0.213030748	0.831305868	0.895724404	-7.05827288	ns
KIF20B	0.003294037	0.062821701	0.212491858	0.831726201	0.895846738	-7.06025569	ns
PPIF	-0.00262617	0.089731501	-0.21170477	0.832340157	0.896177455	-7.07302256	ns
SCG2	-0.00139238	0.003569741	-0.21123355	0.832707821	0.896242845	-7.07288801	ns
LSM1	0.002467359	0.064676575	0.209753073	0.833863241	0.89703938	-7.05992162	ns
NXPE4	-0.00143628	0.033016579	-0.20949804	0.834062301	0.89703938	-7.05901409	ns
NOTCH1	-0.00069202	0.001643544	-0.20908308	0.834386128	0.897057251	-7.07456692	ns
TREML1	-0.00219807	0.037420251	-0.20794217	0.835276855	0.897684364	-7.07304701	ns
C2CD2L	0.001890029	0.003650349	0.205111995	0.837487402	0.899516436	-7.05984167	ns
CCT5	-0.00204098	0.05462942	-0.20497086	0.837597668	0.899516436	-7.06090647	ns
DNAJA1	0.001859054	0.037094083	0.203027818	0.839116039	0.900624033	-7.0591225	ns
EDAR	-0.00406353	0.052893438	-0.20273915	0.839341608	0.900624033	-7.07110324	ns
PPT1	0.003194375	0.199648092	0.202467075	0.839554321	0.900624033	-7.06165721	ns
GSAP	0.003737195	0.246874791	0.196329715	0.844354757	0.905441017	-7.06262843	ns
KIR2DL2	-0.00330949	-0.03595269	-0.19547397	0.845024484	0.905826539	-7.07570064	ns
PRDX5	-0.00288471	-0.12997699	-0.19494246	0.845440541	0.905916224	-7.0779549	ns
LCN15	0.002345506	0.050378763	0.194574467	0.845728639	0.905916224	-7.0776699	ns
ATXN10	0.002995771	-0.02471162	0.193839576	0.846304111	0.906200221	-7.06335316	ns
CCDC134	-0.00185871	0.025800602	-0.19343221	0.846623037	0.90620941	-7.07609501	ns
SUGT1	0.003366655	-0.02594051	0.192645311	0.847239357	0.906491771	-7.06334009	ns
CASP3	0.003633613	-0.05198916	0.191780029	0.847917026	0.906491771	-7.0780119	ns
SLA2	-0.00400756	-0.03263214	-0.19128826	0.848302257	0.906491771	-7.07829969	ns
HRG	-0.00122334	-0.0149451	-0.19114321	0.848415914	0.906491771	-7.07366424	ns
KITLG	0.001497887	-0.01029371	0.190839242	0.848654073	0.906491771	-7.07121064	ns
NDST1	-0.00242339	0.123615671	-0.19071743	0.848749487	0.906491771	-7.07661297	ns
AHSA1	-0.00255963	0.104091082	-0.1890244	0.850076174	0.907576756	-7.07693228	ns
TRIM21	-0.00311344	0.002997749	-0.18834545	0.850608353	0.907813009	-7.07389889	ns
ASRGL1	0.00384164	0.018850975	0.186930757	0.851717439	0.908664568	-7.06439009	ns
RABEPK	-0.00238378	0.093189341	-0.18523046	0.853050775	0.909754661	-7.06473143	ns
DARS1	0.002888173	-0.04285026	0.181547127	0.855940561	0.912503264	-7.07082002	ns
CD40LG	-0.00304756	-0.04097146	-0.18089111	0.856455405	0.912718899	-7.08057781	ns
PECR	0.001029271	0.021820899	0.178997073	0.857942405	0.913970019	-7.06583171	ns
NPTX2	-0.0011598	-0.01579928	-0.17647638	0.859922014	0.915744815	-7.07981443	ns
CYTH3	0.002605501	0.122892007	0.174606243	0.861391396	0.916975164	-7.06445202	ns
CTLA4	0.001028494	-0.0199997	0.173235439	0.862468633	0.917787321	-7.08037734	ns
DYNLT3	-0.00151616	0.022529993	-0.1727206	0.862873309	0.917883447	-7.07986148	ns
EPO	-0.00275365	0.072446807	-0.16910586	0.865715604	0.920571581	-7.07731438	ns
H2AP	0.001737845	0.062393304	0.166283201	0.867936323	0.92258215	-7.06586087	ns
TPSD1	-0.001222	0.031378876	-0.16589941	0.86823827	0.92258215	-7.08280379	ns
PDCD6	0.00251544	-0.18521749	0.165371392	0.868653853	0.92258635	-7.07571596	ns
PCARE	-0.00278447	0.190017493	-0.16507483	0.868887297	0.92258635	-7.06914597	ns
NECAP2	-0.00220703	0.090447479	-0.16469015	0.869190085	0.92258635	-7.06733979	ns
CRIM1	0.000687614	-0.01135655	0.163746654	0.869932735	0.922845173	-7.07954225	ns
TNN	0.001415738	0.013738185	0.163577347	0.870066014	0.922845173	-7.08199297	ns
TRAF2	0.002478588	0.053272983	0.162201213	0.87114954	0.923658917	-7.07845047	ns
PRRT3	0.001195367	0.015882938	0.161666127	0.871570947	0.923770296	-7.06876348	ns
NMI	-0.002319	0.040922428	-0.16064574	0.872374534	0.924128588	-7.07942471	ns
GSN	0.000588227	0.004596979	0.160433177	0.872541958	0.924128588	-7.08003786	ns
CD248	-0.00125059	0.007250087	-0.15748648	0.874863515	0.926251437	-7.08296422	ns
IDS	0.000294448	-0.00462316	0.155786847	0.876203063	0.927333434	-7.08477572	ns
TPMT	0.002403096	0.073347866	0.154198263	0.87745548	0.928322465	-7.06996193	ns
PDCL2	-0.0016404	0.067610293	-0.15346253	0.878035597	0.928599762	-7.06789643	ns
CMIP	0.0025477	0.014338264	0.15240149	0.878872272	0.928748726	-7.08314251	ns
CLEC10A	-0.00116934	-0.01148855	-0.15238307	0.878886822	0.928748726	-7.07691808	ns
DDA1	0.001242079	0.034767663	0.152073959	0.879130644	0.928748726	-7.06932424	ns
LAG3	0.001275429	0.011541399	0.148404656	0.882025382	0.931231114	-7.07806887	ns
EPPK1	-0.00216525	0.027879247	-0.14828399	0.88212063	0.931231114	-7.07082315	ns
VCAM1	0.000839159	0.000291885	0.147882867	0.882437155	0.931231114	-7.07957848	ns
TBR1	-0.00143393	0.082587646	-0.14715534	0.883011372	0.931340921	-7.07126005	ns

CCDC50	-0.00227395	0.154310522	-0.14653776	0.883498786	0.931340921	-7.08580831	ns
CHEK2	-0.00102116	-0.00418134	-0.14637869	0.883624405	0.931340921	-7.0717984	ns
ENOX2	0.000813731	0.017814115	0.146134694	0.883817018	0.931340921	-7.07020373	ns
GIPC3	-0.00287843	-0.09430871	-0.14549292	0.884323603	0.931428678	-7.08382594	ns
DCTN2	-0.00284978	0.080828497	-0.14521349	0.884544231	0.931428678	-7.0785342	ns
RICTOR	0.001414255	0.077930817	0.144817071	0.884857244	0.931428678	-7.07004901	ns
PLCB2	-0.00282747	-0.04359001	-0.1442985	0.885266638	0.931445768	-7.08509807	ns
APRT	-0.00150162	-0.04580861	-0.14398849	0.885511456	0.931445768	-7.07815362	ns
TARM1	-0.00223218	0.174656274	-0.14279877	0.886451012	0.932098291	-7.07188733	ns
AGBL2	0.002629544	-0.08226005	0.142020295	0.887065872	0.932370333	-7.07266171	ns
UBE2B	-0.00082354	0.012489597	-0.14115596	0.887748573	0.932370333	-7.08478209	ns
TIMP2	-0.00071005	-0.00708621	-0.14102606	0.887851192	0.932370333	-7.08581004	ns
FH	-0.0024034	0.175841777	-0.14085427	0.887986951	0.932370333	-7.0728255	ns
CPVL	0.001214998	-0.04548448	0.137301588	0.8907942	0.934874999	-7.08672105	ns
SOD3	0.001658864	0.093856886	0.136669596	0.89129374	0.934874999	-7.08524562	ns
ADRA2A	-0.00201552	0.188818132	-0.13662015	0.891332876	0.934874999	-7.07153963	ns
ATP5F1D	-0.00144974	0.04692515	-0.13285207	0.894312153	0.937663012	-7.07204384	ns
IL12RB2	0.001113927	0.076340952	0.130903138	0.895853684	0.938942124	-7.071954	ns
BCAT1	0.000776605	0.004959264	0.12940615	0.897037962	0.939825749	-7.08737077	ns
CTAG1A_CTAG	0.001311227	0.01151326	0.129023858	0.897340475	0.939825749	-7.07820662	ns
HRAS	-0.00247223	0.270112446	-0.12759569	0.898470625	0.940672006	-7.07902224	ns
EGFR	-0.00042558	0.012870119	-0.12459894	0.900842686	0.942583072	-7.08856847	ns
HSPB1	-0.00186073	-0.14338851	-0.12417301	0.901179953	0.942583072	-7.07388264	ns
CBX2	-0.00098946	0.065873713	-0.1240664	0.901264362	0.942583072	-7.07316469	ns
CLIC5	0.000748007	0.025628319	0.120605338	0.904005244	0.944991231	-7.07479022	ns
NEO1	0.00053746	0.00300611	0.12034147	0.904214212	0.944991231	-7.08928066	ns
KLK12	-0.00356832	-0.54089191	-0.11986525	0.904591497	0.945047289	-7.07463439	ns
PTEN	-0.00073312	-0.00554402	-0.11564258	0.90793736	0.94807646	-7.07537053	ns
EPGN	0.001111054	0.100676841	0.115386394	0.908140362	0.94807646	-7.0880655	ns
PMVK	-0.00273293	-0.30859416	-0.11446988	0.908866851	0.948495785	-7.07407338	ns
EDNRB	-0.00104246	0.062912895	-0.11347762	0.909653424	0.948673771	-7.0740686	ns
COMMD9	-0.00089081	0.046024029	-0.11312179	0.909935467	0.948673771	-7.08832251	ns
DEFB103A_DE	-0.00093958	0.035581048	-0.11289429	0.910115887	0.948673771	-7.06957457	ns
SOWAHA	0.001115793	0.065157369	0.11261542	0.910336955	0.948673771	-7.07571554	ns
PLCB1	-0.00102002	0.069120084	-0.11147322	0.911242591	0.94927876	-7.07429249	ns
MAPKAPK2	0.001617664	0.020175084	0.10882726	0.913340932	0.950725527	-7.08213028	ns
OSBPL2	0.000838997	0.06493277	0.108664551	0.913469966	0.950725527	-7.08957504	ns
MRPL52	-0.00055553	-0.00228687	-0.10849038	0.913608161	0.950725527	-7.07229528	ns
SESTD1	0.001494084	0.061696307	0.107884684	0.914088603	0.950886612	-7.07623164	ns
GBP2	-0.00136836	-0.01985941	-0.10725368	0.914589157	0.951068497	-7.07649646	ns
FGD3	0.001721498	0.024898097	0.106164664	0.915453078	0.951627977	-7.09087554	ns
NEB	-0.00079752	0.034835709	-0.10573206	0.915796354	0.951646034	-7.07525682	ns
NOS2	-0.00083875	0.013017128	-0.10383139	0.917304542	0.95287416	-7.07545461	ns
RNF5	-0.00150023	-0.09088716	-0.10268476	0.918214532	0.953426098	-7.07650592	ns
GRAP2	-0.00284401	-0.10292451	-0.10233904	0.918488909	0.953426098	-7.08409619	ns
CRKL	-0.00236038	-0.08170865	-0.10172381	0.918977229	0.953593998	-7.08600784	ns
EDEM2	-0.00073864	0.051127624	-0.09976876	0.920529242	0.954571462	-7.07586535	ns
IL24	-0.00103854	0.030004918	-0.0997136	0.920573027	0.954571462	-7.07727139	ns
HTRA2	0.001261381	0.025624336	0.095153292	0.924194304	0.957658591	-7.08898317	ns
DNM1	0.002184268	-0.09300698	0.095138397	0.924206133	0.957658591	-7.08984388	ns
BTN1A1	0.000744845	0.047315714	0.093974664	0.925130504	0.958276365	-7.08930209	ns
TYMP	-0.00134104	-0.02481184	-0.09355524	0.925463698	0.958281559	-7.08237511	ns
ADCYAP1R1	-0.00067754	0.027351203	-0.09229623	0.926463916	0.958977183	-7.07753888	ns
RAB33A	-0.00121333	0.04945868	-0.08925757	0.928878405	0.961135699	-7.07685197	ns
ERBB3	-0.00035803	0.004432797	-0.0866875	0.930921053	0.962908068	-7.08415614	ns
MEGF11	0.000570725	0.039142756	0.085831427	0.931601567	0.9631244	-7.07714985	ns
MN1	-0.00100935	0.082549626	-0.08559454	0.931789873	0.9631244	-7.0783751	ns
SLK	-0.00137477	0.186272559	-0.08480585	0.932416855	0.963431429	-7.08444021	ns
YES1	-0.00204993	-0.03419532	-0.08257828	0.934187949	0.96491999	-7.08561989	ns
CALY	-0.00060638	0.022474385	-0.08131764	0.935190426	0.965339796	-7.0775245	ns
HK2	-0.00046415	-0.00680668	-0.08123571	0.935255577	0.965339796	-7.07916139	ns
PTPRH	0.000771961	0.034601966	0.080397066	0.935922501	0.96568682	-7.09112904	ns
BNIP2	0.000546074	0.009900216	0.079715526	0.936464541	0.96580648	-7.09253182	ns
DRG2	0.000974739	0.044038546	0.079143324	0.936919659	0.96580648	-7.08589577	ns
PAK4	-0.00103946	0.074696987	-0.07889306	0.937118734	0.96580648	-7.07892058	ns
VTA1	-0.00121413	0.019069895	-0.07858781	0.937361494	0.96580648	-7.0935594	ns

CRX	-0.00183673	0.339559902	-0.07782938	0.937964813	0.966087215	-7.07943045	ns
SPP1	0.000732484	-0.01950902	0.076657523	0.938896987	0.966706348	-7.09236657	ns
PNPT1	0.002000281	0.41178701	0.075684747	0.939670914	0.967162167	-7.07936426	ns
CDC123	-0.00095649	0.071682469	-0.072724	0.942026719	0.969156897	-7.07696455	ns
BHLHE40	0.001031449	0.084472553	0.072414822	0.94227275	0.969156897	-7.08007357	ns
MUCL3	0.000641328	0.075296659	0.070697575	0.943639392	0.969770165	-7.07710886	ns
GART	0.000382427	0.018293948	0.070436259	0.94384734	0.969770165	-7.09400937	ns
IMPA1	0.000922742	0.039305942	0.070278201	0.943973153	0.969770165	-7.08599703	ns
IL9	0.000793471	0.088876583	0.069873458	0.944295294	0.969770165	-7.08558725	ns
PSCA	-0.00358042	-1.29913264	-0.0692634	0.944780854	0.969770165	-7.09195661	ns
SLC44A4	0.000952654	0.144008815	0.069161875	0.944861685	0.969770165	-7.07963792	ns
DCBLD2	-0.00047632	-0.02543758	-0.06629527	0.94714361	0.971770675	-7.09129711	ns
CBLIF	0.000946504	0.023234483	0.063499609	0.949369501	0.973712309	-7.08668475	ns
ARNTL	-0.00065294	0.106952634	-0.06176294	0.950752437	0.974788314	-7.07891384	ns
SMPDL3A	-0.0008537	-0.0438468	-0.06084063	0.951486925	0.974932868	-7.09042558	ns
ENO1	-0.00101479	-0.04632522	-0.0596618	0.952425777	0.974932868	-7.08933537	ns
MAEA	-0.0005031	0.026024076	-0.05951291	0.952544377	0.974932868	-7.08001023	ns
S100A3	0.000518586	0.060646191	0.059100394	0.952872909	0.974932868	-7.09294328	ns
NGF	-0.0001513	-0.00377047	-0.05877605	0.953131257	0.974932868	-7.08673421	ns
PLA2G4A	0.001113623	-0.02162898	0.058321699	0.95349314	0.974932868	-7.09116954	ns
IL17F	-0.00098328	0.155523911	-0.05824426	0.953554838	0.974932868	-7.08052416	ns
NEK7	0.000873993	0.112703258	0.058232147	0.953564477	0.974932868	-7.08632786	ns
ATF4	0.000401328	0.019725045	0.05760879	0.954061014	0.975099112	-7.07794277	ns
CD1C	0.000279515	-0.00175217	0.055424933	0.95580065	0.976535303	-7.09195425	ns
GATA3	0.000287299	-0.00430468	0.054250543	0.956736274	0.977149325	-7.0781293	ns
KRT8	0.000653344	0.035507696	0.053636748	0.957225288	0.977306938	-7.08124898	ns
TPT1	0.000737509	0.091322776	0.051794316	0.958693269	0.978463595	-7.0808766	ns
MANF	-0.0013277	-0.25297915	-0.05091321	0.959395342	0.978791374	-7.09157146	ns
CTSC	-0.00045397	0.006153173	-0.05054992	0.95968483	0.978791374	-7.08785396	ns
RNF31	-0.00036116	0.023818947	-0.04901717	0.960906261	0.979608166	-7.07926985	ns
EPB41L5	0.000625855	0.057211143	0.048702983	0.961156642	0.979608166	-7.08083535	ns
TYRO3	-0.00028675	-0.01199441	-0.04698403	0.962526568	0.980466644	-7.08820117	ns
PPY	-0.0010222	0.035545384	-0.04680343	0.962670503	0.980466644	-7.09183814	ns
ACSL1	0.000339506	0.04898679	0.045521734	0.963692056	0.981164855	-7.07977907	ns
TNPO1	0.000321388	0.009632085	0.04472832	0.964324445	0.981466497	-7.08701834	ns
SLAMF1	-0.000561	-0.07847509	-0.04395534	0.964940587	0.981751398	-7.08124932	ns
PAPPA	-0.00042527	-0.02010484	-0.04297044	0.965725662	0.981765724	-7.08129183	ns
SAP18	0.00062727	0.092825761	0.042703692	0.965938295	0.981765724	-7.08710625	ns
SPINT3	-0.00125953	0.982776912	-0.04267228	0.965963331	0.981765724	-7.09343491	ns
C1GALT1C1	0.000263961	0.008485413	0.040591519	0.967622084	0.983109425	-7.07998989	ns
REPS1	-0.00030766	0.031770094	-0.03906501	0.968839079	0.984003517	-7.07970514	ns
SHH	-0.00033705	0.006178161	-0.03857082	0.969233081	0.984061404	-7.07972419	ns
PDGFRB	-0.00027836	-0.0410986	-0.03685902	0.970597892	0.985104569	-7.09560445	ns
LYAR	0.000419385	0.053242901	0.035408343	0.971754602	0.985755189	-7.0811464	ns
TBCB	0.000770421	-0.11186307	0.035208297	0.971914106	0.985755189	-7.08783422	ns
RUVBL1	0.000776078	0.285223451	0.034152336	0.972756128	0.98626663	-7.08118979	ns
ITGA2	-0.0002224	0.018631455	-0.0328887	0.973763779	0.986859787	-7.08202787	ns
SCN3A	0.000242024	0.037731567	0.032532127	0.974048126	0.986859787	-7.08148767	ns
BCL7B	-0.00029677	0.057095201	-0.03158296	0.974805039	0.986859787	-7.09418263	ns
NT5C	0.000471461	0.006915487	0.031483917	0.974884024	0.986859787	-7.09519561	ns
ELOB	-0.00023815	0.021645314	-0.03040103	0.975747627	0.986859787	-7.08034913	ns
WASF3	-0.00054597	-0.02752928	-0.03029583	0.975831524	0.986859787	-7.08155554	ns
AKT2	-0.00075642	-0.03975172	-0.02975337	0.976264129	0.986859787	-7.09603381	ns
CEACAM3	-0.00029379	0.01329305	-0.02938556	0.976557479	0.986859787	-7.08134019	ns
LRTM1	0.000245889	0.042338354	0.029304057	0.976622478	0.986859787	-7.08158679	ns
TEX101	0.000288222	0.099581517	0.029180754	0.976720816	0.986859787	-7.08159037	ns
IL36A	-0.00032175	0.067156102	-0.02844779	0.977305383	0.987108861	-7.08006107	ns
TPPP2	-0.00043107	0.081118914	-0.02708077	0.978395663	0.987868374	-7.08764768	ns
TBL1X	0.000481207	-0.04315182	0.025953924	0.979294434	0.988434064	-7.08143449	ns
PDIA2	0.000180352	0.033149884	0.021862613	0.982557839	0.991385241	-7.07935339	ns
NARS1	-0.00019117	-0.05037414	-0.02000806	0.984037198	0.992534928	-7.09627467	ns
PRKAG3	-0.00012437	0.025996868	-0.01898648	0.984852141	0.992712462	-7.0818342	ns
THAP12	0.000350161	0.18956309	0.018850664	0.984960488	0.992712462	-7.0778704	ns
RAC3	-0.00025318	0.160196769	-0.0185089	0.985233122	0.992712462	-7.0818431	ns
PSMC3	-0.00010575	0.012868642	-0.01706404	0.986385752	0.993531009	-7.07944614	ns
ENPP2	9.0744968675	0.021937929	0.016050079	0.987194646	0.993982905	-7.09615128	ns

CD226	-0.00013011	0.018159776	-0.01564826	0.987515208	0.993982905	-7.08034134	ns
SH3BGRL2	-0.00018254	0.113726286	-0.01468972	0.988279915	0.994409839	-7.08257043	ns
AKR1B10	-0.00016798	0.019895561	-0.01396623	0.988857107	0.994607191	-7.08191635	ns
DAPP1	0.00035727	-0.27996901	0.013589964	0.989157288	0.994607191	-7.09142493	ns
ITGB1BP2	-0.00029718	-0.09242788	-0.0123934	0.990111914	0.995224368	-7.08236215	ns
NAA10	0.000121365	-0.00064846	0.011884909	0.990517595	0.99527214	-7.08073793	ns
BRAP	-0.00025303	-0.07880488	-0.01147938	0.990841134	0.99527214	-7.08955563	ns
ICAM3	-5.86E-05	-0.01010071	-0.01069428	0.991467501	0.995558839	-7.09622242	ns
ADAMTS1	-5.23E-05	-0.00245939	-0.00835831	0.993331223	0.996703413	-7.08042824	ns
PBLD	-9.21E-05	0.022806931	-0.00833678	0.993348402	0.996703413	-7.0819768	ns
IGFBP7	4.6194056785	-0.01732856	0.007757623	0.993810474	0.996703413	-7.09624933	ns
BCL7A	-9.47E-05	0.052545431	-0.00747032	0.994039706	0.996703413	-7.07956302	ns
IL1R1	-3.30E-05	-0.01258122	-0.00712645	0.994314055	0.996703413	-7.09069461	ns
GPR15L	-6.68E-05	-0.06430246	-0.00550806	0.995605299	0.997655276	-7.09432422	ns
ISM2	2.7987201173	0.006836245	0.003272635	0.997388862	0.999099649	-7.09527716	ns
AGR3	-3.37E-05	0.008485074	-0.00260714	0.997919838	0.999288727	-7.08091329	ns
HPGDS	1.6601369132	0.012222108	0.00209735	0.998326586	0.999353319	-7.0817668	ns
RPA2	-7.66E-06	0.022340899	-0.00123076	0.999018009	0.999702737	-7.08046218	ns
TYRP1	-3.68E-06	-0.04533902	-0.00029764	0.99976252	0.999800741	-7.08046289	ns
TNFRSF21	-1.38E-06	-0.00547418	-0.00024974	0.999800741	0.999800741	-7.09662684	ns

Health-consicous vs Sweet-tooth

	logFC	AveExpr	t	P.Value	adj.P.Val	B	protein_type
CFH	-0.09397966	-0.04566867	-16.1634056	2.9367184728	8.5752179408	121.5526319	ns
LEP	-0.35882666	-0.17409801	-15.7261954	2.7092842372	3.9555549863	114.8007534	down
SERPINF1	-0.08677468	-0.03790164	-14.269743	6.6704551180	6.4925763148	93.38929077	ns
HGF	-0.11998452	-0.04313962	-14.2415434	9.8177174269	7.1669337217	93.00755069	ns
RARRES2	-0.16668028	-0.0752127	-14.0820383	9.2870216015	5.4236206153	90.78334709	ns
APCS	-0.11375804	-0.08561453	-13.716694	1.4379768904	6.9981541999	85.79394951	ns
TNFRSF1A	-0.08712003	-0.02483846	-13.6603332	3.0911189037	1.1416051802	85.03683191	ns
IL1RN	-0.18662727	-0.00163616	-13.65964	3.1276854252	1.1416051802	85.02517927	ns
FSTL3	-0.09611796	-0.0245828	-13.4667553	4.2063760520	1.3647353413	82.45431684	ns
CFI	-0.06399492	-0.0294017	-13.4494888	5.2650610434	1.5373978246	82.23207826	ns
INHBC	-0.14162588	-0.10462579	-13.4080919	9.1874997595	2.4388635725	81.68149838	ns
FABP4	-0.18421798	-0.0562609	-13.3838427	1.2628404207	3.0729116905	81.36667903	ns
ADM	-0.09031473	-0.05568744	-13.3578998	1.7822749323	4.0032636940	81.02583918	ns
IL18R1	-0.10605834	-0.03755503	-13.3323925	2.4990341112	5.2122711462	80.6915249	ns
RELT	-0.08116719	-0.03067685	-13.3246015	2.7857119510	5.4228525980	80.58435599	ns
GFRA1	-0.08174504	-0.0221911	-13.2118715	1.2273681243	2.2399468269	79.11739546	ns
GGH	-0.11031756	-0.04112374	-13.1207329	4.1095849388	7.0588164832	77.92335686	ns
CST3	-0.08172858	-0.04064463	-13.0024954	1.8917268624	3.0688013546	76.41312978	ns
CSF1	-0.07815317	-0.0134698	-12.9089821	6.2977617667	9.6786654519	75.22365523	ns
BPIFB2	-0.18113721	0.05996641	-12.4114864	3.3315487470	4.8640611707	69.02453044	ns
STC1	-0.12433762	-0.02194037	-12.3193618	1.0355012971	1.4398398989	67.90346501	ns
EPHA1	-0.09676917	-0.0608638	-12.3025159	1.2768088164	1.6946735200	67.69732196	ns
ASGR1	-0.08774745	-0.04427822	-12.293124	1.4368455233	1.8241690991	67.58135467	ns
CD99L2	-0.0696962	-0.01799669	-12.2704056	1.8929949048	2.3031438009	67.30824647	ns
TGFBR2	-0.08433502	-0.01406392	-12.24587	2.5507253612	2.9660983587	67.0132767	ns
NTRK3	0.063418734	0.014814134	12.24288267	2.6410464837	2.9660983587	66.97826177	ns
NHLRC3	-0.08548313	-0.04392814	-12.2062628	4.2008147539	4.5431033635	66.52456576	ns
SEMA3F	-0.07613625	-0.02214061	-12.1963912	4.6749792013	4.8753354528	66.41549173	ns
COLEC12	-0.07191936	-0.03369293	-12.1571547	7.5247042097	7.5765987215	65.94525821	ns
COL6A3	-0.09219235	-0.03672984	-12.1046828	1.4135234028	1.3758294454	65.32093767	ns
CDHR5	-0.11335736	-0.06542682	-11.951658	8.9038819606	8.3868823629	63.50441306	ns
CTSO	-0.08487085	-0.0237383	-11.9151032	1.3693572509	1.2495384915	63.07749961	ns
NPC2	-0.07765948	-0.03478036	-11.8100802	4.7791063046	4.2287849726	61.84549383	ns
CD83	-0.08955644	-0.0265991	-11.7767707	7.0537687412	6.0579425660	61.45979486	ns
LGALS9	-0.08949725	-0.04005442	-11.7556257	9.0468748955	7.5476784842	61.21458839	ns
BGLAP	-0.21843526	-0.05549405	-11.7513301	9.4828765980	7.6916665739	61.16658518	down
VWC2	-0.10487118	-0.04137541	-11.7154429	1.4518435325	1.1457792202	60.74834378	ns
INHBB	-0.12646698	-0.02699148	-11.5703877	7.8469004537	6.0297235065	59.08331531	ns
RNASE6	-0.06912997	-0.02731617	-11.470919	2.4549048807	1.8380313465	57.95523765	ns
A1BG	-0.04334657	-0.02054699	-11.4657728	2.5972545232	1.8959958019	57.89802743	ns
CFB	-0.08786291	-0.0487602	-11.4277855	4.0334215296	2.8725831382	57.46638581	ns
F9	-0.05034282	-0.02595323	-11.3567013	9.0338466504	6.2806743379	56.66925802	ns
TNFRSF4	-0.09352427	-0.01322573	-11.3349836	1.1519752234	7.8227154710	56.42747762	ns

AMBP	-0.05153812	-0.03425718	-11.2967357	1.7756716493	1.1784002763	56.00013711	ns
IGFBP4	-0.10211431	-0.04467782	-11.2923151	1.8757648303	1.2171629565	55.94924772	ns
GHR	-0.07568189	-0.02835094	-11.1684349	7.5440575138	4.7888365088	54.57704047	ns
FAM20A	-0.06987016	-0.04298675	-11.1581473	8.4137745209	5.2272811917	54.4651605	ns
RTN4R	-0.09240942	-0.03712188	-11.0992295	1.6199963839	9.8549780023	53.8181079	ns
HAVCR2	-0.08694432	-0.03890064	-11.0388983	3.1666955704	1.8870920542	53.15865057	ns
FCAMR	-0.17759352	-0.02923245	-11.006787	4.5035626072	2.6300805626	52.80985788	ns
TGFA	-0.11648496	0.008263632	-11.0018498	4.7657214630	2.7286091513	52.75550181	ns
CLMP	-0.05529708	-0.01790542	-10.9552418	7.9297026726	4.4528330392	52.25110394	ns
SHISA5	-0.05573694	-0.02141815	-10.9504994	8.3573413303	4.6044220159	52.20004912	ns
MPO	-0.11940903	-0.00808592	-10.9442587	8.9640328904	4.8472177852	52.13211395	ns
FURIN	-0.09319228	-0.04240017	-10.9128429	1.2625273373	6.7028724094	51.79371359	ns
ORM1	-0.0482866	-0.00760453	-10.8948973	1.5355250521	8.0066663433	51.60044925	ns
CD302	-0.07153369	-0.0288168	-10.8782666	1.8443470054	9.4482337825	51.42146783	ns
CCL22	-0.1295632	-0.04447282	-10.7441113	7.8870354693	3.9707144086	49.99000333	ns
EFNA4	-0.06835558	-0.02651292	-10.7182321	1.0406416069	5.1061680350	49.71562403	ns
SSC4D	-0.30645676	0.095505149	-10.7174103	1.0492126099	5.1061680350	49.70698181	down
CLEC4D	-0.15413014	-0.05301058	-10.7057876	1.1874671262	5.6842688668	49.58386113	ns
CFD	-0.04431208	-0.01534626	-10.6293891	2.6798753986	1.2621348651	48.77959425	ns
TNFRSF1B	-0.08452213	-0.01985275	-10.6162019	3.0886481784	1.4315639176	48.6411336	ns
CD300E	-0.09856563	-0.02326622	-10.6060252	3.4369434484	1.5681054483	48.53422028	ns
LAIR1	-0.08980622	-0.03529584	-10.5647098	5.3358680473	2.3970361074	48.10315943	ns
RNASE4	-0.0610931	-0.03006874	-10.5568652	5.8277482467	2.5783371031	48.02056309	ns
GUSB	-0.15064713	-0.04504515	-10.5219647	8.3704834651	3.6480315997	47.6578673	ns
PALM2	-0.0868967	-0.01341872	-10.3960763	3.1272234291	1.3428665313	46.35739669	ns
ADAMTS15	-0.1115731	-0.04762613	-10.3786914	3.7569501030	1.5898977247	46.17937949	ns
SERPIND1	-0.06525046	-0.03243131	-10.3639628	4.3697718328	1.8228191074	46.02832752	ns
LAMP3	-0.12723803	-0.04649444	-10.3507882	5.0071216071	2.0454302627	45.89327968	ns
TNFRSF14	-0.06902967	-0.0197507	-10.3502681	5.0435266752	2.0454302627	45.88845458	ns
PON3	0.068166087	0.001823046	10.32476615	6.5640063982	2.6256025593	45.62786202	ns
SLC39A5	-0.12993665	-0.06035542	-10.3193868	6.9456370521	2.7407108367	45.57268446	ns
RNASE1	-0.05277052	-0.01455021	-10.3131898	7.3932362040	2.8678115529	45.50984896	ns
IL6	-0.17973687	-0.00484653	-10.3124772	7.4641670556	2.8678115529	45.50281656	ns
KRT18	-0.19581227	-0.00335384	-10.3051296	8.0528923886	3.0538241266	45.42800343	ns
NECTIN2	-0.06522556	-0.01902468	-10.2875306	9.6583686332	3.6156969755	45.24895391	ns
LILRA5	-0.07970597	-0.05301764	-10.2581412	1.3071790958	4.8315986835	44.95074387	ns
IL10RB	-0.06664475	-0.02621208	-10.2517511	1.3937679326	5.0872529541	44.885817	ns
YAP1	-0.05868733	-0.01888789	-10.2375549	1.6109558255	5.7686875263	44.74183566	ns
PALM	-0.06556491	-0.01213568	-10.2371618	1.6199738943	5.7686875263	44.73828095	ns
CPM	-0.08779222	-0.0439586	-10.218207	1.9662341766	6.9173539707	44.54604249	ns
HSPG2	-0.05799902	-0.01699601	-10.1920086	2.5790112269	8.9651342652	44.28309767	ns
TAFA5	-0.07931372	-0.0233528	-10.1656885	3.3677910870	1.1569352910	44.01818811	ns
MZB1	-0.1195919	-0.05932045	-10.1427922	4.2476274542	1.4422176937	43.78829474	ns
AGRN	-0.07652832	-0.02782697	-10.1313246	4.7714410564	1.6014491821	43.67328283	ns
OCLN	-0.09234605	-0.03554469	-10.0902552	7.2277913550	2.3983125859	43.26240021	ns
SMOC1	-0.07670289	-0.05614878	-10.0792934	8.0756483185	2.6495385494	43.15278302	ns
COL15A1	-0.05669043	-0.01423475	-10.0578818	1.0029292009	3.2539480740	42.94032811	ns
APOF	0.054688892	-0.01808688	9.992845441	1.9291837070	6.1903477194	42.29629215	ns
RETN	-0.09513671	-0.01707067	-9.98998726	1.9901801224	6.3166586495	42.26917125	ns
VSIG4	-0.09147405	-0.04366268	-9.98793188	2.0289399968	6.3704352587	42.24785769	ns
OSM	-0.1704173	-0.07276487	-9.97691777	2.2677008074	7.0443471890	42.13944234	ns
WFDC2	-0.07983945	-0.01115656	-9.95825126	2.7495344960	8.4512007667	41.95860797	ns
PLTP	0.084824089	0.006951874	9.939473719	3.2909143827	1.0009864580	41.77063887	ns
PRAP1	-0.09583367	-0.09612293	-9.92560624	3.7773453438	1.1370977736	41.63418636	ns
CD300A	-0.06327686	-0.01863664	-9.91216416	4.3333872115	1.2911725160	41.50478653	ns
LTBP3	-0.12656933	0.009939478	-9.90121426	4.8370444158	1.4156071245	41.39718175	ns
RNASET2	-0.05870519	-0.01705792	-9.90056907	4.8479696047	1.4156071245	41.38936457	ns
PTGDS	-0.06372015	-0.01244456	-9.89240409	5.2737766481	1.5246958230	41.3107138	ns
CD5	-0.07739943	-0.01939922	-9.86317585	7.0204592088	2.0097785186	41.02305931	ns
LGALS1	-0.07732508	-0.03511353	-9.85217678	7.8427873995	2.2233921559	40.91759873	ns
ADAM8	-0.06447348	-0.02241135	-9.79912832	1.3211266035	3.7093170023	40.40102973	ns
NOMO1	-0.055773	-0.02945913	-9.79491693	1.3785794898	3.8337639147	40.36100042	ns
MFAP5	-0.09433964	-0.03717505	-9.78365824	1.5377557226	4.2360818020	40.251394	ns
PI3	-0.1209402	-0.03348576	-9.77826297	1.6280437547	4.4428857607	40.20205786	ns
CTSZ	-0.07182271	-0.02756025	-9.7552628	2.0359522395	5.5046116107	39.97857112	ns
LRIG1	-0.07767983	-0.02725682	-9.70481815	3.3269818570	8.9126486446	39.49166586	ns

PLAUR	-0.05986705	-0.03703801	-9.68465882	4.0594989470	1.0776124477	39.30109618	ns
IFI30	-0.08198411	-0.04655162	-9.68011052	4.2468053239	1.1171776167	39.25714172	ns
C3	-0.10139721	-0.00887647	-9.66061857	5.1554883688	1.3441094675	39.07503985	ns
NBL1	-0.05459654	-0.01560759	-9.62461473	7.2502117066	1.8735060339	38.7262046	ns
LMNB2	-0.05672293	-0.02366368	-9.6149429	7.9559227865	2.0378328540	38.63366696	ns
DDC	0.106409511	0.041972203	9.603204797	8.9280226829	2.2669414116	38.52368895	ns
PRCP	-0.07103082	-0.04334977	-9.57421677	1.1787440424	2.9671832793	38.24797265	ns
NPPC	-0.0991335	-0.00057409	-9.55747406	1.3857143384	3.4583639899	38.0896809	ns
LCN2	-0.08145324	-0.01664785	-9.52041665	1.9874014715	4.9179765228	37.74504474	ns
CD74	-0.07528479	-0.03293008	-9.51678813	2.0435399798	5.0144006229	37.70480104	ns
COL18A1	-0.05027551	-0.03496446	-9.50659051	2.2557706187	5.4890418390	37.61085707	ns
EFNA1	-0.05699701	-0.01533742	-9.46893911	3.2306972397	7.7963933390	37.25716364	ns
CD59	-0.04688004	-0.02144653	-9.45537683	3.6956398762	8.8453019988	37.13519157	ns
F7	-0.06724937	-0.03198176	-9.45036672	3.8550699729	9.1518734317	37.08409849	ns
FGFR2	-0.04533057	-0.02253775	-9.44782857	3.9451472786	9.2901855271	37.05923739	ns
CD300C	-0.07337033	-0.02114561	-9.44235079	4.1532593462	9.7020138327	37.0069881	ns
ITGA5	-0.05523018	-0.02706417	-9.41942187	5.1676710550	1.1975872603	36.79447981	ns
CD72	-0.08557073	-0.01876961	-9.37637562	7.7756580299	1.7877890903	36.39733531	ns
IGFBP6	-0.05943002	-0.01031694	-9.36853582	8.3677979369	1.9089039043	36.32363812	ns
CLEC6A	-0.10094489	-0.02380811	-9.32077718	1.3123867207	2.9706738175	35.88328962	ns
PGF	-0.0519586	-0.02196675	-9.31668342	1.3582453194	3.0508279483	35.84138247	ns
BSG	-0.03995363	-0.0147872	-9.29516603	1.6615656216	3.7036424544	35.64348214	ns
SIT1	-0.12161993	-0.022559	-9.25639124	2.3933786435	5.2944436660	35.29175778	ns
RNF149	-0.05846551	-0.02630013	-9.23968697	2.7963128602	6.1392733473	35.1391808	ns
LILRB4	-0.0929308	-0.03482651	-9.23024937	3.0518085642	6.6502097072	35.05280151	ns
GDF15	-0.0813677	-0.05624946	-9.15479555	6.1127758699	1.3221707807	34.36254291	ns
CD27	-0.08080719	-0.03557877	-9.13382954	7.4175996904	1.5926022864	34.1737796	ns
IGFBP1	0.283405602	0.008823951	9.123497593	8.1532152828	1.7377655931	34.07990212	up
GPD1	-0.1099884	0.002666076	-9.11490444	8.8244092862	1.8671938489	34.00237655	ns
NPDC1	-0.05961192	-0.01434314	-9.06944178	1.3390804341	2.8130322788	33.59540619	ns
TNFRSF19	-0.05950836	-0.0182122	-9.04225886	1.7129817574	3.5727905226	33.3488389	ns
CHCHD10	-0.07602349	-0.04371587	-9.01222196	2.2509268658	4.6614939350	33.08138275	ns
CKAP4	-0.05223284	-0.00595406	-8.99497507	2.6328549113	5.4140396769	32.92840023	ns
CALB2	-0.06351779	-0.00223921	-8.95696001	3.7220694603	7.6003096672	32.5946737	ns
CRHBP	-0.05865314	-0.04154438	-8.90888261	5.7255479566	1.1610138912	32.16763158	ns
PAG1	-0.11513804	-0.01126442	-8.89378515	6.5670264627	1.3224632600	32.03694616	ns
PRSS8	-0.08498993	-0.05488094	-8.88723363	6.9542427930	1.3903174363	31.97722381	ns
C1RL	-0.03862548	-0.02239994	-8.88636635	6.9992007923	1.3903174363	31.96706123	ns
PZP	-0.10584383	0.208852998	-8.88336659	7.1880204322	1.4181770042	31.94039868	ns
ADIPOQ	0.132693378	0.057234325	8.871437344	7.9971521133	1.5672271255	31.8355985	ns
TREM2	-0.10505248	-0.06070506	-8.85584068	9.2023706082	1.7913948117	31.70145206	ns
IGSF9	-0.16987604	-0.0458287	-8.85348403	9.3876935022	1.8153685448	31.67812285	ns
EFEMP1	-0.06239785	-0.03396737	-8.82254941	1.2377818353	2.3778440520	31.4100059	ns
TNFRSF10B	-0.07867418	-0.03318523	-8.79055572	1.6446385735	3.1387873429	31.13168757	ns
SCARB2	-0.05723622	-0.02528381	-8.78828839	1.6808887661	3.1871397384	31.11483826	ns
OGN	-0.08082507	-0.04045438	-8.78144879	1.7815766869	3.3562605973	31.05099429	ns
CD79B	-0.08797343	-0.02614915	-8.76697425	2.0227173137	3.7861118950	30.92350611	ns
PGLYRP1	-0.08598551	-0.00844634	-8.75610987	2.2279293172	4.1436647173	30.83093237	ns
B2M	-0.05606992	-0.0022426	-8.74239759	2.5130720015	4.6444115472	30.71087606	ns
SERPINA7	-0.03535088	-0.01020756	-8.73414247	2.7025734702	4.9632166874	30.63947894	ns
DLL1	-0.05613193	-0.01921755	-8.72777465	2.8593541057	5.2183212430	30.58545876	ns
QPCT	-0.05408162	-0.03056179	-8.7067337	3.4418926707	6.2424388810	30.4052503	ns
LTBR	-0.05301651	-0.00391684	-8.6979799	3.7190785532	6.6823853810	30.33151924	ns
SIRPB1	-0.09053181	-0.03952379	-8.69759253	3.7302356750	6.6823853810	30.32724432	ns
FCER2	-0.10167653	-0.0254095	-8.68720862	4.0838009966	7.2711578721	30.23635768	ns
SNCG	-0.13872734	-0.09860755	-8.6726946	4.6395867520	8.2106626156	30.11325111	ns
CREG1	-0.07706189	-0.00221972	-8.66341721	5.0348086329	8.8564103664	30.03515229	ns
NOS3	-0.10500836	-0.03188146	-8.63474699	6.4682717299	1.1309792485	29.7905378	ns
DPT	-0.05577136	-0.06173397	-8.61993401	7.3380547474	1.2754238013	29.65846613	ns
PIK3IP1	-0.04918642	-0.01434001	-8.60771138	8.1667132669	1.4110534165	29.55593048	ns
FETUB	-0.06859216	-0.03306284	-8.59290407	9.2879319635	1.5953389019	29.43073645	ns
LDLR	-0.11185646	-0.04661866	-8.58841407	9.6513611983	1.6480686958	29.39128482	ns
CCL16	-0.10489407	-0.12633205	-8.57876236	1.0486479712	1.7802628349	29.30798319	ns
TREML2	-0.0665001	-0.00246043	-8.55241754	1.3177082209	2.2241086735	29.08631628	ns
THY1	-0.05417852	-0.01898924	-8.5483825	1.3632894330	2.2878190485	29.05005805	ns
CRIP2	-0.07424235	-0.04293681	-8.52673003	1.6449404639	2.7447006598	28.87084175	ns

CCL18	-0.14298978	-0.03386284	-8.46870277	2.7076824377	4.4922913172	28.38548089	ns
BTN2A1	-0.04754739	-0.01175012	-8.44918027	3.1928253345	5.2672598739	28.21727583	ns
CRELD1	-0.06956573	-0.04364029	-8.42834705	3.8182323216	6.2636170669	28.04787778	ns
ACVRL1	-0.04526047	-0.02774798	-8.42712447	3.8560675072	6.2903447604	28.03543669	ns
NT5C1A	-0.05852247	-0.01621346	-8.42314365	3.9831761132	6.4615968059	27.99845469	ns
ADH4	-0.17434832	-0.03484316	-8.39958926	4.8762707008	7.8666908544	27.80831496	ns
GSTA1	-0.17558046	-0.00775496	-8.37538306	5.9771439506	9.5897034812	27.60422034	ns
POLR2F	-0.06718233	-0.00156641	-8.3461413	7.6503846338	1.2207171109	27.36231165	ns
ITIH4	-0.04671522	0.006965556	-8.33192255	8.6142934010	1.3670509092	27.24168494	ns
RBFOX3	-0.0660611	-0.03056084	-8.29757635	1.1491285342	1.8137596324	26.95848404	ns
EFCAB14	-0.04673215	-0.00638845	-8.29645853	1.1602909616	1.8215320473	26.95028356	ns
PLAT	-0.10379862	-0.09331766	-8.27896075	1.3433559952	2.0976467947	26.80708477	ns
CANT1	-0.03837827	-0.01148163	-8.26736889	1.4809864769	2.3002555918	26.7142957	ns
HJV	-0.08101479	-0.01201012	-8.23360801	1.9599458799	3.0280645341	26.43487889	ns
TNFRSF11A	-0.07015117	-0.0354379	-8.19261383	2.7536808260	4.2319726379	26.10143694	ns
OXT	-0.28082565	0.000105097	-8.17271304	3.2464219276	4.9631162453	25.9400717	down
RBP5	-0.11066751	-0.0139115	-8.14709777	4.0136841128	6.1041445883	25.7370114	ns
LRP11	-0.06517773	-0.02729547	-8.1197228	5.0259358776	7.6040066127	25.51636679	ns
NUCB2	-0.07102105	-0.00627497	-8.09572825	6.1125508873	9.2003343252	25.31985684	ns
DSC2	-0.06057744	-0.01553323	-8.08220303	6.8369249494	1.0237856847	25.21727624	ns
PRTN3	-0.08794231	-0.00236403	-8.0648758	7.8872674787	1.1750418896	25.08469346	ns
CEACAM8	-0.09138098	-0.03628282	-8.06010377	8.1918050346	1.2142167868	25.04221566	ns
XG	-0.05736989	-0.006128	-8.04233122	9.4511855861	1.3938112076	24.89528009	ns
CA5A	-0.17331594	-0.01591403	-8.0357761	9.9842302561	1.4650227310	24.84833865	ns
ANG	-0.06429903	-0.02723474	-8.02298341	1.1068212897	1.6159590829	24.74441653	ns
IFNLR1	-0.0711839	-0.01527757	-8.01327355	1.1968175275	1.7386602888	24.66465677	ns
DPY30	-0.11266696	-0.00910265	-8.00576711	1.2711264297	1.8374698884	24.60308888	ns
C19orf12	-0.11683852	0.073978553	-7.9990118	1.3449375172	1.9345899262	24.55635491	ns
CCDC80	-0.0634406	-0.03987433	-7.99562584	1.3807557256	1.9763758425	24.52605758	ns
IL12B	-0.10063679	-0.02336091	-7.97385074	1.6445027497	2.3424136728	24.34994342	ns
IGSF8	-0.05454819	-0.02036144	-7.96570918	1.7577250279	2.4915325639	24.28958134	ns
RIDA	-0.08833619	0.006284974	-7.96159934	1.8181833111	2.5647803229	24.26072127	ns
VEGFB	-0.05044443	-0.0276868	-7.94591753	2.0635910110	2.8969643040	24.13837156	ns
MMP9	-0.1146491	-0.07186344	-7.93782095	2.1993620939	3.0727929734	24.06906397	ns
LECT2	-0.11293998	-0.06099332	-7.93631564	2.2245799908	3.0932255111	24.05461936	ns
ATRAID	-0.04476529	-0.00162156	-7.89653442	3.0592939877	4.2337149024	23.74328022	ns
ITGAV	0.029720789	-0.00060153	7.894642871	3.1051245738	4.2768696961	23.7268583	ns
FGA	-0.05374514	-0.01775554	-7.86446945	3.9562526577	5.4235951928	23.49891966	ns
IL12A_IL12B	-0.13993815	-0.00296878	-7.85367689	4.3071752842	5.8770802943	23.41200061	ns
SPON2	-0.06012667	-0.02850134	-7.82110845	5.5779855115	7.5756826483	23.16032838	ns
CAPG	-0.10863356	-0.06983188	-7.81627046	5.7946778053	7.8335459220	23.12170445	ns
CCN5	-0.0721884	-0.03328942	-7.78411377	7.4657883027	1.0046129882	22.87150896	ns
IL16	-0.07536397	-0.03643258	-7.73998315	1.0552853630	1.4135014955	22.52951929	ns
HNMT	-0.08627667	-0.05734174	-7.73899835	1.0649188533	1.4198918044	22.52821479	ns
EZR	-0.04484388	-0.02376248	-7.71581961	1.2749945794	1.6922655327	22.3444301	ns
EPHA2	-0.05051954	-0.02213227	-7.70974385	1.3400957124	1.7706241993	22.31019254	ns
BST2	-0.09040805	0.015131446	-7.69332775	1.5195109102	1.9986359720	22.17272296	ns
HLA-E	-0.04909353	-0.00444427	-7.69147839	1.5425065832	2.0197844049	22.16150295	ns
TNFRSF9	-0.06936137	-0.0101551	-7.68471784	1.6263150533	2.1200178373	22.1119801	ns
SIGLEC8	-0.07523064	-0.03199942	-7.67812148	1.7132030242	2.2233568136	22.06536217	ns
CD38	-0.0541055	-0.02282377	-7.6741505	1.7653985846	2.2809574633	22.0311767	ns
CCL7	-0.10756645	-0.02767532	-7.66592378	1.8815477485	2.4203169276	21.96708287	ns
MAD1L1	-0.08759068	9.3353285535	-7.65633723	2.0279138683	2.5971528489	21.89739706	ns
ICAM1	-0.05098583	-0.02943166	-7.64856539	2.1538222650	2.7463585213	21.83878833	ns
SHBG	0.089756579	0.033412638	7.633371644	2.4200048782	3.0723540193	21.71879246	ns
SUOX	-0.10379312	0.065689172	-7.61988077	2.6853780560	3.3945038630	21.61555387	ns
EGLN1	-0.09441325	0.007027714	-7.5813022	3.6164324533	4.5517167085	21.3278634	ns
UPB1	-0.15441317	-0.00720081	-7.55305796	4.4884763622	5.6250433380	21.11369282	ns
CFP	-0.03786676	-0.0121674	-7.54797573	4.6663350193	5.8229479728	21.07565874	ns
PSAP	-0.04504756	-0.01470993	-7.53391434	5.1987085809	6.4596719388	20.97351592	ns
CXCL17	-0.07752008	-0.04873897	-7.51889	5.8257119021	7.2080842179	20.85797908	ns
SELE	-0.09261259	-0.10645912	-7.51544671	5.9809785046	7.3689692968	20.8326906	ns
ARSA	-0.07858594	0.019352267	-7.5057892	6.4403402012	7.9015938604	20.76322601	ns
TNFRSF10A	-0.0571957	-0.04336158	-7.48884748	7.3276521198	8.9526126317	20.63957806	ns
CSF3R	-0.04580314	-0.0081714	-7.48586572	7.5015576123	9.1268950949	20.62219483	ns
GHRL	0.141618565	0.053246639	7.475207451	8.1251413898	9.8445696508	20.53783759	up

FN1	-0.04366008	-0.0144894	-7.47044961	8.4180020200	1.0157258635	20.49871354	ns
SERPINB8	-0.09811904	-0.01466746	-7.44632531	1.0101137963	1.2114062169	20.31984581	ns
CCN3	-0.05463521	-0.02570954	-7.44606585	1.0122709483	1.2114062169	20.31917754	ns
GM2A	-0.05978521	-0.00674073	-7.44560516	1.0171027321	1.2122203991	20.32282317	ns
PRG2	-0.08154508	0.009338928	-7.43878303	1.0694390738	1.2688522262	20.26506292	ns
SCRG1	-0.04140666	-0.02391007	-7.43830177	1.0733099311	1.2688522262	20.26121294	ns
ISM1	-0.06769637	-0.03915717	-7.41934899	1.2385912840	1.4538546932	20.12412985	ns
CCER2	-0.09647664	0.035620043	-7.41934304	1.2397596528	1.4538546932	20.12997685	ns
CCL20	-0.14817711	0.014891783	-7.41788315	1.2515526550	1.4618135011	20.11033476	ns
PCBD1	-0.07638021	0.020059573	-7.4004254	1.4289252241	1.6623353204	19.99147286	ns
SERPINA3	-0.02308996	-0.00450974	-7.39529574	1.4834584907	1.7189280924	19.94636291	ns
TIMD4	-0.07578178	-0.02280482	-7.39289616	1.5107450799	1.7436267325	19.9306485	ns
CALCA	-0.10459997	0.02962224	-7.39172871	1.5246165624	1.7527088041	19.92422279	ns
GOLM2	-0.03878645	-0.02292632	-7.35262218	2.0406194003	2.3367092740	19.63256953	ns
GPR37	-0.10963655	-0.09750955	-7.33336027	2.3587613494	2.6904621641	19.501263	ns
CCL21	-0.06824597	-0.03138217	-7.22828719	5.1207200865	5.8180944174	18.73657713	ns
FTCD	-0.13670104	0.059164144	-7.22476467	5.2539949019	5.9463818269	18.71032941	ns
CNTN4	0.040064772	0.008460208	7.206979012	5.9867313428	6.7495195062	18.58458902	ns
CDHR2	-0.13278855	-0.17423928	-7.2056393	6.0469652105	6.7912070825	18.57691816	ns
FABP5	-0.11747493	-0.02514401	-7.18415195	7.0713874610	7.9112840560	18.42004502	ns
IGFBP2	0.114713913	-0.02548447	7.181781155	7.1940732211	8.0178220632	18.40300673	ns
CSTB	-0.07275517	-0.05140729	-7.18126193	7.2286276478	8.0257006584	18.40670696	ns
IDUA	-0.0766398	-0.06730344	-7.18041453	7.2653461135	8.0359131255	18.39162952	ns
ST3GAL1	0.073311603	0.060155425	7.169002475	7.9002206565	8.7051487989	18.31590672	ns
SCLY	-0.0807635	-0.00612037	-7.15846564	8.5251110948	9.3583926304	18.2365124	ns
PILRA	-0.06855234	-0.07638171	-7.14298767	9.5421770003	1.0435639266	18.1294517	ns
MET	0.033221085	0.019595455	7.133236511	1.0238991262	1.1155915853	18.05933804	ns
CTSD	-0.06501754	0.007988467	-7.13259265	1.0326028228	1.1208922834	18.07957148	ns
CD22	-0.06876941	-0.00931444	-7.10314559	1.2724248005	1.3761038584	17.84507215	ns
SKAP1	-0.12405003	-0.01034146	-7.08577536	1.4432997220	1.5551421359	17.72911188	ns
PIGR	-0.05896549	-0.01968801	-7.08275014	1.4754748225	1.5839656183	17.7093565	ns
LGALS3BP	-0.04701701	0.011790123	-7.08147461	1.4875985954	1.5911310984	17.69327954	ns
APBB1IP	-0.07783902	0.008427928	-7.0781393	1.5245356622	1.6246876400	17.67427433	ns
FAM3C	-0.04618024	-0.02927723	-7.07585737	1.5498139392	1.6456206191	17.65838737	ns
MXRA8	0.04835557	0.034231663	7.073876136	1.5726531110	1.6592055466	17.64846342	ns
GRN	-0.03936114	-0.01926381	-7.07370917	1.5739723850	1.6592055466	17.6433882	ns
DDT	-0.06533317	0.017563997	-7.05977943	1.7385375178	1.8260897669	17.54152263	ns
CLEC7A	-0.09636178	-0.12431534	-7.05166934	1.8423770332	1.9282225580	17.48386367	ns
TNF	-0.06183566	-0.00662835	-7.0365411	2.0556827387	2.1405466337	17.38759981	ns
SMOC2	-0.06172543	-0.02954394	-7.03612043	2.0599096030	2.1405466337	17.37786474	ns
BAIAP2	-0.10406181	-0.01061989	-7.0222357	2.2769971484	2.3577417281	17.28842909	ns
ERBB4	0.0384905	0.011304172	7.021270338	2.2902889570	2.3631250015	17.27391943	ns
MCAM	0.054278134	0.016806363	7.01944982	2.3207485085	2.3861217059	17.26310341	ns
DEFA1_DEFA1I	-0.08302854	0.009762663	-7.01873375	2.3354257999	2.3875087572	17.26815292	ns
PTS	-0.09104045	0.018822638	-7.01852196	2.3384503581	2.3875087572	17.2632266	ns
TNFRSF8	-0.07177018	0.016251564	-6.98776725	2.9066887983	2.9573279759	17.03906144	ns
CA14	0.058881505	0.057241502	6.974038143	3.2040312898	3.2485317244	16.94351819	ns
GLA	-0.05853412	-0.02396195	-6.95891617	3.5699990529	3.6070578666	16.84960387	ns
IL18BP	-0.04724581	-0.01793789	-6.95402591	3.6933195726	3.7187907421	16.80991474	ns
SPINK2	-0.05451195	-0.02333042	-6.94712084	3.8800953508	3.8934290118	16.76758226	ns
SORCS2	-0.05354204	-0.0212106	-6.94571011	3.9200155035	3.9200155035	16.75863625	ns
RBP7	-0.09315889	0.042330818	-6.94478885	3.9412956099	3.9278440891	16.743604	ns
LSP1	-0.06693509	-0.01668445	-6.94044457	4.0652623545	4.0376075086	16.71565791	ns
ASGR2	-0.03813904	-0.00895729	-6.93878792	4.1116577082	4.0698442399	16.70238447	ns
SERPINA6	0.025958414	0.020901187	6.925378585	4.5190921110	4.4580232987	16.60935126	ns
IMMT	-0.06526827	-0.00701378	-6.92434305	4.5526654369	4.4760212376	16.60293303	ns
CA6	0.103422384	0.055544365	6.912490486	4.9503029856	4.8506324557	16.52460056	ns
CD46	-0.0450027	-0.00838459	-6.90532585	5.2077851111	5.0858637205	16.4792806	ns
LCAT	-0.03484195	-0.02928045	-6.90019812	5.3944612672	5.2506089668	16.4376132	ns
TIMP1	-0.04346802	-0.01831708	-6.88618646	5.9574272323	5.7792981788	16.35127836	ns
C1S	-0.03180008	-0.01995883	-6.88507543	5.9975797578	5.7989844016	16.33338644	ns
SFRP1	-0.06860621	-0.05033451	-6.86428954	6.9408559344	6.6888776661	16.19846531	ns
AHNAK	-0.04835808	-0.01915085	-6.85922151	7.1930612473	6.9091246191	16.16652191	ns
MMP8	-0.11777319	-0.03214886	-6.83415575	8.5567550833	8.1920409322	15.98696547	ns
IGSF3	-0.05481719	0.002100542	-6.83282948	8.6399065851	8.2446167413	15.98262683	ns
PLA2G15	-0.04077307	-0.01449308	-6.82790026	8.9380682861	8.5013548519	15.9462312	ns

GIGYF2	-0.11596109	0.043087227	-6.82397274	9.1833738890	8.7063155052	15.91746323	ns
CKB	0.094891927	0.014531382	6.815782556	9.7213806786	9.1865474374	15.86300777	ns
AHSG	-0.03989866	-0.01146139	-6.80539678	1.0447050911	9.8404479555	15.79339606	ns
IL19	-0.1076821	-0.00312237	-6.79596278	1.1165892079	1.0483731470	15.74009359	ns
NECTIN4	-0.04417926	-0.00957149	-6.78746121	1.1828142866	1.1069928580	15.67323365	ns
CXCL13	-0.09307569	0.011000087	-6.77412558	1.2966386358	1.2096437113	15.58130405	ns
APOM	0.042836389	0.01448561	6.766088216	1.3710481863	1.2749874853	15.53188112	ns
ULBP2	-0.05541818	-0.02186822	-6.7651364	1.3802512548	1.2770298781	15.52675351	ns
PDGFC	-0.04568	-0.00982866	-6.76503188	1.3819912380	1.2770298781	15.5306741	ns
FBLN2	-0.04343154	-0.00874705	-6.76229095	1.4068697240	1.2959178530	15.50286731	ns
CD4	-0.04638403	-0.02624323	-6.73534694	1.6936315493	1.5551585296	15.3259706	ns
FABP3	-0.07303929	-0.02465986	-6.7247802	1.8201069085	1.6660539727	15.2516712	ns
TNFRSF12A	-0.05242948	-0.03569182	-6.7063241	2.0651912268	1.8844869945	15.13035202	ns
FCAR	-0.07443322	-0.03028272	-6.69407334	2.2457051980	2.0428221739	15.05018476	ns
CHRDL1	-0.04666588	-0.02839594	-6.69240112	2.2712597012	2.0596516545	15.03912785	ns
GSTA3	-0.12383519	0.061480986	-6.68173533	2.4442502509	2.2096627655	14.9753901	ns
GUCA2A	0.049383314	0.007761604	6.679620196	2.4784127028	2.2336312013	14.95644281	ns
LBR	-0.10700798	0.035417087	-6.6356565	3.3376726365	2.9987704919	14.66050896	ns
AMOT	-0.05885932	0.015524282	-6.63464323	3.3632279917	3.0124618821	14.66363943	ns
PAMR1	-0.04485105	-0.03393919	-6.62192804	3.6630263533	3.2709593124	14.5735818	ns
GAPDH	-0.04969843	-0.08122497	-6.62075492	3.6968152065	3.2910671960	14.58046019	ns
BID	-0.10360297	0.014622055	-6.61492825	3.8402964498	3.4084090071	14.52786082	ns
C1R	-0.0277893	-0.00150595	-6.59647341	4.3469322630	3.8463764267	14.40404856	ns
PHLDB1	-0.07696543	-0.00647467	-6.57711407	4.9505674350	4.3672679487	14.27838878	ns
ADH1B	-0.11030612	0.054649693	-6.56746056	5.2847887090	4.6480671778	14.22386195	ns
ERN1	-0.05360109	-0.00836406	-6.56323637	5.4328933996	4.7639785966	14.18786935	ns
CXCL16	-0.03571697	-0.020789	-6.55017956	5.9279844294	5.1825492616	14.10266761	ns
CSF3	-0.07606254	-0.0016873	-6.54229713	6.2529783953	5.4503572878	14.06030078	ns
TGOLN2	-0.03541465	-0.01089464	-6.53614074	6.5100205338	5.6575178449	14.01200193	ns
CTSS	-0.03774519	-0.01793221	-6.51408916	7.6066216697	6.5909006752	13.95527088	ns
TNFRSF10C	-0.07537509	-0.09670437	-6.5122407	7.6339277746	6.5949908585	13.85986989	ns
SORD	-0.10458021	-0.03154459	-6.51090711	7.7002467930	6.6326609544	13.84826873	ns
REN	-0.10393955	-0.01075304	-6.5036406	8.0829475928	6.9418255797	13.80552448	ns
ADAM12	-0.04921447	0.001891794	-6.49765116	8.4149977748	7.2058045462	13.77333591	ns
PLIN1	-0.05513635	0.007576662	-6.46210059	1.0643053061	9.0870511518	13.5433413	ns
ANGPTL2	-0.06288099	-0.03470297	-6.45755135	1.0960456570	9.3121784949	13.50796537	ns
COL4A1	0.060124755	0.018352175	6.4574303	1.0970511651	9.3121784949	13.50849368	ns
PXN	-0.08092343	-0.0001078	-6.4559883	1.1080435129	9.3782233556	13.50418692	ns
SPINK6	-0.08308222	-0.11993858	-6.43659517	1.2576817832	1.0613961869	13.36936972	ns
SSC5D	-0.06669562	-0.05329165	-6.42874719	1.3245829958	1.1146346823	13.32424127	ns
ITGA11	0.0510061	0.015084387	6.422200675	1.3826974467	1.1601944093	13.28177811	ns
LY6D	-0.05280318	-0.01867656	-6.42099147	1.3939589930	1.1662923380	13.27715928	ns
CNTN3	-0.05003283	-0.041332	-6.41668834	1.4334723201	1.1959254785	13.24644023	ns
FNDC1	-0.06496518	-0.03292912	-6.40248262	1.5726815496	1.3083276709	13.15343696	ns
TMSB10	-0.11568618	-0.04443116	-6.40187517	1.5793172229	1.3101154235	13.15226159	ns
IL2RA	-0.0589957	-0.01663132	-6.3989622	1.6101672269	1.3319230319	13.13826921	ns
BPIFA2	0.156191578	0.132636422	6.393455884	1.6712236610	1.3785234718	13.11905342	up
CD274	-0.04936079	-0.00287671	-6.39155407	1.6897167887	1.3898515558	13.08945074	ns
PENK	-0.03988883	-0.00879142	-6.35902437	2.0885953091	1.7131175007	12.88776595	ns
OLR1	-0.08753708	0.012470295	-6.35439844	2.1522397242	1.7603753487	12.85794895	ns
LBP	-0.08676071	-0.06112739	-6.35141711	2.1934400581	1.7890628407	12.83406831	ns
CPXM2	-0.03353633	-0.0228814	-6.34297643	2.3162068120	1.8839342314	12.77717661	ns
ADGRG2	0.040400673	0.033110771	6.335526539	2.4314189132	1.9721508963	12.73502881	ns
PCDH9	-0.04546644	-0.00992646	-6.33014075	2.5169016776	2.0358318279	12.69731551	ns
CLEC4G	-0.04977902	-0.01516147	-6.3230179	2.6359874438	2.1262661149	12.6547916	ns
ST6GAL1	-0.0427131	-0.02674019	-6.31544637	2.7671818983	2.2203995518	12.60406699	ns
TNFRSF13B	-0.04904917	-0.01794622	-6.31544838	2.7678953317	2.2203995518	12.60810501	ns
TOMM20	-0.10542888	0.041646656	-6.31265822	2.8174979500	2.2539465993	12.58684617	ns
CDCP1	-0.07175359	-0.07187559	-6.31229678	2.8251522443	2.2539465993	12.59006569	ns
ADAMTSL2	-0.04303936	-0.00973417	-6.30658633	2.9299567962	2.3311917834	12.54918477	ns
TFPI2	-0.05294875	-0.00301056	-6.30361272	2.9869994752	2.3701191488	12.53262307	ns
HHEX	-0.10701766	-0.03271949	-6.30050823	3.0468017562	2.4110192759	12.51088234	ns
EPHB6	-0.04328564	0.000193873	-6.28913644	3.2794470232	2.5881041372	12.44617371	ns
DEFB4A_DEFB	-0.21498025	-0.14645893	-6.28546425	3.3581321774	2.6379557615	12.42447699	down
IL1RAP	0.066934968	0.143366245	6.28528976	3.3606833675	2.6379557615	12.41859723	ns
AIFM1	-0.1548151	-0.00148481	-6.28351001	3.3998806433	2.6615687610	12.4104037	ns

IGLC2	-0.03196574	0.004191152	-6.27788429	3.5236653054	2.7510969764	12.37035969	ns
ENPP6	0.052264853	0.036315162	6.274322662	3.6074871688	2.8090300088	12.3576517	ns
FGF23	-0.07966798	0.061514744	-6.26830754	3.7465162247	2.9095285575	12.30920115	ns
HYOU1	-0.03117953	-0.01521823	-6.26371802	3.8581348257	2.9882635785	12.28127849	ns
CES1	-0.11895042	0.040288191	-6.25653895	4.0404390289	3.1211857049	12.24052697	ns
SETMAR	-0.04587887	-0.01864916	-6.24297444	4.4071766085	3.3955028224	12.15961578	ns
FCRL1	-0.065812	-0.0024533	-6.23462739	4.6473112173	3.5710917775	12.10592709	ns
FCN1	-0.07622305	-0.06135624	-6.23099395	4.7550900783	3.6443210049	12.08046769	ns
STAB2	-0.03976547	-0.03118137	-6.21739407	5.1844171542	3.9629576153	11.99505549	ns
BCHE	-0.03884724	-0.02785392	-6.21579986	5.2401897915	3.9951316426	11.99388562	ns
CST7	-0.13830749	0.069101993	-6.18945083	6.1908345091	4.7076137413	11.82663301	ns
BMP10	0.038813165	0.01137317	6.188873051	6.2156255120	4.7141887000	11.82885408	ns
PPL	-0.04319302	-0.00607081	-6.18732228	6.2780682071	4.7492122188	11.82115802	ns
THBD	-0.0390757	-0.01595167	-6.18400424	6.4082745134	4.8351838706	11.79407927	ns
C4BPB	-0.05576153	0.004697046	-6.1667575	7.1440569047	5.3764551963	11.68532379	ns
B4GALT1	-0.0377772	-0.01137044	-6.15654099	7.6193158077	5.7193835883	11.62339121	ns
GAST	-0.15663533	0.237719385	-6.150499	7.9135370427	5.9250072217	11.58430899	ns
SGSH	-0.07514351	-0.01126797	-6.14628231	8.1293986327	6.0710598484	11.56448305	ns
S100A11	-0.06584725	0.026371865	-6.13981031	8.4675779994	6.3074815709	11.52664268	ns
ARSB	-0.07998281	-0.00677018	-6.13670926	8.6325341164	6.4139948142	11.50525258	ns
F11R	-0.08182825	0.017438556	-6.1347752	8.7369681157	6.4751134259	11.49125838	ns
OSCAR	-0.04765294	-0.0420941	-6.11274962	1.0029715658	7.4143720816	11.35846017	ns
LRCH4	-0.0934631	0.028761743	-6.11137042	1.0121781072	7.4635355380	11.35729437	ns
LRRC25	-0.05540648	-0.0111443	-6.10769962	1.0352306817	7.6142911607	11.32904349	ns
BTN3A2	-0.05253895	-0.03912376	-6.10129318	1.0774132542	7.9046399555	11.28826491	ns
DSG2	0.038072386	0.024864908	6.099219944	1.0914030015	7.9872099362	11.27546365	ns
AREG	-0.06245404	0.02018985	-6.09138292	1.1461955369	8.3672274196	11.23029841	ns
ADAMTS8	0.063536211	0.03360228	6.058002854	1.4102190347	1.0268926636	11.02573975	ns
SFRP4	-0.05498358	0.021823741	-6.05589801	1.4293693480	1.0382483821	11.02119433	ns
DUT	-0.13455312	-0.03891855	-6.05519806	1.4348954341	1.0396760962	11.00799921	ns
PDCD1	-0.05789282	0.010834746	-6.05294627	1.4549867233	1.0516240673	10.99299537	ns
TMPRSS11D	-0.04781024	0.003513503	-6.05157244	1.4674504774	1.0580136775	10.98582692	ns
DDAH1	-0.06258886	0.0037605	-6.05011304	1.4811815929	1.0652833131	10.98171536	ns
SULT2A1	-0.07624497	0.05863813	-6.02506431	1.7290708048	1.2405127150	10.82966525	ns
UMOD	0.075922239	0.009973374	6.019632976	1.7875934354	1.2793560861	10.79445511	ns
PON2	-0.04921285	-0.03382266	-6.01326292	1.8602760469	1.3281188403	10.7663143	ns
MMP12	-0.07158564	-0.05599083	-5.989622	2.1501545594	1.5313295886	10.61733077	ns
PHOSPHO1	-0.04062981	-0.03324967	-5.98853454	2.1648960744	1.5380770164	10.61449421	ns
CPE	0.054270265	-0.03581652	5.980008933	2.2805269164	1.6162957757	10.55966484	ns
NCF2	-0.12024807	0.06640549	-5.97723445	2.3199452635	1.6402518570	10.54561642	ns
CCL23	-0.05718112	-0.02345672	-5.96626553	2.4805982554	1.7496007018	10.47898415	ns
FAS	-0.04535006	-0.00716048	-5.94302857	2.8582374708	2.0110972083	10.34083453	ns
TALDO1	-0.05900991	-0.00492585	-5.942603	2.8657796963	2.0115569022	10.33875233	ns
CRELD2	-0.07200373	-0.02925098	-5.92845074	3.1235021628	2.1869884420	10.2566855	ns
PILRB	-0.07906814	-0.12970868	-5.92808923	3.1306889341	2.1869884420	10.25632199	ns
KHK	-0.08395591	-0.05240327	-5.92348873	3.2204170658	2.2443001986	10.2349463	ns
EDA2R	-0.04685967	-0.05887849	-5.91298615	3.4301251856	2.3827774684	10.16131262	ns
WFIKKN2	0.049930853	0.029312183	5.91276707	3.4354428569	2.3827774684	10.16531573	ns
DNMBP	-0.08712935	0.06235885	-5.8941254	3.8460797645	2.6612684627	10.05790777	ns
EPHB4	-0.03115409	-0.01053221	-5.88091186	4.1640066305	2.8744442934	9.974662722	ns
RET	-0.05416414	-0.01237474	-5.87674566	4.2710891867	2.9414104776	9.955836514	ns
CXCL10	-0.09264809	0.031406084	-5.87522375	4.3097542325	2.9610546727	9.944063318	ns
ENTR1	-0.07514524	0.065945696	-5.87203026	4.3954302997	3.0128301585	9.933818573	ns
CNTN1	0.03705981	0.024234468	5.864123944	4.6068897401	3.1503789323	9.876819382	ns
ACE2	-0.06521056	0.018380216	-5.85809665	4.7796444954	3.2608789548	9.852491373	ns
CD300LF	-0.08167153	-0.16256069	-5.84312931	5.2255500023	3.5567846169	9.753784277	ns
ZBTB17	-0.05227511	0.004713698	-5.84073299	5.3042719619	3.6019707276	9.751710644	ns
MEGF10	0.054386036	0.010438591	5.839276129	5.3489206907	3.6238627417	9.737851023	ns
DTNB	-0.05513558	-0.00528024	-5.83825131	5.3812167865	3.6373039390	9.728665561	ns
PDZK1	-0.05571121	0.020532367	-5.83423082	5.5150398377	3.7191492670	9.715567384	ns
MAMDC2	-0.04185878	-0.00412573	-5.83333802	5.5412839408	3.7282371214	9.698382396	ns
TPP1	-0.06074714	-0.01642557	-5.82332632	5.8853337327	3.9506148275	9.649040375	ns
GCHFR	-0.06586444	0.002623943	-5.82202427	5.9345456636	3.9745122334	9.652398705	ns
SCARA5	-0.03841458	-0.04072241	-5.80644598	6.5061046398	4.3473285007	9.542300403	ns
ELOA	-0.0760375	0.022069614	-5.80183543	6.6889566978	4.4583635754	9.521750608	ns
ADGRG1	-0.08375467	0.085784056	-5.80148773	6.7028137315	4.4583635754	9.5197487	ns

BAG3	-0.05523487	-0.04105563	-5.7998617	6.7684821389	4.4918108740	9.51169251	ns
MYH9	-0.14387473	-0.01766112	-5.7949778	6.9658519945	4.6123101642	9.47738985	ns
HDGFL2	-0.08152296	0.024071242	-5.79295766	7.0498610525	4.6573742700	9.465460488	ns
SPON1	-0.04215515	-0.03630137	-5.77562369	7.8151774753	5.1513133697	9.369919373	ns
FSTL1	-0.02751406	-0.01952285	-5.76680623	8.2370302807	5.4171460405	9.326204583	ns
AMN	-0.06407529	-0.00356769	-5.76356459	8.3962316218	5.5094373788	9.307061324	ns
SMNDC1	-0.07857907	0.040771788	-5.7562595	8.7628637666	5.7371215692	9.255959137	ns
PARP1	-0.07408787	0.039102978	-5.74649221	9.2843248374	6.0649280817	9.20256206	ns
FGR	-0.07975597	0.049065703	-5.74008628	9.6449639765	6.2864497346	9.17328025	ns
SMAD5	-0.02746818	-0.03768428	-5.72109045	1.0782290193	6.9971676941	9.060062638	ns
HMOX2	-0.06230803	0.033659794	-5.72112251	1.0783306377	6.9971676941	9.065437811	ns
ITGBL1	-0.04211304	0.002287456	-5.71340569	1.1277350505	7.3015218349	9.011514955	ns
MRC1	-0.03535424	-0.02580756	-5.70051983	1.2162017517	7.8568785732	8.939113809	ns
ACP5	-0.04355879	-0.05512518	-5.69939256	1.2243524441	7.8920731501	8.934149062	ns
CFHR5	-0.05077647	-0.05389789	-5.68565883	1.3269746678	8.5347269386	8.860615546	ns
KCTD5	-0.05535308	-0.00222491	-5.68133552	1.3609811544	8.7336639327	8.837750107	ns
GIPC2	-0.03583748	-0.00497731	-5.68090719	1.3638872442	8.7336639327	8.827424624	ns
SIGLEC10	-0.04387283	-0.01629258	-5.67981834	1.3728276940	8.7716780449	8.8245004	ns
KYNU	-0.06033082	-0.01720816	-5.6729778	1.4283814660	9.1067115305	8.78192901	ns
TGFB1	-0.05044974	-0.01279642	-5.66789831	1.4713170617	9.3600126806	8.753846001	ns
CELSR2	-0.03440502	-0.00448069	-5.66414164	1.5044780415	9.5501649591	8.743254393	ns
COCH	-0.05614894	-0.01111773	-5.66175018	1.5249487960	9.6591116799	8.719531629	ns
CCL3	-0.07251743	-0.02506131	-5.65730461	1.5651382301	9.8922156536	8.697832764	ns
VNN2	-0.06210622	-0.07590843	-5.65674558	1.5703151743	9.9034995874	8.696760927	ns
SDC1	-0.04932452	-0.00568996	-5.64954134	1.6373985530	1.0304318480	8.656092149	ns
HSPB6	-0.04963133	-0.02650018	-5.64842109	1.6476836826	1.0346744845	8.64335513	ns
HIP1R	-0.05233616	0.001337291	-5.64380052	1.6925427419	1.0605632632	8.619681145	ns
VSNL1	-0.04535903	0.002937973	-5.63757617	1.7546650511	1.0971353210	8.584017231	ns
PROK1	-0.08240985	-0.09842003	-5.62514434	1.8858883632	1.1756187925	8.517760309	ns
FOLR2	-0.03808163	-0.01235875	-5.62489763	1.8882370332	1.1756187925	8.512333998	ns
THOP1	-0.04111312	-0.00935662	-5.60702381	2.0941050951	1.3010184846	8.420267119	ns
NELL1	0.052482643	0.038081458	5.603091214	2.1416055020	1.3277044725	8.393400123	ns
ANGPTL4	-0.05961922	-0.05190204	-5.59711947	2.2166445563	1.3713140052	8.361676149	ns
PTPRS	-0.03124378	0.002261013	-5.58970786	2.3128382897	1.4277986904	8.316953041	ns
LGALS7_LGALS	-0.05320798	-0.00016966	-5.57942408	2.4540265475	1.5117631896	8.264873614	ns
MSR1	-0.06023563	-0.07517273	-5.57028645	2.5854855163	1.5893932016	8.208886236	ns
SIGLEC1	-0.05583839	-0.0364217	-5.56781721	2.6225654034	1.6088006256	8.198394207	ns
CFHR2	-0.06889877	-0.12068006	-5.54446563	2.9971222405	1.8347163401	8.070343472	ns
SIGLEC6	-0.04174543	-0.02438559	-5.53632113	3.1390026466	1.9175497339	8.021042842	ns
HEG1	-0.03240565	-0.03468438	-5.53557853	3.1535596983	1.9224205259	8.028745896	ns
AZU1	-0.10053639	0.066564546	-5.53063859	3.2448974803	1.9739793005	8.010837631	ns
MIA	-0.04640788	0.014763211	-5.52889649	3.2747849171	1.9880191181	7.983471867	ns
SELENOP	0.026401424	0.003889723	5.520818804	3.4284508201	2.0769868038	7.937308009	ns
XCL1	-0.07187198	0.011526021	-5.50960948	3.6535359222	2.2087629178	7.874588661	ns
FGF21	-0.15584803	-0.12870227	-5.50397417	3.7721741698	2.2757744991	7.844464621	ns
CD40	-0.063645	0.023642807	-5.4862342	4.1710403998	2.5098608951	7.753533021	ns
VEGFA	-0.07492897	0.029447754	-5.48593863	4.1773712158	2.5098608951	7.747662435	ns
ADAM22	-0.04336767	-0.00237658	-5.48276783	4.2536747388	2.5504579542	7.73554073	ns
IL18	-0.05899834	-0.0342467	-5.47643833	4.4082485798	2.6377225109	7.701302367	ns
TXNDC15	-0.03393632	0.000448018	-5.47599677	4.4185231177	2.6384637022	7.69452287	ns
ESYT2	-0.09920509	0.031658512	-5.47494796	4.4444376914	2.6485220528	7.687027317	ns
FUOM	-0.07674071	-0.00858004	-5.46934288	4.5886636764	2.7288997831	7.66700817	ns
CEACAM16	-0.09514772	-0.02144254	-5.46563445	4.6839432178	2.7799012593	7.637307039	ns
AGXT	-0.09849651	0.063705728	-5.46031484	4.8269531262	2.8589661518	7.612744521	ns
DMD	-0.06187959	0.068997858	-5.44558954	5.2442251010	3.0998253633	7.539019495	ns
TFF3	-0.05646139	0.034613405	-5.44425778	5.2825114225	3.1161481523	7.527248806	ns
NCAM2	0.035544969	0.025691835	5.442580221	5.3318845949	3.1389320599	7.514639606	ns
RGMB	-0.03157825	0.002459327	-5.43122133	5.6812570499	3.3378814055	7.449484263	ns
RNASE3	-0.14004359	0.064748747	-5.43023076	5.7142931644	3.3505494056	7.451966661	ns
PKD2	-0.07085661	0.05123548	-5.42708884	5.8168062476	3.4038224936	7.439948179	ns
CLEC14A	-0.03779954	-0.01898047	-5.42584942	5.8552698088	3.4194775683	7.424383669	ns
CCL19	-0.15165468	0.236038158	-5.42164573	5.9936060970	3.4932794018	7.397897942	ns
APOA1	0.032110517	0.031703827	5.419657996	6.0605745850	3.5252744598	7.387951402	ns
BMPER	-0.02600511	-0.01125567	-5.41439046	6.2434856372	3.6244489186	7.370184256	ns
AFM	-0.02382518	-0.01221922	-5.41068969	6.3714904189	3.6914190522	7.339737146	ns
PRND	-0.05661879	0.015342246	-5.39929882	6.7886943594	3.9253440652	7.278690024	ns

ZHX2	-0.03995854	0.027861721	-5.39163885	7.0840327442	4.0880188958	7.237658729	ns
NRCAM	-0.03424562	0.002249471	-5.37488554	7.7753915314	4.4781347676	7.153307954	ns
CDH3	-0.04224893	0.008297827	-5.37203281	7.8977364642	4.5396437943	7.132071903	ns
LEPR	0.040138736	0.027827652	5.359616005	8.4607258940	4.8536973694	7.071949095	ns
CD80	-0.03771382	-0.01691122	-5.35545238	8.6560861870	4.9560336600	7.044557507	ns
VIT	-0.0423812	-0.04245974	-5.3550525	8.6752160954	4.9572663402	7.042431424	ns
NFKBIE	-0.06247829	0.023023827	-5.35140778	8.8551786187	5.0502190560	7.034742463	ns
APEX1	-0.07562925	-0.01695463	-5.33646531	9.6112203356	5.4707141092	6.943988754	ns
VASN	-0.0339403	-0.01119955	-5.3174445	1.0674639830	6.0641922772	6.857803067	ns
PAXX	-0.05271388	0.01019013	-5.31697667	1.0700328408	6.0669823206	6.851344385	ns
KIT	0.036350134	0.015016934	5.307826175	1.1248588954	6.3654805711	6.797069619	ns
FRZB	-0.03210092	-0.03604365	-5.30369165	1.1507251808	6.4992602088	6.778599224	ns
LPL	0.0593166	0.009287675	5.302326503	1.1592461115	6.5347464202	6.768755753	ns
CWC15	-0.07278121	0.103608748	-5.29632608	1.1978199111	6.7391794614	6.735293387	ns
TPK1	-0.03094341	-0.00937659	-5.29269967	1.2216699284	6.8601465214	6.712849074	ns
SNRPB2	-0.05098242	0.03765377	-5.29022246	1.2382898855	6.9401275734	6.69988435	ns
PTP4A3	-0.06064591	0.02628235	-5.28820078	1.2525479494	7.0065900622	6.701923409	ns
JAM2	-0.02952658	-0.01175586	-5.2877295	1.2552419020	7.0082339463	6.685958213	ns
SCAMP3	-0.10666854	0.02090481	-5.28654208	1.2636308210	7.0416068651	6.686046621	ns
LAMA4	-0.04006105	-0.00547047	-5.280379	1.3066701352	7.2675748474	6.650635761	ns
TNFRSF17	-0.04556291	-0.03280297	-5.27847857	1.3205840381	7.3309988426	6.648885119	ns
DPP6	0.041500115	0.021483137	5.27059186	1.3783138103	7.6369569757	6.60246036	ns
CFC1	-0.05662881	0.017080982	-5.25191208	1.5257912211	8.4380878139	6.511128086	ns
FMNL1	-0.0784494	0.016024313	-5.2483819	1.5552236218	8.5845991981	6.491947284	ns
CNTN5	0.045112437	0.045766772	5.234558462	1.6754596674	9.2308343944	6.409813037	ns
MSLN	-0.06803888	0.033705889	-5.2232989	1.7807329716	9.7775350693	6.357010927	ns
NT5E	-0.06825779	0.037834143	-5.22320392	1.7813865263	9.7775350693	6.351929525	ns
TNFRSF6B	-0.06709249	-0.0338869	-5.21300224	1.8823101180	1.0312092954	6.303371839	ns
CD8A	-0.0601006	-0.01229686	-5.20998405	1.9127568932	1.0459269903	6.280778993	ns
TAX1BP1	-0.07345563	0.078405696	-5.20509167	1.9637635950	1.0718111584	6.25632174	ns
SFTPA2	-0.0604332	0.029464584	-5.19986562	2.0204648349	1.1007009921	6.241124285	ns
RSPO3	-0.03178453	-0.01457522	-5.18033327	2.2426202147	1.2194508430	6.127206137	ns
TRIAP1	-0.07716199	-0.0090717	-5.17613666	2.2935389161	1.2448203782	6.105628072	ns
HAVCR1	-0.07134035	-0.07149016	-5.16830615	2.3915896905	1.2956292943	6.065857283	ns
CILP	-0.06020866	-0.08372801	-5.16118925	2.4842064151	1.3433116170	6.030711188	ns
EGFR	0.01913092	0.012870119	5.158065828	2.5258680435	1.3633150993	6.01403449	ns
MTDH	-0.10319293	-0.00682864	-5.15553401	2.5611683845	1.3798176536	6.014219053	ns
TPR	-0.05563436	0.035559665	-5.15142194	2.6178818618	1.4077744082	5.992592839	ns
RPL14	-0.04523265	0.018893004	-5.1473135	2.6756470324	1.4361928924	5.970809945	ns
ENAH	-0.05658312	-0.00498068	-5.14487166	2.7105599607	1.4522633183	5.957695955	ns
FABP1	-0.11466155	-0.09211696	-5.14420043	2.7197704938	1.4545292750	5.948823178	ns
GH1	0.222065912	0.313304763	5.143056028	2.7364557885	1.4602073221	5.943343852	up
RRM2	-0.06097793	0.037135017	-5.14282294	2.7403890840	1.4602073221	5.948278938	ns
PLIN3	-0.05090915	0.011751979	-5.13497867	2.8563666343	1.5192332554	5.901693427	ns
ACAA1	-0.09791753	0.00510804	-5.12349983	3.0362709351	1.6119838419	5.84980792	ns
GFER	-0.07819907	0.021887839	-5.11761194	3.1324024387	1.6600027442	5.819876535	ns
RASSF2	-0.06975146	0.032255231	-5.10627386	3.3258636128	1.7593336502	5.762335442	ns
APOD	0.048348709	0.047112269	5.101117234	3.4182983703	1.8049604414	5.744277943	ns
ACY1	-0.0746287	-0.00095489	-5.09825807	3.4688181260	1.8237549437	5.715143586	ns
SIAE	-0.05598153	-0.00954337	-5.09795416	3.4741191272	1.8237549437	5.710848408	ns
LGMN	-0.04278607	-0.01921954	-5.09785176	3.4759714551	1.8237549437	5.710213173	ns
PRTG	0.029016961	0.009911725	5.097678299	3.4788750124	1.8237549437	5.705420606	ns
MVK	-0.09252211	-0.01441882	-5.0830579	3.7584402548	1.9667823555	5.643858853	ns
NADK	-0.06498577	0.003413089	-5.06608217	4.1081257291	2.1446134336	5.552778792	ns
ACHE	0.035651482	0.032052004	5.065881654	4.1129572700	2.1446134336	5.557251652	ns
PLA2G2A	-0.06618717	0.038841251	-5.06075623	4.2244232520	2.1988085375	5.525978821	ns
HAO1	-0.15276008	0.020620964	-5.05286852	4.4016864586	2.2869972347	5.479584097	ns
HS6ST2	-0.03508783	0.026341478	-5.03297397	4.8850772460	2.5336457475	5.39437642	ns
CYTL1	-0.0251524	-0.0130558	-5.03176714	4.9142533678	2.5442588358	5.375739254	ns
C2	-0.02824926	-0.03676342	-5.02937995	4.9762847750	2.5718144324	5.368157507	ns
KLRD1	-0.05427464	-0.02532786	-5.01888721	5.2546473301	2.7108781279	5.310259254	ns
TOP2B	-0.08698998	0.014177289	-5.01720561	5.3026802938	2.7308335904	5.315707585	ns
QDPR	-0.05087148	-0.01044036	-5.01611384	5.3320731648	2.7411362044	5.305023628	ns
TNFSF10	-0.03042748	-0.01090176	-5.01135594	5.4642152925	2.8041315736	5.272751099	ns
CRLF1	-0.03044425	-0.00557887	-4.9993126	5.8166630340	2.9797642209	5.216219778	ns
LY96	-0.04184726	-0.01404267	-4.99806962	5.8548179323	2.9888464442	5.213835252	ns

AOC3	0.035123119	-0.00902145	4.998056762	5.8548635825	2.9888464442	5.211775357	ns
HSD11B1	0.047368518	0.013495465	4.996189027	5.9117650740	3.0126272279	5.201695364	ns
FCRL5	-0.06682964	-0.05477698	-4.99462453	5.9597040958	3.0317658466	5.193638708	ns
ERP44	-0.03046498	-0.00243641	-4.99276626	6.0176745442	3.0559321163	5.187423898	ns
SUMF2	-0.06170474	-0.07254732	-4.98623262	6.2233039889	3.1548693832	5.148050334	ns
SEL1L	-0.03408459	0.00306191	-4.98426745	6.2886969327	3.1824948082	5.150783609	ns
DCXR	-0.06321844	0.019268869	-4.97578892	6.5681877811	3.3181848305	5.099118713	ns
LETM1	-0.05710906	0.027133092	-4.9715267	6.7136340632	3.3858050888	5.075373225	ns
GPKOW	-0.03331163	-0.00350804	-4.96335061	7.0044049538	3.5263555974	5.047100695	ns
MMP7	-0.05133168	-0.07096548	-4.95855559	7.1792873283	3.6081788293	5.024331194	ns
CSDE1	-0.13043434	0.236191659	-4.94330327	7.7637667002	3.8952231554	4.949688906	ns
SPOCK1	0.02934704	0.00351267	4.940559433	7.8723373714	3.9429202615	4.93005716	ns
SUSD4	-0.04382561	0.018281077	-4.92193853	8.6574636353	4.3287318176	4.833415233	ns
IGFBPL1	-0.03642793	-0.00875039	-4.9190114	8.7885493472	4.3867630929	4.82368038	ns
DLK1	-0.06286828	-0.01779627	-4.91345298	9.0399075370	4.5045273051	4.790798169	ns
SPINK1	-0.04355555	-0.00950071	-4.91160554	9.1262358176	4.5397970336	4.785300663	ns
WASHC3	-0.08279272	0.072432157	-4.9022141	9.5721326929	4.7535080720	4.736366005	ns
TIGIT	-0.03832511	0.007857633	-4.89977244	9.6931549689	4.8054350610	4.731410601	ns
ECHS1	-0.10239163	-0.02536621	-4.89889721	9.7376009512	4.8192872504	4.731913981	ns
NUDT5	-0.05933238	0.009041588	-4.88434058	1.0481975289	5.1789116488	4.652683201	ns
TNFSF13B	-0.03007997	-0.01005825	-4.87838352	1.0802216742	5.3281204200	4.621087108	ns
GASK1A	-0.04655326	-0.01555889	-4.86684767	1.1454292309	5.6402248810	4.577327147	ns
ANXA2	-0.07605987	0.022895266	-4.86362655	1.1643445835	5.7237141145	4.567380874	ns
SCN4B	-0.04330452	-0.01807436	-4.85928151	1.1899712028	5.8398586761	4.539989561	ns
PBXIP1	-0.03259438	0.009272955	-4.85191041	1.2346962504	6.0491829718	4.493622195	ns
NCR1	-0.04000131	0.008840206	-4.84804529	1.2590484895	6.1581601161	4.477584533	ns
ALDH1A1	-0.06133459	0.032720692	-4.84599673	1.2720714097	6.2114523688	4.467423002	ns
PEBP1	-0.06773449	0.00087832	-4.84295497	1.2916779204	6.2966603135	4.452788908	ns
GPC1	-0.03216532	-0.00822718	-4.81601826	1.4783301788	7.1945402036	4.318902306	ns
SCPEP1	-0.0526256	-0.00077027	-4.81415166	1.4926497533	7.2521418962	4.324840951	ns
CHGA	-0.08089554	0.07489896	-4.80985429	1.5246192904	7.3951633356	4.291281889	ns
PLSCR3	-0.06305173	0.021866194	-4.80513052	1.5610127968	7.5591332782	4.268986507	ns
BOC	0.026502147	0.00589777	4.802796367	1.5796996507	7.6369585764	4.268776273	ns
SRP14	-0.07021303	-0.01340821	-4.79383946	1.6515793404	7.9712589652	4.220277872	ns
KIF1C	-0.03741283	0.023050998	-4.78879445	1.6938564147	8.1618163880	4.203152907	ns
CHCHD6	-0.02904569	0.005997195	-4.78825165	1.6980010625	8.1683082414	4.188809626	ns
CEACAM19	-0.04024419	-0.03398322	-4.78679337	1.7103485932	8.2141741648	4.181948001	ns
STX4	-0.06965616	0.071075477	-4.77951884	1.7734869560	8.5034185740	4.152202604	ns
HPSE	-0.09724906	-0.08430357	-4.77906972	1.7774855015	8.5086191220	4.150526181	ns
SBSN	-0.03505306	0.002892931	-4.77331655	1.8287606688	8.7397400212	4.118398523	ns
TNFSF14	-0.06584437	-0.01293491	-4.76665337	1.8903760003	9.0194410472	4.09185426	ns
INPP5D	-0.06100195	0.010741509	-4.75844803	1.9689614027	9.3790657357	4.058495292	ns
GBP1	-0.06772518	-0.01883414	-4.7386651	2.1704413471	1.0321968621	3.954052965	ns
CLEC4A	0.038683998	0.005587358	4.735758185	2.2018985514	1.0454542715	3.943212671	ns
TDP1	-0.07430009	0.089321489	-4.7353801	2.2058669549	1.0456382318	3.938759157	ns
IL4R	-0.03818357	-0.01938261	-4.72108741	2.3666056573	1.1200143467	3.874316921	ns
SORBS1	-0.0407061	0.031570506	-4.71207003	2.4733966577	1.1686599094	3.827718091	ns
HCLS1	-0.06219302	-0.04580436	-4.71172899	2.4775220644	1.1687180013	3.826068959	ns
PAM	-0.03060165	-0.0376591	-4.71108818	2.4854634726	1.1705731193	3.826196493	ns
CD164	-0.0304889	0.011745166	-4.70918443	2.5088341967	1.1778732026	3.818270913	ns
PRDX3	-0.07832132	0.01579031	-4.70919929	2.5090312742	1.1778732026	3.826320776	ns
SLMAP	-0.08650854	0.053210249	-4.70353793	2.5790217230	1.2087870676	3.789572758	ns
ASAH2	-0.05981804	-0.07316103	-4.69142394	2.7366203101	1.2805979656	3.738537064	ns
C1QA	-0.02497419	9.8688376300	-4.6857062	2.8137144682	1.3145673995	3.705963622	ns
CD55	-0.02814473	-0.00149866	-4.67991352	2.8955488365	1.3506393934	3.701175276	ns
GLO1	-0.06447385	-0.01448294	-4.67291194	2.9952888543	1.3949351602	3.657689427	ns
ALPP	-0.12218479	-0.01356746	-4.66548097	3.1050383393	1.4437439412	3.617353135	ns
GRPEL1	-0.0649039	0.019964552	-4.66384291	3.1298349440	1.4529599422	3.609764243	ns
MNDA	-0.10894993	0.086793132	-4.65182508	3.3176371366	1.5376984823	3.553898657	ns
POF1B	-0.04424453	0.042908855	-4.6510623	3.3304193144	1.5411766082	3.558205788	ns
MANSC1	-0.02845087	-0.00295025	-4.64915449	3.3605867792	1.5526761701	3.537976874	ns
RNASEH2A	-0.05062621	0.083288805	-4.64751019	3.3872836316	1.5625384209	3.527625267	ns
IRAG2	-0.0963371	0.145537998	-4.64495332	3.4302968482	1.5784704894	3.528333029	ns
NOTCH3	0.036601525	0.005703887	4.644762689	3.4326327425	1.5784704894	3.515429796	ns
EEF1D	-0.09483144	0.01657469	-4.6433157	3.4567799896	1.5869889858	3.509061009	ns
LRIG3	-0.0279259	0.005890878	-4.64305884	3.4620273424	1.5869889858	3.522765366	ns

STX8	-0.05325463	0.031013476	-4.64245746	3.4714202153	1.5888004747	3.508693686	ns
CTHRC1	-0.02814044	-0.02702126	-4.63915222	3.5270583545	1.6117387160	3.490264838	ns
PDZD2	-0.06156459	0.09565729	-4.63823762	3.5435423366	1.6145599325	3.498445437	ns
CRYBB1	-0.06609161	0.013665339	-4.63814279	3.5442908107	1.6145599325	3.485216262	ns
CASC3	-0.04221814	0.029887722	-4.63696866	3.5651627069	1.6215381782	3.49179251	ns
CEACAM6	-0.039242	-0.00209125	-4.63530069	3.5932806443	1.6317853004	3.472524511	ns
BCAN	0.037553923	0.042684075	4.634108923	3.6144209501	1.6388368283	3.473116838	ns
GALNT3	-0.04598399	0.001683694	-4.62833039	3.7169179828	1.6802073106	3.451373193	ns
CCL14	-0.04268	-0.02449548	-4.62830252	3.7171709681	1.6802073106	3.447756095	ns
EDN1	-0.02849628	-0.02383583	-4.62094396	3.8516180873	1.7382882249	3.418116932	ns
BAP18	-0.06937305	0.013947613	-4.61893573	3.8881547942	1.7520697529	3.396960038	ns
TCOF1	-0.03054013	-0.00607701	-4.61708162	3.9230186196	1.7650561431	3.388407571	ns
GZMA	-0.03719079	-0.01291418	-4.61271352	4.0061936989	1.7997054770	3.367538761	ns
IQGAP2	-0.05893394	0.000275428	-4.60682428	4.1216369768	1.8487219619	3.347262879	ns
HSPA2	-0.03382983	0.008452284	-4.60323797	4.1928521803	1.8777804243	3.325913069	ns
THPO	-0.04087355	-5.06E-05	-4.59848253	4.2904151490	1.9185317358	3.315038751	ns
TXNDC5	-0.073201	0.024704223	-4.58642103	4.5443660570	2.0289830102	3.247433299	ns
IL7R	0.056948464	0.016943953	4.585654995	4.5613569607	2.0334598970	3.24806785	ns
LRRC59	-0.0462083	0.06827152	-4.58217624	4.6375356905	2.0642689354	3.228930877	ns
IGF2R	-0.02523157	-0.01476592	-4.57874904	4.7143390182	2.0952617859	3.216636558	ns
IFNGR1	-0.02129289	-0.00138398	-4.57753876	4.7415403302	2.1041485963	3.210425198	ns
PLXDC2	0.026169919	0.012825755	4.568883309	4.9411146900	2.1893861752	3.168562523	ns
HEPACAM2	0.041591922	0.026025487	4.568533069	4.9493659484	2.1897194802	3.166973066	ns
PSIP1	-0.06755458	0.035757826	-4.55939671	5.1697308228	2.2837540095	3.129118304	ns
GP1BA	-0.03437869	0.007094555	-4.55830817	5.1969232430	2.2922984697	3.128728926	ns
TNFAIP2	-0.04716745	0.023714261	-4.55543072	5.2677968616	2.3200553296	3.108526098	ns
SLITRK2	-0.04185515	-0.01402865	-4.54531408	5.5267608488	2.4304430239	3.060347325	ns
SRPK2	-0.08846524	0.120764505	-4.53727317	5.7418588037	2.5212372491	3.029169535	ns
NPY	-0.06936682	-0.09276635	-4.53676542	5.7551832676	2.5232935647	3.02178529	ns
C5	-0.01478654	-0.00710707	-4.53150638	5.9002401614	2.5830136838	2.999546305	ns
AHNAK2	-0.02889281	0.007986883	-4.5274017	6.0170543636	2.6302093924	2.993176665	ns
LCP1	-0.04731837	0.058434104	-4.52598235	6.0563246341	2.6434182259	2.975126587	ns
SEMA4D	-0.03129375	-0.00860303	-4.52084983	6.2047809658	2.7041731970	2.950833782	ns
FGFBP3	-0.03583903	-0.01857842	-4.51588312	6.3518439047	2.7641407156	2.929419229	ns
ERMAP	-0.04421839	-0.01702093	-4.5144795	6.3954497253	2.7789751782	2.936593472	ns
DBI	-0.08487203	-0.00851293	-4.5114214	6.4874476954	2.8147618530	2.915067235	ns
MSTN	-0.05458548	0.014857572	-4.50790032	6.5965749510	2.8578633318	2.905426683	ns
DTX3	-0.02418087	-0.02128341	-4.5074641	6.6093770418	2.8591675499	2.897525033	ns
PPP1R12A	-0.10113842	0.033457035	-4.49238419	7.0941445267	3.0643346180	2.826222803	ns
MTUS1	-0.05365815	0.001213783	-4.46452105	8.0823124957	3.4860195698	2.707473471	ns
THBS4	-0.04776539	-0.02449886	-4.45736752	8.3562258459	3.5988465295	2.674509643	ns
SCARF1	-0.04341479	-0.00530759	-4.45660981	8.3849260910	3.6058886871	2.66490931	ns
AMY2A	0.04988182	0.014897039	4.452746936	8.5408179725	3.6675277176	2.674897071	ns
EIF4G1	-0.10502992	-0.09664997	-4.44328019	8.9210399104	3.8251742347	2.605439631	ns
DNM3	-0.05288323	0.052783543	-4.4415619	8.9925337926	3.8501757587	2.597911404	ns
CST1	0.098334704	0.048081719	4.425305406	9.7060205942	4.1494094167	2.588242375	ns
NIT2	-0.07216714	-0.04011081	-4.4247878	9.7198494556	4.1494094167	2.526124329	ns
VNN1	-0.07112223	-0.10123829	-4.42246722	9.8245255675	4.1879729426	2.515690831	ns
SPINT1	-0.02455643	-0.01325497	-4.41989949	9.9426515063	4.2321490377	2.508528584	ns
MEGF9	-0.02155669	-0.00403363	-4.40962856	1.0424748072	4.4308972885	2.459977789	ns
KLRB1	-0.03753939	0.003513487	-4.40837409	1.0484839091	4.4499607774	2.451967959	ns
HNRNPUL1	-0.04824587	0.024157675	-4.4065124	1.0575244599	4.4818162889	2.44387165	ns
CBS	-0.04778386	0.053951592	-4.40210554	1.0794242476	4.5679982655	2.437565079	ns
SLAMF6	-0.03815148	-0.01333314	-4.39541888	1.1131684751	4.7039825576	2.408789442	ns
JAM3	-0.047248	0.039494943	-4.3911585	1.1351269816	4.7898421769	2.3889932	ns
VSIG2	-0.05123616	0.014193269	-4.38736795	1.1549052463	4.8662674159	2.361965063	ns
EIF2S2	-0.04052045	0.058394612	-4.38197462	1.1840301004	4.9817981172	2.349746858	ns
ABL1	-0.07128284	0.054459085	-4.37711948	1.2105535096	5.0860665443	2.322371393	ns
DTYMK	-0.06612344	-0.01380721	-4.37302325	1.2333721448	5.1744923317	2.298632274	ns
DNER	0.026768311	0.008813408	4.372448623	1.2367175114	5.1810834051	2.302042703	ns
TP53I3	-0.05092903	-0.00374249	-4.36744165	1.2656477263	5.2946867636	2.296599222	ns
ALDH3A1	-0.06451173	0.035957889	-4.36594056	1.2740688139	5.3173418590	2.272386384	ns
ADA2	-0.04175806	-0.01800107	-4.36584522	1.2747052401	5.3173418590	2.277037259	ns
PDCD5	-0.06105168	-0.00980959	-4.36547834	1.2766606331	5.3179016388	2.265044688	ns
MSMB	-0.07152411	-0.09660219	-4.35850989	1.3180869092	5.4826407049	2.243283585	ns
KLK8	-0.03556234	-0.02051705	-4.35488724	1.3402293697	5.5620602405	2.236535941	ns

TFF1	-0.07394644	0.023579233	-4.35472898	1.3409898662	5.5620602405	2.225475149	ns
BAG4	-0.04654904	0.03790814	-4.35019866	1.3691041628	5.6706158232	2.213399721	ns
RSPO1	-0.03499856	-0.0177006	-4.34921842	1.3750911378	5.6787902458	2.202099969	ns
TNFRSF21	-0.02607171	-0.00547418	-4.34901326	1.3762406122	5.6787902458	2.193771848	ns
RBKS	-0.05218776	0.009818999	-4.34895244	1.3769121554	5.6787902458	2.208392698	ns
KLRK1	-0.04260847	-0.05432116	-4.34212965	1.4200360771	5.8483855365	2.164940704	ns
SCRIB	-0.08715917	0.181398674	-4.33927753	1.4388209309	5.9174043919	2.164815814	ns
CD7	-0.04012871	-0.00790243	-4.33777549	1.4487196376	5.9497346582	2.160687699	ns
BECN1	-0.05854549	0.047563299	-4.3333499	1.4780804680	6.0617906836	2.139284641	ns
GPRC5C	-0.05159269	0.065085458	-4.33135448	1.4912935848	6.1074014974	2.119386782	ns
CCL5	-0.1048714	-0.11593381	-4.32890333	1.5080262577	6.1672782531	2.112437548	ns
SERPINH1	-0.12193834	-0.02301683	-4.32772872	1.5161320077	6.1917558918	2.110137065	ns
DNAJA2	-0.08407712	-0.04836276	-4.32579265	1.5293690972	6.2370918493	2.095665711	ns
FYB1	-0.09437015	-0.03554146	-4.31970927	1.5721750950	6.4027214472	2.07264259	ns
OTUD7B	-0.06008686	0.023676332	-4.31031371	1.6402738585	6.6707516253	2.027342021	ns
CLEC1B	-0.09729566	-0.0024357	-4.30883279	1.6512912010	6.7062173951	2.020969319	ns
CLUL1	0.045911252	0.035076086	4.302239438	1.7013039872	6.8997328373	1.999837082	ns
DYNLT1	-0.07470789	0.141053321	-4.2995093	1.7224939011	6.9759808478	1.994200002	ns
PPP1R9B	-0.09670721	0.021549955	-4.29003428	1.7972577849	7.2686879946	1.940649077	ns
ENO3	-0.05612934	0.053892122	-4.28457563	1.8422329888	7.4402770779	1.930528547	ns
ALCAM	-0.01861097	-0.00660483	-4.28266166	1.8578173053	7.4928543253	1.909804069	ns
SMAD1	-0.08276106	0.030859878	-4.28138736	1.8688258047	7.5268570343	1.917407318	ns
UBE2L6	-0.05803527	0.006833735	-4.28031099	1.8775430777	7.5515506710	1.900120343	ns
BLNK	-0.03542563	0.001154452	-4.27596271	1.9148716834	7.6910939691	1.895370516	ns
ADAM9	-0.02656542	-0.00934361	-4.26130248	2.0444210310	8.2001502894	1.820459282	ns
NRP1	0.027081973	-0.00772109	4.259883768	2.0574851671	8.2412300249	1.816746581	ns
ERBB2	-0.02423934	-0.025448	-4.25426616	2.1097169700	8.4388678801	1.791722614	ns
SLAMF8	-0.05201354	-0.09001592	-4.2514175	2.1370315574	8.5364324867	1.790399905	ns
LONP1	-0.07958494	0.009066422	-4.23507241	2.2983296032	9.1682000565	1.72106286	ns
RGCC	-0.07041826	-0.03088473	-4.23466918	2.3020627120	9.1705636004	1.706251145	ns
GGT1	-0.04815616	0.002610699	-4.23238686	2.3256948423	9.2520830242	1.701814361	ns
BNIP3L	-0.04089565	0.024587305	-4.2305935	2.3445606826	9.3144451611	1.70369572	ns
MPHOSPH8	-0.07261009	0.034927246	-4.22671902	2.3852018670	9.4630291462	1.685702025	ns
TCN1	-0.03095257	-0.00375252	-4.22468841	2.4067048827	9.5353843386	1.675455609	ns
EPS8L2	-0.029043	-0.01011755	-4.2196541	2.4607047702	9.7361218550	1.643484616	ns
CEBPB	-0.03666118	0.014006497	-4.2180386	2.4787688665	9.7943235321	1.649294094	ns
TNIP1	-0.08297861	0.017423636	-4.21013712	2.5665512300	0.000101275	1.604198033	ns
CD36	-0.04126921	0.029869116	-4.20528203	2.6226633947	0.000103349	1.597646777	ns
GLB1	-0.04023945	-0.00268415	-4.20042306	2.6792854442	0.000105438	1.570229444	ns
VWF	-0.07302773	-0.04195729	-4.19777384	2.7105382911	0.000106525	1.552195861	ns
MAP2	-0.04151001	0.062906109	-4.1834012	2.8876071737	0.000113331	1.492719276	ns
ESAM	-0.02942576	-0.00190025	-4.18130726	2.9142913984	0.000114225	1.483653994	ns
CD6	-0.05220519	-0.03912866	-4.17494163	2.9968971981	0.000117305	1.457249845	ns
PSMG3	-0.0510186	0.0163393	-4.17149484	3.0427242170	0.000118939	1.446962314	ns
MFGE8	-0.04204712	-0.00135325	-4.16631364	3.1125671738	0.000121507	1.425183928	ns
CLIP2	-0.11147812	-0.05597876	-4.16317749	3.1556249371	0.000123023	1.412526618	ns
S100A12	-0.0733918	-0.022183	-4.16083379	3.1883687181	0.000124134	1.408468958	ns
MB	-0.04978336	0.020739999	-4.1575139	3.2345807027	0.000125765	1.385089373	ns
CEP20	-0.07165838	0.010310323	-4.1555707	3.2627097729	0.00012669	1.390191653	ns
CYB5R2	-0.04997517	0.014019797	-4.15401401	3.2846779649	0.000127374	1.377379859	ns
FUS	-0.03910658	0.009529481	-4.150008	3.3429578542	0.000129462	1.36777411	ns
HSPA1A	-0.07018152	0.024582347	-4.14535415	3.4110536234	0.000131924	1.338487459	ns
CTSL	-0.02238587	-0.0219277	-4.14099885	3.4765073525	0.000134278	1.323066334	ns
ATOX1	-0.06977775	-0.06073943	-4.1304752	3.6392045101	0.000140376	1.276115164	ns
MME	-0.07662283	0.083099284	-4.12450838	3.7349072458	0.000143878	1.255639331	ns
IL32	0.039541127	0.009635475	4.123198582	3.7561345671	0.000144505	1.247962122	ns
CTBS	-0.02426914	-0.02082867	-4.11429176	3.9040977482	0.00015	1.215923499	ns
RPE	-0.06540159	0.079024553	-4.11344175	3.9184646867	0.000150354	1.211677204	ns
CNST	-0.10370584	-0.05921551	-4.11089574	3.9618245290	0.000151818	1.199671265	ns
SERPINA5	-0.02907401	0.018690177	-4.11056136	3.9676549769	0.000151842	1.200139778	ns
GADD45GIP1	-0.0467099	0.036425935	-4.10929272	3.9897089633	0.000152486	1.19976861	ns
MEP1A	-0.06302296	0.00702397	-4.1048203	4.0670646819	0.00015524	1.170122784	ns
NFATC3	-0.05729622	0.109977462	-4.10422878	4.0779649711	0.000155404	1.178417095	ns
KRT5	-0.04624685	0.027792833	-4.10380542	4.0854839916	0.000155404	1.176980538	ns
HSDL2	-0.04249727	0.00206624	-4.10370024	4.0873264294	0.000155404	1.17695465	ns
TNFRSF13C	-0.04021416	-0.02108308	-4.10143928	4.1273917829	0.000156723	1.167047584	ns

SNX15	-0.04928186	0.000995616	-4.09218502	4.2954120529	0.000162891	1.129291074	ns
MYDGF	-0.08983487	-0.03194386	-4.08987987	4.3377824873	0.000164284	1.108298062	ns
CLPP	-0.09131208	-0.01695133	-4.08280765	4.4722012683	0.000169156	1.087357036	ns
FABP6	0.051530682	0.047857735	4.081580154	4.4961200131	0.00016984	1.086766275	ns
CRACR2A	-0.08260263	0.108605813	-4.08027135	4.5215203097	0.000170579	1.082165318	ns
SPRR3	-0.05884437	0.035261931	-4.07694206	4.5859573157	0.000172787	1.055852875	ns
SNAP29	-0.08628893	-0.03306719	-4.07399334	4.6444746067	0.000174766	1.045906681	ns
F10	-0.02308901	-0.01711836	-4.07318379	4.6605727770	0.000175146	1.041655998	ns
NMNAT1	-0.0713103	0.037024758	-4.07157668	4.6931346751	0.000176143	1.040801384	ns
IL12RB1	-0.03466496	-0.02995799	-4.06198523	4.8902857954	0.000183307	1.007066257	ns
FNTA	-0.03134289	0.02094789	-4.06145949	4.9007527027	0.000183464	0.994139729	ns
ESM1	0.033347202	0.024057836	4.053426294	5.0722141331	0.00018964	0.966661989	ns
ELN	-0.02943248	-0.04060549	-4.05020612	5.1421793116	0.00019201	0.94785773	ns
C9	-0.04527603	-0.06124499	-4.04683132	5.2167847044	0.000194547	0.935350422	ns
TBC1D5	-0.07509604	0.097886446	-4.0406484	5.3563357968	0.000199496	0.913175745	ns
CALCOCO2	-0.0677493	0.032612566	-4.02443126	5.7392098929	0.000213484	0.856918574	ns
S100P	-0.05035817	0.033779393	-4.01866934	5.8812250021	0.000218488	0.833642515	ns
PLXNA4	-0.07708849	-0.03786337	-4.01828102	5.8903962521	0.000218551	0.823626985	ns
PALM3	-0.06643577	0.152686517	-4.01571027	5.9554982432	0.000220686	0.822945947	ns
AK2	-0.09330098	-0.03490324	-4.00825721	6.1454659490	0.000227437	0.779960656	ns
CDH2	-0.03304716	-0.04225964	-4.00747967	6.1660658200	0.00022791	0.782604538	ns
AMY1A_AMY1B	0.043938127	0.02286981	4.00303612	6.2845927755	0.000231998	0.788109089	ns
OMG	0.063072233	0.052601493	4.001379937	6.3270732992	0.000233271	0.758618102	ns
CLGN	-0.03791374	0.023910683	-3.999261	6.3842852998	0.000235083	0.756602129	ns
VTI1A	-0.05152714	0.054787506	-3.994102	6.5246719727	0.00023995	0.735324748	ns
IL1B	-0.05215619	0.051392367	-3.98994863	6.6401227402	0.000243889	0.721537133	ns
APOA2	0.041676347	-0.00634954	3.988635769	6.6759957438	0.000244898	0.703029455	ns
S100A13	-0.03037768	-0.01072207	-3.98151153	6.8800424726	0.000252067	0.687722817	ns
CD70	-0.04022652	-0.03548506	-3.98021687	6.9169288705	0.000253101	0.672375885	ns
STK11	-0.06732931	-0.00298081	-3.97979756	6.9297135962	0.000253251	0.679201639	ns
CD2AP	-0.059641	0.014470425	-3.9722818	7.1513339675	0.000261024	0.643073554	ns
MYO9B	-0.06208609	0.098124964	-3.96731029	7.3023968294	0.000266205	0.629464915	ns
P4HB	-0.03611585	-0.00640458	-3.96277409	7.4419296307	0.000270953	0.604402326	ns
PSG1	0.105718791	-0.18310122	3.961388007	7.4853781896	0.000272196	0.601092861	ns
ITGAM	0.028802696	0.011571648	3.960929583	7.4995315217	0.000272371	0.595961005	ns
RTN4IP1	-0.07821338	-0.01789122	-3.96011295	7.5248621932	0.000272718	0.588491042	ns
SEPTIN8	-0.02787074	-0.00304184	-3.96002337	7.5277691440	0.000272718	0.589498773	ns
ZBP1	-0.03448792	0.042093612	-3.95710933	7.6210747964	0.000275756	0.591496968	ns
DAAM1	-0.07170842	0.022401802	-3.9548791	7.6921860608	0.000277985	0.580449103	ns
PROS1	-0.02136034	-0.01725959	-3.95095893	7.8183648049	0.000282196	0.553305744	ns
CXADR	-0.04682052	0.043997185	-3.95038115	7.8375828363	0.00028254	0.554825496	ns
PRG3	-0.04227487	0.011750375	-3.94667423	7.9594266346	0.000286579	0.5369391	ns
IL22	-0.0563277	0.027189357	-3.94332741	8.0713372966	0.00029025	0.524344713	ns
CPQ	-0.03010505	-0.01981516	-3.93671658	8.2966072236	0.000297984	0.498686764	ns
LRRFIP1	-0.04254116	0.057675848	-3.93329731	8.4154623053	0.000301881	0.485093737	ns
PLG	-0.01904678	-0.0213721	-3.93191928	8.4647775111	0.000303278	0.491539027	ns
LRG1	-0.02162856	-0.00069428	-3.93028854	8.5212600918	0.000304927	0.472367351	ns
ODAM	0.049308067	0.080863638	3.929775602	8.5405456033	0.000305243	0.48314682	ns
NBN	-0.04707028	0.041310369	-3.92879847	8.5751249492	0.000306105	0.478406982	ns
DNAJC21	-0.03349181	0.045629235	-3.92655407	8.6544424401	0.000308559	0.457868565	ns
GCG	-0.1154127	0.020933821	-3.92600123	8.6754659747	0.000308931	0.468646723	ns
IL13RA1	-0.0205836	-0.00252625	-3.92164357	8.8331567310	0.000314163	0.445531611	ns
CXCL6	-0.07021648	-0.01521787	-3.91982181	8.8994662036	0.000316137	0.430691042	ns
CLEC5A	-0.02571092	-0.01994965	-3.91799889	8.9676763264	0.000318173	0.432346928	ns
TCL1A	-0.09140668	-0.04303184	-3.91570965	9.0530990183	0.000320814	0.42323603	ns
TARBP2	-0.07366391	0.019241948	-3.91113592	9.2265949886	0.000326566	0.40991388	ns
SNCA	-0.09831985	-0.03125633	-3.9083397	9.3336993199	0.000329956	0.398662076	ns
LAT	-0.07028256	-0.11615883	-3.90211306	9.5758775734	0.000338108	0.361869391	ns
WDR46	-0.03207979	0.006255983	-3.89737305	9.7652184789	0.000344377	0.345329158	ns
PINLYP	-0.05744951	-0.06601671	-3.89604586	9.8196337600	0.000345879	0.350999798	ns
FCRL6	-0.06180697	-0.06208882	-3.89265332	9.9573253907	0.000350306	0.329888559	ns
ADGRF5	-0.0238331	-0.02799286	-3.88828758	0.000101375	0.000356216	0.309243269	ns
PPP3R1	-0.03814352	-0.02268153	-3.8835836	0.000103362	0.00036276	0.29786507	ns
TNFRSF11B	-0.02587806	-0.02346092	-3.88001791	0.000104886	0.000367457	0.283220468	ns
TSC22D1	-0.05695795	0.051698312	-3.87985214	0.000104952	0.000367457	0.275855735	ns
L3HYPDH	-0.07524584	0.011406265	-3.87887085	0.000105376	0.000368502	0.27290323	ns

ATP6AP2	-0.03709768	0.028756029	-3.878513	0.000105544	0.000368647	0.284539569	ns
ECHDC3	-0.06089729	0.055146059	-3.86710158	0.000110587	0.000385799	0.227608149	ns
CXCL9	-0.05927844	0.030408137	-3.86660115	0.000110816	0.000386136	0.2283882	ns
CC2D1A	-0.06895287	0.043907301	-3.86570174	0.000111229	0.000387114	0.229022282	ns
REG4	-0.04180777	-0.02641816	-3.86302355	0.000112452	0.000390905	0.216597919	ns
CD63	-0.04697497	-0.05073654	-3.85648368	0.000115503	0.000401032	0.194510997	ns
AZI2	-0.04746582	0.067031324	-3.85550243	0.000115958	0.000402134	0.182270794	ns
LGALS3	-0.02679394	-0.02689129	-3.85408837	0.000116635	0.000404003	0.182405263	ns
KIF22	-0.10263993	-0.16132158	-3.85376549	0.000116799	0.00040409	0.189890749	ns
CA9	0.039154271	0.034974485	3.853438076	0.00011694	0.000404099	0.173659346	ns
WWP2	-0.04928625	0.033169945	-3.84947714	0.000118853	0.000410224	0.166834066	ns
ASAH1	-0.03938126	-0.00626588	-3.84865473	0.00011925	0.00041111	0.162081344	ns
CLSTN2	0.034727188	0.021206573	3.847503305	0.000119805	0.000412536	0.151597499	ns
AMOTL2	-0.04114507	0.101791668	-3.84154256	0.000122754	0.000422193	0.132502115	ns
MOCS2	-0.05615562	0.023097775	-3.84075408	0.000123157	0.000423079	0.138490531	ns
APLP1	0.045475417	0.060587433	3.839538103	0.000123762	0.00042466	0.127905109	ns
NCAM1	0.027486678	0.017233854	3.835687202	0.000125714	0.000430852	0.112020254	ns
PTPRC	-0.0191486	-0.00194468	-3.83448019	0.000126328	0.000432449	0.103195479	ns
NPTX1	0.035689121	0.025436673	3.827942964	0.000129731	0.000443577	0.081346232	ns
TRIM58	-0.07253421	0.022676065	-3.82738826	0.000130021	0.000444047	0.076726281	ns
LYPD3	0.029785026	0.004749544	3.825483979	0.000131032	0.00044698	0.073310702	ns
OMD	0.039209576	-0.00650596	3.813476375	0.00013756	0.000468698	0.025676174	ns
SIRPA	-0.05099563	-0.09106742	-3.8100717	0.000139469	0.000474649	0.014979416	ns
CPPED1	-0.06072406	-0.01339715	-3.80907194	0.000140027	0.000475995	0.00542031	ns
PTN	-0.04297601	-0.04440961	-3.80615017	0.000141705	0.000480855	0.007579602	ns
BGN	0.105231141	0.192235778	3.80601167	0.000141786	0.000480855	0.008329636	ns
TSPAN1	-0.04921728	0.154218891	-3.80462036	0.000142583	0.000482996	0.001795581	ns
PBLD	-0.04555641	0.022806931	-3.80130947	0.0001445	0.000488923	-0.01086456	ns
SLIT2	-0.04617484	-0.00611232	-3.79994243	0.000145299	0.000491057	-0.01602674	ns
CLC	-0.05880545	0.045408347	-3.79832634	0.00014624	0.000493664	-0.02848003	ns
BCL2	-0.04390653	0.027505554	-3.79780132	0.000146544	0.000494119	-0.03529634	ns
CIT	-0.03887251	0.040308686	-3.78632268	0.000153485	0.000516926	-0.06694817	ns
RNF41	-0.06037518	0.040599887	-3.7853045	0.000154117	0.000518457	-0.07031894	ns
ZNRD2	-0.05598931	0.042293767	-3.78485221	0.000154381	0.000518747	-0.08408271	ns
AP3B1	-0.06391774	0.032140695	-3.78353865	0.000155197	0.000520892	-0.08902231	ns
LAT2	-0.11210848	0.02581102	-3.77991919	0.000157475	0.00052793	-0.09749946	ns
FDX1	-0.05171329	0.029802993	-3.7792899	0.000157884	0.000528693	-0.09251134	ns
ARTN	-0.04080955	0.04621528	-3.77751795	0.000159007	0.000531844	-0.10073821	ns
BACH1	-0.0638538	0.030804346	-3.77494377	0.000160647	0.000536714	-0.11852803	ns
YTHDF3	-0.07407069	-0.01614724	-3.76984899	0.000163968	0.000547186	-0.1294928	ns
WARS	-0.03017341	-0.02033913	-3.76843712	0.000164887	0.000549622	-0.14324207	ns
GRHPR	-0.05243589	-0.00937699	-3.76484729	0.000167267	0.00055692	-0.16014449	ns
PGD	-0.0445196	-0.00138277	-3.76364478	0.000168073	0.000558966	-0.16464278	ns
FCN2	-0.03721255	-0.06413633	-3.76307484	0.000168467	0.00055964	-0.15820137	ns
RECK	0.016746978	0.005920219	3.757021726	0.000172595	0.000572702	-0.17675154	ns
CALCB	-0.03142038	0.028331241	-3.75606034	0.000173241	0.000574192	-0.19293466	ns
MECR	-0.07431108	-0.01244222	-3.75431245	0.000174471	0.000577613	-0.18686139	ns
PROC	-0.02237418	-0.0123426	-3.74896692	0.000178217	0.000589347	-0.21429002	ns
IDO1	-0.05193988	0.01788566	-3.74628821	0.000180139	0.000595029	-0.21676173	ns
LGALS8	-0.05373451	0.045095938	-3.74196754	0.000183244	0.0006046	-0.24635267	ns
TDRKH	-0.0751791	0.085080033	-3.74117642	0.00018384	0.000605884	-0.23618517	ns
IL1RL2	-0.03411415	-0.01005191	-3.74021214	0.000184545	0.00060752	-0.2400673	ns
MCFD2	-0.03816148	0.003837824	-3.72909611	0.000192862	0.000634185	-0.2868341	ns
AKR1B1	-0.06608816	-0.02913306	-3.72539775	0.000195721	0.000642862	-0.29384482	ns
HEXIM1	-0.0719064	-0.00232697	-3.71977703	0.000200109	0.000656539	-0.32395722	ns
SERPINE1	-0.05940314	-0.09002708	-3.71663644	0.000202602	0.000663971	-0.33969151	ns
LTA	-0.03469263	-0.01785238	-3.70961846	0.000208304	0.000681892	-0.36145648	ns
SYTL4	-0.05853548	0.093125142	-3.70872834	0.00020903	0.000683503	-0.36854207	ns
PFDN2	-0.04791571	0.000587926	-3.70815394	0.000209525	0.000684353	-0.35839617	ns
GDNF	-0.03233444	-0.0150574	-3.70706277	0.000210428	0.000686537	-0.36241595	ns
PRKAR2A	-0.06697275	-0.04797224	-3.70513157	0.000212017	0.000690949	-0.38091817	ns
MCEMP1	-0.0730991	0.065267171	-3.70392253	0.000213051	0.000693544	-0.37205933	ns
CD160	-0.03879634	0.001850212	-3.70066696	0.000215776	0.000701633	-0.39911832	ns
TACSTD2	0.026713824	0.012061232	3.699151321	0.000217165	0.000705363	-0.34900794	ns
LPCAT2	-0.03517718	0.042602369	-3.69824271	0.000217857	0.000706824	-0.40096523	ns
GLOD4	-0.0313295	-0.01595977	-3.69753797	0.000218462	0.000708	-0.40329441	ns

ATP5IF1	-0.095632	-0.08554101	-3.69457918	0.000221013	0.000715474	-0.41786536	ns
AP1G2	-0.06544459	0.03947534	-3.69138077	0.000223807	0.000723717	-0.43137885	ns
RAB27B	-0.06616649	-0.14291056	-3.68920959	0.000225722	0.000729102	-0.44029264	ns
GIP	-0.04124774	0.075396273	-3.68848223	0.000226388	0.000730447	-0.43027424	ns
RAB10	-0.04315909	0.033676488	-3.68525931	0.000229249	0.00073886	-0.45476781	ns
PSME2	-0.03229971	-0.00070037	-3.68469944	0.000229767	0.000739714	-0.44830562	ns
KRT8	-0.04863907	0.035507696	-3.67719327	0.000236634	0.00076098	-0.47205239	ns
NOS1	-0.04484848	-0.01122511	-3.67665633	0.000237132	0.000761744	-0.47395609	ns
LY9	-0.02471911	-0.01034892	-3.67635328	0.000237408	0.000761792	-0.47716901	ns
MAVS	-0.08102374	0.080756009	-3.67430621	0.000239325	0.0007671	-0.48166693	ns
CEP170	-0.06278371	0.081483561	-3.67091512	0.000242518	0.000776482	-0.4945036	ns
PREB	-0.02517379	0.001283572	-3.67015104	0.000243242	0.000777947	-0.49800004	ns
TNFAIP8L2	-0.05011243	0.055873011	-3.66981209	0.000243562	0.000778119	-0.4993352	ns
PARK7	-0.04590923	-0.00786113	-3.66917829	0.000244154	0.000779157	-0.51005157	ns
DAB2	-0.09679148	-0.10293728	-3.66774768	0.000245514	0.000782644	-0.52029971	ns
AMY2B	0.042040147	0.02244243	3.667283457	0.000246043	0.000783474	-0.47764792	ns
GCNT1	-0.02915954	-0.01039401	-3.66456008	0.000248601	0.000790008	-0.52418465	ns
EIF2AK2	-0.08746306	0.030491877	-3.66451803	0.000248636	0.000790008	-0.52794905	ns
VSTM1	-0.04213352	-0.00777882	-3.66297806	0.000250154	0.000793967	-0.52383313	ns
MARCO	-0.02271081	-0.03160433	-3.65885568	0.000254195	0.000805916	-0.54522862	ns
NPL	-0.03556693	-0.04347188	-3.65378702	0.000259254	0.000821064	-0.56953999	ns
STC2	-0.02194441	-0.00342332	-3.65071148	0.000262382	0.000830072	-0.57721711	ns
PRRT3	-0.02929385	0.015882938	-3.65000296	0.000263123	0.000831515	-0.57056419	ns
STXBP1	-0.06162573	-0.04055021	-3.64866004	0.000264475	0.000834885	-0.58898432	ns
DRAXIN	-0.03600238	0.000665371	-3.64719174	0.000266004	0.000838803	-0.5865982	ns
TFRC	-0.03290128	0.008120676	-3.64490947	0.000268368	0.000845345	-0.59756937	ns
IL15RA	-0.02924972	-0.0277225	-3.64112118	0.00027236	0.000856996	-0.60344808	ns
STX3	-0.03061723	0.064155683	-3.6395332	0.000274021	0.000861292	-0.62115072	ns
CDSN	-0.03592883	-0.00954196	-3.6387344	0.000274871	0.000863035	-0.62426885	ns
RP2	-0.03611658	0.025341801	-3.63601598	0.000277796	0.000871282	-0.62772509	ns
ROBO1	-0.01963037	-0.00732379	-3.63044243	0.000283856	0.000889333	-0.64933075	ns
F2R	-0.04919173	-0.01192922	-3.62841604	0.000286089	0.00089537	-0.65756237	ns
TXLNA	-0.07619051	-0.04624047	-3.62497528	0.000289928	0.000905871	-0.66687203	ns
DSCAM	-0.02737764	0.00866266	-3.62484012	0.000290065	0.000905871	-0.67507663	ns
SAMD9L	-0.06239717	0.019324208	-3.62245663	0.00029276	0.000913311	-0.67899741	ns
HNRNPK	-0.06383232	0.051842254	-3.61761029	0.000298312	0.000929637	-0.68584474	ns
BCR	-0.07738521	-0.03220914	-3.61720184	0.000298761	0.000930044	-0.69790036	ns
LMNB1	-0.04672498	-0.01662526	-3.61553556	0.000300683	0.00093503	-0.70538075	ns
RAB44	-0.03449036	0.038410105	-3.61143291	0.000305475	0.000948921	-0.72148955	ns
MAGED1	-0.04038765	0.037134512	-3.61012849	0.00030704	0.00095277	-0.7139277	ns
EHBP1	-0.05984191	0.041242695	-3.60720381	0.000310488	0.000962448	-0.73761458	ns
SOD2	-0.03071118	0.000823853	-3.60550954	0.000312527	0.00096774	-0.74019335	ns
CR1	-0.02816125	-0.00605057	-3.60461104	0.000313607	0.000970055	-0.74356316	ns
CCL26	-0.07561829	0.034622279	-3.60412593	0.000314197	0.000970851	-0.74481917	ns
SKAP2	-0.08895222	-0.01939159	-3.60377336	0.000314619	0.000971127	-0.74716842	ns
CDC42BPB	-0.08229705	0.060279564	-3.60341114	0.000315054	0.000971444	-0.75017674	ns
RALY	-0.03862401	0.029304294	-3.60030294	0.000318842	0.000982087	-0.76137457	ns
CGREF1	-0.03519279	-0.01338478	-3.59566494	0.000324582	0.000998713	-0.77300316	ns
SERPINA1	-0.00613119	-0.00429912	-3.59456362	0.000325965	0.001001913	-0.77407027	ns
BAX	-0.07478437	0.023373501	-3.59400255	0.000326677	0.001002774	-0.77187798	ns
SERPINB1	-0.07629384	0.019040598	-3.59357459	0.000327197	0.001002774	-0.78090851	ns
IPCEF1	-0.0765666	-0.05433919	-3.59351496	0.000327275	0.001002774	-0.77884396	ns
FEN1	-0.05436509	0.090077812	-3.59254728	0.000328506	0.00100549	-0.77684168	ns
CCN1	-0.06467639	-0.1504574	-3.59206052	0.000329104	0.001006266	-0.78501608	ns
CHMP6	-0.04420476	-0.00209713	-3.59015629	0.000331501	0.001012535	-0.79858479	ns
PSAPL1	-0.0381526	0.009602024	-3.57546684	0.000350669	0.001069963	-0.84986161	ns
CD99	-0.0158141	-0.0158305	-3.57497385	0.000351337	0.00107088	-0.849143	ns
EPCAM	0.083415423	0.117250887	3.57406411	0.000352562	0.001073495	-0.85014989	ns
ADGRE1	-0.04218459	0.019804601	-3.57348876	0.000353328	0.001074706	-0.85688857	ns
DLG4	-0.05617674	0.079126732	-3.57295599	0.000354045	0.001075766	-0.85976283	ns
MMP10	-0.05018256	0.044186699	-3.5704328	0.000357485	0.00108509	-0.86223234	ns
DNAJB14	-0.05043781	0.022745037	-3.5645034	0.000365644	0.001108704	-0.88875954	ns
IFNGR2	-0.04326255	0.106773127	-3.5579608	0.000374878	0.001135523	-0.9060467	ns
ANXA3	-0.05864008	-0.01449547	-3.55300805	0.000381985	0.001155852	-0.93052319	ns
SPTLC1	-0.02887446	0.044138998	-3.55046269	0.000385694	0.001165866	-0.93938468	ns
LGALS4	-0.04353821	-0.03602431	-3.54978084	0.000386707	0.001167718	-0.93584455	ns

CLEC11A	-0.03862134	-0.03148448	-3.54550833	0.000393024	0.001185569	-0.95231076	ns
TIMM10	-0.03075004	0.043848896	-3.54348758	0.000396034	0.001193416	-0.9640196	ns
CTF1	-0.06994214	0.019650402	-3.54136314	0.000399267	0.001201917	-0.95898304	ns
GP2	0.070623286	0.113621163	3.538766193	0.000403229	0.001212595	-0.96206194	ns
IL15	-0.02667157	-0.02098871	-3.52512739	0.000424513	0.001273877	-1.02467253	ns
POSTN	0.028200784	0.005027423	3.524710409	0.00042517	0.001273877	-1.02955126	ns
DCTN6	-0.05909298	0.086028779	-3.52467765	0.000425256	0.001273877	-1.01771343	ns
CCN4	-0.03275671	0.020350445	-3.52459445	0.000425353	0.001273877	-1.03193872	ns
PLXNB3	-0.03862099	0.018616671	-3.52371796	0.000426784	0.001276852	-1.02564983	ns
LILRA2	-0.03021213	-0.03608417	-3.52273123	0.000428364	0.001280268	-1.03339651	ns
SPART	-0.06043023	0.012599756	-3.52166069	0.000430114	0.001284185	-1.02867928	ns
DPP7	-0.04858115	-0.0288278	-3.51874143	0.000434864	0.001296031	-1.04260041	ns
IDI2	-0.03808691	0.025150723	-3.51868517	0.000434969	0.001296031	-1.0386841	ns
SOST	-0.03202144	-0.03158784	-3.51496524	0.000441084	0.001312912	-1.05763927	ns
FLI1	-0.09088525	0.050508276	-3.51455132	0.000441792	0.001313097	-1.05225098	ns
METAP2	-0.04800802	-0.05762615	-3.51437568	0.000442046	0.001313097	-1.06690394	ns
PTPRN2	-0.0257577	-0.00382663	-3.51216552	0.000445761	0.001322786	-1.06435936	ns
DCBLD2	-0.02738839	-0.02543758	-3.51111009	0.000447513	0.001326637	-1.07542473	ns
GCC1	-0.08332709	-0.0693852	-3.50444768	0.000458847	0.001358496	-1.10070481	ns
TANK	-0.05707083	0.03529172	-3.50426765	0.00045919	0.001358496	-1.08929523	ns
FLT4	-0.02819369	-0.05505408	-3.50169149	0.000463621	0.001370215	-1.10824696	ns
PAFAH1B3	-0.09131475	0.44885687	-3.49984089	0.000466841	0.001378338	-1.11763265	ns
SELP	-0.04665454	-0.00719233	-3.49771691	0.000470586	0.001387992	-1.11945443	ns
MTIF3	-0.06589955	-0.00924784	-3.48930208	0.000485631	0.001430922	-1.15235671	ns
IL1R1	-0.01752725	-0.01258122	-3.48798748	0.000488035	0.001436555	-1.15341907	ns
MUC13	-0.03950222	-0.03085114	-3.48644764	0.000490875	0.001443459	-1.15088801	ns
CNDP1	0.036943607	-0.0301518	3.485644789	0.000492323	0.001446262	-1.16134594	ns
FBP1	-0.06061947	-0.01463605	-3.48385204	0.000495634	0.001454524	-1.16633255	ns
LRPAP1	-0.04697906	0.08690824	-3.48151161	0.000499964	0.001465759	-1.18125367	ns
MYOM3	0.069652567	0.000596725	3.477800164	0.000506966	0.001484573	-1.18121538	ns
CXCL11	-0.06302867	0.023056925	-3.47755358	0.000507399	0.001484573	-1.19494173	ns
LIFR	0.018965505	-0.0012579	3.476035828	0.000510291	0.001491543	-1.1954605	ns
PRELP	-0.01813792	-0.02761078	-3.47529504	0.000511704	0.001494174	-1.19585504	ns
OLFM4	-0.09371907	-0.15120375	-3.47061778	0.000520766	0.001519117	-1.18858668	ns
LTA4H	-0.04704852	0.041349871	-3.46800577	0.000525757	0.001532147	-1.22858264	ns
SPINK4	-0.05019296	0.028102072	-3.4669084	0.000527923	0.001536924	-1.22695089	ns
COQ7	-0.03321397	0.063308906	-3.46462265	0.000532452	0.001548565	-1.22585208	ns
SPRY2	-0.07518603	0.051050357	-3.45938419	0.000542906	0.001577398	-1.24459108	ns
TXNRD1	-0.03823662	0.014519003	-3.45769824	0.000546286	0.001585641	-1.25897642	ns
MDH1	-0.03073129	0.01677717	-3.45220344	0.000557523	0.001616652	-1.27726404	ns
SRPX	0.026142806	-0.02342491	3.451018471	0.000559965	0.00162212	-1.28542794	ns
CEMIP2	-0.02351815	0.00662168	-3.44697091	0.00056846	0.001644913	-1.28494258	ns
CTSB	0.04401915	0.074758993	3.446719417	0.00056896	0.001644913	-1.29458497	ns
CAMSAP1	-0.0827807	0.043670477	-3.44373232	0.000575258	0.001661477	-1.31124172	ns
USP8	-0.07677543	0.046545447	-3.44121064	0.000580688	0.001675504	-1.30644605	ns
APOC1	0.027517362	0.016069799	3.435949834	0.000592032	0.00170655	-1.33543567	ns
PRKAB1	-0.04369714	0.058418285	-3.43560205	0.000592829	0.001707159	-1.32606669	ns
NENF	-0.0851453	0.381918551	-3.43513693	0.000593811	0.00170723	-1.33881281	ns
SPAG1	-0.04348933	0.053027227	-3.4350369	0.000594022	0.00170723	-1.34096664	ns
RABEP1	-0.05888046	0.025085284	-3.43349214	0.000597454	0.001715404	-1.33336984	ns
RBPMS2	-0.0784138	0.010229808	-3.43278731	0.000599015	0.001718196	-1.33495449	ns
CDHR1	-0.0339042	0.027509691	-3.42727337	0.000611304	0.001751724	-1.35416293	ns
AHCY	-0.05387969	-0.01930193	-3.42695719	0.00061199	0.001751971	-1.36171233	ns
STIP1	-0.05351518	0.019212221	-3.42439533	0.000617782	0.00176682	-1.3719559	ns
HS1BP3	-0.06759578	-0.01056884	-3.42404897	0.000618596	0.001767418	-1.36472564	ns
PRKRA	-0.05450531	0.022198325	-3.41882719	0.000630584	0.001799908	-1.3820112	ns
PON1	0.019009521	-0.00682404	3.415613743	0.000638019	0.00181935	-1.40514358	ns
KLRF1	-0.03082588	0.007081496	-3.41183019	0.000646982	0.001843109	-1.40469728	ns
MKI67	-0.02912317	0.045300991	-3.40945926	0.000652627	0.00185738	-1.41273297	ns
SOD1	-0.03799961	0.008830319	-3.40859182	0.000654711	0.001861497	-1.41471438	ns
MTSS1	-0.08369184	-0.00883268	-3.40722202	0.000657949	0.001868882	-1.4336603	ns
CORO1A	-0.07091283	0.098387256	-3.40412277	0.00066551	0.001888523	-1.4282992	ns
NEDD9	-0.02498924	0.020931097	-3.40320183	0.000667748	0.00189295	-1.4339142	ns
PSRC1	-0.04687976	0.098925761	-3.40294799	0.000668367	0.00189295	-1.43583433	ns
APOL1	-0.04460671	0.088976768	-3.39879026	0.000678552	0.001919935	-1.46211527	ns
CRISP2	0.045733883	-0.04755949	3.396885377	0.000683285	0.001931455	-1.47121707	ns

PRUNE2	-0.02174993	0.013648507	-3.3911896	0.000697663	0.001970191	-1.48123211	ns
GYS1	-0.07340902	0.009472521	-3.39076828	0.000698759	0.001971379	-1.47767934	ns
WFDC12	-0.05061128	-0.06381056	-3.38977151	0.000701254	0.001976508	-1.49404471	ns
PQBP1	-0.04581296	0.044443246	-3.38406967	0.000716022	0.002016185	-1.49949757	ns
MAP3K5	-0.07936501	0.119041837	-3.38151998	0.000722695	0.002033015	-1.50806877	ns
CNP	-0.05925617	-0.0023474	-3.37191533	0.000748353	0.002103168	-1.53973554	ns
LIF	-0.04088448	0.014678149	-3.37018923	0.000753051	0.002114336	-1.54696551	ns
ERBIN	-0.08187075	-0.05398046	-3.36330215	0.00077205	0.002165595	-1.57620833	ns
USP25	-0.05364095	0.006409947	-3.35803968	0.000786869	0.00220329	-1.59908585	ns
FABP2	0.061600074	0.057176035	3.358000925	0.000786997	0.00220329	-1.59420587	ns
MLN	-0.07040462	-0.0711332	-3.35467183	0.000796517	0.002227806	-1.60741807	ns
SIGLEC7	-0.02198297	-0.03691398	-3.3501225	0.00080971	0.002262539	-1.62046848	ns
ARHGEF1	-0.06604271	-0.03548987	-3.34255734	0.000832066	0.002321384	-1.65156233	ns
ANXA1	-0.03149431	-0.02239906	-3.3424791	0.000832359	0.002321384	-1.63678726	ns
SUSD1	-0.03799053	-0.0524417	-3.34185408	0.000834232	0.002324388	-1.64131403	ns
SIRT2	-0.07016154	-0.02350642	-3.34086262	0.000837184	0.002330388	-1.65096579	ns
FKBP4	-0.03471206	-0.02131707	-3.33768233	0.000846821	0.002354969	-1.66082589	ns
MAP2K1	-0.04678231	0.02138386	-3.33517288	0.000854469	0.002373695	-1.67507577	ns
ATG16L1	-0.06250904	-0.00849424	-3.33494141	0.00085518	0.002373695	-1.67584305	ns
NID1	-0.02962902	-0.02001742	-3.33466276	0.00085606	0.002373878	-1.67149889	ns
GOLGA3	-0.05641621	0.05655925	-3.33396784	0.000858169	0.002377471	-1.68096227	ns
NACC1	-0.04126408	0.03640718	-3.33226179	0.000863454	0.002389843	-1.68479365	ns
COMMD1	-0.0449949	0.020862042	-3.32448918	0.000887919	0.002453381	-1.69857232	ns
AIF1	-0.04059869	-0.01505848	-3.32443684	0.00088809	0.002453381	-1.69827425	ns
CEBPA	-0.02803854	0.048671643	-3.31757876	0.000910167	0.002511993	-1.71984291	ns
HS6ST1	-0.02541964	0.001433456	-3.31474251	0.000919377	0.002535016	-1.74520851	ns
PCSK9	-0.03064364	-0.02306852	-3.3117927	0.00092912	0.002559463	-1.75368325	ns
PTTG1	-0.02968376	0.007710266	-3.31133825	0.000930663	0.002561298	-1.74718793	ns
YARS1	-0.10609824	-0.18103958	-3.30714967	0.000944654	0.002597354	-1.76758169	ns
ERP29	-0.08552453	-0.04564359	-3.30623903	0.000947773	0.002602321	-1.75952801	ns
PDIA3	-0.01856957	-0.00153876	-3.30591123	0.000948882	0.002602321	-1.76060501	ns
CACYBP	-0.07445975	-0.06890145	-3.30583793	0.000949134	0.002602321	-1.7598265	ns
MZT1	-0.03991304	0.068944765	-3.30453931	0.00095355	0.002611975	-1.76381443	ns
PCYT2	-0.05488599	-0.02545529	-3.30008762	0.000968733	0.002651078	-1.79077087	ns
WFIKKN1	-0.03118635	0.013343391	-3.29818757	0.000975344	0.00266667	-1.78946279	ns
CHI3L1	-0.05631661	0.00532414	-3.29757198	0.000977529	0.002670146	-1.78163651	ns
ERI1	-0.02448002	0.003819022	-3.29590896	0.000983275	0.002683331	-1.79784781	ns
SDCCAG8	-0.08329034	-0.01535221	-3.29172274	0.000997991	0.002720945	-1.81824601	ns
ITGB1	0.016522651	0.010320345	3.288972247	0.001007816	0.00274517	-1.82079424	ns
ADA	-0.02683328	0.013489919	-3.28761554	0.001012641	0.002754148	-1.83356032	ns
PAPPA	-0.03530476	-0.02010484	-3.28753373	0.001012998	0.002754148	-1.82071226	ns
KLK13	-0.03331887	-0.01980778	-3.28522527	0.00102135	0.00277427	-1.82503872	ns
IL31RA	0.028739319	0.021292701	3.284643179	0.001023462	0.002777424	-1.82609516	ns
PTPRB	-0.02084204	-0.0190152	-3.28402187	0.00102564	0.00278075	-1.84438213	ns
NFYA	-0.02812667	0.041567128	-3.28202752	0.001032986	0.002798069	-1.83707216	ns
CIRBP	-0.06868798	-0.07475311	-3.28066172	0.001037931	0.002808594	-1.85430309	ns
SMPD1	-0.03688637	-0.01653308	-3.28026126	0.001039408	0.002808594	-1.85552087	ns
SWAP70	-0.02582271	0.015111235	-3.28016555	0.001039757	0.002808594	-1.85592088	ns
LAP3	-0.03723293	-0.01718026	-3.27691418	0.00105185	0.002838635	-1.8553524	ns
CDNF	-0.02330001	-0.00268305	-3.27603206	0.001055109	0.0028448	-1.86414184	ns
SMTN	-0.07029561	0.163001878	-3.27464221	0.001060283	0.002856114	-1.87422485	ns
DAPK2	-0.03842946	0.045879284	-3.27190011	0.001070681	0.002881464	-1.86940598	ns
MSRA	-0.05580029	-0.01907864	-3.27107755	0.001073735	0.002887022	-1.88443112	ns
PRKAR1A	-0.06422133	-0.03534093	-3.26829642	0.001084355	0.002912895	-1.88959217	ns
EFHD1	-0.0239419	-0.0017446	-3.26775291	0.001086405	0.002915719	-1.89821191	ns
ATRN	-0.01199744	-0.01673262	-3.26489254	0.001097432	0.00294261	-1.9066771	ns
ANXA11	-0.05114896	-0.13165385	-3.25898018	0.001120568	0.003001889	-1.92192709	ns
HBEGF	-0.05606205	-0.02610994	-3.25154693	0.001150244	0.003078562	-1.95014911	ns
RCC1	-0.03574895	0.097805566	-3.24865799	0.001161985	0.003107139	-1.9582261	ns
CCL27	-0.04538956	-0.07629135	-3.24822692	0.001163769	0.003109062	-1.95458536	ns
PLEKHO1	-0.04987263	0.046256373	-3.24729537	0.001167551	0.003116315	-1.96354998	ns
AXIN1	-0.07305843	-0.02693033	-3.24594928	0.001173107	0.003127787	-1.9640526	ns
EIF4EBP1	-0.04995933	0.012086571	-3.24573604	0.001173991	0.003127787	-1.96262398	ns
AGRP	-0.03972686	-0.06768214	-3.24494198	0.001177231	0.00313356	-1.97210665	ns
PDIA4	-0.04291289	0.038371811	-3.23791589	0.001206598	0.003208805	-1.99340113	ns
ENG	0.012867617	0.000188558	3.233966285	0.001223393	0.003250508	-2.00686672	ns

VPS37A	-0.06100212	0.032096605	-3.23296399	0.001227726	0.003259055	-2.00345234	ns
MTHFSD	-0.07946573	0.005947298	-3.2315447	0.001233841	0.003272312	-2.00663017	ns
COX6B1	-0.02258823	0.015733357	-3.22962016	0.001242133	0.003291313	-2.02049282	ns
CDKN1A	-0.08123488	-0.04700679	-3.22300988	0.00127117	0.0033652	-2.03538859	ns
GPI	-0.03300022	0.021123681	-3.22058595	0.00128193	0.003390612	-2.0483463	ns
DNAJB2	-0.03895919	0.015920004	-3.21862718	0.001290771	0.003410906	-2.04359113	ns
PTPRF	-0.02128766	-0.01171792	-3.21674799	0.001299184	0.003430034	-2.06206873	ns
PTH	-0.0560044	-0.00385061	-3.21559557	0.001304479	0.003440901	-2.05245907	ns
QSOX1	-0.02928749	0.00675125	-3.21437317	0.001310027	0.003452418	-2.06004309	ns
TFPI	-0.02411753	-0.02593067	-3.21322276	0.001315276	0.003460156	-2.06453902	ns
SNX5	-0.02071873	0.045764639	-3.21321625	0.001315333	0.003460156	-2.06005582	ns
LHPP	-0.04537394	0.026361004	-3.20725219	0.001342839	0.003529335	-2.08823114	ns
TPD52L2	-0.07564113	-0.09761639	-3.20207003	0.001367257	0.00359028	-2.09641838	ns
VEGFD	0.027235975	0.020503335	3.200607305	0.001374146	0.003605128	-2.1133944	ns
SNU13	-0.04543236	0.150081495	-3.19951221	0.001379461	0.003615822	-2.10283008	ns
NIT1	-0.03350478	-0.06819289	-3.19854366	0.001384091	0.003623429	-2.10680454	ns
CAMKK1	-0.03370104	0.027057884	-3.19838797	0.001384845	0.003623429	-2.10682554	ns
BAMBI	-0.02130033	0.002512311	-3.19780901	0.001387626	0.003627456	-2.10866568	ns
STX6	-0.0516991	0.130004856	-3.19727734	0.001390185	0.003630894	-2.11035524	ns
PDLIM7	-0.09599309	0.018858306	-3.19344052	0.001408709	0.003675986	-2.13320054	ns
SLC9A3R1	-0.05890824	0.006415534	-3.18253672	0.001462859	0.003813883	-2.15839152	ns
THTPA	-0.06104557	0.001931599	-3.18113454	0.0014699	0.00382882	-2.17306655	ns
FXN	-0.05421563	-0.00255013	-3.17838857	0.001483867	0.003861756	-2.18523751	ns
KIR2DS4	-0.11899851	0.940693841	-3.1766838	0.001492621	0.003878479	-2.18799356	ns
NAA80	-0.08391221	0.011217804	-3.17663318	0.001492949	0.003878479	-2.17622068	ns
MAPK9	-0.03157435	-0.01240264	-3.17484255	0.001502141	0.00389889	-2.19095282	ns
CEP85	-0.04734763	0.061124103	-3.17425105	0.001505263	0.003903524	-2.18325964	ns
ZFYVE19	-0.06208407	-0.00220973	-3.17276616	0.001512895	0.003919833	-2.20035642	ns
ING1	-0.03433644	0.014180341	-3.17205478	0.001516688	0.003926178	-2.18995477	ns
NSFL1C	-0.0498665	-0.01148979	-3.16765762	0.001539704	0.003982227	-2.21838992	ns
SERPINI2	0.038396232	0.090164688	3.16661043	0.001545263	0.00399307	-2.21975122	ns
VPS53	-0.05835037	0.062721379	-3.16573273	0.001549962	0.004001671	-2.21724236	ns
VAMP8	-0.06098251	0.008701051	-3.16448074	0.001556695	0.004015503	-2.21296293	ns
CACNB3	-0.058031	-0.01162826	-3.16242568	0.001567619	0.004040113	-2.23482063	ns
CPB2	-0.01556451	-0.01355439	-3.16179725	0.001571016	0.004045298	-2.23435455	ns
IRAK1	-0.0518136	0.040088056	-3.15781751	0.001592678	0.004097463	-2.23617051	ns
CEACAM21	-0.06337151	0.246080803	-3.15454547	0.001610586	0.004139886	-2.25479963	ns
VAT1	-0.01427279	0.004499444	-3.14914207	0.001640656	0.004213469	-2.26930913	ns
FOXJ3	-0.03051175	0.039407395	-3.14704498	0.001652516	0.004238942	-2.26764705	ns
PHACTR2	-0.07876359	-0.03393942	-3.14685683	0.001653478	0.004238942	-2.28364191	ns
RAB6A	0.034088845	0.055474091	3.144360977	0.001667674	0.004271587	-2.28668204	ns
CASP1	-0.0500787	-0.00098782	-3.14029467	0.001691042	0.004327645	-2.29070327	ns
PRDX6	0.044364817	0.071347133	3.138264945	0.001702694	0.004350279	-2.31120621	ns
IL7	-0.04743355	0.010567027	-3.13824149	0.001702866	0.004350279	-2.30578949	ns
LYN	-0.0634544	-0.06077442	-3.13742224	0.001707639	0.004358658	-2.30592501	ns
CLEC1A	-0.02302623	-0.0125372	-3.13013259	0.001750612	0.0044639	-2.32221393	ns
MATN2	-0.02146371	-0.00991168	-3.12990114	0.001751928	0.0044639	-2.33086775	ns
VSIR	-0.0448286	0.061948223	-3.12950038	0.001754328	0.004466118	-2.33089201	ns
TIGAR	-0.03427121	-0.02390478	-3.12821261	0.001762089	0.004481968	-2.32721426	ns
SPRR1B	-0.05852315	0.165800298	-3.12754349	0.00176605	0.004488135	-2.3352955	ns
FARSA	-0.04774018	0.118898617	-3.12528911	0.001779667	0.004518806	-2.33701089	ns
RNASE10	-0.03259276	-0.00605866	-3.11633418	0.001834585	0.004654204	-2.36478168	ns
EBAG9	-0.06957916	-0.01514031	-3.1158785	0.001837377	0.004657241	-2.37217649	ns
CD48	-0.02107473	-0.02350429	-3.11341028	0.00185278	0.00469221	-2.38298036	ns
PPCDC	-0.03510794	0.011964808	-3.11214664	0.001860697	0.004708176	-2.39165061	ns
CXCL3	-0.06985826	-0.09518409	-3.11172083	0.001863423	0.004710991	-2.38608586	ns
KRT19	-0.04666185	0.016545685	-3.10995997	0.00187456	0.004735048	-2.39271942	ns
BIN2	-0.06705067	0.032309115	-3.10839938	0.00188447	0.004755966	-2.39877479	ns
NUDT16	-0.05927757	0.000502154	-3.10754364	0.001889917	0.004765594	-2.40340128	ns
MIF	-0.05013215	-0.04617923	-3.10559627	0.001902421	0.004792985	-2.40650561	ns
ABRAXAS2	-0.04507035	0.023782127	-3.10472637	0.00190809	0.004803124	-2.39976825	ns
LRP1	0.019387706	0.014044627	3.103283245	0.001917352	0.004822281	-2.4118073	ns
INPPL1	-0.07122511	-0.01308788	-3.10030452	0.001936729	0.004866823	-2.42099097	ns
PTPRK	-0.01445348	0.011105959	-3.09889157	0.001945945	0.004885779	-2.43062928	ns
PLB1	0.040273702	-0.00954359	3.098620722	0.001947716	0.004886022	-2.43246648	ns
ACOT13	-0.07613322	-0.05739855	-3.09771098	0.001953709	0.00489685	-2.43426532	ns

CNPY4	-0.05521404	-0.02078161	-3.09714006	0.001957511	0.004898359	-2.43073759	ns
SNAP23	-0.06369518	-0.11194088	-3.09711619	0.001957666	0.004898359	-2.43110568	ns
VAMP5	-0.03855955	0.07240875	-3.0880322	0.002018529	0.005046322	-2.45230656	ns
CAPS	-0.06282439	0.183919936	-3.08730017	0.002023401	0.005050051	-2.46727217	ns
FST	-0.03673739	0.010201476	-3.08729263	0.002023479	0.005050051	-2.46346838	ns
NFE2	-0.05098379	0.033972294	-3.08466233	0.00204152	0.005090724	-2.46237942	ns
UBXN1	-0.0467246	0.028770496	-3.08301897	0.002052804	0.005114494	-2.46825072	ns
MDK	-0.0366417	-0.00417166	-3.0822099	0.002058319	0.005123863	-2.48079054	ns
DECR1	-0.06905865	-0.04681159	-3.07727108	0.002092733	0.005205094	-2.49413276	ns
BCL2L15	-0.03822871	0.052175926	-3.07616393	0.002100591	0.005220192	-2.48684605	ns
ADGRV1	0.046183114	0.076882391	3.075390414	0.002106033	0.005225561	-2.49124206	ns
ASPSCR1	-0.04563854	0.002254882	-3.07532599	0.00210638	0.005225561	-2.50485776	ns
NDUFS6	-0.05675529	0.046530358	-3.07509604	0.00210812	0.005225561	-2.4911858	ns
RAB11FIP3	-0.07300816	-0.04093516	-3.07097823	0.002137284	0.005293357	-2.51727736	ns
AP2B1	-0.0328172	0.078026705	-3.06692404	0.002166559	0.005361316	-2.51661037	ns
CDC27	-0.04375143	0.075676815	-3.06478728	0.002182106	0.005395215	-2.52263964	ns
SHMT1	-0.06211137	-0.02802833	-3.06388302	0.002188622	0.005406749	-2.5350354	ns
LEG1	0.04518027	0.056077238	3.0589404	0.002225223	0.005492519	-2.52562538	ns
MPRIP	-0.02961458	-0.02048133	-3.05829028	0.002229853	0.005499299	-2.55439334	ns
GAL	0.046951546	0.068500376	3.054329448	0.002259491	0.005567692	-2.56516159	ns
TJAP1	-0.0598554	0.026097169	-3.0531453	0.002268416	0.005584971	-2.56941949	ns
SCT	0.030197255	0.04387826	3.05242166	0.002273971	0.005593931	-2.56071224	ns
SPINT2	-0.02874268	-0.02112183	-3.05126122	0.002282697	0.005610668	-2.57447235	ns
ENTPD6	-0.02002995	-0.04582874	-3.04733175	0.002312825	0.00567994	-2.5764007	ns
CCL15	-0.03020745	0.03510263	-3.0437504	0.002340401	0.005742833	-2.60036682	ns
GMFG	-0.04772738	-0.01471268	-3.04241165	0.002350836	0.005763595	-2.60407847	ns
SLC9A3R2	-0.03351376	0.062944497	-3.04139912	0.002358759	0.005778167	-2.6060887	ns
METAP1D	-0.05819456	-0.01158085	-3.03897595	0.002377833	0.005820011	-2.61054306	ns
CA2	-0.05107806	0.022368739	-3.03630932	0.002398967	0.00586682	-2.61691914	ns
MEPE	-0.01771951	-0.0170716	-3.03569019	0.002403886	0.005873931	-2.61826165	ns
ACYP1	-0.04428677	-0.00051881	-3.0322829	0.002431241	0.005933326	-2.62033798	ns
YES1	-0.08171654	-0.03419532	-3.03214914	0.002432257	0.005933326	-2.62871311	ns
PPP2R5A	-0.07812024	0.03624698	-3.03167284	0.002436052	0.005937623	-2.63544745	ns
DNAJB1	-0.06292003	-0.03943587	-3.03121772	0.002439769	0.005941722	-2.63151993	ns
ILKAP	-0.04104773	0.019983732	-3.02456205	0.002494135	0.006069063	-2.64452551	ns
SIL1	-0.02132811	0.036300461	-3.02385317	0.002499972	0.006074458	-2.64697343	ns
CMC1	-0.05960421	0.010765939	-3.02377427	0.002500513	0.006074458	-2.66027241	ns
GATD3	-0.05073186	0.025262772	-3.02284942	0.002508274	0.006088246	-2.64998966	ns
MARS1	-0.05652099	-0.01219094	-3.01858469	0.002543818	0.006169392	-2.66259922	ns
CCL4	-0.04690804	0.017137481	-3.0143484	0.002579524	0.006250797	-2.68362879	ns
WFDC1	-0.03643813	0.056233955	-3.01333247	0.00258823	0.006266052	-2.68184648	ns
EGFL7	-0.02824707	-0.00495129	-3.01311506	0.002590111	0.006266052	-2.67871109	ns
TCTN3	-0.01954462	-0.02225449	-3.01229305	0.002597007	0.006277535	-2.69471799	ns
ANXA10	-0.05441052	0.044104346	-3.00860822	0.002628809	0.006349149	-2.69309588	ns
KDR	-0.01803941	-0.01898273	-3.00792038	0.002634659	0.006358019	-2.7056595	ns
ADAMTS4	-0.02304723	-0.01515381	-3.00599597	0.002651494	0.006393363	-2.69892781	ns
NOTCH2	-0.0136626	0.0187563	-3.00427238	0.002666426	0.006424063	-2.71827364	ns
LAYN	-0.02191706	-0.00549234	-3.00336227	0.002674479	0.006438153	-2.71324043	ns
LTBP2	0.021335828	-0.01365268	3.001641613	0.002689554	0.006467744	-2.72689577	ns
SIGLEC5	-0.08327598	-0.34862665	-3.00145954	0.002691202	0.006467744	-2.72351503	ns
DAG1	-0.03401178	-0.01277484	-3.00030989	0.002701362	0.006486823	-2.72772173	ns
TNR	0.029891094	0.04344359	2.999843102	0.002705544	0.006491527	-2.72485908	ns
UFD1	-0.0917387	-0.07061145	-2.9976217	0.002725274	0.006527212	-2.73749078	ns
CTSF	0.028059647	-0.00066035	2.997422552	0.002727062	0.006527212	-2.7369938	ns
CCL17	-0.06352522	-0.08126845	-2.99742026	0.002727123	0.006527212	-2.73217564	ns
RRAS	-0.08528869	0.130325547	-2.99422486	0.002755884	0.006590647	-2.73558923	ns
NFU1	-0.06651833	-0.04513239	-2.99151522	0.002780344	0.006643702	-2.75566875	ns
IFT20	-0.02932392	0.054336889	-2.99080627	0.002786904	0.006653932	-2.7457573	ns
KLF4	-0.02698976	0.003301661	-2.98515538	0.002838891	0.006772517	-2.76301519	ns
GORASP2	-0.03091934	0.037415115	-2.98481526	0.002841934	0.006774242	-2.77557078	ns
GAS2	-0.03461043	0.061635028	-2.98385523	0.002850917	0.006790113	-2.77171537	ns
MPI	-0.06057609	0.02168952	-2.981117	0.00287651	0.006845483	-2.78122827	ns
NFKB1	-0.0486957	0.016001264	-2.97770235	0.00290881	0.006916714	-2.7837165	ns
TNFAIP8	-0.02469699	0.006146925	-2.97558178	0.002928962	0.006958967	-2.79162632	ns
WASF1	-0.06526588	0.084448294	-2.9680905	0.003001273	0.007124973	-2.81328687	ns
MAPRE3	-0.09610662	0.546414276	-2.96429863	0.003038499	0.007207488	-2.82161658	ns

LTO1	-0.02886527	0.019807427	-2.96121766	0.003069018	0.007273972	-2.83400655	ns
TINAGL1	-0.0151586	-0.01256524	-2.95960001	0.00308512	0.007306204	-2.84363474	ns
JUN	-0.03625729	0.052226357	-2.95818462	0.003099362	0.007333984	-2.84292913	ns
FCRL3	-0.03738488	-0.02485141	-2.95488408	0.003132692	0.007406851	-2.85262825	ns
GBA	-0.03503276	-0.02220117	-2.9543644	0.003137967	0.007413319	-2.85342683	ns
LAMTOR5	-0.02663807	0.028809154	-2.95317123	0.00315001	0.007435754	-2.86903845	ns
DPP4	0.020153424	-0.0035965	2.951644951	0.003165759	0.007466894	-2.85876289	ns
ITGB1BP1	-0.02804442	-0.01081812	-2.94726424	0.003210913	0.007567284	-2.8737665	ns
GGCT	-0.03134113	0.019026555	-2.94569384	0.003227115	0.007599336	-2.89115894	ns
TIMP3	-0.09032588	0.089397347	-2.94423182	0.003242406	0.007629189	-2.89341147	ns
TK1	-0.02901087	0.034499433	-2.93857009	0.003302235	0.007763708	-2.90501052	ns
AKR1C4	-0.03646951	0.040982254	-2.93789706	0.003309474	0.007774467	-2.90190794	ns
NUDC	-0.04408846	-0.0570737	-2.93760864	0.003312401	0.00777509	-2.91651719	ns
TNFSF11	-0.04267868	0.033923726	-2.936998	0.003318987	0.007778652	-2.91343642	ns
PTPN1	-0.05533393	0.050322175	-2.93697594	0.003319247	0.007778652	-2.91035698	ns
NOTCH1	-0.01055309	0.001643544	-2.93627024	0.003326726	0.007789929	-2.91983614	ns
COMT	-0.05778781	-0.0497991	-2.93591132	0.003330764	0.007793133	-2.904057	ns
ENPP5	0.03017972	-0.014431	2.935123421	0.00333907	0.007806313	-2.9200286	ns
DCTN1	-0.0453876	0.000973225	-2.93426478	0.003348284	0.00782159	-2.92694513	ns
SSB	-0.03881718	0.078825803	-2.93222107	0.003370533	0.007867272	-2.91862206	ns
KAZN	-0.06722632	-0.05088238	-2.9301846	0.00339256	0.007907417	-2.93618622	ns
KIFBP	-0.05698283	-0.1074722	-2.93013555	0.003393148	0.007907417	-2.9310101	ns
HARS1	-0.05321997	-0.01981114	-2.92366495	0.003464416	0.008067062	-2.9526166	ns
GFRA3	0.019006687	0.018740388	2.922289082	0.003479799	0.008096424	-2.95145867	ns
AIDA	-0.05601275	0.179887789	-2.92130558	0.003490834	0.008115633	-2.94996042	ns
CLSTN3	-0.01931339	0.002806415	-2.92051212	0.003499602	0.008129545	-2.96532443	ns
SCG2	-0.02089547	0.003569741	-2.92018327	0.003503312	0.008131694	-2.96579289	ns
ATXN2L	-0.03038077	0.064236122	-2.91923025	0.003514011	0.008150049	-2.96995924	ns
PPP1R2	-0.05449174	-0.00658199	-2.91834347	0.003524086	0.00816693	-2.965584	ns
DTD1	-0.05541046	0.014357312	-2.91775654	0.003530777	0.008175947	-2.96109429	ns
CST5	0.037601274	0.032074523	2.914790241	0.003564511	0.008246835	-2.96924245	ns
REEP4	-0.04054177	0.104006957	-2.91455584	0.003567038	0.008246835	-2.98351396	ns
CD207	-0.0257024	-0.00969881	-2.91324517	0.003582204	0.008275345	-2.97241376	ns
NRTN	-0.03371937	-0.00696535	-2.91286675	0.003586519	0.008278764	-2.97515703	ns
C1QTNF9	0.031109121	-0.03522758	2.912569279	0.003589928	0.008280087	-2.97539583	ns
HSD17B14	-0.03892455	-0.01463328	-2.9114105	0.003603136	0.008301052	-2.99170834	ns
GMPR2	-0.04266238	0.02200311	-2.911285	0.003604703	0.008301052	-2.97984194	ns
SH3BP1	-0.03151713	0.081250058	-2.90957522	0.00362436	0.008339741	-2.99600838	ns
NDUFB7	-0.05618569	0.022389467	-2.90729773	0.003650841	0.008394059	-3.0025933	ns
SERPINB6	-0.04095567	0.008138884	-2.90578216	0.003668566	0.008428177	-3.00725467	ns
OGFR	-0.02262803	0.002892987	-2.9052338	0.003675046	0.008436426	-3.00323251	ns
CRTAC1	0.020269602	-0.03935703	2.902949042	0.003701876	0.008491342	-3.01654923	ns
ICAM4	0.03655063	-0.0441496	2.902455612	0.003707766	0.008498177	-3.01369254	ns
SLC51B	0.048212198	0.179493295	2.901569919	0.003718368	0.008515791	-3.00616056	ns
DNPH1	-0.03566897	0.001263057	-2.89793666	0.003761564	0.008607968	-3.02723465	ns
CEP43	-0.04608311	0.019300825	-2.89647789	0.003779236	0.008641636	-3.01939336	ns
TNFSF8	-0.02222601	-0.00572543	-2.89522339	0.003794184	0.008669028	-3.03804325	ns
IL20RA	-0.03077594	0.052029597	-2.89464503	0.00380131	0.008678518	-3.02774938	ns
PTGES2	-0.03410763	0.00635611	-2.89364399	0.003813517	0.008699586	-3.02479671	ns
ECI2	-0.03888561	0.081604194	-2.88932266	0.003866245	0.008812985	-3.04140735	ns
PADI2	-0.03919686	0.060466111	-2.8880489	0.0038819	0.008841769	-3.04670614	ns
NMT1	-0.04144769	0.101346928	-2.88632839	0.003903026	0.008882959	-3.06490596	ns
CIAPIN1	-0.04278377	-0.0048109	-2.88510272	0.003918277	0.008910723	-3.06540135	ns
IL17C	-0.04641298	0.029765727	-2.88341872	0.003939395	0.008951776	-3.05998714	ns
EIF4G3	-0.04214719	0.084483404	-2.88040333	0.003977103	0.009030435	-3.08189	ns
SELL	-0.01428107	0.010235057	-2.87969854	0.003986009	0.009043625	-3.08307401	ns
GGACT	-0.04459376	0.024296281	-2.87839136	0.004002558	0.009074122	-3.08678055	ns
GRP	-0.03298979	-0.0078918	-2.87750723	0.004013934	0.009089022	-3.07617734	ns
TRDMT1	-0.05341986	0.072068488	-2.87738995	0.004015356	0.009089022	-3.0818584	ns
PRKG1	-0.08004057	-0.03720489	-2.87548818	0.004039656	0.009136945	-3.08279205	ns
NAGA	-0.03732985	0.083597461	-2.87444788	0.004053033	0.009160105	-3.0824015	ns
GNLY	-0.0348641	-0.04255065	-2.86826335	0.004132936	0.009333468	-3.11210817	ns
CES3	-0.04861167	0.105642	-2.86790869	0.004137596	0.009336769	-3.11033323	ns
F13B	-0.01592929	-0.01699364	-2.86646534	0.004156466	0.009372109	-3.1173563	ns
CELA2A	0.036537527	0.010258095	2.866037019	0.004162057	0.009377474	-3.12162468	ns
AK1	-0.04382594	0.007444219	-2.86511145	0.004174299	0.009397806	-3.11860111	ns

GLRX5	-0.0545909	0.00724146	-2.86221577	0.004212671	0.009476887	-3.12063963	ns
DBNL	-0.06264112	-0.04412986	-2.85907933	0.004254425	0.009563449	-3.13903447	ns
PPP1R14A	-0.04909884	-0.00945929	-2.85681062	0.004284895	0.009624533	-3.14917215	ns
OPLAH	-0.04304718	0.026716267	-2.85595663	0.004296599	0.009643404	-3.13758488	ns
TBCA	-0.05347414	0.01215821	-2.85551384	0.004302565	0.00964506	-3.13968437	ns
GLRX	-0.03880448	0.00327093	-2.85540971	0.004303943	0.00964506	-3.14399513	ns
TF	-0.01352322	-0.00701648	-2.85215537	0.004348132	0.009736614	-3.16154805	ns
DBN1	-0.03277463	0.046510328	-2.85118709	0.004361564	0.009759209	-3.14954799	ns
ATP6V1G1	-0.04544489	0.034673694	-2.84946133	0.004385266	0.009801683	-3.15600252	ns
CDH23	0.01637873	0.015483431	2.84930492	0.00438726	0.009801683	-3.16954499	ns
PMM2	-0.03716545	0.041929373	-2.84760465	0.004410933	0.009847037	-3.16035133	ns
PSME1	-0.02531943	-0.00260616	-2.8456575	0.004437884	0.009899634	-3.17281407	ns
DNPEP	-0.03484485	0.009947578	-2.84370509	0.004465064	0.009952662	-3.18539161	ns
CDH17	0.045124791	0.068689641	2.842060814	0.004488219	0.009996644	-3.18398788	ns
FAM13A	-0.04226295	0.053318985	-2.83750216	0.00455272	0.010132578	-3.20189583	ns
DKK3	0.019437981	0.007080971	2.834018643	0.004602601	0.010235792	-3.2131166	ns
MRPL58	-0.0451709	0.125381575	-2.83240211	0.00462592	0.010279822	-3.21819904	ns
NCLN	-0.03595609	0.102801357	-2.82487952	0.004736039	0.010516527	-3.22606231	ns
DKK1	-0.03930539	-0.04478916	-2.82414796	0.004746696	0.010532183	-3.24143736	ns
PFKFB2	-0.05120531	0.001772355	-2.82147319	0.004786533	0.010612511	-3.24105309	ns
DMP1	0.03693627	0.010928345	2.820325253	0.004803762	0.010642629	-3.23810346	ns
ARHGAP45	-0.05074094	-0.00201376	-2.81925811	0.004819727	0.010669903	-3.24193835	ns
PGLYRP2	-0.01867069	-0.03856765	-2.81714212	0.004851527	0.010730306	-3.25272288	ns
IKBKG	-0.06055989	-0.00835634	-2.81695253	0.004854361	0.010730306	-3.25676122	ns
PCBP2	-0.04418278	-0.00017281	-2.81562722	0.004874347	0.010766335	-3.26529511	ns
NME3	-0.01518251	0.003821683	-2.81454837	0.004890797	0.010782659	-3.26257776	ns
TOR1AIP1	-0.03180238	0.021777381	-2.8145394	0.004891048	0.010782659	-3.25430678	ns
SAA4	-0.01998484	0.03625041	-2.81421312	0.004895843	0.010782659	-3.26841664	ns
MPIG6B	-0.05060813	-0.16822671	-2.81417233	0.004896509	0.010782659	-3.26458401	ns
CD28	-0.02340843	0.002871527	-2.81374802	0.004903113	0.010789066	-3.25603858	ns
LILRA6	-0.04490543	-0.08383911	-2.81322131	0.004910965	0.010798205	-3.27115607	ns
PPIB	-0.06275919	-0.09391115	-2.81274712	0.004918283	0.010806159	-3.26667398	ns
DGKA	-0.03320536	0.034660368	-2.81100238	0.004945131	0.010856978	-3.2626285	ns
SUGP1	-0.02514239	0.04969054	-2.80894715	0.004976616	0.010917894	-3.28204071	ns
HDAC9	-0.0404306	0.087882006	-2.80785535	0.004993669	0.010947083	-3.27346794	ns
TMPRSS5	0.025763932	-0.00588975	2.807032337	0.005006393	0.010966743	-3.27832048	ns
KLK1	0.083427268	0.43172829	2.804723085	0.005042355	0.011037238	-3.28768989	ns
MAN2B2	-0.03626642	-0.06338169	-2.80412947	0.005051544	0.01104907	-3.29654295	ns
ARHGEF12	-0.06840696	-0.03255201	-2.80221492	0.005081666	0.011106634	-3.29792387	ns
BANK1	-0.07011989	-0.09860749	-2.80106093	0.005099864	0.011138072	-3.30113789	ns
SH2B3	-0.06424803	-0.04187252	-2.80072128	0.00510536	0.011141741	-3.29238792	ns
GDF2	0.023586085	0.019448341	2.800212978	0.005113289	0.011150712	-3.30134742	ns
PTRHD1	-0.03690112	-0.00351064	-2.7992125	0.005129221	0.011177107	-3.29791374	ns
PTGR1	0.049815708	0.000851081	2.797155564	0.00516183	0.011239778	-3.31596033	ns
HTRA2	-0.04023527	0.025624336	-2.7956481	0.005185975	0.011283939	-3.31799237	ns
LEO1	-0.01792081	0.00957895	-2.78619058	0.005339622	0.011609602	-3.34726398	ns
GNE	-0.04271063	0.031629852	-2.78488367	0.005361387	0.011648251	-3.33635363	ns
SART1	-0.02299437	0.02881335	-2.78360321	0.005382589	0.011685621	-3.33946947	ns
FCGR3B	-0.03243205	-0.04711524	-2.78256451	0.005399727	0.011714117	-3.34957287	ns
SLC16A1	-0.03766759	0.072578494	-2.77978796	0.005446184	0.01180613	-3.351352	ns
PLA2G7	-0.0202263	-0.01671476	-2.77894129	0.005460259	0.011827862	-3.36283238	ns
MASP1	-0.01510358	-0.01695541	-2.77501393	0.005526661	0.011962825	-3.36904094	ns
PEPD	0.015653178	-0.00093538	2.773724994	0.005548486	0.01200117	-3.37971886	ns
TDO2	-0.02309259	0.031305279	-2.77325925	0.00555659	0.012009802	-3.36895358	ns
LACTB2	-0.04259706	0.015450258	-2.77243762	0.005570543	0.012031055	-3.37828142	ns
PIBF1	-0.04697338	0.025787118	-2.77155404	0.005585583	0.012054622	-3.38770036	ns
MESD	-0.07916745	-0.03171814	-2.77014122	0.005609925	0.012098213	-3.38710515	ns
PPP1CC	-0.05033715	0.042253375	-2.76833194	0.005641115	0.0121565	-3.39471224	ns
TXNDC9	-0.03176399	0.074873574	-2.75833344	0.005816499	0.012525205	-3.42312307	ns
ANXA5	-0.01871437	-0.01588458	-2.75626657	0.005853574	0.012595752	-3.41540398	ns
SCGB3A2	0.0537464	0.052983151	2.755260512	0.00587144	0.012624894	-3.42767211	ns
DDHD2	-0.04026771	0.006013636	-2.75205364	0.005929156	0.012739614	-3.44124357	ns
GRSF1	-0.04871748	0.073775296	-2.75155473	0.005938462	0.012750227	-3.42304408	ns
VWA1	-0.02744498	-0.01039964	-2.75041755	0.005958841	0.012784582	-3.44564937	ns
ITGAL	-0.02653386	-0.03994738	-2.74770236	0.006008598	0.012881868	-3.43750373	ns
STX16	-0.03793543	0.08181261	-2.74659921	0.006028829	0.012915759	-3.4416038	ns

YWHAQ	-0.04413864	0.026547805	-2.74549252	0.006049141	0.012949774	-3.44595404	ns
FOXO1	-0.05484705	-0.01779692	-2.74439568	0.006069289	0.012983388	-3.45733185	ns
HSD17B3	-0.02876357	0.038744087	-2.74355551	0.006084982	0.01299861	-3.44948051	ns
SERPING1	-0.01123874	-0.01211803	-2.74352716	0.006085308	0.01299861	-3.46205798	ns
HPCAL1	-0.04754793	-0.10820676	-2.7409265	0.006133625	0.013092241	-3.47168861	ns
CACNB1	-0.03509517	0.082709257	-2.73895777	0.006170669	0.01316169	-3.46340525	ns
DNAJB6	-0.05080129	-0.00258238	-2.73836332	0.006181872	0.013175962	-3.46046938	ns
MMP3	-0.03009833	0.007562566	-2.73647712	0.006217225	0.013241645	-3.47924917	ns
PLA2G1B	0.027012197	0.024443962	2.734839636	0.006248171	0.013297857	-3.48608369	ns
ITPR1	-0.04180001	0.061217129	-2.73416091	0.006261247	0.013315981	-3.4759688	ns
GC	-0.01525958	0.026826384	-2.73340521	0.00627543	0.01333643	-3.49125601	ns
PDAP1	-0.05820502	-0.03326092	-2.7329839	0.006283461	0.013343787	-3.4923658	ns
MINK1	-0.05558478	-0.04931503	-2.73254984	0.006291737	0.013351652	-3.49349856	ns
FDX2	-0.03434166	0.026710124	-2.73143727	0.006313231	0.013387535	-3.48245545	ns
PIKFYVE	-0.03317902	0.065213124	-2.72999004	0.006340998	0.013436657	-3.48572408	ns
PTEN	-0.01877194	-0.00554402	-2.72851813	0.006369335	0.013486917	-3.49171264	ns
SLURP1	-0.0219793	0.000671023	-2.72363326	0.006464106	0.013677674	-3.51415048	ns
DKK4	-0.02883337	0.009476416	-2.72227992	0.006490695	0.013715617	-3.5127444	ns
CD101	-0.02555703	-0.06271614	-2.72223674	0.006491433	0.013715617	-3.52044623	ns
STAT2	-0.04614663	0.025058675	-2.72131844	0.006509693	0.0137267	-3.50921824	ns
IMPACT	-0.03387138	-0.00900124	-2.72128952	0.006510038	0.0137267	-3.52402609	ns
DDX58	-0.03701651	0.063343732	-2.72126302	0.006510781	0.0137267	-3.51045677	ns
MYBPC2	-0.0487098	0.18236127	-2.71912984	0.006552881	0.013805493	-3.51670836	ns
DOK2	-0.07813123	-0.04117119	-2.717032	0.006594428	0.013883007	-3.52945905	ns
CDC26	-0.0419855	0.044959505	-2.71610521	0.006612851	0.013911762	-3.53576508	ns
PSMD9	-0.03656224	0.050005513	-2.71174265	0.006700529	0.014086066	-3.54343317	ns
MED18	-0.04870428	-0.01869304	-2.70834513	0.006769613	0.014221058	-3.54531672	ns
C2orf69	-0.03377553	0.071056661	-2.70520789	0.006833816	0.014345609	-3.55424793	ns
SFTPA1	-0.03565407	0.089518938	-2.70283038	0.0068829	0.014438268	-3.56014824	ns
SUSD5	-0.01989229	-0.0123051	-2.70177171	0.006904843	0.014473899	-3.56133225	ns
GOPC	-0.06433811	-0.06497915	-2.70029798	0.006935338	0.014527395	-3.57667773	ns
PNLIP	0.035317857	0.042523442	2.697944632	0.006984467	0.014619815	-3.58583313	ns
ADAMTS16	-0.01987687	0.003784119	-2.69611957	0.007023041	0.014690029	-3.578907	ns
PLCB2	-0.05728055	-0.04359001	-2.69324512	0.007083634	0.014806164	-3.59950904	ns
RCOR1	-0.02809543	0.070401337	-2.69219419	0.007106205	0.014842717	-3.58941957	ns
SORT1	-0.02328806	-0.00768881	-2.69074372	0.007137045	0.014891698	-3.59961419	ns
MICALL2	-0.04215297	0.130576645	-2.69061638	0.007139855	0.014891698	-3.59337982	ns
RAPGEF2	-0.0311609	0.06697825	-2.68949092	0.007163885	0.014931153	-3.60176393	ns
ATXN3	-0.0372506	0.011150893	-2.68614987	0.007235768	0.015070215	-3.61848243	ns
EPO	-0.04747706	0.072446807	-2.68577199	0.00724403	0.01507667	-3.61556793	ns
AP3S2	-0.02793665	0.074404045	-2.68531179	0.00725393	0.015086521	-3.62072023	ns
VCAM1	-0.01654446	0.000291885	-2.68469137	0.007267488	0.015103961	-3.61704962	ns
DNAJC6	-0.04945618	0.033372294	-2.68414479	0.007279505	0.015110464	-3.6106615	ns
DSG3	-0.02094518	-0.00361557	-2.68406879	0.007280967	0.015110464	-3.62347246	ns
EVI5	-0.05287477	-0.00236064	-2.6833871	0.007296034	0.015130981	-3.61110866	ns
RILP	-0.03838656	-0.00541491	-2.68049968	0.007359018	0.015250769	-3.6338922	ns
DNAJC9	-0.04540181	-0.0158368	-2.67492768	0.007482402	0.01549372	-3.64737129	ns
FOLH1	-0.06417263	0.318674446	-2.67473781	0.007486863	0.01549372	-3.63414217	ns
CA3	-0.03857155	0.016364658	-2.6689208	0.007617471	0.015752844	-3.66381449	ns
FCRLB	-0.03688673	0.046436327	-2.66768578	0.007645747	0.015800128	-3.65395758	ns
BOLA1	-0.03167913	0.01293374	-2.66126619	0.007792987	0.016082899	-3.67193121	ns
AGR3	-0.0373084	0.008485074	-2.6612414	0.007793596	0.016082899	-3.67026174	ns
HLA-A	-0.01887302	-0.03275361	-2.65977787	0.007827251	0.01613899	-3.68948734	ns
SNAPIN	-0.03368517	0.020923945	-2.65958071	0.007831832	0.01613899	-3.69000871	ns
DCTN2	-0.05664067	0.080828497	-2.65850739	0.007856929	0.016179289	-3.68553671	ns
TTR	0.014062232	-0.00211707	2.657336236	0.007884258	0.016224126	-3.68907164	ns
NPTXR	0.021333368	0.046595402	2.656684469	0.007899511	0.016244065	-3.69065219	ns
NFX1	-0.03980149	0.063418784	-2.65531773	0.007931727	0.016298834	-3.68624935	ns
FGF9	0.023154958	-0.02044946	2.653990067	0.007963014	0.016351618	-3.68668795	ns
YOD1	-0.03729518	0.005495756	-2.6535053	0.00797443	0.016363552	-3.69103161	ns
C1QTNF1	-0.02620222	-0.00940625	-2.65325839	0.007980089	0.016363665	-3.7009578	ns
ARL13B	-0.04098572	0.079931612	-2.65257179	0.007996504	0.016385819	-3.69349348	ns
ICA1	-0.0494176	0.050665138	-2.64884674	0.008085095	0.016555734	-3.70496437	ns
BIRC2	-0.03996741	0.039239979	-2.64577131	0.008158967	0.016695293	-3.71183163	ns
CABP2	-0.03855623	0.107420937	-2.64546687	0.008166268	0.016698531	-3.71312363	ns
AKT1S1	-0.04122965	-0.02756892	-2.64515157	0.008173904	0.01670245	-3.71437094	ns

TRIM25	-0.06322365	-0.00986909	-2.64411067	0.008198856	0.016741719	-3.72885427	ns
STK4	-0.05394471	-0.01258294	-2.63771719	0.008355054	0.017048747	-3.74056825	ns
KLK11	-0.01798081	-0.0091138	-2.63639104	0.00838776	0.017103534	-3.7440226	ns
SEPTIN9	-0.01773497	0.026481088	-2.63456566	0.008432987	0.017183755	-3.74852795	ns
TGFBI	-0.01648829	-0.01174741	-2.63310592	0.008469374	0.017245866	-3.74845163	ns
GTPBP2	-0.04652986	0.035633194	-2.63193255	0.008498697	0.017293515	-3.74862445	ns
KLK10	-0.02402453	-0.01359044	-2.62412794	0.008695686	0.017682035	-3.77901347	ns
TSC1	-0.04370965	0.165846253	-2.61979885	0.008807003	0.017895929	-3.7787128	ns
NUB1	-0.05128989	0.044894868	-2.6167045	0.008887019	0.018045964	-3.79761398	ns
IRAK4	-0.06426503	-0.12338317	-2.61495821	0.008932564	0.018125842	-3.8021554	ns
IGDCC4	0.014701857	0.023081224	2.608559658	0.009101151	0.018455112	-3.82231401	ns
BOLA2_BOLA2	-0.03194818	-0.02938422	-2.60795426	0.00911749	0.018475414	-3.81106683	ns
HSPB1	-0.04241837	-0.14338851	-2.60709591	0.009140381	0.018508954	-3.81278934	ns
GRAP2	-0.07851438	-0.10292451	-2.60245367	0.009264851	0.018748	-3.83242128	ns
CD69	-0.0534436	-0.07174419	-2.60192356	0.009279232	0.018764098	-3.83053357	ns
CNTN2	0.027342434	0.041309625	2.601357405	0.009294378	0.018781719	-3.84301714	ns
HMCN2	-0.03209004	-0.04793224	-2.60012954	0.009327925	0.018836473	-3.83123012	ns
GABARAPL1	-0.03888991	0.151129101	-2.59892082	0.009360726	0.018889647	-3.83984227	ns
ELAC1	-0.04844376	-0.03533095	-2.59644418	0.009428298	0.019012867	-3.85497539	ns
CTRC	0.037606757	0.04297903	2.594561342	0.009480189	0.019104315	-3.85298869	ns
HPGDS	0.022294097	0.012222108	2.593479962	0.009510211	0.019151597	-3.84800136	ns
DIABLO	-0.06666729	-0.02743327	-2.58948851	0.009621	0.019361351	-3.86371015	ns
GOT1	-0.02270915	0.016197126	-2.589203	0.009628836	0.019363776	-3.87232927	ns
OTUD6B	-0.03321264	0.022561018	-2.58839255	0.009651525	0.019396045	-3.8737834	ns
SH2D1A	-0.03371824	0.05250215	-2.58451735	0.009760686	0.019601929	-3.88083334	ns
NAMPT	-0.03232523	0.040595742	-2.58411473	0.009772267	0.019611698	-3.87210224	ns
SPRING1	-0.03521366	0.102760336	-2.58089029	0.009863983	0.019782164	-3.87824771	ns
PMVK	-0.06675254	-0.30859416	-2.57626528	0.009996852	0.020034871	-3.89173651	ns
TRIM5	-0.05196631	-0.00204847	-2.57483393	0.010038105	0.020103748	-3.90566828	ns
PLXNB2	-0.01383449	-0.01441118	-2.57457493	0.010045575	0.02010492	-3.9077618	ns
TP53	-0.02591365	-0.00850661	-2.57431422	0.010053377	0.020106754	-3.89723	ns
CD163	-0.02629097	-0.02174901	-2.57390122	0.010065188	0.020116598	-3.90715465	ns
ADAM23	-0.02087321	-0.03862747	-2.57055898	0.010162683	0.02029756	-3.92152759	ns
RBP1	-0.03976598	0.140594665	-2.56880852	0.010214391	0.020386892	-3.9117972	ns
SNX2	-0.04624227	0.062905844	-2.56651471	0.010281961	0.020507737	-3.92991979	ns
NUMB	-0.04731915	-0.0424034	-2.56353715	0.010370796	0.020670802	-3.92365748	ns
CELA3A	0.033522862	0.035984228	2.562644146	0.010397216	0.020709324	-3.94019917	ns
FOXO3	-0.05808133	0.111166139	-2.5619375	0.010418658	0.020737889	-3.92882665	ns
SNAP25	-0.02556252	0.03154438	-2.55758091	0.010549997	0.020985007	-3.94088642	ns
ZBTB16	-0.04299515	0.114089428	-2.55356855	0.010672303	0.021203037	-3.95010561	ns
GSR	-0.01243748	-0.00752614	-2.553502	0.010674132	0.021203037	-3.95957471	ns
MITD1	-0.05894267	-0.07896957	-2.54989684	0.010785161	0.021409021	-3.96601101	ns
LRP2BP	-0.04643005	0.218250407	-2.54455482	0.010951571	0.021724584	-3.9734395	ns
MERTK	-0.01488615	-0.01656015	-2.54181782	0.011037395	0.021879969	-3.99457354	ns
CLTA	-0.02710444	0.04076626	-2.5390782	0.011124467	0.022037615	-3.98753948	ns
FADD	-0.04510799	-0.00390822	-2.53683458	0.011195824	0.022163936	-4.00005565	ns
TCN2	-0.01760092	-0.01894231	-2.53079089	0.011390386	0.022533827	-4.02072304	ns
PLA2G10	0.035743626	0.023106515	2.527993323	0.01148165	0.022698996	-4.02129839	ns
CD86	-0.01564367	-0.00165069	-2.52670437	0.011523727	0.022766769	-4.03064409	ns
ARF6	-0.05882387	-0.07728748	-2.51783354	0.011817928	0.023332218	-4.05288996	ns
PVR	-0.02089307	-0.01870719	-2.51526981	0.011904157	0.023486581	-4.06128348	ns
BLVRB	-0.04010432	0.066930919	-2.51323026	0.011973267	0.023600564	-4.06182829	ns
KRT14	-0.01641898	0.012872668	-2.51310423	0.011978095	0.023600564	-4.03766196	ns
DYNLT3	-0.02393362	0.022529993	-2.51139926	0.012035478	0.023697638	-4.0690495	ns
NEXN	-0.03819434	0.069892856	-2.51049014	0.012066481	0.023742672	-4.07118221	ns
MYCBP2	-0.03714351	0.084376171	-2.50969126	0.012093771	0.023780344	-4.07518966	ns
CNTNAP4	-0.0580016	0.363790031	-2.50360918	0.012303832	0.024177113	-4.07617966	ns
KLK14	-0.02693254	0.00705239	-2.50328298	0.012314901	0.02418259	-4.09189923	ns
MRPL46	-0.01551873	0.007839907	-2.50193699	0.012362105	0.024251391	-4.08075963	ns
RWDD1	-0.03765151	-0.00280278	-2.50180118	0.012366549	0.024251391	-4.09487651	ns
NGRN	-0.01210672	0.010941475	-2.49589638	0.012574239	0.024642133	-4.10859125	ns
NAGK	-0.03107768	0.008383735	-2.49534661	0.012594041	0.024664386	-4.09563634	ns
DCTD	-0.05159717	-0.05431009	-2.49309692	0.01267402	0.024804382	-4.10313528	ns
CD93	-0.01738123	-0.00281538	-2.48650002	0.012911659	0.025247339	-4.10653876	ns
DPEP1	0.028770517	-0.05640025	2.486324688	0.012917645	0.025247339	-4.12402119	ns
IGFBP3	0.015862221	-0.00385257	2.484555071	0.012981919	0.025355989	-4.13182009	ns

TWF2	-0.0457368	0.016038867	-2.48317901	0.013032068	0.025436925	-4.13967957	ns
USO1	-0.05283086	-0.04736892	-2.48241893	0.013060019	0.025474453	-4.13552398	ns
NFAT5	-0.04746163	0.141869548	-2.47181024	0.013454075	0.026225566	-4.15322863	ns
ITGB1BP2	-0.06431451	-0.09242788	-2.46998232	0.013522967	0.026342271	-4.1595722	ns
NCK2	-0.06121248	-0.03250535	-2.46572146	0.01368457	0.026639296	-4.18020941	ns
PDE5A	-0.0562905	-0.10372039	-2.46542017	0.013696272	0.026644314	-4.17134984	ns
SARG	-0.05945507	-0.06807593	-2.46347569	0.013770565	0.026760008	-4.18637341	ns
IGDCC3	-0.02574476	0.07706132	-2.46339064	0.013774072	0.026760008	-4.17595621	ns
FASLG	0.021510782	0.042671887	2.461230289	0.013856938	0.026903098	-4.19501118	ns
CFHR4	-0.0388659	-0.10948649	-2.45945187	0.013925802	0.027018832	-4.19757011	ns
IL17RB	0.031167705	0.021288541	2.453516823	0.014157855	0.027450821	-4.20344269	ns
M6PR	-0.02135104	0.065963534	-2.45082251	0.014264296	0.027638849	-4.20616693	ns
CD5L	-0.02460669	0.013826106	-2.44762373	0.014391268	0.027866382	-4.22374629	ns
PBK	0.016289112	-0.00099361	2.444327163	0.014523674	0.028104128	-4.21934218	ns
ATXN10	-0.04097937	-0.02471162	-2.44327802	0.014565917	0.028167204	-4.22494717	ns
PDGFB	-0.04919599	-0.07750377	-2.44210727	0.014612965	0.028239482	-4.23782318	ns
TBC1D23	-0.0523934	0.017106392	-2.4406993	0.014670119	0.028331182	-4.23762369	ns
MANF	-0.06909349	-0.25297915	-2.44023213	0.014689012	0.028347485	-4.24237439	ns
BDNF	-0.04383169	-0.09279052	-2.44001045	0.014697977	0.028347485	-4.24533441	ns
CSPG4	0.017404839	0.004740762	2.434308901	0.01493148	0.028749867	-4.2591505	ns
KLB	0.042390438	0.004789808	2.434307701	0.014931839	0.028749867	-4.24669548	ns
MAP2K6	-0.05776937	-0.04439432	-2.43419822	0.014936147	0.028749867	-4.25585503	ns
EREG	-0.04575	0.055358132	-2.4332493	0.014975417	0.028806468	-4.25381527	ns
NRGN	-0.04795233	-0.02731205	-2.4322633	0.015016052	0.028865617	-4.26340067	ns
UHRF2	0.01854766	0.026746076	2.429707715	0.015122634	0.029051376	-4.25646868	ns
DAPP1	-0.06931286	-0.27996901	-2.42867943	0.015165334	0.029114252	-4.26919631	ns
PF4	-0.0510494	-0.05642136	-2.42550363	0.015298602	0.029342259	-4.27696145	ns
FLT1	-0.01206034	-0.00074714	-2.42537267	0.015304199	0.029342259	-4.27372082	ns
VCAN	0.016880282	0.00580492	2.42371202	0.015374146	0.029457025	-4.28609242	ns
AAMDC	-0.02810771	0.012003783	-2.42094483	0.015491956	0.029663285	-4.27851795	ns
CXCL5	-0.0652918	-0.08330736	-2.41871089	0.015587246	0.029826185	-4.28913854	ns
ST13	-0.03003378	-0.0014959	-2.4181961	0.015609388	0.029848993	-4.28598565	ns
SLC27A4	-0.03810354	0.076762618	-2.41486598	0.01575283	0.030103576	-4.29355711	ns
GP6	-0.05013657	-0.0482244	-2.4074102	0.016077851	0.030704595	-4.31974149	ns
CASP7	-0.04395167	-0.01620743	-2.40712792	0.016090152	0.030708003	-4.3249634	ns
IST1	-0.05198404	-0.02995086	-2.4049602	0.016185989	0.030852334	-4.32523833	ns
CTSV	0.022835498	0.03414461	2.404937477	0.016186909	0.030852334	-4.32989392	ns
OXCT1	-0.03593393	0.010908425	-2.40275013	0.016284309	0.031017732	-4.32214334	ns
DFFA	-0.03794665	0.024625016	-2.40164306	0.016333299	0.031090764	-4.33906762	ns
LZTFL1	-0.03727396	0.015091715	-2.39865506	0.016467425	0.031325655	-4.33277325	ns
NINJ1	-0.03205797	0.09235126	-2.39340665	0.016704909	0.031756727	-4.34392935	ns
NUBP1	-0.05248085	0.132931119	-2.38668198	0.01701315	0.032321664	-4.37273664	ns
PTPRH	-0.02486804	0.034601966	-2.38621727	0.017034647	0.032341462	-4.37376626	ns
SERPINF2	-0.00710819	0.00282687	-2.38292419	0.017187959	0.032611332	-4.3727757	ns
PADI4	-0.04118955	0.078538967	-2.37752451	0.017441708	0.03307129	-4.38183617	ns
FHIT	-0.04118186	-0.02496578	-2.37489694	0.017566294	0.033285905	-4.38871762	ns
PODXL2	-0.01491543	0.013059613	-2.37103702	0.017750459	0.033610466	-4.40855576	ns
BRAP	-0.05671827	-0.07880488	-2.37082777	0.017760599	0.033610466	-4.40558255	ns
CPVL	-0.02277218	-0.04548448	-2.37048292	0.017777039	0.033619788	-4.41246541	ns
BLOC1S3	-0.02121751	0.066570655	-2.36638118	0.017975164	0.033972477	-4.42166151	ns
TRAF3	-0.01935923	0.046941262	-2.36547187	0.018019728	0.034034673	-4.40890855	ns
PAGR1	-0.02201637	0.030777056	-2.36472407	0.018056101	0.034081328	-4.41225649	ns
CCL8	-0.03391489	-0.08009675	-2.36331958	0.018124314	0.034187982	-4.43001288	ns
MAX	-0.05813129	-0.00364015	-2.3537253	0.018599111	0.035060945	-4.4384813	ns
ARHGAP25	-0.03291428	0.061211885	-2.34912747	0.018830327	0.035473906	-4.4483118	ns
CD84	-0.01718139	-0.00421392	-2.34765984	0.018904398	0.035590485	-4.46157268	ns
SNX9	-0.03760511	0.030647864	-2.34132617	0.01922813	0.036176636	-4.4741555	ns
FKBP14	-0.05084468	-0.03884897	-2.33960633	0.019316683	0.036319842	-4.48523469	ns
SLA2	-0.05318465	-0.03263214	-2.33865891	0.019365715	0.036388602	-4.48743826	ns
CCL13	-0.03770735	-0.01076245	-2.33689648	0.019457233	0.036537054	-4.49083866	ns
NMI	-0.03660528	0.040922428	-2.33580661	0.019514101	0.036620293	-4.48985157	ns
TYMP	-0.03629981	-0.02481184	-2.33245839	0.019689573	0.036925853	-4.49216731	ns
IFIT1	-0.02661498	0.039074776	-2.32900383	0.019871844	0.037236119	-4.50103783	ns
COL24A1	-0.02775083	-0.05964221	-2.32883708	0.019880517	0.037236119	-4.50827012	ns
ADAMTS13	0.012242931	0.008123303	2.325686991	0.020048081	0.037525895	-4.51698201	ns
MYOM1	-0.0235227	0.053938588	-2.31959409	0.020376135	0.038115511	-4.51783112	ns

LCN15	-0.03033402	0.050378763	-2.31820493	0.020451115	0.038231277	-4.53479385	ns
ELAVL4	-0.04274085	0.071513876	-2.31567451	0.020589396	0.038465155	-4.5268623	ns
ALDH5A1	-0.02342845	0.044616667	-2.31478106	0.020637897	0.038531112	-4.54268028	ns
F2	-0.00751319	-0.00502788	-2.31315313	0.02072725	0.038673207	-4.54557706	ns
FKBP5	-0.05176491	-0.06021899	-2.31256285	0.020759915	0.038709419	-4.5396779	ns
IFNAR1	-0.0129086	0.02396281	-2.3103262	0.020883198	0.038914447	-4.55096323	ns
CSF2RA	-0.03344185	-0.05182923	-2.30419065	0.0212253	0.039526707	-4.55889277	ns
EIF5A	-0.01624918	0.008168778	-2.30371794	0.021251991	0.039538783	-4.55457521	ns
GCLM	-0.02301701	0.058291975	-2.30359676	0.021258866	0.039538783	-4.55317923	ns
JPT2	-0.04394205	-0.03595741	-2.30057155	0.021429104	0.039830035	-4.57332418	ns
PDCD1LG2	-0.01654187	-0.01851512	-2.29993241	0.021465446	0.039872202	-4.56935999	ns
PAIP2B	-0.02674294	0.038378239	-2.29801414	0.021574689	0.040049646	-4.56285153	ns
CETN3	-0.03866812	0.020868665	-2.29302561	0.021859887	0.040553285	-4.59055715	ns
ATP6V1F	-0.03593439	0.046970394	-2.29250496	0.02189027	0.040577812	-4.57871587	ns
CCDC50	-0.03861297	0.154310522	-2.29231306	0.021900901	0.040577812	-4.59414323	ns
ARAF	-0.0336298	0.068298952	-2.29159496	0.021942577	0.040629248	-4.58696489	ns
BNIP2	-0.01703686	0.009900216	-2.29086439	0.021984626	0.040681311	-4.59655921	ns
GP5	-0.01799083	-0.03932689	-2.28832015	0.022132593	0.040929178	-4.58983321	ns
BLOC1S2	-0.03674302	0.087829121	-2.28702381	0.022208224	0.041043046	-4.59241337	ns
VASP	-0.03593506	0.021510264	-2.28528352	0.022309668	0.041204447	-4.60811505	ns
TMEM106A	-0.03472484	0.019383319	-2.28253366	0.022471378	0.04147688	-4.61449721	ns
PSTPIP2	-0.05402673	-0.0979737	-2.27850345	0.022710984	0.041892655	-4.59460801	ns
CSF2RB	-0.02139275	-0.04826485	-2.27518385	0.022908915	0.042231081	-4.6171793	ns
CXCL1	-0.05131002	0.068295683	-2.27411636	0.022972768	0.042322071	-4.62837441	ns
ENDOU	-0.02578836	0.032050028	-2.27319082	0.023029369	0.042399595	-4.6022196	ns
ATP2B4	-0.03107062	0.152626899	-2.27234655	0.023079646	0.042465386	-4.62426132	ns
EIF4B	-0.03031332	-0.04025364	-2.27069305	0.023179431	0.042611065	-4.63507565	ns
SSH3	-0.0148013	0.020138762	-2.27042147	0.02319612	0.042611065	-4.62689188	ns
APP	-0.03511599	0.003109772	-2.27030937	0.0232026	0.042611065	-4.63950101	ns
EIF2AK3	-0.03370506	0.105939308	-2.26984653	0.023230993	0.042636393	-4.62990597	ns
HMOX1	0.024602347	0.017567842	2.268226653	0.023329447	0.042790192	-4.63562675	ns
PNMA1	-0.02753856	0.061157013	-2.26477849	0.023540408	0.043150026	-4.64225542	ns
CASP10	-0.03845685	-0.00639692	-2.26333754	0.023629053	0.043285342	-4.64592781	ns
OSMR	-0.01068526	-0.01526847	-2.25896353	0.023899589	0.043753479	-4.66514578	ns
TPSD1	-0.01805182	0.031378876	-2.25770539	0.023977865	0.043869276	-4.6724307	ns
IL36G	-0.03316646	0.077347882	-2.25442437	0.02418388	0.04421849	-4.66210165	ns
BTC	-0.03951537	0.089865594	-2.25066528	0.024421127	0.044624338	-4.67344257	ns
STAT5B	-0.07287735	-0.04174943	-2.24718965	0.024642103	0.044999964	-4.68858537	ns
MUC2	0.04512831	0.023005902	2.244778645	0.024796406	0.045253441	-4.70004145	ns
FAM3D	-0.04103893	0.096593424	-2.24429121	0.024827756	0.045282353	-4.70049124	ns
CLEC4C	-0.02435637	-0.00674288	-2.24094297	0.025044014	0.045648265	-4.70501302	ns
PCDH17	-0.01790397	0.01419953	-2.23519193	0.025419115	0.046303067	-4.71562347	ns
IL3RA	-0.01631958	-0.01526634	-2.23356847	0.025526062	0.046468891	-4.71279419	ns
TIA1	-0.06294741	0.008295272	-2.23288854	0.025571059	0.046521802	-4.70915484	ns
PRDX5	-0.03586405	-0.12997699	-2.23207166	0.02562443	0.046589873	-4.72971373	ns
TARS1	-0.01801008	0.01433718	-2.22844785	0.025864997	0.046998004	-4.73579531	ns
CRTAP	-0.02464223	0.062242733	-2.22762585	0.025920137	0.047068906	-4.72522976	ns
DXO	-0.02213633	0.016189572	-2.22538767	0.026069556	0.047310816	-4.74353003	ns
PKD1	0.012431363	-0.01444359	2.225018015	0.026094376	0.047326447	-4.74439477	ns
TNC	-0.02464221	0.00349746	-2.22218169	0.026285546	0.047633083	-4.74573205	ns
HGS	-0.04054273	0.049616302	-2.22203036	0.026296072	0.047633083	-4.73710758	ns
LUZP2	-0.0209598	0.030133536	-2.22085144	0.026375469	0.047747285	-4.75259895	ns
CRKL	-0.05591677	-0.08170865	-2.21931458	0.026479883	0.04789699	-4.75239187	ns
MAP4K5	-0.05868368	-0.05973833	-2.21915329	0.026490972	0.04789699	-4.74896679	ns
MRPL28	-0.01772872	0.041628354	-2.21822082	0.026554195	0.047954877	-4.76029691	ns
SLAMF1	-0.03071969	-0.07847509	-2.21820261	0.026555834	0.047954877	-4.74679843	ns
WNT9A	-0.01443615	-0.0054908	-2.21748704	0.026604652	0.048013339	-4.74837625	ns
IFNL2	-0.03001743	0.110522125	-2.21450259	0.026809121	0.048352461	-4.75469289	ns
KLK6	-0.01516026	-0.00094985	-2.21183795	0.026992481	0.048653114	-4.77024189	ns
SCGB2A2	0.014946885	0.01818387	2.207945707	0.027263099	0.04911058	-4.76768823	ns
SMAD2	-0.03312094	0.062553313	-2.20008667	0.027815496	0.050074752	-4.79823909	ns
NFATC1	-0.05022548	0.017820846	-2.19821455	0.027948699	0.05028355	-4.79931181	ns
SEC31A	-0.03716077	-0.07002778	-2.19774257	0.027982605	0.050308169	-4.79096082	ns
GIMAP8	-0.02454475	0.021448399	-2.19754378	0.02799684	0.050308169	-4.78874807	ns
LAIR2	-0.05403997	-0.13744056	-2.19564936	0.028132039	0.050520021	-4.80169935	ns
CASP2	-0.03471784	0.02706732	-2.19262776	0.02834917	0.050878659	-4.81150737	ns

TBCC	-0.04215033	-0.01503337	-2.19180279	0.028408795	0.05095435	-4.81008935	ns
CRTAM	-0.02569355	-0.01534863	-2.19110218	0.028459456	0.05101388	-4.81161592	ns
MRPS16	-0.01404592	0.03521769	-2.18888758	0.028620337	0.051270787	-4.80867263	ns
CETN2	-0.03470736	0.059669333	-2.18839652	0.02865603	0.051303255	-4.81176827	ns
FCGR2B	-0.02960837	0.025043393	-2.18537347	0.028876845	0.051666903	-4.81741917	ns
LRTM2	0.012908241	0.040097765	2.182895901	0.029058407	0.051959919	-4.83759349	ns
SIRT1	-0.04244505	0.197516286	-2.18203676	0.029122247	0.052042204	-4.82355775	ns
PCSK7	-0.02173745	0.031842952	-2.17997468	0.029274381	0.052282074	-4.84203595	ns
XRCC4	-0.01858711	0.008474459	-2.17279395	0.029811359	0.053208538	-4.84559411	ns
RRM2B	-0.03444512	0.014017249	-2.17153805	0.0299061	0.053345029	-4.84738415	ns
STX7	-0.02452408	-0.01084607	-2.1673501	0.030223814	0.053878839	-4.85531106	ns
COX5B	-0.03515545	0.011783154	-2.16307323	0.030550986	0.054425278	-4.87301179	ns
FKBPL	-0.02794196	0.027985797	-2.16285485	0.030567622	0.054425278	-4.87892691	ns
FKBP7	-0.02385922	0.046984911	-2.16214687	0.030622582	0.054489908	-4.86737617	ns
NXPH3	0.014346223	0.00862956	2.158803814	0.030881161	0.05491656	-4.87368992	ns
CD244	-0.01358557	-0.00300112	-2.15794661	0.030947262	0.055000611	-4.89150258	ns
ALDH2	-0.02472038	0.109272298	-2.1561606	0.031086512	0.055214486	-4.89337494	ns
TIMP4	-0.01933041	-0.00530411	-2.15495498	0.031180841	0.055348362	-4.89328527	ns
AARSD1	-0.02876522	0.006943245	-2.15385163	0.03126738	0.055468257	-4.89280264	ns
CHM	-0.0286045	0.04361748	-2.15159597	0.031444983	0.055749453	-4.89074036	ns
CD58	0.008848046	-0.00246532	2.15042671	0.031537095	0.0558486	-4.89973867	ns
TMCO5A	-0.02572549	0.040031296	-2.15040304	0.031539158	0.0558486	-4.89329149	ns
CDC37	-0.04229281	0.032377899	-2.14939924	0.031618261	0.055954741	-4.90665618	ns
VMO1	-0.03388145	-0.09710761	-2.14823261	0.031710933	0.056084752	-4.90482042	ns
SMS	-0.02991753	0.122758021	-2.14651199	0.031848059	0.05626593	-4.9020884	ns
MGMT	-0.06202674	-0.18246909	-2.1464602	0.031851912	0.05626593	-4.91110072	ns
PDGFA	-0.03606149	-0.04660387	-2.14614434	0.031876932	0.056276082	-4.91615573	ns
KDM3A	-0.04279074	0.255803182	-2.14398992	0.032049683	0.056546872	-4.90537542	ns
LATS1	-0.03967378	-0.00192792	-2.1376588	0.032560525	0.057413487	-4.91885056	ns
IL20	-0.02502585	0.048024629	-2.13568017	0.032721524	0.057662554	-4.92540545	ns
ENO1	-0.03941413	-0.04632522	-2.13470964	0.032800561	0.057766971	-4.93577797	ns
LRRC38	-0.02221768	0.009561434	-2.1320949	0.033015121	0.058109797	-4.93226703	ns
NARS1	-0.02210506	-0.05037414	-2.13137933	0.033073567	0.058177599	-4.94809706	ns
PDP1	-0.02539499	0.069024917	-2.12868321	0.033296681	0.058534804	-4.93899296	ns
AKR7L	-0.03222874	0.074744932	-2.12802068	0.033351588	0.058596052	-4.93928788	ns
AGR2	-0.04901629	-0.02632782	-2.1277334	0.033375479	0.058602766	-4.93941204	ns
MTSS2	-0.04624616	-0.01262639	-2.12726684	0.033413712	0.058634639	-4.95490809	ns
HDGF	-0.04732806	0.043572348	-2.1263749	0.033487824	0.058701721	-4.95866244	ns
RBPMS	-0.03064047	0.131082483	-2.12632866	0.033492147	0.058701721	-4.94286621	ns
GGT5	0.010595889	-0.00087408	2.125596908	0.033552841	0.058772823	-4.9521909	ns
CRYM	-0.03453536	0.103205441	-2.12524633	0.033582277	0.058789118	-4.94724685	ns
CEP290	-0.02499146	0.057710574	-2.1246531	0.03363159	0.058811382	-4.95347743	ns
RILPL2	-0.04562583	-0.07044128	-2.12460622	0.033635277	0.058811382	-4.96154048	ns
SEPTIN7	-0.02480316	0.063744073	-2.12427018	0.033663584	0.058825652	-4.95428629	ns
C1QTNF5	-0.01747472	0.003171889	-2.1237864	0.03370417	0.05886135	-4.95070069	ns
MYOC	0.022859603	-0.01576526	2.12352687	0.033725656	0.058863668	-4.96013636	ns
VPS4B	-0.04366789	0.019127495	-2.12055782	0.033974918	0.059263299	-4.96907716	ns
MYL4	-0.02432927	0.067817221	-2.12005144	0.034017994	0.059303011	-4.9577168	ns
CPXM1	-0.03400109	-0.0013521	-2.11929442	0.034081513	0.059378292	-4.9705472	ns
SYAP1	-0.02607443	0.062045785	-2.11486896	0.034457406	0.059997392	-4.96949067	ns
REG3A	-0.02973294	0.007334635	-2.11440899	0.034496261	0.06002925	-4.98338081	ns
TIMM8A	-0.04021977	0.032083689	-2.11393382	0.034536849	0.060031507	-4.98398411	ns
LDLRAP1	-0.04486143	-0.0308755	-2.11391216	0.034538675	0.060031507	-4.98496559	ns
FAM172A	-0.0283959	0.025463418	-2.10825636	0.035025275	0.060841049	-4.98250935	ns
ROR1	-0.01503311	-0.00128194	-2.10604424	0.035216748	0.06113728	-4.99435351	ns
PAK4	-0.030095	0.074696987	-2.10473572	0.035330835	0.061298893	-4.99031337	ns
ARHGEF10	-0.04314953	0.042159981	-2.10411302	0.035384629	0.06135577	-5.00551618	ns
MANSC4	-0.02917553	0.052623327	-2.10246384	0.035529251	0.061569977	-4.99370459	ns
FGD3	-0.0369866	0.024898097	-2.10131165	0.035629696	0.061707421	-5.01137363	ns
PRAME	0.011716257	-0.00051939	2.100943978	0.035662503	0.061727628	-4.99515657	ns
ELOB	-0.01783816	0.021645314	-2.09786433	0.035933784	0.062145716	-5.00330845	ns
ITGB7	0.022395603	0.023779023	2.09771324	0.035946615	0.062145716	-5.01963307	ns
F11	-0.01395633	0.004171939	-2.09448729	0.036233065	0.062603876	-5.01642764	ns
S100A4	-0.02790059	-0.02685058	-2.09394891	0.036280801	0.062649285	-5.02363928	ns
SEMA3G	-0.01968433	0.028069017	-2.09222281	0.036435195	0.062878705	-5.01685332	ns
CERT	-0.0296453	-0.00329397	-2.08815349	0.036800739	0.063472037	-5.0248766	ns

TRIM24	-0.02937241	0.040235476	-2.08577369	0.037015732	0.063805158	-5.03347029	ns
EPHA4	-0.0125813	0.001125959	-2.08364356	0.037209326	0.064101021	-5.03466305	ns
BRK1	-0.02131075	-0.01238464	-2.08082294	0.037466965	0.064506803	-5.04006896	ns
CDK5RAP3	-0.02710269	0.097683472	-2.07986327	0.037554444	0.064619314	-5.05596207	ns
HIF1A	-0.03000251	0.100497978	-2.07789736	0.03773574	0.064893028	-5.04408452	ns
PSMG4	-0.0266432	0.064253249	-2.07615577	0.037896497	0.065131119	-5.04768091	ns
CHIT1	-0.07395561	-0.33566846	-2.07393996	0.038101502	0.065444933	-5.06283235	ns
TNXB	0.012199348	0.005160074	2.070027987	0.038466388	0.066032835	-5.07167687	ns
CCNE1	-0.04340266	0.258550342	-2.0697041	0.038497102	0.066046732	-5.06097742	ns
PPM1F	-0.02384479	0.009556691	-2.06853762	0.038606053	0.0661721	-5.07843219	ns
STAMBP	-0.03169228	0.012628826	-2.06844001	0.038615499	0.0661721	-5.07021512	ns
DENND2B	0.022462647	0.084360074	2.06767745	0.038687434	0.066256485	-5.0658174	ns
FZD8	-0.01886768	0.031289131	-2.06531354	0.038910393	0.066599266	-5.07209868	ns
HMGCL	-0.02192326	0.036305756	-2.06451611	0.038985869	0.06668936	-5.07324187	ns
BAG6	-0.01745394	0.019409766	-2.06228905	0.03919733	0.066998753	-5.07808219	ns
TBCB	-0.04899647	-0.11186307	-2.06212594	0.039212626	0.066998753	-5.08433292	ns
PDLIM5	-0.05353625	-0.09499585	-2.06184451	0.039239149	0.067004863	-5.09306739	ns
RAB3GAP1	-0.02610348	0.078988306	-2.05942581	0.039470595	0.067347761	-5.08417144	ns
CA13	-0.05050985	-0.11725211	-2.05926185	0.039486085	0.067347761	-5.09007314	ns
NTF4	-0.01919348	0.050154806	-2.0582866	0.039579816	0.067468221	-5.08551568	ns
LEFTY2	0.02489582	0.002759612	2.054078263	0.039984852	0.068118884	-5.10888914	ns
CDC25A	0.018530876	0.022917025	2.052443201	0.040143897	0.068349958	-5.09529383	ns
TTF2	-0.0222303	0.04208929	-2.05126745	0.040258106	0.06850447	-5.10035668	ns
PKN3	-0.03341206	0.05040816	-2.05038969	0.040343456	0.068609722	-5.10761942	ns
PCDH7	-0.01531249	0.044885238	-2.049585	0.040421778	0.068702905	-5.11615476	ns
NID2	-0.03585683	0.02735187	-2.0490369	0.040475449	0.068754108	-5.11466452	ns
RASGRF1	0.019855334	0.009859614	2.046641985	0.040710614	0.069113368	-5.10711482	ns
GPR15L	-0.02689989	-0.06430246	-2.04251759	0.04111714	0.069762957	-5.13053386	ns
HSP90B1	-0.03221316	0.15075284	-2.0401297	0.041354907	0.070125626	-5.12074808	ns
IFNL1	-0.02096952	-0.01199736	-2.03725588	0.041641843	0.070505217	-5.12925877	ns
CTSH	-0.0356472	-0.02036375	-2.0371655	0.041650943	0.070505217	-5.12817504	ns
ANGPTL3	-0.01664365	0.002493272	-2.03699003	0.041668227	0.070505217	-5.13624763	ns
PECAM1	-0.01434607	-0.00117818	-2.03681017	0.04168617	0.070505217	-5.13950182	ns
IL17RA	-0.02120509	-0.04241618	-2.03667734	0.04169949	0.070505217	-5.13977083	ns
BMP4	0.021964697	0.039707954	2.035120157	0.041856051	0.070728975	-5.13842909	ns
OPHN1	-0.04867066	-0.01509244	-2.03161968	0.042209195	0.071284471	-5.1536458	ns
PLPBP	-0.03221174	0.002447923	-2.02899136	0.042476441	0.07169434	-5.15275686	ns
PPP1R12B	-0.03214574	0.110767768	-2.02856531	0.042519907	0.071726244	-5.15187638	ns
C8B	-0.01345055	-0.01490046	-2.02632652	0.042748432	0.072070104	-5.16440827	ns
PRTFDC1	-0.0535578	-0.15489868	-2.02526173	0.04285809	0.072213285	-5.15348192	ns
TPSAB1	-0.02486123	-0.03114601	-2.02186472	0.043207472	0.072759988	-5.17368413	ns
STXBP3	-0.0204692	0.054212812	-2.02084918	0.043313061	0.072895757	-5.16143181	ns
KITLG	-0.01719286	-0.01029371	-2.01771644	0.043638216	0.073400686	-5.17544071	ns
IMPG1	-0.01385182	0.031766187	-2.01174608	0.044264466	0.074411192	-5.18066954	ns
DRG2	-0.02686357	0.044038546	-2.00911183	0.044542809	0.07483602	-5.19236244	ns
MENT	0.009586504	0.007306714	2.005544841	0.044922587	0.075430682	-5.19342548	ns
LAMP2	-0.00900933	-0.01160369	-2.00474377	0.045008372	0.075531291	-5.1934832	ns
CX3CL1	-0.01516281	0.008456181	-2.00437846	0.04504716	0.075552962	-5.20108087	ns
RBP2	0.035993236	0.011937797	2.003678819	0.045122078	0.075635171	-5.20320143	ns
GALNT5	0.01164605	-0.00234879	2.000012221	0.045516374	0.076252331	-5.21705117	ns
CMIP	-0.03618651	0.014338264	-1.99386578	0.046184272	0.077326877	-5.22824553	ns
PGR	-0.04562876	0.317079291	-1.98802072	0.046827503	0.078358915	-5.22574504	ns
FOLR3	-0.08828409	-2.48704626	-1.985264	0.047132811	0.078824632	-5.24323566	ns
SERPINA4	-0.01015644	-0.0074732	-1.98342662	0.047337787	0.079122117	-5.24053752	ns
PYDC1	-0.01788442	0.02500071	-1.97892106	0.047843058	0.079920898	-5.24529556	ns
TXN	-0.01905016	0.030304596	-1.97838868	0.047902891	0.079975096	-5.25013528	ns
ATG4A	-0.02799504	0.029637334	-1.97798173	0.047948987	0.08000631	-5.24664864	ns
NEB	-0.01618444	0.034835709	-1.97675678	0.048087376	0.080191398	-5.24861906	ns
COL1A1	0.011694432	0.012764636	1.97553733	0.0482249	0.080374833	-5.26577985	ns
ACP6	-0.03149351	-0.13529999	-1.96545599	0.049379313	0.082251907	-5.28600477	ns
PSMD1	-0.02116408	0.032713185	-1.9648185	0.049453056	0.082327779	-5.28732221	ns
KIR3DL1	-0.05627216	8.5951209784	-1.96392783	0.04955685	0.082436602	-5.27418911	ns
FLT3	0.014313581	0.013453869	1.96377235	0.049574888	0.082436602	-5.27449269	ns
NDST1	-0.02707465	0.123615671	-1.96262969	0.049707135	0.082609468	-5.28969245	ns
NCAN	0.017798156	0.031964853	1.959950888	0.050019413	0.083038768	-5.2968507	ns
TTN	-0.0285748	0.133064876	-1.9599308	0.050022326	0.083038768	-5.28154796	ns

CES2	-0.02554292	0.022069719	-1.95794849	0.050254207	0.083376297	-5.29327194	ns
LSM1	-0.02497532	0.064676575	-1.95502838	0.050598214	0.083899366	-5.29152727	ns
LILRB1	-0.01263995	-0.02574908	-1.95202757	0.050952899	0.084439537	-5.31169043	ns
DBH	0.046605196	-0.1734946	1.949379957	0.051268149	0.08491378	-5.31418095	ns
MUC16	-0.08573581	-0.57778733	-1.94807368	0.051424411	0.08508742	-5.31244854	ns
F3	0.012213339	0.001808745	1.948015192	0.051431266	0.08508742	-5.31740302	ns
CHL1	0.014049735	0.032665862	1.941115414	0.052262557	0.086413741	-5.32749592	ns
DKKL1	0.039465671	-0.12792802	1.939767194	0.052426346	0.086635501	-5.32850444	ns
CBLIF	0.031352503	0.023234483	1.937670596	0.05268182	0.087008436	-5.33233293	ns
TMOD4	0.01712196	-0.0037945	1.934805238	0.053032949	0.087538842	-5.32846516	ns
NAA10	-0.02143133	-0.00064846	-1.93349953	0.053193444	0.087754155	-5.3327066	ns
MCEE	-0.01811004	0.031594142	-1.9323193	0.053338279	0.087943408	-5.3502966	ns
ENPP7	-0.03928397	-0.08705465	-1.92635109	0.054079632	0.08911542	-5.33893854	ns
CASP3	-0.03957585	-0.05198916	-1.92358187	0.054425296	0.089634441	-5.3665056	ns
ROBO2	-0.00999074	-0.0017756	-1.92059245	0.054801424	0.090176864	-5.37279181	ns
CDON	-0.01182586	-0.01290001	-1.92047508	0.054816416	0.090176864	-5.36710483	ns
FXYD5	-0.02899515	0.054979395	-1.91771642	0.055165813	0.090700548	-5.3646143	ns
ITGB6	0.013558376	-0.00077488	1.916108677	0.055370116	0.090985221	-5.36767859	ns
CLEC12A	-0.00806053	-0.00314861	-1.90977597	0.056181074	0.092265881	-5.37735903	ns
EIF1AX	-0.02269017	0.097428929	-1.90753787	0.056469434	0.092687322	-5.39667028	ns
LRRC37A2	0.017777939	0.009153847	1.905755082	0.056700514	0.093014327	-5.39901082	ns
KLK7	-0.01626507	-0.00131242	-1.90499162	0.056800581	0.093126162	-5.37628694	ns
IGBP1	-0.02564957	0.021935775	-1.90451618	0.056861457	0.093173656	-5.40333807	ns
CLEC2L	-0.02184332	0.095013194	-1.90389386	0.056942979	0.093224701	-5.39012692	ns
VASH1	-0.03601442	0.036014577	-1.90378821	0.056956461	0.093224701	-5.39976775	ns
ATF2	-0.031151	0.154533829	-1.9024522	0.057131108	0.093458171	-5.39327978	ns
ITGB2	0.01264189	0.001628109	1.901037941	0.057315983	0.093708102	-5.39813439	ns
KLK12	-0.06141079	-0.54089191	-1.8995021	0.0575176	0.093950377	-5.39792758	ns
ALPI	0.051338478	0.218777755	1.899246521	0.057551192	0.093950377	-5.39729737	ns
ENTPD2	0.018948496	-0.0141158	1.898990647	0.057584821	0.093950377	-5.39889339	ns
INSL5	-0.03963028	0.07745515	-1.89892549	0.057592868	0.093950377	-5.41193056	ns
BTD	-0.00849388	-0.0210061	-1.8941619	0.05822213	0.094923852	-5.42203762	ns
MAPK13	-0.01761483	-0.01175197	-1.89206826	0.058501097	0.095325449	-5.40976907	ns
PCDH12	-0.01016366	0.000365436	-1.89025689	0.058742221	0.095664967	-5.43024319	ns
FCGR2A	-0.02731407	-0.16488572	-1.88917274	0.058887697	0.095848426	-5.42174759	ns
C7	0.012593934	-0.02684234	1.886868785	0.059197136	0.096298405	-5.42353889	ns
CHP1	-0.03059225	0.05134312	-1.88644285	0.059254501	0.096338053	-5.42349855	ns
NPR1	0.017394398	0.083365112	1.88509888	0.05943576	0.096578975	-5.42458804	ns
GART	-0.01110305	0.018293948	-1.88392006	0.059594463	0.096782998	-5.44213498	ns
GET3	-0.02318649	0.09234847	-1.88354782	0.059644884	0.09681104	-5.44193851	ns
AKT2	-0.05190222	-0.03975172	-1.8807634	0.060022815	0.097370344	-5.44804391	ns
MINDY1	-0.0445146	-0.0426184	-1.87730937	0.06049453	0.098081081	-5.45259053	ns
PPBP	-0.03438582	-0.07303779	-1.8711534	0.06134281	0.099401223	-5.46052946	ns
DGKZ	-0.0152346	0.008008366	-1.86927924	0.061603308	0.099767975	-5.45580631	ns
VEGFC	-0.02406701	-0.02186157	-1.86693382	0.061929772	0.100179925	-5.47070668	ns
IFNG	-0.04955199	-0.05072172	-1.86676461	0.061953824	0.100179925	-5.46047691	ns
TRAF2	-0.03096655	0.053272983	-1.86671398	0.061960597	0.100179925	-5.46927003	ns
CRIM1	-0.00850964	-0.01135655	-1.86639163	0.062005566	0.100197152	-5.47102615	ns
UNC79	-0.02308166	0.06290999	-1.86461917	0.062254289	0.100543431	-5.46103165	ns
FGFR4	-0.02134785	0.040690262	-1.86427861	0.062301921	0.100564736	-5.46519997	ns
ITGA6	-0.0230485	0.070687955	-1.86282816	0.062505541	0.100837668	-5.4764756	ns
BCAT1	-0.01212648	0.004959264	-1.86126197	0.062726123	0.101106272	-5.48343472	ns
SERPINE2	-0.02593162	0.019631934	-1.86115453	0.062741289	0.101106272	-5.48363357	ns
NRP2	-0.01236972	-0.00977445	-1.85754766	0.06325239	0.101858912	-5.48659646	ns
ASS1	-0.03165837	0.017910722	-1.85736978	0.063278105	0.101858912	-5.47617866	ns
RPS10	0.015992303	0.03458539	1.856683131	0.063375798	0.101959962	-5.47744649	ns
LACRT	0.020123115	0.134445009	1.855607403	0.063528608	0.102149525	-5.49206637	ns
PTPN6	-0.03892458	-0.16876903	-1.85461331	0.063670589	0.102321475	-5.49170305	ns
STAM	-0.0151827	0.041060788	-1.85291978	0.063912911	0.102654401	-5.49782662	ns
ARNT	-0.01617159	0.047674603	-1.85241677	0.063985492	0.102714479	-5.48700589	ns
BCL2L1	-0.04015022	-0.02771123	-1.85189206	0.064060326	0.102778106	-5.50068693	ns
WAS	-0.02905122	0.044807297	-1.85134203	0.064139866	0.10280341	-5.48898483	ns
NEDD4L	-0.01982475	-0.03797548	-1.85129646	0.064146511	0.10280341	-5.48670125	ns
RASA1	-0.0140718	-0.0007947	-1.85096306	0.064194424	0.102823761	-5.48935809	ns
ADGRE5	-0.01286213	0.006426324	-1.85043929	0.064269376	0.102887378	-5.50049254	ns
ADAM15	0.013107518	0.009517776	1.847279247	0.064726272	0.103557677	-5.49597071	ns

PEAR1	0.009741485	-0.00098816	1.847049402	0.064759013	0.103557677	-5.5099916	ns
MYL1	0.016148189	0.036114753	1.843479363	0.065278724	0.104331622	-5.50173483	ns
TRAF3IP2	0.0188626	0.067928226	1.841596973	0.065553813	0.104680841	-5.50518338	ns
FAP	0.011417803	-0.00573277	1.841489605	0.065568924	0.104680841	-5.52018848	ns
B3GAT3	0.00817281	0.005472928	1.839110769	0.065918606	0.105136902	-5.50973272	ns
SRC	-0.05227358	-0.05705954	-1.83905318	0.065926599	0.105136902	-5.52097435	ns
SDHB	-0.02721742	0.110010598	-1.83778278	0.066113593	0.10537756	-5.52553054	ns
CD276	-0.01465904	-0.04616761	-1.83752522	0.066151746	0.105380851	-5.5207515	ns
IGSF21	0.015909456	0.029836018	1.836261645	0.066338646	0.105620963	-5.51587411	ns
CHRDL2	-0.02327704	0.019447355	-1.83392396	0.066684684	0.106114047	-5.52739451	ns
ITGAX	-0.02146634	0.048829583	-1.83329889	0.066777593	0.106204015	-5.52678673	ns
PRSS22	-0.01326437	0.007900904	-1.82867795	0.067467667	0.107243107	-5.52808079	ns
STK24	-0.02623939	0.03087498	-1.82821851	0.067536524	0.107294151	-5.53096024	ns
COMMD9	-0.01562284	0.046024029	-1.82737293	0.067662996	0.107436622	-5.5445751	ns
SCGN	-0.01864739	0.019422048	-1.82704001	0.067713475	0.10745834	-5.53342629	ns
PAEP	-0.05805846	0.603761557	-1.8257276	0.067910823	0.10771193	-5.54127284	ns
COL9A2	-0.01843628	0.034086214	-1.82547396	0.067949063	0.10771193	-5.54102083	ns
SLITRK1	0.013388344	0.037649771	1.825244744	0.067983934	0.10771193	-5.53431724	ns
PROCR	0.014043918	0.035921165	1.823971807	0.068176179	0.10795794	-5.5373019	ns
ATP5PO	-0.01714935	-0.00259435	-1.8209741	0.068630616	0.108618644	-5.54410306	ns
CLSPN	-0.01524938	0.036394742	-1.81778684	0.069116616	0.109328559	-5.54869789	ns
ACTN4	-0.0134174	0.020719715	-1.81593451	0.069400233	0.109717747	-5.55353966	ns
FIS1	-0.03276318	0.041544459	-1.81299126	0.069852721	0.110373346	-5.56588785	ns
FGL1	-0.02449116	-0.03821862	-1.81272221	0.069894041	0.110378907	-5.57095341	ns
PMS1	-0.02023702	0.028484365	-1.81054871	0.070230586	0.110811036	-5.56249974	ns
COPB2	-0.01705989	0.014551357	-1.8104656	0.070243571	0.110811036	-5.56170546	ns
TXNL1	-0.03978135	0.30466603	-1.80806829	0.070615828	0.111338131	-5.56673945	ns
CDKL5	0.020766708	0.076932762	1.805049363	0.071086899	0.112020369	-5.57287374	ns
NECAP2	-0.02618475	0.090447479	-1.80011177	0.071863032	0.113182337	-5.58029041	ns
C2CD2L	-0.01797873	0.003650349	-1.79801647	0.072194455	0.113643024	-5.58423679	ns
CR2	-0.01943917	0.002131041	-1.79654467	0.072427586	0.113948574	-5.59615724	ns
EIF4E	-0.03534408	0.016366396	-1.7883331	0.073742412	0.115954681	-5.60062758	ns
ERCC1	0.01263003	0.004867171	1.788065925	0.073785519	0.115960019	-5.60004254	ns
C7orf50	-0.00938274	0.021179467	-1.78654003	0.0740314	0.116283856	-5.61891703	ns
VSTM2B	-0.01664797	0.074227913	-1.78491851	0.074294384	0.116634194	-5.61381896	ns
CHMP1A	-0.03307787	-0.05802189	-1.78271819	0.074652048	0.117132714	-5.6184349	ns
ANXA4	-0.0298078	0.023679777	-1.78109115	0.074917481	0.117451187	-5.62042094	ns
FCRL2	-0.02020015	0.008403667	-1.78097894	0.074935466	0.117451187	-5.62916188	ns
CALCOCO1	-0.04382069	0.011091652	-1.77868013	0.07531186	0.117977807	-5.63253017	ns
CPA4	-0.01678669	-0.00519132	-1.77789413	0.075441743	0.118084532	-5.61032505	ns
OBP2B	0.026329572	-0.03002272	1.777584635	0.075492071	0.118084532	-5.62546628	ns
CA4	-0.00972153	-0.00525554	-1.7775261	0.075501308	0.118084532	-5.6352898	ns
DNAJA1	0.01765526	0.037094083	1.775370734	0.075856934	0.118577219	-5.62253236	ns
TIMP2	-0.0097019	-0.00708621	-1.77493629	0.075928044	0.118624873	-5.63951302	ns
ECSCR	-0.02009468	0.102597749	-1.77324458	0.07620837	0.118995958	-5.62945352	ns
PTK7	-0.01545796	-0.0118655	-1.77301108	0.076247068	0.118995958	-5.6298018	ns
RABGAP1L	-0.0250331	0.039207529	-1.77189285	0.076432499	0.119221633	-5.6320974	ns
ACTA2	-0.01754064	0.001956434	-1.77012444	0.076726633	0.119599692	-5.63472601	ns
TGFB2	-0.02060981	-0.02043669	-1.7699404	0.076756789	0.119599692	-5.64730293	ns
FLRT2	-0.01142286	-0.00756602	-1.76861465	0.076978164	0.119880661	-5.64333738	ns
MYH4	0.018003274	0.062714261	1.767837583	0.077108342	0.120019381	-5.63753839	ns
WASF3	-0.03456596	-0.02752928	-1.76741105	0.07717965	0.120066371	-5.63965869	ns
DHPS	-0.0157262	0.015589513	-1.75726616	0.078892257	0.122665276	-5.66953134	ns
UBE2Z	-0.02297776	0.096815073	-1.75669253	0.078990586	0.122752799	-5.65639076	ns
LTB	-0.01310078	0.01994736	-1.75369664	0.079502328	0.123423818	-5.67576283	ns
TMED8	-0.03953391	-0.03805682	-1.75366977	0.079506918	0.123423818	-5.67573582	ns
CRYZL1	-0.03542317	-0.00707406	-1.75230755	0.079741263	0.12367066	-5.66565383	ns
NOP56	-0.04176616	0.32764567	-1.75225339	0.079750635	0.12367066	-5.66481101	ns
CLEC4M	0.018429316	0.040276335	1.74986155	0.080162054	0.124242674	-5.68202977	ns
DHODH	-0.01815606	0.04496766	-1.74912223	0.080290302	0.124375428	-5.67026148	ns
RFC4	-0.00818653	0.007016726	-1.74858158	0.080383232	0.124390102	-5.68467039	ns
TMEM25	0.016803493	0.030939852	1.74857097	0.080384974	0.124390102	-5.68660149	ns
SOX9	-0.01015514	-0.01590623	-1.74706891	0.080645695	0.124727452	-5.67477561	ns
MELTF	0.010942259	0.000390219	1.746300149	0.080778634	0.12486692	-5.68965141	ns
CREBZF	0.009930298	0.007433767	1.738999204	0.082055387	0.126773402	-5.68607852	ns
SCN3B	-0.01834227	0.065220673	-1.73509714	0.082744033	0.127769738	-5.69599059	ns

PACS2	-0.02378497	0.04715147	-1.73304418	0.083108323	0.128264431	-5.69809532	ns
SEZ6	0.011636088	0.016932741	1.729142911	0.083803938	0.129269678	-5.70575589	ns
MAMDC4	0.020483609	0.011583313	1.728031238	0.084002605	0.129507712	-5.72019532	ns
DNM1	-0.04302743	-0.09300698	-1.72670816	0.084240061	0.129805265	-5.72239421	ns
MMP13	0.012675484	-0.00648146	1.726094889	0.084350842	0.129907415	-5.71141801	ns
IL3	0.02263215	-0.06562363	1.723786157	0.084767028	0.130416598	-5.71226786	ns
ZNRF4	-0.02047746	0.023850275	-1.72376219	0.084770789	0.130416598	-5.72752248	ns
GIT1	-0.03710692	0.003260664	-1.72331523	0.084852095	0.130472943	-5.71413634	ns
COMP	-0.01363393	-0.03413648	-1.72275277	0.084953703	0.130560428	-5.71797523	ns
PKLR	-0.0273327	0.06966314	-1.72203948	0.0850825	0.130689585	-5.72683389	ns
B4GAT1	0.009981368	0.014502876	1.719899673	0.085470812	0.131217019	-5.73143659	ns
MAEA	-0.01577481	0.026024076	-1.71825634	0.085770447	0.131607833	-5.72390904	ns
VIPR1	0.008912143	0.008147347	1.716408554	0.086107831	0.132056128	-5.72756187	ns
DGCR6	-0.01639858	0.039580816	-1.7151611	0.086336242	0.132336916	-5.73018775	ns
IL17D	-0.01349666	0.000777489	-1.71323896	0.086689105	0.132787871	-5.73372578	ns
TBL1X	-0.03449341	-0.04315182	-1.71306388	0.086721394	0.132787871	-5.73276691	ns
INPP1	-0.02161582	0.027317281	-1.71237506	0.086847838	0.132911786	-5.74140392	ns
PLA2G4A	-0.0354776	-0.02162898	-1.71123741	0.087057412	0.133162726	-5.74702192	ns
EPN1	-0.01624462	0.069220627	-1.70846056	0.087571246	0.133878554	-5.74661675	ns
NTRK2	-0.01009323	-0.00515125	-1.70729527	0.087787464	0.134138877	-5.75034766	ns
HTR1B	-0.01604124	0.067602951	-1.7067943	0.087880883	0.13421139	-5.74442199	ns
SMARCA2	-0.02537176	0.053405972	-1.69870418	0.089395579	0.136453262	-5.75805586	ns
RLN2	-0.04561234	0.209122159	-1.69116692	0.090825541	0.138563522	-5.77041802	ns
DCUN1D1	-0.02817417	0.195712265	-1.68962283	0.091120808	0.138941388	-5.77341748	ns
SF3B4	-0.0298203	0.043033013	-1.68789968	0.091450872	0.139371893	-5.7827827	ns
CDH1	-0.01568285	0.007051752	-1.6874252	0.091542805	0.139439223	-5.76466514	ns
CSF1R	-0.01437961	0.001406766	-1.6863519	0.091748192	0.139621059	-5.79081754	ns
NT5C	-0.02741412	0.006915487	-1.68630187	0.091757812	0.139621059	-5.79205788	ns
LIPF	0.021543505	0.015005395	1.685054208	0.091998807	0.139914852	-5.78058468	ns
DAND5	0.0209214	-0.02889761	1.683941009	0.092213828	0.140168858	-5.78083355	ns
VIM	-0.04538748	-0.15861497	-1.68367143	0.092265902	0.140175044	-5.78317415	ns
SSNA1	-0.01879313	0.035008125	-1.68040493	0.092898756	0.141063113	-5.80083805	ns
PAFAH2	-0.01836765	0.031797092	-1.67943637	0.093087491	0.141276234	-5.79550651	ns
IL6R	-0.01347061	-0.02117648	-1.67710243	0.093543057	0.141893883	-5.79685275	ns
ECM1	-0.01350476	-0.05004231	-1.67550653	0.093855663	0.142294152	-5.79447663	ns
PSPN	-0.03857421	-0.13095437	-1.67279562	0.094387924	0.143026849	-5.81054271	ns
FGFBP1	0.012318144	0.004076195	1.672007781	0.094543055	0.143187615	-5.81370505	ns
RHOC	-0.04632801	-0.14197442	-1.66998785	0.094942395	0.143717882	-5.80855182	ns
GAGE2A	-0.02306306	0.102086336	-1.66941882	0.095055241	0.14375666	-5.8039816	ns
KIR2DL2	-0.0306841	-0.03595269	-1.66935919	0.095066476	0.14375666	-5.81931012	ns
DPP10	-0.01299818	0.009161886	-1.66899581	0.09513861	0.143791274	-5.81685649	ns
DIPK2B	-0.00977068	0.021271387	-1.66704799	0.095525539	0.144301383	-5.81146554	ns
SHD	-0.03105149	0.212118268	-1.66426159	0.096081049	0.14500947	-5.81327873	ns
SV2A	-0.02232523	0.061479963	-1.66419796	0.096093604	0.14500947	-5.81532113	ns
ARMCX2	-0.02305375	0.119234436	-1.66157371	0.096619067	0.145727105	-5.82015045	ns
CASQ2	0.017429982	0.063475369	1.660659017	0.096802804	0.145928853	-5.82022418	ns
LILRB5	-0.02765225	-0.1654132	-1.65587954	0.097766444	0.147305478	-5.8429684	ns
BMP6	-0.01596906	-0.01019986	-1.65505114	0.09793499	0.147483327	-5.82995307	ns
EDAR	-0.03596498	0.052893438	-1.65290909	0.098369826	0.1480618	-5.84342733	ns
PYY	-0.03449587	0.091013097	-1.65222846	0.098508873	0.148194698	-5.8341097	ns
TFAP2A	-0.02191706	0.062959625	-1.64940507	0.099085508	0.148985419	-5.84018155	ns
PDRG1	-0.01918907	0.06402353	-1.64547572	0.099892537	0.150121569	-5.84612793	ns
YJU2	-0.01179591	0.015210591	-1.64379228	0.100239462	0.150565447	-5.86142982	ns
VAV3	-0.03595359	0.016715733	-1.64283946	0.100437004	0.150784602	-5.85092821	ns
SUGT1	-0.03117038	-0.02594051	-1.64235857	0.100536618	0.150856589	-5.85071698	ns
SERPINA11	-0.01566338	-0.04257808	-1.64072186	0.10087544	0.151287255	-5.86781531	ns
HDDC2	-0.0156643	0.034345231	-1.64005616	0.101013831	0.151380903	-5.86745641	ns
MCTS1	0.009186559	0.037621962	1.63992276	0.101041568	0.151380903	-5.86767397	ns
IL17A	-0.01630162	0.032403889	-1.6372285	0.101603564	0.152144823	-5.86034056	ns
HAGH	-0.02840071	0.031452897	-1.63515877	0.102036101	0.152671543	-5.87682158	ns
AMBN	0.010262376	0.017408876	1.63479923	0.102111978	0.152671543	-5.86429242	ns
DOK1	-0.04876554	-0.15897007	-1.63479581	0.102112166	0.152671543	-5.87706766	ns
IGHMBP2	-0.02006726	0.082171458	-1.6335862	0.10236671	0.152973794	-5.86456352	ns
GPC5	0.021319841	0.023330055	1.629964188	0.103129691	0.15403514	-5.87762493	ns
FOSB	-0.01500805	0.025276102	-1.62491939	0.104200651	0.155555164	-5.87997837	ns
IGF2BP3	-0.01683619	0.075782969	-1.62317643	0.104572664	0.15603075	-5.88141851	ns

ITIH3	-0.01450978	-0.04103274	-1.62249585	0.104717753	0.156167436	-5.89203777	ns
BRDT	-0.01287131	0.00774433	-1.61940712	0.105380673	0.157075837	-5.88749503	ns
PCDH1	-0.00766259	0.012253928	-1.61502749	0.106325125	0.158402737	-5.90867614	ns
DDR1	-0.00819776	-0.00572732	-1.61238906	0.106897653	0.159174476	-5.91422245	ns
MGLL	-0.03314022	-0.09340847	-1.61031483	0.107349662	0.159766061	-5.91258314	ns
THRAP3	-0.04020164	0.382559705	-1.60884244	0.107671797	0.160163855	-5.90445115	ns
HGFAC	-0.00971415	-0.02443488	-1.60825366	0.107800267	0.160273309	-5.91317693	ns
TPM3	0.015176897	0.046215884	1.607948645	0.107867428	0.160291546	-5.90588058	ns
GIPC3	-0.03447813	-0.09430871	-1.60565796	0.108369503	0.160955721	-5.92297194	ns
ABCA2	-0.01279595	0.000989298	-1.60427782	0.108673836	0.161325674	-5.91174296	ns
GALNT2	0.0094398	0.01457175	1.600426263	0.109524406	0.162505724	-5.93405885	ns
ADRA2A	-0.02561814	0.188818132	-1.59980355	0.109663187	0.162629002	-5.91887034	ns
CA1	-0.02333024	0.002897596	-1.59944179	0.109743687	0.16266577	-5.91381108	ns
SPTBN2	-0.01118756	0.035285245	-1.59899138	0.10984347	0.162712061	-5.9216007	ns
HK2	-0.0099195	-0.00680668	-1.59879844	0.109886364	0.162712061	-5.9216827	ns
MAPT	-0.01890474	-0.05906622	-1.59829678	0.10999791	0.162736325	-5.92264123	ns
POLR2A	0.010346669	0.012630235	1.5982237	0.110014215	0.162736325	-5.92070395	ns
SPRED2	-0.01586295	0.055718089	-1.5973113	0.110217348	0.162954256	-5.92215349	ns
PTPRZ1	0.01143754	0.020720232	1.596524367	0.110392171	0.163130132	-5.93860847	ns
DSG4	0.01872808	0.019924538	1.594616828	0.110818648	0.163673884	-5.93388798	ns
GAD1	0.009178712	-0.00547326	1.59437886	0.11087224	0.163673884	-5.92574265	ns
WASL	-0.01990132	0.092964813	-1.59238315	0.111319662	0.164251346	-5.92996859	ns
COL4A4	0.018253006	-0.0504766	1.591824014	0.111444986	0.164353211	-5.93731455	ns
HRC	0.011673171	0.043459423	1.589869634	0.111885159	0.164919063	-5.93556379	ns
NELL2	-0.01048002	0.012721347	-1.58757621	0.11240271	0.165598342	-5.94985949	ns
MMP1	-0.03430518	-0.08202117	-1.57987397	0.114157221	0.16809838	-5.93754103	ns
USP28	-0.01281355	0.036867591	-1.57458632	0.115372372	0.169742195	-5.97315403	ns
CDH15	-0.02064352	0.121763737	-1.57451097	0.115389814	0.169742195	-5.97310297	ns
CD2	-0.01283477	0.022154011	-1.56753931	0.117009733	0.17203848	-5.96967717	ns
ACADSB	-0.02989622	0.063970581	-1.56566194	0.11744821	0.172596262	-5.98713678	ns
TP53BP1	-0.03055157	0.251193469	-1.56484046	0.117641367	0.172793154	-5.97320149	ns
CPOX	0.008948139	0.008684269	1.563082965	0.118054128	0.173229405	-5.97593459	ns
EGF	-0.03811663	-0.08533551	-1.56306912	0.118057026	0.173229405	-5.98461382	ns
ASRGL1	-0.03484837	0.018850975	-1.56223172	0.118254398	0.173415568	-5.97887662	ns
MST1	-0.022539	-0.17445556	-1.56202593	0.118302675	0.173415568	-5.98367998	ns
GALNT7	0.007566604	-0.00772705	1.559671375	0.118858245	0.174142537	-5.98983684	ns
FGF7	0.027334773	0.183344146	1.555143998	0.119932843	0.175628837	-5.9871775	ns
LAMB1	-0.0102316	-0.02319061	-1.55438868	0.12011272	0.175804081	-5.99102956	ns
ARL2BP	-0.01382538	0.02596605	-1.5531186	0.120415855	0.176159467	-5.99205102	ns
MORF4L2	-0.01307497	0.058965624	-1.55125321	0.120862079	0.176723721	-5.99318639	ns
CD34	0.008612	0.007987352	1.549109359	0.121375887	0.177364918	-6.01255759	ns
STEAP4	0.02474968	0.136177115	1.54891942	0.121422079	0.177364918	-5.99996727	ns
NDUFA5	-0.01189873	-0.00320435	-1.5482103	0.121592759	0.177525428	-5.99765605	ns
TYRO3	-0.01022354	-0.01199441	-1.54303155	0.122843792	0.179262305	-6.01581516	ns
PLAU	0.010109339	-0.0023092	1.541110236	0.123310547	0.179853544	-6.02133695	ns
IDS	0.00316209	-0.00462316	1.540785291	0.123389422	0.179878738	-6.02652759	ns
LRRN1	0.014211258	0.029754759	1.537913882	0.124090617	0.180786446	-6.0171204	ns
SCARF2	0.009925502	-0.01020922	1.537726523	0.124135898	0.180786446	-6.0311756	ns
STOML2	-0.03159442	0.116380286	-1.53662055	0.124407317	0.181091408	-6.0156502	ns
GRIK2	-0.01358082	0.066927855	-1.53562393	0.124651021	0.181355746	-6.0343592	ns
GABARAP	-0.01584485	0.031043897	-1.5324929	0.125421322	0.182385587	-6.0372158	ns
CNGB3	-0.0185717	0.079514158	-1.53008814	0.126015892	0.183158986	-6.0287948	ns
MAP1LC3A	0.010416512	-0.01068705	1.529269534	0.126218616	0.183301079	-6.03003995	ns
AKAP12	0.006406351	0.01417554	1.52918609	0.126239202	0.183301079	-6.0305423	ns
NLGN2	0.016248752	0.090306197	1.526883315	0.126811004	0.184039827	-6.03366575	ns
MDGA1	0.025016885	-0.03926383	1.525007922	0.127277831	0.184625567	-6.04226893	ns
IL33	-0.01370185	-0.01659228	-1.52379116	0.127581822	0.184974638	-6.03861854	ns
LAMP1	0.009269799	0.021496303	1.519786083	0.128585773	0.186337695	-6.04351976	ns
APPL2	-0.03758275	-0.06227341	-1.51801077	0.129032555	0.186892391	-6.04747323	ns
EPHX2	-0.02148098	0.095137112	-1.51414345	0.130010438	0.188215409	-6.05270276	ns
PHYKPL	-0.01380201	-0.02113701	-1.51237298	0.130459869	0.188772457	-6.05596753	ns
CRADD	-0.02713127	-0.01467828	-1.51135955	0.130717611	0.189027076	-6.06170949	ns
LILRB2	-0.01222948	-0.05750719	-1.5111713	0.130765306	0.189027076	-6.06757176	ns
NCS1	-0.00968996	-0.0294056	-1.51049504	0.130937513	0.189182354	-6.07317816	ns
FGFBP2	0.016391314	0.026673303	1.509451587	0.131203824	0.189473375	-6.07264252	ns
NTproBNP	0.03365459	0.013750353	1.508853114	0.131356961	0.189600755	-6.06767989	ns

CADPS	-0.01411516	0.059040309	-1.504259	0.132535585	0.191207465	-6.06727001	ns
PSMA1	-0.01672558	0.049360822	-1.50370346	0.132678732	0.191319455	-6.06759319	ns
REXO2	0.016489994	0.098420301	1.501625373	0.133214891	0.19199777	-6.07025623	ns
HRG	-0.01042684	-0.0149451	-1.50101631	0.133371859	0.19212917	-6.08211384	ns
KLKB1	-0.00819875	0.002368563	-1.500441	0.133521053	0.192249248	-6.07585136	ns
TPPP2	0.025921437	0.081118914	1.499284633	0.133820484	0.192585418	-6.0802332	ns
SLC44A4	-0.02239332	0.144008815	-1.49738558	0.134313838	0.1932002	-6.0775277	ns
RANBP1	-0.01744685	0.010970865	-1.49394916	0.135209832	0.19439326	-6.08263048	ns
HSBP1	-0.03120183	0.031978279	-1.49333254	0.135371086	0.194529316	-6.08354631	ns
GMPR	-0.01759644	-0.0478651	-1.49243773	0.135604741	0.194769229	-6.09945099	ns
OGA	-0.02308383	0.01674354	-1.48950801	0.136374041	0.195777876	-6.1017352	ns
S100A16	-0.02167726	0.050048385	-1.48612207	0.13726779	0.196964101	-6.09397529	ns
LILRA3	0.04499207	-0.39521281	1.483007489	0.138092774	0.198050541	-6.11199209	ns
XIAP	-0.01888648	0.048017503	-1.4820594	0.138345449	0.198315518	-6.09860132	ns
CD14	-0.01401109	-0.0185986	-1.48133681	0.138537984	0.198494069	-6.09476427	ns
LYPLA2	-0.03469095	0.294517046	-1.47794519	0.139443336	0.199693252	-6.10677749	ns
SPP1	-0.01530074	-0.01950902	-1.47448145	0.140372341	0.200925115	-6.1242445	ns
HS3ST3B1	0.012866545	-0.00308259	1.472962148	0.140781816	0.201412495	-6.12058522	ns
BTNL10	0.012635929	0.049262722	1.472354318	0.14094617	0.201539328	-6.11179052	ns
IL5RA	-0.0191315	-0.01403151	-1.47212176	0.141008509	0.201539328	-6.12434654	ns
STAU1	-0.02201215	0.141479601	-1.47045943	0.141458307	0.202083296	-6.1145629	ns
GZMH	-0.02494766	-0.03104039	-1.46935345	0.141757825	0.202412151	-6.11914228	ns
LARP1	-0.02050362	0.079535597	-1.46658954	0.1425081	0.203351694	-6.13549821	ns
PTPRR	-0.01157131	0.016323987	-1.46641662	0.142555109	0.203351694	-6.13672461	ns
ICAM3	-0.00871596	-0.01010071	-1.46412118	0.143181106	0.204119893	-6.14047051	ns
BEX3	0.015032913	0.045650722	1.463932022	0.143233446	0.204119893	-6.12408574	ns
SATB1	-0.00971454	0.036292245	-1.46248625	0.143628253	0.204582683	-6.14339721	ns
TARM1	0.024754581	0.174656274	1.458595786	0.144697172	0.206004751	-6.13453614	ns
ATP6V1G2	-0.03065341	-0.00683579	-1.45349351	0.146107657	0.207845689	-6.14097306	ns
ANGPTL7	0.012092102	-0.01204065	1.453401235	0.146132603	0.207845689	-6.15732662	ns
DTX2	0.020556643	0.139973222	1.452411735	0.146407912	0.208135883	-6.14348646	ns
ZP3	0.083192342	-1.06320738	1.451235014	0.146735234	0.2084997	-6.14517719	ns
AFP	0.023060488	0.046349366	1.450494181	0.146941377	0.208610053	-6.15082208	ns
SEZ6L2	0.007887263	0.005849029	1.450442085	0.14695578	0.208610053	-6.15332036	ns
PTH1R	-0.01293403	0.036501877	-1.44493452	0.148496701	0.210695027	-6.16378122	ns
SAFB2	-0.00881521	0.00595971	-1.43909105	0.150144889	0.212930101	-6.17715345	ns
IVD	-0.01654652	-0.00277769	-1.43836263	0.150351959	0.213120252	-6.16410699	ns
CST6	-0.01590416	-0.00076379	-1.43747742	0.150603381	0.213373058	-6.15876928	ns
IGF1R	-0.00704018	-0.00848575	-1.43691877	0.150761526	0.213408822	-6.17273554	ns
ADAMTS1	-0.00976027	-0.00245939	-1.43687334	0.150774794	0.213408822	-6.16417686	ns
EFNB2	-0.01821114	0.027226755	-1.43453178	0.151441254	0.214248286	-6.16915064	ns
DEFB116	-0.02353124	0.196574092	-1.43367989	0.151684369	0.214488309	-6.17085757	ns
CA12	0.010306817	0.010701771	1.432230273	0.152098362	0.214969611	-6.17942429	ns
CACNA1H	0.017979934	0.101908115	1.426446643	0.153760226	0.217213285	-6.17970049	ns
OGT	-0.02013217	0.108604218	-1.42328419	0.154674222	0.218398805	-6.190674	ns
REST	0.011362791	0.023897697	1.422176236	0.154995856	0.218747173	-6.18400117	ns
TBR1	0.015038414	0.082587646	1.421460658	0.155203514	0.218934426	-6.18771134	ns
CAT	-0.01727237	0.057282782	-1.42085519	0.155379325	0.219076595	-6.19346065	ns
SEPTIN3	-0.02315728	-0.00302157	-1.41877535	0.155984598	0.219823854	-6.20602491	ns
CEACAM3	-0.01537344	0.01329305	-1.41588114	0.156831011	0.220910059	-6.19507398	ns
SERPINB9	-0.01115876	-0.06070617	-1.41509688	0.157060485	0.221126623	-6.20294659	ns
FMR1	-0.01114719	-0.00868669	-1.41050493	0.158411321	0.222920992	-6.20262892	ns
MILR1	-0.01670967	0.004733664	-1.40982649	0.158611509	0.223095186	-6.20484425	ns
MMP15	0.014634758	0.08078203	1.408791594	0.158916876	0.223417081	-6.2191612	ns
SLC13A1	-0.01925761	0.119842829	-1.40820141	0.159091559	0.223555031	-6.2189654	ns
RARRES1	-0.01098156	0.017547976	-1.40703234	0.159438461	0.223934731	-6.20749351	ns
IL2RB	-0.01226124	0.031361709	-1.40650362	0.159595202	0.224047111	-6.20949719	ns
BCL7B	-0.01434397	0.057095201	-1.40607328	0.159722475	0.224118034	-6.22194358	ns
CRX	-0.03598933	0.339559902	-1.40438953	0.160223502	0.224713077	-6.21196297	ns
PRSS27	-0.01308021	0.003530051	-1.40401044	0.160336113	0.224763058	-6.21729783	ns
IKZF2	-0.03062976	0.245988819	-1.40279134	0.160699708	0.225164657	-6.21341826	ns
DCC	-0.00690917	0.018421443	-1.39806153	0.162115106	0.227038901	-6.22051338	ns
TSPAN8	-0.03001624	-0.01467177	-1.39484474	0.1630827	0.228284508	-6.2375084	ns
AIF1L	-0.01764377	0.121793856	-1.3921123	0.163908924	0.229331125	-6.22875843	ns
TSPYL1	-0.01221078	0.030297154	-1.39025554	0.164471874	0.229970874	-6.229703	ns
DOC2B	-0.01067444	0.019526688	-1.39008452	0.164523684	0.229970874	-6.23157153	ns

ADAMTSL4	0.007087762	0.001425792	1.386686075	0.165558009	0.231305926	-6.23625534	ns
AMFR	-0.02116585	0.073026188	-1.38554794	0.165905492	0.231667181	-6.23826074	ns
CDC123	0.01978679	0.071682469	1.385229987	0.166002721	0.231667181	-6.23557012	ns
CASP8	-0.0201557	0.032287333	-1.38505833	0.166054593	0.231667181	-6.25243187	ns
DCDC2C	-0.01888751	0.044513062	-1.38229085	0.166902963	0.232739566	-6.23961425	ns
SAT1	0.015078124	0.094941549	1.381731295	0.167074755	0.232867917	-6.24213181	ns
GNAS	-0.02632429	0.228743839	-1.37891874	0.167939677	0.233961764	-6.26147445	ns
RAD23B	-0.01450117	0.020030052	-1.37459746	0.169276752	0.235712025	-6.25235111	ns
RAB33A	-0.02026798	0.04945868	-1.37361934	0.169580373	0.236021497	-6.25324467	ns
GFRA2	-0.0065766	-0.0100504	-1.37335976	0.169660658	0.236021497	-6.26155064	ns
UGDH	-0.02998177	0.276952341	-1.37291491	0.169799282	0.236023253	-6.25420661	ns
GFRAL	0.018355866	0.029409398	1.3728368	0.16982358	0.236023253	-6.25370679	ns
CNTNAP2	-0.01307436	-0.00203018	-1.37038906	0.170585532	0.236969435	-6.2681383	ns
APOE	0.021373864	0.064077749	1.368173746	0.171277537	0.237672319	-6.27528707	ns
RALB	0.009018369	-0.01002296	1.368020393	0.171326137	0.237672319	-6.26019582	ns
F12	-0.01243603	-0.05682967	-1.36798792	0.171335696	0.237672319	-6.27553991	ns
CLIC5	-0.00918307	0.025628319	-1.3637433	0.17266868	0.239407667	-6.26763778	ns
UROD	-0.02089717	0.00481737	-1.36329189	0.172810801	0.239486457	-6.26911899	ns
CHEK2	-0.01032551	-0.00418134	-1.36304234	0.172889538	0.239486457	-6.26885506	ns
HEPH	0.007968168	-0.01941702	1.362285536	0.1731276	0.239630624	-6.28324558	ns
SESTD1	-0.02046969	0.061696307	-1.36197787	0.17322523	0.239630624	-6.27045906	ns
GLYR1	-0.01799179	0.120905003	-1.36193007	0.173239811	0.239630624	-6.28270386	ns
FCER1A	-0.00696554	-0.01828823	-1.35923873	0.174090698	0.240693578	-6.28827128	ns
ESPL1	0.016753962	0.103070526	1.356604969	0.174927147	0.241710883	-6.27459149	ns
CDKN2D	-0.03014954	-0.14583199	-1.35637052	0.175001324	0.241710883	-6.28461054	ns
GJA8	-0.01784698	0.059133844	-1.35614055	0.175074835	0.241710883	-6.27521786	ns
FUT3_FUT5	-0.01423434	-0.02595265	-1.35412389	0.175716586	0.242482245	-6.29520251	ns
ABHD14B	-0.01800596	0.007687654	-1.35172242	0.176484199	0.243347843	-6.28896605	ns
MBL2	-0.03165026	-0.09040089	-1.35164014	0.176510524	0.243347843	-6.28946731	ns
EBI3_IL27	0.009537292	0.003729888	1.349428417	0.177219109	0.244209437	-6.30141278	ns
CARHSP1	-0.01645798	0.032933344	-1.34894129	0.177376096	0.244310472	-6.2880307	ns
ANKRD54	-0.01900549	0.086611443	-1.3455115	0.17848039	0.245715577	-6.2916877	ns
PTX3	0.015645407	-0.07781515	1.344596435	0.178775471	0.246005831	-6.30295916	ns
CD226	-0.01212378	0.018159776	-1.3428696	0.179334474	0.246658814	-6.29409471	ns
SDK2	-0.01109302	0.019304664	-1.34044418	0.180121088	0.247624095	-6.29983337	ns
PFDN6	0.019282575	0.110482804	1.340155239	0.180215025	0.247636646	-6.29934899	ns
CNPY2	-0.01827663	0.093270407	-1.33940707	0.180458393	0.247854425	-6.3006128	ns
BTLA	-0.02497837	0.116180113	-1.3379251	0.180941105	0.248378097	-6.29961648	ns
PIK3AP1	-0.01936686	0.0190266	-1.33760884	0.181043806	0.248378097	-6.31227876	ns
RAB2B	-0.01787287	0.019342448	-1.33745193	0.181094852	0.248378097	-6.31556138	ns
PRDX2	-0.01867202	0.061656396	-1.33476073	0.181974657	0.249467604	-6.31077212	ns
KYAT1	-0.01758066	0.009767978	-1.33286061	0.182597425	0.250097565	-6.31540861	ns
SDC4	-0.01429108	-0.0308522	-1.33283595	0.182605482	0.250097565	-6.31638917	ns
DNAJB8	-0.01748984	0.109921576	-1.33214122	0.182833937	0.25029306	-6.31026402	ns
RABEPK	-0.01857868	0.093189341	-1.32931216	0.183765166	0.251449993	-6.31323146	ns
RPA2	-0.00897688	0.022340899	-1.32816871	0.184142538	0.251848342	-6.31361773	ns
KLK15	0.014804029	0.013451287	1.327467945	0.184373585	0.252046286	-6.3279537	ns
IL25	-0.00992269	-0.01733542	-1.32687835	0.184569171	0.252195591	-6.31221012	ns
ENSA	-0.01495216	0.040915338	-1.32660852	0.184657823	0.252198711	-6.32992629	ns
SH3GLB2	-0.0198257	-0.01082004	-1.32582385	0.184917591	0.252435421	-6.33193759	ns
EPPK1	-0.02094846	0.027879247	-1.32171017	0.186284733	0.254182907	-6.3237526	ns
KIRREL1	-0.01687476	0.123520414	-1.32024445	0.186773458	0.254730732	-6.32617898	ns
PGA4	-0.01823277	0.005538634	-1.31891042	0.187218574	0.255218597	-6.33991392	ns
CRH	0.027173757	0.086425125	1.313547716	0.189018539	0.257552093	-6.33293607	ns
OMP	-0.01394174	0.061522709	-1.31312793	0.189159827	0.257624392	-6.33549682	ns
ADCYAP1R1	0.010464533	0.027351203	1.312611343	0.189333972	0.257741351	-6.335169	ns
CXCL12	0.011967572	0.002110372	1.302588189	0.192735249	0.262211526	-6.34946798	ns
CTSE	0.014703601	0.016807315	1.302406371	0.19279731	0.262211526	-6.34981667	ns
CHAC2	-0.01816975	0.03139876	-1.30128603	0.193180498	0.26261036	-6.34988771	ns
CD1C	-0.00712142	-0.00175217	-1.3006637	0.193393009	0.262776913	-6.36261053	ns
BABAM1	-0.00816114	0.022090663	-1.30029015	0.193521473	0.262829164	-6.35005087	ns
USP47	-0.02472444	0.166035214	-1.298927	0.193988906	0.263288823	-6.35249408	ns
COL3A1	0.006432392	-0.00211634	1.298775705	0.194040256	0.263288823	-6.36683883	ns
PARD3	-0.02092693	0.088625529	-1.29648083	0.194829476	0.264236912	-6.36215614	ns
RAD51	0.015302003	0.045274108	1.294614236	0.195472918	0.2649865	-6.36456103	ns
GAD2	-0.01294693	0.056749684	-1.29375561	0.195769724	0.265265705	-6.35847876	ns

MYBPC1	-0.01933439	0.055926679	-1.29121824	0.196647284	0.266331201	-6.37702079	ns
ACADM	-0.01970478	0.12953047	-1.28941229	0.197274324	0.267056572	-6.37124485	ns
CPA2	-0.01944535	0.031789948	-1.28903517	0.197405173	0.267096911	-6.37843289	ns
FGF5	0.009974991	-0.00604983	1.288800854	0.197487065	0.267096911	-6.36723241	ns
LPP	-0.01885054	0.01550582	-1.28809993	0.197731006	0.267303026	-6.36642058	ns
CCL28	0.0172568	0.041427269	1.287079424	0.198086092	0.267659134	-6.37596078	ns
FUT8	-0.01759096	0.050723072	-1.28673679	0.198205552	0.267696676	-6.37539118	ns
AMIGO2	0.006690376	-0.01109965	1.284461603	0.19899994	0.268545859	-6.3790363	ns
GSTP1	-0.02014704	0.065066671	-1.28441031	0.199018233	0.268545859	-6.37130441	ns
SCRN1	-0.0167792	0.043232895	-1.28261877	0.199645044	0.269267219	-6.38392663	ns
ARID4B	-0.01256753	0.039316032	-1.28098856	0.2002174	0.2699145	-6.37688658	ns
ENPP2	-0.00786153	0.021937929	-1.28049355	0.200390759	0.270023543	-6.39109066	ns
DENR	0.016078624	0.057299741	1.28003512	0.200552534	0.270116881	-6.37603656	ns
PPM1B	-0.01766939	0.110689739	-1.27930301	0.200810117	0.270339115	-6.37696817	ns
CGB3_CGB5_C	-0.0118647	-0.00984943	-1.27763048	0.201398909	0.270911474	-6.39321418	ns
PTPRM	-0.00720912	-0.00924396	-1.27756893	0.201420825	0.270911474	-6.38940281	ns
SPARC	-0.02120873	-0.10878091	-1.27521077	0.202253842	0.271906638	-6.3952132	ns
MRI1	-0.0189787	-0.07440542	-1.27338911	0.202899583	0.272649233	-6.38610904	ns
TGFBR1	-0.0060833	0.001379211	-1.26873541	0.204555089	0.274638762	-6.39035599	ns
C1QTNF6	-0.00865403	0.015246609	-1.26869678	0.204568256	0.274638762	-6.40659405	ns
CLEC3B	-0.00594648	0.000759801	-1.26669602	0.205283541	0.275472399	-6.39612396	ns
CCDC134	-0.01320374	0.025800602	-1.26567356	0.205649063	0.275836134	-6.40847601	ns
LHB	-0.02581112	0.17978426	-1.26124733	0.207239311	0.277841501	-6.40090091	ns
OSBPL2	-0.01055502	0.06493277	-1.25948223	0.20787517	0.278566083	-6.41722773	ns
ITM2A	-0.01742008	-0.00060033	-1.2562891	0.209030298	0.279983291	-6.41716773	ns
BRD3	-0.01320086	0.040608058	-1.2560303	0.209124506	0.279983291	-6.40698683	ns
FES	0.01392868	0.018532069	1.255263845	0.209402495	0.280216543	-6.40838756	ns
GRK5	-0.01513383	0.073062347	-1.25502086	0.209490655	0.280216543	-6.4094679	ns
AKT3	-0.01680207	0.020927993	-1.25466543	0.209619844	0.280260964	-6.40643462	ns
NFIC	-0.01196052	0.05872239	-1.24953594	0.211487957	0.282629214	-6.43058891	ns
GAMT	0.009965759	0.048941785	1.249131085	0.211636583	0.282698455	-6.41558029	ns
MUCL3	0.012296148	0.075296659	1.248086662	0.212018774	0.283079478	-6.415125	ns
BPIFB1	-0.01891704	-0.01802848	-1.24528084	0.213047664	0.284323207	-6.42649129	ns
TPRKB	0.017382928	0.082619474	1.243250675	0.213794876	0.285190058	-6.42111522	ns
ARHGAP1	0.013543023	-0.00398809	1.241645009	0.214386927	0.285849237	-6.42529501	ns
KIR3DL2	-0.02323184	0.221900985	-1.24041728	0.214840465	0.286323213	-6.42461408	ns
ZNF830	0.021928916	0.29561881	1.239636999	0.215128518	0.28657631	-6.44091328	ns
SLAMF7	-0.01698045	-0.04434031	-1.23919949	0.215290582	0.286582609	-6.43837439	ns
PXDNL	0.015662256	0.078730815	1.239093935	0.215329535	0.286582609	-6.44158267	ns
MED21	-0.01398338	0.072308115	-1.23768147	0.215853523	0.287149106	-6.42973717	ns
LRFN2	0.012899153	0.057596577	1.234053026	0.217202333	0.288811845	-6.43515007	ns
ART5	-0.01673166	0.126352444	-1.23333695	0.217469303	0.289035214	-6.43652209	ns
SCARB1	-0.01684699	0.101875395	-1.23266204	0.217721111	0.289238237	-6.4372856	ns
KAZALD1	-0.0130009	-0.03563132	-1.23120532	0.218265011	0.289828936	-6.44565491	ns
DLGAP5	0.014079631	0.113455707	1.227801442	0.219540131	0.291389629	-6.45736481	ns
MTHFD2	0.006841054	0.013441766	1.226549485	0.220010597	0.291881391	-6.45794128	ns
NGFR	-0.00757245	0.000947915	-1.22508155	0.220563125	0.292481528	-6.45977847	ns
S100A3	-0.01162409	0.060646191	-1.22021564	0.222401763	0.294785814	-6.46467015	ns
GP1BB	-0.01804703	0.0065882	-1.2171253	0.223575652	0.296207306	-6.4548268	ns
TREML1	-0.01394058	0.037420251	-1.21477665	0.224469817	0.297257082	-6.47111634	ns
PPME1	-0.01738948	-0.01763579	-1.20323424	0.228904309	0.302992104	-6.47951458	ns
CXCL14	-0.01751168	0.046778147	-1.20276845	0.229084788	0.303093603	-6.4738127	ns
PDGFRA	-0.00784055	-0.00175355	-1.20230513	0.229263925	0.303193234	-6.48093521	ns
PITHD1	0.021175885	0.174158417	1.198939621	0.230570381	0.304782939	-6.47350641	ns
CD200	0.007332659	0.016014069	1.198625799	0.230691993	0.304805711	-6.48755951	ns
ICOSLG	0.005327119	0.003342288	1.19556879	0.231883409	0.306241318	-6.48865798	ns
BRSK2	-0.01014804	0.068094003	-1.19469843	0.232223131	0.30655133	-6.49724364	ns
PDIA5	-0.01571253	0.027333797	-1.19420296	0.23241744	0.306669193	-6.48055565	ns
TNFSF13	-0.00721691	-0.01283804	-1.19385864	0.232551641	0.306707675	-6.49440245	ns
SCGB3A1	-0.00666424	0.007533294	-1.19318488	0.232815665	0.306917265	-6.48534304	ns
CD33	-0.02234044	-0.21778005	-1.18861033	0.234611325	0.309087962	-6.50131528	ns
UPK3A	0.013137316	0.034600832	1.188452308	0.234673976	0.309087962	-6.49056978	ns
NAPRT	-0.00866032	0.036937627	-1.18727585	0.235137023	0.309558209	-6.50508391	ns
CPB1	0.015751742	0.031093953	1.182545031	0.237007635	0.311880259	-6.51106162	ns
SUSD2	0.007834517	0.000705283	1.181286712	0.237507123	0.312396756	-6.50843676	ns
IL10RA	-0.01598322	0.098333949	-1.18093522	0.237647053	0.31244007	-6.49968982	ns

MAPKAPK2	-0.01905587	0.020175084	-1.18028307	0.237906038	0.312639798	-6.50525896	ns
NTF3	0.009711216	0.011981507	1.178827181	0.238485278	0.313260014	-6.51096595	ns
CHRM1	0.009586388	0.020869204	1.177820621	0.23888667	0.313646167	-6.50258459	ns
CBLN1	-0.02458845	0.206291956	-1.17602808	0.239602221	0.314444263	-6.50517641	ns
HDAC8	-0.01613072	0.127582516	-1.17201301	0.241210405	0.31641257	-6.50773272	ns
DEFB103A_DE	-0.01058369	0.035581048	-1.17115359	0.241555768	0.316723325	-6.504195	ns
LSM8	-0.02222578	0.284035071	-1.17063369	0.24176432	0.316854495	-6.5165389	ns
ARG2	-0.0108878	0.049651298	-1.17022179	0.241929776	0.316926721	-6.52409895	ns
CGN	-0.01015103	0.032727112	-1.16995754	0.242036503	0.316926721	-6.51176354	ns
CEACAM20	-0.01130285	0.044162581	-1.1696851	0.242145977	0.316927949	-6.51764279	ns
GSAP	-0.02412161	0.246874791	-1.16684351	0.243292069	0.318285323	-6.51487138	ns
TSNAX	-0.0101854	0.028248271	-1.16263843	0.244994689	0.32036923	-6.51929761	ns
TLR2	-0.00588854	-0.00580661	-1.16079951	0.245741594	0.321202083	-6.52793962	ns
MYH7B	0.010953801	0.05545574	1.156478408	0.247503916	0.323222973	-6.52464642	ns
ACP1	0.011337414	0.062168837	1.156248836	0.247597761	0.323222973	-6.52598171	ns
GZMB	0.017144335	0.032553069	1.156194056	0.247619791	0.323222973	-6.53724595	ns
ACRBP	-0.00962369	0.029913228	-1.1543987	0.248354601	0.324037281	-6.54239041	ns
NAP1L4	-0.01036463	-0.02855715	-1.15311346	0.248881685	0.324579955	-6.54248946	ns
MFAP3	-0.01304153	0.035822233	-1.14890469	0.250613524	0.32669263	-6.53630464	ns
ECE1	0.009380201	-0.01220863	1.146813525	0.251476908	0.32767183	-6.5388539	ns
MORN4	-0.0197152	0.11814595	-1.1460101	0.251809225	0.327958491	-6.53663051	ns
GKN1	0.007281565	-0.02270903	1.142758122	0.253157248	0.32956717	-6.54198296	ns
CTAG1A_CTAG	-0.01257083	0.01151326	-1.13897711	0.254730618	0.331329817	-6.55148119	ns
RAB39B	-0.02278161	0.211991087	-1.13895958	0.254738164	0.331329817	-6.54639517	ns
HNF1A	-0.01817086	0.140364752	-1.13769992	0.255263839	0.331865722	-6.54877602	ns
SCN2A	-0.01167443	0.04412435	-1.13502521	0.2563828	0.33317213	-6.55229271	ns
EDNRB	-0.01131552	0.062912895	-1.13440597	0.256642383	0.333361103	-6.55085919	ns
NCR3LG1	-0.00780645	-0.00379248	-1.1335224	0.257012513	0.333693436	-6.56606792	ns
DDX25	0.009269984	0.03802396	1.13187959	0.257703017	0.334441248	-6.55534411	ns
ACAN	0.007524404	-0.00876062	1.130771331	0.258169193	0.334897398	-6.56059343	ns
CENPJ	-0.0175018	0.10837507	-1.13037592	0.258335613	0.334964472	-6.56260061	ns
EFCAB2	-0.00860408	0.026918773	-1.12613567	0.260125797	0.337135964	-6.56735836	ns
ARHGEF5	-0.01834602	0.062920942	-1.12472792	0.260722252	0.337759084	-6.56386269	ns
TAP1	-0.01450868	0.077547711	-1.11829575	0.263458298	0.341152208	-6.57610804	ns
DNAJA4	-0.0177522	0.047081841	-1.1154527	0.264673771	0.342574207	-6.58733048	ns
SCG3	0.008775133	0.010986714	1.112603244	0.265896283	0.344004053	-6.58547811	ns
FZD10	-0.01436289	0.078663035	-1.11039134	0.266848091	0.345082562	-6.5793013	ns
ITIH5	-0.01108166	0.028145376	-1.10982116	0.267093371	0.345246854	-6.59258326	ns
GBP2	-0.01534479	-0.01985941	-1.10841813	0.267698927	0.34587649	-6.58223697	ns
CPA1	0.015144082	0.018610704	1.107540295	0.268077575	0.346210408	-6.59648038	ns
FAM171A2	0.038340779	0.39026599	1.107269693	0.268194501	0.346210408	-6.59732855	ns
CDH6	0.009612255	0.002957018	1.106821993	0.26838829	0.34630747	-6.59057158	ns
MDM1	-0.00761939	0.006545231	-1.10535084	0.269025189	0.34697595	-6.58485446	ns
FBN2	-0.01434498	-0.03299428	-1.10421662	0.269516761	0.347456486	-6.58610057	ns
HBQ1	0.016186967	0.055969472	1.103844264	0.269677814	0.347510687	-6.60185506	ns
CBLN4	-0.00791229	-0.00472011	-1.09779503	0.272311033	0.350749103	-6.60847826	ns
SH3GL3	-0.02064703	-0.01478156	-1.09295763	0.27442976	0.353322266	-6.60053391	ns
TPPP3	-0.01007973	0.026668434	-1.09204065	0.274832694	0.353685089	-6.59982728	ns
VCPKMT	-0.0208076	0.200195614	-1.08833116	0.276466398	0.355512721	-6.60070726	ns
ASPN	0.006302391	0.007381007	1.088262063	0.276496367	0.355512721	-6.61717619	ns
C9orf40	-0.01969143	0.072596166	-1.08772761	0.276732331	0.355525784	-6.61665291	ns
MLLT1	0.016186629	0.080641185	1.087688633	0.276750037	0.355525784	-6.60140243	ns
VPS28	-0.0159228	0.065348707	-1.08574938	0.277607029	0.356324273	-6.61177762	ns
OPTC	0.01155405	0.035810386	1.085729805	0.277615658	0.356324273	-6.61325801	ns
IL31	0.013118701	0.107510903	1.080090173	0.280119353	0.359379837	-6.61066462	ns
CCN2	-0.01473474	-0.01454191	-1.07775893	0.281158325	0.360554373	-6.62247918	ns
MFAP4	0.007323059	0.016450145	1.076627445	0.281663887	0.36104414	-6.61688975	ns
GHRHR	-0.01383964	0.085430385	-1.07541946	0.282204191	0.361440444	-6.61774012	ns
AGT	0.005219762	0.029067909	1.074945223	0.282416008	0.361440444	-6.63033467	ns
ITGA2	0.007889101	0.018631455	1.07489949	0.282436854	0.361440444	-6.6187383	ns
TRIM21	-0.0192881	0.002997749	-1.07482903	0.282468183	0.361440444	-6.62751425	ns
PSMD5	0.012971312	0.079579642	1.073680311	0.282983129	0.361940751	-6.61916816	ns
CD300LG	0.009064191	0.019753201	1.072411099	0.283552263	0.362509899	-6.63045385	ns
MAGEA3	0.019588325	-0.08175182	1.071950307	0.283759297	0.362615819	-6.62658293	ns
ITPA	-0.01572079	0.00633964	-1.07126692	0.284066628	0.362746285	-6.62078634	ns
CD209	0.009783259	0.0383518	1.071169894	0.284109847	0.362746285	-6.63151067	ns

TBC1D17	-0.01107597	0.041676135	-1.06655489	0.286189968	0.365242442	-6.62718125	ns
RBM17	-0.01755021	0.027297952	-1.06442316	0.287153603	0.366312153	-6.64360215	ns
LAMA1	0.012433343	0.125759385	1.061656228	0.288408833	0.367752748	-6.63097872	ns
ERVV-1	0.010535467	0.046614404	1.05994412	0.289187052	0.368584109	-6.63278481	ns
BLMH	-0.00774258	-0.00352135	-1.05788093	0.290126204	0.369619772	-6.64995655	ns
FABP9	-0.01378868	0.02416113	-1.05039009	0.293555344	0.373825383	-6.65334177	ns
THBS2	-0.00844922	0.012417318	-1.04987372	0.293792682	0.37396453	-6.65423898	ns
SAT2	-0.01586433	0.05538566	-1.04876375	0.294303468	0.374451471	-6.6510339	ns
CD109	0.008132368	-0.00691789	1.045887638	0.295629361	0.375974623	-6.65839318	ns
IL13RA2	0.006470988	-0.01318803	1.044554346	0.296245735	0.376552003	-6.65033931	ns
IL5	0.03549703	0.307193057	1.044347689	0.296341268	0.376552003	-6.65081859	ns
NMRK2	0.009619512	0.067304802	1.041979502	0.29743795	0.377781128	-6.64980316	ns
TIE1	0.010163756	-0.07811984	1.040091893	0.298313421	0.378728344	-6.66851626	ns
CSF2	-0.00824447	0.012711188	-1.03724174	0.299639892	0.38024706	-6.6564579	ns
SOX2	0.005247632	-0.00078117	1.036014815	0.300211406	0.380806822	-6.67198797	ns
EXOSC10	-0.01335055	0.148431382	-1.02813652	0.303902156	0.385313203	-6.66580846	ns
MRPL24	-0.00864536	0.013004816	-1.02786909	0.304027952	0.385313203	-6.66608186	ns
CENPF	-0.01274083	0.062890825	-1.0233189	0.306173306	0.387863797	-6.67725108	ns
ARFIP1	-0.01542355	0.050202676	-1.02002344	0.30773324	0.389670885	-6.68962705	ns
TADA3	-0.00881547	0.008539283	-1.01695318	0.309191627	0.391236709	-6.690803	ns
RNF5	-0.01612547	-0.09088716	-1.01685699	0.309237782	0.391236709	-6.6782258	ns
CHGB	-0.00948238	-0.00350925	-1.01475933	0.31023646	0.392330214	-6.69314523	ns
TSPAN7	-0.0215831	0.292569993	-1.01420923	0.310499197	0.392492492	-6.68140279	ns
PTPN9	-0.00850227	0.02560713	-1.01148863	0.311798929	0.393964895	-6.68093586	ns
MORC3	0.006129625	0.020092046	1.009107314	0.312938923	0.394934771	-6.6996884	ns
ITGB5	0.008698633	0.024052368	1.009055213	0.312963972	0.394934771	-6.69754402	ns
CDA	-0.01051742	0.005324099	-1.00903786	0.312972281	0.394934771	-6.69864532	ns
PSCA	0.056442447	-1.29913264	1.006003872	0.314429449	0.396602156	-6.70174343	ns
CSNK1D	-0.01394437	0.102801037	-1.00490328	0.314959597	0.397099319	-6.68861001	ns
PRL	0.016919196	0.108852202	1.002755548	0.315994468	0.39823213	-6.70702741	ns
RGS10	-0.01302128	0.050055733	-1.00171252	0.316498074	0.398694727	-6.70803724	ns
FUCA1	-0.01941826	-0.2108768	-0.99962698	0.317506724	0.399792857	-6.70743892	ns
MYL6B	-0.02046136	0.267128984	-0.9980797	0.318256688	0.400564453	-6.69704651	ns
RANGAP1	-0.01153133	0.059555221	-0.99634144	0.319100303	0.401318006	-6.69825893	ns
TACC3	-0.02750886	-0.05320279	-0.99627917	0.319130278	0.401318006	-6.70586671	ns
ARNTL	0.011421882	0.106952634	0.995361796	0.319576354	0.401705964	-6.69878391	ns
GATA3	0.00572063	-0.00430468	0.994639314	0.319927739	0.401974612	-6.69774112	ns
IL36A	-0.01220027	0.067156102	-0.9934374	0.320512832	0.402536546	-6.70000262	ns
TG	-0.02386485	0.198544512	-0.99026324	0.322060977	0.404306987	-6.71744438	ns
PODXL	0.003487355	-0.00496073	0.989051258	0.322653706	0.40465468	-6.71300237	ns
FRMD4B	-0.01563768	0.107944685	-0.98900338	0.322677408	0.40465468	-6.70505757	ns
CEP164	-0.01431098	0.098847399	-0.98884733	0.322753681	0.40465468	-6.70692748	ns
ANGPT1	-0.0196505	-0.07270749	-0.98823664	0.323052405	0.404855374	-6.71326401	ns
HBZ	-0.02288107	0.091404729	-0.98790488	0.323214666	0.404884953	-6.72079258	ns
PRKCQ	-0.00998233	0.043364403	-0.97989512	0.327153586	0.409643426	-6.71569099	ns
SYT1	-0.00836518	0.032338692	-0.97622953	0.328965966	0.411736229	-6.73214902	ns
NGF	0.002724436	-0.00377047	0.974691851	0.329728576	0.4125139	-6.72628524	ns
TYRP1	0.013071024	-0.04533902	0.974219774	0.329963102	0.412630517	-6.71880428	ns
SERPINA9	-0.01614209	0.001594051	-0.97287152	0.330632634	0.413290793	-6.72621149	ns
AHSP	-0.01977757	-0.00631884	-0.97078742	0.331669461	0.414310354	-6.73274443	ns
BRD2	-0.00726041	0.017556094	-0.97066227	0.331732057	0.414310354	-6.7243809	ns
BCAM	0.005322511	0.002000712	0.965539664	0.334289772	0.417326265	-6.73876079	ns
C1GALT1C1	0.006807609	0.008485413	0.964454029	0.334833861	0.417826869	-6.72890242	ns
HIP1	-0.00709466	0.038352973	-0.9641599	0.334981277	0.417832263	-6.72918448	ns
CCT5	-0.0104184	0.05462942	-0.96343245	0.335346035	0.418108634	-6.73032704	ns
B3GNT7	-0.00759791	-0.0376696	-0.96170188	0.3362148	0.419012896	-6.73248701	ns
AOC1	-0.01278563	0.084091405	-0.95912295	0.337512159	0.4204503	-6.73571833	ns
UBE2B	-0.00606863	0.012489597	-0.95810573	0.338024436	0.420908893	-6.74860242	ns
IL1A	-0.012596	0.074834604	-0.95562731	0.339275849	0.422287076	-6.73904645	ns
LYPD8	0.011051795	-0.00238495	0.952155042	0.341033657	0.424294111	-6.74106855	ns
SEMA7A	-0.00589481	0.004378665	-0.95106874	0.34158436	0.424798268	-6.75445372	ns
ATP1B2	0.006440436	0.018758175	0.949097356	0.342586357	0.425715449	-6.74351374	ns
PPT1	-0.01625676	0.199648092	-0.94904389	0.342613461	0.425715449	-6.74452125	ns
DYNC1H1	0.00985399	0.047359834	0.947659417	0.343317689	0.426409039	-6.75850276	ns
GPIHBP1	-0.01445869	-0.08396748	-0.94717385	0.343564999	0.426534777	-6.75888561	ns
TREH	-0.01561048	-0.05782232	-0.94651421	0.343901148	0.426770656	-6.76015992	ns

TEX101	0.010133764	0.099581517	0.944984086	0.344682105	0.427429703	-6.74834464	ns
S100G	0.009398034	0.06627761	0.944531934	0.344912592	0.427429703	-6.76338139	ns
CTRL	0.013513674	-0.00451214	0.944109383	0.34512847	0.427429703	-6.76282352	ns
TNN	-0.00886879	0.013738185	-0.94384916	0.345261462	0.427429703	-6.76266529	ns
SIGLEC9	-0.00654854	-0.02995732	-0.94383956	0.345266353	0.427429703	-6.76425927	ns
KIAA0319	-0.00621374	0.017264363	-0.94375318	0.345310503	0.427429703	-6.76315794	ns
ALMS1	-0.00911102	0.03938938	-0.94057515	0.346937502	0.429261655	-6.75805555	ns
PPIF	-0.01265709	0.089731501	-0.93980795	0.347330741	0.429566185	-6.76645089	ns
ATP1B4	0.008663651	0.04827829	0.939473447	0.347502823	0.429597055	-6.75079229	ns
PDGFRB	-0.00767141	-0.0410986	-0.93547756	0.349556806	0.431953395	-6.77129559	ns
KRT6C	-0.00773774	0.040945078	-0.93407856	0.350278376	0.432610443	-6.75757677	ns
RAB37	-0.0183528	0.193401466	-0.93387204	0.350384828	0.432610443	-6.75948604	ns
IMPA1	-0.01327214	0.039305942	-0.93092541	0.351906867	0.434306024	-6.76775812	ns
HMGCS1	0.008867136	0.023525376	0.930454494	0.352150757	0.434423409	-6.76093661	ns
TOP1MT	-0.01012807	0.038744892	-0.92900183	0.352903062	0.435167627	-6.76052079	ns
AMDHD2	0.005174797	-0.03569156	0.927033861	0.353923404	0.436241595	-6.77831018	ns
JMJD1C	-0.01159236	0.054007275	-0.92662468	0.354136321	0.436319855	-6.76271406	ns
DCTPP1	0.006534083	-0.01273703	0.922932985	0.356056676	0.438500841	-6.77617586	ns
EXTL1	0.009738551	0.074238247	0.921550121	0.356778076	0.439204039	-6.76913633	ns
C1QBP	-0.01367351	0.112604731	-0.92089086	0.357121721	0.439441815	-6.78531309	ns
GNPDA1	-0.0065047	0.029462089	-0.92040714	0.357374765	0.43956795	-6.77113233	ns
ATP1B1	0.009859431	0.0610765	0.919201182	0.358005102	0.440157852	-6.77128624	ns
PCDHB15	-0.00789832	0.013597725	-0.91833093	0.358459975	0.440325617	-6.78571836	ns
KIAA1549	-0.0137659	-0.01014461	-0.91824479	0.358505228	0.440325617	-6.77869595	ns
DCN	0.004565497	-0.00308751	0.917891263	0.358690489	0.440325617	-6.77370859	ns
ADD1	-0.01430956	0.017132858	-0.9177875	0.35874474	0.440325617	-6.774409	ns
FOLR1	-0.00571479	0.006530722	-0.91229229	0.361629169	0.443679484	-6.78782211	ns
SLC34A3	-0.00994465	0.03523829	-0.91172525	0.361927998	0.443859619	-6.776333	ns
NUDT10	0.004776796	0.022348909	0.907802065	0.363997315	0.446209975	-6.78259089	ns
ANKMY2	-0.01597346	0.016214311	-0.90523143	0.365357297	0.447689177	-6.78327408	ns
IL6ST	-0.00366622	-0.00855254	-0.90428994	0.36585591	0.448112104	-6.79311544	ns
OFD1	-0.01288243	0.153501516	-0.90335948	0.36634939	0.448528393	-6.79217931	ns
FSHB	0.012928733	0.487550514	0.9025405	0.366784235	0.448833245	-6.78732715	ns
ART3	0.006965982	0.031598704	0.902311063	0.366905806	0.448833245	-6.79620238	ns
NFKB2	-0.01125576	0.103181734	-0.90101221	0.36759604	0.449489295	-6.79428539	ns
NME1	0.004172361	0.006782124	0.899700904	0.368293851	0.450154058	-6.78823748	ns
ADGRE2	-0.00584987	-0.00303074	-0.89639517	0.370055744	0.452118315	-6.80359955	ns
VWC2L	0.009219904	0.087433601	0.894448274	0.371095961	0.453199585	-6.80918521	ns
CD248	-0.00770537	0.007250087	-0.89375753	0.371465531	0.453461267	-6.80844152	ns
TEF	0.008379975	0.016446648	0.891789779	0.372519895	0.454558334	-6.79596949	ns
ARHGAP30	-0.00787773	0.08474333	-0.89131813	0.372772574	0.454676657	-6.80292535	ns
RBM19	-0.00920066	0.061012731	-0.89044338	0.373242108	0.455059271	-6.79716263	ns
TP53INP1	0.006820769	0.047242235	0.888984562	0.37402557	0.45582415	-6.79967953	ns
KLRC1	0.011706575	0.097034294	0.887645105	0.374745842	0.456457972	-6.80108983	ns
SLITRK6	-0.00707016	0.001927655	-0.88732276	0.374919321	0.456457972	-6.8011473	ns
ARG1	0.011026971	0.014040779	0.887145306	0.375014614	0.456457972	-6.80900793	ns
GFOD2	0.026485914	0.48734443	0.885396961	0.375956724	0.457414014	-6.80206431	ns
MAN1A2	0.00497424	-0.00399554	0.884780439	0.37628917	0.457627812	-6.80311078	ns
COL5A1	0.007315611	0.026862329	0.879882066	0.378937162	0.460501331	-6.80829149	ns
KIF20B	0.014810594	0.062821701	0.879826436	0.378967363	0.460501331	-6.80796127	ns
CD200R1	-0.00717486	0.016221367	-0.8793441	0.379228552	0.460627026	-6.81569939	ns
KCNC4	0.010827221	0.109911548	0.878263273	0.379814856	0.461147352	-6.80932777	ns
NUDT15	-0.00984163	0.061754334	-0.87682076	0.380598013	0.461906151	-6.80913329	ns
STX5	-0.007824	0.040134448	-0.87652959	0.380756202	0.461906152	-6.80870203	ns
CDH22	0.005421247	0.013496106	0.872136788	0.383147474	0.464614046	-6.81974554	ns
IGFBP7	-0.00563232	-0.01732856	-0.87105553	0.383737312	0.464849197	-6.82917274	ns
TDGF1	0.029501182	0.194851732	0.870950444	0.383795087	0.464849197	-6.81562399	ns
ZP4	0.006470908	0.038133797	0.870906768	0.383818977	0.464849197	-6.81251274	ns
CYB5A	-0.0136389	0.073296799	-0.86884705	0.384944673	0.465910667	-6.81429447	ns
SYNGAP1	0.010921276	0.080529715	0.868719255	0.385014534	0.465910667	-6.81761832	ns
SOWAHA	0.009297407	0.065157369	0.864290446	0.387442073	0.46865404	-6.82093904	ns
BCL2L11	-0.00879389	-0.07996345	-0.86089324	0.389310297	0.470718869	-6.8334484	ns
ZCCHC8	-0.0113082	0.078328402	-0.86057341	0.389486764	0.470737314	-6.82316911	ns
CLU	-0.00597265	0.027943405	-0.85989876	0.389858155	0.470991234	-6.83845718	ns
GLT8D2	-0.00920453	0.061756019	-0.8543936	0.392900526	0.474470445	-6.82982819	ns
GBP6	-0.0080997	0.046211873	-0.85217211	0.394132152	0.47576101	-6.83032389	ns

ZNF174	-0.00677311	0.051263643	-0.84803605	0.396431397	0.47833871	-6.8331352	ns
IL10	-0.01608511	0.056158783	-0.84409178	0.39863149	0.480794692	-6.8388581	ns
CA11	-0.00601618	0.047150199	-0.84064971	0.400557562	0.482918283	-6.84046844	ns
CD40LG	-0.01534815	-0.04097146	-0.83900915	0.40147709	0.483827116	-6.85704258	ns
NRXN3	-0.00655099	0.05584233	-0.83375903	0.404429961	0.487184606	-6.84671765	ns
IL4	-0.01741216	-0.15304078	-0.83178396	0.405544048	0.488325204	-6.84911437	ns
CXCL8	-0.01298452	-0.00524954	-0.82711181	0.408186603	0.491304568	-6.86029172	ns
FRMD7	0.005909736	-0.00560272	0.826388457	0.408596813	0.491441632	-6.85280196	ns
KIR2DL3	-0.01465363	0.005696045	-0.82631756	0.408637083	0.491441632	-6.85234797	ns
GPA33	0.026600489	0.084181281	0.82592301	0.408860647	0.491508064	-6.86023115	ns
PPP1R14D	0.006731096	0.021593085	0.825201079	0.409270121	0.491797841	-6.86840593	ns
TLR3	-0.01188865	-0.12203715	-0.82444712	0.409698209	0.492109737	-6.86908923	ns
TCP11	-0.0107507	0.119664768	-0.82302934	0.410504163	0.492875065	-6.86114063	ns
FGF16	0.008505062	0.023372222	0.822024161	0.411076181	0.493359001	-6.85473546	ns
CCS	0.009044594	0.028143193	0.819241695	0.412661316	0.495044068	-6.86495685	ns
MEP1B	-0.02280492	-0.20000856	-0.81883189	0.41289508	0.495044068	-6.86634155	ns
DUSP29	-0.01157638	0.098036284	-0.81866791	0.412988819	0.495044068	-6.85911724	ns
TAB2	-0.01932275	0.011923119	-0.81724782	0.413799571	0.495729152	-6.86585636	ns
TERF1	0.005056312	0.013963673	0.817072598	0.413899888	0.495729152	-6.858771	ns
NXPE4	0.006061909	0.033016579	0.814594024	0.415317637	0.497223247	-6.86146741	ns
CSNK2A1	0.007985831	0.038722309	0.813508453	0.415939079	0.49776316	-6.87793493	ns
BTN1A1	0.006985523	0.047315714	0.811759644	0.416942092	0.498759077	-6.87636606	ns
MEGF11	-0.00582398	0.039142756	-0.80691813	0.419726417	0.501884167	-6.86765624	ns
TLR1	0.004565016	0.003647543	0.805855231	0.420339072	0.502411007	-6.86782304	ns
SKIV2L	-0.0068303	0.03970892	-0.80475184	0.420975625	0.502965968	-6.86939225	ns
KCNH2	-0.01325829	0.214804303	-0.80263805	0.422196591	0.504033095	-6.87204011	ns
ANGPT2	-0.00712642	-0.00772069	-0.80260732	0.422214024	0.504033095	-6.88745874	ns
PI16	-0.0042124	0.005984555	-0.80225999	0.422414898	0.504066817	-6.88489496	ns
TEK	-0.00391614	0.00625458	-0.7971777	0.425360097	0.507373972	-6.88785894	ns
DPEP2	-0.00479832	-0.00543528	-0.79422883	0.427074549	0.509210977	-6.88576599	ns
VTA1	-0.01325559	0.019069895	-0.79032793	0.429348366	0.511713154	-6.89645408	ns
PRR4	-0.01208846	0.058512286	-0.78931654	0.429939467	0.512208586	-6.88007968	ns
SPINT3	0.025162897	0.982776912	0.785455766	0.43219843	0.51468981	-6.89822886	ns
LXN	-0.01185221	0.101115315	-0.78487924	0.432536618	0.514882562	-6.88979063	ns
ARID3A	-0.00686882	0.033694877	-0.78089351	0.434877387	0.517458016	-6.88965764	ns
TRIM40	-0.01225552	0.036227833	-0.77987454	0.435476935	0.517960347	-6.8899524	ns
ARHGAP5	-0.01283425	0.160408679	-0.77782136	0.436686583	0.519187631	-6.88882262	ns
LYZL2	0.006063298	0.034659601	0.77738009	0.436946796	0.519253543	-6.88916385	ns
SLC12A2	-0.00448462	0.01478247	-0.77712429	0.437097674	0.519253543	-6.89112303	ns
NFASC	-0.00482884	-0.0135977	-0.77608439	0.437710978	0.519619456	-6.90758471	ns
HMBS	-0.00797783	-0.02120055	-0.77599867	0.437761597	0.519619456	-6.90834858	ns
XPNPEP2	0.017624771	-0.08891704	0.770964681	0.440739667	0.522941823	-6.90830091	ns
MYOM2	0.008308503	0.048417307	0.769154317	0.441813791	0.524003359	-6.89725084	ns
TPBGL	-0.00699635	0.053055573	-0.76656692	0.443351022	0.525613067	-6.90018533	ns
DCLRE1C	0.005317311	0.012530805	0.764726947	0.444446168	0.526697569	-6.89886595	ns
ISLR2	0.006625778	-0.00849424	0.753991019	0.450866447	0.533953965	-6.90779372	ns
SULT1A1	-0.0229708	-0.14694227	-0.75371766	0.451030218	0.533953965	-6.92456654	ns
EP300	-0.00487522	0.001158383	-0.75357218	0.451117956	0.533953965	-6.90836303	ns
STX1B	-0.00702295	0.03973848	-0.75300198	0.451460453	0.534142837	-6.91043487	ns
CEP350	0.006186176	0.013706483	0.751185181	0.452552984	0.535218596	-6.90907305	ns
NAGPA	-0.00347981	-0.00723809	-0.75074791	0.45281605	0.535312901	-6.91299579	ns
ISM2	0.0069416	0.006836245	0.747644959	0.454685693	0.537305635	-6.92771106	ns
TRPV3	0.008090063	0.049848417	0.744946859	0.456315492	0.539013446	-6.91547578	ns
DIPK1C	0.006901397	0.053822815	0.74349603	0.45719298	0.539831582	-6.91750844	ns
EDF1	-0.0112792	0.083597385	-0.74184815	0.458190568	0.540790808	-6.93336482	ns
POMC	-0.01115487	-0.02480853	-0.74028856	0.459136447	0.541688252	-6.91986674	ns
NOS2	-0.00648408	0.013017128	-0.73949221	0.459619718	0.541877984	-6.91950195	ns
KIAA2013	-0.00945594	0.130391587	-0.73941147	0.459668413	0.541877984	-6.9332205	ns
IL34	0.006013661	-0.02777598	0.734901712	0.462410811	0.544890867	-6.92356352	ns
GTF2IRD1	0.011846187	0.201916723	0.733578271	0.463217072	0.545620755	-6.93038063	ns
PNPT1	0.020947586	0.41178701	0.730437944	0.46513386	0.547491137	-6.92784042	ns
GIMAP7	0.004867343	0.012774345	0.730362437	0.46517997	0.547491137	-6.92800921	ns
NUP50	0.005132469	0.034373407	0.727684877	0.466817843	0.549197463	-6.92811579	ns
CD82	0.006670155	0.063498084	0.725998842	0.46785061	0.550190809	-6.93588184	ns
LY75	-0.00660159	-0.02239787	-0.71902535	0.472136248	0.554933672	-6.94491115	ns
CSRP3	0.015091408	0.120643488	0.718818779	0.472263758	0.554933672	-6.93449278	ns

ATXN2	-0.01771645	0.226510673	-0.71808985	0.472713016	0.555238136	-6.93646898	ns
DNLZ	0.014296289	0.235143263	0.716743897	0.473543236	0.555989646	-6.93742926	ns
EHD3	-0.01823906	-0.09078662	-0.71476485	0.474765422	0.557200576	-6.93833349	ns
PCARE	-0.01307024	0.190017493	-0.71356481	0.475507365	0.55784713	-6.93969027	ns
IFIT3	-0.0058779	0.031379092	-0.71174765	0.476631928	0.55894186	-6.94607076	ns
PRC1	-0.00708152	0.072129964	-0.70708841	0.479522201	0.562105511	-6.95647246	ns
RTBDN	0.004742746	0.002371294	0.704921213	0.480869999	0.563459229	-6.95234453	ns
PRSS2	-0.00834687	0.007863912	-0.70347257	0.481771961	0.564289662	-6.95498145	ns
KLK3	-0.01314244	0.561798654	-0.70268098	0.482265145	0.564640827	-6.96014215	ns
KLK4	-0.00972082	0.014642121	-0.7018868	0.482760284	0.564994	-6.96282006	ns
VSIG10	-0.00527176	0.022119706	-0.70117251	0.483205923	0.56528898	-6.96061563	ns
CD177	-0.02099107	-0.24156238	-0.70026166	0.483774527	0.565727521	-6.95768623	ns
MANEAL	0.007752383	-0.01158438	0.695086603	0.48701178	0.569285188	-6.94941497	ns
PECR	-0.00433561	0.021820899	-0.69464942	0.487285737	0.569377492	-6.95243082	ns
CUZD1	-0.00560085	0.048279027	-0.69266659	0.488529455	0.570602403	-6.95380681	ns
CTSC	-0.00673397	0.006153173	-0.69059164	0.489832681	0.571889692	-6.96227611	ns
CAPN3	0.005812061	0.041324709	0.690192225	0.490083679	0.571889692	-6.96821337	ns
SFTPD	-0.01072479	-0.00148899	-0.68985161	0.490297967	0.571889692	-6.96310767	ns
RTKN2	0.007690315	0.068110995	0.689665844	0.490414996	0.571889692	-6.95491009	ns
TMEM132A	-0.00954852	0.003499861	-0.6889226	0.490882577	0.572206437	-6.95637071	ns
RNF168	-0.00621994	0.034100253	-0.68497551	0.493369936	0.574876382	-6.9574298	ns
EPHA10	-0.01017542	0.094182104	-0.68132164	0.495678466	0.577335908	-6.96198981	ns
TNFSF12	-0.00451172	0.020499813	-0.67915836	0.49704765	0.578577006	-6.97773244	ns
EDIL3	0.005728433	-0.00434516	0.679012596	0.497140311	0.578577006	-6.96338866	ns
COL28A1	-0.01098197	0.109267738	-0.6725106	0.501268975	0.583149564	-6.96748922	ns
CD3G	0.013392081	0.247847091	0.670708807	0.502416354	0.584251594	-6.96704689	ns
FHIP2A	-0.00699005	0.014918149	-0.66852025	0.503811827	0.585641136	-6.96919062	ns
NDRG1	-0.01088228	0.134455926	-0.6664593	0.505127777	0.58693717	-6.97195025	ns
LYVE1	-0.00278875	0.000175936	-0.66608384	0.505367429	0.586982057	-6.9855422	ns
IL12RB2	0.006148418	0.076340952	0.665423154	0.505790087	0.587239385	-6.97055853	ns
EDEM2	-0.00533771	0.051127624	-0.66421716	0.506561519	0.587800394	-6.97204223	ns
AHSA1	-0.0097621	0.104091082	-0.66403802	0.506675887	0.587800394	-6.98582763	ns
ANGPTL1	0.004454723	0.012677382	0.659634933	0.509498226	0.590839881	-6.97444254	ns
MAG	-0.00669023	-0.02653284	-0.65321089	0.513630268	0.59539515	-6.98021175	ns
JCHAIN	-0.00933375	-0.03091229	-0.64528223	0.518754105	0.601096027	-6.98609324	ns
ADGRD1	0.005281084	-0.01126659	0.644581712	0.519208052	0.601383384	-6.98665655	ns
TJP3	0.005875203	0.033083203	0.643465848	0.519931654	0.601982724	-6.98699161	ns
PDIA2	-0.00575977	0.033149884	-0.64289241	0.520303726	0.602174745	-6.98413887	ns
CD3D	-0.00455922	0.032253359	-0.64057307	0.521809837	0.603678575	-6.98883861	ns
DDX53	-0.00505711	0.00335894	-0.63685977	0.524225831	0.606233436	-6.9907007	ns
RNF31	-0.00507563	0.023818947	-0.63442057	0.525816043	0.607831689	-6.99059642	ns
FLT3LG	-0.00482215	-0.01131521	-0.62544182	0.531690313	0.614378993	-7.01258432	ns
MRPL52	-0.00346978	-0.00228687	-0.62400796	0.532631858	0.615223506	-6.99470296	ns
CASP9	-0.00836861	0.027052238	-0.62334525	0.533067124	0.615482801	-6.99752302	ns
FGF3	0.004144525	0.022298657	0.617858093	0.536678244	0.619335367	-7.00090954	ns
TPMT	0.01045333	0.073347866	0.617630903	0.536828019	0.619335367	-7.0021824	ns
SH3BGRL2	-0.0083051	0.113726286	-0.61548999	0.538240469	0.620719656	-7.00450491	ns
TSLP	-0.00889695	0.094718357	-0.61138153	0.540956251	0.623605311	-7.00678379	ns
CEACAM1	0.002924791	-0.00458456	0.609341211	0.542307265	0.624916027	-7.01924489	ns
LYAR	-0.00781304	0.053242901	-0.60740569	0.543590882	0.626148077	-7.00841101	ns
CSH1	0.004114075	-0.01500649	0.606196051	0.544393464	0.626825282	-7.02222622	ns
CLASP1	-0.00989279	0.108088646	-0.60451781	0.545508564	0.627750906	-7.00794078	ns
TNNI3	0.011764446	-0.10155824	0.604339081	0.545627328	0.627750906	-7.01104106	ns
ACOX1	-0.00689552	0.023873932	-0.60386146	0.5459447	0.627868659	-7.01842904	ns
CCL11	-0.00541473	-0.02863414	-0.60182829	0.547297228	0.629176341	-7.02699695	ns
MYLPF	-0.0061905	0.075472313	-0.5997051	0.548711776	0.630554264	-7.01354629	ns
KIRREL2	0.005529531	0.000188261	0.598845681	0.549284799	0.630964442	-7.01449079	ns
VTCN1	0.004220863	0.000449847	0.597630092	0.550095777	0.63164753	-7.01426853	ns
CDK1	-0.00477648	-0.01174814	-0.59540523	0.551581601	0.633104667	-7.01337771	ns
ENO2	-0.00919047	0.024600322	-0.59465941	0.552079832	0.633427548	-7.0312279	ns
MSLNL	0.004762435	0.029639386	0.592202351	0.553723954	0.635064393	-7.01799297	ns
APRT	-0.00666215	-0.04580861	-0.58832124	0.55632539	0.637797463	-7.02658944	ns
COL9A1	-0.0061558	0.032680861	-0.58755224	0.556841521	0.638138635	-7.03033689	ns
LYPD1	-0.0087553	0.163751606	-0.58380299	0.559361602	0.640775158	-7.02333698	ns
METAP1	-0.00408915	0.010479665	-0.58149751	0.560913894	0.6423014	-7.02467289	ns
MORF4L1	0.005130384	0.042431567	0.580536548	0.561561416	0.642790802	-7.03038994	ns

ACRV1	-0.00931033	0.3736473	-0.57755352	0.563574242	0.644842001	-7.02486677	ns
NPPB	0.016900541	-0.10626043	0.574873494	0.565385441	0.646660983	-7.02831858	ns
THSD1	0.004230428	0.00957023	0.573673498	0.566197427	0.647336135	-7.02309612	ns
RAP1A	-0.00837133	0.117862017	-0.57093018	0.568055505	0.649206292	-7.02864917	ns
GLP1R	0.004551602	0.040982294	0.568649924	0.569602207	0.650719266	-7.03208502	ns
ANP32C	-0.00515881	0.067964884	-0.56799274	0.570048355	0.650974266	-7.03195145	ns
SSBP1	0.004431492	0.023045651	0.567117741	0.570642657	0.651398186	-7.03080657	ns
ICAM5	0.006064491	-0.04201857	0.566471098	0.57108188	0.651644818	-7.04018996	ns
GADD45B	-0.00614703	0.073312096	-0.56428865	0.572565997	0.65300154	-7.03963983	ns
RNF43	-0.0072949	0.106206631	-0.56406507	0.572718131	0.65300154	-7.03976528	ns
SPINK5	-0.00432952	0.02931036	-0.56195171	0.574157115	0.654386719	-7.04533086	ns
GSTM4	0.008837611	0.149705727	0.559615422	0.575750121	0.655946295	-7.03569664	ns
ENOPH1	0.006527535	0.092678459	0.556734324	0.577717026	0.657930466	-7.05097279	ns
SNED1	-0.00382766	0.001627895	-0.5558974	0.578289241	0.658325374	-7.03871097	ns
SIRT5	0.007164889	0.092522442	0.552196249	0.580822094	0.660951096	-7.04119091	ns
SCP2	0.010043782	0.213841977	0.551403775	0.581365104	0.661311298	-7.04068004	ns
TMPRSS11B	-0.00927191	0.181872052	-0.54904903	0.582979932	0.662889954	-7.04297876	ns
NXPH1	-0.00489415	0.010402595	-0.54843346	0.583402435	0.663112149	-7.04325025	ns
CEP112	-0.00528608	0.066259552	-0.54507392	0.585710767	0.665476825	-7.0436843	ns
HYAL1	-0.00287386	-0.01284062	-0.54247266	0.58750088	0.667251097	-7.05029512	ns
PFDN4	-0.00647457	0.061249285	-0.54161264	0.588093413	0.667664372	-7.04379008	ns
PER3	-0.01398144	0.093361329	-0.54062719	0.588772581	0.668175646	-7.04539797	ns
INSL3	0.008674065	0.640485637	0.539406435	0.58961419	0.668870798	-7.0614029	ns
MYO6	-0.00431689	0.021837581	-0.53616643	0.591851212	0.671147782	-7.06409387	ns
SIGLEC15	-0.00997358	0.215549695	-0.53578377	0.592115908	0.671187287	-7.05007007	ns
FGF20	0.00502995	0.08274584	0.533468521	0.593717182	0.672712888	-7.0492213	ns
INSL4	-0.00571313	0.050455278	-0.53317149	0.593922543	0.672712888	-7.06374457	ns
CTRB1	-0.00666951	0.01138384	-0.53207533	0.594681511	0.673311366	-7.06158552	ns
ADGRB3	0.00410725	0.013372381	0.530253533	0.595943884	0.674479125	-7.05795558	ns
PVALB	-0.01418682	-0.19307949	-0.52961596	0.596385835	0.674717798	-7.06618145	ns
TMED1	-0.00622564	0.028285274	-0.52515549	0.59948273	0.67795878	-7.06084058	ns
IL2	0.003824486	-0.01581604	0.523544753	0.600602896	0.678962623	-7.05684729	ns
PLCB1	-0.00519319	0.069120084	-0.52268353	0.6012022	0.679377099	-7.05488517	ns
APOB	0.002580146	-0.03276531	0.522157014	0.601568485	0.679528037	-7.06938469	ns
SLIRP	0.006532258	0.093171032	0.518711453	0.603969614	0.681976517	-7.05586429	ns
ENPEP	-0.00494271	0.057241602	-0.51834034	0.604228458	0.682005063	-7.0578204	ns
DDI2	-0.00889691	0.046574761	-0.51721916	0.60501073	0.682624162	-7.06023504	ns
GNPDA2	0.004110334	0.030758233	0.516405399	0.605578835	0.683001236	-7.06065329	ns
GFAP	-0.00516667	0.000249587	-0.5129315	0.608006785	0.685474831	-7.06034778	ns
FGF2	-0.00805249	0.05881199	-0.5113547	0.609110066	0.686453645	-7.07197798	ns
CACNA1C	-0.00359234	0.02314259	-0.5089495	0.610795068	0.688087036	-7.06407484	ns
CD164L2	-0.00403316	0.023539022	-0.50754505	0.611779838	0.688930632	-7.0642877	ns
MATN3	0.003725333	0.008724201	0.506949271	0.612197748	0.689135475	-7.06997547	ns
AKR1B10	-0.00660785	0.019895561	-0.50602924	0.612843505	0.689596545	-7.06505168	ns
IL9	-0.00620535	0.088876583	-0.50310137	0.614900262	0.691575882	-7.07211758	ns
PNLIPRP1	0.008271683	-0.00369922	0.502631167	0.615230753	0.691575882	-7.080461	ns
SCIN	0.008109375	0.081131353	0.502514403	0.615313062	0.691575882	-7.0673114	ns
CREB3	0.0060234	0.072483621	0.502165591	0.615558404	0.691585433	-7.06426324	ns
PRKAG3	-0.00355625	0.025996868	-0.50004671	0.61704947	0.692863721	-7.06804457	ns
PPY	0.011853272	0.035545384	0.499874291	0.617170732	0.692863721	-7.0790361	ns
CEND1	0.006347908	0.07432179	0.499170122	0.617666823	0.69315416	-7.06897716	ns
ERBB3	0.002227732	0.004432797	0.496752239	0.619370931	0.694799507	-7.07545688	ns
MAP1LC3B2	0.006873826	0.15389875	0.496036234	0.619876099	0.695099159	-7.06907047	ns
PNMA2	-0.00482989	0.013310812	-0.4945312	0.620938283	0.695885001	-7.07126935	ns
CPLX2	0.003291426	0.002855245	0.494185463	0.621182431	0.695885001	-7.06821677	ns
GBP4	-0.00560993	0.037923251	-0.49403049	0.621291849	0.695885001	-7.07145102	ns
CRYBB2	-0.00709556	0.138784028	-0.49132327	0.623204778	0.697649021	-7.08605071	ns
FAM3B	-0.0040106	-0.03352769	-0.49112566	0.623344622	0.697649021	-7.07950528	ns
CCL24	-0.00963245	0.04480936	-0.48910349	0.624775405	0.698982445	-7.08309351	ns
TGFBR3	0.003562514	-0.00747867	0.484599516	0.6279673	0.70228438	-7.08971915	ns
DDX39A	-0.00234368	0.007271949	-0.48405084	0.628356836	0.70245098	-7.07422434	ns
APOH	0.004072061	0.002796025	0.483179606	0.628975097	0.702873051	-7.08627257	ns
KRT17	0.004092176	-0.00040967	0.482600253	0.629386627	0.703054991	-7.07560874	ns
SLC28A1	-0.00635568	0.097488772	-0.48227243	0.629619453	0.703054991	-7.07400091	ns
INPP5J	-0.00436512	0.024664776	-0.47726505	0.633180111	0.706760674	-7.09378078	ns
DDX4	0.005665097	0.069556098	0.472589646	0.636512831	0.710209196	-7.07967623	ns

CCL25	0.005448549	-0.04666595	0.472243382	0.636759712	0.710213277	-7.09621703	ns
VGF	-0.00302642	0.015262499	-0.47072475	0.637843924	0.711150919	-7.09686431	ns
SCN3A	0.003776377	0.037731567	0.467533632	0.640124876	0.713421618	-7.08368762	ns
NPHS2	0.010669066	0.279344091	0.467121632	0.640419627	0.713477799	-7.08223124	ns
CASP4	-0.01719902	0.424528505	-0.46349038	0.643019514	0.71610106	-7.09115735	ns
CDH5	0.003191862	0.02301617	0.461732174	0.644279871	0.717231118	-7.10048139	ns
SMPDL3B	-0.00767458	0.164424298	-0.46125227	0.644624114	0.717340859	-7.09931202	ns
MN1	0.005869886	0.082549626	0.458478847	0.646615048	0.719118282	-7.087857	ns
UBAC1	0.003790253	0.007699667	0.458341105	0.646713907	0.719118282	-7.09498486	ns
PPIE	0.006115281	0.125400629	0.457248177	0.64749918	0.719717398	-7.09401546	ns
RICTOR	0.004807361	0.077930817	0.453356804	0.650298378	0.722553753	-7.08853143	ns
FGF12	-0.00457793	0.065087724	-0.45143736	0.651680875	0.723581052	-7.08939499	ns
RGL2	0.00625397	0.101106061	0.451384935	0.65171855	0.723581052	-7.09666475	ns
DCUN1D2	-0.00790687	0.144878423	-0.447907	0.654226777	0.726089772	-7.08989561	ns
DEFB104A_DE	0.00537613	0.097146164	0.445932164	0.655652651	0.727217265	-7.0939967	ns
PLXDC1	-0.00355787	0.029775412	-0.44581019	0.655740774	0.727217265	-7.09303999	ns
HRAS	0.00936753	0.270112446	0.445138842	0.656225743	0.727248176	-7.09857904	ns
PDE4D	-0.00589353	0.136357609	-0.44496725	0.656349829	0.727248176	-7.09296622	ns
BATF	0.0083318	0.214586247	0.444583907	0.656626845	0.727248176	-7.09409653	ns
SPINK8	-0.00368545	0.036054929	-0.44439299	0.656764877	0.727248176	-7.09322017	ns
EIF5	-0.00420597	0.045853798	-0.44268453	0.658000302	0.728339986	-7.09397377	ns
IFNW1	0.003390224	0.040090482	0.441576243	0.65880223	0.728568568	-7.09446108	ns
CORO6	0.003529408	0.031450729	0.441489289	0.658865145	0.728568568	-7.09595723	ns
UNG	-0.00334462	0.002390642	-0.44136447	0.658955338	0.728568568	-7.10823948	ns
ACSL1	0.003553908	0.04898679	0.439003438	0.660665355	0.730182754	-7.09558762	ns
VWA5A	-0.00751351	0.153254762	-0.43861514	0.660946735	0.730217354	-7.09399158	ns
PDXDC1	0.005270277	0.082029105	0.437123182	0.662028299	0.730910401	-7.09464125	ns
AGER	0.004152179	0.032440312	0.437046974	0.662083451	0.730910401	-7.10386807	ns
VSTM2L	-0.005868	0.021654526	-0.43641883	0.662539136	0.730910401	-7.09794295	ns
IL13	-0.00515508	0.067688192	-0.43636897	0.662575285	0.730910401	-7.09845829	ns
LELP1	-0.0069691	0.057995305	-0.43481615	0.663702108	0.731534124	-7.09886504	ns
REG3G	-0.00372195	0.001160403	-0.43453593	0.66390538	0.731534124	-7.11121403	ns
SPESP1	-0.00280266	0.025112008	-0.43447866	0.663946937	0.731534124	-7.11227134	ns
INSR	-0.00155782	0.002053235	-0.43420916	0.664142795	0.731534124	-7.09698199	ns
DUOX2	-0.00739429	0.152954815	-0.4337903	0.664446936	0.731593157	-7.09908035	ns
SLC39A14	-0.00322758	0.00447781	-0.43339694	0.66473263	0.731631843	-7.09941326	ns
TUBB3	0.002667969	-0.01333607	0.432813809	0.665156231	0.731822229	-7.09972891	ns
CLNS1A	-0.00324949	0.035504115	-0.43198027	0.665761916	0.731953229	-7.09959013	ns
GID8	-0.00315424	0.040262802	-0.43195979	0.665776636	0.731953229	-7.11426722	ns
ANKRA2	0.005339106	0.03782801	0.430833074	0.66659593	0.73257814	-7.09735594	ns
CEACAM5	-0.00636147	-0.01066302	-0.42875388	0.668108562	0.733964259	-7.09405258	ns
BHLHE40	-0.00661201	0.084472553	-0.42748794	0.669030074	0.734700194	-7.10200727	ns
COPE	0.004585627	0.040668882	0.426766845	0.669555255	0.735000505	-7.10224909	ns
ADAMTSL5	0.003608825	0.005379238	0.426173029	0.669987691	0.735198819	-7.11582637	ns
RANBP2	0.003063653	0.007332881	0.423910188	0.671637361	0.736732191	-7.10352196	ns
ESR1	-0.005432	0.099098763	-0.42235782	0.672769898	0.737697372	-7.10202975	ns
SMPD3	-0.00550557	0.115722524	-0.42168114	0.673263803	0.73779086	-7.10231375	ns
TEX33	-0.00364212	0.043406608	-0.42154856	0.673360494	0.73779086	-7.10961659	ns
CD3E	-0.00319973	0.022095897	-0.42118317	0.67362726	0.737806301	-7.10976972	ns
RUVBL1	0.010346007	0.285223451	0.419231661	0.675052822	0.739090454	-7.10447255	ns
CA7	0.003081416	0.021657627	0.417402868	0.676389627	0.740276503	-7.11134617	ns
GRIN2B	-0.00516602	0.105742331	-0.41468843	0.678375901	0.742172211	-7.10736742	ns
NPTN	-0.00783174	0.178361566	-0.41166108	0.680593722	0.744319726	-7.10760043	ns
CNTF	-0.00304427	-0.00629605	-0.40824871	0.683096885	0.746777575	-7.11000262	ns
CINP	-0.00406987	0.058903026	-0.40588339	0.684833974	0.748396408	-7.11606233	ns
NUDT2	0.005270307	0.044004771	0.402127433	0.687595862	0.751133527	-7.11903892	ns
LMOD2	-0.00564307	0.087807676	-0.40021155	0.689006378	0.752392903	-7.11323366	ns
IL17F	-0.00726167	0.155523911	-0.39641066	0.691807703	0.75516953	-7.11500528	ns
ZNF75D	0.005301181	0.052044865	0.392457782	0.694725478	0.758071149	-7.12139233	ns
SLC1A4	0.004510243	0.083987891	0.391954917	0.695097051	0.758193272	-7.11598872	ns
IGLON5	0.007789011	0.259901331	0.388873008	0.697375601	0.760394606	-7.11445843	ns
TMED4	0.004765349	0.08490461	0.387556651	0.698349641	0.761172434	-7.11673256	ns
GAS6	0.002249177	-0.00389406	0.386897686	0.698837302	0.761419747	-7.12813077	ns
CA8	-0.00216931	0.02581641	-0.38511144	0.700160302	0.762576681	-7.11698454	ns
ANPEP	-0.00248611	0.002068346	-0.38311234	0.701641788	0.763905303	-7.12720255	ns
GLI2	0.002793848	0.027447968	0.382638452	0.701993264	0.764003105	-7.11792863	ns

MMUT	-0.00418854	0.052119822	-0.37940412	0.704393153	0.766329362	-7.11984169	ns
TPT1	-0.00584942	0.091322776	-0.37858174	0.705003806	0.766430125	-7.12187604	ns
EPGN	0.003957501	0.100676841	0.378572262	0.705010725	0.766430125	-7.13384447	ns
MFAP3L	0.005527592	0.15983596	0.377695978	0.70566176	0.766852377	-7.12144579	ns
GSTT2B	0.019521354	-0.51650693	0.374988348	0.707674263	0.768753291	-7.13617252	ns
BHMT2	0.002096665	-0.01491583	0.373470824	0.708803323	0.769693456	-7.12206292	ns
TNPO1	-0.00290536	0.009632085	-0.37227291	0.70969483	0.770007726	-7.12906842	ns
FAM171B	0.002167549	0.026016658	0.372119461	0.709809011	0.770007726	-7.13625354	ns
CLPS	-0.0059164	0.003085875	-0.37158922	0.710203854	0.770007726	-7.13274028	ns
GABRA4	0.005221831	0.120533067	0.371449546	0.710307948	0.770007726	-7.12427005	ns
AMPD3	0.005924202	0.047963447	0.371020726	0.710627165	0.770007726	-7.13764456	ns
TMPRSS15	-0.0079289	-0.00061091	-0.37095667	0.710674939	0.770007726	-7.1310225	ns
SPACA5_SPAC	-0.00228966	0.002076908	-0.36305043	0.716572158	0.775908327	-7.14151298	ns
NPHS1	0.00269484	0.008526345	0.362943337	0.716652315	0.775908327	-7.12523035	ns
H2AP	-0.00409526	0.062393304	-0.3608032	0.718251642	0.777351666	-7.12492198	ns
UNC5D	-0.00209164	0.01333901	-0.35991225	0.718917812	0.777784369	-7.12631983	ns
CCAR2	-0.00425376	0.095377742	-0.35650778	0.721465179	0.780110811	-7.14289565	ns
CLSTN1	-0.00430686	0.03174935	-0.35632457	0.7216025	0.780110811	-7.12967914	ns
NT5C3A	0.00732293	-0.05079403	0.355712907	0.722060468	0.780317012	-7.13909034	ns
PDE1C	0.004213589	0.090444885	0.35300432	0.724090155	0.782220959	-7.12769007	ns
FKBP1B	-0.00906677	0.054004095	-0.35154957	0.725180884	0.783109535	-7.14293911	ns
EGFLAM	0.002306899	0.02256098	0.350248911	0.726156673	0.783873378	-7.14403555	ns
SMAD3	0.003939526	0.055930727	0.34907187	0.727040111	0.784260382	-7.14451993	ns
CALB1	-0.00344784	-0.01608964	-0.34905563	0.727052347	0.784260382	-7.13886434	ns
DDX1	-0.00379185	0.089173718	-0.34735972	0.728325819	0.78534394	-7.14503792	ns
PGM2	0.004583572	0.097148545	0.345635941	0.729621142	0.786450252	-7.13297716	ns
RLN1	-0.00542267	0.178978	-0.34502749	0.73007838	0.786461856	-7.14785979	ns
SPARCL1	0.002238426	0.006737745	0.344904941	0.73017058	0.786461856	-7.13962786	ns
IL18RAP	0.014054522	0.437480727	0.344381929	0.730563848	0.786595293	-7.13699513	ns
SLC4A1	0.003673368	0.081927052	0.343964406	0.730877726	0.786643184	-7.14686111	ns
SEMA4C	-0.00218216	0.004560033	-0.34344759	0.731266479	0.786771598	-7.14079562	ns
PMCH	0.006122503	0.231961657	0.342450528	0.732016657	0.787288633	-7.1331059	ns
ACE	0.001934703	-0.01228102	0.341335514	0.732855648	0.787900771	-7.14703547	ns
ABO	0.010678883	-0.17263805	0.340247158	0.733675024	0.788295258	-7.14740438	ns
PRSS53	0.002774075	0.003924755	0.340130977	0.733762504	0.788295258	-7.14856955	ns
RGMA	0.002411142	0.024678316	0.335567641	0.737201469	0.791698525	-7.15109186	ns
CCND2	-0.00268832	-0.02658451	-0.32949566	0.741785591	0.796069376	-7.14399681	ns
GUK1	0.003058066	0.021779593	0.329454589	0.741816703	0.796069376	-7.13744791	ns
ZPR1	0.006876203	0.223972318	0.329091898	0.742090798	0.796070952	-7.13902533	ns
NEO1	-0.0015801	0.00300611	-0.32593124	0.744480772	0.797919551	-7.15423204	ns
TET2	-0.00718807	0.202701237	-0.32573518	0.7446292	0.797919551	-7.1443513	ns
TST	-0.00572523	0.043955913	-0.32572914	0.744633828	0.797919551	-7.13892024	ns
RRP15	0.006471987	0.006131283	0.322284816	0.747241403	0.800419992	-7.14123017	ns
IL22RA1	0.003238935	0.035876052	0.319600835	0.749275361	0.802304384	-7.14235291	ns
LYSMD3	-0.00704952	0.162853046	-0.31489943	0.752842365	0.805828337	-7.14307272	ns
HMMR	-0.00327967	0.057454686	-0.31022282	0.756395828	0.809198389	-7.14046452	ns
WIF1	0.002484874	0.005403827	0.309889436	0.756649197	0.809198389	-7.1600693	ns
NLGN1	0.004570697	0.147300529	0.309662104	0.756822192	0.809198389	-7.14469926	ns
CBX2	-0.00266608	0.065873713	-0.30797744	0.758103766	0.810271961	-7.14425538	ns
MNAT1	0.002975091	0.041180447	0.307133344	0.758746067	0.810661733	-7.15107644	ns
DHRS4L2	-0.00319252	0.068242472	-0.30628262	0.759393584	0.81105679	-7.15945116	ns
TPSG1	0.001715574	0.01295535	0.305272369	0.760162944	0.811581645	-7.14439257	ns
TSHB	0.005517031	0.028700225	0.304484449	0.760762977	0.8119254	-7.15366164	ns
MTPN	0.0023391	0.025931889	0.302025819	0.762636549	0.813627594	-7.14729156	ns
CGA	-0.00584687	0.196103708	-0.29988974	0.764265317	0.815067467	-7.15765061	ns
ATF4	0.002265985	0.019725045	0.299500656	0.764562215	0.815086406	-7.14504945	ns
ID4	0.002909952	0.076025211	0.298986627	0.764954319	0.815206792	-7.15353198	ns
LILRA4	-0.00332128	0.083317148	-0.29807271	0.765651786	0.815652395	-7.14724026	ns
DDA1	0.002610209	0.034767663	0.294423132	0.768438659	0.818322715	-7.1483155	ns
DARS1	-0.00504512	-0.04285026	-0.29208412	0.770226285	0.819927361	-7.15510706	ns
GSN	0.001144245	0.004596979	0.287471312	0.773755328	0.823383949	-7.16220251	ns
PDCD6	-0.0047364	-0.18521749	-0.2868255	0.774249822	0.823610011	-7.15885437	ns
NEK7	-0.00464	0.112703258	-0.28463022	0.775931316	0.825098121	-7.15769858	ns
CCL2	0.003550992	0.028783068	0.283552419	0.776757257	0.825675715	-7.15779352	ns
RGS8	-0.00436487	0.149895962	-0.28254934	0.777526193	0.826192316	-7.15344708	ns
GGA1	-0.00458293	0.162136785	-0.28186844	0.778048263	0.826446318	-7.15330757	ns

CDH4	0.002894945	0.042352424	0.28139349	0.778412472	0.826532516	-7.15350511	ns
BRD1	0.001884705	0.026491922	0.278332729	0.780760723	0.828724577	-7.15946159	ns
HLA-DRA	-0.00224399	-0.00638852	-0.27687683	0.781878412	0.829248856	-7.16389156	ns
REG1A	0.003049363	0.030304079	0.27673084	0.781990559	0.829248856	-7.15728168	ns
CCDC28A	-0.00176776	-0.00903071	-0.27643905	0.782214647	0.829248856	-7.15438188	ns
TAGLN3	-0.0036668	-0.00809489	-0.27620995	0.782390616	0.829248856	-7.15171682	ns
RAB6B	0.002991718	0.037101954	0.275232516	0.783141381	0.829610544	-7.15514587	ns
GNGT1	0.003193017	0.068466667	0.275025909	0.783300092	0.829610544	-7.15476939	ns
PRKD2	-0.00283427	0.08054571	-0.27329287	0.784631849	0.830719724	-7.15428052	ns
ENOX2	0.001645438	0.017814115	0.272235484	0.785444698	0.831262922	-7.15456736	ns
TCL1B	-0.00501619	0.143829201	-0.27188487	0.785714269	0.831262922	-7.15589324	ns
PPM1A	0.001953607	0.023008063	0.264891232	0.791096883	0.836439454	-7.15697638	ns
UBQLN3	0.002755649	0.065592933	0.264783267	0.791180059	0.836439454	-7.15655735	ns
EVPL	0.001873472	0.029015047	0.259122517	0.795544206	0.840748853	-7.1645959	ns
REPS1	0.002203669	0.031770094	0.257693661	0.796646865	0.841609568	-7.15771145	ns
MYL3	0.003979209	0.031650908	0.254091373	0.799428433	0.844242685	-7.16026926	ns
PCNA	-0.00362914	0.143699095	-0.2525899	0.80058859	0.845162214	-7.15792751	ns
DEFB118	-0.00317578	0.098682971	-0.24855845	0.803705738	0.84814628	-7.16215793	ns
CEP152	-0.00223885	0.020859488	-0.24561013	0.805987404	0.850246828	-7.15965658	ns
TSPAN15	0.002666598	0.077819981	0.244827389	0.806593427	0.85057884	-7.16161427	ns
DOCK9	0.006159201	0.374213515	0.241821833	0.808921509	0.852725923	-7.16380063	ns
SHC1	-0.00600584	0.233263641	-0.24006694	0.810281641	0.853851459	-7.16099526	ns
SEZ6L	0.001533935	0.011829324	0.239571039	0.810666	0.853948311	-7.17535327	ns
GPR158	-0.00181227	0.046783736	-0.23826683	0.811677289	0.854705259	-7.17883034	ns
REG1B	0.003050024	0.042539651	0.236638429	0.812940429	0.855726768	-7.17866134	ns
CEACAM18	-0.00343277	0.137896503	-0.23507576	0.814153084	0.856694416	-7.1705056	ns
KHDC3L	0.003394616	0.114029158	0.234483362	0.814612944	0.856869523	-7.1623128	ns
DLL4	0.004421479	0.096239804	0.233059728	0.81571819	0.857723124	-7.16536396	ns
BRME1	-0.00206583	0.022215792	-0.22883087	0.819003477	0.860867586	-7.166841	ns
CTNNA1	0.001765798	0.053493578	0.228439501	0.819307681	0.860877448	-7.16692999	ns
SUMF1	0.003310284	0.130165319	0.225929457	0.821259358	0.862617743	-7.16535013	ns
ICAM2	0.001655375	-0.03876842	0.223052349	0.823497701	0.864657781	-7.18176739	ns
NECTIN1	-0.00156811	0.008548204	-0.22008082	0.825811204	0.866651827	-7.16664724	ns
BSND	0.001666221	0.025292972	0.219850696	0.825990423	0.866651827	-7.16561853	ns
GUCY2C	-0.00394074	0.227597508	-0.21822872	0.827253823	0.867635638	-7.17430331	ns
CPTP	0.001905766	0.063736817	0.217572735	0.827764983	0.867635638	-7.16788092	ns
PM20D1	-0.00962691	-0.5239588	-0.21750275	0.827819482	0.867635638	-7.17389146	ns
SLK	-0.00381294	0.186272559	-0.2165527	0.82855984	0.868100012	-7.17466569	ns
CAMLG	-0.00255649	0.086588549	-0.21458512	0.830093694	0.869134012	-7.16948447	ns
IL20RB	0.0019879	0.00850253	0.21452313	0.830142041	0.869134012	-7.16784829	ns
MICB_MICA	0.007592594	-0.52832135	0.213461197	0.830970088	0.869689124	-7.17935546	ns
HADH	-0.00301531	0.121435639	-0.21146174	0.832529851	0.871009375	-7.16918463	ns
TXK	-0.00348377	0.224679134	-0.20925749	0.834250025	0.872496444	-7.17621045	ns
COL2A1	-0.00365235	0.076571737	-0.20847976	0.834857199	0.87281884	-7.17076071	ns
TOP1	-0.00503237	-0.06258099	-0.20472625	0.837788701	0.875350023	-7.18420028	ns
IL21R	-0.00237943	0.098147031	-0.20461226	0.837877847	0.875350023	-7.16991386	ns
S100A14	0.002292881	0.084926831	0.201596752	0.840234626	0.877265739	-7.18685231	ns
ATP6V1D	-0.00128378	0.006619696	-0.2014973	0.840312422	0.877265739	-7.17926396	ns
ANK2	0.003134882	0.170783597	0.200475533	0.841111404	0.877786025	-7.17143501	ns
IL1RL1	0.002185996	-0.03532132	0.199909128	0.841554283	0.877934443	-7.18441098	ns
UROS	0.002981589	0.06859401	0.198038308	0.843017755	0.87904541	-7.17287955	ns
PALLD	-0.00173755	0.043042615	-0.19748782	0.843448485	0.87904541	-7.17133835	ns
IGFL4	0.003228212	0.178911512	0.197393425	0.843522343	0.87904541	-7.17135688	ns
SAP18	0.003125138	0.092825761	0.195879285	0.844707258	0.879966177	-7.17890576	ns
RIPK4	-0.00287723	0.172018718	-0.1949187	0.845459232	0.880435434	-7.17252755	ns
CKMT1A_CKMT	0.003090829	-0.0308338	0.194139514	0.846069226	0.880599153	-7.17905892	ns
RBM25	0.001977542	0.017726948	0.19394752	0.846219597	0.880599153	-7.17094832	ns
CRYGD	0.003986726	-4.33E-05	0.189577685	0.849642557	0.883622899	-7.18823141	ns
HTR1A	0.003079433	0.193318319	0.189465536	0.849730514	0.883622899	-7.17502908	ns
DUSP13	0.00168621	0.043209695	0.186486993	0.852065326	0.885735405	-7.18782398	ns
SHPK	0.001698919	0.068751513	0.185407138	0.852912173	0.886300194	-7.18089138	ns
SERPINA12	0.004758678	0.197030936	0.184511537	0.853614692	0.886505456	-7.16946555	ns
RAC3	0.002738345	0.160196769	0.184381191	0.853716898	0.886505456	-7.17547661	ns
TFF2	-0.00234597	-0.01632929	-0.18256287	0.855143423	0.887592353	-7.18548569	ns
HCG22	-0.00212917	0.06826037	-0.18227225	0.855371534	0.887592353	-7.17489951	ns
PNLIPRP2	-0.01237308	-0.91199875	-0.17608619	0.860228557	0.892315235	-7.18427666	ns

SMC3	-0.00209063	0.048593028	-0.17450986	0.861467132	0.89328268	-7.17450955	ns
CLINT1	0.001843837	0.025287201	0.17202517	0.863420059	0.894989908	-7.17493769	ns
SHH	0.001628272	0.006178161	0.171605038	0.863750356	0.895014564	-7.17608864	ns
LPO	-0.00240373	-0.00400836	-0.17079537	0.864386994	0.895286207	-7.17081057	ns
UPK3BL1	0.002009639	0.070514426	0.170491706	0.864625721	0.895286207	-7.18220786	ns
CYTH3	0.002715718	0.122892007	0.167573164	0.86692134	0.897345024	-7.17568947	ns
GPNMB	0.001086053	-0.01349286	0.165825053	0.868296884	0.898450354	-7.17267845	ns
LRTM1	-0.00147081	0.042338354	-0.16144641	0.871743972	0.901697626	-7.1794206	ns
NEFL	0.001544315	-0.03065688	0.160704404	0.872328382	0.901982605	-7.17829305	ns
PHLDB2	-0.0031935	0.156974196	-0.15735556	0.874966751	0.904390411	-7.17734032	ns
HEBP1	-0.0018922	0.021654101	-0.1540432	0.877577733	0.906768217	-7.18085173	ns
SCGB1A1	0.001917052	-0.03176435	0.152126497	0.879089166	0.908008618	-7.18973608	ns
GIPR	0.001455775	0.022491418	0.149443498	0.881205695	0.909872924	-7.17962654	ns
KCNIP4	-0.0017578	0.049283922	-0.14770143	0.882580383	0.910970208	-7.18196672	ns
ROBO4	0.000565289	-0.00486291	0.146991862	0.883140409	0.911226147	-7.18162939	ns
CRNN	-0.00226495	0.007228069	-0.14460643	0.88502357	0.912846636	-7.17791633	ns
NPTX2	-0.00102591	-0.01579928	-0.14378358	0.885673241	0.913194161	-7.19541926	ns
ITIH1	-0.00058085	-0.0184061	-0.14326874	0.886079804	0.913290868	-7.19598191	ns
CALY	0.001147524	0.022474385	0.141772971	0.887261234	0.914138221	-7.18142526	ns
IZUMO1	0.001471311	0.059786932	0.141435214	0.887528034	0.914138221	-7.17970563	ns
BST1	-0.00155478	-0.13496272	-0.13903713	0.889422617	0.915766587	-7.19748062	ns
DUSP3	-0.00279099	0.133040328	-0.13843025	0.889902238	0.915908608	-7.18328584	ns
UXS1	0.001652221	0.002101821	0.138068807	0.890187886	0.915908608	-7.1823883	ns
ENTPD5	-0.00067797	-0.01061553	-0.13758661	0.890568974	0.915977951	-7.18323819	ns
TMED10	-0.00150741	0.0723704	-0.13616177	0.891695222	0.916809605	-7.18220075	ns
OTOA	0.0015672	0.09108119	0.135769255	0.892005509	0.916809605	-7.18371321	ns
THAP12	0.002686299	0.18956309	0.133173574	0.894057897	0.918595728	-7.17940921	ns
LMOD1	0.00097129	0.004095823	0.13217924	0.894844234	0.919080254	-7.19837558	ns
MTR	0.001850722	0.098553785	0.131097885	0.895699629	0.919635343	-7.18287372	ns
YY1	-0.00077542	0.011204456	-0.12906536	0.897307654	0.920962513	-7.18244868	ns
CTLA4	-0.00081282	-0.0199997	-0.1261038	0.899651393	0.923043594	-7.19779229	ns
PRR5	-0.00129198	0.03743917	-0.12564212	0.900016897	0.923094253	-7.18288224	ns
CDAN1	0.000613418	0.008007341	0.122985068	0.902120517	0.924926934	-7.19858309	ns
IL1R2	-0.00055416	-0.01122243	-0.12197582	0.902919775	0.925421461	-7.19100341	ns
CSPG5	-0.00128447	0.057561548	-0.11987689	0.904582265	0.926800075	-7.18573349	ns
GPHA2	0.001183227	0.102839806	0.11665064	0.907138422	0.929093017	-7.19827735	ns
KEL	-0.00091304	-0.00622713	-0.10797695	0.914015413	0.935808207	-7.18701725	ns
SAG	0.001234415	0.010505829	0.106896613	0.914872422	0.936259709	-7.18719722	ns
C1QL2	-0.00097648	0.037425956	-0.10661269	0.915097674	0.936259709	-7.18507993	ns
GALNT10	0.000678125	-0.01727252	0.105636605	0.915872066	0.936723794	-7.19390541	ns
ATP5F1D	0.001185895	0.04692515	0.100118723	0.920251407	0.940873287	-7.1864355	ns
FGF19	-0.0017851	0.02515215	-0.09780696	0.922086849	0.94241988	-7.19843888	ns
SEMA6C	0.000568071	0.031047802	0.09719545	0.922572477	0.942586296	-7.1860343	ns
PGLYRP4	-0.00130005	0.107113057	-0.09569538	0.923763787	0.943473333	-7.1868662	ns
SNX18	0.000885412	0.09396236	0.091329618	0.927231867	0.946684284	-7.20089642	ns
CRISP3	-0.00046973	0.008000267	-0.07978023	0.936413091	0.955723952	-7.19724992	ns
ACY3	-0.00093091	0.017321984	-0.07786489	0.937936566	0.956825014	-7.18840503	ns
NRN1	-0.00060345	0.02868848	-0.07759997	0.938147265	0.956825014	-7.2040699	ns
RPGR	-0.00070898	0.026118205	-0.07700496	0.938620617	0.956973534	-7.189433	ns
LRP2	0.000591755	0.008463945	0.075324441	0.939957573	0.958002134	-7.18956029	ns
APOA4	0.000558323	0.017433054	0.073752312	0.941208425	0.958942289	-7.20348495	ns
SMPDL3A	0.001111766	-0.0438468	0.072970355	0.941830656	0.959241547	-7.1999091	ns
SERPINB5	-0.00080494	-0.03698574	-0.07244638	0.942247635	0.959331622	-7.19004152	ns
SERPINI1	0.000447782	0.028351068	0.068557759	0.94534255	0.962147175	-7.18983394	ns
ST8SIA1	-0.00072901	0.0905163	-0.06692943	0.946638775	0.96313074	-7.18850435	ns
LAG3	-0.00061029	0.011541399	-0.06540957	0.947848757	0.964025904	-7.19732716	ns
SOD3	0.000850667	0.093856886	0.064555831	0.948528487	0.964381331	-7.20290627	ns
APOBR	-0.00055549	-0.06262169	-0.05978691	0.952326141	0.967905441	-7.19059603	ns
CHAD	-0.00064929	0.005613369	-0.05918568	0.952804994	0.968055178	-7.19151795	ns
BCL7A	0.000769895	0.052545431	0.055893236	0.955427603	0.970142026	-7.18809905	ns
ATP1B3	0.000483758	0.053492184	0.05546002	0.955772718	0.970142026	-7.18989039	ns
CRHR1	-0.0007902	0.068512175	-0.05474342	0.956343601	0.970142026	-7.19116117	ns
FUT1	-0.00075895	0.076210297	-0.05460958	0.956450225	0.970142026	-7.1959016	ns
AFAP1	0.001008881	0.1946143	0.054521788	0.956520169	0.970142026	-7.19178166	ns
FOS	0.000416007	-0.02025912	0.053116535	0.957639765	0.970732296	-7.18682491	ns
VSIG10L	-0.00037496	0.011452428	-0.05285908	0.957844888	0.970732296	-7.19003049	ns

KLHL41	0.000568722	0.073846963	0.0521012	0.958448727	0.970732296	-7.20571471	ns
ITPRIP	-0.00052636	0.085454469	-0.05202791	0.958507147	0.970732296	-7.19153334	ns
EVI2B	0.000463168	0.035949431	0.051705096	0.958764364	0.970732296	-7.18832312	ns
GH2	0.001837031	0.507137291	0.047572566	0.962057528	0.973728936	-7.19074213	ns
CLEC10A	0.000387782	-0.01148855	0.046538836	0.96288139	0.974188175	-7.19763475	ns
GPR101	0.000613503	0.112691005	0.046166048	0.963178514	0.974188175	-7.19181957	ns
RYR1	-0.00032288	0.028298676	-0.04236823	0.96620572	0.976911601	-7.19052725	ns
FGF6	-0.0003516	0.044644467	-0.04049949	0.967695448	0.978079165	-7.18991621	ns
SCN2B	0.000252681	0.011103532	0.040076182	0.96803292	0.978081705	-7.19158298	ns
KIAA1549L	0.000242936	0.009595429	0.039469341	0.968516713	0.978232031	-7.19721141	ns
PSMC3	0.000252058	0.012868642	0.037450211	0.970126536	0.979351085	-7.18895507	ns
SELPLG	-0.00023278	-0.01906227	-0.03723835	0.970295442	0.979351085	-7.20132616	ns
L1CAM	0.000242744	-0.02232053	0.0364914	0.970891008	0.979613595	-7.2032344	ns
ACTN2	0.000445782	0.079728462	0.034484637	0.972491151	0.980889175	-7.19082853	ns
RELB	-0.00034713	0.077298736	-0.03245644	0.974108483	0.982181205	-7.19020799	ns
AMIGO1	0.000342623	0.071392548	0.030503127	0.975666202	0.983412258	-7.19191896	ns
CYP24A1	0.000551402	0.218778828	0.029634618	0.97635885	0.983770821	-7.19098316	ns
IL11	0.000412206	0.099355037	0.027469926	0.978085289	0.985170419	-7.19277036	ns
AXL	-0.00016842	0.001915937	-0.0268784	0.978557068	0.985305737	-7.20072222	ns
BCAT2	0.00031478	0.087976137	0.026044885	0.979221882	0.985635262	-7.19254207	ns
IL24	-0.00026496	0.030004918	-0.02344483	0.981295757	0.987050867	-7.19287227	ns
TLR4	0.000145472	0.003920223	0.023434072	0.981304338	0.987050867	-7.19045874	ns
OSTN	-0.00020861	0.042508414	-0.02246209	0.98207965	0.987490557	-7.19213074	ns
MOG	0.000160085	0.006724024	0.021050209	0.983205874	0.988282668	-7.20760935	ns
FH	-0.00037259	0.175841777	-0.02010873	0.983956903	0.988697232	-7.19267831	ns
ERC2	-0.00029269	0.145471502	-0.01819013	0.985487415	0.98989448	-7.1922171	ns
PRDX1	-0.00033739	0.10592598	-0.01686053	0.986548104	0.990619142	-7.18909153	ns
IL2RG	0.000127303	0.025331718	0.016416501	0.986902327	0.990634169	-7.20498572	ns
RNF4	-0.00021646	0.127070525	-0.01488275	0.988125914	0.991521536	-7.19787596	ns
TP73	-0.00010175	-0.00385965	-0.0136214	0.989132214	0.992190335	-7.19228937	ns
TGM2	-0.0002364	0.029870414	-0.01307211	0.989570431	0.992289031	-7.19855651	ns
LPA	0.000445255	-0.13674861	0.011361007	0.990935573	0.993316812	-7.19216549	ns
AGBL2	-0.00019122	-0.08226005	-0.00951092	0.992411626	0.994455027	-7.1928344	ns
PDCL2	-8.79E-05	0.067610293	-0.00756878	0.993961148	0.995666056	-7.18962398	ns
EDDM3B	-5.65E-05	0.185020185	-0.00505447	0.995967202	0.997333412	-7.20510705	ns
NAAA	-6.35E-05	0.052243935	-0.00455466	0.996365978	0.99737861	-7.19626549	ns
TRIM26	-5.04E-05	0.028640413	-0.00414169	0.996695474	0.99737861	-7.18964394	ns
SERPINC1	-6.12E-06	0.002459017	-0.00191139	0.998474957	0.998817018	-7.2044765	ns
EPB41L5	3.5881592904	0.057211143	0.00025718	0.999794803	0.999794803	-7.1923816	ns

Omnivore vs Sweet-tooth

	logFC	AveExpr	t	P.Value	adj.P.Val	B	protein_type
APOF	0.038478123	-0.01808688	7.475284087	8.1157117185	2.3697878218	20.64685201	ns
CNDP1	0.069723936	-0.0301518	6.995107291	2.7600664001	3.2631280145	17.21288595	ns
HAVCR2	-0.05161377	-0.03890064	-6.96769976	3.3525287820	3.2631280145	17.02289036	ns
EPHA1	-0.05123683	-0.0608638	-6.92577448	4.5076595132	3.2905914447	16.73473914	ns
TNFRSF19	-0.04242113	-0.0182122	-6.85289054	7.5096691830	4.3856468028	16.23496899	ns
DLL1	-0.04032231	-0.01921755	-6.66535556	2.7297315966	1.1462214944	14.98314436	ns
NECTIN2	-0.0396202	-0.01902468	-6.6432084	3.1729596731	1.1462214944	14.84010647	ns
TNFRSF1B	-0.04960829	-0.01985275	-6.6250988	3.5854724387	1.1462214944	14.71922415	ns
DSC2	-0.04653203	-0.01553323	-6.59991746	4.2502086814	1.1462214944	14.55847713	ns
TAFA5	-0.04840147	-0.0233528	-6.59457387	4.4045858706	1.1462214944	14.52152332	ns
RELT	-0.03773343	-0.03067685	-6.58626698	4.6566382401	1.1462214944	14.46540117	ns
GGH	-0.05204962	-0.04112374	-6.58466196	4.7104992921	1.1462214944	14.46138825	ns
TNFRSF1A	-0.03926787	-0.02483846	-6.54666256	6.0701896621	1.3634579856	14.20805632	ns
BTN2A1	-0.03373757	-0.01175012	-6.37439166	1.8891260411	3.9401771715	13.10613831	ns
NPC2	-0.03935316	-0.03478036	-6.3630962	2.0338385463	3.9592057034	13.03883907	ns
INHBB	-0.06499336	-0.02699148	-6.32117024	2.6685747271	4.8701488770	12.77828088	ns
CTSO	-0.04195035	-0.0237383	-6.2614499	3.9143999250	6.7235575183	12.39736422	ns
PTGDS	-0.03780882	-0.01244456	-6.24131621	4.4545931102	7.2263399344	12.2806996	ns
EPHA2	-0.03815081	-0.02213227	-6.19178638	6.1045587740	9.3817429580	11.98180107	ns
FGFR2	-0.02783331	-0.02253775	-6.16722931	7.1228331662	1.0399336422	11.81990752	ns
TNFRSF4	-0.04767074	-0.01322573	-6.14243961	8.3246846670	1.1563719121	11.66711926	ns
CKAP4	-0.03350685	-0.00595406	-6.13520541	8.7123911190	1.1563719121	11.62456768	ns
FAM3C	-0.03730862	-0.02927723	-6.07715003	1.2524215602	1.5900308504	11.27710858	ns
SERPINA7	-0.02300735	-0.01020756	-6.04179002	1.5590208196	1.8968086639	11.06044798	ns

EFNA4	-0.03610642	-0.02651292	-6.02004126	1.7837320173	2.0833989963	10.9359934	ns
GFRA1	-0.03462859	-0.0221911	-5.95059096	2.7293371936	3.0652556175	10.51820473	ns
CSF1	-0.03379919	-0.0134698	-5.93592899	2.9845640057	3.1606808949	10.43455921	ns
COL6A3	-0.04247882	-0.03672984	-5.93073479	3.0799124841	3.1606808949	10.40168039	ns
WFDC2	-0.04471095	-0.01115656	-5.92778601	3.1390323957	3.1606808949	10.40259286	ns
TNFRSF14	-0.036769	-0.0197507	-5.8617027	4.6753805620	4.5507037470	10.00255476	ns
CST3	-0.03443216	-0.04064463	-5.82461642	5.8391328790	5.5000864538	9.78875194	ns
COLEC12	-0.03221571	-0.03369293	-5.78980365	7.1848115785	6.5561405653	9.588824745	ns
RNASET2	-0.0318881	-0.01705792	-5.71768513	1.0998219623	9.7317579701	9.175055945	ns
CD83	-0.04038972	-0.0265991	-5.64713358	1.6603711066	1.4259657739	8.779227238	ns
EGFR	0.019556498	0.012870119	5.606859735	2.0954334509	1.7255265061	8.551481286	ns
TGFBR2	-0.03630374	-0.01406392	-5.60424968	2.1273614459	1.7255265061	8.539060606	ns
PI3	-0.06505047	-0.03348576	-5.5913886	2.2916464614	1.8085426128	8.476126523	ns
SHISA5	-0.02662199	-0.02141815	-5.56047232	2.7349418615	2.1015869041	8.295713652	ns
PSAP	-0.03113623	-0.01470993	-5.53529472	3.1580499569	2.3644886857	8.160791706	ns
LTBR	-0.03167205	-0.00391684	-5.52500899	3.3486829386	2.4445385452	8.107474025	ns
CD27	-0.04593542	-0.03557877	-5.51994743	3.4456677087	2.4539877340	8.074676797	ns
HLA-E	-0.033098	-0.00444427	-5.51334501	3.5775953115	2.4872805499	8.039880494	ns
CTSZ	-0.0380229	-0.02756025	-5.4910825	4.0584493485	2.7346901340	7.920485808	ns
KRT18	-0.09810502	-0.00335384	-5.48838489	4.1207659554	2.7346901340	7.905971578	ns
IGFBP4	-0.04629484	-0.04467782	-5.44218233	5.3446091238	3.4680574759	7.657687841	ns
LAIR1	-0.04337119	-0.03529584	-5.42453516	5.8986893793	3.7443854321	7.560648437	ns
AGRN	-0.03802839	-0.02782697	-5.35289788	8.7795853652	5.4545509077	7.175620079	ns
TNFRSF8	-0.05155627	0.016251564	-5.33712778	9.5758932449	5.8253350573	7.089170087	ns
OCLN	-0.0458463	-0.03554469	-5.32635303	1.0160144585	6.0546167734	7.032280054	ns
RNF149	-0.03164317	-0.02630013	-5.31564796	1.0779238051	6.2950750223	6.987166815	ns
ALCAM	-0.02161813	-0.00660483	-5.28981468	1.2410368680	6.9907146265	6.840513271	ns
LTBP3	-0.06357854	0.009939478	-5.28931892	1.2449217828	6.9907146265	6.848897146	ns
ATRAID	-0.02815427	-0.00162156	-5.2801193	1.3084655306	7.2089044329	6.791000355	ns
B2M	-0.03182519	-0.0022426	-5.27412724	1.3518256226	7.3098718854	6.759026032	ns
EFCAB14	-0.0275523	-0.00638845	-5.20042851	2.0136526176	1.0690664806	6.377315985	ns
NBL1	-0.0274597	-0.01560759	-5.14633887	2.6887769475	1.3825615127	6.101032153	ns
NECTIN4	-0.03150401	-0.00957149	-5.1456368	2.6988358296	1.3825615127	6.097453055	ns
LY6D	-0.0397199	-0.01867656	-5.13662538	2.8316476651	1.4159688926	6.055994886	ns
CD302	-0.03176318	-0.0288168	-5.13467263	2.8610330365	1.4159688926	6.045138178	ns
TNFRSF10B	-0.04312728	-0.03318523	-5.12445317	3.0204217612	1.4428748289	5.993469234	ns
TNF	-0.04233697	-0.00662835	-5.12425628	3.0240695834	1.4428748289	5.996990778	ns
INHBC	-0.05088095	-0.10462579	-5.12175621	3.0636383354	1.4428748289	5.977066979	ns
IL10RB	-0.03127992	-0.02621208	-5.11604988	3.1574619855	1.4634585710	5.947511331	ns
FCAMR	-0.07738115	-0.02923245	-5.09916419	3.4518079725	1.5748873874	5.859768419	ns
PIK3IP1	-0.02738431	-0.01434001	-5.09546287	3.5201418824	1.5813560456	5.843999437	ns
SIRPB1	-0.0495077	-0.03952379	-5.0567653	4.3135708494	1.8838243401	5.65208173	ns
LGALS4	-0.05833077	-0.03602431	-5.05636797	4.3224736571	1.8838243401	5.649576674	ns
SPON2	-0.03644497	-0.02850134	-5.03945906	4.7220176318	2.0276899242	5.567029309	ns
CFD	-0.01966453	-0.01534626	-5.01359052	5.4013534243	2.2559463489	5.43132594	ns
CD79B	-0.04731832	-0.02614915	-5.01334523	5.4080905626	2.2559463489	5.429149874	ns
SUSD4	-0.04180014	0.018281077	-4.9915112	6.0560880153	2.4906728175	5.322418969	ns
CD300E	-0.04314428	-0.02326622	-4.93532766	8.0849208324	3.2788845598	5.04531779	ns
FABP1	-0.10334502	-0.09211696	-4.92946335	8.3318048975	3.3327219590	5.021853815	ns
OGN	-0.04244728	-0.04045438	-4.90353508	9.5088702956	3.7521488193	4.893352655	ns
CCER2	-0.05977518	0.035620043	-4.88633187	1.0378735786	4.0407877996	4.818102312	ns
SPINK4	-0.06607543	0.028102072	-4.85252076	1.2309995149	4.7296297154	4.64709304	ns
VWC2	-0.04070238	-0.04137541	-4.8342832	1.3493229653	5.1169130633	4.563960086	ns
EFNA1	-0.02725113	-0.01533742	-4.81315376	1.4998283537	5.6147420423	4.45923682	ns
FSTL1	-0.0215922	-0.01952285	-4.81056813	1.5195741376	5.6166537745	4.454204671	ns
PLA2G15	-0.02698216	-0.01449308	-4.8036888	1.5723215323	5.7159956772	4.412074912	ns
CDHR5	-0.04283945	-0.06542682	-4.80201845	1.5856015405	5.7159956772	4.407414676	ns
CD59	-0.02232296	-0.02144653	-4.78832399	1.6981651052	6.0471245211	4.357044727	ns
NPPC	-0.04650567	-0.00057409	-4.76712374	1.8858928202	6.6347072711	4.240110252	ns
TMPRSS11D	-0.03526223	0.003513503	-4.74582671	2.0951236322	7.2830488170	4.136821308	ns
PRG2	-0.04865187	0.009338928	-4.71828223	2.3992294355	8.2420587667	4.007689841	ns
LAMP3	-0.05439971	-0.04649444	-4.70484711	2.5624869722	8.7005371616	3.944297933	ns
GDF15	-0.03914234	-0.05624946	-4.68296621	2.8514930737	9.5044030713	3.842335689	ns
HSPG2	-0.02505867	-0.01699601	-4.68208525	2.8643406516	9.5044030713	3.847774686	ns
ASGR1	-0.03139906	-0.04427822	-4.67795128	2.9223948862	9.5880820986	3.825369094	ns
TNFRSF12A	-0.03432786	-0.03569182	-4.66809899	3.0655669968	9.9460618121	3.775764546	ns

ICAM1	-0.02922898	-0.02943166	-4.6625261	3.1500801908	0.000101079	3.756056088	ns
TTR	0.023188898	-0.00211707	4.65814873	3.2174934505	0.00010212	3.733119202	ns
IL2RA	-0.04029842	-0.01663132	-4.64726252	3.3919355855	0.000106499	3.68580793	ns
TNFRSF21	-0.02607033	-0.00547418	-4.62380525	3.7980476626	0.000117982	3.569106736	ns
CFH	-0.0252314	-0.04566867	-4.61248323	4.0127739498	0.00012334	3.544146235	ns
ULBP2	-0.03538657	-0.02186822	-4.59382047	4.3868322311	0.000131356	3.438899778	ns
PDCD1	-0.04132275	0.010834746	-4.59309856	4.4015096570	0.000131356	3.429097389	ns
ANG	-0.03460943	-0.02723474	-4.59220004	4.4210910501	0.000131356	3.432828243	ns
RNASE6	-0.02601364	-0.02731617	-4.59065973	4.4535145170	0.000131356	3.422679633	ns
CRHBP	-0.02839422	-0.04154438	-4.5853443	4.5682414158	0.000133393	3.399745846	ns
ADM	-0.029065	-0.05568744	-4.57027734	4.9083545681	0.000141905	3.324700048	ns
SPINK1	-0.03804144	-0.00950071	-4.56117102	5.1262262789	0.000146751	3.287914834	ns
NPDC1	-0.02811317	-0.01434314	-4.54735457	5.4741065668	0.000155188	3.227791712	ns
IL18BP	-0.02903115	-0.01793789	-4.54366028	5.5707199964	0.000156409	3.209937921	ns
A1BG	-0.01612336	-0.02054699	-4.53297235	5.8594775513	0.000162502	3.157999919	ns
SUOX	-0.05805137	0.065689172	-4.53141662	5.9026629249	0.000162502	3.149974212	ns
SMOC1	-0.0324224	-0.05614878	-4.52955993	5.9547086275	0.000162502	3.141087513	ns
FCER2	-0.04978537	-0.0254095	-4.52272922	6.1504024392	0.000166289	3.114883269	ns
FSTL3	-0.03023199	-0.0245828	-4.50285991	6.7540254070	0.000180934	3.028584133	ns
JAM2	-0.02361254	-0.01175586	-4.49604301	6.9731508250	0.000185105	2.991714207	ns
CD72	-0.03834442	-0.01876961	-4.46652378	8.0076129511	0.000210651	2.8722783	ns
EPHB4	-0.02220574	-0.01053221	-4.45730619	8.3577775823	0.000217899	2.820246309	ns
C1QA	-0.02230995	9.8688376300	-4.45004137	8.6453184241	0.000223401	2.789142119	ns
CD5	-0.03273872	-0.01939922	-4.43509323	9.2665764087	0.000237354	2.721914782	ns
SULT2A1	-0.05262241	0.05863813	-4.42129012	9.8787279893	0.000250834	2.665623423	ns
EFEMP1	-0.02934196	-0.03396737	-4.41154311	1.0333659565	0.000259146	2.624458139	ns
BSG	-0.01783169	-0.0147872	-4.41048066	1.0383584797	0.000259146	2.614648677	ns
SORCS2	-0.0319257	-0.0212106	-4.40444639	1.0678673180	0.000264252	2.600244297	ns
PRAP1	-0.03998382	-0.09612293	-4.4025421	1.0770636173	0.000264288	2.580659485	ns
CLEC14A	-0.02883359	-0.01898047	-4.40067456	1.0864180564	0.000264362	2.575180579	ns
TNFSF10	-0.02511613	-0.01090176	-4.39786677	1.1004691894	0.000265568	2.559033082	ns
RGMB	-0.02390677	0.002459327	-4.37181832	1.2401818096	0.00029683	2.445780634	ns
MMP12	-0.04887664	-0.05599083	-4.34772067	1.3844350625	0.000328663	2.344843039	ns
TIMD4	-0.04175989	-0.02280482	-4.33189353	1.4877103787	0.000350332	2.278572994	ns
EPHB6	-0.0279716	0.000193873	-4.32135494	1.5605701898	0.000360706	2.236396056	ns
CD46	-0.02647938	-0.00838459	-4.32063766	1.5656886841	0.000360706	2.235279438	ns
RARRES2	-0.04808087	-0.0752127	-4.31973173	1.5719800645	0.000360706	2.22518365	ns
NTRK3	0.021039151	0.014814134	4.318432077	1.5811760292	0.000360706	2.215850093	ns
AMBP	-0.01847657	-0.03425718	-4.30534186	1.6775215875	0.000379718	2.159866244	ns
TGOLN2	-0.02187995	-0.01089464	-4.29347105	1.7697182140	0.000395344	2.111131748	ns
PIGR	-0.0336137	-0.01968801	-4.2930108	1.7736312448	0.000395344	2.117457681	ns
IL18R1	-0.03209823	-0.03755503	-4.28987272	1.7985641271	0.000397864	2.094044309	ns
SERPINF1	-0.0244105	-0.03790164	-4.26808641	1.9834767179	0.00043547	2.008958538	ns
TNFRSF17	-0.03462875	-0.03280297	-4.26473255	2.0135528673	0.000438774	1.999303452	ns
BGLAP	-0.07443445	-0.05549405	-4.2571463	2.0827248078	0.000450486	1.956663347	ns
SCGN	-0.0407139	0.019422048	-4.24071202	2.2413741040	0.000481236	1.898137949	ns
GUSB	-0.05698715	-0.04504515	-4.23276855	2.3216855182	0.000494841	1.85597818	ns
PRSS8	-0.03788736	-0.05488094	-4.21279918	2.5367333526	0.000536758	1.776297372	ns
SCRG1	-0.0220243	-0.02391007	-4.20664593	2.6064704127	0.000547546	1.744334172	ns
NOTCH2	-0.01798643	0.0187563	-4.20466539	2.6293764202	0.000548413	1.736565164	ns
COL15A1	-0.0222527	-0.01423475	-4.19720034	2.7174282885	0.000562758	1.705478965	ns
TFPI2	-0.03304866	-0.00301056	-4.18291038	2.8939395372	0.000595092	1.648458483	ns
SUMF2	-0.04856573	-0.07254732	-4.17268587	3.0267001909	0.000618039	1.602482057	ns
CRELD2	-0.04762123	-0.02925098	-4.16873717	3.0796831740	0.000624491	1.590501783	ns
BPIFB2	-0.05719183	0.05996641	-4.16642294	3.1109833183	0.000625385	1.578387485	ns
GM2A	-0.03146205	-0.00674073	-4.16528467	3.1269265010	0.000625385	1.583859742	ns
LMNB2	-0.02303825	-0.02366368	-4.15207036	3.3124337204	0.00065798	1.518282749	ns
PON3	0.025690986	0.001823046	4.137420416	3.5309614820	0.000696649	1.460568864	ns
POF1B	-0.03689791	0.042908855	-4.12402402	3.7431101806	0.000733549	1.416418083	ns
PTPRN2	-0.02835457	-0.00382663	-4.11096078	3.9608601775	0.000771047	1.358593914	ns
ASGR2	-0.02122855	-0.00895729	-4.10636857	4.0399210663	0.000776231	1.332189635	ns
GIGYF2	-0.06562756	0.043087227	-4.10632324	4.0406540192	0.000776231	1.330168082	ns
TNFRSF9	-0.03483861	-0.0101551	-4.1047087	4.0692893938	0.000776623	1.33064448	ns
NHLRC3	-0.02688995	-0.04392814	-4.08289449	4.4712416175	0.000847794	1.257557218	ns
IFI30	-0.03247403	-0.04655162	-4.07550023	4.6150401778	0.000869414	1.215967406	ns
THY1	-0.02426571	-0.01898924	-4.07081785	4.7080720703	0.000881255	1.186247616	ns

PLAUR	-0.02363523	-0.03703801	-4.0655322	4.8164382038	0.000895796	1.174689951	ns
XG	-0.02713365	-0.006128	-4.04391218	5.2822377810	0.000970404	1.081544095	ns
PODXL2	-0.02392797	0.013059613	-4.04383014	5.2840489794	0.000970404	1.080563573	ns
CD74	-0.030047	-0.03293008	-4.03846217	5.4061642553	0.000980229	1.056189749	ns
PRRT3	-0.03048921	0.015882938	-4.0381443	5.4142245327	0.000980229	1.067865763	ns
NUCB2	-0.03329116	-0.00627497	-4.03478807	5.4913960938	0.000980229	1.04160894	ns
ACP5	-0.02899446	-0.05512518	-4.03440357	5.5005205720	0.000980229	1.043186193	ns
PBLD	-0.04546435	0.022806931	-4.03422091	5.5053968761	0.000980229	1.051413525	ns
CAPG	-0.05270552	-0.06983188	-4.03215853	5.5535447681	0.000982809	1.037759319	ns
TFF1	-0.06426111	0.023579233	-4.02398754	5.7496638112	0.001011387	1.005104083	ns
CD38	-0.02656845	-0.02282377	-4.00585137	6.2086763688	0.001072318	0.932969714	ns
IGFBP6	-0.02389185	-0.01031694	-4.00543441	6.2198147735	0.001072318	0.933771796	ns
YAP1	-0.02159165	-0.01888789	-4.00465269	6.2398252799	0.001072318	0.922268512	ns
SPOCK1	0.022366789	0.00351267	4.004301436	6.2495307692	0.001072318	0.927115449	ns
RNASE4	-0.0217811	-0.03006874	-4.0023482	6.3019588322	0.001072318	0.928065569	ns
IGLC2	-0.01916784	0.004191152	-4.00158197	6.3213574294	0.001072318	0.911042849	ns
FAM20A	-0.02355982	-0.04298675	-4.00039338	6.3531164950	0.001072318	0.905316199	ns
IL1RN	-0.05138433	-0.00163616	-3.99893986	6.3925674507	0.001072776	0.904982786	ns
ADAM9	-0.02342769	-0.00934361	-3.99539268	6.4886337497	0.001082675	0.886513539	ns
PRCP	-0.0278459	-0.04334977	-3.99048444	6.6243016408	0.001099032	0.868200603	ns
GHRL	0.070816177	0.053246639	3.974084137	7.0974591057	0.00117088	0.806932157	ns
RNASE1	-0.01910243	-0.01455021	-3.96930055	7.2409344724	0.001187839	0.783524806	ns
KRT8	-0.04929242	0.035507696	-3.96308985	7.4326807185	0.001212482	0.769413206	ns
IFNLR1	-0.03306251	-0.01527757	-3.95723554	7.6163522234	0.001235542	0.738681751	ns
SEMA3F	-0.02316376	-0.02214061	-3.94503147	8.0145384831	0.001292953	0.692898541	ns
LRP11	-0.0297707	-0.02729547	-3.94305281	8.0809085868	0.001296497	0.685162544	ns
ISM1	-0.03382449	-0.03915717	-3.94148158	8.1338973471	0.001297868	0.677003032	ns
HGF	-0.03120691	-0.04313962	-3.93806378	8.2500440359	0.001309246	0.659256819	ns
LGALS9	-0.02812784	-0.04005442	-3.92859809	8.5817375915	0.001354523	0.628597393	ns
IL19	-0.0584084	-0.00312237	-3.91997588	8.8952474862	0.001396458	0.603856701	ns
STC1	-0.03709145	-0.02194037	-3.90743009	9.3679122746	0.001462797	0.540079445	ns
AHSG	-0.02150113	-0.01146139	-3.89924211	9.6901487045	0.001504034	0.510268211	ns
ADGRG1	-0.05294034	0.085784056	-3.89813438	9.7350129700	0.001504034	0.510982952	ns
ST3GAL1	0.037429241	0.060155425	3.890780972	0.000100345	0.001542149	0.482594159	ns
KRT19	-0.05472328	0.016545685	-3.87796544	0.000105774	0.001617064	0.4316253	ns
IGFBP3	0.023193248	-0.00385257	3.862912326	0.000112503	0.00171099	0.373804376	ns
TNFRSF11A	-0.03106236	-0.0354379	-3.85675456	0.000115366	0.001745435	0.345072001	ns
CCL22	-0.04368552	-0.04447282	-3.8522423	0.000117522	0.001768888	0.338287652	ns
RBFOX3	-0.02880542	-0.03056084	-3.84699453	0.000120054	0.001797729	0.307769239	ns
CALB2	-0.0256374	-0.00223921	-3.84453891	0.000121276	0.001806761	0.310775471	ns
CDNF	-0.02566901	-0.00268305	-3.83654906	0.000125276	0.00185689	0.274876877	ns
SNAP25	-0.03603262	0.03154438	-3.83392926	0.000126625	0.0018674	0.270309158	ns
MEP1A	-0.05522809	0.00702397	-3.82436771	0.000131623	0.001931351	0.223574147	ns
CCN3	-0.02628364	-0.02570954	-3.80897317	0.000140084	0.002045229	0.16512784	ns
TREM2	-0.04233138	-0.06070506	-3.7939874	0.000148817	0.002161921	0.113545176	ns
LRIG1	-0.02852797	-0.02725682	-3.78914979	0.000151736	0.002193416	0.089430136	ns
CLMP	-0.01790673	-0.01790542	-3.77162532	0.00016279	0.002337243	0.022527274	ns
SPON1	-0.02588615	-0.03630137	-3.7708738	0.000163287	0.002337243	0.025633501	ns
SFTPA2	-0.04118026	0.029464584	-3.76820786	0.000165051	0.002350977	0.023559469	ns
IGSF8	-0.02424183	-0.02036144	-3.76366539	0.000168066	0.002382296	-9.70E-05	ns
PPL	-0.02465401	-0.00607081	-3.75494563	0.000174033	0.002454952	-0.02467763	ns
FBLN2	-0.02264133	-0.00874705	-3.74775272	0.000179074	0.002513928	-0.06455121	ns
BAMBI	-0.02341291	0.002512311	-3.73809909	0.000186104	0.002600108	-0.08845558	ns
LAYN	-0.0256415	-0.00549234	-3.73615053	0.000187541	0.002607711	-0.10052302	ns
AREG	-0.03593174	0.02018985	-3.72540402	0.000195704	0.002708317	-0.14173372	ns
IL6	-0.0610138	-0.00484653	-3.72129137	0.000198916	0.00273979	-0.15691484	ns
GFRA2	-0.016755	-0.0100504	-3.71935284	0.000200448	0.002747921	-0.16406477	ns
FABP5	-0.05714523	-0.02514401	-3.71611427	0.000203024	0.002769685	-0.1812727	ns
NELL1	0.032729428	0.038081458	3.71479467	0.000204086	0.002769685	-0.18488872	ns
PLTP	0.029808528	0.006951874	3.713809754	0.000204881	0.002769685	-0.18892914	ns
VSIG4	-0.03190339	-0.04366268	-3.70385453	0.000213092	0.002867408	-0.22389024	ns
TNFRSF13B	-0.02704097	-0.01794622	-3.70186868	0.000214766	0.002870131	-0.22998414	ns
IL1R1	-0.01749426	-0.01258122	-3.70128686	0.00021526	0.002870131	-0.23225927	ns
CCL15	-0.03450433	0.03510263	-3.69697247	0.000218938	0.0029059	-0.25288645	ns
LGALS1	-0.02719131	-0.03511353	-3.68335368	0.000230978	0.003051835	-0.29656869	ns
HS6ST2	-0.02411379	0.026341478	-3.67756728	0.00023629	0.003107953	-0.30972074	ns

SCARB2	-0.02251376	-0.02528381	-3.67522608	0.000238466	0.003115937	-0.31831606	ns
PTPRS	-0.01931551	0.002261013	-3.67459336	0.000239031	0.003115937	-0.33467337	ns
DCBLD2	-0.02691207	-0.02543758	-3.66783152	0.000245438	0.003176231	-0.35676956	ns
GRN	-0.01918842	-0.01926381	-3.66671281	0.000246522	0.003176231	-0.35673454	ns
ADIPOQ	0.051594425	0.057234325	3.666288117	0.000246919	0.003176231	-0.36420828	ns
IGSF3	-0.02756497	0.002100542	-3.65242357	0.000260644	0.003338073	-0.40864133	ns
AMN	-0.03814656	-0.00356769	-3.64772403	0.000265464	0.003384951	-0.42009245	ns
COL18A1	-0.01810204	-0.03496446	-3.63992479	0.000273613	0.003464586	-0.45527785	ns
TFRC	-0.03089503	0.008120676	-3.63948292	0.000274082	0.003464586	-0.45764757	ns
GFER	-0.05209892	0.021887839	-3.62596525	0.000288835	0.003635338	-0.49774134	ns
CD80	-0.02397172	-0.01691122	-3.61930463	0.000296337	0.00371375	-0.53419217	ns
CCL3	-0.0436025	-0.02506131	-3.6166492	0.000299398	0.003736084	-0.54002207	ns
AKR1B1	-0.06022967	-0.02913306	-3.61067725	0.000306391	0.00380259	-0.55257833	ns
REG3A	-0.04773765	0.007334635	-3.60985568	0.000307333	0.00380259	-0.56847311	ns
SELENOP	0.016236221	0.003889723	3.608611792	0.000308809	0.003804741	-0.57223074	ns
GDF2	-0.02854649	0.019448341	-3.60320893	0.000315309	0.003868498	-0.58606073	ns
DPY30	-0.04759107	-0.00910265	-3.5953172	0.000325005	0.003970771	-0.6197064	ns
CD300C	-0.02621327	-0.02114561	-3.58684554	0.000335732	0.004054294	-0.65100622	ns
REG4	-0.03650917	-0.02641816	-3.58672835	0.000335893	0.004054294	-0.64600991	ns
SERPINA1	-0.00575362	-0.00429912	-3.58664906	0.000336007	0.004054294	-0.64240613	ns
SUSD5	-0.02479779	-0.0123051	-3.58152462	0.000342672	0.00411771	-0.65487641	ns
ART3	-0.02587308	0.031598704	-3.56361471	0.000366892	0.004375141	-0.72804826	ns
PCDH12	-0.0180202	0.000365436	-3.56346276	0.000367092	0.004375141	-0.73389263	ns
ADAMTSL2	-0.02286667	-0.00973417	-3.56214725	0.000368938	0.004379269	-0.73719019	ns
OSCAR	-0.02609614	-0.0420941	-3.55917453	0.000373145	0.004410797	-0.74362175	ns
AIFM1	-0.08246932	-0.00148481	-3.55814482	0.000374616	0.004410797	-0.74477067	ns
CLEC7A	-0.04559407	-0.12431534	-3.54728048	0.000390377	0.004577914	-0.79091496	ns
FABP4	-0.04589486	-0.0562609	-3.54560612	0.000392865	0.004588664	-0.79640818	ns
GP1BA	-0.02509959	0.007094555	-3.5384812	0.000403636	0.004685031	-0.81266336	ns
DDAH1	-0.03442453	0.0037605	-3.53731931	0.000405407	0.004685031	-0.81889288	ns
IL15	-0.02516408	-0.02098871	-3.53618505	0.000407147	0.004685031	-0.82535347	ns
PALM	-0.02130093	-0.01213568	-3.53582096	0.000407706	0.004685031	-0.82623398	ns
CYTL1	-0.01661622	-0.0130558	-3.53389533	0.000410677	0.004685031	-0.83658552	ns
GPC1	-0.02220013	-0.00822718	-3.53385115	0.000410742	0.004685031	-0.83868178	ns
KYNU	-0.03501509	-0.01720816	-3.50047622	0.00046573	0.005291562	-0.95446619	ns
CALCB	-0.02752531	0.028331241	-3.49848777	0.000469217	0.005310513	-0.96050031	ns
ARSA	-0.03442366	0.019352267	-3.49581096	0.000473956	0.005343443	-0.96645396	ns
DDR1	-0.01670664	-0.00572732	-3.49377904	0.000477563	0.005351618	-0.97772859	ns
HMCN2	-0.04055003	-0.04793224	-3.49336034	0.000478347	0.005351618	-0.96583999	ns
EDN1	-0.02024075	-0.02383583	-3.4888947	0.000486406	0.005421013	-0.98116094	ns
KHK	-0.04649519	-0.05240327	-3.48700934	0.000489845	0.005438584	-0.98768035	ns
SLITRK6	-0.02608559	0.001927655	-3.48211991	0.000498871	0.005517816	-1.00538571	ns
LGMN	-0.02747437	-0.01921954	-3.48056336	0.000501744	0.005528655	-1.01992312	ns
TIMP1	-0.02056994	-0.01831708	-3.46513363	0.000531443	0.005833886	-1.06210738	ns
PRTN3	-0.03548896	-0.00236403	-3.46153986	0.000538604	0.005890356	-1.06867535	ns
PALM2	-0.02720149	-0.01341872	-3.46007813	0.000541472	0.005899618	-1.09301962	ns
SLC39A5	-0.04096345	-0.06035542	-3.45896872	0.00054372	0.005902091	-1.09314162	ns
PAPPA	-0.03487949	-0.02010484	-3.45281282	0.000556294	0.006016212	-1.10611045	ns
CCL18	-0.0547937	-0.03386284	-3.4512785	0.000559464	0.006028178	-1.11123926	ns
CFC1	-0.03497408	0.017080982	-3.44949531	0.000563175	0.00604585	-1.116652	ns
ACVRL1	-0.01741115	-0.02774798	-3.44709805	0.000568172	0.006077151	-1.1300376	ns
C19orf12	-0.04722912	0.073978553	-3.43846933	0.000586595	0.006246702	-1.15510273	ns
PDZD2	-0.0429218	0.09565729	-3.43768333	0.000588302	0.006246702	-1.15623613	ns
CD93	-0.02250153	-0.00281538	-3.42241053	0.000622374	0.006565956	-1.19785114	ns
CHCHD6	-0.01952046	0.005997195	-3.42161709	0.000624111	0.006565956	-1.22340303	ns
SMOC2	-0.02822734	-0.02954394	-3.42118127	0.000625115	0.006565956	-1.22312048	ns
SIGLEC1	-0.03218587	-0.0364217	-3.41235597	0.000645694	0.006757803	-1.25302144	ns
TOMM20	-0.05331078	0.041646656	-3.39389219	0.000690795	0.007204005	-1.31799623	ns
CCL21	-0.03004207	-0.03138217	-3.38318112	0.000718297	0.007464151	-1.35131985	ns
DMD	-0.03608681	0.068997858	-3.37570711	0.00073812	0.007619117	-1.3663191	ns
SEZ6L	-0.02032995	0.011829324	-3.37557625	0.000738428	0.007619117	-1.37732977	ns
MCAM	0.02448561	0.016806363	3.367113379	0.000761459	0.007790036	-1.40324433	ns
NT5C1A	-0.0219954	-0.01621346	-3.36597238	0.000764596	0.007790036	-1.41152567	ns
ESAM	-0.02227624	-0.00190025	-3.36592079	0.000764737	0.007790036	-1.41217165	ns
VSIG2	-0.03696473	0.014193269	-3.36558894	0.000765664	0.007790036	-1.41195274	ns
IL4R	-0.02558748	-0.01938261	-3.36372762	0.000770856	0.00781562	-1.41530953	ns

CHGB	-0.02954642	-0.00350925	-3.3612009	0.000777925	0.007860005	-1.42649113	ns
MSMB	-0.05166654	-0.09660219	-3.34790693	0.000816215	0.008218445	-1.46403521	ns
LECT2	-0.04468876	-0.06099332	-3.33887295	0.000843174	0.008440509	-1.5016709	ns
CES2	-0.04097254	0.022069719	-3.33859215	0.000844051	0.008440509	-1.49632272	ns
MUC13	-0.03547873	-0.03085114	-3.32992748	0.000870771	0.008674709	-1.5192887	ns
PRND	-0.03283359	0.015342246	-3.32906482	0.000873412	0.008674709	-1.53404735	ns
MAGED1	-0.03499853	0.037134512	-3.32700239	0.00087996	0.008710113	-1.52813228	ns
TNFRSF11B	-0.02084039	-0.02346092	-3.32213932	0.000895391	0.008826774	-1.55101943	ns
EPHA4	-0.01886538	0.001125959	-3.32139927	0.000897792	0.008826774	-1.54749871	ns
MPO	-0.0339828	-0.00808592	-3.31167448	0.000929532	0.009108168	-1.58794206	ns
GLB1	-0.02981803	-0.00268415	-3.31002479	0.000935038	0.009131475	-1.59017347	ns
TNFRSF10C	-0.03599759	-0.09670437	-3.30709365	0.000944859	0.00919663	-1.60212992	ns
ZBTB17	-0.02779887	0.004713698	-3.30362152	0.000956671	0.00928066	-1.60582644	ns
SPINK6	-0.04009203	-0.11993858	-3.30216436	0.000961586	0.009297451	-1.62400139	ns
STX4	-0.04513384	0.071075477	-3.2920521	0.000996846	0.00960625	-1.64931989	ns
MZB1	-0.03649287	-0.05932045	-3.29085906	0.001001066	0.00960625	-1.6559356	ns
TRIM5	-0.06245513	-0.00204847	-3.29020744	0.001003393	0.00960625	-1.65786062	ns
ERN1	-0.02521623	-0.00836406	-3.28299836	0.001029374	0.009822782	-1.68422386	ns
MMP10	-0.04337987	0.044186699	-3.28178644	0.001033829	0.009830498	-1.68269707	ns
CDCP1	-0.03507066	-0.07187559	-3.28094584	0.001036916	0.009830498	-1.68519666	ns
KRT14	-0.02006176	0.012872668	-3.26756178	0.001087291	0.010274726	-1.70945822	ns
PHLDB1	-0.03593639	-0.00647467	-3.26496436	0.001097159	0.010334529	-1.74261913	ns
AHNAK2	-0.01959433	0.007986883	-3.26368676	0.001102175	0.0103484	-1.73501841	ns
SCARF1	-0.02988703	-0.00530759	-3.26232521	0.001107411	0.010350873	-1.7526538	ns
VGF	-0.01972309	0.015262499	-3.26178286	0.001109528	0.010350873	-1.75483987	ns
DAB2	-0.08080795	-0.10293728	-3.25543466	0.001134616	0.010545956	-1.77595349	ns
GSTA1	-0.06417685	-0.00775496	-3.25468081	0.001137663	0.010545956	-1.77103688	ns
FOLR2	-0.02070355	-0.01235875	-3.25143916	0.001150675	0.010613396	-1.78825493	ns
SMAD1	-0.05909352	0.030859878	-3.25107924	0.001152208	0.010613396	-1.77571795	ns
AGR2	-0.07025817	-0.02632782	-3.24308665	0.001185012	0.010831811	-1.79986994	ns
ADGRE1	-0.03600239	0.019804601	-3.24245737	0.001187548	0.010831811	-1.81524316	ns
RTN4IP1	-0.06022112	-0.01789122	-3.24203124	0.001189315	0.010831811	-1.81853998	ns
MANSC1	-0.01865986	-0.00295025	-3.24168887	0.001190757	0.010831811	-1.8166218	ns
FGF23	-0.03874461	0.061514744	-3.24072525	0.001194777	0.010834623	-1.82275287	ns
PDLIM5	-0.07894468	-0.09499585	-3.2327546	0.001228588	0.011090195	-1.84832434	ns
IQGAP2	-0.03889883	0.000275428	-3.23230582	0.001230556	0.011090195	-1.84258762	ns
CGREF1	-0.02974631	-0.01338478	-3.23117288	0.001235439	0.011099945	-1.84663292	ns
ROR1	-0.02158932	-0.00128194	-3.21557828	0.001304523	0.01168468	-1.89647962	ns
GAPDH	-0.02269369	-0.08122497	-3.21353824	0.001313914	0.01173008	-1.88617704	ns
RBP5	-0.04105335	-0.0139115	-3.21270718	0.001317625	0.01173008	-1.90521554	ns
PCDH9	-0.02168894	-0.00992646	-3.21044376	0.00132801	0.011786592	-1.91767821	ns
GAST	-0.07682737	0.237719385	-3.20747768	0.001341765	0.01185131	-1.92813702	ns
AXL	-0.01889893	0.001915937	-3.2071286	0.001343419	0.01185131	-1.92473228	ns
UPB1	-0.06162532	-0.00720081	-3.20499999	0.001353363	0.01190307	-1.93606755	ns
SBSN	-0.02213173	0.002892931	-3.20389869	0.001358553	0.011912838	-1.93796495	ns
ESYT2	-0.05457047	0.031658512	-3.20206971	0.001367194	0.011952716	-1.9453253	ns
TCN2	-0.02092032	-0.01894231	-3.19873964	0.001383076	0.012055473	-1.95538949	ns
MEGF10	0.027980196	0.010438591	3.19398125	0.001406091	0.012219601	-1.96506649	ns
BMPER	-0.01441896	-0.01125567	-3.19141	0.001418692	0.012292521	-1.96720244	ns
SELE	-0.03692144	-0.10645912	-3.18596432	0.001445581	0.012488449	-1.99682062	ns
DEFB4A_DEFB4	-0.10237879	-0.14645893	-3.18223743	0.001464367	0.012613428	-1.99861493	ns
ADAM8	-0.01966046	-0.02241135	-3.17711197	0.001490416	0.012800044	-2.02440653	ns
TFF3	-0.03095868	0.034613405	-3.174071	0.001506152	0.012897252	-2.02649401	ns
KLRB1	-0.02539808	0.003513487	-3.17097061	0.001522258	0.012997055	-2.04448985	ns
SLAMF6	-0.02584259	-0.01333314	-3.16631498	0.001546917	0.013169086	-2.04539175	ns
CDH1	-0.02762674	0.007051752	-3.16129907	0.001573856	0.013324448	-2.05048871	ns
PENK	-0.01865328	-0.00879142	-3.16120173	0.001574293	0.013324448	-2.06234178	ns
PLIN3	-0.02945516	0.011751979	-3.15868096	0.00158793	0.013401024	-2.07630433	ns
KAZALD1	-0.031345	-0.03563132	-3.15547674	0.001605466	0.01350997	-2.08591956	ns
CLEC4A	0.024214745	0.005587358	3.151852585	0.001625503	0.01362831	-2.09979412	ns
FAS	-0.02261138	-0.00716048	-3.15124542	0.001628863	0.01362831	-2.10398034	ns
QPCT	-0.0184019	-0.03056179	-3.15014129	0.001635048	0.013640976	-2.10402466	ns
CRACR2A	-0.05991858	0.108605813	-3.14764628	0.001649122	0.013719189	-2.10382939	ns
CLIP2	-0.07917195	-0.05597876	-3.14365196	0.001671717	0.013850097	-2.12539345	ns
LIF	-0.03584907	0.014678149	-3.14252453	0.001678223	0.013850097	-2.12061991	ns
YES1	-0.0796666	-0.03419532	-3.14236518	0.001679087	0.013850097	-2.12686236	ns

TPP1	-0.03079749	-0.01642557	-3.13943305	0.001695994	0.013950147	-2.13241588	ns
F11R	-0.03935681	0.017438556	-3.13728964	0.001708396	0.014012685	-2.14556261	ns
SERPINA6	0.011059461	0.020901187	3.13601678	0.001715797	0.014033971	-2.15269138	ns
CD40	-0.03416247	0.023642807	-3.13129831	0.001743627	0.014221763	-2.16188347	ns
SLURP1	-0.02375228	0.000671023	-3.12909363	0.001756743	0.014288826	-2.17094144	ns
FLRT2	-0.01894135	-0.00756602	-3.11875096	0.001819572	0.014724724	-2.19985121	ns
HJV	-0.02885975	-0.01201012	-3.11860464	0.00182042	0.014724724	-2.20784645	ns
TXNDC5	-0.04679779	0.024704223	-3.11755454	0.001826917	0.014736455	-2.21110153	ns
IFNGR1	-0.01363069	-0.00138398	-3.11567605	0.001838606	0.01478989	-2.21332628	ns
CCL20	-0.05841337	0.014891783	-3.1089319	0.001881043	0.015089688	-2.23768898	ns
MYH9	-0.07236132	-0.01766112	-3.09851806	0.001948394	0.015553756	-2.26838682	ns
SPINK2	-0.02286969	-0.02333042	-3.09835461	0.001949546	0.015553756	-2.2573798	ns
DPP10	-0.02267034	0.009161886	-3.09498447	0.001971774	0.015688227	-2.27594489	ns
RTN4R	-0.0242095	-0.03712188	-3.09139884	0.001995688	0.015835347	-2.2922314	ns
PPP1R12A	-0.06532049	0.033457035	-3.08456963	0.00204207	0.016159472	-2.30949825	ns
MGMT	-0.08381223	-0.18246909	-3.08374945	0.002047726	0.016160433	-2.3103668	ns
DUT	-0.06443462	-0.03891855	-3.08289074	0.002053612	0.016163202	-2.31573477	ns
ADGRD1	0.023734162	-0.01126659	3.079544777	0.002076883	0.016302414	-2.31499322	ns
CPXM2	-0.01530767	-0.0228814	-3.07834286	0.002085191	0.016323746	-2.33070904	ns
LAMA4	-0.02194352	-0.00547047	-3.07529055	0.002106655	0.016447684	-2.33730686	ns
SCN4B	-0.02575121	-0.01807436	-3.07153428	0.00213341	0.016612149	-2.33852664	ns
TNFSF13B	-0.01779934	-0.01005825	-3.06964423	0.002146848	0.016672329	-2.3568046	ns
VIT	-0.02282141	-0.04245974	-3.06593794	0.00217362	0.016826845	-2.36850626	ns
HTRA2	-0.04149665	0.025624336	-3.06530033	0.00217827	0.016826845	-2.36826199	ns
EDA2R	-0.02281264	-0.05887849	-3.0603828	0.002214316	0.017060169	-2.38685347	ns
MYOM3	0.057587416	0.000596725	3.05670003	0.002241779	0.017226302	-2.38449413	ns
TRIAP1	-0.04280411	-0.0090717	-3.05267326	0.002271964	0.017372435	-2.41022543	ns
KLK8	-0.02343953	-0.02051705	-3.05215477	0.002276044	0.017372435	-2.39317558	ns
FCRL1	-0.03030044	-0.0024533	-3.05179846	0.002278645	0.017372435	-2.40543945	ns
IGFBPL1	-0.02122951	-0.00875039	-3.04778755	0.002309259	0.017559989	-2.41757042	ns
TNFRSF10A	-0.02183588	-0.04336158	-3.04042043	0.002366483	0.017948393	-2.43906948	ns
CFI	-0.01359497	-0.0294017	-3.03680952	0.00239494	0.018078786	-2.4563724	ns
MSLN	-0.03721524	0.033705889	-3.0366762	0.002396058	0.018078786	-2.44955568	ns
DECR1	-0.06405919	-0.04681159	-3.0349874	0.002409486	0.018133247	-2.45831529	ns
IL5RA	-0.03706853	-0.01403151	-3.03268895	0.002427901	0.018224864	-2.46523086	ns
MTDH	-0.05705524	-0.00682864	-3.03072236	0.002443844	0.018297495	-2.46071319	ns
FAM3D	-0.05208212	0.096593424	-3.02814153	0.002464684	0.018406337	-2.48164829	ns
RGCC	-0.04732415	-0.03088473	-3.02593036	0.002482753	0.018452326	-2.49023666	ns
MIA	-0.02388996	0.014763211	-3.02584446	0.002483481	0.018452326	-2.4874392	ns
CEACAM16	-0.04946892	-0.02144254	-3.02119659	0.002521894	0.018690177	-2.5024822	ns
ERBB4	0.015542389	0.011304172	3.014146332	0.002581219	0.019081413	-2.52247722	ns
SPINT1	-0.01571639	-0.01325497	-3.00706444	0.002642124	0.019482326	-2.54020642	ns
FOLR1	-0.01768588	0.006530722	-3.00108686	0.002694492	0.019818432	-2.55926593	ns
VCAM1	-0.01738362	0.000291885	-2.99928549	0.002710481	0.019885943	-2.56380634	ns
POLR2F	-0.02269715	-0.00156641	-2.99737908	0.00272749	0.019960575	-2.56825198	ns
FETUB	-0.02245148	-0.03306284	-2.99051666	0.002789484	0.020363232	-2.58985121	ns
HNMT	-0.03133577	-0.05734174	-2.98855588	0.002807478	0.020443478	-2.59138847	ns
PPP1R9B	-0.06332575	0.021549955	-2.9866363	0.002825052	0.020520277	-2.60737288	ns
SIGLEC10	-0.02164338	-0.01629258	-2.97914507	0.002895063	0.020976634	-2.62485293	ns
MET	0.013039194	0.019595455	2.977155394	0.002913878	0.021060706	-2.63283856	ns
PGF	-0.01557464	-0.02196675	-2.96908014	0.002991488	0.021536542	-2.65922671	ns
AK2	-0.06499514	-0.03490324	-2.96877537	0.002994464	0.021536542	-2.65923064	ns
RETN	-0.02656075	-0.01707067	-2.9660095	0.003021587	0.021678219	-2.65943375	ns
SGSH	-0.03407753	-0.01126797	-2.96463963	0.003035065	0.021721543	-2.66442369	ns
MZT1	-0.03363789	0.068944765	-2.96178871	0.003063356	0.021870414	-2.66678788	ns
NDUFS6	-0.05137861	0.046530358	-2.96049191	0.003076274	0.021909077	-2.67059569	ns
LGALS3	-0.01931521	-0.02689129	-2.95456864	0.003135823	0.022278837	-2.69638497	ns
LHB	-0.05682613	0.17978426	-2.95304958	0.003151379	0.022335017	-2.69241642	ns
CDH3	-0.0218371	0.008297827	-2.95222659	0.00315964	0.022339343	-2.70875472	ns
FNTA	-0.02138057	0.02094789	-2.94583566	0.00322564	0.02272009	-2.72564282	ns
PRDX3	-0.04608107	0.01579031	-2.94547673	0.003229492	0.02272009	-2.71540006	ns
CTSL	-0.01497333	-0.0219277	-2.94476746	0.003236835	0.02272009	-2.72369306	ns
HCLS1	-0.03649482	-0.04580436	-2.93947608	0.003292517	0.023055517	-2.74597365	ns
ERBIN	-0.06725031	-0.05398046	-2.93677837	0.003321352	0.023201786	-2.74657199	ns
TREML2	-0.02146718	-0.00246043	-2.93549213	0.003335122	0.023242375	-2.75324548	ns
MPHOSPH8	-0.04737414	0.034927246	-2.93317914	0.003360156	0.023308944	-2.75126605	ns

PILRA	-0.02647486	-0.07638171	-2.93312527	0.003360639	0.023308944	-2.7601336	ns
OTUD7B	-0.03842716	0.023676332	-2.93095769	0.003384114	0.023416145	-2.77080292	ns
LILRB4	-0.02774435	-0.03482651	-2.92950505	0.0034001	0.023471137	-2.76192437	ns
MVK	-0.05000822	-0.01441882	-2.92069927	0.003497633	0.024087474	-2.78746691	ns
NOTCH1	-0.00986107	0.001643544	-2.91754819	0.003533013	0.024219132	-2.80925742	ns
CXADR	-0.0325225	0.043997185	-2.91752426	0.003533339	0.024219132	-2.80503847	ns
TARS1	-0.02214452	0.01433718	-2.91339327	0.003580364	0.024428169	-2.81991094	ns
CALCA	-0.03877282	0.02962224	-2.91338185	0.003580567	0.024428169	-2.81290301	ns
NOMO1	-0.01558163	-0.02945913	-2.90957187	0.003624431	0.024669784	-2.82837693	ns
PDZK1	-0.02611745	0.020532367	-2.90802794	0.003642479	0.024734971	-2.82197373	ns
ADH4	-0.05660315	-0.03484316	-2.90049199	0.003731161	0.025278401	-2.84581243	ns
SCG2	-0.01950309	0.003569741	-2.89790157	0.003761965	0.0254281	-2.86494781	ns
PCSK9	-0.02520933	-0.02306852	-2.89708747	0.003771715	0.025435121	-2.86825977	ns
CD84	-0.01991073	-0.00421392	-2.89262249	0.003825773	0.025721531	-2.87678658	ns
NRCAM	-0.01733213	0.002249471	-2.89212846	0.003831803	0.025721531	-2.87605072	ns
NRN1	-0.02106783	0.02868848	-2.88061233	0.003974468	0.026591596	-2.91590792	ns
FOLR3	-0.1204716	-2.48704626	-2.88020606	0.003979633	0.026591596	-2.91323886	ns
GDNF	-0.02361603	-0.0150574	-2.87923082	0.003992077	0.026613843	-2.90671067	ns
USP47	-0.0515179	0.166035214	-2.87728159	0.004016821	0.026717808	-2.91067834	ns
MXRA8	0.018475885	0.034231663	2.87325333	0.004068329	0.026998914	-2.92385618	ns
CX3CL1	-0.02040335	0.008456181	-2.86822312	0.004133498	0.027369195	-2.94358747	ns
AMOT	-0.02392972	0.015524282	-2.86746232	0.004143491	0.027373287	-2.94033991	ns
ARSB	-0.03512877	-0.00677018	-2.86511158	0.004174301	0.027514582	-2.95275924	ns
MAN1A2	0.01513506	-0.00399554	2.861618458	0.004220639	0.027757354	-2.9560336	ns
TMSB10	-0.04855367	-0.04443116	-2.85684801	0.004284447	0.028113677	-2.97914628	ns
LAT2	-0.07967629	0.02581102	-2.85570229	0.004299956	0.028152175	-2.97945215	ns
CCL7	-0.03767558	-0.02767532	-2.85479825	0.00431219	0.028169115	-2.9845922	ns
KIAA0319	-0.01767104	0.017264363	-2.85360753	0.004328317	0.028211349	-2.99177803	ns
TDRKH	-0.05384107	0.085080033	-2.84924455	0.004388258	0.028538336	-2.99188253	ns
IMMT	-0.02522009	-0.00701378	-2.84491497	0.004448168	0.028863667	-3.01546542	ns
APOM	0.016928324	0.01448561	2.842985313	0.00447522	0.02897482	-3.01662197	ns
RNASEH2A	-0.02911894	0.083288805	-2.84225064	0.004485467	0.028976912	-3.02478784	ns
PDCD1LG2	-0.01920908	-0.01851512	-2.83907366	0.004530433	0.029202794	-3.02641087	ns
HSDL2	-0.02763695	0.00206624	-2.83676585	0.004563377	0.029350357	-3.02625264	ns
FGF21	-0.07536877	-0.12870227	-2.83001574	0.004660556	0.029756315	-3.05909741	ns
CD55	-0.01599961	-0.00149866	-2.82975337	0.004664699	0.029756315	-3.03496238	ns
CXCL1	-0.06003402	0.068295683	-2.82956644	0.004667176	0.029756315	-3.05380481	ns
SDC1	-0.02323631	-0.00568996	-2.82956087	0.004667258	0.029756315	-3.05357507	ns
AGR3	-0.03727475	0.008485074	-2.82768125	0.004694852	0.029867033	-3.05102283	ns
IL15RA	-0.02132989	-0.0277225	-2.82272077	0.004768026	0.0302666	-3.06648688	ns
EGLN1	-0.0330383	0.007027714	-2.82069686	0.004798096	0.030317632	-3.08057201	ns
SERPINH1	-0.07471981	-0.02301683	-2.82020621	0.004805466	0.030317632	-3.07838489	ns
CTBS	-0.01563908	-0.02082867	-2.82009003	0.004807214	0.030317632	-3.07809174	ns
PLXNB3	-0.02903343	0.018616671	-2.81622596	0.004865391	0.030618406	-3.08900548	ns
IDUA	-0.0282396	-0.06730344	-2.8128483	0.004916649	0.030820511	-3.10786318	ns
LTA	-0.02474044	-0.01785238	-2.81272426	0.004918616	0.030820511	-3.10284507	ns
GPR37	-0.03950649	-0.09750955	-2.81065088	0.00495052	0.030953998	-3.10022251	ns
MAMDC4	0.031222198	0.011583313	2.800623375	0.005106736	0.031816973	-3.13947513	ns
HNRNPK	-0.0464644	0.051842254	-2.80040811	0.005110329	0.031816973	-3.12660337	ns
LBP	-0.03591023	-0.06112739	-2.79533843	0.005190978	0.032151821	-3.15040882	ns
CD207	-0.02319274	-0.00969881	-2.79396206	0.005213249	0.032151821	-3.144744	ns
CREG1	-0.02337966	-0.00221972	-2.79391231	0.005213995	0.032151821	-3.14842458	ns
ALDH3A1	-0.03882507	0.035957889	-2.79376841	0.005216247	0.032151821	-3.15468828	ns
ERMAP	-0.02572681	-0.01702093	-2.79265734	0.005234308	0.032151821	-3.14806789	ns
RP2	-0.02608075	0.025341801	-2.79112306	0.005259059	0.032151821	-3.16028791	ns
EGFL7	-0.0245998	-0.00495129	-2.7906352	0.005267098	0.032151821	-3.15494836	ns
ITGBL1	-0.01934566	0.002287456	-2.79024442	0.005273268	0.032151821	-3.16865576	ns
CD99L2	-0.01490439	-0.01799669	-2.78999421	0.00527739	0.032151821	-3.16636146	ns
KCTD5	-0.02555758	-0.00222491	-2.7891295	0.005291573	0.032151821	-3.16289371	ns
TMPRSS15	-0.05607885	-0.00061091	-2.78900439	0.005293568	0.032151821	-3.16615063	ns
PTEN	-0.01803882	-0.00554402	-2.78824447	0.005306096	0.032151821	-3.16239578	ns
ATXN10	-0.04397514	-0.02471162	-2.78817382	0.005307253	0.032151821	-3.16259113	ns
MDK	-0.03113605	-0.00417166	-2.78441817	0.005368914	0.032458028	-3.18308189	ns
NRP2	-0.01742161	-0.00977445	-2.78168429	0.005414351	0.032665092	-3.18932286	ns
KIRREL1	-0.03340501	0.123520414	-2.77939988	0.005452688	0.032828552	-3.18691092	ns
GZMB	0.038701812	0.032553069	2.775039719	0.005526175	0.033202533	-3.20727197	ns

CHGA	-0.04387885	0.07489896	-2.77359369	0.005550723	0.033281541	-3.21460164	ns
RNF41	-0.04158441	0.040599887	-2.77269538	0.005566254	0.033306276	-3.20443576	ns
CXCL17	-0.02686924	-0.04873897	-2.77073524	0.005599637	0.033437502	-3.2238437	ns
XCL1	-0.03388958	0.011526021	-2.76197659	0.005752017	0.034277324	-3.24851987	ns
MANSC4	-0.03603995	0.052623327	-2.76095876	0.005770184	0.034315556	-3.23586915	ns
IL16	-0.02526546	-0.03643258	-2.75869397	0.005810076	0.034482565	-3.25686579	ns
CA5A	-0.05590418	-0.01591403	-2.75692132	0.005841862	0.034547759	-3.24855188	ns
PDLIM7	-0.07793607	0.018858306	-2.75673091	0.005845103	0.034547759	-3.25862468	ns
HPGDS	0.022277496	0.012222108	2.756059397	0.005857282	0.034547759	-3.25004157	ns
KIFBP	-0.0504088	-0.1074722	-2.75543292	0.005868386	0.034547759	-3.25845521	ns
TGFA	-0.02739596	0.008263632	-2.75112377	0.005946085	0.034934747	-3.27281511	ns
DNM3	-0.03079959	0.052783543	-2.7504553	0.005958144	0.034935304	-3.27940615	ns
TAX1BP1	-0.03649237	0.078405696	-2.74936559	0.005977991	0.034981432	-3.28144586	ns
CTSD	-0.02353671	0.007988467	-2.74582863	0.006043427	0.035251429	-3.25231008	ns
CNST	-0.06514668	-0.05921551	-2.74553536	0.006048276	0.035251429	-3.28588061	ns
CD160	-0.02704579	0.001850212	-2.74276761	0.006099361	0.035478352	-3.30032168	ns
SLMAP	-0.04734619	0.053210249	-2.73687517	0.006209668	0.035979349	-3.3143869	ns
KLK6	-0.01764248	-0.00094985	-2.73640272	0.006218612	0.035979349	-3.3135306	ns
SCLY	-0.02903695	-0.00612037	-2.73619756	0.006222456	0.035979349	-3.31752433	ns
CD274	-0.01986006	-0.00287671	-2.73474119	0.006250135	0.036067973	-3.31446295	ns
EFNB2	-0.0326082	0.027226755	-2.73209791	0.006300552	0.036287201	-3.31587138	ns
GIP	-0.02872869	0.075396273	-2.73075121	0.006326358	0.036312957	-3.31891664	ns
CPE	0.023304811	-0.03581652	2.73055835	0.006329896	0.036312957	-3.33083289	ns
DEFA1_DEFA1	-0.03034497	0.009762663	-2.72673294	0.006403953	0.036655998	-3.32360998	ns
LEO1	-0.01648894	0.00957895	-2.72576116	0.006422534	0.036655998	-3.3464765	ns
MYDGF	-0.05630572	-0.03194386	-2.72551428	0.006427353	0.036655998	-3.34626294	ns
SNAP29	-0.05424212	-0.03306719	-2.7226152	0.006484018	0.036872495	-3.35187614	ns
PLCB2	-0.05445307	-0.04359001	-2.72218842	0.006492364	0.036872495	-3.3551963	ns
P4HB	-0.0233368	-0.00640458	-2.72164107	0.006503197	0.036872495	-3.35165084	ns
SLITRK1	-0.0187371	0.037649771	-2.7159603	0.006615937	0.037439022	-3.3575552	ns
DBI	-0.04798376	-0.00851293	-2.71160681	0.006703282	0.037859927	-3.37755692	ns
SERPINA11	-0.02428162	-0.04257808	-2.70461075	0.00684591	0.038590842	-3.40299738	ns
PTGR1	0.045254233	0.000851081	2.701717072	0.006905754	0.03885318	-3.4103186	ns
OMG	0.040036645	0.052601493	2.70003263	0.006940892	0.038975781	-3.40833604	ns
SIRT2	-0.05332685	-0.02350642	-2.69926289	0.00695696	0.038991026	-3.41039709	ns
CEACAM6	-0.02148346	-0.00209125	-2.69780921	0.006987305	0.039056505	-3.42024985	ns
WASHC3	-0.04284715	0.072432157	-2.6974234	0.006995395	0.039056505	-3.42176354	ns
TNFAIP2	-0.02624822	0.023714261	-2.69520087	0.007042226	0.039242937	-3.42666081	ns
LRRC59	-0.02553754	0.06827152	-2.69253443	0.007098748	0.039411688	-3.43488574	ns
MRC1	-0.01570073	-0.02580756	-2.69163695	0.007117872	0.039411688	-3.43616178	ns
CLEC6A	-0.027408	-0.02380811	-2.69137602	0.007123662	0.039411688	-3.42475878	ns
HIP1R	-0.02347283	0.001337291	-2.69092434	0.007133091	0.039411688	-3.43804326	ns
PRKAR1A	-0.04972855	-0.03534093	-2.69060214	0.007140061	0.039411688	-3.4339729	ns
EPO	-0.04472341	0.072446807	-2.68997392	0.007153491	0.039411688	-3.43782297	ns
F2R	-0.03424212	-0.01192922	-2.68536491	0.007252837	0.039860502	-3.45012869	ns
LETM1	-0.02900721	0.027133092	-2.6849277	0.007262256	0.039860502	-3.45605615	ns
DCTN2	-0.05379088	0.080828497	-2.68384809	0.00728585	0.039914977	-3.45154797	ns
VAT1	-0.01143147	0.004499444	-2.68117631	0.007344253	0.040159587	-3.45865652	ns
SCAMP3	-0.05087791	0.02090481	-2.68042924	0.007360659	0.040174062	-3.46064293	ns
RSPO3	-0.01543669	-0.01457522	-2.6747832	0.007485597	0.040779744	-3.48363856	ns
PFDN2	-0.03246469	0.000587926	-2.67175154	0.007553763	0.041074464	-3.47778599	ns
GCNT1	-0.01998805	-0.01039401	-2.67024714	0.007587555	0.041180035	-3.48766113	ns
ROBO1	-0.01357671	-0.00732379	-2.6696341	0.007601383	0.041180035	-3.49098401	ns
KIRREL2	-0.0231672	0.000188261	-2.66811884	0.007635889	0.041248647	-3.48740492	ns
CACYBP	-0.05649954	-0.06890145	-2.66673	0.007667474	0.041248647	-3.49018862	ns
GRAP2	-0.07567037	-0.10292451	-2.66662034	0.00766987	0.041248647	-3.49768686	ns
ADA2	-0.02398594	-0.01800107	-2.6665138	0.007672375	0.041248647	-3.49394302	ns
MSRA	-0.04277759	-0.01907864	-2.66596869	0.007684679	0.041248647	-3.50326279	ns
CXCL16	-0.0136411	-0.020789	-2.66019733	0.007817535	0.041884776	-3.52030156	ns
RPL14	-0.02198494	0.018893004	-2.65933274	0.007837833	0.041916618	-3.50979737	ns
B4GALT1	-0.01533472	-0.01137044	-2.657174	0.007887998	0.042107775	-3.52593497	ns
LCN15	-0.03267952	0.050378763	-2.65545832	0.007928159	0.042244936	-3.53411285	ns
CPVL	-0.02398718	-0.04548448	-2.65472831	0.007945347	0.042259404	-3.53551025	ns
HSPB1	-0.04055764	-0.14338851	-2.65081143	0.008038267	0.042675888	-3.53214072	ns
LYN	-0.05041764	-0.06077442	-2.64992432	0.00805926	0.042701204	-3.54128058	ns
TGFB1	-0.02217923	-0.01279642	-2.64912297	0.008078275	0.042701204	-3.55012955	ns

IL12A_IL12B	-0.04439837	-0.00296878	-2.64876658	0.008086906	0.042701204	-3.54432282	ns
PGLYRP2	-0.01649809	-0.03856765	-2.64639688	0.008143772	0.042821253	-3.5495722	ns
LRIG3	-0.01497255	0.005890878	-2.64639407	0.008144	0.042821253	-3.54076091	ns
IL12B	-0.03141054	-0.02336091	-2.64598143	0.008153636	0.042821253	-3.55900235	ns
NXPH3	-0.01652886	0.00862956	-2.6445113	0.008189396	0.042925208	-3.547465	ns
TSC22D1	-0.03650478	0.051698312	-2.6439452	0.008202831	0.042925208	-3.5643743	ns
CA4	-0.01359027	-0.00525554	-2.64233054	0.008242032	0.042998149	-3.56816244	ns
F7	-0.01767765	-0.03198176	-2.64175855	0.008256061	0.042998149	-3.56339043	ns
MFAP5	-0.02395382	-0.03717505	-2.64155384	0.008260946	0.042998149	-3.57019795	ns
LGALS8	-0.03566587	0.045095938	-2.64077427	0.00827997	0.043020486	-3.57269292	ns
LCN2	-0.02122251	-0.01664785	-2.63831161	0.008340745	0.04325928	-3.55559549	ns
CLEC1B	-0.05601119	-0.0024357	-2.63737991	0.008363255	0.043261662	-3.58157901	ns
SAMD9L	-0.04272249	0.019324208	-2.63707604	0.008370835	0.043261662	-3.57757445	ns
TARBP2	-0.04663921	0.019241948	-2.63332393	0.008463999	0.04366586	-3.57887489	ns
ACP1	0.024273726	0.062168837	2.632089312	0.008494824	0.04372922	-3.57996625	ns
TNFSF8	-0.01900303	-0.00572543	-2.63162309	0.008506232	0.04372922	-3.59519612	ns
CCDC80	-0.01962514	-0.03987433	-2.62965908	0.0085556	0.043789513	-3.5947681	ns
CD101	-0.02321588	-0.06271614	-2.62908788	0.008569892	0.043789513	-3.60124356	ns
DLK1	-0.03163294	-0.01779627	-2.62888957	0.008574863	0.043789513	-3.6033031	ns
LBR	-0.03987059	0.035417087	-2.62876735	0.008577946	0.043789513	-3.6040746	ns
PMVK	-0.06401961	-0.30859416	-2.62735671	0.008613842	0.043896018	-3.59301286	ns
ACOT13	-0.06060323	-0.05739855	-2.62160197	0.008760327	0.044518551	-3.62073839	ns
RAB27B	-0.04421823	-0.14291056	-2.6213614	0.008766495	0.044518551	-3.62246737	ns
PTH	-0.04288638	-0.00385061	-2.61911698	0.008824572	0.044670662	-3.61566432	ns
CSDE1	-0.0650052	0.236191659	-2.61902324	0.008827045	0.044670662	-3.61450545	ns
PLSCR3	-0.03229731	0.021866194	-2.61682758	0.008883743	0.044870725	-3.63347096	ns
ADAM22	-0.01945575	-0.00237658	-2.61573126	0.008912419	0.044870725	-3.63024173	ns
PTPRH	-0.02564	0.034601966	-2.61571712	0.008912678	0.044870725	-3.63602475	ns
ITGB1BP2	-0.06401733	-0.09242788	-2.61500908	0.008931395	0.044887562	-3.62657087	ns
PRTFDC1	-0.06492907	-0.15489868	-2.61097466	0.0090374	0.045342282	-3.63699466	ns
CXCL6	-0.04397046	-0.01521787	-2.60968676	0.009071208	0.04543384	-3.65361376	ns
FUOM	-0.0343902	-0.00858004	-2.60556382	0.00918129	0.045899373	-3.65109033	ns
CLC	-0.03793464	0.045408347	-2.6050267	0.009195594	0.045899373	-3.658715	ns
THBD	-0.01547751	-0.01595167	-2.60414678	0.009219226	0.045938805	-3.66098819	ns
HDGFL2	-0.0344504	0.024071242	-2.60280598	0.00925523	0.045961567	-3.67049362	ns
MAVS	-0.05396981	0.080756009	-2.60281427	0.009255274	0.045961567	-3.6572511	ns
CXCL9	-0.03747535	0.030408137	-2.59905386	0.009357014	0.046332682	-3.67744676	ns
CCL16	-0.02987649	-0.12633205	-2.59824948	0.009378898	0.046332682	-3.68274064	ns
AXIN1	-0.0549955	-0.02693033	-2.59792363	0.009387896	0.046332682	-3.67922587	ns
TIMM8A	-0.046485	0.032083689	-2.5977162	0.009393475	0.046332682	-3.68362213	ns
DAPP1	-0.06967013	-0.27996901	-2.59555852	0.009452725	0.046546303	-3.68531775	ns
CD164	-0.01579279	0.011745166	-2.59359443	0.009506851	0.046677391	-3.6907372	ns
CRYBB1	-0.03475366	0.013665339	-2.59315649	0.009518891	0.046677391	-3.69541449	ns
PMM2	-0.03182921	0.041929373	-2.59257994	0.009535126	0.046677391	-3.68284159	ns
GABARAP	-0.02520487	0.031043897	-2.5920355	0.009549973	0.046677391	-3.69735472	ns
LONP1	-0.04581813	0.009066422	-2.59170841	0.009559274	0.046677391	-3.68587946	ns
SAFB2	-0.01489598	0.00595971	-2.58563137	0.009729108	0.047427372	-3.71563037	ns
SUSD1	-0.02758775	-0.0524417	-2.58118577	0.009855521	0.047963535	-3.71372311	ns
PLB1	0.031512412	-0.00954359	2.577837817	0.009951212	0.048348649	-3.73465028	ns
UBE2L6	-0.03285998	0.006833735	-2.57680109	0.0099811	0.048390167	-3.73735373	ns
CIRBP	-0.05073709	-0.07475311	-2.57638369	0.009993168	0.048390167	-3.737315	ns
METAP1D	-0.04639201	-0.01158085	-2.57582222	0.010009473	0.048390167	-3.73593695	ns
ANGPTL3	-0.01978612	0.002493272	-2.57482727	0.010038329	0.048449458	-3.73606346	ns
GPD1	-0.02920046	0.002666076	-2.57300775	0.010091139	0.048602308	-3.74609939	ns
EIF4G1	-0.05718535	-0.09664997	-2.57202661	0.010119721	0.048602308	-3.75040444	ns
MOCS2	-0.03536822	0.023097775	-2.57154519	0.010134031	0.048602308	-3.73842445	ns
GIPC2	-0.01525669	-0.00497731	-2.57138862	0.010138396	0.048602308	-3.75112134	ns
LAT	-0.04355193	-0.11615883	-2.57074961	0.010157093	0.048602308	-3.75366184	ns
FTCD	-0.0457435	0.059164144	-2.57031549	0.010169866	0.048602308	-3.75257334	ns
EIF2AK2	-0.05766888	0.030491877	-2.56831553	0.010228742	0.048787695	-3.75612438	ns
TLR2	-0.01224891	-0.00580661	-2.56786679	0.010242074	0.048787695	-3.75263061	ns
ATP5IF1	-0.06249263	-0.08554101	-2.56701019	0.010267309	0.048828243	-3.75954887	ns
SIGLEC8	-0.02362529	-0.03199942	-2.56304491	0.010385477	0.049309908	-3.75914693	ns
FGF7	0.042361508	0.183344146	2.562223328	0.010410103	0.049346594	-3.758818	ns
PLIN1	-0.02054988	0.007576662	-2.5612387	0.010439614	0.049385973	-3.76455791	ns
AGXT	-0.04344577	0.063705728	-2.56062394	0.010457943	0.049385973	-3.77246789	ns

GRP	-0.02761391	-0.0078918	-2.56025644	0.01046915	0.049385973	-3.76623115	ns
MASP1	-0.01310156	-0.01695541	-2.5595509	0.010490317	0.049406007	-3.77319639	ns
JAM3	-0.02587427	0.039494943	-2.55635672	0.01058713	0.049781675	-3.77704667	ns
APOA1	0.014229547	0.031703827	2.552670292	0.010699566	0.050229473	-3.79845313	ns
MCFD2	-0.0245519	0.003837824	-2.55070645	0.01076014	0.05043276	-3.79702088	ns
SPTLC1	-0.01948678	0.044138998	-2.54764187	0.010854944	0.05079557	-3.81141447	ns
CD70	-0.02419691	-0.03548506	-2.54555199	0.010920208	0.051019211	-3.81282325	ns
MANF	-0.06776578	-0.25297915	-2.54472895	0.010945922	0.051033105	-3.81603791	ns
CCL23	-0.02293171	-0.02345672	-2.54403726	0.010967613	0.051033105	-3.81778367	ns
CRISP3	0.014085094	0.008000267	2.543784004	0.010975613	0.051033105	-3.81585977	ns
ITGA11	0.018996313	0.015084387	2.543073476	0.010997944	0.051055637	-3.81907845	ns
FURIN	-0.02039786	-0.04240017	-2.53937573	0.011114775	0.051441096	-3.83008565	ns
MAMDC2	-0.01713792	-0.00412573	-2.53932954	0.011116209	0.051441096	-3.8324404	ns
SNX5	-0.01539221	0.045764639	-2.53744506	0.011176475	0.05156188	-3.82389509	ns
IKBKG	-0.05130589	-0.00835634	-2.53740354	0.011177627	0.05156188	-3.83336516	ns
PALM3	-0.0394031	0.152686517	-2.53195921	0.011352815	0.052287412	-3.836886	ns
BAX	-0.04953215	0.023373501	-2.53139851	0.011370966	0.052288534	-3.83925821	ns
IRAG2	-0.04933935	0.145537998	-2.53029978	0.011406609	0.052323194	-3.84270501	ns
NOS3	-0.0289273	-0.03188146	-2.52952271	0.011431886	0.052323194	-3.84462346	ns
SKAP2	-0.05872158	-0.01939159	-2.52950425	0.01143226	0.052323194	-3.85435372	ns
ARG1	0.02953211	0.014040779	2.52579834	0.011553577	0.05279569	-3.86083069	ns
APOA4	-0.01793385	0.017433054	-2.51792433	0.011814864	0.053905315	-3.88583892	ns
SRC	-0.06729486	-0.05705954	-2.51707297	0.011843498	0.053951658	-3.88498753	ns
ST6GAL1	-0.01600018	-0.02674019	-2.51562302	0.011892238	0.054089306	-3.89232018	ns
BNIP2	-0.01758294	0.009900216	-2.51380456	0.011953719	0.054284385	-3.89641495	ns
CDH17	-0.03751503	0.068689641	-2.51203117	0.012014051	0.054473649	-3.89471574	ns
ALPP	-0.06185827	-0.01356746	-2.51082713	0.012055094	0.054574998	-3.89728538	ns
BRAP	-0.05646525	-0.07880488	-2.50938126	0.01210452	0.054713928	-3.9018532	ns
RGMA	-0.01694251	0.024678316	-2.50707266	0.012183712	0.05494212	-3.91407659	ns
ECHS1	-0.04929098	-0.02536621	-2.50680229	0.012193319	0.05494212	-3.90054411	ns
SERPINB6	-0.0332236	0.008138884	-2.50626961	0.012211451	0.05494212	-3.91440852	ns
DLG4	-0.03702984	0.079126732	-2.50409082	0.012286843	0.055196277	-3.92053835	ns
IL12RB1	-0.02008735	-0.02995799	-2.50228163	0.012350056	0.055395027	-3.9126072	ns
GCHFR	-0.02662146	0.002623943	-2.50173928	0.012369214	0.055395865	-3.90266683	ns
DYNLT3	-0.02241746	0.022529993	-2.50115091	0.012389308	0.055400886	-3.92694354	ns
EIF2S2	-0.02173739	0.058394612	-2.49874404	0.012473985	0.055694245	-3.9205463	ns
PKD2	-0.03067995	0.05123548	-2.49808475	0.012497231	0.055702634	-3.92137908	ns
CDSN	-0.02319409	-0.00954196	-2.49759955	0.012514016	0.055702634	-3.9369016	ns
PTPN6	-0.04926914	-0.16876903	-2.4958709	0.012575221	0.055889871	-3.93706423	ns
AZI2	-0.02887905	0.067031324	-2.49416257	0.012635789	0.056073716	-3.9460952	ns
DTNB	-0.02213839	-0.00528024	-2.49242046	0.012697992	0.056264243	-3.94727737	ns
C3	-0.02457475	-0.00887647	-2.48906366	0.012819022	0.056586403	-3.92978881	ns
MTUS1	-0.02812731	0.001213783	-2.48881599	0.012827499	0.056586403	-3.95087312	ns
MOG	-0.01780278	0.006724024	-2.48877416	0.012828835	0.056586403	-3.96003302	ns
SIT1	-0.03073418	-0.022559	-2.48670631	0.012903894	0.056743195	-3.95114102	ns
ITGAV	0.008805179	-0.00060153	2.486582641	0.012908092	0.056743195	-3.9654414	ns
MRPL58	-0.03729019	0.125381575	-2.48618052	0.01292268	0.056743195	-3.96581336	ns
CNPY4	-0.04168186	-0.02078161	-2.48541077	0.012950802	0.056781294	-3.96026398	ns
MAP2	-0.02313945	0.062906109	-2.47945739	0.013168793	0.057650488	-3.98142878	ns
PRG3	-0.02495438	0.011750375	-2.4767153	0.013270362	0.05800817	-3.98768365	ns
NEFL	-0.02235995	-0.03065688	-2.47443213	0.01335578	0.058294286	-3.97975063	ns
CACNB3	-0.04269223	-0.01162826	-2.47372237	0.013381972	0.058321431	-3.99646268	ns
SLIT2	-0.02824827	-0.00611232	-2.47211356	0.013442645	0.058498545	-3.98701508	ns
PRKAB1	-0.0295686	0.058418285	-2.47141972	0.01346872	0.058524795	-3.98872674	ns
CCL25	-0.0267963	-0.04666595	-2.46922111	0.013551428	0.058796685	-4.00746922	ns
DOK2	-0.06675405	-0.04117119	-2.46802318	0.013597002	0.058906892	-4.00341001	ns
LRRC25	-0.02104003	-0.0111443	-2.46548927	0.013693521	0.059237159	-4.00917942	ns
TRDMT1	-0.04302297	0.072068488	-2.4644595	0.013732939	0.059319795	-4.01070595	ns
TPK1	-0.01354357	-0.00937659	-2.4630469	0.013786986	0.059465285	-4.02171249	ns
GP2	0.046196056	0.113621163	2.462054822	0.013825627	0.059478184	-4.00544385	ns
IL17RA	-0.02410745	-0.04241618	-2.46191302	0.013830715	0.059478184	-4.02091165	ns
RAB10	-0.02709258	0.033676488	-2.45966784	0.013917411	0.059763002	-4.02996318	ns
BIRC2	-0.03491719	0.039239979	-2.45818609	0.013975269	0.059923328	-4.02022016	ns
VPS53	-0.04259111	0.062721379	-2.45634442	0.014046887	0.060142095	-4.03150206	ns
KLK13	-0.02339833	-0.01980778	-2.4524334	0.014200692	0.060711598	-4.03176599	ns
SCARF2	-0.01487999	-0.01020922	-2.45109955	0.014253015	0.060846203	-4.05173259	ns

CTRB1	-0.02884421	0.01138384	-2.44667934	0.014429028	0.06150768	-4.05799905	ns
EPS8L2	-0.01582938	-0.01011755	-2.44497808	0.014497158	0.061708019	-4.06612773	ns
SPRR3	-0.03317118	0.035261931	-2.44352968	0.014555449	0.061791873	-4.06915878	ns
KIR2DS4	-0.08609234	0.940693841	-2.44343778	0.014559181	0.061791873	-4.06832291	ns
NCR3LG1	-0.01575107	-0.00379248	-2.43159247	0.015043889	0.063756395	-4.09696529	ns
CD7	-0.02109842	-0.00790243	-2.42499354	0.015320383	0.064834085	-4.09838675	ns
APOBR	-0.02118248	-0.06262169	-2.42340596	0.015387425	0.064967099	-4.10443727	ns
SNAP23	-0.04686988	-0.11194088	-2.42296026	0.015406133	0.064967099	-4.1127207	ns
ABL1	-0.0371151	0.054459085	-2.42266736	0.015418562	0.064967099	-4.1129933	ns
PRELP	-0.01188614	-0.02761078	-2.42133485	0.015475161	0.065111627	-4.11657692	ns
PPP3R1	-0.0223507	-0.02268153	-2.41999418	0.015532351	0.065258225	-4.11908092	ns
ANXA11	-0.03571244	-0.13165385	-2.41931389	0.015561366	0.065286191	-4.12367682	ns
DBNL	-0.04980151	-0.04412986	-2.41683062	0.015667835	0.065567333	-4.13077647	ns
LYPD8	-0.02637364	-0.00238495	-2.41642951	0.015685387	0.065567333	-4.12115923	ns
GADD45B	-0.02474964	0.073312096	-2.4161851	0.015695742	0.065567333	-4.12755668	ns
IGSF9	-0.04359217	-0.0458287	-2.41557722	0.015721789	0.065582322	-4.13658256	ns
CDKN2D	-0.05049403	-0.14583199	-2.41477907	0.01575641	0.065632976	-4.13191877	ns
DNAJC21	-0.01936726	0.045629235	-2.41417219	0.015782517	0.065648078	-4.13989535	ns
LIPF	0.029015053	0.015005395	2.412346442	0.015862073	0.06588514	-4.13095874	ns
QDPR	-0.02297143	-0.01044036	-2.4083063	0.016038534	0.066458756	-4.14523267	ns
NFKBIE	-0.02643716	0.023023827	-2.40814664	0.016045693	0.066458756	-4.14097622	ns
USP28	0.018426382	0.036867591	2.407487795	0.016074309	0.066482978	-4.15588193	ns
PRSS2	-0.02685719	0.007863912	-2.40687008	0.016101624	0.066501756	-4.15266215	ns
LRPAP1	-0.03047241	0.08690824	-2.40105447	0.016359603	0.06747181	-4.17217998	ns
ATOX1	-0.03813824	-0.06073943	-2.40009323	0.016402665	0.067488734	-4.17131729	ns
ERBB2	-0.01286037	-0.025448	-2.39961558	0.016424073	0.067488734	-4.17245441	ns
DPT	-0.01459934	-0.06173397	-2.39940488	0.016433468	0.067488734	-4.175651	ns
LYVE1	-0.00944962	0.000175936	-2.39889978	0.016456157	0.067488734	-4.17611392	ns
PHYKPL	-0.02055893	-0.02113701	-2.39483097	0.016640104	0.068147411	-4.17364175	ns
CDC42BPB	-0.0514325	0.060279564	-2.39426116	0.016665721	0.068156731	-4.18632383	ns
ITGB1BP1	-0.02138643	-0.01081812	-2.39023235	0.016849946	0.068813764	-4.18360407	ns
TYMP	-0.03495877	-0.02481184	-2.38801988	0.016951559	0.069132057	-4.19348054	ns
VASN	-0.01433321	-0.01119955	-2.38714037	0.01699234	0.069201718	-4.18719097	ns
CEP20	-0.0386818	0.010310323	-2.38561019	0.017063114	0.069393164	-4.19455109	ns
CSTB	-0.02271343	-0.05140729	-2.38432185	0.01712287	0.069445702	-4.1994857	ns
OPHN1	-0.0537228	-0.01509244	-2.3843001	0.017123598	0.069445702	-4.21099492	ns
NCK2	-0.05546693	-0.03250535	-2.37561014	0.017532116	0.071003853	-4.22875896	ns
CLEC4D	-0.0321589	-0.05301058	-2.37498087	0.017562078	0.071026687	-4.22909652	ns
CKB	-0.03105151	0.014531382	-2.3710982	0.017747486	0.071677259	-4.24164728	ns
PPIB	-0.04972838	-0.09391115	-2.37017591	0.017791947	0.071716862	-4.23876178	ns
WWP2	-0.0285324	0.033169945	-2.36987589	0.017806413	0.071716862	-4.23814161	ns
CAPS	-0.04529717	0.183919936	-2.36673001	0.017958239	0.072228728	-4.25240094	ns
KLRK1	-0.02181494	-0.05432116	-2.36367588	0.018106901	0.072726478	-4.25956713	ns
ERC2	0.035717625	0.145471502	2.361050615	0.018235861	0.072954657	-4.25299759	ns
YARS1	-0.07124472	-0.18103958	-2.36103653	0.018236269	0.072954657	-4.26469051	ns
CD99	-0.0098226	-0.0158305	-2.36098935	0.018238664	0.072954657	-4.26233102	ns
DNMBP	-0.032789	0.06235885	-2.35881764	0.018345786	0.073282757	-4.26407625	ns
CRELD1	-0.01829526	-0.04364029	-2.35636871	0.018467255	0.073667192	-4.26426686	ns
ENTPD6	-0.01455412	-0.04582874	-2.35513801	0.018528578	0.073735207	-4.26707274	ns
CHP1	-0.0359119	0.05134312	-2.35501282	0.018534809	0.073735207	-4.26743359	ns
NFU1	-0.04923953	-0.04513239	-2.35433439	0.018568345	0.0737639	-4.28036617	ns
DIABLO	-0.05699398	-0.02743327	-2.35385366	0.018592545	0.0737639	-4.27439604	ns
FOXJ3	-0.02144174	0.039407395	-2.35137934	0.018716789	0.074156072	-4.27365659	ns
MATN3	0.016227875	0.008724201	2.348155527	0.018879352	0.074698791	-4.287975	ns
FCRL5	-0.02949494	-0.05477698	-2.34380049	0.019101049	0.075326861	-4.30244226	ns
BPIFB1	-0.03347264	-0.01802848	-2.34354122	0.019114421	0.075326861	-4.2987758	ns
PXN	-0.0276231	-0.0001078	-2.34350474	0.019116447	0.075326861	-4.29414855	ns
COMT	-0.04335582	-0.0497991	-2.34288535	0.019148314	0.075326861	-4.29197074	ns
SERPINB8	-0.02903305	-0.01466746	-2.34251005	0.019167075	0.075326861	-4.30989579	ns
NEXN	-0.03349127	0.069892856	-2.34053381	0.019268824	0.075624539	-4.31248352	ns
ADAM23	-0.01787243	-0.03862747	-2.34003325	0.019294617	0.075624539	-4.31564868	ns
EHBP1	-0.03649621	0.041242695	-2.33907055	0.019344427	0.075718131	-4.3170169	ns
FGD3	-0.0387081	0.024898097	-2.3382437	0.019387238	0.075743764	-4.31983113	ns
RAB11FIP3	-0.05227551	-0.04093516	-2.33794323	0.019402855	0.075743764	-4.31963221	ns
EXTL1	-0.02322532	0.074238247	-2.33642735	0.019482049	0.075951378	-4.30893922	ns
ITIH3	-0.01964105	-0.04103274	-2.33537212	0.019536802	0.076018906	-4.32083084	ns

ITGA5	-0.01287706	-0.02706417	-2.33509161	0.019551438	0.076018906	-4.32265317	ns	
KITLG	-0.01869074	-0.01029371	-2.33206252	0.019710302	0.076502237	-4.32708983	ns	
STX8	-0.02515698	0.031013476	-2.33172294	0.019728145	0.076502237	-4.33008188	ns	
ZBP1	-0.01910346	0.042093612	-2.3305054	0.019792613	0.076587687	-4.322121	ns	
MED18	-0.03940475	-0.01869304	-2.33031547	0.019802638	0.076587687	-4.32386808	ns	
DRAXIN	-0.02161627	0.000665371	-2.32873171	0.019886265	0.076809384	-4.33402253	ns	
COMMD1	-0.02963623	0.020862042	-2.3275736	0.019947922	0.07691981	-4.33022399	ns	
GCC1	-0.05204427	-0.0693852	-2.32719804	0.01996754	0.07691981	-4.34444381	ns	
NMI	-0.03428628	0.040922428	-2.32623685	0.020018842	0.077015834	-4.34345512	ns	
SPACA5_SPAC	-0.0137824	0.002076908	-2.32361284	0.020159101	0.077453389	-4.35366434	ns	
REEP4	-0.03035195	0.104006957	-2.32005422	0.020350845	0.078082674	-4.36186138	ns	
LGALS7_LGALS	-0.02080527	-0.00016966	-2.31958585	0.020376369	0.078082674	-4.35649513	ns	
SLAMF1	-0.03015869	-0.07847509	-2.31506337	0.020622798	0.078923421	-4.35986765	ns	
DKKL1	0.04426574	-0.12792802	2.312797403	0.020746981	0.07926406	-4.37103877	ns	
LDLR	-0.02831901	-0.04661866	-2.31217728	0.020781026	0.07926406	-4.37664927	ns	
PAM	-0.01412385	-0.0376591	-2.31195545	0.020793243	0.07926406	-4.37709801	ns	
APOC1	0.017412116	0.016069799	2.311022095	0.020844725	0.079356712	-4.37985271	ns	
LMNB1	-0.02808283	-0.01662526	-2.31022401	0.020888887	0.07942129	-4.38104484	ns	
ACAA1	-0.0414979	0.00510804	-2.3092002	0.020945956	0.079534708	-4.37245054	ns	
BST2	-0.02551544	0.015131446	-2.3085644	0.020980852	0.079563749	-4.3882487	ns	
CYB5R2	-0.02609497	0.014019797	-2.30579096	0.021135629	0.079940217	-4.38667945	ns	
HPCAL1	-0.03761133	-0.10820676	-2.30507241	0.021175636	0.079940217	-4.39620371	ns	
PON1	0.012065661	-0.00682404	2.305008338	0.02117927	0.079940217	-4.39434452	ns	
NCR1	-0.01788619	0.008840206	-2.30482545	0.021189633	0.079940217	-4.39193861	ns	
CBS	-0.0235271	0.053951592	-2.30391501	0.021240946	0.080030401	-4.38456413	ns	
MTHFSD	-0.05319677	0.005947298	-2.30059907	0.021427717	0.080606383	-4.39795248	ns	
PDGFC	-0.01460405	-0.00982866	-2.30009687	0.021456354	0.080606383	-4.39325934	ns	
NPY	-0.03306885	-0.09276635	-2.29935594	0.021497949	0.080606383	-4.40991404	ns	
SLA2	-0.04917709	-0.03263214	-2.29924511	0.021504237	0.080606383	-4.40954254	ns	
AIDA	-0.04136461	0.179887789	-2.29464392	0.021767179	0.081283997	-4.40632178	ns	
DNAJA2	-0.04193438	-0.04836276	-2.29406092	0.021800337	0.081283997	-4.41930155	ns	
CCDC50	-0.03633902	0.154310522	-2.29380127	0.02181521	0.081283997	-4.42194549	ns	
SRPK2	-0.0420609	0.120764505	-2.29365763	0.021823624	0.081283997	-4.41741079	ns	
ELAC1	-0.04024789	-0.03533095	-2.29364497	0.021824196	0.081283997	-4.42230117	ns	
MMP7	-0.02231399	-0.07096548	-2.29269362	0.021879379	0.081368022	-4.41022784	ns	
GRHPR	-0.03002489	-0.00937699	-2.2920834	0.02191418	0.081368022	-4.42495581	ns	
MARS1	-0.04036553	-0.01219094	-2.29180897	0.021930354	0.081368022	-4.41208226	ns	
WASF1	-0.04735381	0.084448294	-2.29053349	0.022004157	0.081511593	-4.4159155	ns	
ATXN2L	-0.02241589	0.064236122	-2.29016884	0.022024879	0.081511593	-4.43020508	ns	
RNASE10	-0.02252724	-0.00605866	-2.28954727	0.022061326	0.081543131	-4.41729385	ns	
CES3	-0.03648547	0.105642	-2.28814991	0.022142368	0.081739208	-4.42728376	ns	
PILRB	-0.02869694	-0.12970868	-2.28744314	0.022183548	0.081787827	-4.42932132	ns	
MCTS1	0.012041561	0.037621962	2.285375958	0.022304264	0.082105265	-4.43906707	ns	
NELL2	-0.01418363	0.012721347	-2.28453105	0.022353866	0.082105265	-4.43928472	ns	
LSM1	-0.02744268	0.064676575	-2.28453445	0.022354002	0.082105265	-4.4286424	ns	
STK11	-0.03631356	-0.00298081	-2.28305553	0.02244096	0.082321109	-4.43287688	ns	
PIKFYVE	-0.02606889	0.065213124	-2.2805889	0.022586714	0.082751824	-4.43626437	ns	
ARHGEF12	-0.05230163	-0.03255201	-2.27794991	0.02274325	0.083220916	-4.45302856	ns	
SERPINB1	-0.04545266	0.019040598	-2.27619545	0.022848076	0.083403247	-4.45525518	ns	
INPPL1	-0.0491917	-0.01308788	-2.27615978	0.022850205	0.083403247	-4.45442721	ns	
DBN1	-0.02459325	0.046510328	-2.27473432	0.022935891	0.08350588	-4.44949015	ns	
ERP29	-0.05534654	-0.04564359	-2.27453034	0.02294804	0.08350588	-4.45221617	ns	
SHMT1	-0.04336319	-0.02802833	-2.2742592	0.022964117	0.08350588	-4.46138194	ns	
MTIF3	-0.04035894	-0.00924784	-2.27195726	0.023102765	0.083905564	-4.46940094	ns	
USP8	-0.0476259	0.046545447	-2.27050796	0.023190836	0.084120796	-4.46121255	ns	
MAD1L1	-0.02442231	9.3353285535	-2.26995507	0.023224248	0.084137475	-4.46609164	ns	
CMIP	-0.03873421	0.014338264	-2.26929025	0.023264405	0.084157502	-4.47557255	ns	
L3HYPDH	-0.04139657	0.011406265	-2.26891127	0.023287418	0.084157502	-4.47738099	ns	
GZMH	0.036172235	-0.03104039	2.266013525	0.023464679	0.084693281	-4.4713242	ns	
CTSS	-0.01234682	-0.01793221	-2.26526736	0.023514101	0.084766824	-4.36295738	ns	
NUDC	-0.03197031	-0.0570737	-2.26472769	0.023543115	0.084766824	-4.487658	ns	
CEP85	-0.03174884	0.061124103	-2.26360215	0.023612779	0.084902936	-4.47585597	ns	
EEF1D	-0.0434673	0.01657469	-2.2629186	0.023654493	0.084902936	-4.4908523	ns	
BIN2	-0.04590434	0.032309115	-2.26269898	0.023668147	0.084902936	-4.48774641	ns	
PRKG1	-0.05922813	-0.03720489	-2.26197148	0.023713278	0.084958115	-4.48047123	ns	
CCN4	-0.01976244	0.020350445	-2.26069573	0.023791896	0.084958115	-4.49736394	ns	

TPD52L2	-0.05022558	-0.09761639	-2.260245	0.023820188	0.084958115	-4.48434324	ns
CRKL	-0.05355639	-0.08170865	-2.2599453	0.023838592	0.084958115	-4.49294284	ns
SETMAR	-0.01561733	-0.01864916	-2.25973484	0.023851796	0.084958115	-4.48932161	ns
FLT3LG	-0.01638668	-0.01131521	-2.25962869	0.023858101	0.084958115	-4.49910166	ns
COX6B1	-0.01485888	0.015733357	-2.25884107	0.023907119	0.085016082	-4.49999809	ns
MESD	-0.06070335	-0.03171814	-2.25843344	0.023932609	0.085016082	-4.49731201	ns
DTD1	-0.04026016	0.014357312	-2.25367148	0.024231081	0.085971759	-4.49905883	ns
TNIP1	-0.04172642	0.017423636	-2.25096282	0.024401845	0.086472557	-4.51756875	ns
CLPP	-0.04731027	-0.01695133	-2.24927055	0.02450951	0.086748812	-4.51319374	ns
NARS1	-0.0219139	-0.05037414	-2.24662461	0.024678024	0.087239504	-4.52819965	ns
MAP2K6	-0.05010988	-0.04439432	-2.24496438	0.024784604	0.08751033	-4.52702822	ns
FCRL6	-0.03349972	-0.06208882	-2.24324771	0.024895056	0.087794157	-4.53084986	ns
GLRX5	-0.04020015	0.00724146	-2.24061629	0.025065377	0.08828818	-4.5281574	ns
CD300A	-0.01344578	-0.01863664	-2.23906885	0.025165897	0.088535444	-4.53159527	ns
TPSD1	-0.01682982	0.031378876	-2.23803895	0.025232639	0.088663424	-4.54729948	ns
BANK1	-0.05266446	-0.09860749	-2.23679832	0.025313831	0.08884181	-4.54518135	ns
METAP2	-0.02872143	-0.05762615	-2.23543866	0.025402748	0.089046849	-4.55295678	ns
F9	-0.00931301	-0.02595323	-2.2337806	0.025511879	0.089322167	-4.55048935	ns
CASP3	-0.04320947	-0.05198916	-2.23324758	0.025546827	0.089337408	-4.55747386	ns
TJAP1	-0.04114985	0.026097169	-2.23149969	0.025662348	0.089634038	-4.55863255	ns
CEACAM5	-0.03108118	-0.01066302	-2.22799843	0.025895533	0.090240538	-4.54910473	ns
HHEX	-0.03559256	-0.03271949	-2.22795516	0.025897798	0.090240538	-4.56868569	ns
TBCB	-0.04976689	-0.11186307	-2.2274205	0.0259337	0.09025793	-4.56296151	ns
DDX58	-0.02846468	0.063343732	-2.22541262	0.026068318	0.090618438	-4.56087487	ns
DOK1	-0.06236577	-0.15897007	-2.22292026	0.026235545	0.09109131	-4.57980193	ns
PHACTR2	-0.05230016	-0.03393942	-2.22175599	0.026314164	0.091255769	-4.58332211	ns
APOA2	-0.02181993	-0.00634954	-2.22030128	0.026412748	0.091488998	-4.5844858	ns
STX3	-0.01754612	0.064155683	-2.21762397	0.026594927	0.091902173	-4.59146812	ns
REN	-0.03332902	-0.01075304	-2.21717388	0.026625845	0.091902173	-4.58632014	ns
PRR4	-0.03194812	0.058512286	-2.21716883	0.026626451	0.091902173	-4.57655791	ns
USO1	-0.04436503	-0.04736892	-2.21599047	0.026706815	0.091975485	-4.58848749	ns
PRKAR2A	-0.03766773	-0.04797224	-2.21561366	0.026732481	0.091975485	-4.59500728	ns
HARS1	-0.03792808	-0.01981114	-2.21540516	0.026746869	0.091975485	-4.59279547	ns
MARCO	-0.0129319	-0.03160433	-2.21501521	0.026773686	0.091975485	-4.59106564	ns
CELSR2	-0.01264733	-0.00448069	-2.21344512	0.02688182	0.092082787	-4.58817723	ns
DRG2	-0.02783831	0.044038546	-2.21321184	0.026897746	0.092082787	-4.59459171	ns
TGFBR1	-0.00998067	0.001379211	-2.21318974	0.026899527	0.092082787	-4.58646667	ns
CD48	-0.01408086	-0.02350429	-2.21167482	0.027003786	0.092331445	-4.60063651	ns
ENO1	-0.03839934	-0.04632522	-2.21073987	0.027068576	0.092438224	-4.60211776	ns
GSTP1	-0.03260389	0.065066671	-2.2103149	0.027098329	0.092438224	-4.59373589	ns
DSCAM	-0.01568465	0.00866266	-2.20805379	0.027255087	0.092864475	-4.61343167	ns
SCARA5	-0.01370587	-0.04072241	-2.20268596	0.027631548	0.094037437	-4.62518324	ns
GOPC	-0.04932441	-0.06497915	-2.20106646	0.027746157	0.094317552	-4.62383576	ns
ELOB	-0.01760001	0.021645314	-2.20042308	0.027792046	0.094363693	-4.61493693	ns
DCTD	-0.04281196	-0.05431009	-2.19905493	0.027889079	0.094583172	-4.61966806	ns
SCRIB	-0.04154527	0.181398674	-2.19859226	0.027922064	0.094585182	-4.61974191	ns
MAP3K5	-0.04848576	0.119041837	-2.19698123	0.028036983	0.094761988	-4.62323115	ns
BACH1	-0.03495134	0.030804346	-2.19692561	0.028040657	0.094761988	-4.63286892	ns
PLEKHO1	-0.031728	0.046256373	-2.19649039	0.028071616	0.094761988	-4.63774243	ns
SIAE	-0.02264575	-0.00954337	-2.19243827	0.028362779	0.095634314	-4.64355066	ns
SH2B3	-0.04717299	-0.04187252	-2.1869188	0.028763799	0.096874618	-4.64510828	ns
TK1	-0.0202899	0.034499433	-2.18565012	0.028856352	0.097074365	-4.65372401	ns
PTPRR	-0.01621054	0.016323987	-2.18423496	0.02895992	0.097310663	-4.66437422	ns
MINK1	-0.04177796	-0.04931503	-2.18366718	0.029001653	0.097338883	-4.66560441	ns
PRDX5	-0.03297933	-0.12997699	-2.18218098	0.02911111	0.097558525	-4.66974793	ns
SIRPA	-0.02746109	-0.09106742	-2.1818746	0.029133916	0.097558525	-4.66456801	ns
CD200R1	-0.01672313	0.016221367	-2.17927601	0.029326382	0.098090534	-4.66950861	ns
CCL4	-0.03179441	0.017137481	-2.17260912	0.029824995	0.099572041	-4.68467742	ns
PON2	-0.01671233	-0.03382266	-2.17199821	0.029871342	0.099572041	-4.67825114	ns
UFD1	-0.06252146	-0.07061145	-2.17198934	0.029871612	0.099572041	-4.68976623	ns
RRAS	-0.05808956	0.130325547	-2.16913875	0.030087687	0.100177933	-4.68415971	ns
GPR15L	-0.02683306	-0.06430246	-2.16615596	0.0303145	0.100818155	-4.70231913	ns
CALCOCO2	-0.03429503	0.032612566	-2.1654573	0.03036831	0.100882214	-4.69145605	ns
CEMIP2	-0.01387887	0.00662168	-2.16279579	0.030572601	0.101423715	-4.69583012	ns
ATRN	-0.00747621	-0.01673262	-2.16242369	0.030600785	0.101423715	-4.71115855	ns
FYB1	-0.04440669	-0.03554146	-2.16104163	0.030707557	0.10147355	-4.70977968	ns

PAK4	-0.02905554	0.074696987	-2.16091213	0.030717833	0.10147355	-4.70204231	ns
IFNGR2	-0.02470759	0.106773127	-2.16088044	0.030720075	0.10147355	-4.70779015	ns
VEGFA	-0.02771407	0.029447754	-2.15750984	0.03098131	0.102220818	-4.72099033	ns
MYO9B	-0.03173857	0.098124964	-2.15603219	0.031096889	0.102370516	-4.71250162	ns
PAG1	-0.02623815	-0.01126442	-2.15571715	0.031121542	0.102370516	-4.71319562	ns
TRIM25	-0.04847444	-0.00986909	-2.15534704	0.031150071	0.102370516	-4.72548981	ns
ATP6V1G1	-0.03233388	0.034673694	-2.1550351	0.031174869	0.102370516	-4.71378786	ns
PADI2	-0.02750839	0.060466111	-2.15468854	0.031201972	0.102370516	-4.71537421	ns
PRSS27	-0.01886519	0.003530051	-2.15310823	0.031325811	0.102597651	-4.72312823	ns
TMPRSS5	-0.01858251	-0.00588975	-2.15290972	0.031341474	0.102597651	-4.7217566	ns
CEP170	-0.03461276	0.081483561	-2.1512083	0.031475599	0.102921333	-4.72196059	ns
MPIG6B	-0.03637605	-0.16822671	-2.15061942	0.031521784	0.102957056	-4.73274704	ns
FOXO1	-0.04038245	-0.01779692	-2.14839383	0.031698086	0.103303894	-4.73747261	ns
PPP1R2	-0.03773159	-0.00658199	-2.14838619	0.031698729	0.103303894	-4.73526745	ns
UMOD	0.025466417	0.009973374	2.146961771	0.031811808	0.103556834	-4.74409753	ns
HLA-A	-0.01432146	-0.03275361	-2.14601663	0.031887108	0.103686366	-4.7474244	ns
CRIM1	-0.00919725	-0.01135655	-2.14479994	0.031984428	0.103887129	-4.74628131	ns
TRAF2	-0.03344514	0.053272983	-2.14361002	0.032079819	0.10408119	-4.74765703	ns
GGACT	-0.03122518	0.024296281	-2.14294522	0.032133032	0.10413813	-4.75305674	ns
EBAG9	-0.04496658	-0.01514031	-2.1414233	0.032255696	0.10441977	-4.74931041	ns
CD63	-0.02452502	-0.05073654	-2.14091892	0.032296393	0.104435732	-4.74938193	ns
PI16	-0.01056616	0.005984555	-2.13957021	0.032405108	0.104671366	-4.75908143	ns
FGFBP2	0.021831246	0.026673303	2.137293916	0.032589713	0.105079841	-4.76450993	ns
AP1G2	-0.03563433	0.03947534	-2.13712428	0.03260354	0.105079841	-4.7644556	ns
BTN3A2	-0.01729927	-0.03912376	-2.13597486	0.032697243	0.105265656	-4.76386479	ns
QSOX1	-0.01830031	0.00675125	-2.13537528	0.032746312	0.105307524	-4.75956897	ns
LGALS3BP	-0.01333588	0.011790123	-2.13485957	0.032788126	0.105325992	-4.76991654	ns
VTI1A	-0.02586713	0.054787506	-2.13151876	0.03306248	0.106090595	-4.76471874	ns
RBPMS2	-0.04578539	0.010229808	-2.13059098	0.03313899	0.106219374	-4.76574227	ns
BID	-0.03134886	0.014622055	-2.12815146	0.033340418	0.106747831	-4.78041218	ns
SULT1A1	-0.06092249	-0.14694227	-2.12540614	0.033568501	0.107360375	-4.79110294	ns
SDCCAG8	-0.05053106	-0.01535221	-2.12340656	0.033735646	0.107776899	-4.79344717	ns
CHRDL1	-0.01390491	-0.02839594	-2.12015814	0.034008675	0.108243061	-4.79727668	ns
MPI	-0.0405234	0.02168952	-2.1199394	0.034027211	0.108243061	-4.79505645	ns
STK4	-0.04077248	-0.01258294	-2.11957107	0.034058283	0.108243061	-4.79626725	ns
WFDC12	-0.02976414	-0.06381056	-2.11945914	0.034067549	0.108243061	-4.80308179	ns
BAG3	-0.01898094	-0.04105563	-2.1192772	0.034083171	0.108243061	-4.79511148	ns
DCTN1	-0.0308306	0.000973225	-2.11902753	0.034103978	0.108243061	-4.80515465	ns
ADGRE2	-0.01299212	-0.00303074	-2.11675528	0.034296599	0.108736232	-4.80556443	ns
PCBD1	-0.02053393	0.020059573	-2.11516312	0.034432365	0.109048269	-4.79800629	ns
CMC1	-0.03918206	0.010765939	-2.11344039	0.03457904	0.109079928	-4.81535377	ns
C1RL	-0.00864263	-0.02239994	-2.11319684	0.034599903	0.109079928	-4.81464504	ns
C2CD2L	-0.01986876	0.003650349	-2.11277357	0.034636594	0.109079928	-4.8027996	ns
ASPSCR1	-0.02948065	0.002254882	-2.11221651	0.034683811	0.109079928	-4.81882261	ns
TOP2B	-0.03444157	0.014177289	-2.11204175	0.034699316	0.109079928	-4.8034307	ns
C1R	-0.00837115	-0.00150595	-2.11202142	0.034700573	0.109079928	-4.81802958	ns
SUGP1	-0.01778071	0.04969054	-2.11198355	0.034703854	0.109079928	-4.81728188	ns
PRTG	0.01130056	0.009911725	2.110634436	0.034819699	0.109283502	-4.82276522	ns
PAXX	-0.01968145	0.01019013	-2.11035069	0.034844512	0.109283502	-4.80925173	ns
SYT1	-0.01700408	0.032338692	-2.10987575	0.034885043	0.109283502	-4.82276819	ns
RAB44	-0.0189474	0.038410105	-2.10949029	0.034918324	0.109283502	-4.82266772	ns
GRPEL1	-0.02760967	0.019964552	-2.10898774	0.034961848	0.109302564	-4.81802837	ns
CTF1	-0.03910543	0.019650402	-2.10600504	0.035220414	0.109839389	-4.81826798	ns
NRGN	-0.03904845	-0.02731205	-2.10585476	0.035233032	0.109839389	-4.83008433	ns
BCAT1	-0.01290309	0.004959264	-2.10570078	0.035246407	0.109839389	-4.83155242	ns
KIF1C	-0.01546489	0.023050998	-2.1043526	0.035364261	0.110071627	-4.82003742	ns
SPRED2	-0.01965215	0.055718089	-2.10398496	0.035396321	0.110071627	-4.82027657	ns
CWC15	-0.0271655	0.103608748	-2.10221486	0.03555067	0.110433995	-4.83505887	ns
CFB	-0.01518923	-0.0487602	-2.09958266	0.035782036	0.111034585	-4.83463132	ns
NIT2	-0.03218237	-0.04011081	-2.09814509	0.035908548	0.111308873	-4.84597951	ns
EVI5	-0.03882456	-0.00236064	-2.09492409	0.036194431	0.112076075	-4.83914499	ns
SNAPIN	-0.02494	0.020923945	-2.09369166	0.036303638	0.112295152	-4.85747331	ns
CDKN1A	-0.04959487	-0.04700679	-2.09168176	0.036483441	0.112644709	-4.85408654	ns
LEP	-0.04488809	-0.17409801	-2.09156595	0.036493799	0.112644709	-4.85476354	ns
USP25	-0.03141306	0.006409947	-2.09095974	0.036547969	0.112683817	-4.86127898	ns
GBP4	-0.02232351	0.037923251	-2.09056656	0.03658365	0.112683817	-4.85036868	ns

LPP	-0.02876711	0.01550582	-2.08971977	0.036659745	0.11278187	-4.85047409	ns
PCYT2	-0.03267937	-0.02545529	-2.08901666	0.036722532	0.11278187	-4.86514307	ns
FCER1A	-0.01006789	-0.01828823	-2.08891795	0.036731356	0.11278187	-4.86737814	ns
RBM25	0.020008399	0.017726948	2.086225917	0.036974988	0.113410678	-4.85606151	ns
IFIT1	-0.02240092	0.039074776	-2.08466031	0.037116723	0.113419665	-4.86762576	ns
ADGRV1	0.029433317	0.076882391	2.084389542	0.037141537	0.113419665	-4.8632599	ns
SNU13	-0.02783617	0.150081495	-2.0839817	0.037178677	0.113419665	-4.8623515	ns
SLAMF8	-0.02397371	-0.09001592	-2.0839231	0.037183998	0.113419665	-4.863264	ns
FKBP5	-0.04385536	-0.06021899	-2.08349305	0.037222894	0.113419665	-4.87070754	ns
MECR	-0.03879154	-0.01244222	-2.08320812	0.037249063	0.113419665	-4.86476459	ns
PTTG1	-0.01755984	0.007710266	-2.08319758	0.037249814	0.113419665	-4.87064962	ns
HSD17B3	-0.02051613	0.038744087	-2.08033023	0.03751215	0.114099457	-4.8698928	ns
LRCH4	-0.02991693	0.028761743	-2.07960792	0.037578416	0.114182077	-4.87138301	ns
SYTL4	-0.03084533	0.093125142	-2.07790463	0.037734575	0.114438055	-4.88923875	ns
MICB_MICA	0.069511182	-0.52832135	2.077836105	0.037741044	0.114438055	-4.88538482	ns
GLA	-0.01642717	-0.02396195	-2.07620104	0.037892224	0.114753859	-4.87914603	ns
DCDC2C	-0.02667983	0.044513062	-2.07586083	0.037923792	0.114753859	-4.87745626	ns
RCOR1	-0.02035049	0.070401337	-2.07413189	0.038084007	0.11511936	-4.88434671	ns
GART	-0.01148548	0.018293948	-2.07210185	0.038272415	0.115463621	-4.90208941	ns
SIGLEC6	-0.01469321	-0.02438559	-2.07167412	0.038312323	0.115463621	-4.90359792	ns
PEPD	0.010992351	-0.00093538	2.070976824	0.038377495	0.115463621	-4.90234928	ns
AGER	-0.01850731	0.032440312	-2.07084574	0.038389947	0.115463621	-4.89655608	ns
ASAH1	-0.0199292	-0.00626588	-2.0706811	0.038405309	0.115463621	-4.89856827	ns
CORO1A	-0.04054019	0.098387256	-2.07036649	0.038435151	0.115463621	-4.88785084	ns
NAA10	-0.0215527	-0.00064846	-2.06710468	0.038741369	0.116263923	-4.89709658	ns
BCAN	0.015736805	0.042684075	2.065089088	0.038931411	0.11671429	-4.908578	ns
TBC1D5	-0.03606317	0.097886446	-2.06312627	0.039117417	0.117098218	-4.91559968	ns
VWF	-0.03374949	-0.04195729	-2.06289009	0.039139678	0.117098218	-4.92052967	ns
SLITRK2	-0.0178594	-0.01402865	-2.06193239	0.039230783	0.117250651	-4.92357429	ns
GP6	-0.04036778	-0.0482244	-2.06074946	0.039343777	0.117404585	-4.9198021	ns
FN1	-0.0113278	-0.0144894	-2.06009394	0.039406222	0.117404585	-4.92550021	ns
BOC	-0.01068449	0.00589777	-2.05947953	0.039465467	0.117404585	-4.91438576	ns
DDHD2	-0.02834005	0.006013636	-2.059408	0.039471809	0.117404585	-4.92810595	ns
ATG16L1	-0.03630508	-0.00849424	-2.05928841	0.039483323	0.117404585	-4.92631775	ns
IRAK4	-0.04757277	-0.12338317	-2.05814648	0.039592924	0.11761072	-4.92577984	ns
TBC1D23	-0.04153747	0.017106392	-2.05691999	0.039710839	0.117795133	-4.92561775	ns
SELPLG	0.012091551	-0.01906227	2.056661298	0.039735687	0.117795133	-4.92881124	ns
CXCL12	0.01776468	0.002110372	2.055532777	0.039844724	0.117998573	-4.92242042	ns
RALY	-0.0207319	0.029304294	-2.05478752	0.039916311	0.118090808	-4.93566836	ns
PTPRZ1	-0.01383658	0.020720232	-2.05353528	0.040037445	0.118295609	-4.93918308	ns
HEXIM1	-0.03733032	-0.00232697	-2.0532365	0.040066561	0.118295609	-4.93579323	ns
PSTPIP2	-0.04572357	-0.0979737	-2.05130128	0.040255429	0.118733185	-4.91437747	ns
PCDH17	-0.01545043	0.01419953	-2.05072643	0.040310583	0.118775886	-4.93867415	ns
LRRFIP1	-0.02085065	0.057675848	-2.04980796	0.040400018	0.118919408	-4.94580739	ns
MFAP3	-0.02186702	0.035822233	-2.04897156	0.04048218	0.119041254	-4.93579727	ns
GASK1A	-0.01841466	-0.01555889	-2.04657351	0.040717333	0.119612287	-4.93898385	ns
MTSS1	-0.04726572	-0.00883268	-2.04582238	0.04079073	0.119707469	-4.95373973	ns
STAT5B	-0.06221209	-0.04174943	-2.03920869	0.041446353	0.121509389	-4.96160256	ns
THOP1	-0.01404665	-0.00935662	-2.03652413	0.041715014	0.122173008	-4.96532638	ns
ESM1	0.015754775	0.024057836	2.036034275	0.041764075	0.122173008	-4.96840737	ns
SNCG	-0.03061672	-0.09860755	-2.03545716	0.041822125	0.122173008	-4.968847	ns
ELN	-0.0139105	-0.04060549	-2.03527628	0.041840071	0.122173008	-4.9762185	ns
PHOSPHO1	-0.0129599	-0.03324967	-2.03136924	0.042234835	0.123186691	-4.97709317	ns
SLC34A3	-0.02083756	0.03523829	-2.03100955	0.042271597	0.123186691	-4.96880706	ns
ART5	-0.02587264	0.126352444	-2.02817861	0.042559567	0.123640335	-4.97791271	ns
CBLN1	-0.03987446	0.206291956	-2.02816844	0.042560604	0.123640335	-4.97793316	ns
ZFYVE19	-0.03732655	-0.00220973	-2.02805615	0.042571627	0.123640335	-4.98964346	ns
TIMM10	-0.01654906	0.043848896	-2.02763029	0.042615082	0.123640335	-4.99162895	ns
GNE	-0.02923744	0.031629852	-2.02740137	0.042638978	0.123640335	-4.97851402	ns
GTPBP2	-0.03367403	0.035633194	-2.02597594	0.04278478	0.123940039	-4.98202352	ns
LILRA5	-0.01478793	-0.05301764	-2.02326717	0.04306298	0.124622301	-4.99357762	ns
TPPP3	-0.01754776	0.026668434	-2.02169909	0.043225088	0.124967581	-4.9908263	ns
PPP2R5A	-0.04885347	0.03624698	-2.0156655	0.043852231	0.126655305	-5.01449842	ns
PTN	-0.0213597	-0.04440961	-2.01208205	0.044229054	0.12761743	-5.01009832	ns
ABO	0.059339376	-0.17263805	2.010182235	0.04442922	0.128068433	-5.0254283	ns
AMOTL2	-0.02020048	0.101791668	-2.00515255	0.044964219	0.12939587	-5.03357678	ns

STX6	-0.03047981	0.130004856	-2.00465116	0.045018241	0.12939587	-5.02400865	ns
CDH6	-0.01637477	0.002957018	-2.00460684	0.045022673	0.12939587	-5.03144522	ns
RHOC	-0.0522387	-0.14197442	-2.0025145	0.045247187	0.129913262	-5.03149223	ns
MRPS16	-0.01207884	0.03521769	-2.001361	0.045371439	0.130142044	-5.02922214	ns
FLI1	-0.04858367	0.050508276	-1.9980025	0.045734362	0.130854363	-5.03720739	ns
NEB	-0.01538692	0.034835709	-1.99789742	0.045745768	0.130854363	-5.03662198	ns
CBLIF	0.030405999	0.023234483	1.997721278	0.045764577	0.130854363	-5.04452858	ns
GYS1	-0.04065621	0.009472521	-1.99740619	0.045799027	0.130854363	-5.03928259	ns
DNAJB14	-0.02654991	0.022745037	-1.99484952	0.046076816	0.131519358	-5.05591143	ns
FUT8	-0.02562699	0.050723072	-1.99346723	0.046228068	0.131822225	-5.05276029	ns
DDX25	0.015334748	0.03802396	1.991563474	0.046436939	0.132288647	-5.05059291	ns
IGHMBP2	-0.02299228	0.082171458	-1.98976513	0.046634901	0.132723109	-5.05270533	ns
CD300LF	-0.02615323	-0.16256069	-1.98926517	0.046689466	0.132749018	-5.06961639	ns
SPRY2	-0.04063804	0.051050357	-1.98774374	0.046858062	0.132984757	-5.05836044	ns
CNGB3	-0.02268624	0.079514158	-1.98769523	0.046863464	0.132984757	-5.0585458	ns
ADAMTS15	-0.02008785	-0.04762613	-1.98635285	0.047011971	0.133276656	-5.06714294	ns
CALB1	-0.01843727	-0.01608964	-1.98419292	0.047252177	0.133703042	-5.0713969	ns
CAMSAP1	-0.04485778	0.043670477	-1.98417468	0.047253952	0.133703042	-5.07901968	ns
SERPINA9	-0.03092711	0.001594051	-1.98135285	0.047569695	0.134466128	-5.07445114	ns
NAA80	-0.04914686	0.011217804	-1.97894605	0.0478402	0.134978348	-5.07543991	ns
NMT1	-0.02672634	0.101346928	-1.97891383	0.047843353	0.134978348	-5.08936268	ns
NDUFB7	-0.0359452	0.022389467	-1.97746555	0.04800676	0.135182726	-5.09016726	ns
NFE2	-0.03074768	0.033972294	-1.97745516	0.048008386	0.135182726	-5.07774351	ns
CCL19	-0.05200729	0.236038158	-1.97683927	0.048077488	0.135246883	-5.09344062	ns
CA13	-0.04558439	-0.11725211	-1.97602648	0.048169756	0.135376021	-5.08681783	ns
PSPN	0.042812972	-0.13095437	1.974008415	0.048398694	0.135761138	-5.09406565	ns
HSPA1A	-0.03143249	0.024582347	-1.97399841	0.048399776	0.135761138	-5.09461181	ns
CD69	-0.03810807	-0.07174419	-1.97259241	0.048560095	0.136080112	-5.09137037	ns
KIF22	-0.04940644	-0.16132158	-1.9720523	0.048621939	0.136122783	-5.08750934	ns
AKT2	-0.0511458	-0.03975172	-1.97060749	0.04878653	0.136423817	-5.10563733	ns
PPP1R14A	-0.03184769	-0.00945929	-1.97028984	0.048822907	0.136423817	-5.10625835	ns
PARK7	-0.02315868	-0.00786113	-1.96798525	0.049087685	0.137032542	-5.10621726	ns
CNPY2	-0.02523356	0.093270407	-1.96691581	0.049211241	0.137246251	-5.09923281	ns
SPINT2	-0.01740781	-0.02112183	-1.9648674	0.049447519	0.137773622	-5.11308862	ns
FUS	-0.01740093	0.009529481	-1.963686	0.049584938	0.138024803	-5.10399692	ns
APPL2	-0.045679	-0.06227341	-1.96137503	0.049853584	0.138592435	-5.11009991	ns
CCL14	-0.01700402	-0.02449548	-1.96084786	0.049914972	0.138592435	-5.11520737	ns
SMPD1	-0.02074013	-0.01653308	-1.96070631	0.049931247	0.138592435	-5.122714	ns
FGF5	-0.01426884	-0.00604983	-1.95987354	0.050028931	0.138689393	-5.11292808	ns
VSNL1	-0.0148288	0.002937973	-1.95957413	0.050063483	0.138689393	-5.12618751	ns
KLK10	-0.01687282	-0.01359044	-1.95918905	0.050108668	0.138689393	-5.12357	ns
TALDO1	-0.01828655	-0.00492585	-1.95805317	0.050241785	0.138926147	-5.12685381	ns
HS1BP3	-0.03635182	-0.01056884	-1.95733576	0.050326428	0.139028542	-5.11700722	ns
GORASP2	-0.0190447	0.037415115	-1.95463505	0.050644155	0.139774039	-5.13470047	ns
TP53	-0.0184615	-0.00850661	-1.95042419	0.051144127	0.141020633	-5.1303796	ns
STXBP1	-0.03091871	-0.04055021	-1.94641078	0.051623432	0.142106568	-5.15265609	ns
THPO	-0.01626487	-5.06E-05	-1.9463165	0.051635298	0.142106568	-5.13921348	ns
NGFR	-0.01130739	0.000947915	-1.94500696	0.051792213	0.142272228	-5.15445778	ns
ADH1B	-0.03072665	0.054649693	-1.94478116	0.051819808	0.142272228	-5.14224838	ns
MGLL	-0.03764061	-0.09340847	-1.94459735	0.051841661	0.142272228	-5.1512561	ns
CNTN4	0.01016437	0.008460208	1.944063338	0.051906037	0.142315145	-5.15263964	ns
RRM2B	-0.02897306	0.014017249	-1.94251022	0.052094002	0.142696516	-5.14565978	ns
PEAR1	-0.00963095	-0.00098816	-1.94178086	0.052181717	0.142802825	-5.16112944	ns
DHRS4L2	-0.01903235	0.068242472	-1.94137101	0.052231392	0.14280493	-5.16141398	ns
ECHDC3	-0.0287468	0.055146059	-1.94091938	0.052286176	0.142820985	-5.1623376	ns
SEMA4D	-0.01262607	-0.00860303	-1.93937716	0.052473543	0.143198827	-5.16622164	ns
MICALL2	-0.02855214	0.130576645	-1.93846754	0.052584801	0.143368459	-5.15408727	ns
SELP	-0.02431041	-0.00719233	-1.93799021	0.052642728	0.143392506	-5.16314592	ns
NUDT5	-0.02211769	0.009041588	-1.93594639	0.052892582	0.143778133	-5.1682623	ns
C4BPB	-0.01646399	0.004697046	-1.93571651	0.052920669	0.143778133	-5.17005497	ns
SUGT1	-0.03453704	-0.02594051	-1.93526363	0.052976673	0.143778133	-5.15959691	ns
POSTN	0.014564253	0.005027423	1.935221913	0.052981257	0.143778133	-5.17274371	ns
MMP3	-0.02000583	0.007562566	-1.9339511	0.053137477	0.144068183	-5.17209262	ns
IGFBP2	-0.02903785	-0.02548447	-1.93316963	0.053233514	0.144194677	-5.17664363	ns
ASAH2	-0.02316831	-0.07316103	-1.93231582	0.05333901	0.144346532	-5.17183927	ns
FADD	-0.03230332	-0.00390822	-1.93146728	0.053443729	0.144496008	-5.17424354	ns

DNM1	-0.0452117	-0.09300698	-1.92898416	0.053751108	0.145192632	-5.18411856	ns
SOD2	-0.01544327	0.000823853	-1.92774166	0.053905672	0.145475565	-5.1839875	ns
TGFBR3	-0.01331934	-0.00747867	-1.92657799	0.054050492	0.145677802	-5.19030445	ns
LEPR	0.013569427	0.027827652	1.926340298	0.05408039	0.145677802	-5.18405455	ns
CXCL10	-0.02856076	0.031406084	-1.92572708	0.054156831	0.14574926	-5.18862819	ns
CASP2	-0.02865435	0.02706732	-1.92411283	0.054358916	0.146158412	-5.19054688	ns
IGFBP1	0.05612497	0.008823951	1.921405944	0.054698885	0.146817302	-5.1993196	ns
MITD1	-0.04176432	-0.07896957	-1.92136319	0.054704529	0.146817302	-5.1927756	ns
SMAD2	-0.02717711	0.062553313	-1.91949312	0.054940352	0.147253922	-5.20240398	ns
WNT9A	-0.01175336	-0.0054908	-1.91927725	0.054968074	0.147253922	-5.19103452	ns
CSH1	0.012245796	-0.01500649	1.918455628	0.055071636	0.147396129	-5.2041929	ns
CXCL3	-0.04045123	-0.09518409	-1.91593038	0.055392551	0.148119275	-5.20408352	ns
TRIM24	-0.02535694	0.040235476	-1.9148551	0.0555297	0.14835016	-5.20307718	ns
IL1RAP	0.01917119	0.143366245	1.914082504	0.055628166	0.148407966	-5.2100186	ns
CCN1	-0.03242004	-0.1504574	-1.91388919	0.055652987	0.148407966	-5.20665671	ns
XIAP	-0.02292549	0.048017503	-1.91275859	0.055797942	0.148658749	-5.20122094	ns
CASP7	-0.03283486	-0.01620743	-1.91199219	0.055895612	0.148783214	-5.21757439	ns
CDC37	-0.03534342	0.032377899	-1.90960446	0.056202677	0.149464315	-5.2198647	ns
BCR	-0.0383424	-0.03220914	-1.90555683	0.056726398	0.150621588	-5.22579814	ns
PZP	-0.02136018	0.208852998	-1.90544294	0.056741009	0.150621588	-5.22973069	ns
KLK12	-0.05784246	-0.54089191	-1.90270338	0.057098337	0.151432465	-5.22157649	ns
EIF2AK3	-0.02655448	0.105939308	-1.90110094	0.057307897	0.151850327	-5.22380352	ns
NDST1	-0.02465126	0.123615671	-1.90003139	0.0574476	0.151946746	-5.23927985	ns
MERTK	-0.01046639	-0.01656015	-1.90002486	0.057448359	0.151946746	-5.24113931	ns
PCBP2	-0.0280225	-0.00017281	-1.89876362	0.057614088	0.152206101	-5.24354614	ns
DUSP13	-0.01614449	0.043209695	-1.89848629	0.057650667	0.152206101	-5.24219138	ns
MAPK9	-0.0177541	-0.01240264	-1.8980789	0.057704437	0.152210438	-5.23990761	ns
REG1A	-0.01963984	0.030304079	-1.89537202	0.058062121	0.153015697	-5.2387284	ns
RABEP1	-0.03051746	0.025085284	-1.89178148	0.058539161	0.154045388	-5.24309994	ns
STOML2	-0.03658403	0.116380286	-1.89163871	0.05855835	0.154045388	-5.23994383	ns
GMFG	-0.02790245	-0.01471268	-1.89113745	0.058624642	0.154080967	-5.25697464	ns
VPS37A	-0.03351917	0.032096605	-1.88837809	0.058994113	0.154912599	-5.25544864	ns
ANXA5	-0.01204321	-0.01588458	-1.88622701	0.059283614	0.155532931	-5.25273032	ns
ARL13B	-0.02739329	0.079931612	-1.88471076	0.059488173	0.155929502	-5.25457863	ns
FXN	-0.03016179	-0.00255013	-1.87988886	0.060141942	0.157501767	-5.27956137	ns
CD36	-0.01734019	0.029869116	-1.87866623	0.060309413	0.157798822	-5.26532894	ns
GADD45GIP1	-0.02007131	0.036425935	-1.87694948	0.060544312	0.158217901	-5.26987871	ns
PLA2G4A	-0.03659123	-0.02162898	-1.87659248	0.060592844	0.158217901	-5.28130585	ns
PLXNA4	-0.03385351	-0.03786337	-1.87621365	0.060644915	0.158217901	-5.2808452	ns
NUMB	-0.03256744	-0.0424034	-1.87591495	0.060686318	0.158217901	-5.27045226	ns
ARL2BP	-0.01569429	0.02596605	-1.87428244	0.060910886	0.158661719	-5.27402078	ns
PDGFRA	-0.01148703	-0.00175355	-1.87274024	0.061123308	0.159073138	-5.28506325	ns
LTA4H	-0.02379994	0.041349871	-1.8651007	0.062186147	0.161695057	-5.30703636	ns
TST	-0.03079176	0.043955913	-1.86281128	0.062508314	0.162141208	-5.29576567	ns
MRPL28	-0.01400145	0.041628354	-1.8626975	0.062523713	0.162141208	-5.31084748	ns
LILRB1	-0.01134287	-0.02574908	-1.86269333	0.062524315	0.162141208	-5.3103968	ns
VSTM1	-0.02014149	-0.00777882	-1.86210081	0.062608333	0.162215025	-5.29825768	ns
KLK15	0.019504031	0.013451287	1.859666328	0.062951775	0.162843601	-5.31378914	ns
SLIRP	0.022027504	0.093171032	1.859594335	0.062962475	0.162843601	-5.29956119	ns
UNC5D	-0.01016203	0.01333901	-1.85916478	0.063023307	0.162856688	-5.30148178	ns
DTX3	-0.00936415	-0.02128341	-1.85553262	0.063539425	0.164045201	-5.31644668	ns
VWA5A	0.029869908	0.153254762	1.853809779	0.063785967	0.164536239	-5.31021466	ns
MYO6	-0.01403067	0.021837581	-1.85288778	0.06391741	0.164729776	-5.32892979	ns
ISLR2	-0.01530579	-0.00849424	-1.85184872	0.064067181	0.164970165	-5.31468458	ns
GLO1	-0.0240169	-0.01448294	-1.85144877	0.064124572	0.164972467	-5.3179095	ns
OTOA	0.020084167	0.09108119	1.850347895	0.064282972	0.165234401	-5.3200006	ns
WASF3	-0.03401999	-0.02752928	-1.8498143	0.064359886	0.165279552	-5.32087804	ns
CXCL5	-0.04694814	-0.08330736	-1.84912695	0.064458825	0.165279552	-5.32679571	ns
DYNLT1	-0.03020644	0.141053321	-1.84904834	0.064470346	0.165279552	-5.32206533	ns
HEG1	-0.01017498	-0.03468438	-1.84770928	0.064663898	0.165630334	-5.32484277	ns
TBL1X	-0.03497462	-0.04315182	-1.84722411	0.064734291	0.16566532	-5.32476184	ns
OGFR	-0.01352606	0.002892987	-1.84605815	0.064903069	0.165866915	-5.33384312	ns
CHCHD10	-0.01464521	-0.04371587	-1.84589387	0.064926673	0.165866915	-5.34079836	ns
GNLY	0.021090397	-0.04255065	1.844865155	0.065076384	0.166080541	-5.3390861	ns
ARHGEF10	-0.03557561	0.042159981	-1.84453639	0.065124048	0.166080541	-5.34424875	ns
ASRGL1	-0.03869001	0.018850975	-1.84365914	0.065252442	0.166262766	-5.33131127	ns

CGB3_CGB5_C	-0.01608845	-0.00984943	-1.84198685	0.065496189	0.166738337	-5.34684442	ns
TIA1	-0.04880643	0.008295272	-1.84147519	0.065571777	0.166785355	-5.33153295	ns
AP3B1	-0.02925085	0.032140695	-1.84085491	0.065661969	0.166869409	-5.34893318	ns
THTPA	-0.03319938	0.001931599	-1.83955415	0.065852927	0.167209171	-5.35052937	ns
SELL	-0.00857782	0.010235057	-1.83840996	0.066021147	0.167490661	-5.35423015	ns
CASP9	-0.02320146	0.027052238	-1.83745902	0.066161883	0.167701994	-5.34127765	ns
CXCL11	-0.03126877	0.023056925	-1.83433369	0.066623625	0.16872592	-5.36287651	ns
ING1	-0.01866363	0.014180341	-1.83328149	0.066780484	0.168938636	-5.3497936	ns
AMPD3	0.02752656	0.047963447	1.832936104	0.066831243	0.168938636	-5.36444695	ns
FCRLB	-0.02382526	0.046436327	-1.83244656	0.066904674	0.168938636	-5.35173176	ns
COMMD9	-0.01473203	0.046024029	-1.83221225	0.066939042	0.168938636	-5.36484764	ns
ITIH5	-0.01718668	0.028145376	-1.8301487	0.067246961	0.169284873	-5.36859681	ns
MBL2	-0.04031956	-0.09040089	-1.82992265	0.06728103	0.169284873	-5.36154349	ns
BABAM1	-0.01080172	0.022090663	-1.82982446	0.067295981	0.169284873	-5.355164	ns
LRTM2	-0.0101761	0.040097765	-1.8297391	0.067308129	0.169284873	-5.37122157	ns
NCS1	-0.01103141	-0.0294056	-1.82818802	0.067540522	0.169723172	-5.37467027	ns
AFM	-0.007564	-0.01221922	-1.82577413	0.067903528	0.170488652	-5.37719932	ns
VPS4B	-0.03533066	0.019127495	-1.82425613	0.068132664	0.170666845	-5.37927953	ns
PKN3	-0.027949	0.05040816	-1.82399953	0.068171681	0.170666845	-5.37298674	ns
NT5C	-0.02788558	0.006915487	-1.82377416	0.068205484	0.170666845	-5.38112145	ns
GFRAL	0.022932492	0.029409398	1.823759552	0.06820829	0.170666845	-5.3663282	ns
LACTB2	-0.02634787	0.015450258	-1.82317806	0.06829592	0.1707398	-5.37592755	ns
HMBS	0.017610647	-0.02120055	1.82114286	0.068604387	0.171085582	-5.38742532	ns
SOST	0.015602824	-0.03158784	1.820753875	0.068663823	0.171085582	-5.3797199	ns
SARG	-0.04132806	-0.06807593	-1.82072547	0.068667978	0.171085582	-5.38444492	ns
CSF3	-0.01991103	-0.0016873	-1.8206195	0.068684491	0.171085582	-5.37403923	ns
PPM1F	-0.01973564	0.009556691	-1.8203357	0.068727188	0.171085582	-5.38733761	ns
SERPINA4	-0.00876632	-0.0074732	-1.81942363	0.068866518	0.171286399	-5.37926283	ns
GAS6	-0.00994161	-0.00389406	-1.81874124	0.068970338	0.171398627	-5.38698354	ns
TXNRD1	-0.01888461	0.014519003	-1.81574504	0.069428969	0.172241039	-5.39196562	ns
SIGLEC5	-0.04736444	-0.34862665	-1.8151165	0.06952549	0.172241039	-5.39309772	ns
CST7	-0.03814186	0.069101993	-1.81506828	0.069532922	0.172241039	-5.39190447	ns
PSMD5	0.020615168	0.079579642	1.814989923	0.069545269	0.172241039	-5.38396074	ns
FABP3	-0.01852656	-0.02465986	-1.81362183	0.069755312	0.17261484	-5.39944139	ns
IL36G	-0.02507724	0.077347882	-1.81266387	0.069903707	0.172835583	-5.38451073	ns
SORT1	-0.01473785	-0.00768881	-1.81026063	0.07027501	0.173567947	-5.39861971	ns
ICAM5	-0.01822588	-0.04201857	-1.80997804	0.070318795	0.173567947	-5.3997169	ns
BTC	-0.02985454	0.089865594	-1.80835825	0.070570723	0.174042662	-5.39523025	ns
CDH5	-0.01174492	0.02301617	-1.80664886	0.070836417	0.174550497	-5.41241797	ns
OPLAH	-0.02559041	0.026716267	-1.80469114	0.071142971	0.175158073	-5.4018252	ns
SEPTIN9	-0.01141362	0.026481088	-1.80236129	0.071508256	0.175909106	-5.41292462	ns
DDC	0.018765047	0.041972203	1.800334824	0.071827494	0.176545693	-5.41639699	ns
TNFRSF6B	-0.02176647	-0.0338869	-1.79851193	0.072115662	0.177104905	-5.41945995	ns
STXBP3	-0.01707708	0.054212812	-1.79298132	0.072995994	0.17911622	-5.42269691	ns
MRPL46	-0.01045469	0.007839907	-1.79240644	0.073087938	0.179191249	-5.42458648	ns
MLN	-0.0353484	-0.0711332	-1.79079753	0.073345496	0.179671853	-5.43622331	ns
CRISP2	0.022657817	-0.04755949	1.789177573	0.073605716	0.180158164	-5.44467912	ns
PSME2	-0.01473662	-0.00070037	-1.78756804	0.07386563	0.180642914	-5.4370639	ns
CAMKK1	-0.01768844	0.027057884	-1.78528132	0.074235839	0.18139636	-5.43636277	ns
RILPL2	-0.03601739	-0.07044128	-1.78324169	0.074566545	0.1820521	-5.4536519	ns
FMNL1	-0.025058	0.016024313	-1.7827364	0.074649349	0.182102004	-5.44173401	ns
EFCAB2	-0.01279894	0.026918773	-1.78149922	0.074850805	0.182441028	-5.44899703	ns
SEL1L	-0.01143654	0.00306191	-1.77769214	0.075474581	0.183558978	-5.44979781	ns
SCT	0.016529949	0.04387826	1.777234895	0.075549704	0.183558978	-5.45122992	ns
ICOSLG	-0.00744915	0.003342288	-1.77716894	0.075560313	0.183558978	-5.45765662	ns
CA11	-0.01195928	0.047150199	-1.77716729	0.075560922	0.183558978	-5.45069982	ns
LRFN2	0.017456553	0.057596577	1.776345223	0.075696144	0.183734614	-5.45279802	ns
SERPING1	0.006843336	-0.01211803	1.775709332	0.075800507	0.183835117	-5.46505766	ns
MUC16	-0.07347291	-0.57778733	-1.77463934	0.075977339	0.184111062	-5.46211344	ns
CUZD1	-0.01348337	0.048279027	-1.7736383	0.076143128	0.184290603	-5.45756434	ns
ZNRD2	-0.02467546	0.042293767	-1.773427	0.076177657	0.184290603	-5.4698322	ns
CTSV	0.015830497	0.03414461	1.772564248	0.076320626	0.184483632	-5.47209252	ns
SPART	-0.02860417	0.012599756	-1.77206777	0.076403409	0.18453098	-5.46064932	ns
DAG1	-0.01887301	-0.01277484	-1.77016939	0.076718689	0.185139315	-5.47383234	ns
TCL1A	-0.03883886	-0.04303184	-1.76942763	0.076842519	0.185228946	-5.47003129	ns
MAEA	-0.01527171	0.026024076	-1.76905246	0.076905381	0.185228946	-5.46497298	ns

MYCBP2	-0.02462071	0.084376171	-1.76880453	0.076946134	0.185228946	-5.48000598	ns
PTPN1	-0.03132273	0.050322175	-1.76799019	0.077082508	0.185404386	-5.47345741	ns
ITPA	-0.02438992	0.00633964	-1.76684961	0.077273707	0.185711295	-5.46803698	ns
PPP1CC	-0.03019142	0.042253375	-1.76546501	0.077505394	0.186114928	-5.4839769	ns
CFHR2	-0.02063627	-0.12068006	-1.76503563	0.077577643	0.186135348	-5.48155125	ns
CDC26	-0.02564606	0.044959505	-1.76380269	0.077784995	0.186479626	-5.48539537	ns
ADA	-0.01352547	0.013489919	-1.76181295	0.078120575	0.1871305	-5.49222226	ns
OSMR	-0.00783411	-0.01526847	-1.76123355	0.07821874	0.187209773	-5.48893297	ns
LTO1	-0.01614209	0.019807427	-1.76043701	0.078353973	0.187209773	-5.4809352	ns
TWF2	-0.03049927	0.016038867	-1.76040272	0.07835929	0.187209773	-5.49325087	ns
CPM	-0.01422354	-0.0439586	-1.76010326	0.078410121	0.187209773	-5.49297234	ns
TEK	-0.00812871	0.00625458	-1.75914879	0.078572049	0.187443123	-5.49369219	ns
ARHGAP1	-0.01801742	-0.00398809	-1.75672402	0.078985206	0.188115458	-5.48653251	ns
FDX1	-0.0226061	0.029802993	-1.75630334	0.079056988	0.188115458	-5.48646596	ns
NFX1	-0.02475307	0.063418784	-1.75554607	0.079186313	0.188115458	-5.487785	ns
DNAJB6	-0.03062398	-0.00258238	-1.75510366	0.079262005	0.188115458	-5.48547732	ns
NUB1	-0.03235268	0.044894868	-1.75493477	0.079290422	0.188115458	-5.49930362	ns
GCG	-0.04850943	0.020933821	-1.75489922	0.079296903	0.188115458	-5.48971084	ns
DCC	-0.0081535	0.018421443	-1.75485227	0.079304839	0.188115458	-5.49044231	ns
CD86	-0.0102058	-0.00165069	-1.75253738	0.079701266	0.188544255	-5.50637267	ns
APOD	0.015611524	0.047112269	1.752381107	0.079729005	0.188544255	-5.48508836	ns
NECAP2	-0.02397773	0.090447479	-1.75236653	0.079731189	0.188544255	-5.49331702	ns
TDP1	-0.02585796	0.089321489	-1.75228943	0.079743889	0.188544255	-5.50697026	ns
FKBP7	-0.01816974	0.046984911	-1.75075479	0.080008554	0.188810787	-5.49649597	ns
AKR7L	-0.02493626	0.074744932	-1.75061491	0.080032659	0.188810787	-5.49582635	ns
CES1	-0.03130883	0.040288191	-1.75050831	0.080050601	0.188810787	-5.5061364	ns
CASC3	-0.01497222	0.029887722	-1.74911201	0.080291956	0.189051598	-5.5004187	ns
TIMP2	-0.00899185	-0.00708621	-1.74906189	0.080300129	0.189051598	-5.5135483	ns
FGF16	0.017017855	0.023372222	1.748794643	0.080346929	0.189051598	-5.49898571	ns
CXCL14	0.023933772	0.046778147	1.747556764	0.080561125	0.18933535	-5.50334648	ns
SAT1	0.01793201	0.094941549	1.746912689	0.080672906	0.18933535	-5.50278274	ns
DPP7	-0.0226838	-0.0288278	-1.74687546	0.080679102	0.18933535	-5.50866247	ns
C7	-0.01096387	-0.02684234	-1.74660107	0.080726887	0.18933535	-5.50576351	ns
TREH	0.027076344	-0.05782232	1.745344376	0.080944779	0.189694024	-5.51944044	ns
MED21	-0.01853888	0.072308115	-1.74440133	0.081109571	0.189927784	-5.50713154	ns
EIF4G3	-0.02399421	0.084483404	-1.74354405	0.081258424	0.190097092	-5.52402292	ns
MYL3	0.025682967	0.031650908	1.743240033	0.081312078	0.190097092	-5.50996371	ns
ICA1	-0.0305348	0.050665138	-1.73994778	0.081888554	0.191233479	-5.51650862	ns
BCL2L1	-0.0354737	-0.02771123	-1.73964747	0.081940788	0.191233479	-5.52978845	ns
FRMD4B	-0.02586759	0.107944685	-1.73919138	0.082021575	0.191233479	-5.51613343	ns
VSIG10L	-0.01160352	0.011452428	-1.73897223	0.082060119	0.191233479	-5.5165115	ns
CRIP2	-0.01423425	-0.04293681	-1.73850745	0.082141594	0.191270697	-5.52473955	ns
CTRL	-0.02336577	-0.00451214	-1.73562536	0.082649972	0.192301131	-5.536723	ns
PDE5A	-0.03724876	-0.10372039	-1.73430481	0.082884361	0.192692941	-5.52632562	ns
TRIM58	-0.03090311	0.022676065	-1.73383558	0.082967208	0.192732099	-5.53888553	ns
GAD2	-0.01629867	0.056749684	-1.73166638	0.083353491	0.193331252	-5.52855353	ns
RIDA	-0.01807336	0.006284974	-1.73164276	0.08335755	0.193331252	-5.53090204	ns
RET	-0.0149995	-0.01237474	-1.72998154	0.083653742	0.193864228	-5.53974989	ns
GPI	-0.01666041	0.021123681	-1.72873183	0.083877041	0.194227565	-5.54746534	ns
SPRR1B	-0.03040182	0.165800298	-1.72782849	0.0840392	0.194355829	-5.54242594	ns
TXLNA	-0.03414666	-0.04624047	-1.72768155	0.084065552	0.194355829	-5.54335369	ns
ATP6AP2	-0.01553274	0.028756029	-1.72701345	0.084185777	0.194479801	-5.53787003	ns
LY96	-0.01359172	-0.01404267	-1.72631837	0.084310379	0.194613681	-5.54568931	ns
FGA	-0.01109611	-0.01775554	-1.72542788	0.084470844	0.194780844	-5.54181787	ns
CHMP6	-0.01997828	-0.00209713	-1.72517322	0.084516209	0.194780844	-5.55472269	ns
PLA2G1B	-0.01601549	0.024443962	-1.72398639	0.084730271	0.194927669	-5.55547581	ns
NFKB1	-0.02651783	0.016001264	-1.72383034	0.084759037	0.194927669	-5.54251798	ns
CACNA1C	-0.01144051	0.02314259	-1.7237129	0.084780185	0.194927669	-5.5444903	ns
LAP3	-0.01841712	-0.01718026	-1.72315936	0.084880174	0.195004019	-5.54534638	ns
CILP	-0.01890313	-0.08372801	-1.72268146	0.084966112	0.195047994	-5.55843199	ns
GATD3	-0.02716161	0.025262772	-1.72032303	0.085394258	0.195876852	-5.54933329	ns
SEMA3G	-0.01521853	0.028069017	-1.71956236	0.085532454	0.196039847	-5.55158151	ns
CEBPA	-0.01365628	0.048671643	-1.71800745	0.085815845	0.196535112	-5.55192389	ns
NFIC	-0.01545817	0.05872239	-1.7171119	0.085978675	0.196753707	-5.5694039	ns
GABARAPL1	-0.0241222	0.151129101	-1.71434564	0.086485561	0.197758684	-5.56544765	ns
GSTA3	-0.02983747	0.061480986	-1.71212882	0.086893542	0.198414895	-5.56326039	ns

RAD51	0.019028215	0.045274108	1.712046314	0.086908442	0.198414895	-5.56935572	ns
APEX1	-0.02281388	-0.01695463	-1.71146653	0.087015074	0.198503137	-5.57777761	ns
VSIR	-0.02302845	0.061948223	-1.70917298	0.087439181	0.199314916	-5.57569143	ns
TPR	-0.01735901	0.035559665	-1.70873607	0.087520492	0.199344646	-5.5682164	ns
MAX	-0.03963257	-0.00364015	-1.7064872	0.087938012	0.200045931	-5.57369589	ns
VEGFB	-0.01018845	-0.0276868	-1.70634023	0.087965403	0.200045931	-5.57173995	ns
AKT3	-0.02148552	0.020927993	-1.70583947	0.088058734	0.200102336	-5.57104069	ns
ABRAXAS2	-0.02323895	0.023782127	-1.70182471	0.088808911	0.201650094	-5.57990779	ns
SRP14	-0.02341747	-0.01340821	-1.69959543	0.089227381	0.202442853	-5.59144467	ns
KLK11	-0.01089052	-0.0091138	-1.69777466	0.08957061	0.203063805	-5.59499845	ns
PEBP1	-0.02231862	0.00087832	-1.69670839	0.089772055	0.203362607	-5.59938036	ns
KAZN	-0.03657414	-0.05088238	-1.69486108	0.090121911	0.203996884	-5.60501383	ns
PBXIP1	-0.01068748	0.009272955	-1.69141321	0.090778061	0.205185018	-5.61080606	ns
TARM1	0.026986759	0.174656274	1.691322274	0.090795883	0.205185018	-5.59903518	ns
CSF3R	-0.00973128	-0.0081714	-1.69099956	0.090857613	0.205185018	-5.59758972	ns
DNAJA1	0.015796206	0.037094083	1.688722042	0.091293462	0.206009977	-5.60027365	ns
OXT	-0.05454186	0.000105097	-1.68768417	0.091491981	0.20606268	-5.61911954	ns
NUDT16	-0.03028462	0.000502154	-1.68766579	0.09149561	0.20606268	-5.61638154	ns
NFASC	-0.00987477	-0.0135977	-1.68730232	0.091565314	0.20606268	-5.61972202	ns
CCS	0.017515149	0.028143193	1.687128369	0.091599095	0.20606268	-5.61204799	ns
TACC3	-0.04378701	-0.05320279	-1.68575131	0.091864102	0.206499752	-5.61469352	ns
SH3BP1	-0.01716427	0.081250058	-1.68466122	0.092074132	0.206812665	-5.62211486	ns
FKBP14	-0.03441814	-0.03884897	-1.6839401	0.092213352	0.20696617	-5.62537558	ns
SCPEP1	-0.01731075	-0.00077027	-1.68328242	0.0923412	0.207093935	-5.61004658	ns
STK24	-0.02267836	0.03087498	-1.68031625	0.09291653	0.208224303	-5.61766009	ns
PGLYRP1	-0.01546667	-0.00844634	-1.67501962	0.093950659	0.21038031	-5.63666383	ns
BNIP3L	-0.01521384	0.024587305	-1.67336198	0.094276861	0.210861732	-5.62702151	ns
FASLG	0.013754636	0.042671887	1.673189795	0.094310076	0.210861732	-5.64325058	ns
BAG4	-0.01682601	0.03790814	-1.6718846	0.094567889	0.211268539	-5.62947281	ns
CD4	-0.01082604	-0.02624323	-1.67153374	0.094636729	0.211268539	-5.63981952	ns
HPSE	-0.03198346	-0.08430357	-1.67104432	0.094733427	0.211322847	-5.63879982	ns
PDIA5	-0.02065969	0.027333797	-1.66934568	0.095069727	0.211860727	-5.63254587	ns
UROS	0.023625597	0.06859401	1.669092988	0.095119662	0.211860727	-5.63608686	ns
HIF1A	-0.02261982	0.100497978	-1.66564548	0.09580488	0.213121788	-5.63980101	ns
GPA33	0.050461152	0.084181281	1.665508533	0.095831818	0.213121788	-5.64834569	ns
TNFRSF13C	-0.01534678	-0.02108308	-1.66395065	0.096143032	0.213651183	-5.64481925	ns
STX16	-0.02159218	0.08181261	-1.66255517	0.096422346	0.214108936	-5.64623935	ns
TSC1	-0.02606321	0.165846253	-1.66090741	0.096752893	0.214584093	-5.64761859	ns
SLC44A4	-0.02334597	0.144008815	-1.6604409	0.096846529	0.214584093	-5.65037564	ns
ECI2	-0.02102021	0.081604194	-1.66039042	0.096856793	0.214584093	-5.64900559	ns
CAPN3	-0.01312834	0.041324709	-1.65766186	0.097406038	0.215637325	-5.66705065	ns
CA8	-0.00877987	0.02581641	-1.65722022	0.097495824	0.21567258	-5.65368688	ns
MSTN	-0.01886228	0.014857572	-1.65684647	0.097571341	0.215676242	-5.65654114	ns
TBR1	0.016472341	0.082587646	1.656091725	0.097724002	0.215805265	-5.65753021	ns
NFATC3	-0.02174137	0.109977462	-1.65561208	0.097821174	0.215805265	-5.6585491	ns
FDX2	-0.01957869	0.026710124	-1.6554633	0.097851429	0.215805265	-5.65710903	ns
RASSF2	-0.02125618	0.032255231	-1.65487545	0.097970627	0.215901168	-5.65887605	ns
BPIFA2	0.038012936	0.132636422	1.654290391	0.098090087	0.215901168	-5.64240409	ns
HMMR	0.016450064	0.057454686	1.65415615	0.09811673	0.215901168	-5.6562267	ns
PBK	0.010363039	-0.00099361	1.653253648	0.098300146	0.216138948	-5.65906491	ns
ACTN2	0.020099076	0.079728462	1.652896415	0.09837283	0.216138948	-5.66132115	ns
SPAG1	-0.01965796	0.053027227	-1.65093112	0.098772869	0.216854721	-5.6799891	ns
FHIT	-0.0268919	-0.02496578	-1.64916927	0.099133806	0.217219939	-5.66909823	ns
CABP2	-0.02259703	0.107420937	-1.64913213	0.099141348	0.217219939	-5.66893997	ns
GIMAP7	0.010337705	0.012774345	1.649029211	0.09916239	0.217219939	-5.66943245	ns
MIF	-0.02502564	-0.04617923	-1.64816379	0.099339555	0.217444903	-5.67885636	ns
IRAK1	-0.0254112	0.040088056	-1.64639636	0.099702984	0.217852391	-5.673643	ns
LRP1	-0.00967593	0.014044627	-1.64637058	0.099708026	0.217852391	-5.67978743	ns
ARHGAP25	-0.0216882	0.061211885	-1.64617057	0.099749537	0.217852391	-5.6731289	ns
ENTR1	-0.01978099	0.065945696	-1.64306073	0.100391219	0.218816758	-5.67822613	ns
SPP1	-0.01603323	-0.01950902	-1.6429987	0.100403507	0.218816758	-5.69144312	ns
PRDX1	-0.03092675	0.10592598	-1.64294226	0.100415909	0.218816758	-5.67543197	ns
VAMP8	-0.02971934	0.008701051	-1.63969562	0.101089391	0.220120075	-5.68234411	ns
CERT	-0.02187032	-0.00329397	-1.63819924	0.101401018	0.220562742	-5.68698579	ns
DNAJB1	-0.03198422	-0.03943587	-1.6379616	0.10145027	0.220562742	-5.69348768	ns
ITGAL	-0.01487374	-0.03994738	-1.63763258	0.10151929	0.220562742	-5.68569768	ns

SMTN	-0.03303917	0.163001878	-1.63641564	0.101772969	0.220845758	-5.70198319	ns
MINDY1	-0.03649042	-0.0426184	-1.63628263	0.101800819	0.220845758	-5.7019877	ns
LSP1	-0.0148129	-0.01668445	-1.63305675	0.10247758	0.222148874	-5.70414584	ns
GH2	0.059192991	0.507137291	1.630194932	0.103081242	0.223291711	-5.69909097	ns
ADAMTSL5	-0.01297395	0.005379238	-1.62900663	0.103331952	0.223517668	-5.71474933	ns
IL22	-0.02188156	0.027189357	-1.62879078	0.103377705	0.223517668	-5.71412329	ns
MAP4K5	-0.04050354	-0.05973833	-1.62861514	0.103415195	0.223517668	-5.70701354	ns
STAT2	-0.02589221	0.025058675	-1.62343672	0.104517045	0.225732079	-5.70865926	ns
HAO1	-0.04614056	0.020620964	-1.62257044	0.104701574	0.225846713	-5.72668626	ns
TINAGL1	-0.00781687	-0.01256524	-1.62246345	0.104724812	0.225846713	-5.71746164	ns
CRYM	-0.02477326	0.103205441	-1.62125418	0.104984046	0.226041018	-5.71448408	ns
TPPP2	0.026352503	0.081118914	1.620959837	0.105046903	0.226041018	-5.71995345	ns
HK2	-0.00945535	-0.00680668	-1.62096	0.105047144	0.226041018	-5.71488687	ns
SLC9A3R1	-0.02820653	0.006415534	-1.61996215	0.105261228	0.226334894	-5.71656395	ns
PTS	-0.01974415	0.018822638	-1.61862926	0.105548089	0.226765554	-5.71662581	ns
CRYZL1	-0.03077655	-0.00707406	-1.61830949	0.105616833	0.226765554	-5.71826546	ns
DGKA	-0.01795202	0.034660368	-1.61582082	0.106154041	0.22772057	-5.72089549	ns
TMEM106A	-0.02311481	0.019383319	-1.61552436	0.106217608	0.22772057	-5.7354762	ns
CEBPB	-0.01319081	0.014006497	-1.61423337	0.106497758	0.228153671	-5.72568118	ns
CIAPIN1	-0.02250836	-0.0048109	-1.61365432	0.106622867	0.228254231	-5.73713795	ns
ANXA10	-0.02742339	0.044104346	-1.61253412	0.106866703	0.228608625	-5.72836866	ns
PCDHB15	-0.01302509	0.013597725	-1.61024295	0.107365257	0.229405029	-5.74392828	ns
AIF1	-0.01848923	-0.01505848	-1.61010407	0.107396121	0.229405029	-5.73138142	ns
PM20D1	0.066956578	-0.5239588	1.608169204	0.107818862	0.230105584	-5.73951641	ns
ARHGAP45	-0.02722202	-0.00201376	-1.60788266	0.107881724	0.230105584	-5.73590406	ns
HBQ1	0.022172861	0.055969472	1.60752087	0.107960496	0.230105584	-5.75080403	ns
CINP	-0.01513237	0.058903026	-1.60491772	0.108532596	0.231156222	-5.74562447	ns
FABP9	-0.01980882	0.02416113	-1.6044059	0.108645192	0.231227377	-5.75016103	ns
ARHGEF5	-0.02460234	0.062920942	-1.60400098	0.108734781	0.231249498	-5.74208553	ns
IL18	-0.01624358	-0.0342467	-1.60313501	0.108925523	0.231486555	-5.74874328	ns
NIT1	-0.01579105	-0.06819289	-1.60241602	0.109084592	0.231656007	-5.74365554	ns
PIBF1	-0.02551283	0.025787118	-1.60056076	0.109494589	0.232357704	-5.76124421	ns
LAIR2	-0.03703442	-0.13744056	-1.60016494	0.109582684	0.232375771	-5.75385915	ns
CD300LG	-0.01270739	0.019753201	-1.59855574	0.109939908	0.232964101	-5.75983459	ns
BCL2L11	-0.01533678	-0.07996345	-1.59635849	0.110429372	0.233802574	-5.76293926	ns
FST	0.017863327	0.010201476	1.596060912	0.110495737	0.233802574	-5.76343162	ns
TYRO3	-0.00993679	-0.01199441	-1.59448792	0.110847439	0.234376917	-5.76364146	ns
UBE2Z	-0.01959525	0.096815073	-1.59281664	0.111222353	0.234999473	-5.75750634	ns
ADGRE5	-0.0104012	0.006426324	-1.5914124	0.111537296	0.235331152	-5.77214683	ns
HSPA2	-0.0109994	0.008452284	-1.59139788	0.111540519	0.235331152	-5.77386142	ns
IPCEF1	-0.03187379	-0.05433919	-1.59066803	0.111705045	0.23550811	-5.7678748	ns
GAS2	-0.01733811	0.061635028	-1.58963676	0.111937411	0.235795091	-5.7698401	ns
TMED8	-0.03370048	-0.03805682	-1.58934633	0.112002668	0.235795091	-5.77693164	ns
ORM1	-0.00661286	-0.00760453	-1.58602388	0.112754373	0.237206606	-5.78130364	ns
KIR2DL2	-0.02737461	-0.03595269	-1.58354532	0.113317692	0.238061819	-5.78623031	ns
MAPK13	-0.01386676	-0.01175197	-1.58352041	0.113323948	0.238061819	-5.77101521	ns
LAMP2	0.00668663	-0.01160369	1.582189188	0.113627457	0.238527802	-5.77530351	ns
PLXDC2	0.008519781	0.012825755	1.58147807	0.113789173	0.238695678	-5.79039816	ns
PCNA	0.021332778	0.143699095	1.578520153	0.114467216	0.239945635	-5.77885737	ns
HMOX2	-0.01614968	0.033659794	-1.57690682	0.114837959	0.2405501	-5.78473197	ns
GP5	-0.0116539	-0.03932689	-1.57577612	0.115098294	0.240846645	-5.78660551	ns
HYOU1	-0.0073737	-0.01521823	-1.57516417	0.115238949	0.240846645	-5.80079645	ns
NFYA	-0.0126963	0.041567128	-1.57495069	0.11528887	0.240846645	-5.78611423	ns
CGA	-0.02887222	0.196103708	-1.57455474	0.115379839	0.240846645	-5.79761608	ns
LDLRAP1	-0.03141907	-0.0308755	-1.57416087	0.115470642	0.240846645	-5.80282609	ns
MCEMP1	-0.02921922	0.065267171	-1.57414739	0.115474419	0.240846645	-5.78683244	ns
MKI67	-0.01263934	0.045300991	-1.57325183	0.115681559	0.241106461	-5.78823052	ns
IL20	-0.01732625	0.048024629	-1.5718743	0.116000648	0.241599068	-5.79260416	ns
CNP	-0.02597931	-0.0023474	-1.57136693	0.116118466	0.241668908	-5.79142569	ns
DDI2	0.025420715	0.046574761	1.571016491	0.116199708	0.241668908	-5.79403472	ns
ENAH	-0.01624463	-0.00498068	-1.57023111	0.116382298	0.241831954	-5.79516516	ns
MSR1	-0.01596739	-0.07517273	-1.56996463	0.116443742	0.241831954	-5.80937862	ns
TNFAIP8L2	-0.02015717	0.055873011	-1.56922631	0.116616105	0.242017788	-5.79682316	ns
CLIC5	-0.00993108	0.025628319	-1.56869075	0.116740948	0.242104806	-5.79733195	ns
ADRA2A	-0.02360263	0.188818132	-1.56691441	0.117155748	0.242570214	-5.79863791	ns
HNRNPUL1	-0.01612924	0.024157675	-1.5663565	0.117285564	0.242570214	-5.81498619	ns

DBH	0.03521899	-0.1734946	1.566353121	0.117286467	0.242570214	-5.81182438	ns
FZD8	-0.0134551	0.031289131	-1.56630741	0.117297651	0.242570214	-5.80136023	ns
GIPC3	-0.0315997	-0.09430871	-1.56457562	0.117702913	0.243236026	-5.81569064	ns
CR2	-0.015917	0.002131041	-1.5641919	0.117793121	0.243250292	-5.81255102	ns
CEACAM21	-0.02954293	0.246080803	-1.56359444	0.117933463	0.243367994	-5.8143011	ns
AKT1S1	-0.02288316	-0.02756892	-1.56121221	0.118494689	0.24419406	-5.80916184	ns
ADAMTS4	-0.01125792	-0.01515381	-1.56118556	0.118501022	0.24419406	-5.8069899	ns
EDAR	-0.03190144	0.052893438	-1.55886089	0.119049931	0.245152185	-5.82163352	ns
TCTN3	0.009509061	-0.02225449	1.558252634	0.119193804	0.245255349	-5.82663072	ns
GCLM	-0.01464293	0.058291975	-1.55794216	0.119268012	0.245255349	-5.81254454	ns
BAG6	-0.01238603	0.019409766	-1.55660706	0.119584812	0.245657778	-5.81631604	ns
LRG1	-0.00805493	-0.00069428	-1.55574172	0.119789946	0.245657778	-5.83020668	ns
NFAT5	-0.02809414	0.141869548	-1.55566126	0.119809711	0.245657778	-5.81553032	ns
ATP5PO	-0.0137749	-0.00259435	-1.55542733	0.119865331	0.245657778	-5.81810485	ns
FCGR3B	-0.01704608	-0.04711524	-1.55534609	0.119884361	0.245657778	-5.82432969	ns
RNASE3	-0.03771071	0.064748747	-1.55478044	0.120019129	0.245761469	-5.82357323	ns
SESTD1	-0.02196378	0.061696307	-1.55407055	0.120188591	0.245772746	-5.82019753	ns
CCL11	-0.01315062	-0.02863414	-1.55396871	0.120212256	0.245772746	-5.83413354	ns
FGF3	0.00980226	0.022298657	1.553699576	0.120277142	0.245772746	-5.81855575	ns
IL3RA	-0.01065415	-0.01526634	-1.55015417	0.121125466	0.247165135	-5.82623995	ns
YWHAQ	-0.02344299	0.026547805	-1.55014403	0.121127845	0.247165135	-5.82634889	ns
CPPED1	-0.0232353	-0.01339715	-1.54966751	0.121241746	0.24722479	-5.84079359	ns
CKMT1A_CKMT	-0.02319597	-0.0308338	-1.54914617	0.12136732	0.247308147	-5.83251575	ns
TPSAB1	-0.01788833	-0.03114601	-1.54673738	0.121946998	0.248305838	-5.84467224	ns
ICAM3	-0.00865733	-0.01010071	-1.54640573	0.122027013	0.248305838	-5.8453348	ns
NUBP1	-0.0319633	0.132931119	-1.54557979	0.122226566	0.2485387	-5.84516862	ns
DAAM1	-0.02635258	0.022401802	-1.54505479	0.122353905	0.248624497	-5.83416238	ns
CDC123	0.020743284	0.071682469	1.543883427	0.122637638	0.24902775	-5.83249928	ns
FAM172A	-0.01949985	0.025463418	-1.53892584	0.123843387	0.251301382	-5.84258303	ns
PRUNE2	-0.00926772	0.013648507	-1.53671188	0.124384603	0.252224334	-5.85191972	ns
MCEE	-0.01354211	0.031594142	-1.53634189	0.124474931	0.252232338	-5.86118937	ns
TOP1	0.035441221	-0.06258099	1.53290045	0.125320843	0.253620193	-5.86436637	ns
PCSK7	-0.01437508	0.031842952	-1.53284899	0.125333541	0.253620193	-5.86461203	ns
KLB	-0.02506467	0.004789808	-1.53064555	0.12587801	0.25454556	-5.85604004	ns
ARHGEF1	-0.02839435	-0.03548987	-1.52796318	0.126542086	0.255583067	-5.87295435	ns
JPT2	-0.02744895	-0.03595741	-1.52786652	0.126566135	0.255583067	-5.87201044	ns
KLK7	-0.01226715	-0.00131242	-1.52733472	0.126699234	0.255675026	-5.84854071	ns
M6PR	-0.0124859	0.065963534	-1.52346083	0.127664424	0.257444834	-5.86607443	ns
CXCL8	-0.02248488	-0.00524954	-1.5227136	0.127851063	0.257643274	-5.87531884	ns
CANT1	-0.00663737	-0.01148163	-1.52012603	0.128499958	0.258423104	-5.87853708	ns
CCL8	-0.02051638	-0.08009675	-1.51982935	0.128574267	0.258423104	-5.88627186	ns
ADAMTS1	-0.00970798	-0.00245939	-1.51954129	0.128647331	0.258423104	-5.87062501	ns
CNTF	-0.01065477	-0.00629605	-1.51952663	0.128650956	0.258423104	-5.87296541	ns
PVALB	-0.03828233	-0.19307949	-1.519407	0.128680546	0.258423104	-5.88541077	ns
GPKOW	-0.00958512	-0.00350804	-1.51873399	0.128850447	0.258586463	-5.87405716	ns
VAV3	-0.03123709	0.016715733	-1.51791032	0.129057974	0.258825057	-5.87540064	ns
CA6	0.021331533	0.055544365	1.515858624	0.129575747	0.259685093	-5.88588338	ns
APBB1IP	-0.01567576	0.008427928	-1.51527049	0.129724578	0.259805054	-5.88540445	ns
STAM	-0.01166568	0.041060788	-1.51378329	0.130101267	0.260380877	-5.89342989	ns
SH3GL3	-0.02687934	-0.01478156	-1.51308546	0.130278785	0.26055757	-5.88421802	ns
ERP44	-0.00868277	-0.00243641	-1.51252988	0.130419808	0.260661081	-5.88883671	ns
FKBP4	-0.01477424	-0.02131707	-1.51071012	0.130883014	0.261407936	-5.89190703	ns
COL24A1	-0.01690749	-0.05964221	-1.50850486	0.131445795	0.26235251	-5.90117704	ns
MYH7B	0.013425215	0.05545574	1.506899767	0.131857377	0.262994222	-5.88846251	ns
STIP1	-0.02213917	0.019212221	-1.5061028	0.132061388	0.263221333	-5.90115203	ns
CLNS1A	0.010644348	0.035504115	1.505094093	0.132320782	0.263558448	-5.89429596	ns
FNDC1	-0.0143562	-0.03292912	-1.50429961	0.132524614	0.263784508	-5.90855377	ns
GIPR	0.01376616	0.022491418	1.502527781	0.132981861	0.264514327	-5.89612788	ns
TTF2	-0.01529545	0.04208929	-1.50118915	0.133327533	0.265021373	-5.90011901	ns
GRSF1	-0.02498605	0.073775296	-1.50030808	0.133555755	0.265263353	-5.89532386	ns
SERPINA3	-0.00440561	-0.00450974	-1.50001511	0.133630956	0.265263353	-5.91276932	ns
PTX3	-0.01638222	-0.07781515	-1.49694446	0.134428278	0.266664791	-5.91549745	ns
VSTM2B	-0.01311506	0.074227913	-1.49538326	0.134835117	0.267290252	-5.9140833	ns
GGCT	-0.01495717	0.019026555	-1.49480502	0.134985747	0.267407313	-5.9219967	ns
CREB3	-0.01684743	0.072483621	-1.49323875	0.135395702	0.268037593	-5.90879114	ns
CSF2	-0.01115615	0.012711188	-1.4920979	0.135694467	0.268447049	-5.91215772	ns

RPE	-0.0222592	0.079024553	-1.48885378	0.136546533	0.269949815	-5.92374951	ns
ANP32C	-0.01271809	0.067964884	-1.4884496	0.136653278	0.26997806	-5.91838031	ns
ADCYAP1R1	0.011142071	0.027351203	1.486316923	0.137216271	0.270907039	-5.92150346	ns
CALCOCO1	-0.03442348	0.011091652	-1.48557174	0.13741282	0.27092976	-5.9359987	ns
IDS	0.002867642	-0.00462316	1.485569406	0.137413348	0.27092976	-5.93728168	ns
PDAP1	-0.02972931	-0.03326092	-1.48419786	0.137776788	0.271463038	-5.93841287	ns
CSF1R	-0.01187332	0.001406766	-1.4804608	0.138770668	0.273201079	-5.94274269	ns
EPCAM	0.032488568	0.117250887	1.480161679	0.138850657	0.273201079	-5.93836422	ns
TP73	-0.01039245	-0.00385965	-1.47982952	0.13893959	0.273201079	-5.93170288	ns
CEACAM3	-0.01507964	0.01329305	-1.4769849	0.139700619	0.274512657	-5.93522032	ns
TIE1	-0.01355908	-0.07811984	-1.47545267	0.140111183	0.275134266	-5.95171116	ns
B3GNT7	-0.01094825	-0.0376696	-1.47302326	0.140765638	0.276233644	-5.94104103	ns
GLOD4	-0.01172035	-0.01595977	-1.47081177	0.1413626	0.2772188	-5.95124999	ns
RBPMS	-0.01992372	0.131082483	-1.47005038	0.141569035	0.277437304	-5.94401377	ns
CD28	-0.0114941	0.002871527	-1.46932226	0.141766316	0.277637587	-5.94641883	ns
TAB2	-0.03264342	0.011923119	-1.46826976	0.142051564	0.278009763	-5.95394521	ns
CFP	-0.00692487	-0.0121674	-1.46711158	0.142366	0.278438526	-5.96312753	ns
PQBP1	-0.01866141	0.044443246	-1.46596847	0.142677778	0.278861521	-5.95130187	ns
GOLGA3	-0.02331992	0.05655925	-1.46529991	0.142859364	0.279029661	-5.96698747	ns
BCL7B	-0.0140472	0.057095201	-1.46411242	0.14318358	0.279475971	-5.96681418	ns
IL1RL2	-0.01254627	-0.01005191	-1.46231964	0.143674441	0.280246738	-5.95748883	ns
NADK	-0.01762777	0.003413089	-1.4610092	0.144033441	0.280759444	-5.96596445	ns
TCL1B	-0.02532835	0.143829201	-1.46018666	0.144259633	0.280914898	-5.96060113	ns
BCAT2	-0.01659282	0.087976137	-1.46001932	0.144305598	0.280914898	-5.96091414	ns
ARFIP1	-0.0207429	0.050202676	-1.4586034	0.144694529	0.281484359	-5.97670192	ns
IL7	-0.02071273	0.010567027	-1.4570211	0.145131019	0.282145524	-5.97400393	ns
CDC27	-0.01952706	0.075676815	-1.45470184	0.145772688	0.283204423	-5.96762417	ns
SNRPB2	-0.01317863	0.03765377	-1.45395453	0.145979161	0.283416987	-5.98244479	ns
PFKFB2	-0.02478578	0.001772355	-1.45167329	0.14661301	0.284458464	-5.97832435	ns
UBXN1	-0.02067087	0.028770496	-1.44992986	0.147098786	0.284911978	-5.97548039	ns
PDIA4	-0.01807384	0.038371811	-1.44980111	0.147134245	0.284911978	-5.98792624	ns
SFTPA1	-0.01798107	0.089518938	-1.44962063	0.147185186	0.284911978	-5.97494427	ns
ENPEP	0.013001191	0.057241602	1.44943456	0.147237124	0.284911978	-5.9744059	ns
CD3D	-0.00969824	0.032253359	-1.44908509	0.147334619	0.284911978	-5.97669242	ns
FOS	0.010672175	-0.02025912	1.448643733	0.147458023	0.284961898	-5.97244064	ns
CPB2	0.006705482	-0.01355439	1.447908116	0.14766303	0.285169344	-5.98929511	ns
OSBPL2	-0.01139402	0.06493277	-1.44556269	0.148320115	0.286248998	-5.99451693	ns
MST1	-0.01958244	-0.17445556	-1.44357273	0.148879785	0.286995605	-5.98878083	ns
ILKAP	-0.01842166	0.019983732	-1.44348919	0.148903542	0.286995605	-5.98386896	ns
NAGA	-0.0176015	0.083597461	-1.44110855	0.149574886	0.288099384	-5.98486772	ns
SCN2A	-0.01390949	0.04412435	-1.43814816	0.150412774	0.28952228	-5.99235597	ns
CD1C	-0.00740094	-0.00175217	-1.43704179	0.150726387	0.289934815	-6.00443848	ns
CD163	-0.0137959	-0.02174901	-1.43616374	0.150976129	0.290060278	-6.00311551	ns
ACRV1	-0.02177385	0.3736473	-1.4361155	0.150990282	0.290060278	-5.99293108	ns
TXNDC15	-0.00836671	0.000448018	-1.43528386	0.151226486	0.290323037	-6.00694356	ns
PPP1R14D	-0.01100219	0.021593085	-1.43415195	0.15154905	0.290751134	-6.01179528	ns
GSAP	-0.0278588	0.246874791	-1.43317099	0.15182976	0.291098424	-5.99846637	ns
LPA	0.05274661	-0.13674861	1.430854572	0.152492585	0.292177395	-6.00180553	ns
CD2AP	-0.02018735	0.014470425	-1.42947296	0.152888746	0.292659348	-6.01118528	ns
LAMTOR5	-0.01212508	0.028809154	-1.42927796	0.152944577	0.292659348	-6.01681896	ns
TNFSF12	0.008922687	0.020499813	1.427987232	0.153315639	0.293177254	-6.02051916	ns
CHAC2	0.018737924	0.03139876	1.427165954	0.153552822	0.293399375	-6.00698638	ns
AP3S2	-0.01395889	0.074404045	-1.42658746	0.153718983	0.293399375	-6.02155632	ns
TANK	-0.0218542	0.03529172	-1.42653987	0.153733234	0.293399375	-6.00876038	ns
ANXA4	-0.02244523	0.023679777	-1.4258885	0.153921021	0.293565892	-6.01433896	ns
OTUD6B	-0.01718895	0.022561018	-1.42423087	0.154399831	0.293831099	-6.02379641	ns
CTHRC1	-0.00812465	-0.02702126	-1.42394339	0.154483013	0.293831099	-6.02479322	ns
IFIT3	-0.01105699	0.031379092	-1.42385506	0.154508871	0.293831099	-6.01766758	ns
PYDC1	-0.0121046	0.02500071	-1.42371466	0.154549781	0.293831099	-6.0118907	ns
ANKMY2	-0.02362769	0.016214311	-1.4236685	0.154563208	0.293831099	-6.01058723	ns
TNFAIP8	-0.01109539	0.006146925	-1.4211382	0.155297228	0.294873978	-6.01639052	ns
PDCD5	-0.01869107	-0.00980959	-1.42101951	0.155331196	0.294873978	-6.03040528	ns
PSIP1	-0.01979688	0.035757826	-1.4206173	0.155448365	0.294873978	-6.02597451	ns
STEAP4	0.021341494	0.136177115	1.420386979	0.155515728	0.294873978	-6.01754026	ns
SORBS1	-0.01152938	0.031570506	-1.41905839	0.155902073	0.2954147	-6.03316189	ns
GPC5	0.017446324	0.023330055	1.418434687	0.156084302	0.295568199	-6.02599142	ns

CRX	-0.0341526	0.339559902	-1.41751728	0.156352427	0.295884048	-6.02150928	ns
PSAPL1	-0.01421867	0.009602024	-1.41668028	0.156596571	0.296154136	-6.03443869	ns
MDGA1	-0.02182544	-0.03926383	-1.414859	0.157130233	0.296971056	-6.03101723	ns
CLEC5A	-0.00872094	-0.01994965	-1.41305565	0.157659778	0.297727926	-6.03222693	ns
MAP1LC3A	-0.00904774	-0.01068705	-1.41261711	0.157788975	0.297727926	-6.02845909	ns
CD226	-0.01199367	0.018159776	-1.41245549	0.157836585	0.297727926	-6.02636138	ns
RPA2	-0.00896922	0.022340899	-1.41094259	0.158282232	0.298375801	-6.02848014	ns
KLK1	0.039451413	0.43172829	1.409904516	0.158588277	0.298609489	-6.0374975	ns
CDHR1	-0.01311494	0.027509691	-1.40982932	0.158610725	0.298609489	-6.03225903	ns
HRG	-0.0092035	-0.0149451	-1.40840345	0.15903184	0.299209389	-6.04340355	ns
TACSTD2	0.009553196	0.012061232	1.407046513	0.159436209	0.299777031	-5.99074911	ns
FCAR	-0.0146939	-0.03028272	-1.40503927	0.160029844	0.300576696	-6.0478123	ns
TP53I3	-0.01540975	-0.00374249	-1.40491554	0.160067385	0.300576696	-6.02732432	ns
IL13RA2	-0.00817412	-0.01318803	-1.40321405	0.160573612	0.30114747	-6.04159294	ns
RRM2	-0.0156445	0.037135017	-1.40320081	0.160577606	0.30114747	-6.04063221	ns
RAPGEF2	-0.01528182	0.06697825	-1.40268653	0.160730671	0.301195107	-6.04734984	ns
ANGPTL4	-0.01404979	-0.05190204	-1.40238066	0.160821786	0.301195107	-6.05153643	ns
IDI2	-0.01427127	0.025150723	-1.40207834	0.160912455	0.301195107	-6.04307022	ns
AHCY	-0.02071597	-0.01930193	-1.40084587	0.16128044	0.301690508	-6.05138043	ns
KLK14	-0.01417036	0.00705239	-1.40025161	0.161457976	0.30182925	-6.06010952	ns
LAMP1	0.008027222	0.021496303	1.398557486	0.161966346	0.302585879	-6.04645315	ns
EPN1	-0.01249491	0.069220627	-1.39750621	0.162281706	0.30298119	-6.05454591	ns
MAN2B2	-0.01697754	-0.06338169	-1.39572046	0.162818698	0.303789519	-6.06483267	ns
DNAJA4	0.020883301	0.047081841	1.3951634	0.16298656	0.303908529	-6.06554952	ns
ITGB7	-0.01399752	0.023779023	-1.39388564	0.163372099	0.304433011	-6.06893481	ns
LPL	0.014655943	0.009287675	1.392853738	0.163684251	0.304820162	-6.06245698	ns
CLEC4M	0.013778635	0.040276335	1.390881301	0.16428145	0.305737307	-6.07000109	ns
MFGE8	-0.01319499	-0.00135325	-1.39015732	0.164501243	0.305951357	-6.06882928	ns
ARF6	-0.03052252	-0.07728748	-1.38898693	0.164856728	0.306417343	-6.07298737	ns
GALNT3	-0.01295547	0.001683694	-1.3862349	0.165695649	0.307749134	-6.06499569	ns
GUK1	-0.01210129	0.021779593	-1.38594572	0.165784037	0.307749134	-6.06369764	ns
COMP	0.010302102	-0.03413648	1.384229607	0.166308602	0.308526758	-6.06850968	ns
FIS1	-0.02347627	0.041544459	-1.38138776	0.167179904	0.309946234	-6.07894534	ns
IL3	0.017051382	-0.06562363	1.38072632	0.167383666	0.310127096	-6.06917886	ns
VASP	-0.02039797	0.021510264	-1.37921607	0.16784807	0.310748816	-6.0863858	ns
ACE2	-0.01443171	0.018380216	-1.37894538	0.167932066	0.310748816	-6.07501451	ns
FAP	0.008037747	-0.00573277	1.378474254	0.168076792	0.310819654	-6.08902829	ns
ENPP2	-0.00795228	0.021937929	-1.37733407	0.16842886	0.31127359	-6.09058725	ns
SAA4	-0.00919106	0.03625041	-1.37562779	0.168956784	0.312051746	-6.09215532	ns
LEG1	0.019078584	0.056077238	1.373560137	0.169599292	0.313040412	-6.06595461	ns
RAB33A	-0.01905465	0.04945868	-1.37284907	0.169819753	0.313249323	-6.0816196	ns
SERPINB5	-0.01430783	-0.03698574	-1.36967399	0.170809103	0.314875367	-6.0876554	ns
COPB2	-0.01213135	0.014551357	-1.36864022	0.171132219	0.315113871	-6.08734303	ns
TMED1	-0.01525588	0.028285274	-1.36856856	0.171154315	0.315113871	-6.09425414	ns
CD22	-0.01243266	-0.00931444	-1.36527252	0.172187187	0.316658652	-6.10743382	ns
GPRC5C	-0.01529138	0.065085458	-1.36498642	0.172277194	0.316658652	-6.10594829	ns
IL7R	0.015938773	0.016943953	1.364625853	0.172390653	0.316658652	-6.10372285	ns
ATP6V1F	-0.02011544	0.046970394	-1.36451125	0.172427143	0.316658652	-6.09332297	ns
BHMT2	-0.00720232	-0.01491583	-1.36384888	0.172635554	0.316842124	-6.09383718	ns
PLAU	-0.00840295	-0.0023092	-1.36201597	0.173212808	0.31745804	-6.10725333	ns
MAPKAPK2	-0.02067353	0.020175084	-1.36175032	0.173296789	0.31745804	-6.10348922	ns
CDK5RAP3	-0.01668908	0.097683472	-1.36174765	0.1732973	0.31745804	-6.11223332	ns
GET3	-0.01574301	0.09234847	-1.35975203	0.1739282	0.318414008	-6.11400549	ns
IL11	0.01917764	0.099355037	1.358639186	0.174281238	0.318745179	-6.10256925	ns
TPM3	0.012061534	0.046215884	1.358493861	0.174327415	0.318745179	-6.10106839	ns
NFATC1	-0.02917686	0.017820846	-1.35772654	0.174570439	0.318989789	-6.11266102	ns
TIGIT	-0.00998164	0.007857633	-1.35712405	0.174761862	0.319024228	-6.10972908	ns
MYL1	0.011181323	0.036114753	1.356980455	0.174807796	0.319024228	-6.10310688	ns
ATP1B2	-0.008659	0.018758175	-1.35653008	0.174950943	0.319086043	-6.10371307	ns
S100A3	-0.01214268	0.060646191	-1.35530851	0.175339096	0.319492442	-6.11900999	ns
CHMP1A	-0.02364439	-0.05802189	-1.35514127	0.175392598	0.319492442	-6.11307901	ns
SSC4D	-0.03641596	0.095505149	-1.35388053	0.175794515	0.319973712	-6.11496681	ns
AFAP1	0.02356192	0.1946143	1.353625895	0.175875961	0.319973712	-6.10940796	ns
C1S	-0.00587869	-0.01995883	-1.35281949	0.176133045	0.3202419	-6.1230309	ns
HSBP1	-0.02652914	0.031978279	-1.34964164	0.177151224	0.321883241	-6.1137913	ns
EGF	-0.03094415	-0.08533551	-1.34931354	0.177256251	0.321883241	-6.12158939	ns

EFHD1	-0.00929271	-0.0017446	-1.34857495	0.177493206	0.322113214	-6.12994712	ns
INPP1	-0.01600711	0.027317281	-1.34796906	0.177688292	0.322266965	-6.12304122	ns
PRDX6	0.017802403	0.071347133	1.33881969	0.180648988	0.327161787	-6.14359603	ns
TSHB	0.022815454	0.028700225	1.338721856	0.180681118	0.327161787	-6.13581223	ns
HTR1B	-0.01182995	0.067602951	-1.33859335	0.180723275	0.327161787	-6.12948053	ns
CTAG1A_CTAG	-0.01388205	0.01151326	-1.3375696	0.181056756	0.327562408	-6.13443759	ns
RYR1	-0.00957629	0.028298676	-1.3358605	0.181615097	0.328369093	-6.13131746	ns
NAGPA	0.005821203	-0.00723809	1.335085635	0.181868387	0.328623571	-6.13413552	ns
FRMD7	0.008955584	-0.00560272	1.332006363	0.182878181	0.330243839	-6.13788275	ns
KRT6C	-0.01037397	0.040945078	-1.33131326	0.183106147	0.330451144	-6.13733348	ns
JUN	-0.01533196	0.052226357	-1.32982253	0.183596828	0.33093303	-6.14099903	ns
IFT20	-0.01225824	0.054336889	-1.32981338	0.183599832	0.33093303	-6.14077831	ns
DSG4	0.014685177	0.019924538	1.329136743	0.183822723	0.331130383	-6.14688519	ns
CETN2	-0.01981269	0.059669333	-1.32847332	0.184041911	0.331320826	-6.14276594	ns
RAB6B	-0.01354718	0.037101954	-1.32535738	0.185072742	0.332687093	-6.14686783	ns
GIT1	-0.02683796	0.003260664	-1.32521487	0.185120033	0.332687093	-6.14482982	ns
STAB2	-0.00797225	-0.03118137	-1.32514491	0.185142646	0.332687093	-6.15954761	ns
CLPS	-0.01983338	0.003085875	-1.32433231	0.185412444	0.332966996	-6.15642289	ns
SEZ6	-0.00837387	0.016932741	-1.32356764	0.185666697	0.333218657	-6.14899893	ns
CASP1	-0.01982657	-0.00098782	-1.32211724	0.186149184	0.333879372	-6.15112301	ns
SLC27A4	-0.01960564	0.076762618	-1.32133735	0.186408967	0.334140075	-6.15214565	ns
VTCN1	-0.00875902	0.000449847	-1.31891249	0.187218447	0.335385193	-6.15444378	ns
GPNMB	0.008119397	-0.01349286	1.318102886	0.187489511	0.33566485	-6.14984307	ns
IDO1	-0.01718645	0.01788566	-1.31766589	0.187635538	0.335720448	-6.15609799	ns
GID8	-0.00902534	0.040262802	-1.31417727	0.188805975	0.337607745	-6.1753912	ns
PSMA1	-0.01373681	0.049360822	-1.31339692	0.189069269	0.337871644	-6.16164563	ns
WASL	0.015387003	0.092964813	1.30902075	0.190547328	0.340304708	-6.16598997	ns
CLSTN1	-0.01485125	0.03174935	-1.3066304	0.191358209	0.341478184	-6.17131756	ns
CHEK2	-0.00930435	-0.00418134	-1.30639478	0.191438283	0.341478184	-6.17165542	ns
CACNA1H	0.015482437	0.101908115	1.305788121	0.191644603	0.341627079	-6.17072263	ns
TSPAN1	-0.0158774	0.154218891	-1.30546131	0.191755747	0.341627079	-6.17286483	ns
CST6	-0.01358011	-0.00076379	-1.30494123	0.19193301	0.341734383	-6.16668863	ns
PLAT	-0.0153784	-0.09331766	-1.30442982	0.192106506	0.341834856	-6.18550678	ns
BRD2	-0.00917048	0.017556094	-1.30383263	0.19231056	0.341989547	-6.17504375	ns
ARAF	-0.0179795	0.068298952	-1.30291617	0.192623034	0.342336737	-6.18126234	ns
DSG3	-0.00955628	-0.00361557	-1.30188194	0.192976154	0.3427557	-6.18966939	ns
HEPACAM2	0.011101679	0.026025487	1.296532891	0.194811257	0.345450209	-6.19726809	ns
SSH3	-0.00795003	0.020138762	-1.29647906	0.194830403	0.345450209	-6.18105425	ns
ACADSB	-0.02328282	0.063970581	-1.29642588	0.194848115	0.345450209	-6.19746035	ns
SLC12A2	-0.00703395	0.01478247	-1.29577653	0.19507238	0.345637955	-6.18364224	ns
IL10	-0.02319868	0.056158783	-1.29418097	0.195622702	0.346402844	-6.18739114	ns
NUP50	0.008580347	0.034373407	1.293265922	0.195938976	0.346752612	-6.1868665	ns
ZNRF4	-0.01438567	0.023850275	-1.28758977	0.197908088	0.350025207	-6.20780621	ns
CEP43	-0.01924893	0.019300825	-1.28693504	0.198136828	0.35020255	-6.19405477	ns
CBLN4	0.008722406	-0.00472011	1.286613507	0.198248224	0.35020255	-6.21160186	ns
PECAM1	-0.00851722	-0.00117818	-1.28574484	0.198551497	0.350526222	-6.20744352	ns
CELA2A	-0.01538652	0.010258095	-1.28310811	0.199473407	0.351940996	-6.21394634	ns
GSTM4	-0.01903563	0.149705727	-1.28140985	0.200069513	0.352779576	-6.20200826	ns
DCTN6	-0.02016659	0.086028779	-1.2791489	0.200864323	0.353967304	-6.20656732	ns
FGFBP3	-0.00954274	-0.01857842	-1.27845833	0.201107012	0.354181228	-6.22035988	ns
NCAN	-0.01091491	0.031964853	-1.27797483	0.201277432	0.354267693	-6.2219586	ns
SLC9A3R2	-0.01321678	0.062944497	-1.27520564	0.202255624	0.355519893	-6.22338452	ns
DXO	-0.01192791	0.016189572	-1.27494519	0.202347754	0.355519893	-6.22481059	ns
KIR2DL3	-0.0212596	0.005696045	-1.27492881	0.202354131	0.355519893	-6.21103548	ns
TXNDC9	-0.0137978	0.074873574	-1.27393913	0.20270409	0.355920592	-6.22608289	ns
THSD1	-0.00882731	0.00957023	-1.27279321	0.203111298	0.356368398	-6.2084965	ns
LY9	-0.00804657	-0.01034892	-1.27253326	0.203203213	0.356368398	-6.21713268	ns
RTBDN	-0.00805041	0.002371294	-1.27194465	0.203412122	0.356520645	-6.22185239	ns
FAM3B	-0.00976414	-0.03352769	-1.27103466	0.203735644	0.356749378	-6.22300041	ns
PMCH	-0.0213734	0.231961657	-1.27089134	0.203786974	0.356749378	-6.21532506	ns
CLGN	-0.01130684	0.023910683	-1.2685877	0.20460769	0.357971513	-6.21969379	ns
BLOC1S3	-0.01069329	0.066570655	-1.26802745	0.204807192	0.358105989	-6.23353871	ns
CD82	-0.01095027	0.063498084	-1.26750674	0.204993485	0.358217222	-6.22641509	ns
ITGAM	0.008654257	0.011571648	1.265415467	0.205741617	0.359309522	-6.23317422	ns
FSHB	-0.01704642	0.487550514	-1.26491923	0.205919831	0.359405802	-6.22367019	ns
STAMBP	-0.01820692	0.012628826	-1.26351792	0.206422445	0.359903301	-6.23081868	ns

KIR3DL1	-0.03403277	8.5951209784	-1.26316003	0.206551254	0.359903301	-6.22585242	ns
CHM	-0.0157875	0.04361748	-1.26309469	0.206574634	0.359903301	-6.22659212	ns
ZHX2	-0.00879798	0.027861721	-1.26217961	0.206903155	0.360260712	-6.24087992	ns
TFF2	-0.01524081	-0.01632929	-1.26100022	0.207327891	0.360785127	-6.23834887	ns
POMC	-0.01786022	-0.02480853	-1.25991764	0.207718576	0.361161151	-6.22993435	ns
EPPK1	-0.0187832	0.027879247	-1.25971595	0.207791347	0.361161151	-6.23018642	ns
CPXM1	-0.01898916	-0.0013521	-1.2583127	0.208297723	0.361698264	-6.24344236	ns
PGLYRP4	-0.01607847	0.107113057	-1.25817474	0.20834811	0.361698264	-6.23127818	ns
MUCL3	0.01165482	0.075296659	1.257684385	0.208525471	0.361791072	-6.23020415	ns
FMR1	-0.00929435	-0.00868669	-1.25071268	0.211058777	0.365823516	-6.24137418	ns
RAB3GAP1	-0.01490554	0.078988306	-1.25059902	0.211100214	0.365823516	-6.24249754	ns
DNAJB8	0.015425528	0.109921576	1.249669583	0.211439685	0.366174982	-6.24357935	ns
NEDD9	-0.0086283	0.020931097	-1.24935739	0.211553834	0.366174982	-6.241705	ns
C9orf40	0.021260549	0.072596166	1.2485955	0.211831962	0.366439176	-6.25670496	ns
SPRING1	-0.01601644	0.102760336	-1.24800021	0.21205043	0.366599914	-6.24224043	ns
SOD1	-0.01307785	0.008830319	-1.2474864	0.212238715	0.366708312	-6.24317826	ns
OFD1	-0.01671661	0.153501516	-1.24662618	0.212553723	0.367035406	-6.25245822	ns
MAP2K1	-0.01643965	0.02138386	-1.24604444	0.212766958	0.367186475	-6.25986242	ns
ANPEP	0.007600079	0.002068346	1.245304416	0.213038916	0.36743865	-6.25610013	ns
ANGPTL2	-0.01138683	-0.03470297	-1.24338378	0.21374541	0.36843955	-6.2598597	ns
FLT4	-0.00940772	-0.05505408	-1.24220943	0.214178174	0.368967709	-6.26342008	ns
LMOD2	-0.01645408	0.087807676	-1.24099611	0.214626493	0.369522027	-6.2543664	ns
KIAA2013	-0.01492085	0.130391587	-1.24056905	0.214783828	0.369575002	-6.26678597	ns
CCL26	-0.02444604	0.034622279	-1.2388236	0.215429783	0.370468179	-6.26583142	ns
ANXA3	-0.01919714	-0.01449547	-1.23671782	0.216210617	0.371592113	-6.27258931	ns
CCAR2	0.013864134	0.095377742	1.235424912	0.21669113	0.372198883	-6.27403427	ns
RAD23B	-0.01223115	0.020030052	-1.23301681	0.21758876	0.373520976	-6.26317626	ns
SERPIND1	-0.00730117	-0.03243131	-1.23267124	0.217717192	0.373521858	-6.2755431	ns
RABEPK	-0.0161949	0.093189341	-1.23230882	0.217853007	0.373535397	-6.26404208	ns
CTSE	-0.01307576	0.016807315	-1.23125377	0.218247084	0.373991483	-6.26631697	ns
ADAM12	-0.0087554	0.001891794	-1.22904139	0.219075523	0.375190925	-6.26668096	ns
IZUMO1	0.012021957	0.059786932	1.228623456	0.219232263	0.375239277	-6.2660442	ns
COL9A2	0.011661178	0.034086214	1.227920211	0.219495852	0.37547035	-6.27542182	ns
PLPBP	-0.0183295	0.002447923	-1.22749042	0.219657197	0.375526355	-6.27742132	ns
PGD	-0.01364756	-0.00138277	-1.22671248	0.219949322	0.375652893	-6.28473209	ns
KLKB1	0.006304037	0.002368563	1.226609388	0.219988509	0.375652893	-6.27411873	ns
ZBTB16	-0.01937688	0.114089428	-1.22388618	0.221014339	0.377184026	-6.27430413	ns
TRIM40	-0.01802211	0.036227833	-1.21981743	0.222553123	0.379588271	-6.27989463	ns
ASPN	-0.00662832	0.007381007	-1.21692043	0.223653033	0.380934416	-6.29660422	ns
PTGES2	-0.01348798	0.00635611	-1.21678468	0.223705425	0.380934416	-6.27799096	ns
TBCA	-0.02143328	0.01215821	-1.21670945	0.223733741	0.380934416	-6.2839785	ns
FGF19	-0.02086857	0.02515215	-1.21572791	0.224107206	0.381347926	-6.29514175	ns
CPB1	-0.01521903	0.031093953	-1.21493704	0.224408597	0.381638383	-6.29945685	ns
ITPR1	-0.01746623	0.061217129	-1.21441094	0.22460986	0.381758318	-6.28578846	ns
LATS1	-0.02110711	-0.00192792	-1.20917904	0.226613081	0.384939032	-6.29070343	ns
NXPH1	-0.01014247	0.010402595	-1.20863613	0.226821634	0.385069285	-6.2935852	ns
PPT1	-0.01945114	0.199648092	-1.20779579	0.227144717	0.385393709	-6.2943714	ns
LYZL2	0.008847442	0.034659601	1.205959244	0.227852197	0.38636958	-6.29341399	ns
NRXN3	-0.00889657	0.05584233	-1.20434804	0.228473944	0.387199023	-6.29849683	ns
ATF2	-0.01853198	0.154533829	-1.20356221	0.22877774	0.387414408	-6.29965658	ns
VNN1	-0.0182013	-0.10123829	-1.2033317	0.228866388	0.387414408	-6.31174826	ns
TNC	0.012541645	0.00349746	1.202605195	0.229147683	0.387665837	-6.30887162	ns
SV2A	-0.01513208	0.061479963	-1.19978576	0.23024136	0.389290546	-6.30393771	ns
VNN2	-0.01235766	-0.07590843	-1.19648604	0.231525487	0.391235198	-6.31425862	ns
MDH1	-0.00999878	0.01677717	-1.1944403	0.232324201	0.392357817	-6.31932319	ns
SMS	-0.01563604	0.122758021	-1.19304582	0.232870199	0.392876214	-6.31226257	ns
PDP1	-0.01338249	0.069024917	-1.19296921	0.232900249	0.392876214	-6.3113698	ns
PACS2	-0.01537939	0.04715147	-1.19127537	0.23356431	0.39376893	-6.31256275	ns
SEC31A	-0.01891958	-0.07002778	-1.18938494	0.234306955	0.394793022	-6.3156295	ns
ACY1	-0.01636721	-0.00095489	-1.18875462	0.234554682	0.394982509	-6.32385467	ns
BRD1	-0.00756221	0.026491922	-1.18766814	0.234982656	0.395345122	-6.3236585	ns
CRTAP	-0.01235045	0.062242733	-1.1875211	0.235040799	0.395345122	-6.31846197	ns
PSRC1	-0.01535789	0.098925761	-1.18557957	0.235807101	0.396251506	-6.32008868	ns
CETN3	-0.01879826	0.020868665	-1.18516201	0.23597161	0.396251506	-6.3333012	ns
CXCL13	-0.01531492	0.011000087	-1.18512353	0.23598677	0.396251506	-6.33543209	ns
CDON	-0.00685424	-0.01290001	-1.18348169	0.236636617	0.397114324	-6.33139473	ns

TSLP	-0.01615174	0.094718357	-1.18054191	0.23780339	0.398843136	-6.32691411	ns
ACE	-0.00628768	-0.01228102	-1.17945447	0.238235383	0.3993383	-6.3399764	ns
INPP5D	-0.01418194	0.010741509	-1.17591544	0.239647233	0.401474423	-6.33143338	ns
LIFR	0.006031381	-0.0012579	1.175342488	0.23987596	0.401515335	-6.34186471	ns
ITGA2	0.008111499	0.018631455	1.174895474	0.240055008	0.401515335	-6.33358551	ns
VSIG10	-0.00830723	0.022119706	-1.17482168	0.24008417	0.401515335	-6.34557742	ns
CLEC1A	-0.00812236	-0.0125372	-1.17439183	0.240256678	0.40157384	-6.33409853	ns
ALDH1A1	-0.01393626	0.032720692	-1.17074748	0.241718445	0.403785961	-6.34759461	ns
AHSP	0.022411339	-0.00631884	1.169536962	0.242205539	0.40436831	-6.34794379	ns
VASH1	-0.02078781	0.036014577	-1.168366	0.242677317	0.404924438	-6.34997685	ns
AGRP	-0.01343761	-0.06768214	-1.16693	0.243256553	0.405659129	-6.35662694	ns
PAMR1	-0.0074304	-0.03393919	-1.16644198	0.243453895	0.405756491	-6.35258709	ns
BLOC1S2	-0.01758891	0.087829121	-1.16428014	0.244328925	0.406982579	-6.34590037	ns
TNN	-0.01028453	0.013738185	-1.16359772	0.244605006	0.407052671	-6.35902465	ns
SCN2B	0.00689508	0.011103532	1.163185222	0.244772723	0.407052671	-6.34683963	ns
MAPT	0.012934544	-0.05906622	1.162901351	0.244887945	0.407052671	-6.34738846	ns
ANKRA2	0.01355422	0.03782801	1.162801273	0.244928611	0.407052671	-6.34412364	ns
UNC79	-0.01351364	0.06290999	-1.16060901	0.24581938	0.408300677	-6.34665019	ns
TET2	-0.02402504	0.202701237	-1.15766048	0.247020761	0.409882324	-6.35757593	ns
AFP	-0.01730744	0.046349366	-1.15758316	0.247052359	0.409882324	-6.35757048	ns
CCDC134	-0.01134503	0.025800602	-1.15631565	0.247569948	0.410378662	-6.36698154	ns
CC2D1A	-0.01939241	0.043907301	-1.15616294	0.247632604	0.410378662	-6.36097667	ns
CASP4	-0.04029186	0.424528505	-1.15472802	0.248219895	0.411118602	-6.36193695	ns
SEMA6C	-0.00634314	0.031047802	-1.15391813	0.248552077	0.411435412	-6.35547976	ns
CPTP	-0.00947967	0.063736817	-1.15052047	0.24994787	0.41351149	-6.35990513	ns
LZTFL1	-0.01681065	0.015091715	-1.1500176	0.250154782	0.413619458	-6.36227709	ns
NCF2	-0.02170764	0.06640549	-1.14728509	0.251281731	0.415112991	-6.37323064	ns
SCG3	-0.00850948	0.010986714	-1.14713819	0.251342386	0.415112991	-6.3734177	ns
GBP1	-0.01541005	-0.01883414	-1.14642475	0.25163718	0.415364933	-6.37844962	ns
TCOF1	-0.00712751	-0.00607701	-1.14568411	0.251943581	0.415635738	-6.38010957	ns
CAT	0.013078184	0.057282782	1.144041708	0.252624311	0.416523426	-6.373411	ns
PAFAH1B3	-0.02803515	0.44885687	-1.14248934	0.253268297	0.417349563	-6.384663	ns
SPINK5	-0.00827374	0.02931036	-1.14182725	0.25354362	0.417476549	-6.38077609	ns
REG3G	-0.00919421	0.001160403	-1.14134194	0.253745343	0.417476549	-6.38405147	ns
TPSG1	-0.00603233	0.01295535	-1.14127357	0.253774272	0.417476549	-6.36987575	ns
ACY3	-0.01282655	0.017321984	-1.14053705	0.254080798	0.417732916	-6.37125101	ns
FKBP1B	-0.02766036	0.054004095	-1.14021065	0.25421623	0.417732916	-6.38450361	ns
DFFA	-0.01693552	0.024625016	-1.13955082	0.254491057	0.417737162	-6.3879598	ns
DTYMK	-0.01620557	-0.01380721	-1.13951765	0.254504935	0.417737162	-6.38710131	ns
MATN2	-0.00734599	-0.00991168	-1.13894364	0.254744429	0.417895356	-6.38271179	ns
PSCA	0.060022864	-1.29913264	1.137404232	0.255386963	0.418714167	-6.38833475	ns
SNX9	-0.01717116	0.030647864	-1.13662254	0.255713907	0.418827044	-6.38399668	ns
EP300	-0.00691564	0.001158383	-1.13655451	0.255742678	0.418827044	-6.37520782	ns
DEFB103A_DE	-0.00964412	0.035581048	-1.13485802	0.256453062	0.419755012	-6.37277371	ns
C2orf69	-0.01330328	0.071056661	-1.13332263	0.257096768	0.420572864	-6.38085918	ns
DEFB116	-0.0174663	0.196574092	-1.1316993	0.257778873	0.42145258	-6.38300819	ns
CLEC11A	-0.01158222	-0.03148448	-1.13038499	0.258331719	0.4221201	-6.39239084	ns
GC	0.005930866	0.026826384	1.129167873	0.258844505	0.422721452	-6.39844548	ns
GOLM2	-0.00557211	-0.02292632	-1.1230886	0.261417254	0.426684395	-6.40647847	ns
TEX33	-0.00911278	0.043406608	-1.12168086	0.2620158	0.427422423	-6.39925813	ns
CASP8	-0.01533154	0.032287333	-1.12007628	0.262698635	0.428297049	-6.40910774	ns
ARNTL	0.012074826	0.106952634	1.118639386	0.263311889	0.429057319	-6.39579086	ns
CPLX2	-0.00700277	0.002855245	-1.11780481	0.26366824	0.429398361	-6.39502625	ns
CA1	0.015325341	0.002897596	1.117363496	0.263856963	0.429466183	-6.3919417	ns
TSPAN15	0.011429065	0.077819981	1.115524336	0.264643628	0.430506627	-6.39924313	ns
PRSS22	-0.00760112	0.007900904	-1.11417883	0.265220286	0.431204475	-6.40018946	ns
ACP6	-0.01674853	-0.13529999	-1.11132525	0.266445612	0.432955586	-6.41939639	ns
HTR1A	-0.01697022	0.193318319	-1.11037454	0.266855373	0.43338025	-6.40672582	ns
RASA1	-0.00793475	-0.0007947	-1.10990672	0.267056937	0.433466512	-6.40713737	ns
HGS	-0.01897836	0.049616302	-1.10617775	0.268667281	0.435621152	-6.41035539	ns
RANBP2	-0.00751716	0.007332881	-1.10614186	0.268682772	0.435621152	-6.4113798	ns
LYPD1	-0.01558188	0.163751606	-1.10489358	0.269223347	0.436024302	-6.41264521	ns
ICAM2	-0.00771127	-0.03876842	-1.10487689	0.269230074	0.436024302	-6.42613729	ns
GLYR1	-0.01371551	0.120905003	-1.10392192	0.269644203	0.436300258	-6.4257375	ns
CRH	0.021470907	0.086425125	1.103796092	0.269699303	0.436300258	-6.411988	ns
LILRA4	-0.01156146	0.083317148	-1.10304923	0.270023443	0.436582755	-6.41297227	ns

PALLD	-0.00912262	0.043042615	-1.10242454	0.270294794	0.436779634	-6.41311372	ns
CEP164	0.014995286	0.098847399	1.101489688	0.27070114	0.437047183	-6.41638308	ns
C1QL2	-0.00948761	0.037425956	-1.10135524	0.27075971	0.437047183	-6.41428266	ns
PAIP2B	-0.01203626	0.038378239	-1.09954778	0.271546868	0.43807561	-6.41367362	ns
CCL27	-0.01441907	-0.07629135	-1.09705672	0.272633853	0.439586334	-6.42784202	ns
EHD3	-0.02629633	-0.09078662	-1.09540467	0.273356886	0.440296551	-6.42214475	ns
SNX2	-0.01855709	0.062905844	-1.09511729	0.273482324	0.440296551	-6.43534397	ns
EDNRB	-0.01027306	0.062912895	-1.09501708	0.273526693	0.440296551	-6.42118809	ns
CD8A	-0.01187181	-0.01229686	-1.09422434	0.273873595	0.440467738	-6.43823406	ns
NGF	0.002875738	-0.00377047	1.09408552	0.27393473	0.440467738	-6.43027785	ns
SLC39A14	-0.00764533	0.00447781	-1.09171804	0.27497451	0.441896296	-6.42700208	ns
IGF1R	-0.00502922	-0.00848575	-1.09116051	0.275219445	0.44197134	-6.43379502	ns
NENF	-0.02543106	0.381918551	-1.09092266	0.275323927	0.44197134	-6.43989357	ns
IST1	-0.02216506	-0.02995086	-1.09008014	0.275694956	0.442323776	-6.43655013	ns
TREML1	-0.01174251	0.037420251	-1.08792828	0.276643707	0.443602211	-6.44294081	ns
SNX15	-0.01231173	0.000995616	-1.08678857	0.277147557	0.444166228	-6.43243589	ns
SCIN	0.016472725	0.081131353	1.085548477	0.27769616	0.444622882	-6.43376936	ns
ABCA2	-0.00814403	0.000989298	-1.08545626	0.277737033	0.444622882	-6.43207138	ns
C8B	0.006775739	-0.01490046	1.084938451	0.277965797	0.444745275	-6.44703407	ns
F10	0.005780951	-0.01711836	1.084305803	0.278246121	0.444949985	-6.44684501	ns
APCS	-0.00845703	-0.08561453	-1.08392235	0.27841626	0.444978369	-6.44329053	ns
CDH2	-0.00839873	-0.04225964	-1.08280971	0.278909951	0.445445509	-6.44324972	ns
AKR1C4	-0.01263459	0.040982254	-1.08257678	0.279013643	0.445445509	-6.43689434	ns
CEACAM19	-0.00854111	-0.03398322	-1.08008976	0.280119055	0.446929871	-6.45139351	ns
GFAP	-0.01022804	0.000249587	-1.07979793	0.280249519	0.446929871	-6.43821163	ns
EVI2B	-0.00908115	0.035949431	-1.07776926	0.281154047	0.448029268	-6.43862843	ns
SCRN1	-0.01325832	0.043232895	-1.07756298	0.28124577	0.448029268	-6.45115668	ns
PTRHD1	-0.01333848	-0.00351064	-1.07562423	0.282112481	0.44916491	-6.44441187	ns
GBP2	-0.01397642	-0.01985941	-1.07325958	0.283171809	0.450605821	-6.4468382	ns
DAPK2	-0.01185096	0.045879284	-1.07279312	0.283381167	0.450693359	-6.44508633	ns
NXPE4	0.007498188	0.033016579	1.071159436	0.284114944	0.45136271	-6.44736603	ns
OGA	-0.01561397	0.01674354	-1.07115549	0.28411624	0.45136271	-6.46092959	ns
NCAM2	0.006577362	0.025691835	1.070823171	0.284265762	0.45136271	-6.45872275	ns
SWAP70	-0.00792615	0.015111235	-1.0704362	0.284439721	0.45139347	-6.46169389	ns
PTPRC	-0.00502483	-0.00194468	-1.06972189	0.284761188	0.451658158	-6.46304705	ns
KLRF1	-0.00907973	0.007081496	-1.06849348	0.285315126	0.452291079	-6.44965334	ns
CCL5	-0.02429256	-0.11593381	-1.06625925	0.286323235	0.45364289	-6.46234815	ns
IGFL4	-0.01637333	0.178911512	-1.06447286	0.287131637	0.454657235	-6.45390737	ns
VCAN	0.006970629	0.00580492	1.064155432	0.287274863	0.454657235	-6.47043586	ns
RCC1	-0.01098227	0.097805566	-1.06115541	0.288635866	0.456319185	-6.4716783	ns
FOXO3	-0.02262126	0.111166139	-1.06115278	0.288637512	0.456319185	-6.45876273	ns
F11	0.006636448	0.004171939	1.058971729	0.289629439	0.457262834	-6.46658265	ns
GALNT2	-0.00587456	0.01457175	-1.0588662	0.2896772	0.457262834	-6.47664563	ns
IMPA1	-0.01419488	0.039305942	-1.05880753	0.289704193	0.457262834	-6.46795742	ns
CD177	-0.02980952	-0.24156238	-1.05735982	0.290364048	0.457600973	-6.47212351	ns
CSF2RB	-0.00934801	-0.04826485	-1.05705037	0.290505582	0.457600973	-6.46171864	ns
PMS1	-0.01110747	0.028484365	-1.05700083	0.2905281	0.457600973	-6.46378567	ns
GAL	0.01527735	0.068500376	1.056698357	0.290665834	0.457600973	-6.47443852	ns
BECN1	-0.0134298	0.047563299	-1.05661998	0.290701988	0.457600973	-6.46354861	ns
GRIN2B	-0.01232716	0.105742331	-1.05232853	0.292665712	0.460427503	-6.46900012	ns
TAGLN3	0.013136408	-0.00809489	1.052007628	0.29281297	0.460427503	-6.4658444	ns
CA7	-0.00728966	0.021657627	-1.05011769	0.293680629	0.461543291	-6.47636328	ns
SLC51B	0.016402942	0.179493295	1.04945495	0.29398565	0.461774125	-6.4701978	ns
WAS	-0.01547343	0.044807297	-1.04827386	0.294529219	0.462379203	-6.47313373	ns
PROS1	0.005323177	-0.01725959	1.046512451	0.295340824	0.463287026	-6.48767163	ns
MFAP4	-0.00669481	0.016450145	-1.04633127	0.295424809	0.463287026	-6.47524662	ns
CNTN3	0.007664682	-0.041332	1.045175706	0.295958207	0.463854521	-6.48564828	ns
MENT	0.004698165	0.007306714	1.044859919	0.296104393	0.463854521	-6.47677296	ns
GFRA3	-0.00635191	0.018740388	-1.03862963	0.298993514	0.468109839	-6.48728361	ns
CNTNAP2	-0.00931643	-0.00203018	-1.03824712	0.299171379	0.468109839	-6.4924278	ns
APOE	0.015256193	0.064077749	1.037966744	0.299301736	0.468109839	-6.49651004	ns
PLXDC1	-0.00778304	0.029775412	-1.03714381	0.299685514	0.468459155	-6.48375324	ns
TYRP1	0.013074702	-0.04533902	1.036110352	0.300167327	0.468720455	-6.48346093	ns
MB	-0.01166736	0.020739999	-1.03609556	0.300173716	0.468720455	-6.499203	ns
CFHR5	-0.00869585	-0.05389789	-1.0354985	0.300452595	0.468905173	-6.49152281	ns
PROK1	-0.01424927	-0.09842003	-1.03412965	0.301091853	0.469651822	-6.49666133	ns

RWDD1	-0.01463206	-0.00280278	-1.0337308	0.301278152	0.469691512	-6.50210555	ns
ADGRG2	0.006185472	0.033110771	1.0312607	0.302434875	0.471243242	-6.49731594	ns
PCDH7	-0.00723936	0.044885238	-1.0302072	0.302928862	0.471761215	-6.50362282	ns
NCAM1	0.006939812	0.017233854	1.029763053	0.303137471	0.471834443	-6.50030063	ns
IL36A	-0.01187852	0.067156102	-1.02839577	0.303780242	0.472583008	-6.49136143	ns
CD109	-0.00750562	-0.00691789	-1.02634582	0.304744758	0.473796386	-6.50500103	ns
FCGR2A	-0.01395129	-0.16488572	-1.02604917	0.304884729	0.473796386	-6.49937768	ns
ACADM	-0.01468011	0.12953047	-1.0215856	0.306993264	0.476819325	-6.50568831	ns
CEACAM20	-0.00927382	0.044162581	-1.02062253	0.307449482	0.477274049	-6.50666405	ns
GZMA	0.007730457	-0.01291418	1.019356987	0.308049396	0.477951241	-6.5167108	ns
FXYD5	-0.01446594	0.054979395	-1.01711887	0.309113175	0.479224382	-6.50505443	ns
SART1	-0.00790212	0.02881335	-1.0169403	0.309198197	0.479224382	-6.50352682	ns
ROBO4	-0.00367729	-0.00486291	-1.01641251	0.309449299	0.479314028	-6.50489604	ns
ITGAX	-0.01118547	0.048829583	-1.01590912	0.309688766	0.479314028	-6.51142622	ns
CTSH	-0.0167118	-0.02036375	-1.01578426	0.309748483	0.479314028	-6.50526816	ns
XRCC4	-0.00815742	0.008474459	-1.01406238	0.310569269	0.480329589	-6.50812186	ns
OXCT1	-0.01416688	0.010908425	-1.00692496	0.313986994	0.485358402	-6.51441873	ns
LUZP2	-0.0089324	0.030133536	-1.00634402	0.31426587	0.485532456	-6.52790474	ns
VIPR1	0.004909488	0.008147347	1.005063645	0.314882333	0.485830393	-6.51627646	ns
GUCA2A	-0.00698759	0.007761604	-1.0046742	0.315069675	0.485830393	-6.52327464	ns
GATA3	0.005433332	-0.00430468	1.004334512	0.31523358	0.485830393	-6.51447251	ns
SCGB1A1	0.011898168	-0.03176435	1.003958768	0.315414314	0.485830393	-6.52649035	ns
ITGB1	0.004742449	0.010320345	1.00391884	0.315433653	0.485830393	-6.5236787	ns
NAPRT	0.006887043	0.036937627	1.003869905	0.315456995	0.485830393	-6.5313051	ns
TUBB3	-0.00580715	-0.01333607	-1.00185705	0.316428715	0.486843245	-6.52043155	ns
EIF4E	-0.01862215	0.016366396	-1.001817	0.316448109	0.486843245	-6.51812869	ns
BRDT	-0.00748619	0.00774433	-1.00129085	0.31670233	0.48697778	-6.51919438	ns
DHPS	-0.00840921	0.015589513	-0.99911437	0.317754936	0.488339165	-6.53509852	ns
BGN	-0.02592648	0.192235778	-0.99717101	0.318697576	0.489530206	-6.52342031	ns
HDDC2	0.008949475	0.034345231	0.996207305	0.319165019	0.489990461	-6.5378074	ns
IL17C	-0.015057	0.029765727	-0.99442628	0.320031305	0.491062224	-6.52769864	ns
HDGF	0.020780927	0.043572348	0.992536685	0.3209513	0.492215229	-6.54330097	ns
PAGR1	0.008673911	0.030777056	0.990304163	0.322041363	0.493627706	-6.53088585	ns
C9	-0.01040649	-0.06124499	-0.98895075	0.322702699	0.494381889	-6.54493387	ns
ZP3	0.053278308	-1.06320738	0.987924199	0.323205626	0.494892725	-6.53322138	ns
OLR1	-0.01278245	0.012470295	-0.98662849	0.323840558	0.495605046	-6.53651541	ns
FGF12	-0.00940133	0.065087724	-0.98569955	0.32429643	0.495645696	-6.53401911	ns
ELOA	-0.01215159	0.022069614	-0.9856223	0.324334081	0.495645696	-6.54253394	ns
C1GALT1C1	0.006543648	0.008485413	0.985536765	0.324376344	0.495645696	-6.53472132	ns
SNCA	-0.02329735	-0.03125633	-0.9844994	0.324885754	0.496021126	-6.53753826	ns
PTPRK	-0.00431826	0.011105959	-0.98434404	0.324961786	0.496021126	-6.54946519	ns
METAP1	-0.00650568	0.010479665	-0.98381451	0.325222519	0.496159746	-6.53810018	ns
FUT1	-0.01284196	0.076210297	-0.98267642	0.32578232	0.496754243	-6.5437818	ns
PRSS53	-0.00753327	0.003924755	-0.98206183	0.326084717	0.496955832	-6.55279403	ns
GRIK2	-0.0081572	0.066927855	-0.98071255	0.326749797	0.497482787	-6.5550918	ns
CD276	-0.00735843	-0.04616761	-0.98066951	0.326771228	0.497482787	-6.54776374	ns
RNF5	-0.01462524	-0.09088716	-0.98032334	0.326942266	0.497483803	-6.5406427	ns
SERPINI2	0.011167534	0.090164688	0.979191517	0.327500678	0.498073948	-6.55448497	ns
PTP4A3	-0.01054837	0.02628235	-0.97795459	0.328112516	0.498744689	-6.54156332	ns
CREBZF	0.00525078	0.007433767	0.977577851	0.328298898	0.498768356	-6.54077744	ns
TEX101	0.009845542	0.099581517	0.976539037	0.328813041	0.499289693	-6.54495344	ns
COPE	0.009853225	0.040668882	0.975158738	0.32949718	0.500068486	-6.5465118	ns
COX5B	-0.0149001	0.011783154	-0.97455761	0.329795158	0.500260708	-6.5532942	ns
ERI1	0.00680104	0.003819022	0.973787256	0.33017761	0.5005808	-6.55300523	ns
CTNNA1	-0.00706839	0.053493578	-0.97246382	0.330835458	0.501007311	-6.54921948	ns
AMDHD2	-0.00510517	-0.03569156	-0.97228346	0.330924739	0.501007311	-6.56176931	ns
PRC1	-0.00915705	0.072129964	-0.97218515	0.330973666	0.501007311	-6.56143778	ns
EIF5	-0.00868052	0.045853798	-0.97127018	0.331429381	0.501437198	-6.54856977	ns
CENPJ	-0.01413213	0.10837507	-0.97067366	0.331726188	0.501626343	-6.55600883	ns
IL13RA1	-0.00479066	-0.00252625	-0.970208	0.331958194	0.501717354	-6.55662425	ns
RBP2	-0.01635959	0.011937797	-0.96809819	0.333010638	0.503047627	-6.55951975	ns
IL31RA	0.007962335	0.021292701	0.967654982	0.333232345	0.503122258	-6.54969867	ns
TNFSF13	-0.00549148	-0.01283804	-0.96588957	0.334114595	0.5041936	-6.56556569	ns
GMPR2	-0.01328384	0.02200311	-0.96365411	0.335234756	0.505519203	-6.55768372	ns
IFNL1	-0.00932535	-0.01199736	-0.96344587	0.33533928	0.505519203	-6.55777594	ns
CEACAM1	0.004346648	-0.00458456	0.962730365	0.335697935	0.505798746	-6.56916309	ns

TFPI	-0.00678606	-0.02593067	-0.96143604	0.336348234	0.506517196	-6.56398081	ns
CLTA	-0.00964304	0.04076626	-0.96081609	0.336660066	0.50672546	-6.56031891	ns
FGR	-0.01253972	0.049065703	-0.95972803	0.33720751	0.507287959	-6.56132289	ns
C1QTNF1	-0.00890656	-0.00940625	-0.95900389	0.337571886	0.507566091	-6.57011847	ns
PDGFRB	-0.00739305	-0.0410986	-0.95864899	0.337750548	0.507566091	-6.57585539	ns
TRIM21	-0.01617466	0.002997749	-0.95832527	0.337913863	0.507566091	-6.57159692	ns
ARHGAP5	0.014848113	0.160408679	0.956689859	0.338739213	0.508544216	-6.56081918	ns
SIL1	-0.00633233	0.036300461	-0.95431247	0.339940794	0.510085878	-6.56560552	ns
CDKL5	0.01029812	0.076932762	0.951923435	0.341151072	0.511555527	-6.56882812	ns
C2	-0.00502749	-0.03676342	-0.95168729	0.341270605	0.511555527	-6.57645798	ns
SIRT5	-0.01157564	0.092522442	-0.94871119	0.342782737	0.513219685	-6.57175283	ns
CD2	-0.007304	0.022154011	-0.94832403	0.342979768	0.513219685	-6.57041989	ns
MELTF	-0.00558784	0.000390219	-0.94816975	0.343057839	0.513219685	-6.58530849	ns
WARS	-0.00713903	-0.02033913	-0.94801478	0.343136834	0.513219685	-6.58173168	ns
EGFLAM	-0.0058715	0.02256098	-0.9477674	0.343262692	0.513219685	-6.58452932	ns
INSL4	0.009547888	0.050455278	0.947428357	0.343435365	0.513219685	-6.5850174	ns
OPTC	-0.0094771	0.035810386	-0.94667865	0.343817507	0.513527939	-6.57986754	ns
TNNI3	0.017291988	-0.10155824	0.944810912	0.344770571	0.514688174	-6.57544913	ns
LRP2	-0.00697487	0.008463945	-0.94433533	0.34501341	0.51478751	-6.57564008	ns
ARMCX2	0.012309333	0.119234436	0.943485473	0.345447759	0.515172348	-6.57676205	ns
MEGF11	-0.0063947	0.039142756	-0.94188119	0.346268606	0.516132889	-6.57646106	ns
ODAM	0.011101941	0.080863638	0.940923832	0.346759001	0.516249042	-6.5783642	ns
PSMG3	-0.0108233	0.0163393	-0.94091601	0.346762716	0.516249042	-6.58800163	ns
CNTNAP4	-0.02048922	0.363790031	-0.94069362	0.346876925	0.516249042	-6.57904549	ns
GNPDA2	0.007035964	0.030758233	0.939712649	0.347379962	0.516579337	-6.58028954	ns
CEP152	0.008055975	0.020859488	0.939571168	0.347452677	0.516579337	-6.57692162	ns
FGF9	0.007699078	-0.02044946	0.938151848	0.348181499	0.517235791	-6.57680655	ns
DDX4	0.010575698	0.069556098	0.938021398	0.348248481	0.517235791	-6.57951707	ns
SERPINB9	-0.00695015	-0.06070617	-0.93729399	0.348622179	0.517527586	-6.58829227	ns
MYBPC1	0.013192605	0.055926679	0.936763848	0.348894652	0.517668894	-6.59597597	ns
PPCDC	-0.00993158	0.011964808	-0.93606237	0.349255654	0.517676577	-6.59756412	ns
SCGB2A2	0.005959441	0.01818387	0.935856354	0.349362186	0.517676577	-6.58207303	ns
AHNAK	-0.00620615	-0.01915085	-0.93572129	0.349431689	0.517676577	-6.58303633	ns
CRADD	-0.01578257	-0.01467828	-0.93482673	0.349892418	0.517682142	-6.58928829	ns
IL17A	-0.00875347	0.032403889	-0.93459782	0.350010544	0.517682142	-6.58495074	ns
IL17D	-0.00692557	0.000777489	-0.93457271	0.350023484	0.517682142	-6.58497402	ns
ROBO2	-0.00457124	-0.0017756	-0.93433706	0.350144599	0.517682142	-6.59916531	ns
IGFBP7	-0.00567852	-0.01732856	-0.93383642	0.350402814	0.517801729	-6.59915471	ns
GPR158	-0.00665877	0.046783736	-0.93084036	0.351950624	0.519825909	-6.60239456	ns
DNAJC6	-0.01608867	0.033372294	-0.92816284	0.353337964	0.521611149	-6.59002498	ns
COL2A1	0.015289946	0.076571737	0.927718318	0.353568554	0.521687811	-6.59043423	ns
RAB2B	-0.01164611	0.019342448	-0.92664003	0.35412794	0.522249286	-6.60434753	ns
VPS28	-0.01276407	0.065348707	-0.92560294	0.354666995	0.522780225	-6.59841007	ns
GGT1	0.009901925	0.002610699	0.925228871	0.354861432	0.522802916	-6.60172785	ns
MMP9	-0.01256101	-0.07186344	-0.92474355	0.355113878	0.522911006	-6.60226733	ns
PLXNB2	-0.0046691	-0.01441118	-0.92406435	0.355467276	0.522926054	-6.60529279	ns
TP53BP1	0.016967801	0.251193469	0.924036261	0.355482266	0.522926054	-6.59243497	ns
SHC1	-0.02165956	0.233263641	-0.92044716	0.357353912	0.525414614	-6.59456657	ns
S100A11	-0.00927189	0.026371865	-0.91929252	0.357957186	0.525928758	-6.60218553	ns
WFIKKN1	-0.00817277	0.013343391	-0.91907732	0.358069704	0.525928758	-6.60328667	ns
SLAMF7	-0.01184063	-0.04434031	-0.91874399	0.358243938	0.525928758	-6.60845891	ns
IL13	0.010187825	0.067688192	0.916780765	0.359272169	0.527173233	-6.60131435	ns
PECR	-0.00536488	0.021820899	-0.91368037	0.36089943	0.528940366	-6.60325735	ns
RILP	-0.01230548	-0.00541491	-0.91353855	0.360973598	0.528940366	-6.61750945	ns
KLRD1	-0.00928763	-0.02532786	-0.91309375	0.361207409	0.528940366	-6.61860097	ns
CA12	-0.00617868	0.010701771	-0.91269134	0.361419281	0.528940366	-6.61122009	ns
CACNB1	-0.01099438	0.082709257	-0.91249709	0.361521689	0.528940366	-6.60529333	ns
TDO2	-0.00714299	0.031305279	-0.91241785	0.361563346	0.528940366	-6.60503884	ns
RFC4	-0.0040093	0.007016726	-0.91054244	0.362550713	0.529928934	-6.61902136	ns
YY1	-0.00514421	0.011204456	-0.91037346	0.362640184	0.529928934	-6.60486778	ns
CCN5	-0.00793795	-0.03328942	-0.91010096	0.362783541	0.529928934	-6.61668448	ns
ARTN	-0.00924178	0.04621528	-0.90942411	0.363140807	0.530113722	-6.60797902	ns
TCN1	-0.00626523	-0.00375252	-0.90917326	0.363273136	0.530113722	-6.60974331	ns
L1CAM	-0.00568178	-0.02232053	-0.90805223	0.363864804	0.530580254	-6.61991562	ns
AP2B1	-0.00913336	0.078026705	-0.90787972	0.36395625	0.530580254	-6.6091368	ns
CARHSP1	0.010408098	0.032933344	0.907178552	0.364326891	0.530855549	-6.60999033	ns

PAFAH2	-0.00930778	0.031797092	-0.90506817	0.365443557	0.532217051	-6.6170603	ns
CTRC	0.012315856	0.04297903	0.90349694	0.366276413	0.533008585	-6.62028074	ns
NAP1L4	-0.00763692	-0.02855715	-0.90327256	0.366395358	0.533008585	-6.6240449	ns
SDC4	0.009105984	-0.0308522	0.902900576	0.366592872	0.533008585	-6.62048854	ns
NPHS2	-0.01939086	0.279344091	-0.90266683	0.366717208	0.533008585	-6.61179893	ns
PSG1	0.022621028	-0.18310122	0.901402694	0.367388466	0.533718568	-6.62103296	ns
HSP90B1	-0.01335242	0.15075284	-0.89937517	0.368467316	0.535019673	-6.61437412	ns
BMP10	-0.0053024	0.01137317	-0.89879878	0.368774139	0.535199048	-6.61760475	ns
HLA-DRA	-0.0068366	-0.00638852	-0.89688043	0.369796796	0.536416614	-6.62815396	ns
NEDD4L	-0.00901138	-0.03797548	-0.89471874	0.370951778	0.537824823	-6.61888552	ns
GPHA2	0.008523696	0.102839806	0.893410864	0.371651091	0.538410997	-6.63418593	ns
DAND5	0.010438103	-0.02889761	0.893273818	0.371724853	0.538410997	-6.6201671	ns
LPCAT2	-0.0079835	0.042602369	-0.89221104	0.37229386	0.538967809	-6.62955936	ns
CENPF	0.010408633	0.062890825	0.889062619	0.373983435	0.54067024	-6.63130685	ns
HAGH	0.014524632	0.031452897	0.889052782	0.373988529	0.54067024	-6.63940978	ns
BAIAP2	-0.01238743	-0.01061989	-0.8889851	0.374025303	0.54067024	-6.62531702	ns
KDM3A	-0.01666647	0.255803182	-0.88785821	0.374631229	0.541153496	-6.62495205	ns
NPTX1	0.00778355	0.025436673	0.887660088	0.374737512	0.541153496	-6.63670084	ns
CDH23	0.004797322	0.015483431	0.887328952	0.37491559	0.541153496	-6.64066329	ns
LHPP	-0.0117966	0.026361004	-0.88651446	0.375354235	0.541518956	-6.63734914	ns
CD3E	0.0063145	0.022095897	0.883939475	0.376742805	0.543253823	-6.63581327	ns
UBAC1	0.006871859	0.007699667	0.883353652	0.377059105	0.543441553	-6.63736188	ns
NACC1	-0.01027652	0.03640718	-0.88238722	0.377581173	0.543925518	-6.64406827	ns
UBE2B	-0.00524509	0.012489597	-0.88048476	0.378610455	0.545139313	-6.64573234	ns
PTPRM	-0.00466659	-0.00924396	-0.87929788	0.379253581	0.545731573	-6.64270876	ns
KLK3	0.015464794	0.561798654	0.879035665	0.37939558	0.545731573	-6.64742438	ns
APOB	-0.0040813	-0.03276531	-0.87817106	0.379864522	0.546137078	-6.6475608	ns
VMO1	-0.01302275	-0.09710761	-0.87773198	0.380102969	0.546210959	-6.64227365	ns
SPINT3	0.026422427	0.982776912	0.876875828	0.380567709	0.546609793	-6.64870972	ns
DENR	0.010334191	0.057299741	0.874734783	0.381732202	0.548012797	-6.63642656	ns
CEACAM8	-0.00930756	-0.03628282	-0.87319552	0.382570432	0.548946271	-6.64003698	ns
VWC2L	-0.00845057	0.087433601	-0.8716798	0.383396567	0.549861481	-6.6553069	ns
NRP1	0.005194557	-0.00772109	0.868898637	0.384916128	0.551769805	-6.65329628	ns
BRD3	-0.00856638	0.040608058	-0.86648475	0.386238214	0.553393319	-6.64409652	ns
BAP18	-0.01222981	0.013947613	-0.86576049	0.386634919	0.553690026	-6.65942456	ns
KIT	0.005570573	0.015016934	0.864861182	0.38712852	0.554125137	-6.65471038	ns
KCNIP4	-0.00966007	0.049283922	-0.86317682	0.388053995	0.555006273	-6.64863417	ns
IL1A	0.010700731	0.074834604	0.86304896	0.38812425	0.555006273	-6.648756	ns
ENOPH1	0.009509106	0.092678459	0.862352094	0.388507178	0.555281919	-6.66141253	ns
FCRL2	-0.0091923	0.008403667	-0.86167954	0.388877214	0.555538877	-6.66328396	ns
DOC2B	-0.00621651	0.019526688	-0.86107101	0.389212592	0.555677567	-6.65021306	ns
CRLF1	-0.00493022	-0.00557887	-0.86081215	0.389354898	0.555677567	-6.66080032	ns
FAM13A	-0.01205182	0.053318985	-0.86026735	0.389655024	0.555702754	-6.66302493	ns
ANK2	0.012651375	0.170783597	0.860089973	0.389753164	0.555702754	-6.64957361	ns
SLC13A1	-0.01105719	0.119842829	-0.85971222	0.389961035	0.55572778	-6.66366815	ns
ID4	0.007861641	0.076025211	0.859022427	0.39034163	0.555998809	-6.65735947	ns
PARP1	-0.01036383	0.039102978	-0.85468027	0.392741521	0.559144437	-6.66482736	ns
MORF4L2	-0.00676717	0.058965624	-0.85357159	0.393356017	0.559746379	-6.65341734	ns
RECK	0.00356045	0.005920219	0.849045498	0.395869455	0.563048616	-6.65977655	ns
DGKZ	-0.00650429	0.008008366	-0.84841668	0.396219398	0.563271977	-6.66117915	ns
GP1BB	-0.01181438	0.0065882	-0.84704511	0.396983464	0.564083559	-6.66062069	ns
LILRB2	-0.0064418	-0.05750719	-0.84654537	0.397261715	0.564204381	-6.6730994	ns
FGFBP1	0.005841733	0.004076195	0.842983569	0.399250695	0.566753539	-6.67644714	ns
MRPL24	-0.0066253	0.013004816	-0.83738745	0.402388128	0.570929705	-6.66869055	ns
CHI3L1	-0.01342162	0.00532414	-0.83591265	0.403217446	0.57182853	-6.66555274	ns
NGRN	-0.00380461	0.010941475	-0.83394549	0.404324552	0.573120238	-6.68625614	ns
ATP6V1D	-0.00499374	0.006619696	-0.83318979	0.404750705	0.573231524	-6.68008271	ns
DKK3	-0.0053737	0.007080971	-0.83310971	0.404795686	0.573231524	-6.68746478	ns
ENO3	-0.01026046	0.053892122	-0.83253681	0.405119195	0.57341156	-6.67354791	ns
NPTN	-0.01481913	0.178361566	-0.82838733	0.407464325	0.576410089	-6.67694278	ns
UGDH	-0.01701083	0.276952341	-0.82809036	0.407632478	0.576410089	-6.67637171	ns
SKAP1	-0.01362695	-0.01034146	-0.82764629	0.407883807	0.576486311	-6.68300556	ns
ENPP6	0.006481504	0.036315162	0.827088045	0.408200248	0.576654439	-6.67803387	ns
UNG	-0.00589145	0.002390642	-0.82664397	0.408451651	0.57673057	-6.69133725	ns
CCT5	-0.00837743	0.05462942	-0.82387233	0.410025165	0.578672538	-6.68064347	ns
MTPN	0.005977985	0.025931889	0.820995814	0.411661643	0.580216282	-6.68390851	ns

SFRP4	-0.00701157	0.021823741	-0.82095389	0.411685454	0.580216282	-6.68401699	ns
HDAC9	-0.01111486	0.087882006	-0.82090192	0.411715115	0.580216282	-6.68405643	ns
CST5	-0.00993725	0.032074523	-0.8191199	0.412730954	0.581200291	-6.68530655	ns
FRZB	0.004660259	-0.03604365	0.818747834	0.412943127	0.581200291	-6.68933681	ns
GHRHR	0.009906714	0.085430385	0.818630019	0.413010481	0.581200291	-6.6858001	ns
IFNL2	0.010417633	0.110522125	0.817324178	0.413756107	0.581969091	-6.68696405	ns
IFNAR1	-0.00427929	0.02396281	-0.81427332	0.41550093	0.584141895	-6.70123966	ns
OSM	-0.01305238	-0.07276487	-0.81254334	0.416492588	0.585254262	-6.69768236	ns
SOWAHA	0.008181614	0.065157369	0.808971248	0.418544418	0.587815195	-6.69337632	ns
ENTPD5	-0.00374643	-0.01061553	-0.80867015	0.418717673	0.587815195	-6.69387317	ns
NOTCH3	0.005988646	0.005703887	0.808110124	0.419039583	0.587984422	-6.70782324	ns
YTHDF3	-0.01492601	-0.01614724	-0.80758336	0.419343186	0.588127811	-6.69472417	ns
CGN	-0.0065819	0.032727112	-0.80687718	0.419749942	0.588415665	-6.6950549	ns
CD244	-0.00477565	-0.00300112	-0.80648285	0.419976838	0.588451232	-6.70956495	ns
HMGCL	-0.00804935	0.036305756	-0.80573602	0.420407783	0.588772531	-6.69532908	ns
NLGN1	0.011171955	0.147300529	0.805065149	0.420794749	0.589031959	-6.6965039	ns
ATP1B4	-0.0069753	0.04827829	-0.80414999	0.421323081	0.589488929	-6.69405771	ns
CCL2	-0.0094296	0.028783068	-0.80065028	0.423346614	0.592036452	-6.7050796	ns
MYL6B	-0.01539539	0.267128984	-0.79876236	0.424440769	0.593282453	-6.70151858	ns
DCUN1D1	-0.0125155	0.195712265	-0.79819607	0.424769291	0.593457574	-6.70229394	ns
ANGPT2	0.00665324	-0.00772069	0.796631033	0.425677534	0.594442085	-6.71807955	ns
CD248	-0.00645478	0.007250087	-0.79595401	0.426070968	0.594707088	-6.71647297	ns
SMPDL3B	-0.0124255	0.164424298	-0.79404325	0.427182449	0.595943789	-6.71755301	ns
CCND2	-0.00608821	-0.02658451	-0.79356849	0.427459015	0.595943789	-6.71102429	ns
PDE1C	0.008902996	0.090444885	0.792965774	0.427810286	0.595943789	-6.7029185	ns
DEFB104A_DE	0.008985394	0.097146164	0.792608626	0.428018352	0.595943789	-6.7067034	ns
TBC1D17	0.007739818	0.041676135	0.792569578	0.428041117	0.595943789	-6.7066299	ns
PPIF	-0.01003092	0.089731501	-0.79181956	0.428478028	0.595943789	-6.71973019	ns
SSBP1	-0.00581914	0.023045651	-0.79178765	0.428496994	0.595943789	-6.704999	ns
ISM2	0.006913613	0.006836245	0.791628054	0.428589711	0.595943789	-6.71988065	ns
CLINT1	0.007952828	0.025287201	0.788826627	0.430225792	0.59793399	-6.70616632	ns
NAMPT	-0.00926134	0.040595742	-0.78735951	0.431083847	0.598785878	-6.7098279	ns
CLUL1	0.007900324	0.035076086	0.787077208	0.43124887	0.598785878	-6.716985	ns
IFNG	-0.019601	-0.05072172	-0.78500556	0.432462617	0.600185762	-6.71256191	ns
PRKRA	-0.01173498	0.022198325	-0.78279852	0.433757758	0.601697223	-6.71338054	ns
FCN2	0.007275979	-0.06413633	0.782255091	0.434076847	0.601790087	-6.71842424	ns
ATP6V1G2	-0.01551177	-0.00683579	-0.78191876	0.434274632	0.601790087	-6.71324785	ns
EIF5A	-0.00518607	0.008168778	-0.78163225	0.43444298	0.601790087	-6.7151836	ns
SPESP1	-0.00473418	0.025112008	-0.78032131	0.4352137	0.602571837	-6.72926271	ns
SORD	-0.01174443	-0.03154459	-0.77735848	0.436959203	0.604701836	-6.73082963	ns
TGM2	0.013212968	0.029870414	0.776729555	0.43733037	0.604928792	-6.7244493	ns
MEP1B	0.020330493	-0.20000856	0.776096181	0.437704189	0.605114052	-6.72611728	ns
BTLA	-0.01362361	0.116180113	-0.7758009	0.437878764	0.605114052	-6.71627616	ns
IGF2BP3	0.007563838	0.075782969	0.775228966	0.438216569	0.60529441	-6.71841542	ns
ANGPTL1	-0.00491277	0.012677382	-0.77351061	0.439232405	0.606410696	-6.71932931	ns
AIF1L	-0.00921418	0.121793856	-0.77278435	0.439662132	0.606717119	-6.72113245	ns
TIGAR	-0.00794948	-0.02390478	-0.77153094	0.440404418	0.60745437	-6.72163901	ns
BTN1A1	0.006240678	0.047315714	0.771089393	0.440665761	0.607527867	-6.73433388	ns
CLSTN3	-0.00478874	0.002806415	-0.76992637	0.441355305	0.60819136	-6.73720355	ns
AMIGO2	-0.00375732	-0.01109965	-0.76681067	0.443205927	0.610453447	-6.73277517	ns
AKAP12	0.003019953	0.01417554	0.766315482	0.443500552	0.610571246	-6.72704031	ns
NT5C3A	-0.01482578	-0.05079403	-0.76570268	0.443865008	0.610785026	-6.73636147	ns
TBCC	-0.01383926	-0.01503337	-0.76528758	0.444112147	0.610837243	-6.73358517	ns
HGFAC	0.004341681	-0.02443488	0.764488534	0.444587956	0.611203782	-6.73491057	ns
VTA1	-0.01204146	0.019069895	-0.76334199	0.44527108	0.611854849	-6.74320633	ns
DOCK9	0.018267525	0.374213515	0.762734689	0.44563357	0.612064922	-6.7297536	ns
NTF3	-0.00590161	0.011981507	-0.76168405	0.446260306	0.612637562	-6.73940687	ns
ATXN3	-0.00992726	0.011150893	-0.76112025	0.446596824	0.612798773	-6.74389341	ns
OBP2B	0.010598113	-0.03002272	0.760784706	0.446797462	0.612798773	-6.73476947	ns
XPNPEP2	-0.01632711	-0.08891704	-0.75928473	0.447693843	0.613646648	-6.74298701	ns
DCXR	-0.00906747	0.019268869	-0.75873223	0.448024336	0.613646648	-6.74340315	ns
CNTN2	-0.00749603	0.041309625	-0.75820508	0.448339732	0.613646648	-6.74772541	ns
CHAD	-0.00782335	0.005613369	-0.75810436	0.448400306	0.613646648	-6.73324991	ns
RAB37	-0.01400979	0.193401466	-0.75784881	0.44855332	0.613646648	-6.73334731	ns
IL31	-0.00865503	0.107510903	-0.75764305	0.448676573	0.613646648	-6.73124325	ns
MMP8	-0.01223444	-0.03214886	-0.7548403	0.45035629	0.615655602	-6.74875909	ns

CA9	0.007207417	0.034974485	0.754121668	0.450787609	0.615956864	-6.75078966	ns
CADPS	-0.00664831	0.059040309	-0.7536061	0.451097519	0.616092027	-6.73629353	ns
SMPD3	-0.00919191	0.115722524	-0.74853904	0.454146794	0.619966637	-6.73804547	ns
BDNF	-0.01262114	-0.09279052	-0.74693443	0.455114485	0.620997335	-6.75400006	ns
CCL13	-0.01132234	-0.01076245	-0.74604013	0.455654484	0.621443761	-6.75537726	ns
PSMD1	-0.00753331	0.032713185	-0.74361893	0.4571183	0.62314913	-6.75794728	ns
FGFR4	0.008005571	0.040690262	0.743201849	0.457371012	0.623202685	-6.74434896	ns
IL1R2	0.003161958	-0.01122243	0.740022431	0.459297746	0.625458525	-6.75200512	ns
TFAP2A	-0.00924326	0.062959625	-0.73976358	0.459454978	0.625458525	-6.74687554	ns
CSNK2A1	-0.00681177	0.038722309	-0.73780923	0.460641539	0.626781591	-6.76221786	ns
S100A4	-0.0092286	-0.02685058	-0.73633076	0.461540619	0.627712439	-6.76002065	ns
WFDC1	-0.00834315	0.056233955	-0.73354791	0.463235676	0.629724476	-6.75411942	ns
IGSF21	-0.00596108	0.029836018	-0.73134665	0.464578808	0.631218164	-6.7520519	ns
BTD	-0.00308425	-0.0210061	-0.73103832	0.464766799	0.631218164	-6.76591314	ns
GBA	-0.00812456	-0.02220117	-0.72876216	0.466158454	0.632634887	-6.75456325	ns
TMED10	-0.00758412	0.0723704	-0.72827591	0.466456048	0.632634887	-6.7534352	ns
CHRDL2	0.008691035	0.019447355	0.727991956	0.466629645	0.632634887	-6.76201431	ns
KLRC1	0.009024224	0.097034294	0.727682594	0.466819207	0.632634887	-6.75567027	ns
KIF20B	0.011516558	0.062821701	0.727561728	0.466893213	0.632634887	-6.75575753	ns
STX5	0.006098805	0.040134448	0.726455918	0.467570631	0.633258925	-6.75420366	ns
RNF43	0.008829406	0.106206631	0.726050464	0.467818965	0.633301519	-6.76192759	ns
ACHE	0.004799198	0.032052004	0.724942957	0.468498226	0.633927164	-6.75764742	ns
MYH4	0.00694067	0.062714261	0.724533087	0.468749798	0.633973789	-6.75613265	ns
MTR	-0.00958767	0.098553785	-0.72199362	0.47030963	0.635506219	-6.75795493	ns
CCDC28A	-0.00434063	-0.00903071	-0.7219797	0.47031813	0.635506219	-6.75944453	ns
MAG	0.00693432	-0.02653284	0.720133203	0.471454121	0.636725781	-6.76076553	ns
TSPAN8	-0.01456935	-0.01467177	-0.71980361	0.471656803	0.636725781	-6.77314754	ns
RGL2	-0.00936125	0.101106061	-0.71853905	0.472435978	0.637482928	-6.76731102	ns
ENG	-0.00268389	0.000188558	-0.71726426	0.473221859	0.63824842	-6.77658223	ns
BLMH	0.0049263	-0.00352135	0.715730896	0.474168303	0.639229661	-6.77767251	ns
IGF2R	-0.00370733	-0.01476592	-0.71532546	0.474418824	0.63927225	-6.77376373	ns
CD40LG	-0.01230059	-0.04097146	-0.71488601	0.474690226	0.639342925	-6.77871077	ns
HMOX1	0.007286636	0.017567842	0.714271893	0.475070104	0.639559568	-6.76570983	ns
TLR1	-0.00379593	0.003647543	-0.71245868	0.476191848	0.640704501	-6.76419491	ns
FGL1	-0.00904627	-0.03821862	-0.71189408	0.476541099	0.640704501	-6.77874523	ns
BRSK2	-0.0056867	0.068094003	-0.7118331	0.476578828	0.640704501	-6.78090263	ns
CSF2RA	0.009692736	-0.05182923	0.710208388	0.477585766	0.641762741	-6.77390537	ns
KLHL41	0.007275242	0.073846963	0.708658893	0.478546822	0.642486383	-6.78313995	ns
MORF4L1	-0.00588864	0.042431567	-0.70863095	0.478564344	0.642486383	-6.77432654	ns
COCH	-0.0066016	-0.01111773	-0.70776391	0.479102531	0.642913323	-6.78283683	ns
FARSA	-0.0101523	0.118898617	-0.70691472	0.479630375	0.643325997	-6.77012339	ns
APP	-0.01027648	0.003109772	-0.70642159	0.479936707	0.643441316	-6.78004465	ns
ADGRF5	-0.00406884	-0.02799286	-0.70579511	0.480326179	0.643667941	-6.78421773	ns
DNAJB2	-0.00803002	0.015920004	-0.70523737	0.480673373	0.643835308	-6.77162844	ns
PDIA2	-0.00594012	0.033149884	-0.70488609	0.480892057	0.643835308	-6.76836367	ns
VWA1	-0.0066118	-0.01039964	-0.70449456	0.481135421	0.643865916	-6.7859426	ns
ERVV-1	0.006583343	0.046614404	0.704111742	0.481374095	0.643890224	-6.77060553	ns
DCLRE1C	-0.00459407	0.012530805	-0.70242931	0.482422353	0.644755377	-6.77007878	ns
PNPT1	0.018947305	0.41178701	0.702364823	0.482462499	0.644755377	-6.77353948	ns
SH2D1A	0.008596367	0.05250215	0.700579328	0.483576205	0.645940356	-6.78373902	ns
ZNF75D	-0.00889401	0.052044865	-0.70023436	0.48379163	0.645940356	-6.78019557	ns
PNLIPRP2	-0.04625407	-0.91199875	-0.69985424	0.484028961	0.645961867	-6.78189664	ns
TTN	-0.00958978	0.133064876	-0.69924979	0.484406757	0.646170732	-6.77399028	ns
ACTN4	-0.0048382	0.020719715	-0.69611601	0.486366888	0.648243761	-6.77787471	ns
IL25	0.004896018	-0.01733542	0.696055596	0.486404822	0.648243761	-6.77247099	ns
TGFB2	-0.00760848	-0.02043669	-0.69475021	0.487222305	0.648909766	-6.79090167	ns
MTSS2	-0.0142007	-0.01262639	-0.69454807	0.487349012	0.648909766	-6.79104101	ns
IL20RB	0.00601639	0.00850253	0.690307298	0.490011615	0.652019113	-6.77960939	ns
ENTPD2	0.006475091	-0.0141158	0.690117723	0.490130806	0.652019113	-6.78110083	ns
FOLH1	-0.01555682	0.318674446	-0.68941234	0.490574462	0.652312127	-6.78022194	ns
MILR1	-0.00767548	0.004733664	-0.68844499	0.49118317	0.65282424	-6.78314366	ns
GFOD2	0.019329778	0.48734443	0.687193642	0.491971304	0.653574253	-6.78309873	ns
RGS8	-0.00996525	0.149895962	-0.68576776	0.492870094	0.654470521	-6.78496882	ns
CTSC	-0.00628	0.006153173	-0.68473468	0.493521736	0.654664324	-6.79203657	ns
NOS2	-0.00564533	0.013017128	-0.68444769	0.49370306	0.654664324	-6.7841507	ns
F3	-0.00403405	0.001808745	-0.68418456	0.493868913	0.654664324	-6.797478	ns

TIMP4	-0.00577139	-0.00530411	-0.6840955	0.493925179	0.654664324	-6.79544787	ns
LRRN1	0.005943447	0.029754759	0.683760273	0.494137044	0.654664324	-6.78633272	ns
NPR1	0.005919561	0.083365112	0.681993416	0.495253622	0.655846066	-6.78581435	ns
SMAD3	-0.00723464	0.055930727	-0.68160712	0.49549763	0.655871749	-6.7998774	ns
SPINK8	-0.00531309	0.036054929	-0.68106602	0.495840205	0.65594443	-6.78644143	ns
LYAR	-0.00823242	0.053242901	-0.68063736	0.496111455	0.65594443	-6.78754753	ns
ECM1	-0.00515568	-0.05004231	-0.68039611	0.496264147	0.65594443	-6.78633192	ns
THRAP3	0.015985918	0.382559705	0.68010083	0.496451093	0.65594443	-6.78709317	ns
CLEC2L	-0.0073163	0.095013194	-0.67828476	0.497601544	0.656717685	-6.78979729	ns
S100G	-0.00634664	0.06627761	-0.678212	0.497647393	0.656717685	-6.8040923	ns
EVPL	-0.00461018	0.029015047	-0.67811187	0.49771104	0.656717685	-6.79532375	ns
TADA3	-0.00550045	0.008539283	-0.67468395	0.499886681	0.659290474	-6.80453647	ns
GUCY2C	0.011449976	0.227597508	0.674316451	0.500120357	0.659300877	-6.7978704	ns
CDHR2	-0.0116205	-0.17423928	-0.67031126	0.50266951	0.66236235	-6.80157994	ns
UPK3BL1	0.007425382	0.070514426	0.669897323	0.502933402	0.662411156	-6.79945345	ns
FLT1	-0.00312949	-0.00074714	-0.66893287	0.503548417	0.662658728	-6.80137762	ns
EDDM3B	-0.00703427	0.185020185	-0.66889062	0.503575246	0.662658728	-6.80839864	ns
ATP1B1	-0.00672059	0.0610765	-0.66608838	0.505364831	0.664707672	-6.79645085	ns
RASGRF1	0.006074692	0.009859614	0.665701309	0.505612257	0.664707672	-6.79500524	ns
SLC28A1	0.008247381	0.097488772	0.665329666	0.505849874	0.664707672	-6.79525062	ns
SPTBN2	-0.0043701	0.035285245	-0.66423906	0.506547473	0.664707672	-6.7994791	ns
S100A12	0.01101999	-0.022183	0.664093562	0.506640535	0.664707672	-6.80193943	ns
MAPRE3	-0.02025117	0.546414276	-0.66406286	0.506660272	0.664707672	-6.79608604	ns
APOH	-0.0052627	0.002796025	-0.66396014	0.50672578	0.664707672	-6.80891548	ns
PGA4	0.008581471	0.005538634	0.660006614	0.509259405	0.667731236	-6.81406559	ns
BCHE	-0.00387652	-0.02785392	-0.6595844	0.509530599	0.667786961	-6.80364486	ns
ANKRD54	-0.00873741	0.086611443	-0.6578398	0.510651142	0.66866787	-6.80268499	ns
SLC4A1	0.006606521	0.081927052	0.657661073	0.510765718	0.66866787	-6.81621814	ns
COQ7	0.00592954	0.063308906	0.657468535	0.510889732	0.66866787	-6.80295135	ns
IL21R	0.007182822	0.098147031	0.656720817	0.511370498	0.668997246	-6.80205262	ns
NDUFA5	0.00473979	-0.00320435	0.655731447	0.512006984	0.669529956	-6.80142672	ns
BCL2L15	-0.00765416	0.052175926	-0.65485382	0.512571893	0.669968634	-6.80326735	ns
LAMB1	-0.00403616	-0.02319061	-0.65178306	0.514551063	0.672254632	-6.80664405	ns
RAB6A	-0.00662248	0.055474091	-0.64948416	0.516035202	0.673892124	-6.81796491	ns
TSNAX	-0.00533069	0.028248271	-0.6468684	0.517726995	0.675735043	-6.80896934	ns
GBP6	0.005780997	0.046211873	0.64658683	0.517909256	0.675735043	-6.80915001	ns
CCL24	0.011944076	0.04480936	0.644813774	0.519057491	0.676692961	-6.82098566	ns
CRNN	-0.00949664	0.007228069	-0.6446536	0.519161607	0.676692961	-6.80743854	ns
EPHX2	-0.00859464	0.095137112	-0.64436436	0.519349001	0.676692961	-6.81230989	ns
MMP13	-0.0044473	-0.00648146	-0.64402285	0.519570418	0.676692961	-6.81249538	ns
S100A16	-0.00881712	0.050048385	-0.64281006	0.520357151	0.677415462	-6.81257854	ns
PSMD9	0.008145657	0.050005513	0.642216621	0.520742166	0.677614583	-6.81987504	ns
MYLPF	0.006227524	0.075472313	0.641687545	0.521085808	0.677759715	-6.81376196	ns
IL6ST	-0.00244503	-0.00855254	-0.64117256	0.521420176	0.677838686	-6.82099055	ns
GMPR	-0.00710245	-0.0478651	-0.64044222	0.52189459	0.677838686	-6.82877496	ns
COL5A1	-0.00500877	0.026862329	-0.640417	0.521911223	0.677838686	-6.81490028	ns
SH3BGRL2	-0.00812257	0.113726286	-0.64016499	0.52207507	0.677838686	-6.8150575	ns
SUMF1	0.008788336	0.130165319	0.63773623	0.523655109	0.679587963	-6.81424347	ns
RNF168	0.005438437	0.034100253	0.636780843	0.524277301	0.679894463	-6.81484751	ns
ACYP1	-0.00874376	-0.00051881	-0.63648374	0.524470842	0.679894463	-6.81526474	ns
RELB	0.006400675	0.077298736	0.636301209	0.524589803	0.679894463	-6.81515042	ns
CA14	0.005042356	0.057241502	0.634938221	0.525478068	0.680743548	-6.83293729	ns
MFAP3L	0.00872634	0.15983596	0.634212459	0.525951776	0.681055071	-6.81849339	ns
CRYGD	0.012536367	-4.33E-05	0.633825317	0.526204182	0.681079881	-6.83200142	ns
IL5	0.020218187	0.307193057	0.632355428	0.527164222	0.681789676	-6.81989554	ns
CCNE1	-0.01245853	0.258550342	-0.63166341	0.527616444	0.681789676	-6.81806761	ns
DKK1	-0.00826171	-0.04478916	-0.6311563	0.527947634	0.681789676	-6.83467158	ns
SPARCL1	-0.00385105	0.006737745	-0.63096239	0.52807455	0.681789676	-6.82660884	ns
RGS10	-0.00770937	0.050055733	-0.63059433	0.528315093	0.681789676	-6.83501654	ns
HBZ	-0.01373488	0.091404729	-0.63051295	0.528368336	0.681789676	-6.83413459	ns
MYBPC2	0.010625325	0.18236127	0.630484802	0.528386999	0.681789676	-6.82019213	ns
ENSA	-0.00665948	0.040915338	-0.62823933	0.529856453	0.683383764	-6.83456243	ns
MMUT	-0.00651019	0.052119822	-0.62690128	0.530733471	0.68361494	-6.82158633	ns
PIK3AP1	-0.00853581	0.0190266	-0.62681944	0.530786928	0.68361494	-6.83231738	ns
ATP2B4	-0.00806159	0.152626899	-0.62677475	0.530816418	0.68361494	-6.82166502	ns
RNF31	-0.00471447	0.023818947	-0.62653721	0.530972152	0.68361494	-6.82126718	ns

FEN1	-0.00890307	0.090077812	-0.62567843	0.531535377	0.683871189	-6.82316318	ns
TXK	-0.00979229	0.224679134	-0.62551937	0.531639589	0.683871189	-6.82933906	ns
IL18RAP	0.023989888	0.437480727	0.624982305	0.531992066	0.684023264	-6.82741769	ns
NAAA	-0.00818266	0.052243935	-0.62398333	0.532647915	0.684565102	-6.82851287	ns
DIPK2B	-0.00343268	0.021271387	-0.62260702	0.533552173	0.685425581	-6.82605958	ns
SIGLEC7	-0.0038224	-0.03691398	-0.61931577	0.535717573	0.687904711	-6.8346594	ns
IL22RA1	-0.00589126	0.035876052	-0.61798815	0.536592463	0.688725272	-6.82880733	ns
CEP350	-0.00478331	0.013706483	-0.61751001	0.536907743	0.688827157	-6.82568162	ns
NMNAT1	-0.01013983	0.037024758	-0.61567588	0.538117639	0.6898054	-6.83609056	ns
ACRBP	-0.00482686	0.029913228	-0.61563774	0.538142706	0.6898054	-6.84234005	ns
AARSD1	-0.00772008	0.006943245	-0.61448256	0.538905631	0.690480229	-6.83716717	ns
SERPINE1	-0.00922344	-0.09002708	-0.61363641	0.539464619	0.690636782	-6.84501654	ns
CNTN5	0.004974054	0.045766772	0.61358164	0.539500856	0.690636782	-6.84347234	ns
ERBB3	0.002585764	0.004432797	0.613100415	0.539819078	0.690741326	-6.83701698	ns
AAMDC	-0.00668536	0.012003783	-0.61207229	0.540499159	0.691308605	-6.83154185	ns
NME1	-0.00266657	0.006782124	-0.61135797	0.540971864	0.691610264	-6.83058856	ns
ALDH2	-0.00658544	0.109272298	-0.61074031	0.541380502	0.691829788	-6.84532019	ns
NOP56	-0.01365315	0.32764567	-0.60893555	0.542576364	0.692921172	-6.83260061	ns
EDEM2	-0.00459907	0.051127624	-0.60840321	0.542929275	0.692921172	-6.83292208	ns
NSFL1C	-0.00900765	-0.01148979	-0.60837688	0.542946452	0.692921172	-6.8486822	ns
SAG	0.006595407	0.010505829	0.607389272	0.543601753	0.693454399	-6.83534245	ns
TLR4	-0.00354299	0.003920223	-0.60682683	0.543975005	0.693627517	-6.83332677	ns
DNPEP	0.00698351	0.009947578	0.60596398	0.544547546	0.694054489	-6.84914099	ns
ATP1B3	-0.00496418	0.053492184	-0.60501383	0.545178937	0.69454141	-6.83496232	ns
IMPG1	-0.00391309	0.031766187	-0.60437778	0.545601602	0.69454141	-6.83715281	ns
TG	0.013696995	0.198544512	0.60431494	0.545643149	0.69454141	-6.849194	ns
IL9	-0.00699882	0.088876583	-0.60344747	0.546220017	0.694963675	-6.84279696	ns
BCAM	-0.00312677	0.002000712	-0.60310027	0.546450889	0.694963675	-6.84717621	ns
BCL2	-0.00654972	0.027505554	-0.60232394	0.546967361	0.69516817	-6.85021607	ns
ITIH1	-0.00229684	-0.0184061	-0.60214291	0.547087827	0.69516817	-6.85117861	ns
BRK1	-0.00579185	-0.01238464	-0.601393	0.547587306	0.695500188	-6.8388338	ns
HRAS	0.011839759	0.270112446	0.598243738	0.549686202	0.697862482	-6.84489475	ns
PCARE	-0.01028578	0.190017493	-0.59718591	0.550392231	0.698455156	-6.84143978	ns
PGR	-0.01279153	0.317079291	-0.59255908	0.553485183	0.701977871	-6.84181587	ns
CEP290	-0.00655134	0.057710574	-0.59231419	0.553648985	0.701977871	-6.84940185	ns
REG1B	-0.0071634	0.042539651	-0.59098812	0.55453699	0.702798615	-6.85855412	ns
AMY2B	-0.00634966	0.02244243	-0.58897907	0.555884752	0.704201074	-6.813011	ns
HEPH	0.003224573	-0.01941702	0.586154624	0.557780153	0.70629577	-6.86091381	ns
IL33	-0.00494031	-0.01659228	-0.58407196	0.559180624	0.707762211	-6.84903267	ns
LCP1	-0.00573434	0.058434104	-0.58310453	0.559831424	0.708278924	-6.86211912	ns
SDK2	0.004534324	0.019304664	0.582463981	0.560262867	0.708313171	-6.85005817	ns
FBN2	-0.00710844	-0.03299428	-0.58200238	0.560573759	0.708313171	-6.84999445	ns
DIPK1C	-0.00507894	0.053822815	-0.58198389	0.560586211	0.708313171	-6.85000512	ns
LILRA2	-0.0046776	-0.03608417	-0.5799099	0.56198388	0.709772029	-6.86078822	ns
IL12RB2	0.005034491	0.076340952	0.579317924	0.562383353	0.709969472	-6.84951346	ns
PPY	0.012875471	0.035545384	0.577248124	0.563780284	0.711425424	-6.86309594	ns
RBKS	-0.00650678	0.009818999	-0.57647064	0.564305754	0.711780908	-6.85064373	ns
ARID3A	-0.00475414	0.033694877	-0.57478403	0.565445945	0.712911122	-6.85446439	ns
GGA1	-0.00877412	0.162136785	-0.57386798	0.566065708	0.713293497	-6.85488194	ns
GAMT	-0.00430483	0.048941785	-0.57361376	0.566237783	0.713293497	-6.85332171	ns
AZU1	-0.00977643	0.066564546	-0.57223172	0.56717372	0.714164408	-6.84526034	ns
MN1	0.006879232	0.082549626	0.571512684	0.567660662	0.714469454	-6.85599716	ns
IL32	0.005147085	0.009635475	0.57065602	0.568241193	0.714891979	-6.86568641	ns
CASP10	-0.00910461	-0.00639692	-0.56982534	0.568804664	0.715292687	-6.85717565	ns
RBP7	-0.00717778	0.042330818	-0.56889117	0.569438263	0.7157812	-6.86964518	ns
SOX9	-0.00310204	-0.01590623	-0.56763403	0.570291926	0.716249513	-6.85818907	ns
KYAT1	-0.00704204	0.009767978	-0.56761993	0.57030141	0.716249513	-6.86416846	ns
TMCO5A	-0.00637469	0.040031296	-0.56677651	0.570874446	0.716660956	-6.85867166	ns
FKBPL	-0.00686814	0.027985797	-0.56521529	0.57193554	0.717684476	-6.8717137	ns
NID1	-0.00471922	-0.02001742	-0.56474448	0.572255862	0.717777972	-6.86647541	ns
CCN2	0.007195177	-0.01454191	0.559585109	0.575770647	0.721699012	-6.87003316	ns
HDAC8	-0.00723738	0.127582516	-0.55909708	0.576103792	0.721699012	-6.86093298	ns
BTNL10	0.004513041	0.049262722	0.559068326	0.576123424	0.721699012	-6.85979171	ns
PDGFA	-0.00881606	-0.04660387	-0.55791431	0.576910954	0.722303874	-6.87740894	ns
MRPL52	-0.00291425	-0.00228687	-0.55716156	0.577425367	0.722303874	-6.85946303	ns
IL6R	0.004206915	-0.02117648	0.556946522	0.577572185	0.722303874	-6.86688201	ns

LILRA3	0.015892623	-0.39521281	0.556907754	0.577598506	0.722303874	-6.87693768	ns
SERPINI1	0.00341743	0.028351068	0.556241464	0.578054033	0.722303874	-6.86382525	ns
RANBP1	-0.0061106	0.010970865	-0.55618819	0.578090464	0.722303874	-6.86392991	ns
KCNH2	0.008612623	0.214804303	0.55457972	0.579190369	0.723160349	-6.86545668	ns
DMP1	0.006831349	0.010928345	0.554461527	0.579271252	0.723160349	-6.86488126	ns
KIAA1549	0.007803933	-0.01014461	0.553595748	0.579863644	0.72359053	-6.87141423	ns
FES	-0.00576083	0.018532069	-0.55212746	0.580869234	0.724535738	-6.86613846	ns
PRR5	-0.00533073	0.03743917	-0.55117951	0.581518814	0.725036267	-6.86529384	ns
ADAMTSL4	-0.00264758	0.001425792	-0.55060085	0.58191548	0.725221171	-6.86699771	ns
LPO	0.007258495	-0.00400836	0.54848317	0.583368413	0.726721743	-6.86160047	ns
DDT	-0.00476075	0.017563997	-0.54690644	0.584450892	0.727328055	-6.88241542	ns
IL34	-0.00420618	-0.02777598	-0.54661777	0.584649467	0.727328055	-6.86933773	ns
H2AP	-0.00583311	0.062393304	-0.54636022	0.584826465	0.727328055	-6.86676041	ns
FOSB	-0.00474502	0.025276102	-0.54632381	0.584851463	0.727328055	-6.87018879	ns
CDAN1	-0.00256075	0.008007341	-0.54587184	0.585161894	0.727404312	-6.88348569	ns
NOS1	-0.0062565	-0.01122511	-0.54543344	0.585463522	0.727469568	-6.87067103	ns
ITGA6	-0.00633829	0.070687955	-0.54466588	0.585991254	0.727815594	-6.88006889	ns
CD5L	-0.00513152	0.013826106	-0.54259892	0.587413838	0.729272282	-6.88147464	ns
DLGAP5	-0.00584745	0.113455707	-0.5421823	0.587700708	0.729318346	-6.88646641	ns
CPQ	-0.00389301	-0.01981516	-0.54124651	0.588345487	0.729808336	-6.88472874	ns
ARNT	-0.00442959	0.047674603	-0.53940582	0.589614819	0.731045609	-6.87392735	ns
LILRB5	0.008465887	-0.1654132	0.53907383	0.589843649	0.731045609	-6.88766427	ns
HSD11B1	-0.00479984	0.013495465	-0.53827421	0.59039556	0.731419192	-6.88350323	ns
TSPYL1	-0.00443684	0.030297154	-0.53709571	0.591209454	0.732116882	-6.87289723	ns
CEACAM18	-0.00727577	0.137896503	-0.52986804	0.596211135	0.737997675	-6.88416892	ns
C1QTNF9	0.005314369	-0.03522758	0.528933544	0.596859313	0.738486946	-6.87881718	ns
HS6ST1	-0.00380105	0.001433456	-0.52695889	0.598229668	0.739868967	-6.89519175	ns
BHLHE40	-0.00764346	0.084472553	-0.52553657	0.599217968	0.740777505	-6.88134678	ns
MYOC	-0.00531378	-0.01576526	-0.52484582	0.599697944	0.740995618	-6.89097121	ns
AKR1B10	-0.00643987	0.019895561	-0.52455261	0.599901931	0.740995618	-6.88153168	ns
HSD17B14	-0.00657665	-0.01463328	-0.52301181	0.600973514	0.742005353	-6.89561008	ns
PDE4D	0.00649103	0.136357609	0.520993438	0.602379017	0.743168419	-6.88189526	ns
AHSA1	-0.00720248	0.104091082	-0.5209278	0.602424537	0.743168419	-6.89575164	ns
ELAVL4	-0.00901824	0.071513876	-0.51961138	0.603342089	0.743986022	-6.88441895	ns
DNER	0.002988404	0.008813408	0.519015571	0.603757389	0.744183864	-6.8911136	ns
RARRES1	0.003806213	0.017547976	0.51863568	0.604022452	0.744196439	-6.88392904	ns
SERPINC1	-0.00155968	0.002459017	-0.51757004	0.604765698	0.744797908	-6.89653855	ns
GHR	-0.00329653	-0.02835094	-0.51714827	0.605060214	0.744846469	-6.88568867	ns
C5	-0.00158225	-0.00710707	-0.515384	0.606292067	0.746048392	-6.89928717	ns
GLI2	-0.00353092	0.027447968	-0.51416228	0.607146095	0.746422018	-6.88485713	ns
CPOX	0.002766516	0.008684269	0.513818157	0.607386683	0.746422018	-6.88503262	ns
PRKAG3	-0.00343188	0.025996868	-0.51327113	0.607769175	0.746422018	-6.88734003	ns
TPRKB	0.006748421	0.082619474	0.51313113	0.607867142	0.746422018	-6.88422495	ns
PARD3	-0.00778815	0.088625529	-0.51312141	0.607873822	0.746422018	-6.8928347	ns
SERPINA5	0.003406715	0.018690177	0.511995023	0.608661913	0.747075572	-6.89271029	ns
TGFBI	-0.00300609	-0.01174741	-0.51047245	0.609727939	0.748069572	-6.8914929	ns
IVD	-0.00549113	-0.00277769	-0.50760797	0.611735729	0.750137517	-6.89043138	ns
LARP1	-0.00666779	0.079535597	-0.50711438	0.612081805	0.750137517	-6.9027979	ns
ZPR1	-0.00996071	0.223972318	-0.50696877	0.612184145	0.750137517	-6.89085761	ns
TXNL1	-0.01047112	0.30466603	-0.50606997	0.612814944	0.750479571	-6.89049526	ns
TPMT	0.008050233	0.073347866	0.505838671	0.612977321	0.750479571	-6.89043286	ns
PTH1R	0.004247363	0.036501877	0.504500258	0.613917125	0.751315174	-6.90097747	ns
SSB	-0.00625714	0.078825803	-0.50263562	0.61522783	0.752205374	-6.89292341	ns
CST1	0.010501533	0.048081719	0.502537161	0.615297911	0.752205374	-6.83664612	ns
RAP1A	0.006922742	0.117862017	0.501988324	0.615683085	0.752205374	-6.8909939	ns
GKN1	-0.00300689	-0.02270903	-0.50176212	0.615842221	0.752205374	-6.89174226	ns
BLNK	-0.0039088	0.001154452	-0.5016337	0.615932551	0.752205374	-6.89117045	ns
FUT3_FUT5	-0.00494636	-0.02595265	-0.50030951	0.616864261	0.752796684	-6.9081336	ns
CDH4	-0.00483617	0.042352424	-0.4999174	0.617140536	0.752796684	-6.89437986	ns
CD6	0.00587847	-0.03912866	0.499796783	0.617225267	0.752796684	-6.90769335	ns
CELA3A	0.006145886	0.035984228	0.499480576	0.617447965	0.752796684	-6.90690021	ns
CD209	0.004268818	0.0383518	0.497035316	0.619171249	0.754582657	-6.90495438	ns
RLN1	0.007332901	0.178978	0.49608733	0.619839822	0.754989885	-6.91021422	ns
NINJ1	-0.0062425	0.09235126	-0.49564169	0.620154475	0.754989885	-6.89549896	ns
SMC3	0.005582961	0.048593028	0.495448613	0.620290734	0.754989885	-6.89307195	ns
UXS1	-0.00557099	0.002101821	-0.49509594	0.620539631	0.754989885	-6.89576719	ns

TCP11	-0.0060576	0.119664768	-0.49317923	0.621893045	0.756321404	-6.90279108	ns
RPS10	0.003984711	0.03458539	0.491801167	0.622867093	0.757190637	-6.89656232	ns
PTK7	-0.00400701	-0.0118655	-0.48874818	0.625027101	0.759463662	-6.89975355	ns
DARS1	-0.00793329	-0.04285026	-0.48836602	0.625297634	0.759463662	-6.90452369	ns
ECE1	0.003753912	-0.01220863	0.488056166	0.62551716	0.759463662	-6.90008889	ns
GPR101	0.006088221	0.112691005	0.487213158	0.626114365	0.759872795	-6.90060128	ns
KLF4	-0.00412185	0.003301661	-0.48482203	0.627809635	0.760907071	-6.90175434	ns
SHPK	-0.00417608	0.068751513	-0.48467174	0.627916171	0.760907071	-6.90691847	ns
APRT	-0.00516053	-0.04580861	-0.48462456	0.62794964	0.760907071	-6.90763184	ns
ADAM15	-0.0032312	0.009517776	-0.48435766	0.628139103	0.760907071	-6.90190592	ns
ECSCR	-0.0051593	0.102597749	-0.48417388	0.628269503	0.760907071	-6.90206592	ns
PODXL	-0.00160357	-0.00496073	-0.48344982	0.628783302	0.761177319	-6.90854475	ns
CD200	-0.00277859	0.016014069	-0.4829193	0.629159928	0.761177319	-6.91155049	ns
CSNK1D	-0.00630053	0.102801037	-0.4827579	0.629274674	0.761177319	-6.90038818	ns
ANXA2	-0.00707498	0.022895266	-0.48105231	0.630486406	0.761854504	-6.89541147	ns
TERF1	-0.00279769	0.013963673	-0.48067763	0.63075263	0.761854504	-6.90138242	ns
WIF1	-0.00362491	0.005403827	-0.48060956	0.630800791	0.761854504	-6.91836477	ns
MEGF9	0.002208763	-0.00403363	0.480500788	0.630878147	0.761854504	-6.9151895	ns
NID2	-0.00788586	0.02735187	-0.47914436	0.631842754	0.762703944	-6.91373731	ns
DEFB118	-0.00574817	0.098682971	-0.47844361	0.632341437	0.762990494	-6.90480242	ns
KEL	0.003795537	-0.00622713	0.477332793	0.633132086	0.763406439	-6.90522459	ns
SNX18	-0.00434731	0.09396236	-0.47675097	0.633546202	0.763406439	-6.91745068	ns
DCN	-0.00222786	-0.00308751	-0.47641093	0.633788557	0.763406439	-6.90569361	ns
FGF6	-0.00388889	0.044644467	-0.47626511	0.633892439	0.763406439	-6.90347713	ns
PDIA3	-0.00251578	-0.00153876	-0.47612336	0.633993361	0.763406439	-6.90590405	ns
FUCA1	-0.00868033	-0.2108768	-0.47513886	0.634694721	0.763935938	-6.9176663	ns
EDF1	-0.00671278	0.083597385	-0.46944097	0.638761061	0.768257882	-6.92298049	ns
ABHD14B	-0.00588016	0.007687654	-0.4693703	0.638811691	0.768257882	-6.91354977	ns
PDCD6	-0.00725184	-0.18521749	-0.46689505	0.640581608	0.770069286	-6.91680186	ns
SCN3A	0.003534352	0.037731567	0.465418529	0.641638473	0.771022362	-6.91057355	ns
NEO1	-0.00211756	0.00300611	-0.46442875	0.642347108	0.771556378	-6.9253031	ns
LRP2BP	-0.00791571	0.218250407	-0.46142269	0.644502014	0.773826431	-6.91241084	ns
TNFSF14	-0.00597571	-0.01293491	-0.46004054	0.645493675	0.774698533	-6.91915409	ns
TMOD4	0.003824667	-0.0037945	0.459481251	0.645895261	0.774862023	-6.91011011	ns
MANEAL	0.00480149	-0.01158438	0.457688629	0.647182777	0.775588348	-6.91092572	ns
SERPINF2	-0.00128376	0.00282687	-0.45758244	0.64725901	0.775588348	-6.91735633	ns
CASQ2	0.004517197	0.063475369	0.457528916	0.647297536	0.775588348	-6.91270272	ns
DNLZ	0.008521474	0.235143263	0.454336952	0.649592864	0.778019345	-6.91595778	ns
IL2RB	-0.00372234	0.031361709	-0.45392914	0.649886354	0.778051723	-6.91604936	ns
TPT1	-0.00658693	0.091322776	-0.45320613	0.650406825	0.778355709	-6.91637473	ns
PRKCQ	-0.00432761	0.043364403	-0.45160852	0.651557511	0.778955559	-6.91709186	ns
VAMP5	-0.00529763	0.07240875	-0.45125513	0.651812172	0.778955559	-6.91727062	ns
SMNDC1	-0.00579228	0.040771788	-0.45111531	0.651912771	0.778955559	-6.92925327	ns
BST1	0.004743636	-0.13496272	0.451028754	0.651975132	0.778955559	-6.93139701	ns
GSR	-0.002054	-0.00752614	-0.44843358	0.653846629	0.780872048	-6.92714872	ns
GLRX	0.005721075	0.00327093	0.447567876	0.654471457	0.781298714	-6.92326633	ns
PLCB1	-0.00417317	0.069120084	-0.44657863	0.65518576	0.781715889	-6.91706771	ns
TJP3	-0.00383215	0.033083203	-0.44634241	0.655356334	0.781715889	-6.91952995	ns
TMEM25	-0.00402534	0.030939852	-0.44537741	0.656053245	0.782189986	-6.93390385	ns
CPA1	-0.00572289	0.018610704	-0.4450504	0.656289543	0.782189986	-6.93357809	ns
SERPINE2	-0.0058127	0.019631934	-0.44356895	0.657360458	0.78265936	-6.93376727	ns
DENND2B	0.004532377	0.084360074	0.443520647	0.657395572	0.78265936	-6.91896437	ns
SIRT1	-0.00811199	0.197516286	-0.44339357	0.657487469	0.78265936	-6.91847391	ns
ITPRIP	-0.00420934	0.085454469	-0.4424731	0.658153235	0.782949781	-6.92123608	ns
PF4	-0.00875782	-0.05642136	-0.44231471	0.658267709	0.782949781	-6.93005911	ns
RAB39B	-0.00827721	0.211991087	-0.43992036	0.660001113	0.783859185	-6.92054223	ns
NCLN	-0.00526502	0.102801357	-0.4397372	0.660133747	0.783859185	-6.92234129	ns
TNPO1	-0.00322675	0.009632085	-0.43969533	0.660164011	0.783859185	-6.92754524	ns
CRTAM	-0.00484668	-0.01534863	-0.43953593	0.660279473	0.783859185	-6.92830567	ns
PPM1A	-0.00304723	0.023008063	-0.43940486	0.660374519	0.783859185	-6.92158538	ns
BOLA2_BOLA2	0.005034145	-0.02938422	0.436815852	0.662251157	0.785427419	-6.92273492	ns
SEZ6L2	0.002232916	0.005849029	0.436501906	0.662478777	0.785427419	-6.92997318	ns
NPTXR	-0.00329664	0.046595402	-0.43646899	0.662502648	0.785427419	-6.93043893	ns
SERPINA12	-0.01057449	0.197030936	-0.4359229	0.662898991	0.78557835	-6.91737923	ns
DPEP2	-0.00247311	-0.00543528	-0.43515114	0.663458864	0.785922873	-6.93055734	ns
MTHFD2	0.002275837	0.013441766	0.433840754	0.664410123	0.786516281	-6.93794739	ns

IGDCC4	-0.0022975	0.023081224	-0.43337559	0.664747967	0.786516281	-6.93763785	ns
EIF4B	-0.00543958	-0.04025364	-0.43331323	0.66479335	0.786516281	-6.93100047	ns
PKLR	-0.0064639	0.06966314	-0.43297746	0.665037226	0.786516281	-6.93361112	ns
MRI1	0.006057948	-0.07440542	0.432331176	0.665506899	0.786753095	-6.92530965	ns
ICAM4	-0.00510919	-0.0441496	-0.43137037	0.666205153	0.787259833	-6.93493516	ns
PPIE	-0.00540065	0.125400629	-0.42944422	0.667606041	0.788596133	-6.93196565	ns
JMJD1C	0.005038693	0.054007275	0.428193594	0.668516318	0.789200767	-6.92388762	ns
PVR	-0.0033437	-0.01870719	-0.42799809	0.668658458	0.789200767	-6.9414425	ns
SMARCA2	-0.00600315	0.053405972	-0.42741634	0.669082216	0.789381847	-6.92762927	ns
HS3ST3B1	0.003503788	-0.00308259	0.426390224	0.669829523	0.789944349	-6.9343022	ns
NPL	0.003884801	-0.04347188	0.42432244	0.671336569	0.791402012	-6.94205572	ns
ACSL1	0.003214402	0.04898679	0.422112132	0.67294921	0.792982927	-6.92815776	ns
NAGK	-0.00493815	0.008383735	-0.42145722	0.673427265	0.793226145	-6.92814531	ns
MORC3	-0.00240216	0.020092046	-0.42046798	0.674149445	0.793481178	-6.9436162	ns
NTRK2	0.002336834	-0.00515125	0.420250287	0.674308521	0.793481178	-6.93733287	ns
PKD1	0.002207241	-0.01444359	0.420044187	0.674459001	0.793481178	-6.9438482	ns
OMP	-0.00418906	0.061522709	-0.41959409	0.674787986	0.793548498	-6.93102076	ns
NUDT10	0.002074484	0.022348909	0.419067001	0.675173136	0.793681786	-6.93027101	ns
TMED4	-0.00482359	0.08490461	-0.41703868	0.676656073	0.795104923	-6.93026979	ns
RUVBL1	0.009569929	0.285223451	0.412400546	0.68005174	0.798620547	-6.93299668	ns
EPHA10	0.005789068	0.094182104	0.412205096	0.680194966	0.798620547	-6.93396529	ns
GIMAP8	0.004324687	0.021448399	0.411646054	0.680604743	0.798780487	-6.93078166	ns
PLG	0.001873389	-0.0213721	0.411074912	0.681023456	0.79895078	-6.93326779	ns
S100A14	-0.00438837	0.084926831	-0.4102483	0.681629477	0.799340592	-6.94881851	ns
IGBP1	-0.00516978	0.021935775	-0.40814851	0.683170279	0.800825859	-6.94967122	ns
DUSP29	-0.00539017	0.098036284	-0.40544668	0.685154945	0.802830031	-6.93648335	ns
ALDH5A1	-0.00382891	0.044616667	-0.40223815	0.687514307	0.805271471	-6.95204789	ns
GH1	0.016319706	0.313304763	0.401808919	0.687830276	0.805318527	-6.94425001	ns
SHBG	-0.00443639	0.033412638	-0.40114747	0.688317091	0.805398489	-6.95036276	ns
MDM1	-0.00259841	0.006545231	-0.40094483	0.68846641	0.805398489	-6.9382843	ns
MYOM2	0.004070483	0.048417307	0.400592262	0.688726037	0.805398489	-6.9369407	ns
POLR2A	-0.00242628	0.012630235	-0.39847837	0.690283261	0.806896366	-6.93723212	ns
SOX2	0.001894798	-0.00078117	0.397730146	0.690834595	0.807067134	-6.95250311	ns
SIGLEC15	0.006958677	0.215549695	0.397530222	0.690982135	0.807067134	-6.93986031	ns
TAP1	-0.00483623	0.077547711	-0.39642464	0.691797333	0.807696206	-6.94549528	ns
CTSF	0.003479386	-0.00066035	0.395141574	0.692743869	0.808357784	-6.95156742	ns
IL20RA	-0.00394952	0.052029597	-0.39490626	0.692917648	0.808357784	-6.94090423	ns
FHIP2A	0.003876686	0.014918149	0.39414925	0.693476442	0.80852427	-6.93948092	ns
PPME1	-0.00535614	-0.01763579	-0.3939626	0.693614142	0.80852427	-6.94750075	ns
NFKB2	0.00461476	0.103181734	0.392853204	0.694433391	0.808931999	-6.94689382	ns
MAGEA3	0.006747988	-0.08175182	0.392714585	0.69453578	0.808931999	-6.94694785	ns
AMIGO1	-0.00413987	0.071392548	-0.39202278	0.695046908	0.808931999	-6.94179414	ns
CSPG5	-0.00394949	0.057561548	-0.39198877	0.69507205	0.808931999	-6.94213537	ns
ST13	-0.00456371	-0.0014959	-0.39062273	0.696081617	0.809784192	-6.9426686	ns
CAMLG	0.004360631	0.086588549	0.389314651	0.697048884	0.810280429	-6.94284386	ns
TF	-0.00173663	-0.00701648	-0.38929516	0.697063164	0.810280429	-6.95588822	ns
NRTN	-0.00420878	-0.00696535	-0.38651252	0.699122576	0.812350944	-6.94415815	ns
LACRT	-0.00391067	0.134445009	-0.38340748	0.701422942	0.814699678	-6.95657938	ns
ST8SIA1	-0.00392025	0.0905163	-0.38267188	0.701968477	0.815009126	-6.94335736	ns
SH3GLB2	0.005359254	-0.01082004	0.381055942	0.703166961	0.816076123	-6.95929099	ns
GNGT1	-0.00413943	0.068466667	-0.37923588	0.704518034	0.816571363	-6.94668665	ns
ITM2A	-0.00494224	-0.00060033	-0.37895792	0.704724352	0.816571363	-6.95600807	ns
IMPACT	-0.00442694	-0.00900124	-0.37815709	0.705319075	0.816571363	-6.96038305	ns
PRDX2	0.004971645	0.061656396	0.377968286	0.705459431	0.816571363	-6.95131768	ns
RABGAP1L	0.005022938	0.039207529	0.377960609	0.705465167	0.816571363	-6.9474015	ns
DTX2	-0.00502774	0.139973222	-0.3778386	0.705555803	0.816571363	-6.94721145	ns
JCHAIN	0.005140767	-0.03091229	0.377822116	0.705568052	0.816571363	-6.94745343	ns
DNAJC9	-0.00602611	-0.0158368	-0.37746821	0.705830864	0.816571363	-6.9595322	ns
EZR	-0.00203022	-0.02376248	-0.37141083	0.710336631	0.821458599	-6.9638891	ns
ITGB6	-0.00245673	-0.00077488	-0.36909328	0.712063373	0.823129473	-6.95068785	ns
IGLON5	-0.00694142	0.259901331	-0.36843806	0.712551812	0.823368141	-6.94750221	ns
DUSP3	-0.00697472	0.133040328	-0.36787819	0.712969229	0.823524585	-6.95111974	ns
GNAS	-0.006588	0.228743839	-0.36692531	0.713679733	0.823889965	-6.96552555	ns
PYY	-0.00720176	0.091013097	-0.36669745	0.713849867	0.823889965	-6.94984247	ns
GJA8	-0.00451913	0.059133844	-0.36507781	0.715058463	0.824667675	-6.94872493	ns
COL3A1	0.00169873	-0.00211634	0.364641561	0.715383966	0.824667675	-6.96485465	ns

IL17F	-0.00627839	0.155523911	-0.36435372	0.715598986	0.824667675	-6.9524124	ns
EBI3_IL27	-0.00242046	0.003729888	-0.36411998	0.715773385	0.824667675	-6.96642549	ns
YOD1	0.004811042	0.005495756	0.363891834	0.715943901	0.824667675	-6.95085933	ns
SFRP1	-0.0034167	-0.05033451	-0.36349739	0.716238418	0.824667675	-6.95597863	ns
PNLIPRP1	-0.005612	-0.00369922	-0.36257693	0.716925889	0.824667675	-6.96610958	ns
PNMA1	-0.00414703	0.061157013	-0.36252705	0.716963298	0.824667675	-6.95219397	ns
PDGFB	-0.00686601	-0.07750377	-0.36239012	0.717065489	0.824667675	-6.96329992	ns
KDR	-0.00203233	-0.01898273	-0.36026532	0.718653664	0.826150862	-6.96463973	ns
NEK7	-0.00551399	0.112703258	-0.35971172	0.719067703	0.826150862	-6.9592681	ns
SATB1	0.00224607	0.036292245	0.359529458	0.719203935	0.826150862	-6.96819155	ns
STC2	-0.00202907	-0.00342332	-0.35891347	0.719664749	0.826355119	-6.96373169	ns
RBP1	-0.00521098	0.140594665	-0.3580434	0.720315833	0.826777607	-6.95443807	ns
CPA2	-0.00505407	0.031789948	-0.35617255	0.721716218	0.827812552	-6.96691371	ns
REXO2	0.00367826	0.098420301	0.35608153	0.721784506	0.827812552	-6.95364893	ns
ENDOU	-0.0037922	0.032050028	-0.35539492	0.722298971	0.828077345	-6.93330132	ns
CLEC12A	-0.00140119	-0.00314861	-0.35297407	0.724112826	0.829698499	-6.9541953	ns
GOT1	-0.00291018	0.016197126	-0.35274913	0.724281327	0.829698499	-6.96908796	ns
SSC5D	-0.00343162	-0.05329165	-0.35172035	0.725052828	0.830256572	-6.96504471	ns
MMP15	0.003416498	0.08078203	0.349681526	0.726582474	0.831682017	-6.97072238	ns
PTPRB	-0.00208257	-0.0190152	-0.34889555	0.72717247	0.831996303	-6.97099483	ns
LY75	0.003009886	-0.02239787	0.348556723	0.727426904	0.831996303	-6.966982	ns
MUC2	0.00656326	0.023005902	0.347075817	0.728539078	0.832942094	-6.97105802	ns
SEMA7A	-0.00201929	0.004378665	-0.34646994	0.728994291	0.833136333	-6.97018665	ns
KCNC4	0.0040067	0.109911548	0.345634585	0.729622173	0.833527678	-6.959098	ns
FGF20	0.003047503	0.08274584	0.343649877	0.731114416	0.834665687	-6.95741758	ns
MME	0.006003925	0.083099284	0.343549261	0.731190009	0.834665687	-6.96594775	ns
ITGB2	0.002143919	0.001628109	0.342939175	0.731648973	0.834863229	-6.9624175	ns
CLEC4C	-0.00348094	-0.00674288	-0.34051958	0.733469944	0.836279507	-6.96972989	ns
RICTOR	0.003393106	0.077930817	0.340218368	0.73369684	0.836279507	-6.95858174	ns
FLT3	0.002331292	0.013453869	0.340148633	0.733749348	0.836279507	-6.95997077	ns
APLP1	-0.00378195	0.060587433	-0.33948455	0.734249394	0.836387226	-6.96777697	ns
MLLT1	-0.00474403	0.080641185	-0.33891149	0.734681145	0.836387226	-6.957863	ns
CORO6	-0.00254746	0.031450729	-0.33888224	0.734703163	0.836387226	-6.96139103	ns
HEBP1	0.003898252	0.021654101	0.337547619	0.735708824	0.837205676	-6.96176714	ns
VEGFC	-0.0040768	-0.02186157	-0.33621029	0.736716865	0.838026196	-6.97295265	ns
HAVCR1	0.004347042	-0.07149016	0.334787205	0.737790143	0.838920256	-6.9767615	ns
TEF	0.002937656	0.016446648	0.332343042	0.739634876	0.840690478	-6.96175605	ns
VEGFD	0.002650674	0.020503335	0.331175538	0.740516373	0.841127157	-6.97712637	ns
NECTIN1	0.00221865	0.008548204	0.331071386	0.740595179	0.841127157	-6.96162782	ns
STAU1	-0.00464235	0.141479601	-0.32970017	0.741631125	0.841976239	-6.96091821	ns
FCRL3	0.00390632	-0.02485141	0.328229946	0.742742357	0.842201413	-6.9648242	ns
TOR1AIP1	-0.0034831	0.021777381	-0.32766685	0.743168122	0.842201413	-6.96413012	ns
MORN4	-0.00530205	0.11814595	-0.3276584	0.743174524	0.842201413	-6.96158403	ns
PROC	0.001838576	-0.0123426	0.327553136	0.743254034	0.842201413	-6.97258973	ns
ITGB5	-0.00265584	0.024052368	-0.32752988	0.74327159	0.842201413	-6.97581143	ns
AMY2A	-0.00344109	0.014897039	-0.32672393	0.743881387	0.842565419	-6.94842229	ns
DNPH1	-0.00376609	0.001263057	-0.32532529	0.744939312	0.84317883	-6.97529251	ns
DPEP1	0.003539519	-0.05640025	0.325244533	0.745000473	0.84317883	-6.9703693	ns
DPP6	0.002390384	0.021483137	0.322713805	0.746916407	0.845019724	-6.97283523	ns
AOC3	-0.00211832	-0.00902145	-0.32052915	0.748571601	0.846283715	-6.9754484	ns
CSRP3	0.006329036	0.120643488	0.320474252	0.7486133	0.846283715	-6.96559981	ns
UHRF2	0.002297544	0.026746076	0.319959285	0.749003642	0.846397305	-6.96576342	ns
RNF4	0.004342297	0.127070525	0.317512238	0.750859297	0.848166014	-6.97344706	ns
COL28A1	0.0048657	0.109267738	0.316927909	0.751302681	0.848338681	-6.96820569	ns
CD3G	-0.00591976	0.247847091	-0.31522247	0.752597121	0.849471819	-6.96670923	ns
STX7	-0.00334563	-0.01084607	-0.31437033	0.753244146	0.849873612	-6.96697537	ns
REPS1	0.00251133	0.031770094	0.312239592	0.754862775	0.851370916	-6.96763772	ns
INSL3	0.004698879	0.640485637	0.310681061	0.756047274	0.852126446	-6.9834465	ns
ANGPT1	0.005809504	-0.07270749	0.310590335	0.756116309	0.852126446	-6.97615399	ns
PSME1	-0.00259032	-0.00260616	-0.30959497	0.756873203	0.852650368	-6.97660182	ns
ALMS1	0.002811525	0.03938938	0.308669505	0.757577155	0.853114266	-6.97619427	ns
ERCC1	-0.00204441	0.004867171	-0.30770714	0.75830945	0.853609713	-6.96787243	ns
TMPRSS11B	-0.00487557	0.181872052	-0.30703626	0.758820011	0.853855273	-6.97159579	ns
LTB	-0.00215379	0.01994736	-0.3065542	0.759186848	0.853938982	-6.98377122	ns
SLC1A4	-0.00331029	0.083987891	-0.30598391	0.75962111	0.854082913	-6.97158767	ns
KRT5	0.003238689	0.027792833	0.305617918	0.759899798	0.854082913	-6.97192224	ns

VIM	0.007714843	-0.15861497	0.304396127	0.760830322	0.854411255	-6.97232486	ns
RSPO1	-0.00230019	-0.0177006	-0.30397434	0.761151584	0.854411255	-6.97831285	ns
LELP1	0.0045789	0.057995305	0.303817325	0.761271259	0.854411255	-6.97257128	ns
CTLA4	-0.00184132	-0.0199997	-0.30369761	0.761362358	0.854411255	-6.98506537	ns
NBN	0.003350933	0.041310369	0.297334098	0.76621554	0.859197402	-6.97441251	ns
DCTPP1	-0.00197996	-0.01273703	-0.29733373	0.766215765	0.859197402	-6.98109491	ns
IL2RG	-0.00215992	0.025331718	-0.29615989	0.767111962	0.859606235	-6.98687978	ns
C1QTNF6	-0.00189856	0.015246609	-0.29594064	0.767279375	0.859606235	-6.98887382	ns
KHDC3L	-0.00402661	0.114029158	-0.2956997	0.767463512	0.859606235	-6.97146667	ns
SCN3B	-0.00290507	0.065220673	-0.29224734	0.770101539	0.862230251	-6.97599262	ns
ASS1	-0.004669	0.017910722	-0.29120532	0.770898313	0.862531798	-6.97448137	ns
CNTN1	0.001730207	0.024234468	0.291122348	0.770961641	0.862531798	-6.98980677	ns
LYSMD3	0.006107689	0.162853046	0.290191965	0.771673364	0.862997404	-6.97625833	ns
EPGN	0.002846447	0.100676841	0.289518951	0.772188165	0.863242512	-6.98880971	ns
EREG	-0.0050601	0.055358132	-0.28616098	0.774758722	0.865784718	-6.98222493	ns
CRHR1	-0.00386992	0.068512175	-0.28516045	0.775525138	0.866309642	-6.97795091	ns
CSPG4	0.00190508	0.004740762	0.283269043	0.776974393	0.867596645	-6.99101522	ns
SEPTIN8	-0.00185018	-0.00304184	-0.27952314	0.779847118	0.870471554	-6.99209984	ns
PLA2G2A	0.00340945	0.038841251	0.277245638	0.781595246	0.871801018	-6.98714931	ns
PTPN9	0.002191632	0.02560713	0.277193568	0.781635297	0.871801018	-6.97672549	ns
CD164L2	0.002066071	0.023539022	0.27654833	0.782130725	0.872020511	-6.98009437	ns
HYAL1	-0.00134844	-0.01284062	-0.27063823	0.786672961	0.876750018	-6.98559048	ns
PNLIP	0.003324809	0.042523442	0.270040689	0.787132551	0.876927527	-6.99402635	ns
PFDN4	-0.00302357	0.061249285	-0.26889875	0.788011293	0.877571692	-6.97897316	ns
PDXDC1	-0.00300718	0.082029105	-0.26516745	0.79088411	0.880435227	-6.97996172	ns
CDK1	-0.00199562	-0.01174814	-0.26446796	0.79142298	0.880699352	-6.98014551	ns
DDX1	0.002701651	0.089173718	0.263124775	0.792457914	0.88151509	-6.99587714	ns
ATF4	0.001864657	0.019725045	0.26201742	0.793311604	0.881934582	-6.98078553	ns
CCL17	-0.00521892	-0.08126845	-0.26185198	0.793439091	0.881934582	-6.99015334	ns
CRYBB2	-0.00354432	0.138784028	-0.26093961	0.794142548	0.882380609	-6.99755513	ns
GAD1	0.001406736	-0.00547326	0.259784287	0.795033732	0.882450699	-6.98136358	ns
ENPP5	0.002512163	-0.014431	0.259774235	0.795041396	0.882450699	-6.99497798	ns
TLR3	-0.00352049	-0.12203715	-0.2595574	0.795208637	0.882450699	-6.9988719	ns
OSTN	0.002263958	0.042508414	0.259290725	0.795414466	0.882450699	-6.98468196	ns
INSR	0.000865056	0.002053235	0.256361832	0.797674964	0.884106131	-6.98339938	ns
EXOSC10	0.003130434	0.148431382	0.256284303	0.797734824	0.884106131	-6.98396603	ns
BOLA1	0.002867533	0.01293374	0.256180512	0.797814951	0.884106131	-6.98580555	ns
STX1B	-0.00223579	0.03973848	-0.2549787	0.798743016	0.88453432	-6.98578208	ns
NPHS1	-0.00178004	0.008526345	-0.25489564	0.798807192	0.88453432	-6.98377124	ns
CD33	-0.00448595	-0.21778005	-0.25373865	0.799700849	0.885188203	-6.99709403	ns
PITHD1	0.004201675	0.174158417	0.252904958	0.800345127	0.885565658	-6.9816668	ns
SHD	0.004397097	0.212118268	0.250549229	0.80216607	0.88724429	-6.98445514	ns
CDA	-0.00244645	0.005324099	-0.24956934	0.802923729	0.887746039	-6.9993884	ns
B4GAT1	0.001359821	0.014502876	0.249134696	0.803259921	0.887781593	-6.99685166	ns
SUSD2	-0.00154892	0.000705283	-0.2483193	0.803890688	0.888142569	-6.99705292	ns
SFTPD	-0.00360335	-0.00148899	-0.24641956	0.805360768	0.889430198	-6.99483079	ns
NLGN2	0.002449607	0.090306197	0.244796371	0.806617435	0.890481251	-6.98863492	ns
CHIT1	-0.00819235	-0.33566846	-0.24434083	0.806970124	0.890533924	-6.99750564	ns
LYPLA2	-0.00535859	0.294517046	-0.24278122	0.808178189	0.891530152	-6.98912237	ns
IGDCC3	-0.00235488	0.07706132	-0.23962738	0.810622398	0.893348287	-6.98987716	ns
HNF1A	-0.00359282	0.140364752	-0.23926736	0.810901516	0.893348287	-6.98963443	ns
VSTM2L	-0.00302452	0.021654526	-0.23925495	0.810911152	0.893348287	-6.98989385	ns
AK1	0.003439724	0.007444219	0.239075672	0.811050106	0.893348287	-6.99659979	ns
GLT8D2	-0.00241632	0.061756019	-0.2385152	0.811484772	0.89349002	-6.99003631	ns
PHLDB2	0.004525166	0.156974196	0.237049927	0.81262129	0.894031202	-6.98696685	ns
CTSB	-0.00284246	0.074758993	-0.23669604	0.812895774	0.894031202	-6.9978609	ns
ATP5F1D	0.002635634	0.04692515	0.236548533	0.813010275	0.894031202	-6.98879121	ns
SCGB3A2	0.004335484	0.052983151	0.236302641	0.813200984	0.894031202	-6.99957859	ns
ARID4B	0.002154821	0.039316032	0.233567044	0.815324288	0.895692459	-6.99119517	ns
TSPAN7	-0.00465414	0.292569993	-0.23258169	0.816089406	0.895692459	-6.99152771	ns
NME3	-0.00117944	0.003821683	-0.23246913	0.816176762	0.895692459	-6.99949389	ns
GLP1R	-0.00174964	0.040982294	-0.23246137	0.816182843	0.895692459	-6.99155547	ns
F12	0.001987558	-0.05682967	0.232380239	0.816245765	0.895692459	-7.00429255	ns
CDH22	-0.0013506	0.013496106	-0.23106752	0.817265442	0.896474489	-6.99697501	ns
CALY	0.001753903	0.022474385	0.230357901	0.817816806	0.896532387	-6.99022513	ns
MSLNL	-0.00174055	0.029639386	-0.23020925	0.817932288	0.896532387	-6.99174413	ns

FCN1	-0.00263281	-0.06135624	-0.22884466	0.818992682	0.897175101	-7.00311928	ns
TRIM26	-0.00261767	0.028640413	-0.22866404	0.819133157	0.897175101	-6.98890424	ns
ADAMTS16	-0.00157352	0.003784119	-0.22701442	0.820415613	0.89816596	-6.9927253	ns
NDRG1	-0.00348105	0.134455926	-0.22670912	0.820653007	0.89816596	-6.99276114	ns
MPRIP	-0.00204596	-0.02048133	-0.2246551	0.822250571	0.899577246	-7.0047705	ns
HMGCS1	0.001994088	0.023525376	0.222444684	0.823970752	0.901121571	-6.99200268	ns
FABP6	-0.00262623	0.047857735	-0.22125108	0.824899934	0.901800003	-6.99400698	ns
SHH	0.001965324	0.006178161	0.220223749	0.825699892	0.902243856	-6.99194338	ns
ARHGAP30	0.001824837	0.08474333	0.219574033	0.826205852	0.902243856	-6.99954475	ns
CFHR4	0.003263859	-0.10948649	0.219539256	0.826232901	0.902243856	-7.00633365	ns
RIPK4	0.003029612	0.172018718	0.218188337	0.827285333	0.903055392	-6.99293308	ns
DCUN1D2	0.003586404	0.144878423	0.215989591	0.828998794	0.904587622	-6.99169999	ns
BSND	-0.00153307	0.025292972	-0.21505472	0.829727574	0.904716374	-6.9918999	ns
LAG3	-0.00188572	0.011541399	-0.21484353	0.829892178	0.904716374	-7.00160521	ns
ATXN2	-0.00497678	0.226510673	-0.21452269	0.830142373	0.904716374	-6.99553338	ns
RAC3	0.002991529	0.160196769	0.214248555	0.830356124	0.904716374	-6.9952634	ns
FAM171A2	0.006867275	0.39026599	0.210871935	0.832989972	0.907247564	-7.01026736	ns
CHRM1	-0.00160219	0.020869204	-0.20937976	0.834154614	0.907903786	-6.99628664	ns
PLA2G10	-0.0027828	0.023106515	-0.20930098	0.834216076	0.907903786	-7.0024224	ns
ANXA1	0.001846843	-0.02239906	0.208402532	0.834917499	0.907903786	-6.99378533	ns
CR1	-0.00153011	-0.00605057	-0.20826603	0.835024009	0.907903786	-7.00640604	ns
SYAP1	-0.00241359	0.062045785	-0.20810842	0.835147113	0.907903786	-6.99688109	ns
C1QTNF5	0.001600636	0.003171889	0.20680024	0.836168649	0.908092604	-6.99715036	ns
TRAF3	-0.00158902	0.046941262	-0.20643682	0.836452511	0.908092604	-6.99486152	ns
DDX39A	0.000939528	0.007271949	0.20631391	0.836548507	0.908092604	-6.99488669	ns
FABP2	-0.00355453	0.057176035	-0.20600744	0.836787837	0.908092604	-7.00390224	ns
CBX2	-0.00167662	0.065873713	-0.20589496	0.836875753	0.908092604	-6.9955194	ns
NT5E	-0.00249835	0.037834143	-0.20324636	0.838945154	0.909999944	-7.00854312	ns
PPP1R12B	0.002992832	0.110767768	0.200850688	0.840818003	0.911639535	-7.00345027	ns
LRTM1	-0.00171669	0.042338354	-0.20042982	0.841147139	0.911639535	-6.99810622	ns
TP53INP1	-0.00144031	0.047242235	-0.19966716	0.841743592	0.911639535	-6.99851411	ns
COL4A1	0.001742192	0.018352175	0.198931694	0.842318819	0.911639535	-7.00532385	ns
FGF2	-0.00294399	0.05881199	-0.19877238	0.842443439	0.911639535	-7.00765876	ns
CLEC10A	0.001557126	-0.01148855	0.198729789	0.842476767	0.911639535	-7.00456247	ns
F13B	-0.00103774	-0.01699364	-0.19850831	0.842650006	0.911639535	-7.00800208	ns
TIMP3	-0.0057165	0.089397347	-0.19811927	0.842954364	0.911639535	-7.00898587	ns
CYP24A1	-0.00344443	0.218778828	-0.19679482	0.843990765	0.912422448	-6.99733734	ns
SEPTIN3	-0.00298655	-0.00302157	-0.19455303	0.845745424	0.913980991	-7.01355034	ns
CLSPN	-0.00153092	0.036394742	-0.19404015	0.846147068	0.914050783	-6.99825923	ns
PTPRF	0.001205308	-0.01171792	0.19367096	0.846436068	0.914050783	-7.01324945	ns
VCPKMT	0.003471482	0.200195614	0.193038751	0.84693123	0.91424739	-6.99635693	ns
GALNT7	0.000873901	-0.00772705	0.191484647	0.848148449	0.915019422	-7.00631436	ns
GAGE2A	-0.00248619	0.102086336	-0.19132553	0.848273142	0.915019422	-6.99668359	ns
ZNF174	0.001426901	0.051263643	0.18995334	0.849348258	0.915227084	-6.99810289	ns
OLFM4	-0.00481399	-0.15120375	-0.18953021	0.849679923	0.915227084	-6.98189921	ns
IFNW1	0.001368607	0.040090482	0.189505656	0.849699081	0.915227084	-6.99873423	ns
IL1B	-0.00232922	0.051392367	-0.1894415	0.849749364	0.915227084	-6.99769513	ns
DSG2	-0.00111005	0.024864908	-0.18907973	0.850032826	0.915227084	-7.00990947	ns
RPGR	0.001624959	0.026118205	0.187726476	0.851093594	0.91549808	-7.00055244	ns
TPBGL	-0.001608	0.053055573	-0.18739657	0.851352224	0.91549808	-7.00061383	ns
SCGB3A1	-0.00098414	0.007533294	-0.18731503	0.851416146	0.91549808	-7.00096015	ns
LILRA6	-0.0028098	-0.08383911	-0.18715874	0.851538625	0.91549808	-7.01401545	ns
PLA2G7	-0.00127589	-0.01671476	-0.18638769	0.852143204	0.915583654	-7.01041099	ns
UROD	-0.0026855	0.00481737	-0.18624488	0.852255222	0.915583654	-7.00115848	ns
KRT17	0.001482454	-0.00040967	0.185857666	0.852558889	0.915583654	-6.99941354	ns
KIR3DL2	-0.00322402	0.221900985	-0.18300908	0.854793362	0.917309484	-6.99822795	ns
SF3B4	-0.00304155	0.043033013	-0.18300794	0.854794214	0.917309484	-7.00788938	ns
S100P	-0.00214163	0.033779393	-0.18178021	0.855757663	0.917623743	-7.00189899	ns
KIAA1549L	-0.00105102	0.009595429	-0.18159548	0.855902604	0.917623743	-7.00710391	ns
PXDNL	0.002156856	0.078730815	0.181433354	0.85602982	0.917623743	-7.01406764	ns
SIGLEC9	0.001169651	-0.02995732	0.179213892	0.857772098	0.919101618	-7.01644088	ns
CD58	-0.00069226	-0.00246532	-0.17887526	0.858038017	0.919101618	-7.00837214	ns
INPP5J	-0.00152813	0.024664776	-0.17765013	0.85900007	0.919794722	-7.01667216	ns
TMEM132A	-0.00230485	0.003499861	-0.17676516	0.859695231	0.920101702	-7.00189269	ns
ZP4	-0.00123341	0.038133797	-0.17648289	0.859916968	0.920101702	-6.9993918	ns
PCDH1	0.000783008	0.012253928	0.175466323	0.860715526	0.920618804	-7.0157146	ns

LCAT	0.000825483	-0.02928045	0.173816557	0.862011905	0.921421039	-7.01521871	ns
PSMG4	0.002096628	0.064253249	0.173708786	0.862096671	0.921421039	-7.00103353	ns
HIP1	-0.00118307	0.038352973	-0.17091989	0.864289058	0.92342629	-7.00205743	ns
SRPX	-0.00121277	-0.02342491	-0.17021721	0.864841539	0.923678601	-7.01701971	ns
GSTT2B	0.00830035	-0.51650693	0.169524034	0.865386682	0.923922892	-7.01708126	ns
AMFR	-0.00241299	0.073026188	-0.16797555	0.866604767	0.924885204	-7.00426142	ns
PAEP	0.004983412	0.603761557	0.166651242	0.867646679	0.925262734	-7.01037777	ns
SAP18	0.002497869	0.092825761	0.166499999	0.867765689	0.925262734	-7.00971098	ns
PRKD2	0.001622501	0.08054571	0.166317769	0.86790912	0.925262734	-7.00282737	ns
CEND1	0.001980402	0.07432179	0.165612811	0.868463885	0.925431651	-7.00475713	ns
PREB	-0.00106534	0.001283572	-0.16511639	0.868854581	0.925431651	-7.00474626	ns
TRAF3IP2	-0.00158538	0.067928226	-0.16454804	0.869301943	0.925431651	-7.00311785	ns
ENO2	-0.00239121	0.024600322	-0.1645056	0.869335281	0.925431651	-7.01891076	ns
DDA1	0.001368131	0.034767663	0.164055247	0.869689858	0.925471714	-7.00319818	ns
CDH15	-0.0020141	0.121763737	-0.16331264	0.870274416	0.925756392	-7.0178205	ns
RLN2	-0.00413018	0.209122159	-0.16279753	0.870680031	0.925850579	-7.00446893	ns
HBEGF	0.002615227	-0.02610994	0.16125834	0.871892038	0.926801875	-7.01870542	ns
HSPB6	-0.0013142	-0.02650018	-0.15901028	0.873662844	0.928346253	-7.02043644	ns
LAMA1	-0.00171684	0.125759385	-0.15584459	0.876157615	0.930002121	-7.00450121	ns
SEPTIN7	-0.00171098	0.063744073	-0.15583778	0.876162947	0.930002121	-7.01141614	ns
CLEC4G	0.001153768	-0.01516147	0.15582038	0.876176656	0.930002121	-7.01521571	ns
TNR	-0.00145262	0.04344359	-0.15503204	0.876798106	0.930323572	-7.01223223	ns
PRL	0.002446534	0.108852202	0.154169452	0.877478134	0.93070692	-7.02054522	ns
DGCR6	-0.0013793	0.039580816	-0.15341974	0.878069333	0.930824815	-7.00668697	ns
WFIKKN2	0.001216935	0.029312183	0.153219952	0.878226837	0.930824815	-7.01465944	ns
COL4A4	-0.00164237	-0.0504766	-0.15230577	0.878947787	0.931250921	-7.01116129	ns
ADAMTS13	-0.00074446	0.008123303	-0.15037786	0.880468464	0.932506179	-7.02064043	ns
RBM19	-0.00145332	0.061012731	-0.14952537	0.881141099	0.932506179	-7.00545851	ns
AGBL2	-0.00282077	-0.08226005	-0.1492006	0.881397338	0.932506179	-7.00732039	ns
ANGPTL7	-0.00116698	-0.01204065	-0.14912148	0.881459715	0.932506179	-7.02194844	ns
GSN	0.000556018	0.004596979	0.14853009	0.881926383	0.932506179	-7.01764861	ns
CRTAC1	0.000974292	-0.03935703	0.148375132	0.882048653	0.932506179	-7.02093732	ns
SLK	-0.00243817	0.186272559	-0.14726274	0.882926587	0.932592172	-7.01270567	ns
CDC25A	-0.00124944	0.022917025	-0.14712338	0.88303661	0.932592172	-7.00410498	ns
CD14	-0.00130835	-0.0185986	-0.14705812	0.883088136	0.932592172	-7.00022777	ns
ENOX2	0.000831707	0.017814115	0.146284875	0.883698467	0.932899322	-7.00593405	ns
SPARC	-0.00226688	-0.10878091	-0.14490419	0.884788406	0.933712377	-7.01862158	ns
SCP2	0.002468731	0.213841977	0.144136972	0.885394241	0.934014156	-7.00706289	ns
RBM17	-0.00221639	0.027297952	-0.14292883	0.886348248	0.934295216	-7.02219545	ns
WDR46	-0.00110379	0.006255983	-0.14258502	0.886619786	0.934295216	-7.02031355	ns
SNED1	0.000922146	0.001627895	0.142357269	0.886799715	0.934295216	-7.00734	ns
PDCL2	0.001552535	0.067610293	0.142178998	0.886940527	0.934295216	-7.00481458	ns
IL4	0.002765067	-0.15304078	0.140420156	0.888329906	0.93540022	-7.00848965	ns
ACOX1	0.001504113	0.023873932	0.140040025	0.888630209	0.93540022	-7.0152284	ns
PPBP	-0.00240876	-0.07303779	-0.13933454	0.889187623	0.935649679	-7.01691881	ns
SMPDL3A	0.001965467	-0.0438468	0.137160133	0.890906017	0.937120161	-7.01847153	ns
HADH	0.001812738	0.121435639	0.135145264	0.892498832	0.938457541	-7.0074893	ns
TNXB	-0.00074084	0.005160074	-0.13366118	0.893672257	0.939353129	-7.01878399	ns
GTF2IRD1	0.002017853	0.201916723	0.132887074	0.894284441	0.939658355	-7.01470381	ns
ZCCHC8	-0.0016347	0.078328402	-0.13225089	0.894787622	0.939848869	-7.00787325	ns
MNDA	-0.00289577	0.086793132	-0.13145036	0.895420783	0.940175724	-7.01638544	ns
GPIHBP1	-0.00186483	-0.08396748	-0.12988043	0.896662715	0.941032984	-7.02186074	ns
FAM171B	-0.00071	0.026016658	-0.12960355	0.896881779	0.941032984	-7.02206682	ns
CLU	0.00082853	0.027943405	0.126784808	0.899112365	0.943034521	-7.02311159	ns
ESR1	0.001527078	0.099098763	0.126243442	0.899540913	0.94314523	-7.00809647	ns
COL1A1	0.000699916	0.012764636	0.125727367	0.899949367	0.943192871	-7.02401769	ns
RRP15	-0.00236479	0.006131283	-0.12523266	0.900341035	0.943192871	-7.01058357	ns
GABRA4	-0.00165188	0.120533067	-0.12496189	0.900555385	0.943192871	-7.01061717	ns
THAP12	0.002336139	0.18956309	0.123123628	0.902010852	0.944369121	-7.00587549	ns
SCARB1	0.001577291	0.101875395	0.122726532	0.90232529	0.944369121	-7.01078715	ns
S100A13	0.000875624	-0.01072207	0.122021932	0.902883279	0.944404526	-7.00861639	ns
BRME1	0.00103379	0.022215792	0.121779687	0.903075125	0.944404526	-7.01100671	ns
PPM1B	-0.00157779	0.110689739	-0.12145864	0.903329397	0.944404526	-7.00868443	ns
FH	0.00203081	0.175841777	0.116559254	0.90721088	0.948123039	-7.01162398	ns
LMOD1	-0.0007918	0.004095823	-0.11456998	0.908787456	0.949430902	-7.02581897	ns
CHL1	-0.00077092	0.032665862	-0.11325654	0.909828652	0.950178706	-7.02005956	ns

F2	-0.00034408	-0.00502788	-0.11259614	0.910352203	0.950385567	-7.02479694	ns
GGT5	0.000523309	-0.00087408	0.111638271	0.911111693	0.950838508	-7.01797359	ns
FBP1	-0.00181796	-0.01463605	-0.11107933	0.911554893	0.950867678	-7.01883673	ns
AOC1	0.001380674	0.084091405	0.110105552	0.912327128	0.950867678	-7.01225736	ns
UBQLN3	-0.00107751	0.065592933	-0.11006629	0.912358273	0.950867678	-7.01054009	ns
C1QBP	-0.00153556	0.112604731	-0.1099604	0.912442203	0.950867678	-7.02633254	ns
BLVRB	-0.00161833	0.066930919	-0.10783195	0.914130402	0.952287112	-7.02187893	ns
CEP112	-0.00096658	0.066259552	-0.10595593	0.915618735	0.953497399	-7.01098058	ns
PFDN6	-0.00141592	0.110482804	-0.10467063	0.91663856	0.953841077	-7.01260041	ns
CLSTN2	0.000885988	0.021206573	0.104360791	0.916884385	0.953841077	-7.02689732	ns
ITIH4	0.000550223	0.006965556	0.104304907	0.916928734	0.953841077	-7.0256893	ns
ATG4A	-0.0013784	0.029637334	-0.10357311	0.917509512	0.954105333	-7.01204771	ns
IL1RL1	-0.00104643	-0.03532132	-0.10176952	0.918940937	0.955014762	-7.024611	ns
REST	0.000763765	0.023897697	0.101629222	0.91905234	0.955014762	-7.00971898	ns
DYNC1H1	0.000990021	0.047359834	0.101234986	0.919365238	0.955014762	-7.02531619	ns
NPPB	0.002747334	-0.10626043	0.099397149	0.920824268	0.955920059	-7.01339086	ns
NUDT15	0.001044367	0.061754334	0.098915196	0.921206922	0.955920059	-7.01169616	ns
LRRC37A2	0.000867774	0.009153847	0.098900125	0.921218852	0.955920059	-7.02537762	ns
CIT	-0.00093449	0.040308686	-0.09681535	0.922874314	0.956944662	-7.0133856	ns
TOP1MT	-0.00098874	0.038744892	-0.09641923	0.923188908	0.956944662	-7.01023084	ns
GALNT10	-0.00058226	-0.01727252	-0.09641855	0.923189422	0.956944662	-7.01989395	ns
CD34	-0.00049895	0.007987352	-0.0954146	0.923986758	0.957431275	-7.02653731	ns
ADGRB3	0.000681649	0.013372381	0.093574811	0.92544815	0.958605391	-7.01877568	ns
CA2	0.001471464	0.022368739	0.092999967	0.925904806	0.958738309	-7.02119289	ns
TXN	0.000829825	0.030304596	0.091657243	0.926971576	0.95950266	-7.01766198	ns
DDX53	0.000678235	0.00335894	0.090848587	0.927614106	0.959530232	-7.01394111	ns
COL9A1	0.000894704	0.032680861	0.090796563	0.927655426	0.959530232	-7.02317233	ns
CLASP1	0.001351158	0.108088646	0.087778224	0.930054116	0.961309392	-7.01102043	ns
LYPD3	0.000642671	0.004749544	0.087760909	0.930067853	0.961309392	-7.02236145	ns
PDRG1	-0.00095813	0.06402353	-0.08738943	0.930363131	0.961309392	-7.01424698	ns
PADI4	-0.0013957	0.078538967	-0.08567114	0.931728987	0.962359238	-7.01392493	ns
CA3	0.001158987	0.016364658	0.085281966	0.932038331	0.962359238	-7.02736942	ns
ADD1	-0.00124057	0.017132858	-0.08458518	0.932592298	0.962392615	-7.01481746	ns
GRK5	-0.000957	0.073062347	-0.0844122	0.932729829	0.962392615	-7.01475731	ns
RALB	0.00051098	-0.01002296	0.082412906	0.93431947	0.963692283	-7.01263343	ns
DKK4	-0.00080369	0.009476416	-0.08069234	0.935687686	0.964501738	-7.01934128	ns
CLEC3B	-0.00035594	0.000759801	-0.0805953	0.935764871	0.964501738	-7.01575494	ns
CCL28	-0.00097719	0.041427269	-0.07749764	0.938228686	0.966523478	-7.02208667	ns
GNPDA1	-0.00051391	0.029462089	-0.07729691	0.938388376	0.966523478	-7.01442961	ns
BMP4	-0.00075234	0.039707954	-0.07412202	0.940914264	0.968680204	-7.02039418	ns
RTKN2	-0.00077442	0.068110995	-0.07383105	0.941145801	0.968680204	-7.01384582	ns
CPA4	0.000648494	-0.00519132	0.073013042	0.941796724	0.968868839	-7.00424228	ns
IL24	0.000773576	0.030004918	0.072766763	0.941992683	0.968868839	-7.01564487	ns
HCG22	0.0007902	0.06826037	0.071913824	0.942671445	0.96922557	-7.01398444	ns
IL10RA	0.000894849	0.098333949	0.070287377	0.94396586	0.970214822	-7.01582083	ns
PGM2	0.000863605	0.097148545	0.069266977	0.944778028	0.970707896	-7.01565541	ns
CYB5A	0.000991197	0.073296799	0.067129647	0.946479385	0.97196199	-7.01260719	ns
BCL7A	0.000864641	0.052545431	0.066735082	0.946793493	0.97196199	-7.01263339	ns
OGT	0.000884218	0.108604218	0.066479168	0.946997213	0.97196199	-7.0212725	ns
SOD3	-0.0008082	0.093856886	-0.06521041	0.948007313	0.972656836	-7.02809984	ns
THBS2	0.00048711	0.012417318	0.064355735	0.948687812	0.973013141	-7.02559334	ns
AMY1A_AMY1B	0.000652983	0.02286981	0.063275306	0.949548152	0.97332429	-6.99775086	ns
NTproBNP	-0.0013197	0.013750353	-0.06290423	0.949843584	0.97332429	-7.02299535	ns
UPK3A	0.00065193	0.034600832	0.062718906	0.949991173	0.97332429	-7.01641258	ns
APOL1	0.000748123	0.088976768	0.060606759	0.951673173	0.974493559	-7.02838725	ns
KLK4	-0.00078693	0.014642121	-0.06040789	0.951831553	0.974493559	-7.03117217	ns
PROCR	0.000434773	0.035921165	0.060028664	0.952133604	0.974493559	-7.01476238	ns
C7orf50	-0.00029291	0.021179467	-0.05929918	0.952714579	0.974714069	-7.02959652	ns
DHODH	-0.00057019	0.04496766	-0.05839574	0.95343418	0.974714069	-7.01485832	ns
BEX3	0.000559848	0.045650722	0.057961311	0.953780217	0.974714069	-7.01317613	ns
ESPL1	-0.00067328	0.103070526	-0.05795878	0.95378223	0.974714069	-7.01317628	ns
EDIL3	0.000451663	-0.00434516	0.05694389	0.954590647	0.974714069	-7.01668359	ns
YJU2	0.000382548	0.015210591	0.056682253	0.954799045	0.974714069	-7.02880726	ns
PSMC3	0.000357808	0.012868642	0.056518939	0.95492916	0.974714069	-7.01325804	ns
THBS4	0.000568472	-0.02449886	0.056405511	0.955019503	0.974714069	-7.02313674	ns
PRAME	-0.00027691	-0.00051939	-0.05279002	0.957899916	0.976650506	-7.01346025	ns

SDHB	-0.0007309	0.110010598	-0.05246981	0.958155031	0.976650506	-7.02886497	ns
ENPP7	-0.00100168	-0.08705465	-0.05223837	0.958339461	0.976650506	-7.00796806	ns
SEMA4C	-0.00031183	0.004560033	-0.05217154	0.958392692	0.976650506	-7.02315599	ns
BATF	-0.00091488	0.214586247	-0.05192497	0.958589161	0.976650506	-7.01669808	ns
IL17RB	-0.00058808	0.021288541	-0.04921778	0.960746391	0.978417875	-7.02035771	ns
EIF4EBP1	0.000708066	0.012086571	0.048907068	0.960993995	0.978417875	-7.02377185	ns
LEFTY2	-0.00054607	0.002759612	-0.04790301	0.961794161	0.978576834	-7.03107592	ns
SYNGAP1	0.000565895	0.080529715	0.047870126	0.961820382	0.978576834	-7.01722724	ns
EPB41L5	-0.00062227	0.057211143	-0.04743933	0.962163709	0.978585172	-7.0169192	ns
SAT2	-0.00065001	0.05538566	-0.04569877	0.963550941	0.979654857	-7.02242907	ns
SLC16A1	-0.00056263	0.072578494	-0.04415446	0.96478187	0.980564936	-7.01729216	ns
MEPE	-0.00023908	-0.0170716	-0.04354682	0.965266211	0.980715845	-7.02396681	ns
IL2	-0.00029183	-0.01581604	-0.04246917	0.966125249	0.98124721	-7.01737695	ns
SKIV2L	-0.00033184	0.03970892	-0.04156418	0.966846682	0.981638495	-7.01569298	ns
NTF4	-0.00035309	0.050154806	-0.04026914	0.96787909	0.982345131	-7.01656506	ns
EIF1AX	-0.00044276	0.097428929	-0.03957553	0.968432046	0.982564828	-7.03056421	ns
PNMA2	-0.00034426	0.013310812	-0.03748603	0.970097972	0.983839534	-7.01766696	ns
TRPV3	0.000379553	0.049848417	0.037154539	0.97036228	0.983839534	-7.01586518	ns
AGT	-0.00016472	0.029067909	-0.0360659	0.971230278	0.98437779	-7.02956444	ns
ARG2	-0.00030739	0.049651298	-0.03512882	0.971977476	0.984793279	-7.02978916	ns
ADAMTS8	0.000338831	0.03360228	0.034346013	0.972601677	0.984846426	-7.0284548	ns
DPP4	0.000217779	-0.0035965	0.033916517	0.972944174	0.984846426	-7.01441783	ns
MAP1LC3B2	-0.00043726	0.15389875	-0.03354482	0.973240573	0.984846426	-7.01599179	ns
ACAN	-0.0002089	-0.00876062	-0.03337117	0.973379036	0.984846426	-7.02091296	ns
LTBP2	-0.00021777	-0.01365268	-0.03257756	0.974011886	0.985145379	-7.03133481	ns
ZNF830	0.000517587	0.29561881	0.031110513	0.975181808	0.985987146	-7.02992123	ns
NMRK2	0.000250932	0.067304802	0.028896935	0.976947174	0.987430165	-7.01442847	ns
AMBN	-0.00016397	0.017408876	-0.0277688	0.977846918	0.987908837	-7.01788919	ns
LXN	-0.0003884	0.101115315	-0.02734801	0.978182525	0.987908837	-7.0210497	ns
SSNA1	-0.00028347	0.035008125	-0.02694773	0.978501772	0.987908837	-7.02987071	ns
MNAT1	-0.00023978	0.041180447	-0.02632443	0.978998917	0.987908837	-7.02312139	ns
DLL4	-0.00046729	0.096239804	-0.02618216	0.979112388	0.987908837	-7.01705362	ns
HRC	-0.00017462	0.043459423	-0.02528016	0.979831832	0.988293247	-7.01707665	ns
RANGAP1	0.000261044	0.059555221	0.023986732	0.98086351	0.988992213	-7.0170841	ns
B3GAT3	9.8308494339	0.005472928	0.023517626	0.981237691	0.98902798	-7.01627564	ns
OMD	-0.00021223	-0.00650596	-0.02194677	0.982490699	0.98994922	-7.02822264	ns
MYL4	0.000229656	0.067817221	0.021272289	0.98302873	0.99010462	-7.01716921	ns
PINLYP	-0.00028726	-0.06601671	-0.02070929	0.983477831	0.99010462	-7.01815415	ns
TDGF1	0.000652279	0.194851732	0.020478219	0.983662159	0.99010462	-7.01805133	ns
NPTX2	0.000133893	-0.01579928	0.019949826	0.984083655	0.990128652	-7.03063346	ns
ALPI	0.000498253	0.218777755	0.019598144	0.984364204	0.990128652	-7.01581225	ns
TNFSF11	-0.00023353	0.033923726	-0.01708656	0.986367785	0.991657418	-7.02711793	ns
DUOX2	-0.0002699	0.152954815	-0.0168415	0.986563287	0.991657418	-7.01815155	ns
MMP1	0.000323726	-0.08202117	0.015853657	0.987351356	0.991838475	-7.00372043	ns
INSL5	0.000309446	0.07745515	0.015764146	0.987422756	0.991838475	-7.03010772	ns
FZD10	0.000146179	0.078663035	0.012020265	0.990409606	0.994496578	-7.01796399	ns
BMP6	8.0901644579	-0.01019986	0.008918352	0.992884397	0.996278337	-7.01752499	ns
LSM8	-0.00015699	0.284035071	-0.00879363	0.992983901	0.996278337	-7.02342684	ns
MYOM1	-7.90E-05	0.053938588	-0.00828464	0.993389995	0.996278337	-7.01833003	ns
PER3	-0.00019296	0.093361329	-0.00793315	0.993670432	0.996278337	-7.01597157	ns
LRRC38	7.1602661528	0.009561434	0.007308585	0.994168741	0.996278337	-7.01800917	ns
CYTH3	0.000110217	0.122892007	0.007230315	0.994231189	0.996278337	-7.01481678	ns
FCGR2B	8.0637117217	0.025043393	0.006329586	0.994949839	0.996379566	-7.01734966	ns
NUDT2	-7.70E-05	0.044004771	-0.00624834	0.995014663	0.996379566	-7.02448708	ns
GALNT5	-1.53E-05	-0.00234879	-0.00280139	0.997764856	0.998791011	-7.0313928	ns
IKZF2	-1.70E-05	0.245988819	-0.00082966	0.999338037	0.999863924	-7.01736919	ns
SMAD5	-8.09E-07	-0.03768428	-0.00017902	0.999857162	0.999863924	-7.02450644	ns
ACTA2	-1.59E-06	0.001956434	-0.00017055	0.999863924	0.999863924	-7.01829225	ns
