# Supplementary material for: Artificial intelligence driven definition of food preference endotypes in UK Biobank volunteers is associated with distinctive health outcomes and blood based metabolomic and proteomic profiles
Source: J Transl Med. 2024 Oct 1;22:881. doi: 10.1186/s12967-024-05663-0 (PMC11443809; doi:10.1186/s12967-024-05663-0)
Supplement: Supplementary file 4 — Supplementary Material 4. [file 12967_2024_5663_MOESM4_ESM.pdf]

Supplementary information for

Artificial intelligence driven definition of food preference endotypes in UK Biobank volunteers is associated with distinctive health outcomes and blood based metabolomic and proteomic profiles

INDEX

Supplementary Table 1. Study population characteristics of whole UK Biobank cohort and participants with FPQ
Supplementary Table 2: Exploratory Factor Analysis (EFA) results
Supplementary Table 3: Dimension of FPQ items based on Exploratory Factor Analysis
Supplementary Table 4: Actual daily nutrient intake for each food preference groups
Supplementary Table 5: Blood biochemistry and blood count for each food preference groups
Supplementary Table 6: pairwise group tests for metabolomics differential expression for Volcono3D plot
Supplementary Table 7: Limma differential expression for metabolomics data
Supplementary Table 8: Limma differential expression for proteomics data
Supplementary Table 9: Multinomial logistic regression for differentially expressed protein adjusted for age, sex and BMI
Supplementary Table 10: GO Term from Enrichment analysis of differentially expressed protein
Supplementary Table 11: Relative Risk of chronic diseases
Supplementary Data 1: UK Biobank Field ID
Supplementary Data 2: List of the 41 chronic conditions included in the definition of multimorbidity and its ICD10 code use for analysis.

Supplementary Data 1: UK Biobank Field ID

Field ID	Description
31	Sex
21003	Age when attended assessment centre
21001	Body mass index (BMI)
23099	Body fat percentage
21002	Weight
6138	Qualifications
22189	Townsend deprivation index at recruitment
22032	IPAQ activity group
20116	Smoking status
20600	Liking for adding salt to foods
20601	Liking for aniseed
20602	Liking for apple juice
20603	Liking for apples
20604	Liking for asparagus
20605	Liking for aubergine
20606	Liking for avocados
20607	Liking for bacon
20608	Liking for baked/steamed fish
20609	Liking for bananas
20610	Liking for barbequed or grilled meat
20611	Liking for beef steak
20612	Liking for beetroot
20613	Liking for bell pepper
20615	Liking for biscuits
20616	Liking for bitter foods
20617	Liking for bitter/ale
20618	Liking for black olives
20619	Liking for black pepper
20620	Liking for blue cheese
20621	Liking for bolognese sauce
20622	Liking for broad beans
20623	Liking for broccoli
20624	Liking for brown rice
20625	Liking for brussel sprouts
20626	Liking for burgers (meat)
20627	Liking for burn of spicy foods
20628	Liking for butter on bread
20629	Liking for butternut squash
20630	Liking for cabbage
20631	Liking for cake
20632	Liking for cake icing
20633	Liking for capers
20634	Liking for cauliflower
20635	Liking for cereal/granola bar
20636	Liking for cheesecake

20637	Liking for cherries
20638	Liking for chicken
20639	Liking for chilli pepper
20640	Liking for chips/french fries
20642	Liking for cod
20643	Liking for coffee with sugar
20644	Liking for coffee without sugar
20645	Liking for coriander
20646	Liking for corn flakes
20647	Liking for cream
20648	Liking for croissant
20649	Liking for cucumber
20650	Liking for curry
20651	Liking for dairy products
20652	Liking for dark chocolate
20653	Liking for diet fizzy drinks
20654	Liking for dried fruit
20655	Liking for eggs
20658	Liking for extra virgin olive oil
20659	Liking for fatty foods
20660	Liking for fresh tomatoes
20661	Liking for fried chicken
20662	Liking for fried/battered fish
20663	Liking for fruit
20664	Liking for garlic
20665	Liking for gherkins
20666	Liking for globe artichoke
20667	Liking for goat's cheese
20671	Liking for grapefruit
20672	Liking for green olives
20673	Liking for haddock
20674	Liking for ham
20675	Liking for hard cheese
20676	Liking for herring
20677	Liking for honey
20678	Liking for horseradish/wasabi
20679	Liking for ice cream
20680	Liking for jam
20681	Liking for kiwi fruit
20682	Liking for lager
20683	Liking for lamb
20684	Liking for lemons
20685	Liking for lentils/beans
20686	Liking for liver
20687	Liking for mackerel
20688	Liking for marzipan
20689	Liking for mayonnaise
20690	Liking for melon

20691 Liking for milk chocolate
20692 Liking for mushrooms
20693 Liking for onions
20694 Liking for orange juice
20695 Liking for oranges
20696 Liking for pasta
20697 Liking for pears
20698 Liking for pizza
20699 Liking for plain yogurt
20700 Liking for plums
20701 Liking for pollock
20702 Liking for pork chop
20703 Liking for porridge
20704 Liking for potato crisps
20705 Liking for potatoes
20706 Liking for prawns
20707 Liking for raw carrots
20708 Liking for red meat
20709 Liking for red wine
20710 Liking for regular (non-diet) fizzy drinks
20711 Liking for roast chicken
20712 Liking for salad dressing
20713 Liking for salad leaves
20714 Liking for salami
20715 Liking for salmon
20716 Liking for salty foods
20717 Liking for salty pretzels
20718 Liking for sardines
20719 Liking for sausages (meat)
20720 Liking for savoury biscuits
20721 Liking for shellfish (other than prawns)
20722 Liking for skimmed milk
20723 Liking for smoked fish
20724 Liking for soft cheese
20725 Liking for soy sauce
20726 Liking for soya milk
20727 Liking for spicy foods
20728 Liking for spinach
20729 Liking for spirits
20730 Liking for strawberries
20731 Liking for sweet coffee house drinks
20732 Liking for sweet foods
20734 Liking for tea with sugar
20735 Liking for tea without sugar
20737 Liking for tinned tuna
20736 Liking for tomato ketchup
20738 Liking for turnip (white)
20739 Liking for vegetables

20740	Liking for vinegar
20742	Liking for whisky
20743	Liking for white bread
20744	Liking for white rice
20745	Liking for white wine
20746	Liking for whole grain breakfast cereal
20747	Liking for whole milk
20748	Liking for wholemeal bread
26013	Carbohydrate
26002	Energy
26017	Englyst fibre
26008	Fat
26012	Free sugar
26005	Protein
26014	Saturated fatty acids
26011	Total sugars
30620	Alanine aminotransferase
30600	Albumin
30610	Alkaline phosphatase
30630	Apolipoprotein A
30640	Apolipoprotein B
30650	Aspartate aminotransferase
30710	C-reactive protein
30680	Calcium
30690	Cholesterol
30700	Creatinine
30720	Cystatin C
30660	Direct bilirubin
30730	Gamma glutamyltransferase
30740	Glucose
30750	Glycated haemoglobin (HbA1c)
30760	HDL cholesterol
30770	IGF-1
30780	LDL direct
30790	Lipoprotein A
30800	Oestradiol
30810	Phosphate
30820	Rheumatoid factor
30830	SHBG
30850	Testosterone
30840	Total bilirubin
30860	Total protein
30870	Triglycerides
30880	Urate
30670	Urea
30890	Vitamin D
23474	3-Hydroxybutyrate
23475	Acetate

23476 Acetoacetate
23477 Acetone
23460 Alanine
23479 Albumin
23440 Apolipoprotein A1
23439 Apolipoprotein B
23484 Cholesterol in Chylomicrons and Extremely Large VLDL
23526 Cholesterol in IDL
23561 Cholesterol in Large HDL
23533 Cholesterol in Large LDL
23498 Cholesterol in Large VLDL
23568 Cholesterol in Medium HDL
23540 Cholesterol in Medium LDL
23505 Cholesterol in Medium VLDL
23575 Cholesterol in Small HDL
23547 Cholesterol in Small LDL
23512 Cholesterol in Small VLDL
23554 Cholesterol in Very Large HDL
23491 Cholesterol in Very Large VLDL
23519 Cholesterol in Very Small VLDL
23485 Cholesteryl Esters in Chylomicrons and Extremely Large VLDL
23418 Cholesteryl Esters in HDL
23527 Cholesteryl Esters in IDL
23417 Cholesteryl Esters in LDL
23562 Cholesteryl Esters in Large HDL
23534 Cholesteryl Esters in Large LDL
23499 Cholesteryl Esters in Large VLDL
23569 Cholesteryl Esters in Medium HDL
23541 Cholesteryl Esters in Medium LDL
23506 Cholesteryl Esters in Medium VLDL
23576 Cholesteryl Esters in Small HDL
23548 Cholesteryl Esters in Small LDL
23513 Cholesteryl Esters in Small VLDL
23416 Cholesteryl Esters in VLDL
23555 Cholesteryl Esters in Very Large HDL
23492 Cholesteryl Esters in Very Large VLDL
23520 Cholesteryl Esters in Very Small VLDL
23473 Citrate
23404 Clinical LDL Cholesterol
23481 Concentration of Chylomicrons and Extremely Large VLDL Particles
23430 Concentration of HDL Particles
23523 Concentration of IDL Particles
23429 Concentration of LDL Particles
23558 Concentration of Large HDL Particles
23530 Concentration of Large LDL Particles
23495 Concentration of Large VLDL Particles
23565 Concentration of Medium HDL Particles
23537 Concentration of Medium LDL Particles

23502	Concentration of Medium VLDL Particles
23572	Concentration of Small HDL Particles
23544	Concentration of Small LDL Particles
23509	Concentration of Small VLDL Particles
23428	Concentration of VLDL Particles
23551	Concentration of Very Large HDL Particles
23488	Concentration of Very Large VLDL Particles
23516	Concentration of Very Small VLDL Particles
23478	Creatinine
23443	Degree of Unsaturation
23450	Docosahexaenoic Acid
23486	Free Cholesterol in Chylomicrons and Extremely Large VLDL
23422	Free Cholesterol in HDL
23528	Free Cholesterol in IDL
23421	Free Cholesterol in LDL
23563	Free Cholesterol in Large HDL
23535	Free Cholesterol in Large LDL
23500	Free Cholesterol in Large VLDL
23570	Free Cholesterol in Medium HDL
23542	Free Cholesterol in Medium LDL
23507	Free Cholesterol in Medium VLDL
23577	Free Cholesterol in Small HDL
23549	Free Cholesterol in Small LDL
23514	Free Cholesterol in Small VLDL
23420	Free Cholesterol in VLDL
23556	Free Cholesterol in Very Large HDL
23493	Free Cholesterol in Very Large VLDL
23521	Free Cholesterol in Very Small VLDL
23470	Glucose
20280	Glucose-lactate
23461	Glutamine
23462	Glycine
23480	Glycoprotein Acetyls
23406	HDL Cholesterol
23463	Histidine
23465	Isoleucine
23405	LDL Cholesterol
23471	Lactate
23466	Leucine
23449	Linoleic Acid
23447	Monounsaturated Fatty Acids
23444	Omega-3 Fatty Acids
23445	Omega-6 Fatty Acids
23468	Phenylalanine
23437	Phosphatidylcholines
23434	Phosphoglycerides
23483	Phospholipids in Chylomicrons and Extremely Large VLDL
23414	Phospholipids in HDL

23525	Phospholipids in IDL
23413	Phospholipids in LDL
23560	Phospholipids in Large HDL
23532	Phospholipids in Large LDL
23497	Phospholipids in Large VLDL
23567	Phospholipids in Medium HDL
23539	Phospholipids in Medium LDL
23504	Phospholipids in Medium VLDL
23574	Phospholipids in Small HDL
23546	Phospholipids in Small LDL
23511	Phospholipids in Small VLDL
23412	Phospholipids in VLDL
23553	Phospholipids in Very Large HDL
23490	Phospholipids in Very Large VLDL
23518	Phospholipids in Very Small VLDL
23446	Polyunsaturated Fatty Acids
23472	Pyruvate
23402	Remnant Cholesterol (Non-HDL, Non-LDL -Cholesterol)
23448	Saturated Fatty Acids
20281	Spectrometer-corrected alanine
23438	Sphingomyelins
23400	Total Cholesterol
23401	Total Cholesterol Minus HDL-C
23436	Total Cholines
23464	Total Concentration of Branched-Chain Amino Acids (Leucine + Isoleucine + Valine)
23427	Total Concentration of Lipoprotein Particles
23415	Total Esterified Cholesterol
23442	Total Fatty Acids
23419	Total Free Cholesterol
23482	Total Lipids in Chylomicrons and Extremely Large VLDL
23426	Total Lipids in HDL
23524	Total Lipids in IDL
23425	Total Lipids in LDL
23559	Total Lipids in Large HDL
23531	Total Lipids in Large LDL
23496	Total Lipids in Large VLDL
23423	Total Lipids in Lipoprotein Particles
23566	Total Lipids in Medium HDL
23538	Total Lipids in Medium LDL
23503	Total Lipids in Medium VLDL
23573	Total Lipids in Small HDL
23545	Total Lipids in Small LDL
23510	Total Lipids in Small VLDL
23424	Total Lipids in VLDL
23552	Total Lipids in Very Large HDL
23489	Total Lipids in Very Large VLDL
23517	Total Lipids in Very Small VLDL

23411	Total Phospholipids in Lipoprotein Particles
23407	Total Triglycerides
23487	Triglycerides in Chylomicrons and Extremely Large VLDL
23410	Triglycerides in HDL
23529	Triglycerides in IDL
23409	Triglycerides in LDL
23564	Triglycerides in Large HDL
23536	Triglycerides in Large LDL
23501	Triglycerides in Large VLDL
23571	Triglycerides in Medium HDL
23543	Triglycerides in Medium LDL
23508	Triglycerides in Medium VLDL
23578	Triglycerides in Small HDL
23550	Triglycerides in Small LDL
23515	Triglycerides in Small VLDL
23408	Triglycerides in VLDL
23557	Triglycerides in Very Large HDL
23494	Triglycerides in Very Large VLDL
23522	Triglycerides in Very Small VLDL
23469	Tyrosine
23403	VLDL Cholesterol
23467	Valine
1072	olink_data
41270	Diagnoses - ICD10

Supplementary Data 2: List of the 41 chronic conditions included in the definition of multimorbidity and its ICD10 code use for analysis.

No	Chronic condition	ICD10 code
1	Anaemia	D5[0-3]
2	Angina pectoris	I20
3	Anxiety	F41
4	Asthma	J45
5	Atrial fibrilation	I48
6	Bronchiectasis	J47
7	Cancer	C[0-9]
8	Chronic kidney disease	N18
9	Chronic obstructive pulmonary disease	J44
10	Chronic sinusitis	J32
11	Cirrhosis	K74
12	Dementia	F0[1-3]
13	Depression	F3[2-3]
14	Dermatitis	L[2-3]
15	Diabetes	E1[0-4]
16	Epilepsy	G4[0-1]
17	Glaucoma	H4[0-2]
18	Heart failure	I50
19	Hepatitis	B1[5-9]
20	Hypertention	I1[0-5]
21	Inflammatory bowel disease	K5[0-2]
22	Irritable bowel syndrome	K58
23	Meningitis	G0[0-3]
24	Migraine	G43
25	Multiple schlerosis	G35
26	Myocardial infarction	I2[1-2]
27	Osteoporosis	M8[0-2]
28	Parkinson's disease	G2[0-1]
29	Peripheral vascular diseases	I73
30	Prostate problem	N4[0-2]
31	Rheumatoid arthritis	M0[5-6]
32	Schizophrenia	F2[0-9]
33	Stroke	I6[0-9]
34	Thyroid problem	E0[0-7]
35	Tuberculosis	A1[5-9]
36	Vestibular disorder	H8[1-3]
37	Constipation	K59
38	Dyspepsia	K3[0-1]
39	Diverticular disease of intestine	K5[7-8]
40	Endometriosis	N80
41	Hearing loss	H9[0-1]
